# PCNA-associated factor KIAA0101 transcriptionally induced by ELK1 controls cell proliferation and apoptosis in nasopharyngeal carcinoma: an integrated bioinformatics and experimental study

**DOI:** 10.18632/aging.102991

**Published:** 2020-04-09

**Authors:** Hu Zhao, Miaosheng Chen, Jie Wang, Gang Cao, Wei Chen, Jinke Xu

**Affiliations:** 1 Fujian Provincial Key Laboratory of Transplant Biology, Department of Urology, 900 Hospital of the Joint Logistics Team, Xiamen University, Fuzhou 350025, Fujian, P.R. China; 2 Office of Science Education, 900 Hospital of the Joint Logistics Team, Xiamen University, Fuzhou 350025, Fujian, P.R. China; 3 Pathology Department, Longyan First Hospital Affiliated to Fujian Medical University, Longyan 364000, Fujian, P.R. China; 4 Department of Oral and Maxillofacial Surgery, Medical School of Nanjing University, Nanjing 210002, Jiangsu, P.R. China

**Keywords:** KIAA0101, ELK1, proliferation, DNA replication, bioinformatics, nasopharyngeal carcinoma

## Abstract

KIAA0101, previously identified as PCNA-associated factor, is overexpressed among almost majority of human cancers and has emerged as an important regulator of cancer progression; however, its function in human nasopharyngeal carcinoma (NPC) remain unknown. Integrated bioinformatics approaches were employed to determine the KIAA0101 expressions in the NPC samples. Lentiviral vectors carrying KIAA0101 shRNA were constructed and stable transfected cells were validated by qRT-PCR and western blot. Cellular functions were then evaluated by MTT, colony formation, Brdu staining, and flow cytometry. Mechanistic studies were systematically investigated by UCSC Genome Browser, GEO, UALCAN, QIAGEN, PROMO and JASPAR, ChIP, and the cBioPortal, et al. The results showed that KIAA0101 ranked top overexpressed gene lists in GSE6631 dataset. KIAA0101 was highly expressed in NPC tissues and cell lines. Furthermore, knockdown of KIAA0101 significantly inhibited cell proliferation and DNA replication, promoted apoptosis and cell cycle arrest in vitro. Meanwhile, the mechanistic study revealed that MAP kinase phosphorylation-dependent activation of ELK1 may enhance neighbor gene expressions of KIAA0101 and TRIP4 by binding both promotor regions in the NPC cells. Taken together, our findings indicate that overexpression of KIAA0101 activated by MAP kinase phosphorylation-dependent activation of ELK1 may play an important role in NPC progression.

## INTRODUCTION

Nasopharyngeal carcinoma (NPC), one of the most common malignant tumors of head and neck cancer in southern China, has distinct racial and regional characteristics [1, 2]. More than 95% NPC belongs to poorly differentiated or undifferentiated cancer types, prone to early metastasis, which further leads to the death of patients with NPC [3, 4]. Despite the widespread employment and improvement of radiotherapy and chemotherapy, the five-year survival rate of NPC remains at 50%-60%, and the long-term survival of patients has not improved [5]. It is urgent to identify new molecular basis behind the pathogenesis of NPC and explore efficient therapeutic targets for NPC patients.

In the last decade, with the rapid advance of RNA-seq and omics technology, effective targets for the diagnosis or treatment of cancers may be dogged by bioinformatics analysis, which points the way for the follow-up experimental research [6]. Proliferating cell nuclear antigen (PCNA) is an evolutionarily well-conserved protein critically essential for DNA replication, cell cycle regulation and DNA damage repair response in eukaryotic cells [7]. KIAA0101 is a PCNA-associated factor by interacting with PCNA binding motif [8, 9]. Increasing evidence has revealed that KIAA0101 plays a multifunctional role in biological process regulations of human cancer development such as cell proliferation [10], migration [11], DNA repair [12], cell cycle [10], and chemoresistance [13]. *Masayo Hosokawa* et al elucidated that KIAA0101 was precisely regulated by the p53-p21 signal axis in pancreatic cancer [9]. In addition, *Neha Jain* group found that miR-197-5p suppressed proliferation, invasion, migration and induced cellular senescence of HT1080 fibrosarcoma cells by targeting KIAA0101 [14]. Our previous studies also indicated that frequently downregulated miR-30a-5p inhibited cell proliferative capacity by targeting PCLAF in prostate cancer cells [15]. Moreover, overexpression of KIAA0101 predicted poor prognosis and promoted the proliferation of rectal cancer [16], hepatocellular carcinoma [17], adrenal cancer [18], pancreatic cancer [9] and gastric cancer cells [19]. Therefore, KIAA0101 may act as an oncogenic role in the development of several cancers. However, whether KIAA0101 is involved in the oncogenesis of NPC and the molecular mechanisms by which KIAA0101 is regulated in NPC are unclear.

With the help of bioinformatics analysis and experimental study, our study indicated that KIAA0101 was overexpressed in NPC samples and that cell proliferation, apoptosis, cell cycle arrest and DNA replication were the primary biological functions of KIAA0101 in NPC cells. Increasing evidence showed that eukaryotic gene clusters within genomic neighborhoods were nonrandomly distributed, which may have co-expression, co-regulation, and co-functionality possibilities [20]. We found that TRIP4, clustered within the same genomic neighborhoods of KIAA0101, was identified to have the similar expression patterns in GDS2520 and TCGA HNSC samples, which may be co-regulated by the same transcriptional factor ELK1. As a key member of the Ets family and ternary complex factor (TCF) subfamily, ELK1 has influenced various steps of many tumor development largely through Ras-Raf-MAPK signaling cascade [21–23]. However, little is known about role of ELK1 in NPC. In this study, we showed that ELK1 was also highly expressed in HNSC samples, and ChiP assay further proved that KIAA0101 was transcriptionally induced by ELK1. Results from our study indicated that KIAA0101, activated by MAP kinase phosphorylation-dependent activation of ELK1, is a key regulator of cell proliferation, cell cycle arrest, and DNA replication in NPC.

## RESULTS

### Top ranked and highly expressed KIAA0101 in NPC samples

**Figure 1:**
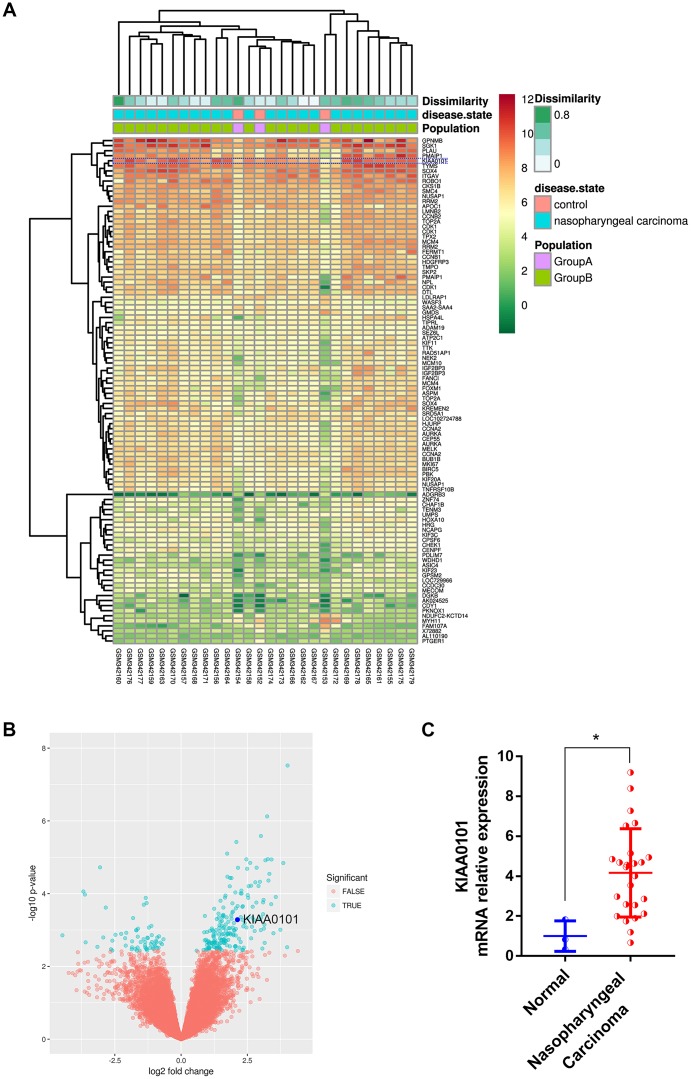
**Top ranked and highly expressed KIAA0101 in nasopharyngeal carcinoma dataset GDS3610.** (**A**) Heatmap showing top 100 ranking genes, based on GeoDiver analysis. (**B**) Volcano plot of differential gene expressions; KIAA0101 was marked by dark blue circle. (**C**) Relative mRNA expression of KIAA0101 in GDS3610. *P<0.05.

**Figure 2:**
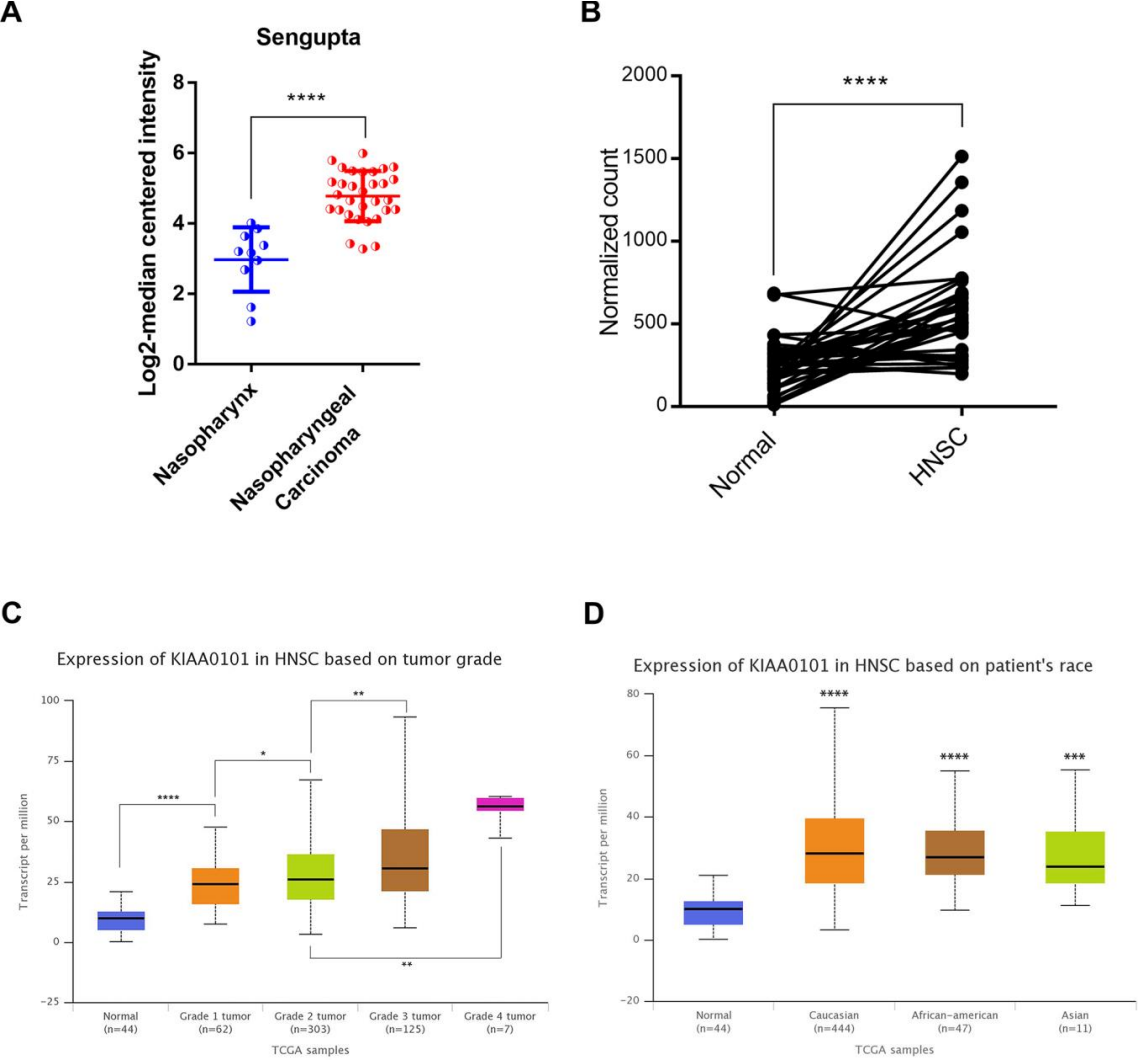
**The high-level expression of KIAA0101 in TCGA HNSC and Sengupta NPC samples.** (**A**) KIAA0101 mRNA expression was compared between normal and HNSC samples in 40 patients (****P<0.0001, paired t-test). (**B**) KIAA0101 mRNA was highly up-regulated in Sengupta NPC compared with normal samples. *P<0.05, **P<0.01, ****P<0.00001. (**C**) KIAA0101 RNA expression was positively correlated with tumor grade. (**D**) Expression of KIAA0101 in HNSC based on patient’s race.

In order to discover the critical genes involved in NPC progression, GDS3610 dataset containing 25 undifferentiated NPC samples and 3 normal controls were downloaded and analyzed by GeoDiver [24]. After normalization to get rid of biases in microarray data, results of heatmap and volcano plot showed that KIAA0101 was ranked top in differential gene lists (Figure 1A, 1B). TIMER analysis of KIAA0101 expressions across TCGA tumors showed that this gene was up-regulated in all tumors compared to normal tissues as long as normal data were available (Supplementary Figure 1, gray columns, ***p<0.001). Microarray data further revealed that KIAA0101 was over-expressed in the human NPC GDS3610 tissues, significantly (Figure 1C, *p<0.05). Then we expanded the sample quantity with Sengupta NPC samples and TCGA HNSC samples to make the result more persuasive. As Figure 2A showed that KIAA0101 was overexpressed dramatically in 31 Sengupta NPC samples compared with 10 normal healthy nasopharyngeal tissue specimens (****p<0.0001, Supplementary Table 1, 10, t-test). KIAA0101 mRNA expression was also upregulated significantly in 40 paired TCGA HNSC-normal samples (Figure 2B, Supplementary Table 2, 10, ****P<0.0001, paired t-test). Further analysis elucidated that this expression was positively correlated with patient’s tumor grade, and irrelevant with patient’s race (Figure 2C, 2D). However, unfortunately, there was no significant difference for the overall survival and disease-free survival Kaplan-Meier estimate among HNSC patients (Supplementary Figure 2). All data above indicates that KIAA0101 may be involved in the development of NPC.

### KIAA0101 knockdown inhibits cell proliferation and promotes cell apoptosis in vitro

**Figure 3:**
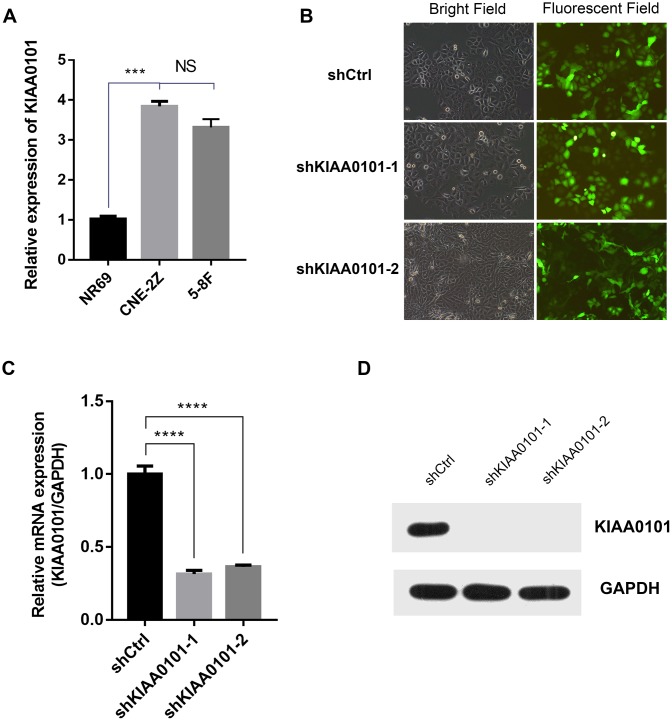
**Lentivirus mediated KIAA0101 downregulation is effective in NPCs.** (**A**) The mRNA expression level of KIAA0101 was detected with qRT-PCR in two NPC cells. Histogram is the average value (mean ± SD) of three independent experiments. (**B**) Representative bright field and fluorescent field graphs of CNE-2Z cells infected with two indicated lentiviruses are shown by GFP. The mRNA (**C**) and protein (**D**) levels of KIAA0101 were measured by qRT-PCR and Western blotting in CNE-2Z cells after lentivirus infections. All were done at least three independent experiments. ****P<0.00001.

**Figure 4:**
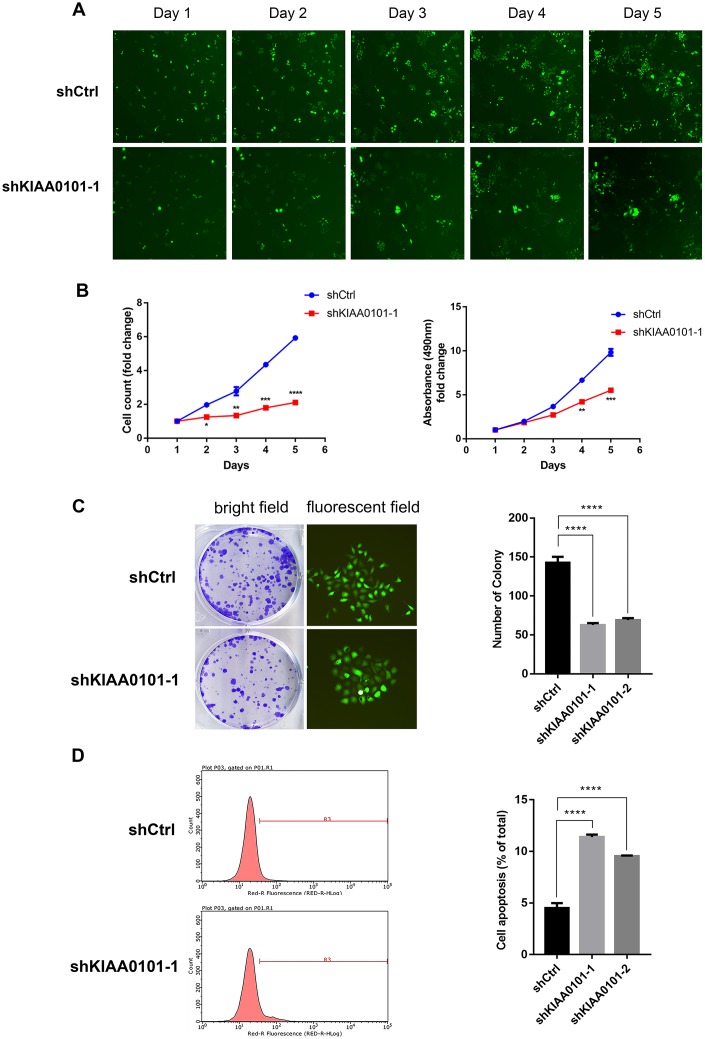
**Down-regulation of KIAA0101 inhibits the proliferation and promotes apoptosis of NPC cells.** (**A**) Reprehensive fluorescent graphs of shCtrl and shKIAA0101-1 NPC groups taken by Nexcelom Celigo Image Cytometer in five continuous days. (**B**) Statistical result of cell count/fold and growth curve. Data are presented as the mean ± SD from three independent experiments. **p<0.01, ***p<0.001, ****p<0.00001. (**C**) Downregulation of KIAA0101 suppressed colony formation ability of NPC cell line CNE-2Z (Left: bright field; Right: fluorescent field). (**D**) Apoptosis ratios of shKIAA0101 groups were increased compared with those in shCtrl group by flowcytometry. Histogram is the average cell apoptosis rate (mean ± SD) of three independent experiments. ****p<0.00001.

To further identify the role of KIAA0101 gene in NPC, we constructed shKIAA0101 and shCtrl lentivirus vectors to evaluate the specific cellular functions of NPC cells. As Figure 3A showed that KIAA0101 mRNA were both highly expressed in CNE-2Z and 5-8F cells compared with internal reference gene GAPDH, and CNE-2Z cell line was chosen for following experiments (if not stated otherwise). After PSCSI-GFP lentivirus infections (Figure 3B, ***p<0.001), mRNA and protein expressions of KIAA0101 were downregulated significantly in CNE-2Z cells (Figure 3C, 3D). Moreover, by employing the immunofluorescence staining, we found that cell proliferation rates of CNE-2Z cells were significantly reduced after shKIAA0101 lentivirus infections at different time points from day 1 to day 5, compared with shCtrl group (Figure 4A, 4B, **p<0.01, ***p<0.001, ****p<0.0001). As expected, similar results were also obtained by MTT assay (Figure 4B, **p<0.01, ***p<0.001) and colony formation assay (Figure 4C, ****p<0.0001). Flow cytometric analysis of apoptosis was carried out on CNE-2Z shKIAA0101 and shCtrl cells. Representative bar chart showed that number of apoptotic and necrotic cells was elevated significantly after shKIAA0101 lentivirus treatment (Figure 4D, ****p<0.0001). All data suggested that KIAA0101 played an essential role in mediating the proliferation rate and apoptosis ability of NPC cells.

### Depletion of KIAA0101 results in cell cycle arrest and decrease of DNA replication in vitro

**Figure 5:**
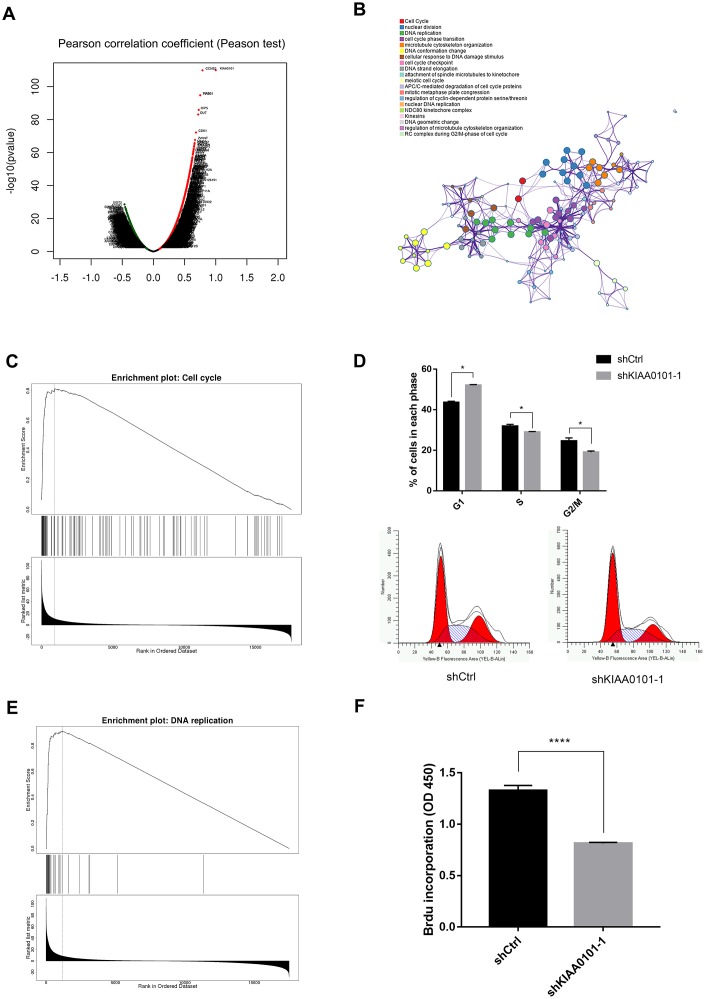
**Cell cycle and DNA synthesis process suppressed after downregulation of KIAA0101.** (**A**) LinkedOmics analysis of positively and negatively related genes with KIAA0101 in TCGA HNSC samples. (**B**) Metascape color clustering of positively correlated significant genes, *P*<0.0001, FDR(BH)<0.0001, Statistic>0). Each color box represents a biological process or pathway. (**C**) LinkeOmics GSEA KEGG module analysis of differentially expressed genes for associations between KIAA0101 expressions and Hiseq RNA expression profiles in TCGA HNSC samples revealed that cell cycle was positively enriched. (**D**) S and G2/M phases of shKIAA0101 groups were decreased compared with those in shCtrl group by flowcytometry. Histogram is the average ratio (mean ± SD) of three independent experiments. *P<0.005. (**E**) LinkeOmics GSEA KEGG analysis revealed that DNA replication was also identified with the strongest association with KIAA0101-higher expression. (**F**) BrdU incorporation in cultured CNE-2Z cells following control (shCtrl) and shKIAA0101 lentivirus infection. Histogram is the average ratio (mean ± SD) of three independent experiments. ****p<0.00001 by Student’s t test.

Previous experiments have determined the phenotypic change of CNE-2Z cells after KIAA0101 knockdown. So, what is the possible mechanism behind this phenomenon? MEM co-expression analysis of all ArrayExpress datasets and tumor only datasets with two KIAA0101 probes 202503_S_AT and 211713_X_AT showed that 175 positively co-expressed genes were found in both two datasets (Supplementary Figure 3A–3C, Supplementary Tables 3–5). Further Cytoscape KEGGscape analysis of these positively co-expressed genes with KIAA0101 revealed that signaling pathways including DNA replication, cell cycle, homologous recombination, et al were significantly enriched (Supplementary Figure 3D, 3E). To investigate the specific signaling pathway induced by KIAA0101 in NPC, LinkedOmics platform was employed to analyze co-expression genes with positive correlations (pearson test, r value > 0.2) with KIAA0101 in TCGA HNSC samples (Figure 5A, Supplementary Table 6), and Metascape enrichment result also showed that cell cycle (red dot) and DNA replication (green dot) may be induced by KIAA0101 in NPC (Figure 5B). Cell cycle (ES: 0.81484; NES: 2.4081) and DNA replication (ES: 0.91498; NES: 2.2868) were also significantly enriched by LinkedOmics GSEA analysis (Figure 5C, 5D left panel, Supplementary Figure 4). Flow cytometry-based cell cycle analysis showed that KIAA0101 depletion significantly decreased the percentage of CNE-2Z cells in S-phase and G2/M-phase (Figure 5C right panel, *p<0.05). BrdU incorporation assay showed that KIAA0101 knockdown significantly decreased the percentage of BrdU-positive CNE-2Z cells (Figure 5D right panel, ****p<0.0001). These results indicate that KIAA0101 may inhibit the proliferation of NPC cells through cell cycle arrest and stopping DNA replication process.

### Neighboring gene TRIP4 shows correlated co-expression pattern with KIAA0101 in HNSC (including NPC)

**Figure 6:**
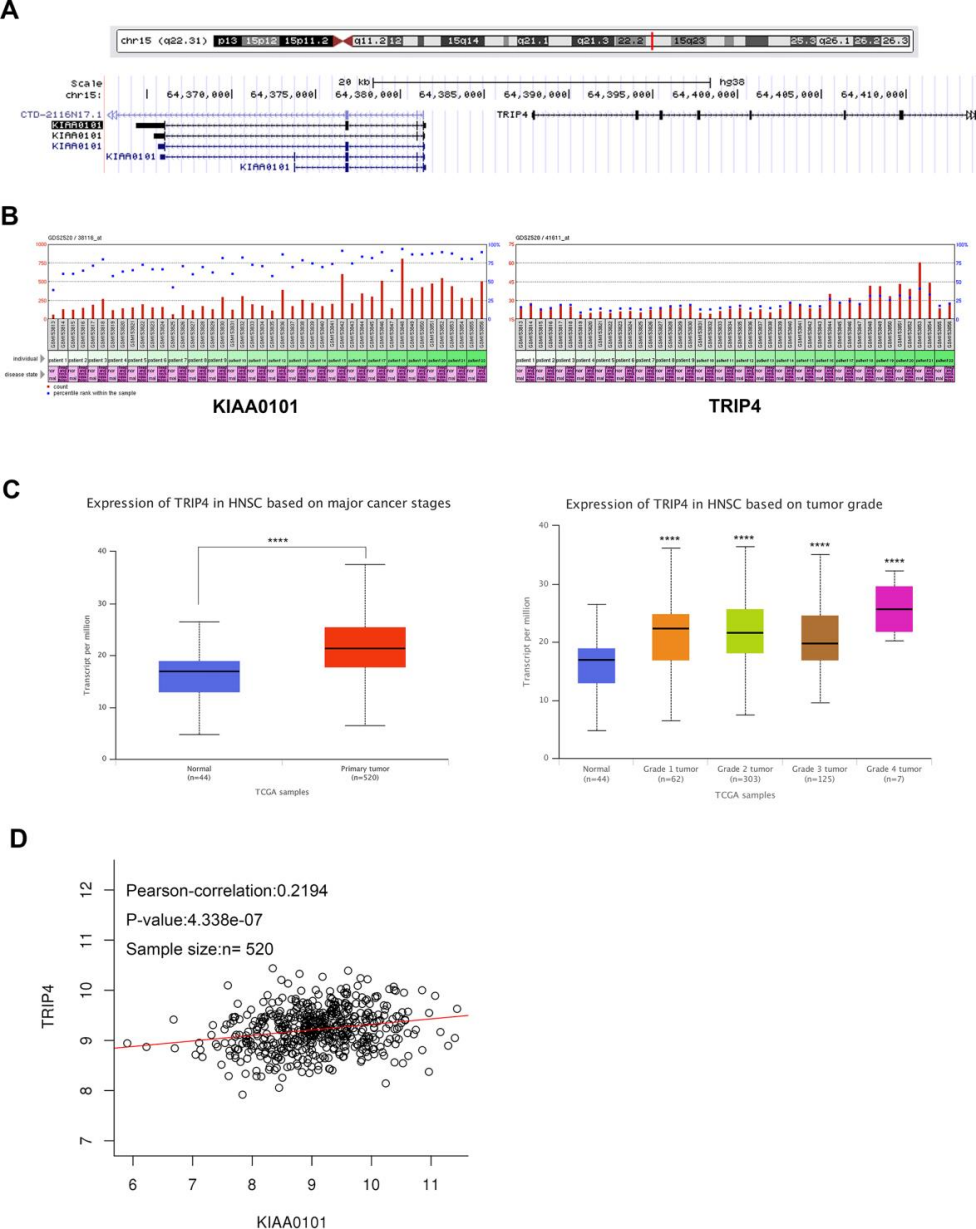
**Neighboring gene TRIP4 shows correlated co-expression pattern with KIAA0101 in HNSC.** (**A**) Chromosomal locations of human KIAA0101 and its neighbour gene TRIP4. (**B**) KIAA0101 has similar expression profiles with TRIP4 in GDS2520 HNSC samples. (**C**) TRIP4 expression and its association with tumour grade in HNSC were analysed by UALCAN. (**D**) Correlated expressions between TRIP4 and KIAA0101 in TCGA HNSC samples, ****p<0.0001.

The chromosomal localization of a gene determines its expressions and regulation modes to some extent. As Figure 6A showed that TRIP4 was the nearest neighboring gene in a divergent orientation (←, →) with KIAA0101 with the help of UCSC Genome Browser. Further expression analysis revealed that similar expression profiles of KIAA0101 and TRIP were found in in GDS2520 NPC samples (Figure 6B). TRIP4 mRNA expression was also upregulated significantly in TCGA HNSC-normal samples (Figure 6C left, ****P<0.0001), and this expression was positively correlated with patient’s tumor grade (Figure 6C right, ****P<0.0001). LinkedOmics correlation analysis further proved our suspicion that TRIP4 showed correlated co-expression patterns with KIAA0101 in NPC (Figure 6D, ****P<0.0001). All data suggested that KIAA0101 and its neighboring gene TRIP4 may be co-regulated by certain transcriptional factor.

### The expression of KIAA0101 is transcriptionally induced by ELK1 in vitro

**Figure 7:**
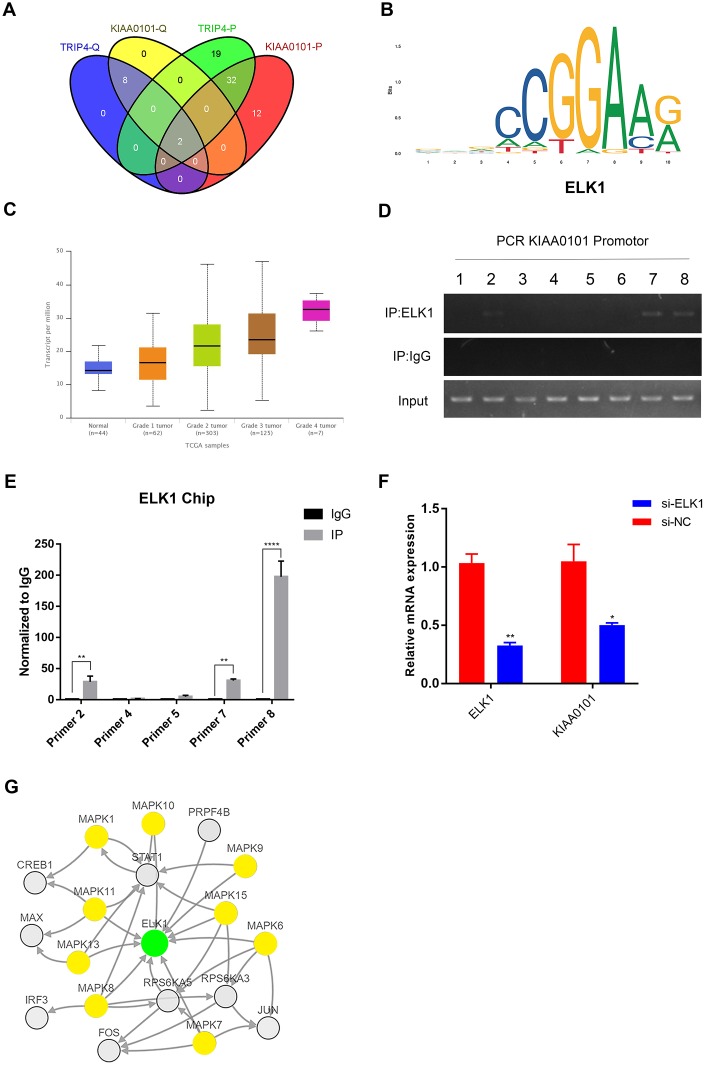
**ELK1 transcriptionally induces KIAA0101 expression.** (**A**) Venn diagram of transcription factors prediction of KIAA0101 and TRIP4 by PROMO and QIAGEN. (**B**) Representative sequence logo of ELK-1 binding specificity queried from the Jaspar^2018^, (identifier MA0028.1). (**C**) ELK1 expression and its association with tumour grade in HNSC were analysed by UALCAN. (**D**, **E**) ChIP assay was performed in CNE-2Z cells using anti-ELK1, and normal IgG. Input of sheared chromatin was prepared prior to immunoprecipitation. **p<0.01, ****p<0.0001. (**F**) Relative expression of KIAA0101 in CNE-2Z cells after siELK1 treatment. Histogram is the average ratio (mean ± SD) of three independent experiments. **p<0.001, *p<0.005 by Student’s t test. (**G**) MAPK-ELK1 signalling pathway revealed by cBioPortal. Network view of the ELK1 neighbourhood in HNSC. The network contains 19 nodes, including 1 query gene (green node) and the 18 most frequently altered neighbour genes. The depth of colour represents the degree of alteration.

In order to identify the mechanisms controlling the proliferation rate and apoptosis of NPC cell lines at the transcriptional level, PROMO and QIAGEN were chosen to predict the transcription factor binding sites for KIAA0101 and TRIP4 (Supplementary Table 7). As Figure 7A showed that two transcriptional factors ELK1 and IRF2 were obtained after taking the intersections. Then we searched for possible binding sites for two transcriptional factors in KIAA0101 and TRIP4 promoter regions with the help of Jaspar database, and found that 8 sets of high comparability DNA alignment with sequence logo for ELK1 in the promoter region of KIAA0101 and 15 binding sites in TRIP4, while with only 1 binding sites for IRF2 in the promoter region KIAA0101 (Supplementary Tables 8, 9). 6 position specific weight matrixes with p-values lower than 1e^-10^ were found in sequence logo for ELK1 in the promoter region of KIAA0101 (Figure 7B). Further expression analysis also elucidated that ELK1 expression was overexpressed significantly and positively correlated with HNSC patient’s tumor grades (Figure 7C). We then conducted the ChIP assay to detect the binding capacity of ELK1 on KIAA0101 promoter region. The PCR-gel electrophoresis and real-time quantitative PCR results showed that ELK1 directly bound to position 2, 7, 8 binding sites of the KIAA0101 promoter among the eight predicted sites (Figure 7D–7E). Using RNA interference technique to knock down the ELK1 expression in CNE-2Z cells, we found that KIAA0101 expression was also downregulated significantly in contrast with negative control (Figure 7F). The gene network, which could further reveal the regulation mechanism of signal transduction that interacted with ELK1 in HNSC. As Figure 7G showed that among 18 most frequently altered neighbor genes, there were 9 genes (yellow node) belonging to MAPK signaling pathway. These findings confirmed that the expression and function of KIAA0101 in NPC were activated by MAPK-ELK1 signaling.

## DISCUSSION

NPC is the main cancer of otorhinolaryngology malignant tumors that occurs in the epithelium of the nasopharyngeal mucosa [25]. It is also called “Canton tumor”. According to statistics from the World Health Organization, 80% of patients with NPC occur in China, and the incidence of NPC is higher in southern China than that in northern China [26]. To date, the main treatments for NPC are radiotherapy combined with chemotherapy or targeted therapy [27]. However, these treatments may lead to strong side effects and certain side effects could not reverse over time. Therefore, there is an urgent need to clarify the pathogenesis of NPC progression and discover better therapeutic targets of NPC treatment.

In our previous study, we found that KIAA0101 was significantly overexpressed and promoted the proliferation rates of the prostate cancer cells [15]. These results correlated well with previous observations in breast cancer [10], rectal cancer [16], renal cell carcinoma [11], which further supported the emerging role of KIAA0101 as a potential therapeutic target for cancer treatment. However, few studies have paid attention to explore the function of KIAA0101 gene in NPC. Our study aimed to investigate comprehensively the multifunction of KIAA0101 in proliferation, apoptosis, cell cycle arrest, and DNA replication ability in vivo, as well as in transcriptional regulations.

By employing a combination of bioinformatics approaches, we firstly investigated the expression patterns of dysregulated genes in NPC, and found that KIAA0101 was top ranked and overexpressed in GDS3610 NPC dataset (Figure 1). We also validated this finding in Sengupta GEO dataset and cohort of 40 paired HNSC and adjacent normal tissues. Further expression analysis revealed that this high expression of KIAA0101 was positively correlated with patient’s tumor grade, suggesting a possible essential role of KIAA0101 overexpression in NPC development (Figure 2).

Next, we investigated the phenotypical changes of the NPC cells in the presence and absence of KIAA0101. It is known that KIAA0101 knockdown suppressed cell proliferation, cell cycle progression and DNA synthesis [10, 28], suggesting a role for KIAA0101 in modulating the cell division. Consistently, we demonstrated that KIAA0101 silencing inhibited the proliferation rate, colony formation ability, and promoted the apoptosis of NPC cells, due to the cell cycle arrest and DNA replication blocking (Figures 4, 5).

**Figure 8:**
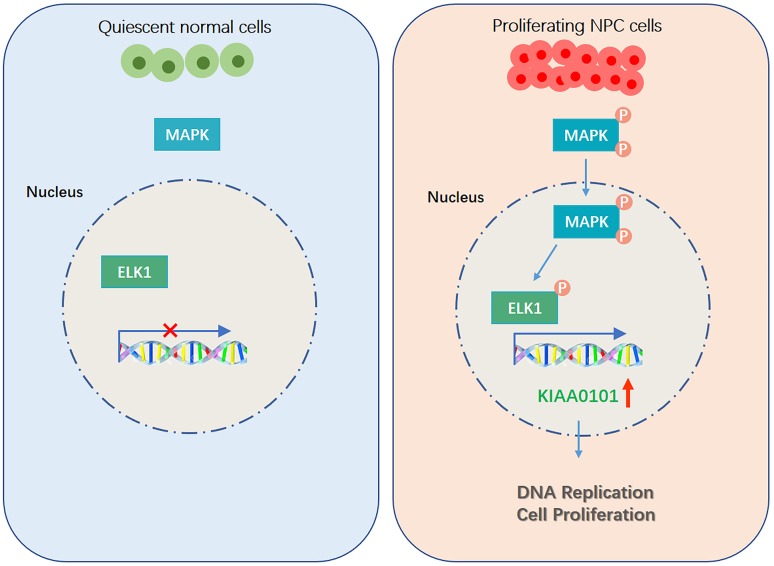
**Graphical abstract: Schematic figure illustrating the role of KIAA0101 in NPC.**

Although the expression of KIAA0101 has been found to be overexpressed in human NPC, the factors involved in this overexpression pattern in NPC patients are still not elucidated. Interestingly, more and more studies have revealed that eukaryotic genes with similar expression levels are not randomly distributed but tend to cluster in the genome [29]. In this study, using the GEO and TCGA datasets, we found that neighboring gene TRIP4 showed the similar expression profiles with KIAA0101 in NPC samples. For KIAA0101 and TRIP4 were in a divergent orientation (←, →) in the genome, both transcriptions may be induced by some transcriptional factors [30] (Figure 6). By using the PROMO, QIAGEN and JASPR prediction databases, we found that ELK1 was the most likely to bind to the promoter region of KIAA0101. Then, by applying ChIP and qRT-PCR assays, we determined that ELK1 could bind to the promoter regions of KIAA0101 and enhance its transcriptional activity. Finally, ELK1 upstream signalling pathways were unravelled by cBioPortal (Figure 7). Consistent with previous reports [31, 32], our findings combined with previous studies suggest that the abnormal activation of ELK1 by MAPK may play an important role in KIAA0101 overexpression in the human NPC progression (Figure 8). Therefore, MAPK-ELK1 mediated KIAA0101 overexpression could account for its controlling of cell proliferation and apoptosis in NPC. Taking the findings together, our study shows that KIAA0101 is overexpressed in the NPC sample, and increased KIAA0101 is associated with grade of NPC patients. Knockdown of KIAA0101 shows tumor-suppressive effects by inhibiting cell proliferation, and promoting apoptosis. Furthermore, the transcription factor ELK1 activated KIAA0101 transcription. Our findings expand our understanding of the NPC pathogenesis and may facilitate the development of KIAA0101-directed diagnostics and therapeutics in NPC. However, whether KIAA0101 can be induced by other different mechanisms was not investigated, which is worthy for our further research.

## MATERIALS AND METHODS

### Chemicals and reagents

Fetal bovine serum (FBS) was obtained from Gemini Bio (California, USA). RPMI 1640 medium, ECL-PLUS/Kit, prestained protein marker, apoptosis detection kit, and puromycin were purchased from Thermo Fisher Scientific (MA, USA). TRIzol, trypsin, dNTPs, oligo dT, Rnase Inhibitor were obtained from Invitrogen (Carlsbad, CA, USA). Trypsin, BCA protein assay kit and RIPA lysis buffer were obtained from Beyotime biotechnology (Shanghai, China). GIEMSA staining reagent and paraformaldehyde were purchased from Dingguo Biotechnology (Shanghai, China).

### Cell culture and construction of stable cell lines

Human NPC cell lines (CNE-2Z, and 5-8F) were obtained from the Cell Center of Central South University (Changsha, China). Both cells were cultured in RPMI 1640 medium supplemented with 10% fetal bovine serum and placed in a humidified incubator containing 5% CO_2_ at 37°C. Lentiviral vector PSCSI-GFP shRNA for KIAA0101 knockdown (shKIAA0101-1 and shKIAA0101-2, more information in Supplementary Table 1) were purchased from GENECHEM (Shanghai, China). CNE-2Z cells were employed to establish stable cell lines by puromycin (1 μg/ml) selection for 3 weeks. Cells in the exponential growth phase were used for all the experiments.

### Total RNA extraction and quantitative Real-time PCR detection

Cells were harvested and the total RNA was extracted by using TRIzol reagent, and then quantified with nanodrop 2000 spectrophotometer (Thermo Fisher Scientific, MA, USA). For RNA reverse transcription, 2 μg of total RNA was reverse-transcribed in a volume of 10 μl using oligo dT primers under standard conditions. For qRT-PCR assays, riboSCRIPT qRT-PCR Starter Kit (Ribio, Guangzhou, China) was used to determine the expression levels of KIAA0101 and ELK1 in NPC cells according to the manufacturer’s instructions. Glyceraldehyde-3-phosphate dehydrogenase (GAPDH) was used as endogenous reference gene. Bar graphs are the mean±SD of three separate experiments. The primer sequences are listed in Supplementary Table 1.

### Western blotting analysis

Cultured cells were rinsed with PBS twice and lysed in precooled 2×lysis buffer [1M Tris-HCl (pH 6.8), 2% β-mercaptoethanol, 20% glycerol, and 4% sodium dodecyl sulfate (SDS)] for 15 min. BCA protein assay kit (Pierce Biotech, Rockford, IL) was then employed to determine the protein concentrations in each sample. Protein were separated in 10% resolving gels and transferred to polyvinylidene difluoride (PVDF) membranes (Merck Millipore, Burlington, USA). After 1 hour blocking in TBST (tris-buffered saline, 0.1% tween 20) containing 5% skimmed milk, membranes were incubated with primary antibodies against KIAA0101 (ab56773) and GAPDH (sc-32233) followed by incubation with goat anti-mouse IgG-HRP secondary antibody (sc-2005). The dilution of primary antibodies was as follows: KIAA0101 (1: 500, Abcam, Cambridge, UK); GAPDH (1: 2000, Santa Cruz Biotechnology, Texas, USA); secondary antibody (1:2000, Santa Cruz Biotechnology, Texas, USA). Enhanced chemiluminescence detection was finally carried out with standard techniques.

### Celigo cell count and MTT assay

Each experimental group of the NPC cells in the logarithmic growth phase were digested with trypsin, suspended in the complete medium, counted with blood corpuscle counting meter, and finally planted in 96-well plates at the density of 1500 cells/well (five replicates per group). For cell count, the plates were read by Celigo Imaging Cytometer (Nexcelom Bioscience, Lawrence, Massachusetts, USA) once a day for five consecutive days. By adjusting the input parameters of analysis settings, the number of cells with green fluorescence in each scanned plate was calculated accurately, and the cell proliferation curves were statistically plotted. For MTT assay, 20 μl MTT (5 mg/mL) was added into each well 4 hours before culture termination with 100 μl dimethyl sulfoxide (DMSO), and the OD value at 490 nm was measured by Tecan infinite M2009PR Microplate Reader (BioSurplus, Inc. San Diego, California, United States). Bar graphs are the mean±SD of three separate experiments. **p<0.01, ***p<0.0001, ****p<0.00001.

### DNA synthesis proliferation assay by BrdU detection

DNA synthesis proliferation rate was analyzed by Cell Proliferation ELISA, BrdU kit (Roche, Penzberg, Upper Bavaria, Germany) according to the manufacturer’s instructions. Briefly, cells were planted in 96-well plates at at 1,500 cells/well and allowed to attach for 24 h. The cells were treated with BrdU labeling reagent reagent for 6 h to allow incorporation into newly synthesized DNA and then fixed with FixDenat ready-to-use reagent and recognized with anti-BrdU-peroxidase antibody. After washing and substrate reactions, the OD value at 450 nm was measured by Tecan infinite M2009PR Microplate Reader (BioSurplus, Inc. San Diego, California, United States). Bar graphs are the mean±SD of three separate experiments. ****p<0.00001.

### Colony formation assay

Lentivirus mediated stable NPC cell lines in the logarithmic growth phase were also digested and suspended with complete medium. After cell counting, cells were planted in 6-well plates at the density of 1000 cells/well (triple replicates per group). The inoculated cells were cultured in the incubator for about 10 days, during which the culture medium was changed every 3 days. 1 mL 4% paraformaldehyde was then added into each well to fixe cells for 45 minutes. Colonies were finally stained with GIEMSA dyeing solution for 10 minutes and counted. Bar graphs are the mean±SD of three separate experiments. **p<0.01, ***p<0.0001, ****p<0.00001.

### Apoptosis and cell cycle detection by flow cytometry

Previous stable NPC cell lines were planted in 6-well plates and cultured until the coverage rate to about 80%. For apoptosis assay, both the adherent cells and the cells in supernatant were collected and detected by the apoptosis detection kit. For cell cycle detection, the adherent cells were collected and stained with PI dyeing solution and counted by Guava® easyCyte 5HT Benchtop Flow Cytometer (Merck Millipore, Burlington, Massachusetts, USA). In order to ensure that the number of cells for testing was enough, the number of cells was more than or equal to 5 *10^5^/group). Bar graphs are the mean±SD of three separate experiments. **p<0.01, ***p<0.0001, ****p<0.00001.

### Sequence analysis and ChIP assay

The neighbor genes and promoter region of homo sapiens KIAA0101 were analyzed by The NCBI gene browser (https://www.ncbi.nlm.nih.gov/gene/) [33]. Then the potential transcription factors which bound with the KIAA0101 promoter region were predicted by means of QIAGEN (http://www.qiagen.com) and PROMO 3.0 (http://alggen.lsi.upc.es/cgi-bin/promo_v3/promo/promoinit.cgi?dirDB=TF_8.3) [34] and the candidate transcription factor binding sites were predicted by means of the JASPAR database (http://jaspar.genereg.net/).

Chromatin immunoprecipitation (ChIP) was performed using ab500 ChIP kit (Abcam, Cambrige, UK) following the manufacturer's protocol. CNE-2Z cells (3×10^7^) were crosslinked with 37% formaldehyde for 10 minutes. The chromatin was then cleaved into fragments between 200 and 600 bp by ultrasound waves. Anti-ELK1 ChIP grade antibody was purchased from Abcam (ab32106, Cambrige, UK) and qRT-PCR was performed with PrimeScript™ RT-PCR Kit (TaKaRa, Kyoto, Japan). Primer details are presented in Supplementary Table 1. The expression levels of specific DNA were determined by the gray scale method. Bar graphs are the mean±SD of three separate experiments. **p<0.01, ****p<0.00001.

### Public datasets analysis

ScanGeo (http://scangeo.dartmouth.edu/ScanGEO/), GeoDiver (http://www.geodiver.co.uk) and Oncomine (www.oncomine.org) analysis tools were employed to screen the differentially expressed genes and determine the mRNA expression profiles of KIAA0101 and TRIP4 in NPC microarray datasets [35–37]. UALCAN (http://ualcan.path.uab.edu) and GEPIA (http://gepia.cancer-pku.cn) were then used to carry out the KIAA0101 and ELK1 mRNA expressions and survival analysis in TCGA HNSC datasets [38, 39]. TIMER database (https://cistrome.shinyapps.io/timer/) was also employed to analyze the differential expressions between tumor and adjacent normal tissues for KIAA0101 across all TCGA tumors [40]. Correlated gene lists and KEGG biological process enrichment of TCGA HNSC samples were analyzed by LinkedOmics (http://www.linkedomics.org/) and Metascape (http://metascape.org/gp/) [41, 42]. MEM (Multi Experiment Matrix, http://biit.cs.ut.ee/mem/) was also employed to assess co-expression gene lists across hundreds of microarray datasets or only tumor datasets [43]. cBioPortal (http://www.cbioportal.org/) was finally employed to perform interactive analysis and construct networks of signaling pathways that were altered in TCGA HNSC samples [44].

### Statistical analysis

Statistical analyses were performed by using the Statistical Product and Service Solutions (SPSS) 17.0 and GraphPad Prism 7.0. Student’s t-test was used for statistical comparison and differences among the groups due to lentivirus mediated KIAA0101 knockdown were considered significant at p value < 0.05.

### Ethics approval and consent to participate

The study protocol was approved by the Ethics Committee of Medical school of Nanjing University. Data collections and processions were performed according to policies of GEO and TCGA project.

## Supplementary Material

Supplementary Figures

Supplementary Tables 1, 2, 6, 8-10

Supplementary Table 3

Supplementary Table 4

Supplementary Table 5

Supplementary Table 7
