# Supplementary material for: PCNA-associated factor KIAA0101 transcriptionally induced by ELK1 controls cell proliferation and apoptosis in nasopharyngeal carcinoma: an integrated bioinformatics and experimental study
Source: Aging (Albany NY). 2020 Apr 9;12(7):5992–6017. doi: 10.18632/aging.102991 (PMC7185143; doi:10.18632/aging.102991)
Supplement: Supplementary Figures [file aging-12-102991-s005..pdf]

SUPPLEMENTARY FIGURES

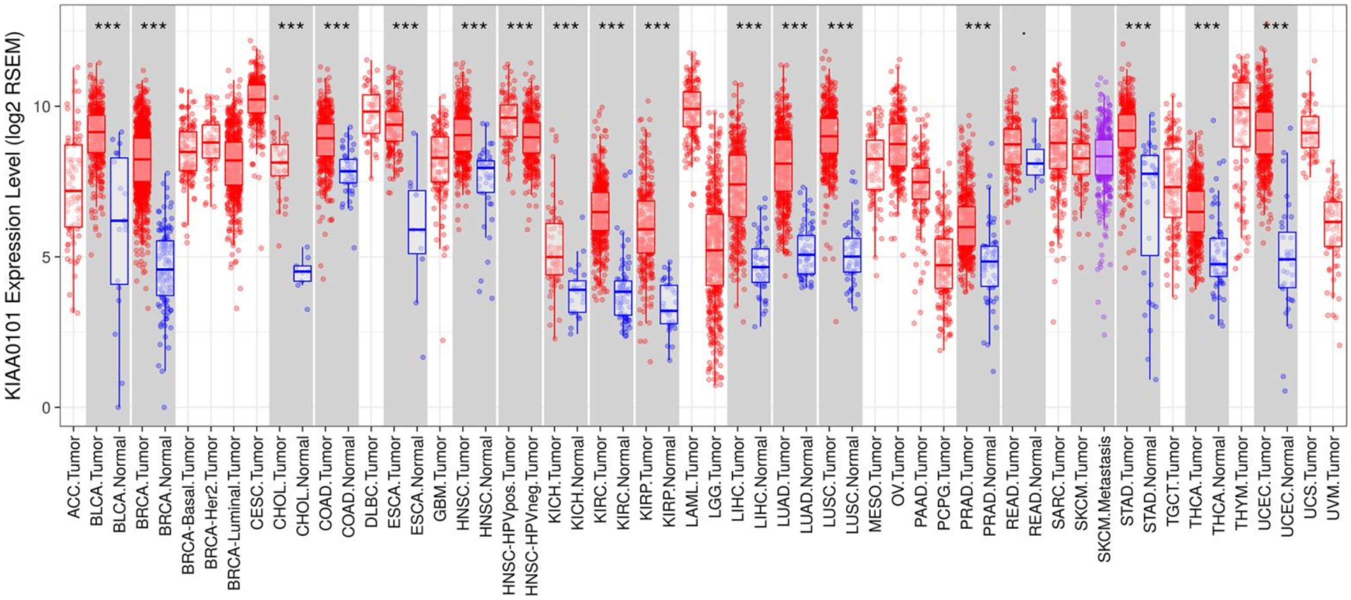

Supplementary Figure 1. Highly expressed KIAA0101 in several human cancer types.

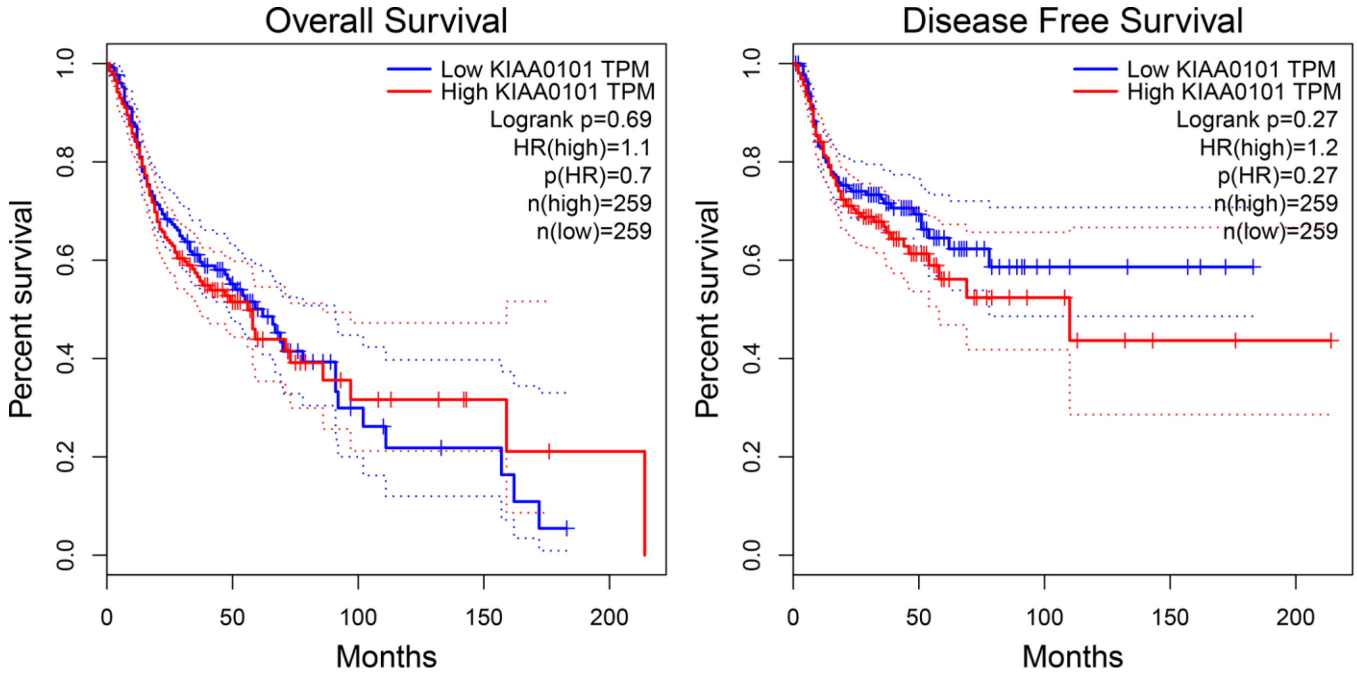

Supplementary Figure 2. Overall and disease-free survival analysis of KIAA0101 in TCGA HNSC samples.

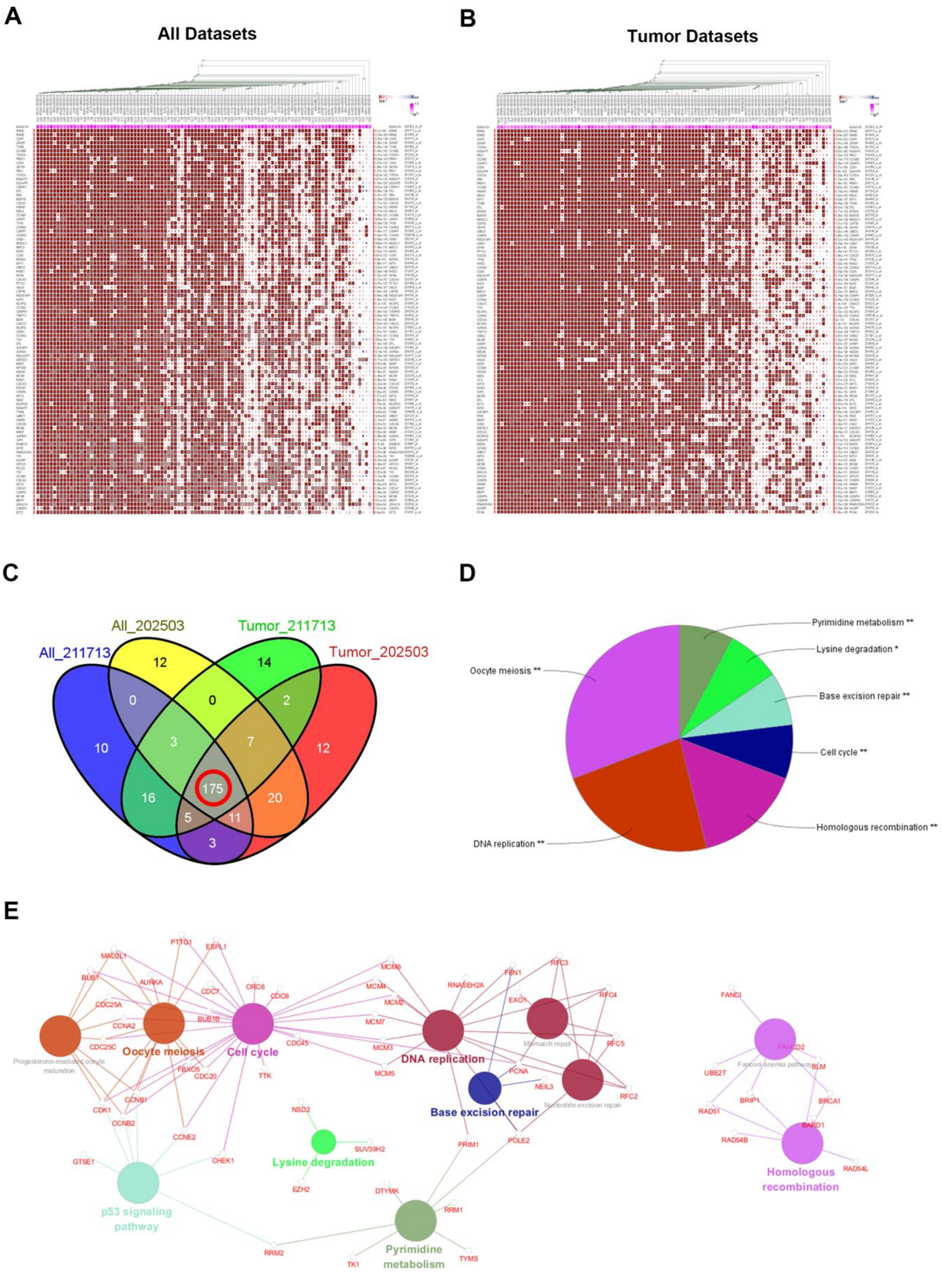

Supplementary Figure 3. MEM co-expression analysis of KIAA0101 reveals DNA replication and cell cycle pathways participate in tumor process. (**A**, **B**) Heatmap analysis of all and tumor datasets showing top 100 co-expressed genes with KIAA0101 probe 202503_S_AT. (**C**) Overlap venn diagram of co-expressed genes in all and tumor datasets queried with KIAA0101 probes 202503_S_AT and 211713_S_AT. (**D**, **E**) Pie chart and pathway enrichment analysis of 158 common genes with Cytoscape CluoGO KEGG (17 same genes deleted).

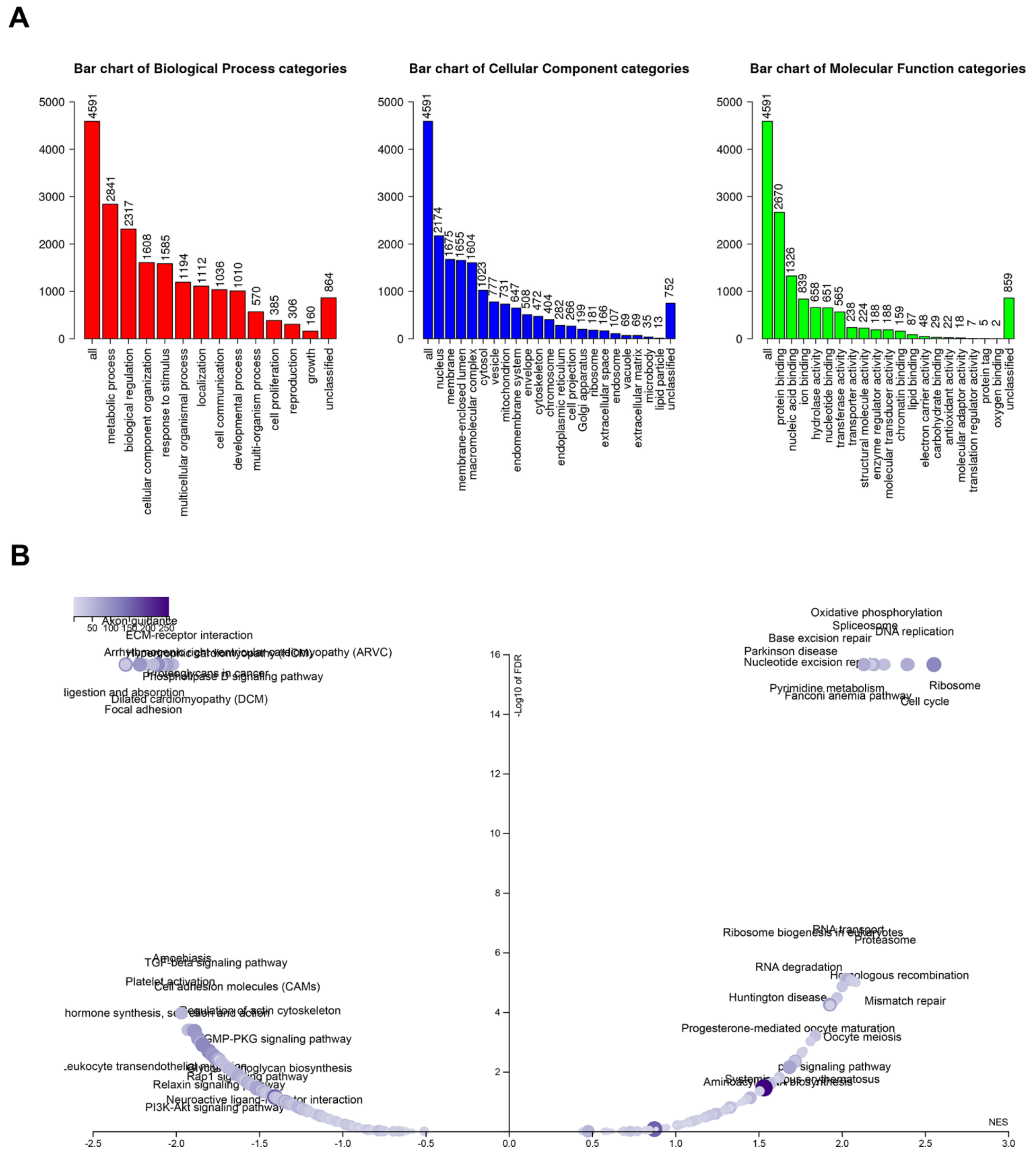

Supplementary Figure 4. LinkedOmics GSEA analysis results of co-expression genes with positive or negative correlations with KIAA0101 in TCGA HNSC samples.
