# Supplementary material for: PCNA-associated factor KIAA0101 transcriptionally induced by ELK1 controls cell proliferation and apoptosis in nasopharyngeal carcinoma: an integrated bioinformatics and experimental study
Source: Aging (Albany NY). 2020 Apr 9;12(7):5992–6017. doi: 10.18632/aging.102991 (PMC7185143; doi:10.18632/aging.102991)
Supplement: Supplementary Tables 1, 2, 6, 8-10 [file aging-12-102991-s004..pdf]

SUPPLEMENTARY TABLES

Supplementary Table 1. All sequences employed in this study.

Primer ID	Forward Primer	Reverse Primer	Comment
KIAA0101	CTCTGCCACTAATTCGACATCA	TTCAGAATCTTTAGGGGACAAC	qRT-PCR
ELK1			
GAPDH	TGACTTCAACAGCGACACCCA	CACCCTGTTGCTGTAGCCAAA	qRT-PCR
KIAA0101-1	GGAAATAATGGCATTTAAGAA	TTCTTCGCCCAGGTTGGAAT	ChIP-qPCR for scanning region
KIAA0101-2	ATTCCAACCTGGGCGAAGAA	TCCAATGAATTGCTCGAACT	ChIP-qPCR for scanning region
KIAA0101-3	AGTTCGAGCAATTCATTGGA	AACCCAGTCTCTACTAAAATAA	ChIP-qPCR for scanning region
KIAA0101-4	TTATTTTAGTAGAGACTGGGTT	CAAGGAAATGAAATGAAACGA	ChIP-qPCR for scanning region
KIAA0101-5	TCGTTTCATTTCATTTCCTTG	AGGTCGAGACCAGCCTGACC	ChIP-qPCR for scanning region
KIAA0101-6	GGTCAGGCTGGTCTCGACCT	GTACAGCATCCATACTAAAAC	ChIP-qPCR for scanning region
KIAA0101-7	GTTTTAGTATGGATGCTGTAC	AATCCGTCCATCAACACGCAA	ChIP-qPCR for scanning region
KIAA0101-8	TTGCGTGTTGATGGACGGATT	GCCTCACCTTTTCTGTAAGTG	ChIP-qPCR for scanning region
shKIAA0101-1	AACCTGATCACACAAATGA		
shKIAA0101-2	TGTAAACTCGAGTTTACATT		

Supplementary Table 2. KIAA0101 expression data in Sengupta Head-Neck.

Nasopharynx	Nasopharyngeal Carcinoma
1.2183	3.27893
1.61622	3.34779
2.68014	3.42557
2.94873	4.05322
3.16481	4.10715
3.20637	4.121
3.37418	4.25036
3.63642	4.37505
3.85271	4.38508
4.01089	4.39445
	4.40828
	4.48598
	4.627
	4.65279
	4.66512
	4.81555
	4.90956
	5.05575
	5.11346
	5.12048
	5.13239
	5.17643
	5.24821
	5.46213
	5.4686
	5.48934
	5.55987
	5.5913
	5.60084
	5.78752
	5.99109

Supplementary Table 6. Common gene lists of KIAA0101 co-expressed genes in all and tumor datasets.

KIAA0101	CENPW	FANCD2	RFC5	CHEK1
RRM2	TRIP13	STIL	DEPDC1B	RAD51
CDK1	BUB1	KIF18A	KNSTRN	SGOL2
ZWINT	CASC5	RFC4	TACC3	DDIAS
TYMS	GINS2	CENPN	CCNE2	BRCA1
CCNB2	TTK	CDC45	GMNN	TCF19
TOP2A	SHCBP1	PLK4	EXO1	CDC7
PRR11	AURKA	RFC3	SKA1	KIAA1524
ASPM	RACGAP1	AURKB	KNTC1	ERCC6L
PRC1	DEPDC1	SPAG5	WDHD1	PSMC3IP
NUSAP1	MKI67	NCAPH	MCM5	
DLGAP5	KIF18B	CENPM	LMNB1	
CENPU	NDC80	NCAPD2	ATAD2	
DTL	MCM4	DTYMK	MIS18A	
PBK	CDCA5	MCM10	SPC24	
BUB1B	FOXM1	MND1	VRK1	
CDC20	CENPA	MCM3	DHFR	
HMMR	KIF15	GTSE1	ZNF367	
MELK	NEK2	RRM1	LMNB2	
CCNB1	NCAPG2	PRIM1	TMPO	
UHRF1	UBE2T	CDC6	CDT1	
TPX2	CDCA8	CENPH	KIFC1	
CDKN3	MCM2	MTFR2	DSCC1	
CENPF	OIP5	FBXO5	LRR1	
GINS1	KIF23	PARPBP	POLQ	
MAD2L1	RNASEH2A	ASF1B	BLM	
BIRC5	TK1	HELLS	SUV39H2	
KIF2C	HJURP	SMC4	CENPO	
KIF20A	SPC25	EZH2	MCM7	
KIF11	POLE2	TIMELESS	MYBL2	
UBE2C	CDCA2	ESPL1	MCM8	
FANCI	KIF14	H2AFZ	NEIL3	
KIF4A	CDCA7	SKA3	CDC25A	
CDCA3	CENPE	RAD54L	CSE1L	
PTTG1	MCM6	E2F8	CENPI	
ANLN	BRIP1	CKAP2L	CHAF1A	
CEP55	ZWILCH	WHSC1	RFC2	
RAD51AP1	CENPK	HMGB2	RAD54B	
NUF2	ECT2	ORC6	BARD1	
NCAPG	FEN1	STMN1	TIPIN	
CCNA2	PCNA	CDC25C	C4ORF46	

Supplementary Table 8. Transcriptional factors prediction of KIAA0101 promotor by QIAGEN and PROMO.

QIAGEN		PROMO	
Elk-1	Elk-1	Ik-1	GR
CREB	CREB	AP-2αA	GR-α
deltaCREB	deltaCREB	GR-β	HNF-3α
IRF-2	IRF-2	C/EBPβ	GR-β
E2F-4	E2F-4	C/EBPα	STAT4
E2F-5	E2F-5	RXR-α	c-Ets-1
E2F	E2F	RAR-β	Elk-1
E2F-1	E2F-1	PXR-1:RXR-α	AP-2αA
E2F-2	E2F-2	TFII-I	Pax-5
E2F-3a	E2F-3a	STAT4	p53
		IRF-2	C/EBPβ
		YY1	FOXP3
		TFIID	TFII-I
		GR	YY1
		FOXP3	NF-AT2
		SRY	TFIID
		GR-α	GATA-1
		FOXO4	C/EBPα
		c-Ets-2	NF-Y
		Pax-5	RXR-α
		p53	XBP-1
		c-Ets-1	LEF-1
		HNF-1A	TCF-4
		NF-Y	SRY
		XBP-1	PR B
		ER-α	PR A
		EBF	POU2F2
		GATA-1	ER-α
		PEA3	IRF-2
		Elk-1	HNF-1A
		IRF-1	c-Myc
		POU2F1	Ik-1
		T3R-β1	PXR-1:RXR-α
		AP-1	USF1
		c-Jun	NFI/CTF
		LEF-1	c-Ets-2
		Sp1	AhR
		TCF-4E	Sp1
		HNF-3α	ELF-1
		PR B	TBP
		PR A	IRF-1
		HOXD9	HIF-1

HOXD10	NF-AT1
PITX2	NF-AT1
VDR	PITX2
c-Myb	GCF
	WT1 I -KTS
	WT1 -KTS
	WT1 I
	WT1-del2
	WT1 I-del2
	NF-1
	TCF-4E
	CTF

Supplementary Table 9. JASPAR analysis of ELK1 binding sites in promotor regions of KIAA0101 and TRIP4.

Matrix ID	Name	Score	Relative score	Sequence ID	Start	End	Strand	Predicted sequence
MA0028.1	ELK1	6.52728	0.829157	KIAA0101	47	56	+	AACCCGGGAG
MA0028.1	ELK1	10.9935	0.977574	KIAA0101	220	229	-	CAACCGGAAA
MA0028.1	ELK1	5.80972	0.805311	KIAA0101	520	529	+	GAGACGGAGA
MA0028.1	ELK1	6.52728	0.829157	KIAA0101	590	599	-	AACCCGGGAG
MA0028.1	ELK1	6.14512	0.816457	KIAA0101	982	991	-	GAAACGAAAA
MA0028.1	ELK1	6.37588	0.824125	KIAA0101	1183	1192	-	GAGACGGGAG
MA0028.1	ELK1	6.92053	0.842225	KIAA0101	1567	1576	-	GAAAAGGAAG
MA0028.1	ELK1	5.75032	0.803337	KIAA0101	1635	1644	+	CTAAAGGAAA

Matrix ID	Name	Score	Relative score	Sequence ID	Start	End	Strand	Predicted sequence
MA0028.1	ELK1	6.60984	0.8319	seq1	29	38	-	CAGAAGGAAG
MA0028.1	ELK1	5.94028	0.80965	seq1	33	42	-	GCTCCAGAAG
MA0028.1	ELK1	7.63633	0.866012	seq1	185	194	-	GAGACTGAAA
MA0028.1	ELK1	7.17167	0.85057	seq1	200	209	-	GCTCAGGAAA
MA0028.1	ELK1	5.96268	0.810394	seq1	441	450	+	ACCCTGGAAA
MA0028.1	ELK1	6.50414	0.828388	seq1	606	615	-	GGCCTGGAAG
MA0028.1	ELK1	6.66503	0.833734	seq1	734	743	+	GGTCCGGAAC
MA0028.1	ELK1	6.86817	0.840485	seq1	1078	1087	+	AAGCCAGAAA
MA0028.1	ELK1	5.85542	0.80683	seq1	1089	1098	+	GAATGGGAAG
MA0028.1	ELK1	5.96268	0.810394	seq1	1287	1296	+	CTCCTGGAAA
MA0028.1	ELK1	6.52728	0.829157	seq1	1499	1508	+	AACCCGGGAG
MA0028.1	ELK1	9.39302	0.924389	seq1	1718	1727	-	TACCCGGAAA
MA0028.1	ELK1	5.67811	0.800938	seq1	1856	1865	+	ACTCCGGAGG
MA0028.1	ELK1	8.29429	0.887877	seq1	1886	1895	+	AAGCAGGAAG
MA0028.2	ELK1	8.7674	0.879778	seq1	736	745	+	TCCGGAACTC

Supplementary Table 10. All sample information of GDS2520 and GDS3610 datasets.

Sample	Title
GSM153813	Normal mucosa 1
GSM153814	Cancer 1
GSM153815	Normal mucosa 2
GSM153816	Cancer 2
GSM153817	Normal mucosa 3
GSM153818	Cancer 3
GSM153819	Normal mucosa 4
GSM153820	Cancer 4
GSM153821	Normal mucosa 5
GSM153822	Cancer 5
GSM153823	Normal mucosa 6
GSM153824	Cancer 6
GSM153825	Normal mucosa 7
GSM153826	Cancer 7
GSM153827	Normal mucosa 8
GSM153828	Cancer 8
GSM153829	Normal mucosa 9
GSM153830	Cancer 9
GSM153831	Normal mucosa 10
GSM153832	Cancer 10
GSM153833	Normal mucosa 11
GSM153834	Cancer 11
GSM153835	Normal mucosa 12
GSM153836	Cancer 12
GSM153837	Normal mucosa 13
GSM153838	Cancer 13
GSM153839	Normal mucosa 14
GSM153840	Cancer 14
GSM153841	Normal mucosa 15
GSM153842	Cancer 15
GSM153843	Normal mucosa 16
GSM153844	Cancer 16
GSM153845	Normal mucosa 17
GSM153846	Cancer 17
GSM153847	Normal mucosa 18
GSM153848	Cancer 18
GSM153849	Normal mucosa 19
GSM153850	Cancer 19
GSM153851	Normal mucosa 20
GSM153852	Cancer 20
GSM153853	Normal mucosa 21
GSM153854	Cancer 21
GSM153855	Normal mucosa 22
GSM153856	Cancer 22

Samples	Factors	Title
	disease state	
GSM342152	control	Normal control 1
GSM342153	control	Normal control 2
GSM342154	control	Normal control 3
GSM342155	nasopharyngeal carcinoma	NPC1
GSM342156	nasopharyngeal carcinoma	NPC2
GSM342157	nasopharyngeal carcinoma	NPC3
GSM342158	nasopharyngeal carcinoma	NPC4
GSM342159	nasopharyngeal carcinoma	NPC5
GSM342160	nasopharyngeal carcinoma	NPC6
GSM342161	nasopharyngeal carcinoma	NPC7
GSM342162	nasopharyngeal carcinoma	NPC8
GSM342163	nasopharyngeal carcinoma	NPC9
GSM342164	nasopharyngeal carcinoma	NPC10
GSM342165	nasopharyngeal carcinoma	NPC11
GSM342166	nasopharyngeal carcinoma	NPC12
GSM342167	nasopharyngeal carcinoma	NPC13
GSM342168	nasopharyngeal carcinoma	NPC14
GSM342169	nasopharyngeal carcinoma	NPC15
GSM342170	nasopharyngeal carcinoma	NPC16
GSM342171	nasopharyngeal carcinoma	NPC17
GSM342172	nasopharyngeal carcinoma	NPC18
GSM342173	nasopharyngeal carcinoma	NPC19
GSM342174	nasopharyngeal carcinoma	NPC20
GSM342175	nasopharyngeal carcinoma	NPC21
GSM342176	nasopharyngeal carcinoma	NPC22
GSM342177	nasopharyngeal carcinoma	NPC23
GSM342178	nasopharyngeal carcinoma	NPC24
GSM342179	nasopharyngeal carcinoma	NPC25
