# Supplementary material for: PCNA-associated factor KIAA0101 transcriptionally induced by ELK1 controls cell proliferation and apoptosis in nasopharyngeal carcinoma: an integrated bioinformatics and experimental study
Source: Aging (Albany NY). 2020 Apr 9;12(7):5992–6017. doi: 10.18632/aging.102991 (PMC7185143; doi:10.18632/aging.102991)
Supplement: Supplementary Table 3 [file aging-12-102991-s003..docx]

**Supplementary Table 3**. **Specific patient information and KIAA0101 expression data in TCGA HNSC.**

| **No.** | **bcr_patient_barcode** | **clinical_stage** | **form_completion_date** | **anatomic_neoplasm_subdivision** | **gender** |
| --- | --- | --- | --- | --- | --- |
| 1 | TCGA-CV-7091 | Stage I | 40827 | Oral Cavity | MALE |
| 2 | TCGA-CV-7177 | Stage I | 40827 | Larynx | FEMALE |
| 3 | TCGA-CV-6938 | Stage II | 40799 | Oral Cavity | MALE |
| 4 | TCGA-CV-6961 | Stage II | 40798 | Oral Tongue | MALE |
| 5 | TCGA-CV-6955 | Stage II | 40827 | Oral Cavity | FEMALE |
| 6 | TCGA-CV-7101 | Stage II | 40822 | Larynx | MALE |
| 7 | TCGA-CV-7103 | Stage II | 40827 | Oral Tongue | MALE |
| 8 | TCGA-CV-7183 | Stage II | 40827 | Oral Cavity | MALE |
| 9 | TCGA-CV-7238 | Stage II | 40882 | Oral Tongue | FEMALE |
| 10 | TCGA-CV-7255 | Stage II | 40882 | Oral Tongue | FEMALE |
| 11 | TCGA-CV-7406 | Stage II | 41008 | Base of tongue | MALE |
| 12 | TCGA-CV-7423 | Stage II | 40952 | Oral Cavity | MALE |
| 13 | TCGA-CV-7437 | Stage II | 41008 | Larynx | MALE |
| 14 | TCGA-CV-7438 | Stage II | 40952 | Oral Tongue | FEMALE |
| 15 | TCGA-CV-7440 | Stage II | 41008 | Larynx | MALE |
| 16 | TCGA-CV-6935 | Stage III | 40795 | Larynx | MALE |
| 17 | TCGA-CV-6943 | Stage III | 40795 | Base of tongue | MALE |
| 18 | TCGA-CV-6959 | Stage III | 40794 | Oral Tongue | MALE |
| 19 | TCGA-CV-6962 | Stage III | 40780 | Larynx | MALE |
| 20 | TCGA-CV-6960 | Stage III | 40794 | Oral Cavity | MALE |
| 21 | TCGA-CV-7097 | Stage III | 40821 | Oral Cavity | MALE |
| 22 | TCGA-CV-7235 | Stage III | 40889 | Floor of mouth | MALE |
| 23 | TCGA-CV-7242 | Stage III | 40879 | Larynx | FEMALE |
| 24 | TCGA-CV-7245 | Stage III | 40879 | Larynx | MALE |
| 25 | TCGA-CV-7250 | Stage III | 40882 | Larynx | MALE |
| 26 | TCGA-CV-7252 | Stage III | 40879 | Oral Cavity | FEMALE |
| 27 | TCGA-CV-7261 | Stage III | 40879 | Larynx | MALE |
| 28 | TCGA-CV-7425 | Stage III | 40952 | Oral Cavity | FEMALE |
| 29 | TCGA-CV-7432 | Stage III | 40952 | Oral Cavity | MALE |
| 30 | TCGA-CV-7434 | Stage III | 40952 | Oral Cavity | MALE |
| 31 | TCGA-HD-8635 | Stage III | 41271 | Oral Tongue | FEMALE |
| 32 | TCGA-HD-A6I0 | Stage III | 41537 | Oral Cavity | MALE |
| 33 | TCGA-CV-6933 | Stage IVA | 40795 | Oral Tongue | MALE |
| 34 | TCGA-CV-6934 | Stage IVA | 40799 | Oral Tongue | FEMALE |
| 35 | TCGA-CV-6936 | Stage IVA | 40799 | Floor of mouth | MALE |
| 36 | TCGA-CV-6939 | Stage IVA | 40799 | Oral Tongue | MALE |
| 37 | TCGA-CV-6956 | Stage IVA | 40827 | Oral Tongue | MALE |
| 38 | TCGA-CV-7178 | Stage IVA | 40827 | Oral Cavity | FEMALE |
| 39 | TCGA-CV-7416 | Stage IVA | 40939 | Oral Cavity | FEMALE |
| 40 | TCGA-CV-7424 | Stage IVA | 40946 | Larynx | MALE |

| **bcr_patient_barcode** | **Fold Change of Cancer/Normal(Normalized)** | **Raw Count in Cancer** | **Raw Count in Normal** | **Normalized Count in Cancer** | **Normalized Count in Normal** |
| --- | --- | --- | --- | --- | --- |
| TCGA-CV-6933 | 45.2693 | 1609 | 18 | 511.358 | 11.2959 |
| TCGA-CV-6934 | 7.98982 | 1819 | 173 | 503.181 | 62.9778 |
| TCGA-CV-6935 | 1.11017 | 584 | 507 | 237.592 | 214.014 |
| TCGA-CV-6936 | 4.69661 | 3881 | 573 | 1186.58 | 252.646 |
| TCGA-CV-6938 | 3.54058 | 966 | 486 | 681.744 | 192.552 |
| TCGA-CV-6939 | 29.3193 | 1542 | 20 | 508.575 | 17.3461 |
| TCGA-CV-6943 | 0.75647 | 896 | 734 | 283.724 | 375.064 |
| TCGA-CV-6959 | 0.73855 | 351 | 651 | 197.635 | 267.598 |
| TCGA-CV-6961 | 2.33734 | 1457 | 482 | 660.471 | 282.574 |
| TCGA-CV-6962 | 1.96367 | 1804 | 757 | 585.742 | 298.29 |
| TCGA-CV-6955 | 1.20343 | 676 | 364 | 235.889 | 196.015 |
| TCGA-CV-6956 | 3.26044 | 2345 | 342 | 680.044 | 208.574 |
| TCGA-CV-6960 | 1.19067 | 734 | 674 | 310.359 | 260.659 |
| TCGA-CV-7091 | 46.8395 | 1644 | 17 | 627.481 | 13.3964 |
| TCGA-CV-7097 | 4.73739 | 1344 | 202 | 507.937 | 107.219 |
| TCGA-CV-7101 | 1.49952 | 1143 | 821 | 476.25 | 317.602 |
| TCGA-CV-7103 | 0.77403 | 801 | 774 | 275.069 | 355.372 |
| TCGA-CV-7177 | 1.03735 | 367 | 601 | 259.364 | 250.026 |
| TCGA-CV-7178 | 3.95634 | 2079 | 282 | 688.696 | 174.074 |
| TCGA-CV-7183 | 0.64959 | 759 | 1141 | 445.423 | 685.697 |
| TCGA-CV-7235 | 10.9297 | 3340 | 367 | 1514.22 | 138.543 |
| TCGA-CV-7238 | 3.66352 | 1113 | 255 | 451.521 | 123.248 |
| TCGA-CV-7242 | 0.55704 | 727 | 1118 | 240.566 | 431.869 |
| TCGA-CV-7245 | 7.11388 | 1926 | 276 | 760.063 | 106.842 |
| TCGA-CV-7250 | 2.0231 | 1181 | 486 | 532.342 | 263.131 |
| TCGA-CV-7252 | 2.73884 | 1895 | 572 | 776.707 | 283.59 |
| TCGA-CV-7255 | 2.06562 | 2191 | 689 | 597.736 | 289.374 |
| TCGA-CV-7261 | 1.47874 | 972 | 912 | 482.682 | 326.414 |
| TCGA-CV-7406 | 3.08876 | 1510 | 288 | 501.162 | 162.254 |
| TCGA-CV-7416 | 1.08 | 1383.99 | 885 | 468.99 | 434.249 |
| TCGA-CV-7423 | 1.66621 | 1149 | 599 | 487.691 | 292.695 |
| TCGA-CV-7424 | 3.13077 | 1345 | 453 | 620.674 | 198.25 |
| TCGA-CV-7425 | 1.14701 | 1631 | 1320 | 774.454 | 675.192 |
| TCGA-CV-7432 | 20.178 | 1430 | 63 | 550.212 | 27.2679 |
| TCGA-CV-7434 | 13.5325 | 1708 | 130 | 657.901 | 48.6163 |
| TCGA-CV-7437 | 0.87801 | 687 | 1025 | 303.579 | 345.761 |
| TCGA-CV-7438 | 1.17549 | 692 | 569 | 304.578 | 259.108 |
| TCGA-CV-7440 | 4.34014 | 2724 | 748 | 1055.41 | 243.173 |
| TCGA-HD-8635 | 1.18577 | 577 | 441 | 343.803 | 289.941 |
| TCGA-HD-A6I0 | 9.67839 | 1542.98 | 182 | 1357.06 | 140.216 |
