# Supplementary material for: PCNA-associated factor KIAA0101 transcriptionally induced by ELK1 controls cell proliferation and apoptosis in nasopharyngeal carcinoma: an integrated bioinformatics and experimental study
Source: Aging (Albany NY). 2020 Apr 9;12(7):5992–6017. doi: 10.18632/aging.102991 (PMC7185143; doi:10.18632/aging.102991)
Supplement: Supplementary Table 4 [file aging-12-102991-s002..docx]

**Supplementary Table 4**. **MEM analysis of all query datasets obtains 300 KIAA0101 co-expressed gene.**

| #Score | #Gene name | #probeset id | #Gene description |  | | |  |  |
| --- | --- | --- | --- | --- | --- | --- | --- | --- |
| 8.10E-180 | RRM2 | 209773_s_at | ribonucleotide reductase M2 [Source:HGNC Symbol;Acc:HGNC:10452] | | | | | |
| 3.24E-165 | RRM2 | 201890_at | ribonucleotide reductase M2 [Source:HGNC Symbol;Acc:HGNC:10452] | | | | | |
| 3.32E-148 | CDK1 | 203213_at | cyclin-dependent kinase 1 [Source:HGNC Symbol;Acc:HGNC:1722] | | | | | |
| 1.36E-143 | ZWINT | 204026_s_at | ZW10 interacting kinetochore protein [Source:HGNC Symbol;Acc:HGNC:13195] | | | | | |
| 3.68E-143 | TYMS | 202589_at | thymidylate synthetase [Source:HGNC Symbol;Acc:HGNC:12441] | | | | | |
| 1.94E-142 | CCNB2 | 202705_at | cyclin B2 [Source:HGNC Symbol;Acc:HGNC:1580] | | | | | |
| 3.41E-142 | TOP2A | 201292_at | topoisomerase (DNA) II alpha 170kDa [Source:HGNC Symbol;Acc:HGNC:11989] | | | | | |
| 6.65E-135 | PRR11 | 228273_at | proline rich 11 [Source:HGNC Symbol;Acc:HGNC:25619] | | | | | |
| 1.47E-134 | CDK1 | 210559_s_at | cyclin-dependent kinase 1 [Source:HGNC Symbol;Acc:HGNC:1722] | | | | | |
| 1.68E-134 | ASPM | 219918_s_at | asp (abnormal spindle) homolog, microcephaly associated (Drosophila) [Source:HGNC Symbol;Acc:HGNC:19048] | | | | | |
| 1.27E-133 | PRC1 | 218009_s_at | protein regulator of cytokinesis 1 [Source:HGNC Symbol;Acc:HGNC:9341] | | | | | |
| 4.23E-132 | TOP2A | 201291_s_at | topoisomerase (DNA) II alpha 170kDa [Source:HGNC Symbol;Acc:HGNC:11989] | | | | | |
| 4.07E-130 | NUSAP1 | 218039_at | nucleolar and spindle associated protein 1 [Source:HGNC Symbol;Acc:HGNC:18538] | | | | | |
| 1.43E-128 | DLGAP5 | 203764_at | discs, large (Drosophila) homolog-associated protein 5 [Source:HGNC Symbol;Acc:HGNC:16864] | | | | | |
| 4.92E-128 | CENPU | 218883_s_at | centromere protein U [Source:HGNC Symbol;Acc:HGNC:21348] | | | | | |
| 8.86E-128 | DTL | 218585_s_at | denticleless E3 ubiquitin protein ligase homolog (Drosophila) [Source:HGNC Symbol;Acc:HGNC:30288] | | | | | |
| 1.41E-127 | PBK | 219148_at | PDZ binding kinase [Source:HGNC Symbol;Acc:HGNC:18282] | | | | | |
| 2.29E-125 | BUB1B | 203755_at | BUB1 mitotic checkpoint serine/threonine kinase B [Source:HGNC Symbol;Acc:HGNC:1149] | | | | | |
| 1.71E-123 | CDC20 | 202870_s_at | cell division cycle 20 [Source:HGNC Symbol;Acc:HGNC:1723] | | | | | |
| 2.24E-123 | HMMR | 207165_at | hyaluronan-mediated motility receptor (RHAMM) [Source:HGNC Symbol;Acc:HGNC:5012] | | | | | |
| 6.21E-123 | MELK | 204825_at | maternal embryonic leucine zipper kinase [Source:HGNC Symbol;Acc:HGNC:16870] | | | | | |
| 1.95E-122 | CCNB1 | 214710_s_at | cyclin B1 [Source:HGNC Symbol;Acc:HGNC:1579] | | | | | |
| 5.08E-121 | UHRF1 | 225655_at | ubiquitin-like with PHD and ring finger domains 1 [Source:HGNC Symbol;Acc:HGNC:12556] | | | | | |
| 9.38E-120 | TPX2 | 210052_s_at | TPX2, microtubule-associated [Source:HGNC Symbol;Acc:HGNC:1249] | | | | | |
| 2.72E-118 | CDKN3 | 209714_s_at | cyclin-dependent kinase inhibitor 3 [Source:HGNC Symbol;Acc:HGNC:1791] | | | | | |
| 1.08E-117 | CENPF | 207828_s_at | centromere protein F, 350/400kDa [Source:HGNC Symbol;Acc:HGNC:1857] | | | | | |
| 1.64E-116 | CDKN3 | 1555758_a_at | cyclin-dependent kinase inhibitor 3 [Source:HGNC Symbol;Acc:HGNC:1791] | | | | | |
| 9.98E-116 | GINS1 | 206102_at | GINS complex subunit 1 (Psf1 homolog) [Source:HGNC Symbol;Acc:HGNC:28980] | | | | | |
| 5.88E-115 | MAD2L1 | 203362_s_at | MAD2 mitotic arrest deficient-like 1 (yeast) [Source:HGNC Symbol;Acc:HGNC:6763] | | | | | |
| 1.46E-114 | BIRC5 | 202095_s_at | baculoviral IAP repeat containing 5 [Source:HGNC Symbol;Acc:HGNC:593] | | | | | |
| 2.63E-112 | KIF2C | 209408_at | kinesin family member 2C [Source:HGNC Symbol;Acc:HGNC:6393] | | | | | |
| 4.87E-112 | CDK1 | 203214_x_at | cyclin-dependent kinase 1 [Source:HGNC Symbol;Acc:HGNC:1722] | | | | | |
| 9.80E-112 | KIF20A | 218755_at | kinesin family member 20A [Source:HGNC Symbol;Acc:HGNC:9787] | | | | | |
| 1.68E-111 | KIF11 | 204444_at | kinesin family member 11 [Source:HGNC Symbol;Acc:HGNC:6388] | | | | | |
| 5.53E-110 | UBE2C | 202954_at | ubiquitin-conjugating enzyme E2C [Source:HGNC Symbol;Acc:HGNC:15937] | | | | | |
| 2.06E-109 | FANCI | 213007_at | Fanconi anemia, complementation group I [Source:HGNC Symbol;Acc:HGNC:25568] | | | | | |
| 1.23E-107 | KIF4A | 218355_at | kinesin family member 4A [Source:HGNC Symbol;Acc:HGNC:13339] | | | | | |
| 3.73E-107 | CDCA3 | 223307_at | cell division cycle associated 3 [Source:HGNC Symbol;Acc:HGNC:14624] | | | | | |
| 4.57E-107 | PTTG1 | 203554_x_at | pituitary tumor-transforming 1 [Source:HGNC Symbol;Acc:HGNC:9690] | | | | | |
| 9.64E-107 | ANLN | 222608_s_at | anillin, actin binding protein [Source:HGNC Symbol;Acc:HGNC:14082] | | | | | |
| 1.84E-106 | CEP55 | 218542_at | centrosomal protein 55kDa [Source:HGNC Symbol;Acc:HGNC:1161] | | | | | |
| 6.66E-106 | RAD51AP1 | 204146_at | RAD51 associated protein 1 [Source:HGNC Symbol;Acc:HGNC:16956] | | | | | |
| 1.42E-105 | NUF2 | 223381_at | NUF2, NDC80 kinetochore complex component [Source:HGNC Symbol;Acc:HGNC:14621] | | | | | |
| 6.70E-105 | NCAPG | 218663_at | non-SMC condensin I complex, subunit G [Source:HGNC Symbol;Acc:HGNC:24304] | | | | | |
| 3.11E-104 | CCNA2 | 213226_at | cyclin A2 [Source:HGNC Symbol;Acc:HGNC:1578] | | | | | |
| 1.29E-103 | CENPW | 226936_at | centromere protein W [Source:HGNC Symbol;Acc:HGNC:21488] | | | | | |
| 2.08E-103 | TRIP13 | 204033_at | thyroid hormone receptor interactor 13 [Source:HGNC Symbol;Acc:HGNC:12307] | | | | | |
| 1.91E-102 | BUB1 | 209642_at | BUB1 mitotic checkpoint serine/threonine kinase [Source:HGNC Symbol;Acc:HGNC:1148] | | | | | |
| 7.63E-102 | CASC5 | 228323_at | cancer susceptibility candidate 5 [Source:HGNC Symbol;Acc:HGNC:24054] | | | | | |
| 1.27E-101 | NCAPG | 218662_s_at | non-SMC condensin I complex, subunit G [Source:HGNC Symbol;Acc:HGNC:24304] | | | | | |
| 1.67E-101 | GINS2 | 221521_s_at | GINS complex subunit 2 (Psf2 homolog) [Source:HGNC Symbol;Acc:HGNC:24575] | | | | | |
| 3.07E-101 | CCNA2 | 203418_at | cyclin A2 [Source:HGNC Symbol;Acc:HGNC:1578] | | | | | |
| 5.66E-101 | TTK | 204822_at | TTK protein kinase [Source:HGNC Symbol;Acc:HGNC:12401] | | | | | |
| 1.32E-100 | DTL | 222680_s_at | denticleless E3 ubiquitin protein ligase homolog (Drosophila) [Source:HGNC Symbol;Acc:HGNC:30288] | | | | | |
| 3.81E-100 | SHCBP1 | 219493_at | SHC SH2-domain binding protein 1 [Source:HGNC Symbol;Acc:HGNC:29547] | | | | | |
| 4.75E-100 | AURKA | 208079_s_at | aurora kinase A [Source:HGNC Symbol;Acc:HGNC:11393] | | | | | |
| 6.52E-100 | RACGAP1 | 222077_s_at | Rac GTPase activating protein 1 [Source:HGNC Symbol;Acc:HGNC:9804] | | | | | |
| 9.71E-100 | DEPDC1 | 222958_s_at | DEP domain containing 1 [Source:HGNC Symbol;Acc:HGNC:22949] | | | | | |
| 2.07E-98 | MKI67 | 212022_s_at | marker of proliferation Ki-67 [Source:HGNC Symbol;Acc:HGNC:7107] | | | | | |
| 6.63E-98 | KIF18B | 222039_at | kinesin family member 18B [Source:HGNC Symbol;Acc:HGNC:27102] | | | | | |
| 1.46E-97 | NDC80 | 204162_at | NDC80 kinetochore complex component [Source:HGNC Symbol;Acc:HGNC:16909] | | | | | |
| 3.46E-97 | MCM4 | 222036_s_at | minichromosome maintenance complex component 4 [Source:HGNC Symbol;Acc:HGNC:6947] | | | | | |
| 1.98E-96 | FANCI | 213008_at | Fanconi anemia, complementation group I [Source:HGNC Symbol;Acc:HGNC:25568] | | | | | |
| 3.22E-96 | CDCA5 | 224753_at | cell division cycle associated 5 [Source:HGNC Symbol;Acc:HGNC:14626] | | | | | |
| 9.34E-96 | FOXM1 | 202580_x_at | forkhead box M1 [Source:HGNC Symbol;Acc:HGNC:3818] | | | | | |
| 1.83E-95 | CENPA | 204962_s_at | centromere protein A [Source:HGNC Symbol;Acc:HGNC:1851] | | | | | |
| 7.37E-95 | KIF15 | 219306_at | kinesin family member 15 [Source:HGNC Symbol;Acc:HGNC:17273] | | | | | |
| 1.82E-94 | NEK2 | 204641_at | NIMA-related kinase 2 [Source:HGNC Symbol;Acc:HGNC:7745] | | | | | |
| 1.79E-93 | NCAPG2 | 219588_s_at | non-SMC condensin II complex, subunit G2 [Source:HGNC Symbol;Acc:HGNC:21904] | | | | | |
| 3.73E-93 | NUSAP1 | 219978_s_at | nucleolar and spindle associated protein 1 [Source:HGNC Symbol;Acc:HGNC:18538] | | | | | |
| 5.48E-93 | TYMS | 1554696_s_at | thymidylate synthetase [Source:HGNC Symbol;Acc:HGNC:12441] | | | | | |
| 5.46E-92 | UBE2T | 223229_at | ubiquitin-conjugating enzyme E2T [Source:HGNC Symbol;Acc:HGNC:25009] | | | | | |
| 2.74E-91 | HMMR | 209709_s_at | hyaluronan-mediated motility receptor (RHAMM) [Source:HGNC Symbol;Acc:HGNC:5012] | | | | | |
| 2.81E-91 | CDCA8 | 221520_s_at | cell division cycle associated 8 [Source:HGNC Symbol;Acc:HGNC:14629] | | | | | |
| 6.83E-90 | MCM2 | 202107_s_at | minichromosome maintenance complex component 2 [Source:HGNC Symbol;Acc:HGNC:6944] | | | | | |
| 7.66E-90 | MKI67 | 212021_s_at | marker of proliferation Ki-67 [Source:HGNC Symbol;Acc:HGNC:7107] | | | | | |
| 2.58E-89 | AURKA | 204092_s_at | aurora kinase A [Source:HGNC Symbol;Acc:HGNC:11393] | | | | | |
| 4.71E-89 | OIP5 | 213599_at | Opa interacting protein 5 [Source:HGNC Symbol;Acc:HGNC:20300] | | | | | |
| 1.70E-88 | FAM83D | 225687_at | family with sequence similarity 83, member D [Source:HGNC Symbol;Acc:HGNC:16122] | | | | | |
| 2.73E-88 | KIF23 | 204709_s_at | kinesin family member 23 [Source:HGNC Symbol;Acc:HGNC:6392] | | | | | |
| 3.07E-88 | RNASEH2A | 203022_at | ribonuclease H2, subunit A [Source:HGNC Symbol;Acc:HGNC:18518] | | | | | |
| 3.26E-88 | TK1 | 1554408_a_at | thymidine kinase 1, soluble [Source:HGNC Symbol;Acc:HGNC:11830] | | | | | |
| 9.16E-88 | HJURP | 218726_at | Holliday junction recognition protein [Source:HGNC Symbol;Acc:HGNC:25444] | | | | | |
| 2.46E-87 | SPC25 | 209891_at | SPC25, NDC80 kinetochore complex component [Source:HGNC Symbol;Acc:HGNC:24031] | | | | | |
| 4.97E-87 | POLE2 | 205909_at | polymerase (DNA directed), epsilon 2, accessory subunit [Source:HGNC Symbol;Acc:HGNC:9178] | | | | | |
| 3.07E-86 | TK1 | 202338_at | thymidine kinase 1, soluble [Source:HGNC Symbol;Acc:HGNC:11830] | | | | | |
| 3.73E-86 | CCNB1 | 228729_at | cyclin B1 [Source:HGNC Symbol;Acc:HGNC:1579] | | | | | |
| 4.40E-86 | CDCA2 | 226661_at | cell division cycle associated 2 [Source:HGNC Symbol;Acc:HGNC:14623] | | | | | |
| 1.33E-85 | KIF14 | 206364_at | kinesin family member 14 [Source:HGNC Symbol;Acc:HGNC:19181] | | | | | |
| 1.88E-84 | CDCA7 | 224428_s_at | cell division cycle associated 7 [Source:HGNC Symbol;Acc:HGNC:14628] | | | | | |
| 3.46E-84 | CENPE | 205046_at | centromere protein E, 312kDa [Source:HGNC Symbol;Acc:HGNC:1856] | | | | | |
| 3.72E-84 | MCM6 | 201930_at | minichromosome maintenance complex component 6 [Source:HGNC Symbol;Acc:HGNC:6949] | | | | | |
| 4.61E-84 | BRIP1 | 235609_at | BRCA1 interacting protein C-terminal helicase 1 [Source:HGNC Symbol;Acc:HGNC:20473] | | | | | |
| 5.06E-84 | ZWILCH | 222606_at | zwilch kinetochore protein [Source:HGNC Symbol;Acc:HGNC:25468] | | | | | |
| 7.31E-84 | CENPK | 222848_at | centromere protein K [Source:HGNC Symbol;Acc:HGNC:29479] | | | | | |
| 8.60E-84 | ECT2 | 219787_s_at | epithelial cell transforming 2 [Source:HGNC Symbol;Acc:HGNC:3155] | | | | | |
| 3.30E-83 | FEN1 | 204767_s_at | flap structure-specific endonuclease 1 [Source:HGNC Symbol;Acc:HGNC:3650] | | | | | |
| 5.95E-83 | PCNA | 201202_at | proliferating cell nuclear antigen [Source:HGNC Symbol;Acc:HGNC:8729] | | | | | |
| 9.33E-83 | CHEK1 | 205394_at | checkpoint kinase 1 [Source:HGNC Symbol;Acc:HGNC:1925] | | | | | |
| 1.72E-82 | CKS2 | 204170_s_at | CDC28 protein kinase regulatory subunit 2 [Source:HGNC Symbol;Acc:HGNC:2000] | | | | | |
| 5.18E-81 | RAD51 | 205024_s_at | RAD51 recombinase [Source:HGNC Symbol;Acc:HGNC:9817] | | | | | |
| 6.01E-81 | SGOL2 | 230165_at | shugoshin-like 2 (S. pombe) [Source:HGNC Symbol;Acc:HGNC:30812] | | | | | |
| 6.75E-81 | FANCD2 | 242560_at | Fanconi anemia, complementation group D2 [Source:HGNC Symbol;Acc:HGNC:3585] | | | | | |
| 1.67E-80 | STIL | 205339_at | SCL/TAL1 interrupting locus [Source:HGNC Symbol;Acc:HGNC:10879] | | | | | |
| 1.93E-80 | KIF18A | 221258_s_at | kinesin family member 18A [Source:HGNC Symbol;Acc:HGNC:29441] | | | | | |
| 1.20E-79 | RFC4 | 204023_at | replication factor C (activator 1) 4, 37kDa [Source:HGNC Symbol;Acc:HGNC:9972] | | | | | |
| 1.37E-79 | CENPN | 219555_s_at | centromere protein N [Source:HGNC Symbol;Acc:HGNC:30873] | | | | | |
| 4.53E-79 | CHEK1 | 205393_s_at | checkpoint kinase 1 [Source:HGNC Symbol;Acc:HGNC:1925] | | | | | |
| 7.62E-79 | CDC45 | 204126_s_at | cell division cycle 45 [Source:HGNC Symbol;Acc:HGNC:1739] | | | | | |
| 3.27E-78 | PLK4 | 204887_s_at | polo-like kinase 4 [Source:HGNC Symbol;Acc:HGNC:11397] | | | | | |
| 6.01E-78 | RFC3 | 204127_at | replication factor C (activator 1) 3, 38kDa [Source:HGNC Symbol;Acc:HGNC:9971] | | | | | |
| 2.96E-77 | AURKB | 209464_at | aurora kinase B [Source:HGNC Symbol;Acc:HGNC:11390] | | | | | |
| 3.43E-77 | SPAG5 | 203145_at | sperm associated antigen 5 [Source:HGNC Symbol;Acc:HGNC:13452] | | | | | |
| 1.17E-76 | NCAPH | 212949_at | non-SMC condensin I complex, subunit H [Source:HGNC Symbol;Acc:HGNC:1112] | | | | | |
| 1.42E-76 | CENPM | 218741_at | centromere protein M [Source:HGNC Symbol;Acc:HGNC:18352] | | | | | |
| 3.43E-76 | SMC2 | 204240_s_at | structural maintenance of chromosomes 2 [Source:HGNC Symbol;Acc:HGNC:14011] | | | | | |
| 1.84E-75 | IQGAP3 | 229538_s_at | IQ motif containing GTPase activating protein 3 [Source:HGNC Symbol;Acc:HGNC:20669] | | | | | |
| 4.23E-75 | BIRC5 | 202094_at | baculoviral IAP repeat containing 5 [Source:HGNC Symbol;Acc:HGNC:593] | | | | | |
| 5.96E-75 | DEPDC1 | 235545_at | DEP domain containing 1 [Source:HGNC Symbol;Acc:HGNC:22949] | | | | | |
| 1.06E-74 | NCAPD2 | 201774_s_at | non-SMC condensin I complex, subunit D2 [Source:HGNC Symbol;Acc:HGNC:24305] | | | | | |
| 1.54E-74 | DTYMK | 1553984_s_at | deoxythymidylate kinase (thymidylate kinase) [Source:HGNC Symbol;Acc:HGNC:3061] | | | | | |
| 1.56E-74 | DTYMK | 203270_at | deoxythymidylate kinase (thymidylate kinase) [Source:HGNC Symbol;Acc:HGNC:3061] | | | | | |
| 1.84E-74 | KIF14 | 236641_at | kinesin family member 14 [Source:HGNC Symbol;Acc:HGNC:19181] | | | | | |
| 3.61E-74 | MCM10 | 220651_s_at | minichromosome maintenance complex component 10 [Source:HGNC Symbol;Acc:HGNC:18043] | | | | | |
| 4.03E-74 | MND1 | 223700_at | meiotic nuclear divisions 1 homolog (S. cerevisiae) [Source:HGNC Symbol;Acc:HGNC:24839] | | | | | |
| 8.04E-74 | ANLN | 1552619_a_at | anillin, actin binding protein [Source:HGNC Symbol;Acc:HGNC:14082] | | | | | |
| 1.17E-73 | CENPF | 209172_s_at | centromere protein F, 350/400kDa [Source:HGNC Symbol;Acc:HGNC:1857] | | | | | |
| 1.93E-72 | MCM3 | 201555_at | minichromosome maintenance complex component 3 [Source:HGNC Symbol;Acc:HGNC:6945] | | | | | |
| 2.50E-72 | MKI67 | 212023_s_at | marker of proliferation Ki-67 [Source:HGNC Symbol;Acc:HGNC:7107] | | | | | |
| 2.72E-72 | GTSE1 | 204318_s_at | G-2 and S-phase expressed 1 [Source:HGNC Symbol;Acc:HGNC:13698] | | | | | |
| 8.53E-72 | RRM1 | 201477_s_at | ribonucleotide reductase M1 [Source:HGNC Symbol;Acc:HGNC:10451] | | | | | |
| 1.67E-71 | PRIM1 | 205053_at | primase, DNA, polypeptide 1 (49kDa) [Source:HGNC Symbol;Acc:HGNC:9369] | | | | | |
| 1.88E-71 | CDC6 | 203968_s_at | cell division cycle 6 [Source:HGNC Symbol;Acc:HGNC:1744] | | | | | |
| 3.87E-71 | CENPH | 231772_x_at | centromere protein H [Source:HGNC Symbol;Acc:HGNC:17268] | | | | | |
| 5.69E-71 | CDCA3 | 221436_s_at | cell division cycle associated 3 [Source:HGNC Symbol;Acc:HGNC:14624] | | | | | |
| 7.35E-71 | KIF2C | 211519_s_at | kinesin family member 2C [Source:HGNC Symbol;Acc:HGNC:6393] | | | | | |
| 1.19E-70 | MTFR2 | 228069_at | mitochondrial fission regulator 2 [Source:HGNC Symbol;Acc:HGNC:21115] | | | | | |
| 1.40E-70 | FBXO5 | 218875_s_at | F-box protein 5 [Source:HGNC Symbol;Acc:HGNC:13584] | | | | | |
| 1.79E-70 | PARPBP | 220060_s_at | PARP1 binding protein [Source:HGNC Symbol;Acc:HGNC:26074] | | | | | |
| 2.22E-70 | ASF1B | 218115_at | anti-silencing function 1B histone chaperone [Source:HGNC Symbol;Acc:HGNC:20996] | | | | | |
| 2.87E-70 | CDC6 | 203967_at | cell division cycle 6 [Source:HGNC Symbol;Acc:HGNC:1744] | | | | | |
| 4.40E-70 | HELLS | 223556_at | helicase, lymphoid-specific [Source:HGNC Symbol;Acc:HGNC:4861] | | | | | |
| 2.33E-69 | SMC4 | 201663_s_at | structural maintenance of chromosomes 4 [Source:HGNC Symbol;Acc:HGNC:14013] | | | | | |
| 2.71E-69 | EZH2 | 203358_s_at | enhancer of zeste 2 polycomb repressive complex 2 subunit [Source:HGNC Symbol;Acc:HGNC:3527] | | | | | |
| 4.34E-69 | PLK4 | 204886_at | polo-like kinase 4 [Source:HGNC Symbol;Acc:HGNC:11397] | | | | | |
| 4.65E-69 | TIMELESS | 203046_s_at | timeless circadian clock [Source:HGNC Symbol;Acc:HGNC:11813] | | | | | |
| 4.65E-69 | ESPL1 | 38158_at | extra spindle pole bodies homolog 1 (S. cerevisiae) [Source:HGNC Symbol;Acc:HGNC:16856] | | | | | |
| 4.90E-69 | HELLS | 227350_at | helicase, lymphoid-specific [Source:HGNC Symbol;Acc:HGNC:4861] | | | | | |
| 7.72E-69 | H2AFZ | 200853_at | H2A histone family, member Z [Source:HGNC Symbol;Acc:HGNC:4741] | | | | | |
| 8.73E-69 | SKA3 | 227165_at | spindle and kinetochore associated complex subunit 3 [Source:HGNC Symbol;Acc:HGNC:20262] | | | | | |
| 1.02E-68 | RAD54L | 204558_at | RAD54-like (S. cerevisiae) [Source:HGNC Symbol;Acc:HGNC:9826] | | | | | |
| 1.30E-68 | DIAPH3 | 229097_at | diaphanous-related formin 3 [Source:HGNC Symbol;Acc:HGNC:15480] | | | | | |
| 1.76E-68 | ZWILCH | 218349_s_at | zwilch kinetochore protein [Source:HGNC Symbol;Acc:HGNC:25468] | | | | | |
| 1.87E-68 | E2F8 | 219990_at | E2F transcription factor 8 [Source:HGNC Symbol;Acc:HGNC:24727] | | | | | |
| 3.22E-68 | CKAP2L | 229610_at | cytoskeleton associated protein 2-like [Source:HGNC Symbol;Acc:HGNC:26877] | | | | | |
| 5.66E-68 | WHSC1 | 209053_s_at | Wolf-Hirschhorn syndrome candidate 1 [Source:HGNC Symbol;Acc:HGNC:12766] | | | | | |
| 7.54E-68 | HN1 | 217755_at | hematological and neurological expressed 1 [Source:HGNC Symbol;Acc:HGNC:14569] | | | | | |
| 9.10E-68 | BIRC5 | 210334_x_at | baculoviral IAP repeat containing 5 [Source:HGNC Symbol;Acc:HGNC:593] | | | | | |
| 1.68E-67 | SMC4 | 201664_at | structural maintenance of chromosomes 4 [Source:HGNC Symbol;Acc:HGNC:14013] | | | | | |
| 3.43E-67 | HMGB2 | 208808_s_at | high mobility group box 2 [Source:HGNC Symbol;Acc:HGNC:5000] | | | | | |
| 3.80E-67 | ORC6 | 219105_x_at | origin recognition complex, subunit 6 [Source:HGNC Symbol;Acc:HGNC:17151] | | | | | |
| 5.05E-67 | MCM4 | 222037_at | minichromosome maintenance complex component 4 [Source:HGNC Symbol;Acc:HGNC:6947] | | | | | |
| 5.75E-67 | STMN1 | 200783_s_at | stathmin 1 [Source:HGNC Symbol;Acc:HGNC:6510] | | | | | |
| 8.23E-67 | CDC25C | 205167_s_at | cell division cycle 25C [Source:HGNC Symbol;Acc:HGNC:1727] | | | | | |
| 1.04E-66 | DDIAS | 228281_at | DNA damage-induced apoptosis suppressor [Source:HGNC Symbol;Acc:HGNC:26351] | | | | | |
| 2.89E-66 | BRCA1 | 204531_s_at | breast cancer 1, early onset [Source:HGNC Symbol;Acc:HGNC:1100] | | | | | |
| 4.23E-66 | TCF19 | 223274_at | transcription factor 19 [Source:HGNC Symbol;Acc:HGNC:11629] | | | | | |
| 1.17E-65 | RFC5 | 203209_at | replication factor C (activator 1) 5, 36.5kDa [Source:HGNC Symbol;Acc:HGNC:9973] | | | | | |
| 1.56E-65 | MCM10 | 222962_s_at | minichromosome maintenance complex component 10 [Source:HGNC Symbol;Acc:HGNC:18043] | | | | | |
| 7.69E-65 | DEPDC1B | 226980_at | DEP domain containing 1B [Source:HGNC Symbol;Acc:HGNC:24902] | | | | | |
| 1.26E-64 | KNSTRN | 225300_at | kinetochore-localized astrin/SPAG5 binding protein [Source:HGNC Symbol;Acc:HGNC:30767] | | | | | |
| 1.32E-64 | TACC3 | 218308_at | transforming, acidic coiled-coil containing protein 3 [Source:HGNC Symbol;Acc:HGNC:11524] | | | | | |
| 1.40E-64 | CCNE2 | 205034_at | cyclin E2 [Source:HGNC Symbol;Acc:HGNC:1590] | | | | | |
| 1.15E-63 | SPDL1 | 221685_s_at | spindle apparatus coiled-coil protein 1 [Source:HGNC Symbol;Acc:HGNC:26010] | | | | | |
| 1.49E-63 | GMNN | 218350_s_at | geminin, DNA replication inhibitor [Source:HGNC Symbol;Acc:HGNC:17493] | | | | | |
| 1.72E-63 | EXO1 | 204603_at | exonuclease 1 [Source:HGNC Symbol;Acc:HGNC:3511] | | | | | |
| 1.74E-63 | CKAP2 | 218252_at | cytoskeleton associated protein 2 [Source:HGNC Symbol;Acc:HGNC:1990] | | | | | |
| 2.85E-63 | SKA1 | 217640_x_at | spindle and kinetochore associated complex subunit 1 [Source:HGNC Symbol;Acc:HGNC:28109] | | | | | |
| 3.11E-63 | CHEK1 | 238075_at | checkpoint kinase 1 [Source:HGNC Symbol;Acc:HGNC:1925] | | | | | |
| 3.94E-63 | H2AFZ | 213911_s_at | H2A histone family, member Z [Source:HGNC Symbol;Acc:HGNC:4741] | | | | | |
| 6.42E-63 | KNTC1 | 206316_s_at | kinetochore associated 1 [Source:HGNC Symbol;Acc:HGNC:17255] | | | | | |
| 8.07E-63 | WDHD1 | 216228_s_at | WD repeat and HMG-box DNA binding protein 1 [Source:HGNC Symbol;Acc:HGNC:23170] | | | | | |
| 9.34E-63 | MCM5 | 216237_s_at | minichromosome maintenance complex component 5 [Source:HGNC Symbol;Acc:HGNC:6948] | | | | | |
| 1.48E-62 | LMNB1 | 203276_at | lamin B1 [Source:HGNC Symbol;Acc:HGNC:6637] | | | | | |
| 1.64E-62 | FBXO5 | 234863_x_at | F-box protein 5 [Source:HGNC Symbol;Acc:HGNC:13584] | | | | | |
| 2.17E-62 | ATAD2 | 218782_s_at | ATPase family, AAA domain containing 2 [Source:HGNC Symbol;Acc:HGNC:30123] | | | | | |
| 2.91E-62 | NUP37 | 218622_at | nucleoporin 37kDa [Source:HGNC Symbol;Acc:HGNC:29929] | | | | | |
| 6.26E-62 | PARPBP | 227928_at | PARP1 binding protein [Source:HGNC Symbol;Acc:HGNC:26074] | | | | | |
| 7.64E-62 | RMI2 | 226456_at | RecQ mediated genome instability 2 [Source:HGNC Symbol;Acc:HGNC:28349] | | | | | |
| 1.06E-61 | MCM4 | 212141_at | minichromosome maintenance complex component 4 [Source:HGNC Symbol;Acc:HGNC:6947] | | | | | |
| 1.58E-61 | ATAD2 | 222740_at | ATPase family, AAA domain containing 2 [Source:HGNC Symbol;Acc:HGNC:30123] | | | | | |
| 2.44E-61 | FAM64A | 221591_s_at | family with sequence similarity 64, member A [Source:HGNC Symbol;Acc:HGNC:25483] | | | | | |
| 3.34E-61 | MIS18A | 228597_at | MIS18 kinetochore protein A [Source:HGNC Symbol;Acc:HGNC:1286] | | | | | |
| 3.56E-61 | RFC3 | 204128_s_at | replication factor C (activator 1) 3, 38kDa [Source:HGNC Symbol;Acc:HGNC:9971] | | | | | |
| 4.49E-61 | BORA | 219544_at | bora, aurora kinase A activator [Source:HGNC Symbol;Acc:HGNC:24724] | | | | | |
| 7.34E-61 | SPC24 | 235572_at | SPC24, NDC80 kinetochore complex component [Source:HGNC Symbol;Acc:HGNC:26913] | | | | | |
| 8.93E-61 | VRK1 | 203856_at | vaccinia related kinase 1 [Source:HGNC Symbol;Acc:HGNC:12718] | | | | | |
| 8.93E-61 | FEN1 | 204768_s_at | flap structure-specific endonuclease 1 [Source:HGNC Symbol;Acc:HGNC:3650] | | | | | |
| 1.25E-60 | DHFR | 202534_x_at | dihydrofolate reductase [Source:HGNC Symbol;Acc:HGNC:2861] | | | | | |
| 1.39E-60 | MKI67 | 212020_s_at | marker of proliferation Ki-67 [Source:HGNC Symbol;Acc:HGNC:7107] | | | | | |
| 1.59E-60 | SNRPF | 203832_at | small nuclear ribonucleoprotein polypeptide F [Source:HGNC Symbol;Acc:HGNC:11162] | | | | | |
| 3.45E-60 | ZNF367 | 229551_x_at | zinc finger protein 367 [Source:HGNC Symbol;Acc:HGNC:18320] | | | | | |
| 5.10E-60 | TROAP | 204649_at | trophinin associated protein [Source:HGNC Symbol;Acc:HGNC:12327] | | | | | |
| 6.49E-60 | LMNB2 | 216952_s_at | lamin B2 [Source:HGNC Symbol;Acc:HGNC:6638] | | | | | |
| 7.57E-60 | SAE1 | 217946_s_at | SUMO1 activating enzyme subunit 1 [Source:HGNC Symbol;Acc:HGNC:30660] | | | | | |
| 8.68E-60 | TMPO | 203432_at | thymopoietin [Source:HGNC Symbol;Acc:HGNC:11875] | | | | | |
| 8.71E-60 | ESPL1 | 204817_at | extra spindle pole bodies homolog 1 (S. cerevisiae) [Source:HGNC Symbol;Acc:HGNC:16856] | | | | | |
| 8.99E-60 | H2AFX | 205436_s_at | H2A histone family, member X [Source:HGNC Symbol;Acc:HGNC:4739] | | | | | |
| 1.92E-59 | DEPDC1 | 220295_x_at | DEP domain containing 1 [Source:HGNC Symbol;Acc:HGNC:22949] | | | | | |
| 2.05E-59 | CDT1 | 209832_s_at | chromatin licensing and DNA replication factor 1 [Source:HGNC Symbol;Acc:HGNC:24576] | | | | | |
| 3.15E-59 | KIFC1 | 209680_s_at | kinesin family member C1 [Source:HGNC Symbol;Acc:HGNC:6389] | | | | | |
| 3.36E-59 | DSCC1 | 219000_s_at | DNA replication and sister chromatid cohesion 1 [Source:HGNC Symbol;Acc:HGNC:24453] | | | | | |
| 4.28E-59 | POC1A | 226355_at | POC1 centriolar protein A [Source:HGNC Symbol;Acc:HGNC:24488] | | | | | |
| 6.95E-59 | KIAA0101 | 211713_x_at | KIAA0101 [Source:HGNC Symbol;Acc:HGNC:28961] | | | | | |
| 1.68E-58 | DONSON | 221677_s_at | downstream neighbor of SON [Source:HGNC Symbol;Acc:HGNC:2993] | | | | | |
| 2.05E-58 | HAUS1 | 225297_at | HAUS augmin-like complex, subunit 1 [Source:HGNC Symbol;Acc:HGNC:25174] | | | | | |
| 2.21E-58 | LRR1 | 235113_at | leucine rich repeat protein 1 [Source:HGNC Symbol;Acc:HGNC:19742] | | | | | |
| 2.98E-58 | POLQ | 219510_at | polymerase (DNA directed), theta [Source:HGNC Symbol;Acc:HGNC:9186] | | | | | |
| 3.31E-58 | BLM | 205733_at | Bloom syndrome, RecQ helicase-like [Source:HGNC Symbol;Acc:HGNC:1058] | | | | | |
| 3.63E-58 | FANCI | 223785_at | Fanconi anemia, complementation group I [Source:HGNC Symbol;Acc:HGNC:25568] | | | | | |
| 2.51E-57 | SUV39H2 | 1554572_a_at | suppressor of variegation 3-9 homolog 2 (Drosophila) [Source:HGNC Symbol;Acc:HGNC:17287] | | | | | |
| 6.83E-57 | CENPO | 226118_at | centromere protein O [Source:HGNC Symbol;Acc:HGNC:28152] | | | | | |
| 1.34E-56 | GTSE1 | 215942_s_at | G-2 and S-phase expressed 1 [Source:HGNC Symbol;Acc:HGNC:13698] | | | | | |
| 1.58E-56 | MCM7 | 210983_s_at | minichromosome maintenance complex component 7 [Source:HGNC Symbol;Acc:HGNC:6950] | | | | | |
| 1.83E-56 | MYBL2 | 201710_at | v-myb avian myeloblastosis viral oncogene homolog-like 2 [Source:HGNC Symbol;Acc:HGNC:7548] | | | | | |
| 2.97E-56 | CENPU | 229305_at | centromere protein U [Source:HGNC Symbol;Acc:HGNC:21348] | | | | | |
| 3.09E-56 | MCM8 | 224320_s_at | minichromosome maintenance complex component 8 [Source:HGNC Symbol;Acc:HGNC:16147] | | | | | |
| 5.49E-56 | NEIL3 | 219502_at | nei endonuclease VIII-like 3 (E. coli) [Source:HGNC Symbol;Acc:HGNC:24573] | | | | | |
| 5.51E-56 | CDC25A | 204695_at | cell division cycle 25A [Source:HGNC Symbol;Acc:HGNC:1725] | | | | | |
| 5.75E-56 | RFC5 | 203210_s_at | replication factor C (activator 1) 5, 36.5kDa [Source:HGNC Symbol;Acc:HGNC:9973] | | | | | |
| 6.29E-56 | CSE1L | 210766_s_at | CSE1 chromosome segregation 1-like (yeast) [Source:HGNC Symbol;Acc:HGNC:2431] | | | | | |
| 1.26E-55 | DHFR | 48808_at | dihydrofolate reductase [Source:HGNC Symbol;Acc:HGNC:2861] | | | | | |
| 1.49E-55 | MCM5 | 201755_at | minichromosome maintenance complex component 5 [Source:HGNC Symbol;Acc:HGNC:6948] | | | | | |
| 4.01E-55 | NUDT1 | 204766_s_at | nudix (nucleoside diphosphate linked moiety X)-type motif 1 [Source:HGNC Symbol;Acc:HGNC:8048] | | | | | |
| 4.22E-55 | TUBB | 212320_at | tubulin, beta class I [Source:HGNC Symbol;Acc:HGNC:20778] | | | | | |
| 4.35E-55 | GGH | 203560_at | gamma-glutamyl hydrolase (conjugase, folylpolygammaglutamyl hydrolase) [Source:HGNC Symbol;Acc:HGNC:4248] | | | | | |
| 6.07E-55 | RPA3 | 209507_at | replication protein A3, 14kDa [Source:HGNC Symbol;Acc:HGNC:10291] | | | | | |
| 7.85E-55 | RUVBL2 | 1559946_s_at | RuvB-like AAA ATPase 2 [Source:HGNC Symbol;Acc:HGNC:10475] | | | | | |
| 9.39E-55 | NPM1 | 221923_s_at | nucleophosmin (nucleolar phosphoprotein B23, numatrin) [Source:HGNC Symbol;Acc:HGNC:7910] | | | | | |
| 9.40E-55 | CENPI | 214804_at | centromere protein I [Source:HGNC Symbol;Acc:HGNC:3968] | | | | | |
| 9.65E-55 | CSE1L | 201112_s_at | CSE1 chromosome segregation 1-like (yeast) [Source:HGNC Symbol;Acc:HGNC:2431] | | | | | |
| 9.92E-55 | SNRPD1 | 202690_s_at | small nuclear ribonucleoprotein D1 polypeptide 16kDa [Source:HGNC Symbol;Acc:HGNC:11158] | | | | | |
| 1.07E-54 | HELLS | 220085_at | helicase, lymphoid-specific [Source:HGNC Symbol;Acc:HGNC:4861] | | | | | |
| 1.07E-54 | CDT1 | 228868_x_at | chromatin licensing and DNA replication factor 1 [Source:HGNC Symbol;Acc:HGNC:24576] | | | | | |
| 1.13E-54 | SMC2 | 213253_at | structural maintenance of chromosomes 2 [Source:HGNC Symbol;Acc:HGNC:14011] | | | | | |
| 1.30E-54 | C1ORF112 | 220840_s_at | chromosome 1 open reading frame 112 [Source:HGNC Symbol;Acc:HGNC:25565] | | | | | |
| 1.75E-54 | NDC1 | 234672_s_at | NDC1 transmembrane nucleoporin [Source:HGNC Symbol;Acc:HGNC:25525] | | | | | |
| 7.35E-54 | RP11-303E16.2 | 228559_at | N/A | |  |  |  |  |
| 7.62E-54 | LSM5 | 211747_s_at | LSM5 homolog, U6 small nuclear RNA associated (S. cerevisiae) [Source:HGNC Symbol;Acc:HGNC:17162] | | | | | |
| 1.04E-53 | ORC1 | 205085_at | origin recognition complex, subunit 1 [Source:HGNC Symbol;Acc:HGNC:8487] | | | | | |
| 1.08E-53 | RMI1 | 218979_at | RecQ mediated genome instability 1 [Source:HGNC Symbol;Acc:HGNC:25764] | | | | | |
| 1.12E-53 | HMGB3 | 203744_at | high mobility group box 3 [Source:HGNC Symbol;Acc:HGNC:5004] | | | | | |
| 1.86E-53 | CHAF1A | 214426_x_at | chromatin assembly factor 1, subunit A (p150) [Source:HGNC Symbol;Acc:HGNC:1910] | | | | | |
| 2.77E-53 | PSRC1 | 201896_s_at | proline/serine-rich coiled-coil 1 [Source:HGNC Symbol;Acc:HGNC:24472] | | | | | |
| 5.52E-53 | WHSC1 | 209054_s_at | Wolf-Hirschhorn syndrome candidate 1 [Source:HGNC Symbol;Acc:HGNC:12766] | | | | | |
| 1.03E-52 | TUBB | 211714_x_at | tubulin, beta class I [Source:HGNC Symbol;Acc:HGNC:20778] | | | | | |
| 1.38E-52 | ATAD2 | 228401_at | ATPase family, AAA domain containing 2 [Source:HGNC Symbol;Acc:HGNC:30123] | | | | | |
| 3.48E-52 | RFC2 | 203696_s_at | replication factor C (activator 1) 2, 40kDa [Source:HGNC Symbol;Acc:HGNC:9970] | | | | | |
| 4.38E-52 | WDHD1 | 204728_s_at | WD repeat and HMG-box DNA binding protein 1 [Source:HGNC Symbol;Acc:HGNC:23170] | | | | | |
| 4.70E-52 | SGOL2 | 235425_at | shugoshin-like 2 (S. pombe) [Source:HGNC Symbol;Acc:HGNC:30812] | | | | | |
| 4.81E-52 | PRIM2 | 205628_at | primase, DNA, polypeptide 2 (58kDa) [Source:HGNC Symbol;Acc:HGNC:9370] | | | | | |
| 6.95E-52 | PAICS | 201013_s_at | phosphoribosylaminoimidazole carboxylase, phosphoribosylaminoimidazole succinocarboxamide synthetase [Source:HGNC Symbol;Acc:HGNC:8587] | | | | | |
| 7.58E-52 | PPIH | 204228_at | peptidylprolyl isomerase H (cyclophilin H) [Source:HGNC Symbol;Acc:HGNC:14651] | | | | | |
| 8.24E-52 | TUBG1 | 201714_at | tubulin, gamma 1 [Source:HGNC Symbol;Acc:HGNC:12417] | | | | | |
| 1.21E-51 | SNRPC | 201342_at | small nuclear ribonucleoprotein polypeptide C [Source:HGNC Symbol;Acc:HGNC:11157] | | | | | |
| 2.01E-51 | CDCA4 | 218399_s_at | cell division cycle associated 4 [Source:HGNC Symbol;Acc:HGNC:14625] | | | | | |
| 2.88E-51 | CBX3 | 201091_s_at | chromobox homolog 3 [Source:HGNC Symbol;Acc:HGNC:1553] | | | | | |
| 3.12E-51 | CCDC34 | 226287_at | coiled-coil domain containing 34 [Source:HGNC Symbol;Acc:HGNC:25079] | | | | | |
| 3.33E-51 | RAD54B | 219494_at | RAD54 homolog B (S. cerevisiae) [Source:HGNC Symbol;Acc:HGNC:17228] | | | | | |
| 3.50E-51 | MIS18A | 219004_s_at | MIS18 kinetochore protein A [Source:HGNC Symbol;Acc:HGNC:1286] | | | | | |
| 3.76E-51 | SKP2 | 211042_x_at | S-phase kinase-associated protein 2, E3 ubiquitin protein ligase [Source:HGNC Symbol;Acc:HGNC:10901] | | | | | |
| 3.94E-51 | RFC2 | 1053_at | replication factor C (activator 1) 2, 40kDa [Source:HGNC Symbol;Acc:HGNC:9970] | | | | | |
| 4.42E-51 | BARD1 | 227545_at | BRCA1 associated RING domain 1 [Source:HGNC Symbol;Acc:HGNC:952] | | | | | |
| 4.74E-51 | GINS3 | 45633_at | GINS complex subunit 3 (Psf3 homolog) [Source:HGNC Symbol;Acc:HGNC:25851] | | | | | |
| 5.46E-51 | PLK1 | 202240_at | polo-like kinase 1 [Source:HGNC Symbol;Acc:HGNC:9077] | | | | | |
| 6.33E-51 | NDC1 | 218073_s_at | NDC1 transmembrane nucleoporin [Source:HGNC Symbol;Acc:HGNC:25525] | | | | | |
| 7.19E-51 | TIPIN | 219258_at | TIMELESS interacting protein [Source:HGNC Symbol;Acc:HGNC:30750] | | | | | |
| 7.36E-51 | CCT5 | 208696_at | chaperonin containing TCP1, subunit 5 (epsilon) [Source:HGNC Symbol;Acc:HGNC:1618] | | | | | |
| 1.06E-50 | MCM10 | 223570_at | minichromosome maintenance complex component 10 [Source:HGNC Symbol;Acc:HGNC:18043] | | | | | |
| 1.27E-50 | C4ORF46 | 238015_at | chromosome 4 open reading frame 46 [Source:HGNC Symbol;Acc:HGNC:27320] | | | | | |
| 1.51E-50 | CDC25A | 1555772_a_at | cell division cycle 25A [Source:HGNC Symbol;Acc:HGNC:1725] | | | | | |
| 2.22E-50 | KIAA1524 | 231855_at | KIAA1524 [Source:HGNC Symbol;Acc:HGNC:29302] | | | | | |
| 3.25E-50 | ANP32E | 208103_s_at | acidic (leucine-rich) nuclear phosphoprotein 32 family, member E [Source:HGNC Symbol;Acc:HGNC:16673] | | | | | |
| 3.87E-50 | C4ORF46 | 235088_at | chromosome 4 open reading frame 46 [Source:HGNC Symbol;Acc:HGNC:27320] | | | | | |
| 8.04E-50 | DIAPH3 | 232596_at | diaphanous-related formin 3 [Source:HGNC Symbol;Acc:HGNC:15480] | | | | | |
| 8.23E-50 | ERCC6L | 219650_at | excision repair cross-complementation group 6-like [Source:HGNC Symbol;Acc:HGNC:20794] | | | | | |
| 9.95E-50 | ASPM | 232238_at | asp (abnormal spindle) homolog, microcephaly associated (Drosophila) [Source:HGNC Symbol;Acc:HGNC:19048] | | | | | |
| 1.15E-49 | PSMC3IP | 213951_s_at | PSMC3 interacting protein [Source:HGNC Symbol;Acc:HGNC:17928] | | | | | |
| 1.31E-49 | UCK2 | 209825_s_at | uridine-cytidine kinase 2 [Source:HGNC Symbol;Acc:HGNC:12562] | | | | | |
| 1.45E-49 | GTSE1 | 204315_s_at | G-2 and S-phase expressed 1 [Source:HGNC Symbol;Acc:HGNC:13698] | | | | | |
| 1.97E-49 | DHFR | 202532_s_at | dihydrofolate reductase [Source:HGNC Symbol;Acc:HGNC:2861] | | | | | |
| 1.99E-49 | TMPO | 224944_at | thymopoietin [Source:HGNC Symbol;Acc:HGNC:11875] | | | | | |
| 2.35E-49 | DNMT1 | 201697_s_at | DNA (cytosine-5-)-methyltransferase 1 [Source:HGNC Symbol;Acc:HGNC:2976] | | | | | |
| 2.82E-49 | CDK2 | 204252_at | cyclin-dependent kinase 2 [Source:HGNC Symbol;Acc:HGNC:1771] | | | | | |
| 3.32E-49 | EME1 | 234464_s_at | essential meiotic structure-specific endonuclease 1 [Source:HGNC Symbol;Acc:HGNC:24965] | | | | | |
| 3.74E-49 | BUB1 | 215509_s_at | BUB1 mitotic checkpoint serine/threonine kinase [Source:HGNC Symbol;Acc:HGNC:1148] | | | | | |
| 4.47E-49 | HNRNPAB | 201277_s_at | heterogeneous nuclear ribonucleoprotein A/B [Source:HGNC Symbol;Acc:HGNC:5034] | | | | | |
| 9.90E-49 | CKAP5 | 212832_s_at | cytoskeleton associated protein 5 [Source:HGNC Symbol;Acc:HGNC:28959] | | | | | |
| 1.05E-48 | CDC7 | 204510_at | cell division cycle 7 [Source:HGNC Symbol;Acc:HGNC:1745] | | | | | |
| 1.40E-48 | UNG | 202330_s_at | uracil-DNA glycosylase [Source:HGNC Symbol;Acc:HGNC:12572] | | | | | |
