# Supplementary material for: PCNA-associated factor KIAA0101 transcriptionally induced by ELK1 controls cell proliferation and apoptosis in nasopharyngeal carcinoma: an integrated bioinformatics and experimental study
Source: Aging (Albany NY). 2020 Apr 9;12(7):5992–6017. doi: 10.18632/aging.102991 (PMC7185143; doi:10.18632/aging.102991)
Supplement: Supplementary Table 5 [file aging-12-102991-s001..docx]

**Supplementary Table 5**. **MEM analysis of tumor query datasets obtains 300 KIAA0101 co-expressed gene.**

| #Score | #Gene name | #Gene description |
| --- | --- | --- |
| #QUERY | KIAA0101 | KIAA0101 [Source:HGNC Symbol;Acc:HGNC:28961] |
| 4.71E-99 | KIAA0101 | KIAA0101 [Source:HGNC Symbol;Acc:HGNC:28961] |
| 7.78E-91 | GTSE1 | G-2 and S-phase expressed 1 [Source:HGNC Symbol;Acc:HGNC:13698] |
| 2.94E-88 | NCAPG | non-SMC condensin I complex, subunit G [Source:HGNC Symbol;Acc:HGNC:24304] |
| 5.66E-84 | ORC6 | origin recognition complex, subunit 6 [Source:HGNC Symbol;Acc:HGNC:17151] |
| 1.56E-83 | GTSE1 | G-2 and S-phase expressed 1 [Source:HGNC Symbol;Acc:HGNC:13698] |
| 2.52E-80 | NCAPG | non-SMC condensin I complex, subunit G [Source:HGNC Symbol;Acc:HGNC:24304] |
| 8.61E-80 | BIRC5 | baculoviral IAP repeat containing 5 [Source:HGNC Symbol;Acc:HGNC:593] |
| 1.55E-79 | KIF14 | kinesin family member 14 [Source:HGNC Symbol;Acc:HGNC:19181] |
| 1.91E-79 | DTL | denticleless E3 ubiquitin protein ligase homolog (Drosophila) [Source:HGNC Symbol;Acc:HGNC:30288] |
| 3.02E-79 | TOP2A | topoisomerase (DNA) II alpha 170kDa [Source:HGNC Symbol;Acc:HGNC:11989] |
| 6.67E-79 | KIF2C | kinesin family member 2C [Source:HGNC Symbol;Acc:HGNC:6393] |
| 1.49E-78 | NUF2 | NUF2, NDC80 kinetochore complex component [Source:HGNC Symbol;Acc:HGNC:14621] |
| 2.52E-78 | CENPM | centromere protein M [Source:HGNC Symbol;Acc:HGNC:18352] |
| 1.20E-77 | GINS2 | GINS complex subunit 2 (Psf2 homolog) [Source:HGNC Symbol;Acc:HGNC:24575] |
| 3.52E-77 | CENPU | centromere protein U [Source:HGNC Symbol;Acc:HGNC:21348] |
| 7.30E-77 | PRC1 | protein regulator of cytokinesis 1 [Source:HGNC Symbol;Acc:HGNC:9341] |
| 2.21E-76 | CDC45 | cell division cycle 45 [Source:HGNC Symbol;Acc:HGNC:1739] |
| 2.81E-76 | BUB1B | BUB1 mitotic checkpoint serine/threonine kinase B [Source:HGNC Symbol;Acc:HGNC:1149] |
| 3.06E-76 | CCNB2 | cyclin B2 [Source:HGNC Symbol;Acc:HGNC:1580] |
| 4.49E-76 | GINS1 | GINS complex subunit 1 (Psf1 homolog) [Source:HGNC Symbol;Acc:HGNC:28980] |
| 8.08E-76 | TK1 | thymidine kinase 1, soluble [Source:HGNC Symbol;Acc:HGNC:11830] |
| 1.08E-75 | NUSAP1 | nucleolar and spindle associated protein 1 [Source:HGNC Symbol;Acc:HGNC:18538] |
| 1.40E-75 | AURKB | aurora kinase B [Source:HGNC Symbol;Acc:HGNC:11390] |
| 1.48E-75 | NDC80 | NDC80 kinetochore complex component [Source:HGNC Symbol;Acc:HGNC:16909] |
| 2.83E-75 | RRM1 | ribonucleotide reductase M1 [Source:HGNC Symbol;Acc:HGNC:10451] |
| 5.60E-75 | DTL | denticleless E3 ubiquitin protein ligase homolog (Drosophila) [Source:HGNC Symbol;Acc:HGNC:30288] |
| 1.00E-74 | CENPF | centromere protein F, 350/400kDa [Source:HGNC Symbol;Acc:HGNC:1857] |
| 1.02E-74 | FANCI | Fanconi anemia, complementation group I [Source:HGNC Symbol;Acc:HGNC:25568] |
| 1.13E-74 | UHRF1 | ubiquitin-like with PHD and ring finger domains 1 [Source:HGNC Symbol;Acc:HGNC:12556] |
| 1.20E-74 | KIF15 | kinesin family member 15 [Source:HGNC Symbol;Acc:HGNC:17273] |
| 3.31E-74 | OIP5 | Opa interacting protein 5 [Source:HGNC Symbol;Acc:HGNC:20300] |
| 5.44E-74 | DHFR | dihydrofolate reductase [Source:HGNC Symbol;Acc:HGNC:2861] |
| 6.04E-74 | MCM10 | minichromosome maintenance complex component 10 [Source:HGNC Symbol;Acc:HGNC:18043] |
| 1.52E-73 | MKI67 | marker of proliferation Ki-67 [Source:HGNC Symbol;Acc:HGNC:7107] |
| 2.67E-73 | MCM10 | minichromosome maintenance complex component 10 [Source:HGNC Symbol;Acc:HGNC:18043] |
| 3.80E-73 | KIF11 | kinesin family member 11 [Source:HGNC Symbol;Acc:HGNC:6388] |
| 5.86E-73 | FOXM1 | forkhead box M1 [Source:HGNC Symbol;Acc:HGNC:3818] |
| 9.16E-73 | FANCI | Fanconi anemia, complementation group I [Source:HGNC Symbol;Acc:HGNC:25568] |
| 2.87E-72 | FEN1 | flap structure-specific endonuclease 1 [Source:HGNC Symbol;Acc:HGNC:3650] |
| 4.16E-72 | CDK1 | cyclin-dependent kinase 1 [Source:HGNC Symbol;Acc:HGNC:1722] |
| 8.18E-72 | KIFC1 | kinesin family member C1 [Source:HGNC Symbol;Acc:HGNC:6389] |
| 9.61E-72 | NCAPG2 | non-SMC condensin II complex, subunit G2 [Source:HGNC Symbol;Acc:HGNC:21904] |
| 2.60E-71 | CDCA3 | cell division cycle associated 3 [Source:HGNC Symbol;Acc:HGNC:14624] |
| 5.25E-71 | DHFR | dihydrofolate reductase [Source:HGNC Symbol;Acc:HGNC:2861] |
| 9.41E-71 | CDCA5 | cell division cycle associated 5 [Source:HGNC Symbol;Acc:HGNC:14626] |
| 1.20E-70 | EXO1 | exonuclease 1 [Source:HGNC Symbol;Acc:HGNC:3511] |
| 1.62E-70 | TTK | TTK protein kinase [Source:HGNC Symbol;Acc:HGNC:12401] |
| 1.83E-70 | RAD54L | RAD54-like (S. cerevisiae) [Source:HGNC Symbol;Acc:HGNC:9826] |
| 1.91E-70 | HELLS | helicase, lymphoid-specific [Source:HGNC Symbol;Acc:HGNC:4861] |
| 1.02E-69 | KIF23 | kinesin family member 23 [Source:HGNC Symbol;Acc:HGNC:6392] |
| 1.13E-69 | ATAD2 | ATPase family, AAA domain containing 2 [Source:HGNC Symbol;Acc:HGNC:30123] |
| 1.55E-69 | NCAPH | non-SMC condensin I complex, subunit H [Source:HGNC Symbol;Acc:HGNC:1112] |
| 2.37E-69 | CDC6 | cell division cycle 6 [Source:HGNC Symbol;Acc:HGNC:1744] |
| 2.87E-69 | KNTC1 | kinetochore associated 1 [Source:HGNC Symbol;Acc:HGNC:17255] |
| 5.22E-69 | STIL | SCL/TAL1 interrupting locus [Source:HGNC Symbol;Acc:HGNC:10879] |
| 5.69E-69 | MND1 | meiotic nuclear divisions 1 homolog (S. cerevisiae) [Source:HGNC Symbol;Acc:HGNC:24839] |
| 5.85E-69 | TOP2A | topoisomerase (DNA) II alpha 170kDa [Source:HGNC Symbol;Acc:HGNC:11989] |
| 5.90E-69 | MCM4 | minichromosome maintenance complex component 4 [Source:HGNC Symbol;Acc:HGNC:6947] |
| 7.76E-69 | HJURP | Holliday junction recognition protein [Source:HGNC Symbol;Acc:HGNC:25444] |
| 8.31E-69 | ZWINT | ZW10 interacting kinetochore protein [Source:HGNC Symbol;Acc:HGNC:13195] |
| 1.28E-68 | KIF2C | kinesin family member 2C [Source:HGNC Symbol;Acc:HGNC:6393] |
| 1.53E-68 | NUSAP1 | nucleolar and spindle associated protein 1 [Source:HGNC Symbol;Acc:HGNC:18538] |
| 2.15E-68 | TK1 | thymidine kinase 1, soluble [Source:HGNC Symbol;Acc:HGNC:11830] |
| 2.18E-68 | ASPM | asp (abnormal spindle) homolog, microcephaly associated (Drosophila) [Source:HGNC Symbol;Acc:HGNC:19048] |
| 2.21E-68 | RAD51 | RAD51 recombinase [Source:HGNC Symbol;Acc:HGNC:9817] |
| 2.82E-68 | CDC6 | cell division cycle 6 [Source:HGNC Symbol;Acc:HGNC:1744] |
| 4.06E-68 | KIF18B | kinesin family member 18B [Source:HGNC Symbol;Acc:HGNC:27102] |
| 1.01E-67 | SGOL1 | shugoshin-like 1 (S. pombe) [Source:HGNC Symbol;Acc:HGNC:25088] |
| 1.46E-67 | PLK4 | polo-like kinase 4 [Source:HGNC Symbol;Acc:HGNC:11397] |
| 2.06E-67 | CCNA2 | cyclin A2 [Source:HGNC Symbol;Acc:HGNC:1578] |
| 2.40E-67 | CASC5 | cancer susceptibility candidate 5 [Source:HGNC Symbol;Acc:HGNC:24054] |
| 3.08E-67 | TACC3 | transforming, acidic coiled-coil containing protein 3 [Source:HGNC Symbol;Acc:HGNC:11524] |
| 3.20E-67 | MCM10 | minichromosome maintenance complex component 10 [Source:HGNC Symbol;Acc:HGNC:18043] |
| 3.41E-67 | KIF20A | kinesin family member 20A [Source:HGNC Symbol;Acc:HGNC:9787] |
| 3.87E-67 | MKI67 | marker of proliferation Ki-67 [Source:HGNC Symbol;Acc:HGNC:7107] |
| 4.12E-67 | FBXO5 | F-box protein 5 [Source:HGNC Symbol;Acc:HGNC:13584] |
| 6.94E-67 | RRM2 | ribonucleotide reductase M2 [Source:HGNC Symbol;Acc:HGNC:10452] |
| 1.15E-66 | ATAD2 | ATPase family, AAA domain containing 2 [Source:HGNC Symbol;Acc:HGNC:30123] |
| 1.63E-66 | MCM2 | minichromosome maintenance complex component 2 [Source:HGNC Symbol;Acc:HGNC:6944] |
| 1.71E-66 | CDK1 | cyclin-dependent kinase 1 [Source:HGNC Symbol;Acc:HGNC:1722] |
| 2.77E-66 | MKI67 | marker of proliferation Ki-67 [Source:HGNC Symbol;Acc:HGNC:7107] |
| 3.27E-66 | BRCA1 | breast cancer 1, early onset [Source:HGNC Symbol;Acc:HGNC:1100] |
| 3.67E-66 | MCM6 | minichromosome maintenance complex component 6 [Source:HGNC Symbol;Acc:HGNC:6949] |
| 5.65E-66 | HELLS | helicase, lymphoid-specific [Source:HGNC Symbol;Acc:HGNC:4861] |
| 6.08E-66 | RACGAP1 | Rac GTPase activating protein 1 [Source:HGNC Symbol;Acc:HGNC:9804] |
| 6.68E-66 | DHFR | dihydrofolate reductase [Source:HGNC Symbol;Acc:HGNC:2861] |
| 7.97E-66 | MCM8 | minichromosome maintenance complex component 8 [Source:HGNC Symbol;Acc:HGNC:16147] |
| 1.13E-65 | PRR11 | proline rich 11 [Source:HGNC Symbol;Acc:HGNC:25619] |
| 1.26E-65 | CCNB1 | cyclin B1 [Source:HGNC Symbol;Acc:HGNC:1579] |
| 3.85E-65 | CENPK | centromere protein K [Source:HGNC Symbol;Acc:HGNC:29479] |
| 4.49E-65 | CHAF1A | chromatin assembly factor 1, subunit A (p150) [Source:HGNC Symbol;Acc:HGNC:1910] |
| 4.49E-65 | CDK1 | cyclin-dependent kinase 1 [Source:HGNC Symbol;Acc:HGNC:1722] |
| 5.42E-65 | TYMS | thymidylate synthetase [Source:HGNC Symbol;Acc:HGNC:12441] |
| 6.11E-65 | NEK2 | NIMA-related kinase 2 [Source:HGNC Symbol;Acc:HGNC:7745] |
| 6.49E-65 | BUB1 | BUB1 mitotic checkpoint serine/threonine kinase [Source:HGNC Symbol;Acc:HGNC:1148] |
| 7.28E-65 | SHCBP1 | SHC SH2-domain binding protein 1 [Source:HGNC Symbol;Acc:HGNC:29547] |
| 7.52E-65 | PLK4 | polo-like kinase 4 [Source:HGNC Symbol;Acc:HGNC:11397] |
| 7.52E-65 | SPC25 | SPC25, NDC80 kinetochore complex component [Source:HGNC Symbol;Acc:HGNC:24031] |
| 1.09E-64 | TIMELESS | timeless circadian clock [Source:HGNC Symbol;Acc:HGNC:11813] |
| 1.28E-64 | MELK | maternal embryonic leucine zipper kinase [Source:HGNC Symbol;Acc:HGNC:16870] |
| 1.68E-64 | HMMR | hyaluronan-mediated motility receptor (RHAMM) [Source:HGNC Symbol;Acc:HGNC:5012] |
| 1.77E-64 | CDC20 | cell division cycle 20 [Source:HGNC Symbol;Acc:HGNC:1723] |
| 2.37E-64 | HELLS | helicase, lymphoid-specific [Source:HGNC Symbol;Acc:HGNC:4861] |
| 2.94E-64 | DSN1 | DSN1, MIS12 kinetochore complex component [Source:HGNC Symbol;Acc:HGNC:16165] |
| 3.05E-64 | BIRC5 | baculoviral IAP repeat containing 5 [Source:HGNC Symbol;Acc:HGNC:593] |
| 3.12E-64 | AURKA | aurora kinase A [Source:HGNC Symbol;Acc:HGNC:11393] |
| 3.29E-64 | GINS4 | GINS complex subunit 4 (Sld5 homolog) [Source:HGNC Symbol;Acc:HGNC:28226] |
| 3.60E-64 | DLGAP5 | discs, large (Drosophila) homolog-associated protein 5 [Source:HGNC Symbol;Acc:HGNC:16864] |
| 3.81E-64 | PSMC3IP | PSMC3 interacting protein [Source:HGNC Symbol;Acc:HGNC:17928] |
| 5.29E-64 | ATAD2 | ATPase family, AAA domain containing 2 [Source:HGNC Symbol;Acc:HGNC:30123] |
| 6.32E-64 | RAD51AP1 | RAD51 associated protein 1 [Source:HGNC Symbol;Acc:HGNC:16956] |
| 8.72E-64 | SPAG5 | sperm associated antigen 5 [Source:HGNC Symbol;Acc:HGNC:13452] |
| 1.21E-63 | RRM2 | ribonucleotide reductase M2 [Source:HGNC Symbol;Acc:HGNC:10452] |
| 1.38E-63 | KIF4A | kinesin family member 4A [Source:HGNC Symbol;Acc:HGNC:13339] |
| 1.51E-63 | WDR76 | WD repeat domain 76 [Source:HGNC Symbol;Acc:HGNC:25773] |
| 1.99E-63 | ORC1 | origin recognition complex, subunit 1 [Source:HGNC Symbol;Acc:HGNC:8487] |
| 2.29E-63 | TICRR | TOPBP1-interacting checkpoint and replication regulator [Source:HGNC Symbol;Acc:HGNC:28704] |
| 3.05E-63 | MCM4 | minichromosome maintenance complex component 4 [Source:HGNC Symbol;Acc:HGNC:6947] |
| 7.94E-63 | MKI67 | marker of proliferation Ki-67 [Source:HGNC Symbol;Acc:HGNC:7107] |
| 1.04E-62 | FANCD2 | Fanconi anemia, complementation group D2 [Source:HGNC Symbol;Acc:HGNC:3585] |
| 1.10E-62 | UBE2C | ubiquitin-conjugating enzyme E2C [Source:HGNC Symbol;Acc:HGNC:15937] |
| 1.69E-62 | SPC24 | SPC24, NDC80 kinetochore complex component [Source:HGNC Symbol;Acc:HGNC:26913] |
| 2.00E-62 | UBE2T | ubiquitin-conjugating enzyme E2T [Source:HGNC Symbol;Acc:HGNC:25009] |
| 2.98E-62 | DNA2 | DNA replication helicase/nuclease 2 [Source:HGNC Symbol;Acc:HGNC:2939] |
| 8.14E-62 | BRIP1 | BRCA1 interacting protein C-terminal helicase 1 [Source:HGNC Symbol;Acc:HGNC:20473] |
| 1.15E-61 | KIF14 | kinesin family member 14 [Source:HGNC Symbol;Acc:HGNC:19181] |
| 1.63E-61 | DSCC1 | DNA replication and sister chromatid cohesion 1 [Source:HGNC Symbol;Acc:HGNC:24453] |
| 1.99E-61 | TYMS | thymidylate synthetase [Source:HGNC Symbol;Acc:HGNC:12441] |
| 3.03E-61 | TPX2 | TPX2, microtubule-associated [Source:HGNC Symbol;Acc:HGNC:1249] |
| 3.14E-61 | CDCA3 | cell division cycle associated 3 [Source:HGNC Symbol;Acc:HGNC:14624] |
| 3.15E-61 | BIRC5 | baculoviral IAP repeat containing 5 [Source:HGNC Symbol;Acc:HGNC:593] |
| 3.50E-61 | MCM5 | minichromosome maintenance complex component 5 [Source:HGNC Symbol;Acc:HGNC:6948] |
| 3.94E-61 | PTTG1 | pituitary tumor-transforming 1 [Source:HGNC Symbol;Acc:HGNC:9690] |
| 4.16E-61 | BRCA2 | breast cancer 2, early onset [Source:HGNC Symbol;Acc:HGNC:1101] |
| 5.77E-61 | CCNB1 | cyclin B1 [Source:HGNC Symbol;Acc:HGNC:1579] |
| 1.05E-60 | CDCA2 | cell division cycle associated 2 [Source:HGNC Symbol;Acc:HGNC:14623] |
| 1.06E-60 | CENPL | centromere protein L [Source:HGNC Symbol;Acc:HGNC:17879] |
| 1.41E-60 | ESPL1 | extra spindle pole bodies homolog 1 (S. cerevisiae) [Source:HGNC Symbol;Acc:HGNC:16856] |
| 1.83E-60 | CDC25C | cell division cycle 25C [Source:HGNC Symbol;Acc:HGNC:1727] |
| 2.63E-60 | ASF1B | anti-silencing function 1B histone chaperone [Source:HGNC Symbol;Acc:HGNC:20996] |
| 2.93E-60 | CENPE | centromere protein E, 312kDa [Source:HGNC Symbol;Acc:HGNC:1856] |
| 3.29E-60 | LMNB1 | lamin B1 [Source:HGNC Symbol;Acc:HGNC:6637] |
| 3.93E-60 | DEPDC1B | DEP domain containing 1B [Source:HGNC Symbol;Acc:HGNC:24902] |
| 5.00E-60 | WHSC1 | Wolf-Hirschhorn syndrome candidate 1 [Source:HGNC Symbol;Acc:HGNC:12766] |
| 6.19E-60 | CDCA8 | cell division cycle associated 8 [Source:HGNC Symbol;Acc:HGNC:14629] |
| 1.59E-59 | PARPBP | PARP1 binding protein [Source:HGNC Symbol;Acc:HGNC:26074] |
| 2.24E-59 | TROAP | trophinin associated protein [Source:HGNC Symbol;Acc:HGNC:12327] |
| 2.52E-59 | ESPL1 | extra spindle pole bodies homolog 1 (S. cerevisiae) [Source:HGNC Symbol;Acc:HGNC:16856] |
| 2.92E-59 | BLM | Bloom syndrome, RecQ helicase-like [Source:HGNC Symbol;Acc:HGNC:1058] |
| 3.21E-59 | ZNF367 | zinc finger protein 367 [Source:HGNC Symbol;Acc:HGNC:18320] |
| 3.36E-59 | CENPI | centromere protein I [Source:HGNC Symbol;Acc:HGNC:3968] |
| 4.98E-59 | PBK | PDZ binding kinase [Source:HGNC Symbol;Acc:HGNC:18282] |
| 5.11E-59 | ARHGAP11A | Rho GTPase activating protein 11A [Source:HGNC Symbol;Acc:HGNC:15783] |
| 5.60E-59 | DTYMK | deoxythymidylate kinase (thymidylate kinase) [Source:HGNC Symbol;Acc:HGNC:3061] |
| 5.97E-59 | FBXO5 | F-box protein 5 [Source:HGNC Symbol;Acc:HGNC:13584] |
| 6.10E-59 | BARD1 | BRCA1 associated RING domain 1 [Source:HGNC Symbol;Acc:HGNC:952] |
| 6.24E-59 | CENPF | centromere protein F, 350/400kDa [Source:HGNC Symbol;Acc:HGNC:1857] |
| 6.50E-59 | DEPDC1 | DEP domain containing 1 [Source:HGNC Symbol;Acc:HGNC:22949] |
| 8.38E-59 | EZH2 | enhancer of zeste 2 polycomb repressive complex 2 subunit [Source:HGNC Symbol;Acc:HGNC:3527] |
| 9.70E-59 | NCAPD2 | non-SMC condensin I complex, subunit D2 [Source:HGNC Symbol;Acc:HGNC:24305] |
| 1.14E-58 | BRCA1 | breast cancer 1, early onset [Source:HGNC Symbol;Acc:HGNC:1100] |
| 1.21E-58 | ZWILCH | zwilch kinetochore protein [Source:HGNC Symbol;Acc:HGNC:25468] |
| 1.28E-58 | MCM5 | minichromosome maintenance complex component 5 [Source:HGNC Symbol;Acc:HGNC:6948] |
| 1.29E-58 | POLE2 | polymerase (DNA directed), epsilon 2, accessory subunit [Source:HGNC Symbol;Acc:HGNC:9178] |
| 1.41E-58 | CHAF1A | chromatin assembly factor 1, subunit A (p150) [Source:HGNC Symbol;Acc:HGNC:1910] |
| 2.27E-58 | CHAF1A | chromatin assembly factor 1, subunit A (p150) [Source:HGNC Symbol;Acc:HGNC:1910] |
| 3.06E-58 | RFC4 | replication factor C (activator 1) 4, 37kDa [Source:HGNC Symbol;Acc:HGNC:9972] |
| 3.08E-58 | CKAP2L | cytoskeleton associated protein 2-like [Source:HGNC Symbol;Acc:HGNC:26877] |
| 3.81E-58 | ATAD2 | ATPase family, AAA domain containing 2 [Source:HGNC Symbol;Acc:HGNC:30123] |
| 8.79E-58 | RFC2 | replication factor C (activator 1) 2, 40kDa [Source:HGNC Symbol;Acc:HGNC:9970] |
| 1.21E-57 | DTYMK | deoxythymidylate kinase (thymidylate kinase) [Source:HGNC Symbol;Acc:HGNC:3061] |
| 1.51E-57 | CENPN | centromere protein N [Source:HGNC Symbol;Acc:HGNC:30873] |
| 1.54E-57 | CENPU | centromere protein U [Source:HGNC Symbol;Acc:HGNC:21348] |
| 1.63E-57 | TRMU | tRNA 5-methylaminomethyl-2-thiouridylate methyltransferase [Source:HGNC Symbol;Acc:HGNC:25481] |
| 2.16E-57 | GTSE1 | G-2 and S-phase expressed 1 [Source:HGNC Symbol;Acc:HGNC:13698] |
| 2.40E-57 | TRIP13 | thyroid hormone receptor interactor 13 [Source:HGNC Symbol;Acc:HGNC:12307] |
| 2.98E-57 | SGOL2 | shugoshin-like 2 (S. pombe) [Source:HGNC Symbol;Acc:HGNC:30812] |
| 4.78E-57 | RP11-303E16.2 | N/A |
| 6.28E-57 | TMPO | thymopoietin [Source:HGNC Symbol;Acc:HGNC:11875] |
| 7.11E-57 | GTSE1 | G-2 and S-phase expressed 1 [Source:HGNC Symbol;Acc:HGNC:13698] |
| 7.65E-57 | CDC25A | cell division cycle 25A [Source:HGNC Symbol;Acc:HGNC:1725] |
| 9.82E-57 | CCNA2 | cyclin A2 [Source:HGNC Symbol;Acc:HGNC:1578] |
| 1.12E-56 | MCM3 | minichromosome maintenance complex component 3 [Source:HGNC Symbol;Acc:HGNC:6945] |
| 1.20E-56 | AURKA | aurora kinase A [Source:HGNC Symbol;Acc:HGNC:11393] |
| 1.36E-56 | SKA3 | spindle and kinetochore associated complex subunit 3 [Source:HGNC Symbol;Acc:HGNC:20262] |
| 2.01E-56 | WDHD1 | WD repeat and HMG-box DNA binding protein 1 [Source:HGNC Symbol;Acc:HGNC:23170] |
| 2.36E-56 | CCNE2 | cyclin E2 [Source:HGNC Symbol;Acc:HGNC:1590] |
| 2.70E-56 | HMMR | hyaluronan-mediated motility receptor (RHAMM) [Source:HGNC Symbol;Acc:HGNC:5012] |
| 3.16E-56 | RFC3 | replication factor C (activator 1) 3, 38kDa [Source:HGNC Symbol;Acc:HGNC:9971] |
| 3.34E-56 | TMPO | thymopoietin [Source:HGNC Symbol;Acc:HGNC:11875] |
| 5.28E-56 | CEP55 | centrosomal protein 55kDa [Source:HGNC Symbol;Acc:HGNC:1161] |
| 5.99E-56 | CENPA | centromere protein A [Source:HGNC Symbol;Acc:HGNC:1851] |
| 6.00E-56 | CASC5 | cancer susceptibility candidate 5 [Source:HGNC Symbol;Acc:HGNC:24054] |
| 6.65E-56 | PRIM1 | primase, DNA, polypeptide 1 (49kDa) [Source:HGNC Symbol;Acc:HGNC:9369] |
| 6.76E-56 | RNASEH2A | ribonuclease H2, subunit A [Source:HGNC Symbol;Acc:HGNC:18518] |
| 1.11E-55 | WHSC1 | Wolf-Hirschhorn syndrome candidate 1 [Source:HGNC Symbol;Acc:HGNC:12766] |
| 1.55E-55 | MASTL | microtubule associated serine/threonine kinase-like [Source:HGNC Symbol;Acc:HGNC:19042] |
| 1.63E-55 | POLQ | polymerase (DNA directed), theta [Source:HGNC Symbol;Acc:HGNC:9186] |
| 2.22E-55 | ECT2 | epithelial cell transforming 2 [Source:HGNC Symbol;Acc:HGNC:3155] |
| 2.66E-55 | KNSTRN | kinetochore-localized astrin/SPAG5 binding protein [Source:HGNC Symbol;Acc:HGNC:30767] |
| 2.74E-55 | MTFR2 | mitochondrial fission regulator 2 [Source:HGNC Symbol;Acc:HGNC:21115] |
| 4.40E-55 | CHAF1B | chromatin assembly factor 1, subunit B (p60) [Source:HGNC Symbol;Acc:HGNC:1911] |
| 6.00E-55 | SMC4 | structural maintenance of chromosomes 4 [Source:HGNC Symbol;Acc:HGNC:14013] |
| 8.74E-55 | TCF19 | transcription factor 19 [Source:HGNC Symbol;Acc:HGNC:11629] |
| 1.10E-54 | PLK1 | polo-like kinase 1 [Source:HGNC Symbol;Acc:HGNC:9077] |
| 1.12E-54 | DEPDC1 | DEP domain containing 1 [Source:HGNC Symbol;Acc:HGNC:22949] |
| 1.16E-54 | FAM64A | family with sequence similarity 64, member A [Source:HGNC Symbol;Acc:HGNC:25483] |
| 1.39E-54 | CCNF | cyclin F [Source:HGNC Symbol;Acc:HGNC:1591] |
| 1.39E-54 | RFC2 | replication factor C (activator 1) 2, 40kDa [Source:HGNC Symbol;Acc:HGNC:9970] |
| 2.54E-54 | FEN1 | flap structure-specific endonuclease 1 [Source:HGNC Symbol;Acc:HGNC:3650] |
| 2.76E-54 | RFC5 | replication factor C (activator 1) 5, 36.5kDa [Source:HGNC Symbol;Acc:HGNC:9973] |
| 3.10E-54 | MYBL2 | v-myb avian myeloblastosis viral oncogene homolog-like 2 [Source:HGNC Symbol;Acc:HGNC:7548] |
| 3.35E-54 | E2F8 | E2F transcription factor 8 [Source:HGNC Symbol;Acc:HGNC:24727] |
| 4.94E-54 | CENPH | centromere protein H [Source:HGNC Symbol;Acc:HGNC:17268] |
| 4.94E-54 | ESCO2 | establishment of sister chromatid cohesion N-acetyltransferase 2 [Source:HGNC Symbol;Acc:HGNC:27230] |
| 7.11E-54 | MAD2L1 | MAD2 mitotic arrest deficient-like 1 (yeast) [Source:HGNC Symbol;Acc:HGNC:6763] |
| 1.17E-53 | CDT1 | chromatin licensing and DNA replication factor 1 [Source:HGNC Symbol;Acc:HGNC:24576] |
| 1.25E-53 | SKP2 | S-phase kinase-associated protein 2, E3 ubiquitin protein ligase [Source:HGNC Symbol;Acc:HGNC:10901] |
| 1.30E-53 | SGOL2 | shugoshin-like 2 (S. pombe) [Source:HGNC Symbol;Acc:HGNC:30812] |
| 1.41E-53 | CHEK1 | checkpoint kinase 1 [Source:HGNC Symbol;Acc:HGNC:1925] |
| 3.12E-53 | NASP | nuclear autoantigenic sperm protein (histone-binding) [Source:HGNC Symbol;Acc:HGNC:7644] |
| 3.93E-53 | CDC25A | cell division cycle 25A [Source:HGNC Symbol;Acc:HGNC:1725] |
| 5.45E-53 | NEIL3 | nei endonuclease VIII-like 3 (E. coli) [Source:HGNC Symbol;Acc:HGNC:24573] |
| 7.92E-53 | MAD2L1 | MAD2 mitotic arrest deficient-like 1 (yeast) [Source:HGNC Symbol;Acc:HGNC:6763] |
| 8.56E-53 | MCM7 | minichromosome maintenance complex component 7 [Source:HGNC Symbol;Acc:HGNC:6950] |
| 8.62E-53 | RECQL4 | RecQ protein-like 4 [Source:HGNC Symbol;Acc:HGNC:9949] |
| 9.50E-53 | ZWILCH | zwilch kinetochore protein [Source:HGNC Symbol;Acc:HGNC:25468] |
| 1.15E-52 | ASPM | asp (abnormal spindle) homolog, microcephaly associated (Drosophila) [Source:HGNC Symbol;Acc:HGNC:19048] |
| 1.21E-52 | SKA1 | spindle and kinetochore associated complex subunit 1 [Source:HGNC Symbol;Acc:HGNC:28109] |
| 1.24E-52 | POLE | polymerase (DNA directed), epsilon, catalytic subunit [Source:HGNC Symbol;Acc:HGNC:9177] |
| 1.91E-52 | CDKN3 | cyclin-dependent kinase inhibitor 3 [Source:HGNC Symbol;Acc:HGNC:1791] |
| 3.78E-52 | SNRPA | small nuclear ribonucleoprotein polypeptide A [Source:HGNC Symbol;Acc:HGNC:11151] |
| 6.24E-52 | HMGB2 | high mobility group box 2 [Source:HGNC Symbol;Acc:HGNC:5000] |
| 8.46E-52 | CDCA7 | cell division cycle associated 7 [Source:HGNC Symbol;Acc:HGNC:14628] |
| 9.75E-52 | DDIAS | DNA damage-induced apoptosis suppressor [Source:HGNC Symbol;Acc:HGNC:26351] |
| 1.16E-51 | CHEK1 | checkpoint kinase 1 [Source:HGNC Symbol;Acc:HGNC:1925] |
| 1.26E-51 | DEPDC1 | DEP domain containing 1 [Source:HGNC Symbol;Acc:HGNC:22949] |
| 1.43E-51 | PCNA | proliferating cell nuclear antigen [Source:HGNC Symbol;Acc:HGNC:8729] |
| 2.50E-51 | LIG1 | ligase I, DNA, ATP-dependent [Source:HGNC Symbol;Acc:HGNC:6598] |
| 2.98E-51 | ANLN | anillin, actin binding protein [Source:HGNC Symbol;Acc:HGNC:14082] |
| 2.99E-51 | TIPIN | TIMELESS interacting protein [Source:HGNC Symbol;Acc:HGNC:30750] |
| 3.24E-51 | PIF1 | PIF1 5'-to-3' DNA helicase [Source:HGNC Symbol;Acc:HGNC:26220] |
| 5.08E-51 | ERCC6L | excision repair cross-complementation group 6-like [Source:HGNC Symbol;Acc:HGNC:20794] |
| 7.07E-51 | CDKN3 | cyclin-dependent kinase inhibitor 3 [Source:HGNC Symbol;Acc:HGNC:1791] |
| 7.20E-51 | MIS18A | MIS18 kinetochore protein A [Source:HGNC Symbol;Acc:HGNC:1286] |
| 7.48E-51 | CDC7 | cell division cycle 7 [Source:HGNC Symbol;Acc:HGNC:1745] |
| 1.27E-50 | TROAP | trophinin associated protein [Source:HGNC Symbol;Acc:HGNC:12327] |
| 2.33E-50 | IQGAP3 | IQ motif containing GTPase activating protein 3 [Source:HGNC Symbol;Acc:HGNC:20669] |
| 2.58E-50 | STMN1 | stathmin 1 [Source:HGNC Symbol;Acc:HGNC:6510] |
| 3.01E-50 | RMI2 | RecQ mediated genome instability 2 [Source:HGNC Symbol;Acc:HGNC:28349] |
| 3.48E-50 | ATAD5 | ATPase family, AAA domain containing 5 [Source:HGNC Symbol;Acc:HGNC:25752] |
| 4.28E-50 | POLA2 | polymerase (DNA directed), alpha 2, accessory subunit [Source:HGNC Symbol;Acc:HGNC:30073] |
| 5.33E-50 | KIAA1524 | KIAA1524 [Source:HGNC Symbol;Acc:HGNC:29302] |
| 5.42E-50 | RFC5 | replication factor C (activator 1) 5, 36.5kDa [Source:HGNC Symbol;Acc:HGNC:9973] |
| 5.45E-50 | DBF4 | DBF4 zinc finger [Source:HGNC Symbol;Acc:HGNC:17364] |
| 8.46E-50 | ASPM | asp (abnormal spindle) homolog, microcephaly associated (Drosophila) [Source:HGNC Symbol;Acc:HGNC:19048] |
| 1.18E-49 | DUT | deoxyuridine triphosphatase [Source:HGNC Symbol;Acc:HGNC:3078] |
| 1.48E-49 | SUV39H2 | suppressor of variegation 3-9 homolog 2 (Drosophila) [Source:HGNC Symbol;Acc:HGNC:17287] |
| 2.60E-49 | WDR76 | WD repeat domain 76 [Source:HGNC Symbol;Acc:HGNC:25773] |
| 4.73E-49 | RAD54B | RAD54 homolog B (S. cerevisiae) [Source:HGNC Symbol;Acc:HGNC:17228] |
| 5.17E-49 | KIF18A | kinesin family member 18A [Source:HGNC Symbol;Acc:HGNC:29441] |
| 5.79E-49 | TMPO-AS1 | TMPO antisense RNA 1 [Source:HGNC Symbol;Acc:HGNC:44158] |
| 7.13E-49 | GMNN | geminin, DNA replication inhibitor [Source:HGNC Symbol;Acc:HGNC:17493] |
| 8.22E-49 | RBL1 | retinoblastoma-like 1 [Source:HGNC Symbol;Acc:HGNC:9893] |
| 1.16E-48 | FANCA | Fanconi anemia, complementation group A [Source:HGNC Symbol;Acc:HGNC:3582] |
| 1.19E-48 | GSG2 | germ cell associated 2 (haspin) [Source:HGNC Symbol;Acc:HGNC:19682] |
| 1.20E-48 | SMC4 | structural maintenance of chromosomes 4 [Source:HGNC Symbol;Acc:HGNC:14013] |
| 1.68E-48 | USP1 | ubiquitin specific peptidase 1 [Source:HGNC Symbol;Acc:HGNC:12607] |
| 3.58E-48 | DUT | deoxyuridine triphosphatase [Source:HGNC Symbol;Acc:HGNC:3078] |
| 5.50E-48 | CENPO | centromere protein O [Source:HGNC Symbol;Acc:HGNC:28152] |
| 5.85E-48 | E2F1 | E2F transcription factor 1 [Source:HGNC Symbol;Acc:HGNC:3113] |
| 5.93E-48 | CDT1 | chromatin licensing and DNA replication factor 1 [Source:HGNC Symbol;Acc:HGNC:24576] |
| 9.38E-48 | WDHD1 | WD repeat and HMG-box DNA binding protein 1 [Source:HGNC Symbol;Acc:HGNC:23170] |
| 1.55E-47 | LRR1 | leucine rich repeat protein 1 [Source:HGNC Symbol;Acc:HGNC:19742] |
| 2.02E-47 | GINS3 | GINS complex subunit 3 (Psf3 homolog) [Source:HGNC Symbol;Acc:HGNC:25851] |
| 2.69E-47 | HAUS8 | HAUS augmin-like complex, subunit 8 [Source:HGNC Symbol;Acc:HGNC:30532] |
| 2.83E-47 | PSRC1 | proline/serine-rich coiled-coil 1 [Source:HGNC Symbol;Acc:HGNC:24472] |
| 3.79E-47 | CDCA2 | cell division cycle associated 2 [Source:HGNC Symbol;Acc:HGNC:14623] |
| 3.94E-47 | C4ORF46 | chromosome 4 open reading frame 46 [Source:HGNC Symbol;Acc:HGNC:27320] |
| 6.17E-47 | CENPL | centromere protein L [Source:HGNC Symbol;Acc:HGNC:17879] |
| 7.20E-47 | MCM4 | minichromosome maintenance complex component 4 [Source:HGNC Symbol;Acc:HGNC:6947] |
| 1.04E-46 | CENPW | centromere protein W [Source:HGNC Symbol;Acc:HGNC:21488] |
| 1.16E-46 | BRIP1 | BRCA1 interacting protein C-terminal helicase 1 [Source:HGNC Symbol;Acc:HGNC:20473] |
| 1.20E-46 | PKMYT1 | protein kinase, membrane associated tyrosine/threonine 1 [Source:HGNC Symbol;Acc:HGNC:29650] |
| 1.21E-46 | CSE1L | CSE1 chromosome segregation 1-like (yeast) [Source:HGNC Symbol;Acc:HGNC:2431] |
| 1.74E-46 | SAPCD2 | suppressor APC domain containing 2 [Source:HGNC Symbol;Acc:HGNC:28055] |
| 2.06E-46 | H2AFZ | H2A histone family, member Z [Source:HGNC Symbol;Acc:HGNC:4741] |
| 2.47E-46 | TMPO | thymopoietin [Source:HGNC Symbol;Acc:HGNC:11875] |
| 2.59E-46 | RAD1 | RAD1 checkpoint DNA exonuclease [Source:HGNC Symbol;Acc:HGNC:9806] |
| 2.86E-46 | E2F2 | E2F transcription factor 2 [Source:HGNC Symbol;Acc:HGNC:3114] |
| 3.18E-46 | LMNB2 | lamin B2 [Source:HGNC Symbol;Acc:HGNC:6638] |
| 3.45E-46 | MCM4 | minichromosome maintenance complex component 4 [Source:HGNC Symbol;Acc:HGNC:6947] |
| 3.76E-46 | NCAPD3 | non-SMC condensin II complex, subunit D3 [Source:HGNC Symbol;Acc:HGNC:28952] |
| 3.81E-46 | VRK1 | vaccinia related kinase 1 [Source:HGNC Symbol;Acc:HGNC:12718] |
| 4.60E-46 | RECQL4 | RecQ protein-like 4 [Source:HGNC Symbol;Acc:HGNC:9949] |
| 5.87E-46 | SKP2 | S-phase kinase-associated protein 2, E3 ubiquitin protein ligase [Source:HGNC Symbol;Acc:HGNC:10901] |
| 7.30E-46 | DUT | deoxyuridine triphosphatase [Source:HGNC Symbol;Acc:HGNC:3078] |
| 1.38E-45 | MIS18A | MIS18 kinetochore protein A [Source:HGNC Symbol;Acc:HGNC:1286] |
| 2.64E-45 | SPDL1 | spindle apparatus coiled-coil protein 1 [Source:HGNC Symbol;Acc:HGNC:26010] |
| 3.06E-45 | TRAIP | TRAF interacting protein [Source:HGNC Symbol;Acc:HGNC:30764] |
