# Supplementary material for: PCNA-associated factor KIAA0101 transcriptionally induced by ELK1 controls cell proliferation and apoptosis in nasopharyngeal carcinoma: an integrated bioinformatics and experimental study
Source: Aging (Albany NY). 2020 Apr 9;12(7):5992–6017. doi: 10.18632/aging.102991 (PMC7185143; doi:10.18632/aging.102991)
Supplement: Supplementary Table 7 [file aging-12-102991-s006..docx]

**Supplementary Table 7**. **KIAA0101 co-expression gene lists in TCGA_HNSC and GSEA KEGG enrichment of positively correlated genes by Linkedomics.**

| Query | Statistic | P-value | FDR (BH) |
| --- | --- | --- | --- |
| KIAA0101 | 1 | 1.00E-110 | 1.00E-106 |
| CCNB2 | 0.7867894 | 1.25E-110 | 1.26E-106 |
| TIPIN | 0.7511525 | 1.72E-95 | 9.07E-92 |
| RAD51 | 0.7511033 | 1.80E-95 | 9.07E-92 |
| OIP5 | 0.7270192 | 1.26E-86 | 5.08E-83 |
| DUT | 0.7188542 | 7.72E-84 | 2.59E-80 |
| CDK1 | 0.6837461 | 6.66E-73 | 1.92E-69 |
| ZWINT | 0.6677766 | 2.01E-68 | 5.06E-65 |
| MND1 | 0.6607585 | 1.53E-66 | 3.43E-63 |
| HMGB2 | 0.6588518 | 4.87E-66 | 9.81E-63 |
| FANCI | 0.6580931 | 7.69E-66 | 1.41E-62 |
| ASF1B | 0.654013 | 8.83E-65 | 1.48E-61 |
| ZWILCH | 0.6528007 | 1.81E-64 | 2.81E-61 |
| DNAJC9 | 0.6517817 | 3.30E-64 | 4.75E-61 |
| NUSAP1 | 0.6471436 | 4.94E-63 | 6.64E-60 |
| UBE2T | 0.644452 | 2.32E-62 | 2.90E-59 |
| H2AFZ | 0.6443663 | 2.44E-62 | 2.90E-59 |
| MAD2L1 | 0.640749 | 1.91E-61 | 2.14E-58 |
| PBK | 0.6400456 | 2.84E-61 | 3.01E-58 |
| FAM96A | 0.63888 | 5.46E-61 | 5.51E-58 |
| RRM2 | 0.6353358 | 3.93E-60 | 3.78E-57 |
| KIFC1 | 0.632332 | 2.06E-59 | 1.88E-56 |
| CENPA | 0.6260478 | 6.16E-58 | 5.40E-55 |
| GINS2 | 0.6250661 | 1.04E-57 | 8.74E-55 |
| TROAP | 0.6244249 | 1.46E-57 | 1.18E-54 |
| GMNN | 0.6233382 | 2.61E-57 | 2.02E-54 |
| SPC25 | 0.6229179 | 3.25E-57 | 2.43E-54 |
| VRK1 | 0.6218856 | 5.61E-57 | 4.04E-54 |
| FEN1 | 0.6183226 | 3.62E-56 | 2.52E-53 |
| PPIL5 | 0.6181167 | 4.03E-56 | 2.71E-53 |
| CDCA5 | 0.6176079 | 5.25E-56 | 3.42E-53 |
| SFRS3 | 0.6161934 | 1.09E-55 | 6.88E-53 |
| CCNB1 | 0.6152955 | 1.74E-55 | 1.06E-52 |
| NUF2 | 0.614685 | 2.37E-55 | 1.41E-52 |
| KIF23 | 0.6134774 | 4.41E-55 | 2.54E-52 |
| SKA1 | 0.6133806 | 4.63E-55 | 2.59E-52 |
| PRC1 | 0.612053 | 9.11E-55 | 4.97E-52 |
| POLE2 | 0.608872 | 4.55E-54 | 2.42E-51 |
| CENPH | 0.606185 | 1.75E-53 | 9.03E-51 |
| SKA3 | 0.604126 | 4.85E-53 | 2.45E-50 |
| CDC25C | 0.6024935 | 1.09E-52 | 5.34E-50 |
| C1orf135 | 0.6009654 | 2.30E-52 | 1.10E-49 |
| UBE2C | 0.5980361 | 9.54E-52 | 4.48E-49 |
| PCNA | 0.5969505 | 1.61E-51 | 7.39E-49 |
| BUB1B | 0.5966057 | 1.90E-51 | 8.53E-49 |
| C15orf23 | 0.5965463 | 1.96E-51 | 8.59E-49 |
| PSMC3IP | 0.5955762 | 3.12E-51 | 1.34E-48 |
| RAD54L | 0.5946927 | 4.77E-51 | 2.00E-48 |
| CDC25A | 0.5946095 | 4.96E-51 | 2.04E-48 |
| CCNA2 | 0.5941852 | 6.08E-51 | 2.45E-48 |
| CDC45 | 0.5918305 | 1.86E-50 | 7.36E-48 |
| KIF2C | 0.5911152 | 2.61E-50 | 1.01E-47 |
| CDC6 | 0.590994 | 2.76E-50 | 1.05E-47 |
| POC1A | 0.5888465 | 7.59E-50 | 2.83E-47 |
| AURKB | 0.5885577 | 8.69E-50 | 3.19E-47 |
| TRAIP | 0.588102 | 1.08E-49 | 3.87E-47 |
| ORC6L | 0.5877547 | 1.26E-49 | 4.47E-47 |
| WDR76 | 0.5875379 | 1.40E-49 | 4.87E-47 |
| TK1 | 0.5874562 | 1.45E-49 | 4.97E-47 |
| RNASEH2A | 0.5859596 | 2.92E-49 | 9.80E-47 |
| ORC1L | 0.5855372 | 3.55E-49 | 1.17E-46 |
| BLM | 0.5849817 | 4.59E-49 | 1.49E-46 |
| EXO1 | 0.5842495 | 6.43E-49 | 2.06E-46 |
| CDT1 | 0.5827334 | 1.29E-48 | 4.07E-46 |
| PIF1 | 0.580368 | 3.81E-48 | 1.18E-45 |
| RFC2 | 0.5795007 | 5.65E-48 | 1.72E-45 |
| C21orf45 | 0.5784184 | 9.22E-48 | 2.77E-45 |
| CKS2 | 0.5783362 | 9.57E-48 | 2.84E-45 |
| ITGB3BP | 0.5765988 | 2.09E-47 | 6.11E-45 |
| GINS1 | 0.5762462 | 2.45E-47 | 7.06E-45 |
| C15orf42 | 0.5755808 | 3.30E-47 | 9.38E-45 |
| NEK2 | 0.5750653 | 4.16E-47 | 1.16E-44 |
| CDKN3 | 0.5735551 | 8.15E-47 | 2.25E-44 |
| C17orf53 | 0.5718371 | 1.75E-46 | 4.76E-44 |
| MCM10 | 0.5712119 | 2.30E-46 | 6.18E-44 |
| DSCC1 | 0.5703677 | 3.34E-46 | 8.77E-44 |
| DEPDC1B | 0.5703572 | 3.35E-46 | 8.77E-44 |
| SFRS7 | 0.5698018 | 4.28E-46 | 1.11E-43 |
| PLK4 | 0.5683036 | 8.24E-46 | 2.10E-43 |
| EXOSC9 | 0.5677168 | 1.06E-45 | 2.68E-43 |
| SPAG5 | 0.5664703 | 1.83E-45 | 4.55E-43 |
| CKS1B | 0.5658537 | 2.39E-45 | 5.88E-43 |
| NDC80 | 0.564519 | 4.25E-45 | 1.03E-42 |
| MLF1IP | 0.5636497 | 6.18E-45 | 1.48E-42 |
| PKMYT1 | 0.5632788 | 7.25E-45 | 1.72E-42 |
| PSRC1 | 0.5623506 | 1.08E-44 | 2.53E-42 |
| GTF2A2 | 0.5617436 | 1.40E-44 | 3.24E-42 |
| CENPW | 0.560862 | 2.04E-44 | 4.67E-42 |
| SKA2 | 0.5606842 | 2.20E-44 | 4.98E-42 |
| HMGN2 | 0.5606013 | 2.28E-44 | 5.10E-42 |
| BIRC5 | 0.5605307 | 2.35E-44 | 5.20E-42 |
| SUV39H1 | 0.5602722 | 2.62E-44 | 5.74E-42 |
| PPIH | 0.5599043 | 3.06E-44 | 6.63E-42 |
| C12orf48 | 0.5590144 | 4.46E-44 | 9.57E-42 |
| KIF11 | 0.5577247 | 7.69E-44 | 1.63E-41 |
| DTL | 0.5576368 | 7.98E-44 | 1.68E-41 |
| NCAPG | 0.5568378 | 1.12E-43 | 2.32E-41 |
| CDC20 | 0.5564087 | 1.34E-43 | 2.75E-41 |
| KIF22 | 0.5547584 | 2.66E-43 | 5.43E-41 |
| FAM72B | 0.5546171 | 2.83E-43 | 5.70E-41 |
| PTTG1 | 0.5538694 | 3.86E-43 | 7.70E-41 |
| LOC100128191 | 0.5534575 | 4.58E-43 | 9.05E-41 |
| EME1 | 0.5527236 | 6.21E-43 | 1.21E-40 |
| CDCA3 | 0.5504437 | 1.59E-42 | 3.08E-40 |
| PTTG3P | 0.5480478 | 4.24E-42 | 8.13E-40 |
| CENPK | 0.5480226 | 4.28E-42 | 8.14E-40 |
| CDCA2 | 0.5461785 | 9.05E-42 | 1.71E-39 |
| KIF4A | 0.5448717 | 1.54E-41 | 2.87E-39 |
| NASP | 0.5437604 | 2.40E-41 | 4.44E-39 |
| PRIM1 | 0.5437279 | 2.43E-41 | 4.46E-39 |
| C16orf59 | 0.5435058 | 2.66E-41 | 4.83E-39 |
| SGOL1 | 0.543441 | 2.73E-41 | 4.90E-39 |
| FAM54A | 0.5434235 | 2.75E-41 | 4.90E-39 |
| C1orf112 | 0.5430825 | 3.15E-41 | 5.57E-39 |
| PSMA4 | 0.54171 | 5.46E-41 | 9.57E-39 |
| TYMS | 0.5415885 | 5.73E-41 | 9.95E-39 |
| BUB3 | 0.5411325 | 6.87E-41 | 1.18E-38 |
| SNRPA | 0.5404413 | 9.04E-41 | 1.54E-38 |
| RPA3 | 0.5403124 | 9.52E-41 | 1.61E-38 |
| MCM7 | 0.5400443 | 1.06E-40 | 1.78E-38 |
| RFC5 | 0.537223 | 3.22E-40 | 5.37E-38 |
| HAUS8 | 0.5353139 | 6.81E-40 | 1.13E-37 |
| DSN1 | 0.5339726 | 1.15E-39 | 1.88E-37 |
| DLGAP5 | 0.5337699 | 1.24E-39 | 2.02E-37 |
| MELK | 0.5328096 | 1.80E-39 | 2.91E-37 |
| MYBL2 | 0.5323549 | 2.15E-39 | 3.44E-37 |
| AURKA | 0.5322015 | 2.28E-39 | 3.62E-37 |
| RFC4 | 0.5321552 | 2.32E-39 | 3.66E-37 |
| RACGAP1 | 0.5313651 | 3.15E-39 | 4.93E-37 |
| CHAF1A | 0.5312131 | 3.34E-39 | 5.18E-37 |
| RCC1 | 0.5295239 | 6.40E-39 | 9.84E-37 |
| CDCA8 | 0.5294037 | 6.70E-39 | 1.02E-36 |
| CENPM | 0.5290764 | 7.59E-39 | 1.15E-36 |
| SPC24 | 0.5284053 | 9.81E-39 | 1.48E-36 |
| GPN3 | 0.5283113 | 1.02E-38 | 1.52E-36 |
| HJURP | 0.5280412 | 1.13E-38 | 1.67E-36 |
| CHAF1B | 0.524429 | 4.43E-38 | 6.52E-36 |
| RCCD1 | 0.5243122 | 4.63E-38 | 6.76E-36 |
| FANCD2 | 0.5235237 | 6.22E-38 | 9.03E-36 |
| NUP37 | 0.5234445 | 6.41E-38 | 9.19E-36 |
| SNRPF | 0.523438 | 6.43E-38 | 9.19E-36 |
| KIF18B | 0.522841 | 8.04E-38 | 1.14E-35 |
| SNRPG | 0.5219817 | 1.11E-37 | 1.56E-35 |
| CDC7 | 0.5209896 | 1.61E-37 | 2.25E-35 |
| EXOSC8 | 0.5209557 | 1.63E-37 | 2.26E-35 |
| STMN1 | 0.5205752 | 1.87E-37 | 2.59E-35 |
| TTK | 0.5198008 | 2.50E-37 | 3.42E-35 |
| POLA2 | 0.5179998 | 4.86E-37 | 6.62E-35 |
| LSM6 | 0.5179433 | 4.96E-37 | 6.71E-35 |
| ESCO2 | 0.5178175 | 5.20E-37 | 6.99E-35 |
| CDCA7 | 0.5173065 | 6.27E-37 | 8.38E-35 |
| LSM3 | 0.516625 | 8.06E-37 | 1.07E-34 |
| MAGOH | 0.5159508 | 1.03E-36 | 1.36E-34 |
| RSL24D1 | 0.5154611 | 1.23E-36 | 1.61E-34 |
| CLN6 | 0.515456 | 1.24E-36 | 1.61E-34 |
| ARHGAP11A | 0.5140552 | 2.06E-36 | 2.66E-34 |
| DEPDC1 | 0.5136053 | 2.43E-36 | 3.12E-34 |
| MCM6 | 0.5128859 | 3.15E-36 | 4.02E-34 |
| LIG1 | 0.5125828 | 3.52E-36 | 4.46E-34 |
| MCM3 | 0.5118359 | 4.61E-36 | 5.81E-34 |
| RFC3 | 0.5106935 | 6.95E-36 | 8.71E-34 |
| TCF19 | 0.5096683 | 1.00E-35 | 1.25E-33 |
| RAD54B | 0.5095484 | 1.05E-35 | 1.30E-33 |
| C13orf27 | 0.5093919 | 1.11E-35 | 1.36E-33 |
| FANCA | 0.509191 | 1.19E-35 | 1.46E-33 |
| KIF15 | 0.5083373 | 1.62E-35 | 1.97E-33 |
| DBF4 | 0.5082959 | 1.64E-35 | 1.98E-33 |
| NEIL3 | 0.5067841 | 2.81E-35 | 3.38E-33 |
| DONSON | 0.5049851 | 5.32E-35 | 6.35E-33 |
| FANCB | 0.5023987 | 1.32E-34 | 1.57E-32 |
| SNUPN | 0.5017099 | 1.68E-34 | 1.98E-32 |
| SNRPA1 | 0.501139 | 2.05E-34 | 2.40E-32 |
| PDSS1 | 0.5009201 | 2.21E-34 | 2.58E-32 |
| TPX2 | 0.5005223 | 2.54E-34 | 2.95E-32 |
| LMNB1 | 0.5000571 | 2.99E-34 | 3.44E-32 |
| E2F1 | 0.4996805 | 3.41E-34 | 3.90E-32 |
| RBMX | 0.4995216 | 3.60E-34 | 4.10E-32 |
| ERCC6L | 0.4993644 | 3.80E-34 | 4.30E-32 |
| SNRNP40 | 0.4986684 | 4.83E-34 | 5.45E-32 |
| CENPN | 0.4980678 | 5.95E-34 | 6.67E-32 |
| HAUS1 | 0.4979261 | 6.25E-34 | 6.96E-32 |
| HNRNPL | 0.496654 | 9.68E-34 | 1.07E-31 |
| CACYBP | 0.4962377 | 1.12E-33 | 1.23E-31 |
| PTMA | 0.4957095 | 1.34E-33 | 1.47E-31 |
| SUV39H2 | 0.4956305 | 1.38E-33 | 1.50E-31 |
| CHTF18 | 0.4952849 | 1.55E-33 | 1.68E-31 |
| C15orf44 | 0.4947449 | 1.86E-33 | 2.01E-31 |
| C16orf75 | 0.4945655 | 1.98E-33 | 2.12E-31 |
| CCDC59 | 0.4924042 | 4.13E-33 | 4.40E-31 |
| XRCC3 | 0.4920826 | 4.60E-33 | 4.88E-31 |
| MCM5 | 0.4902443 | 8.55E-33 | 9.03E-31 |
| RAD51AP1 | 0.4899963 | 9.29E-33 | 9.76E-31 |
| TACC3 | 0.4897679 | 1.00E-32 | 1.05E-30 |
| OBFC2B | 0.4893539 | 1.15E-32 | 1.20E-30 |
| ZNF534 | 0.488983 | 1.31E-32 | 1.35E-30 |
| FBXO5 | 0.4877398 | 1.98E-32 | 2.04E-30 |
| CEP55 | 0.4872325 | 2.34E-32 | 2.40E-30 |
| TMPO | 0.4858849 | 3.67E-32 | 3.73E-30 |
| POLD1 | 0.4854289 | 4.26E-32 | 4.32E-30 |
| KPNA2 | 0.4843673 | 6.05E-32 | 6.10E-30 |
| HSPB11 | 0.4842133 | 6.37E-32 | 6.36E-30 |
| TUBA1B | 0.4842123 | 6.37E-32 | 6.36E-30 |
| CCNF | 0.4826553 | 1.06E-31 | 1.06E-29 |
| HELLS | 0.4822837 | 1.20E-31 | 1.19E-29 |
| UHRF1 | 0.4819476 | 1.34E-31 | 1.32E-29 |
| POLR3K | 0.4805398 | 2.12E-31 | 2.08E-29 |
| TPRKB | 0.4804694 | 2.17E-31 | 2.12E-29 |
| FANCG | 0.4795213 | 2.96E-31 | 2.87E-29 |
| BCL2L12 | 0.4794879 | 2.99E-31 | 2.88E-29 |
| SNRPD1 | 0.4790587 | 3.44E-31 | 3.30E-29 |
| POLE | 0.4787594 | 3.78E-31 | 3.62E-29 |
| TMEM106C | 0.4780512 | 4.76E-31 | 4.53E-29 |
| GINS4 | 0.4774406 | 5.80E-31 | 5.49E-29 |
| COX5A | 0.4772262 | 6.21E-31 | 5.85E-29 |
| HMGB1 | 0.476268 | 8.45E-31 | 7.93E-29 |
| SASS6 | 0.4757967 | 9.83E-31 | 9.18E-29 |
| VPS29 | 0.4757268 | 1.01E-30 | 9.34E-29 |
| DTYMK | 0.4737221 | 1.91E-30 | 1.76E-28 |
| SMC4 | 0.4730965 | 2.33E-30 | 2.14E-28 |
| ANP32A | 0.4724724 | 2.84E-30 | 2.60E-28 |
| TRIP13 | 0.471887 | 3.42E-30 | 3.12E-28 |
| PXMP2 | 0.4717662 | 3.55E-30 | 3.22E-28 |
| C9orf40 | 0.4698451 | 6.51E-30 | 5.88E-28 |
| MRPS11 | 0.4694689 | 7.32E-30 | 6.59E-28 |
| HNRNPA1 | 0.4688817 | 8.80E-30 | 7.89E-28 |
| LOC100130932 | 0.4688406 | 8.92E-30 | 7.96E-28 |
| MRPL46 | 0.4674595 | 1.37E-29 | 1.22E-27 |
| BUB1 | 0.4672532 | 1.46E-29 | 1.30E-27 |
| C19orf48 | 0.4668376 | 1.67E-29 | 1.47E-27 |
| SNRNP25 | 0.4663727 | 1.93E-29 | 1.69E-27 |
| PLK1 | 0.4661841 | 2.04E-29 | 1.78E-27 |
| DNA2 | 0.4659402 | 2.20E-29 | 1.92E-27 |
| HNRNPC | 0.4656787 | 2.39E-29 | 2.07E-27 |
| SIDT2 | -0.465338 | 2.66E-29 | 2.29E-27 |
| NUP62CL | 0.4642152 | 3.76E-29 | 3.23E-27 |
| KIF18A | 0.4640598 | 3.94E-29 | 3.37E-27 |
| RECQL4 | 0.4636259 | 4.51E-29 | 3.84E-27 |
| C12orf65 | 0.463156 | 5.21E-29 | 4.41E-27 |
| MTHFS | 0.461825 | 7.84E-29 | 6.61E-27 |
| EXOSC2 | 0.4616299 | 8.32E-29 | 6.99E-27 |
| CENPO | 0.461193 | 9.51E-29 | 7.96E-27 |
| SNRPE | 0.4598148 | 1.45E-28 | 1.21E-26 |
| WDR34 | 0.4597805 | 1.46E-28 | 1.21E-26 |
| APITD1 | 0.4597101 | 1.49E-28 | 1.23E-26 |
| MCM2 | 0.4587403 | 2.01E-28 | 1.65E-26 |
| SFRS2 | 0.4575057 | 2.91E-28 | 2.39E-26 |
| COMMD4 | 0.4568808 | 3.52E-28 | 2.87E-26 |
| TOE1 | 0.4568726 | 3.53E-28 | 2.87E-26 |
| C9orf100 | 0.455797 | 4.87E-28 | 3.95E-26 |
| MASTL | 0.4555027 | 5.32E-28 | 4.29E-26 |
| MRPL22 | 0.4550634 | 6.07E-28 | 4.88E-26 |
| FBXO22 | 0.4542757 | 7.69E-28 | 6.15E-26 |
| LSM5 | 0.4536775 | 9.19E-28 | 7.32E-26 |
| HNRNPA2B1 | 0.4535168 | 9.64E-28 | 7.65E-26 |
| ACVR1 | -0.4530686 | 1.10E-27 | 8.71E-26 |
| LSM2 | 0.452684 | 1.23E-27 | 9.73E-26 |
| C15orf63 | 0.4519113 | 1.55E-27 | 1.22E-25 |
| DLEU2 | 0.4518016 | 1.60E-27 | 1.25E-25 |
| TMEM97 | 0.4514062 | 1.80E-27 | 1.40E-25 |
| LOC81691 | 0.4512955 | 1.86E-27 | 1.45E-25 |
| ZNF511 | 0.4510689 | 1.99E-27 | 1.54E-25 |
| BCCIP | 0.4504424 | 2.40E-27 | 1.85E-25 |
| ANP32B | 0.4494893 | 3.17E-27 | 2.43E-25 |
| CALML4 | 0.4494689 | 3.19E-27 | 2.44E-25 |
| RPUSD2 | 0.4491485 | 3.51E-27 | 2.67E-25 |
| CEP78 | 0.4488156 | 3.87E-27 | 2.93E-25 |
| ACYP1 | 0.4481848 | 4.65E-27 | 3.51E-25 |
| PRPF38A | 0.4478496 | 5.13E-27 | 3.86E-25 |
| PA2G4 | 0.447451 | 5.77E-27 | 4.32E-25 |
| FBXO22OS | 0.4473769 | 5.89E-27 | 4.40E-25 |
| BAT1 | 0.4468864 | 6.80E-27 | 5.06E-25 |
| POLE3 | 0.4462449 | 8.20E-27 | 6.08E-25 |
| SH3PXD2B | -0.4458635 | 9.16E-27 | 6.76E-25 |
| CCNE2 | 0.4455229 | 1.01E-26 | 7.44E-25 |
| C21orf58 | 0.4450146 | 1.17E-26 | 8.59E-25 |
| EZH2 | 0.4447593 | 1.26E-26 | 9.19E-25 |
| FAM72D | 0.4447558 | 1.26E-26 | 9.19E-25 |
| ZCCHC24 | -0.4442167 | 1.48E-26 | 1.07E-24 |
| RAD51C | 0.4439075 | 1.61E-26 | 1.17E-24 |
| CWF19L1 | 0.4437813 | 1.67E-26 | 1.20E-24 |
| PPCDC | 0.4433937 | 1.87E-26 | 1.34E-24 |
| CENPQ | 0.443163 | 2.00E-26 | 1.43E-24 |
| CDK2 | 0.4428908 | 2.16E-26 | 1.54E-24 |
| SLC24A3 | -0.442628 | 2.33E-26 | 1.66E-24 |
| ZNF367 | 0.4411076 | 3.61E-26 | 2.55E-24 |
| SUMO2 | 0.440906 | 3.82E-26 | 2.69E-24 |
| RANBP1 | 0.4406922 | 4.06E-26 | 2.85E-24 |
| VBP1 | 0.4406433 | 4.12E-26 | 2.88E-24 |
| ESPL1 | 0.4404347 | 4.37E-26 | 3.05E-24 |
| PHF19 | 0.4403297 | 4.50E-26 | 3.13E-24 |
| HNRNPD | 0.4403124 | 4.52E-26 | 3.14E-24 |
| SFRS13A | 0.4402488 | 4.61E-26 | 3.18E-24 |
| NUDT1 | 0.439899 | 5.09E-26 | 3.50E-24 |
| DEK | 0.4396558 | 5.46E-26 | 3.74E-24 |
| FAM111B | 0.4394376 | 5.80E-26 | 3.97E-24 |
| RAD18 | 0.4392864 | 6.06E-26 | 4.13E-24 |
| CENPL | 0.4392459 | 6.13E-26 | 4.16E-24 |
| LRP1 | -0.4392291 | 6.16E-26 | 4.17E-24 |
| NUP85 | 0.4391365 | 6.32E-26 | 4.26E-24 |
| PDCD7 | 0.437782 | 9.28E-26 | 6.24E-24 |
| ENDOD1 | -0.4376515 | 9.63E-26 | 6.45E-24 |
| ATXN1 | -0.4371484 | 1.11E-25 | 7.41E-24 |
| MMP2 | -0.436995 | 1.16E-25 | 7.71E-24 |
| CHEK1 | 0.436296 | 1.41E-25 | 9.36E-24 |
| PTGER3 | -0.4354938 | 1.77E-25 | 1.17E-23 |
| H2AFV | 0.4353367 | 1.85E-25 | 1.22E-23 |
| BRCA1 | 0.4350027 | 2.03E-25 | 1.33E-23 |
| TIMELESS | 0.434375 | 2.42E-25 | 1.58E-23 |
| NSMCE4A | 0.4338015 | 2.84E-25 | 1.85E-23 |
| C4orf21 | 0.4337792 | 2.86E-25 | 1.86E-23 |
| KHDRBS1 | 0.4328394 | 3.71E-25 | 2.41E-23 |
| SLBP | 0.4328216 | 3.73E-25 | 2.41E-23 |
| HNRNPA1L2 | 0.4327271 | 3.83E-25 | 2.47E-23 |
| ATAD2 | 0.4326897 | 3.87E-25 | 2.49E-23 |
| ERH | 0.4317054 | 5.09E-25 | 3.26E-23 |
| H2AFX | 0.4313787 | 5.57E-25 | 3.55E-23 |
| C4orf27 | 0.4313751 | 5.57E-25 | 3.55E-23 |
| RHEBL1 | 0.4303827 | 7.33E-25 | 4.65E-23 |
| NFKBIL2 | 0.4302906 | 7.52E-25 | 4.75E-23 |
| COQ3 | 0.4300878 | 7.95E-25 | 5.01E-23 |
| LIN9 | 0.4299578 | 8.24E-25 | 5.18E-23 |
| LSM4 | 0.4297609 | 8.70E-25 | 5.45E-23 |
| SRRT | 0.4291427 | 1.03E-24 | 6.44E-23 |
| FANCL | 0.42846 | 1.24E-24 | 7.74E-23 |
| NR2C2AP | 0.428387 | 1.27E-24 | 7.88E-23 |
| HSPG2 | -0.427959 | 1.43E-24 | 8.83E-23 |
| NCAPH | 0.4277775 | 1.50E-24 | 9.25E-23 |
| PRIM2 | 0.4273509 | 1.69E-24 | 1.04E-22 |
| MRPL16 | 0.4273211 | 1.70E-24 | 1.04E-22 |
| EBP | 0.4270702 | 1.82E-24 | 1.11E-22 |
| NME1 | 0.426933 | 1.89E-24 | 1.15E-22 |
| CHCHD4 | 0.4268298 | 1.94E-24 | 1.18E-22 |
| KIF26B | -0.4268072 | 1.96E-24 | 1.18E-22 |
| SLC17A5 | -0.4264963 | 2.13E-24 | 1.28E-22 |
| PTGES3 | 0.4264755 | 2.14E-24 | 1.29E-22 |
| CDK4 | 0.4259372 | 2.48E-24 | 1.49E-22 |
| IMP3 | 0.4255545 | 2.75E-24 | 1.64E-22 |
| SAP30 | 0.4253234 | 2.93E-24 | 1.75E-22 |
| KNTC1 | 0.4252954 | 2.95E-24 | 1.75E-22 |
| C14orf143 | 0.4242072 | 3.96E-24 | 2.35E-22 |
| U2AF1 | 0.4241521 | 4.02E-24 | 2.38E-22 |
| MXD3 | 0.4238768 | 4.33E-24 | 2.55E-22 |
| DDX39 | 0.4235479 | 4.73E-24 | 2.78E-22 |
| CCDC150 | 0.4231071 | 5.33E-24 | 3.12E-22 |
| PLXDC2 | -0.4230082 | 5.47E-24 | 3.20E-22 |
| FH | 0.4229888 | 5.50E-24 | 3.21E-22 |
| GTSE1 | 0.4228922 | 5.65E-24 | 3.28E-22 |
| LRRC32 | -0.4221893 | 6.82E-24 | 3.95E-22 |
| UBE2S | 0.4217948 | 7.58E-24 | 4.38E-22 |
| MAP1A | -0.4217582 | 7.66E-24 | 4.41E-22 |
| DHFR | 0.42166 | 7.86E-24 | 4.51E-22 |
| GSG2 | 0.4204755 | 1.08E-23 | 6.18E-22 |
| HAUS2 | 0.4202568 | 1.14E-23 | 6.53E-22 |
| C18orf56 | 0.419996 | 1.23E-23 | 6.98E-22 |
| BOLA3 | 0.4196872 | 1.33E-23 | 7.56E-22 |
| FOXM1 | 0.4192505 | 1.50E-23 | 8.47E-22 |
| CACNA1C | -0.4185768 | 1.79E-23 | 1.01E-21 |
| PCDHGA2 | -0.4182065 | 1.97E-23 | 1.11E-21 |
| RACGAP1P | 0.4178645 | 2.16E-23 | 1.21E-21 |
| CCDC18 | 0.4177295 | 2.24E-23 | 1.25E-21 |
| HMGN1 | 0.4170159 | 2.70E-23 | 1.51E-21 |
| RBBP7 | 0.4169468 | 2.75E-23 | 1.53E-21 |
| RPL26L1 | 0.4168548 | 2.82E-23 | 1.57E-21 |
| TOP2A | 0.41668 | 2.95E-23 | 1.64E-21 |
| MRPL11 | 0.4166419 | 2.98E-23 | 1.65E-21 |
| POLQ | 0.4164462 | 3.14E-23 | 1.73E-21 |
| USP1 | 0.4155586 | 3.96E-23 | 2.18E-21 |
| SAE1 | 0.4151359 | 4.43E-23 | 2.43E-21 |
| POP5 | 0.414139 | 5.75E-23 | 3.14E-21 |
| KIF20A | 0.4136261 | 6.57E-23 | 3.58E-21 |
| CCDC58 | 0.4133629 | 7.04E-23 | 3.83E-21 |
| AK2 | 0.4132749 | 7.21E-23 | 3.91E-21 |
| TRA2B | 0.412918 | 7.91E-23 | 4.27E-21 |
| CHRNA5 | 0.4128438 | 8.06E-23 | 4.35E-21 |
| PDE3A | -0.4127504 | 8.26E-23 | 4.44E-21 |
| TIMP2 | -0.4127257 | 8.31E-23 | 4.46E-21 |
| THAP10 | 0.4124605 | 8.91E-23 | 4.76E-21 |
| DDR2 | -0.4117499 | 1.07E-22 | 5.71E-21 |
| C14orf80 | 0.4117257 | 1.08E-22 | 5.73E-21 |
| MRPL9 | 0.4115664 | 1.12E-22 | 5.95E-21 |
| C4orf43 | 0.4115609 | 1.12E-22 | 5.95E-21 |
| SNHG1 | 0.4114108 | 1.17E-22 | 6.17E-21 |
| C15orf21 | 0.4112907 | 1.21E-22 | 6.35E-21 |
| SIVA1 | 0.4111001 | 1.27E-22 | 6.65E-21 |
| HNRNPAB | 0.4109999 | 1.30E-22 | 6.81E-21 |
| MRVI1 | -0.4107108 | 1.40E-22 | 7.32E-21 |
| LAMA2 | -0.4100598 | 1.66E-22 | 8.64E-21 |
| DUSP3 | -0.4098843 | 1.73E-22 | 9.01E-21 |
| UQCRH | 0.4089132 | 2.23E-22 | 1.15E-20 |
| SHCBP1 | 0.4086712 | 2.37E-22 | 1.22E-20 |
| ANP32E | 0.408465 | 2.50E-22 | 1.29E-20 |
| SRP14 | 0.4083388 | 2.58E-22 | 1.33E-20 |
| SNRPC | 0.4083218 | 2.59E-22 | 1.33E-20 |
| GINS3 | 0.4083087 | 2.60E-22 | 1.33E-20 |
| ACP1 | 0.4082575 | 2.63E-22 | 1.34E-20 |
| DDX12 | 0.408061 | 2.77E-22 | 1.41E-20 |
| RPP30 | 0.4073714 | 3.30E-22 | 1.68E-20 |
| AIMP1 | 0.4072296 | 3.43E-22 | 1.74E-20 |
| COL6A3 | -0.407188 | 3.46E-22 | 1.75E-20 |
| HAUS5 | 0.4070009 | 3.63E-22 | 1.83E-20 |
| SFPQ | 0.4068618 | 3.76E-22 | 1.89E-20 |
| CENPE | 0.4066617 | 3.96E-22 | 1.98E-20 |
| ITGB5 | -0.406655 | 3.97E-22 | 1.98E-20 |
| SARNP | 0.4066541 | 3.97E-22 | 1.98E-20 |
| PCGF6 | 0.4064911 | 4.14E-22 | 2.06E-20 |
| CEP152 | 0.4060552 | 4.62E-22 | 2.29E-20 |
| ALG6 | 0.4059755 | 4.72E-22 | 2.34E-20 |
| CDKN2C | 0.4058348 | 4.89E-22 | 2.42E-20 |
| FILIP1L | -0.4058187 | 4.91E-22 | 2.42E-20 |
| NOP58 | 0.4058075 | 4.92E-22 | 2.42E-20 |
| STIL | 0.4051788 | 5.77E-22 | 2.83E-20 |
| DAZAP1 | 0.4051723 | 5.78E-22 | 2.83E-20 |
| IQGAP3 | 0.4048411 | 6.29E-22 | 3.07E-20 |
| MTMR3 | -0.4047788 | 6.39E-22 | 3.11E-20 |
| LSM12 | 0.4047145 | 6.49E-22 | 3.15E-20 |
| COX7B | 0.4043022 | 7.21E-22 | 3.49E-20 |
| ADAMTS2 | -0.4038315 | 8.12E-22 | 3.92E-20 |
| C2orf48 | 0.4036438 | 8.51E-22 | 4.10E-20 |
| MRPS36 | 0.4034914 | 8.84E-22 | 4.26E-20 |
| KDM6B | -0.4031686 | 9.59E-22 | 4.61E-20 |
| ANTXR1 | -0.4031282 | 9.69E-22 | 4.64E-20 |
| SF3A3 | 0.402905 | 1.03E-21 | 4.90E-20 |
| HSPE1 | 0.4027657 | 1.06E-21 | 5.06E-20 |
| TMEM85 | 0.402641 | 1.10E-21 | 5.21E-20 |
| MRPL35 | 0.4024126 | 1.16E-21 | 5.50E-20 |
| SNX19 | -0.4022446 | 1.21E-21 | 5.73E-20 |
| PRPS2 | 0.4021905 | 1.23E-21 | 5.79E-20 |
| RPA2 | 0.4020702 | 1.26E-21 | 5.96E-20 |
| NDUFB6 | 0.4019919 | 1.29E-21 | 6.06E-20 |
| C1orf77 | 0.401929 | 1.31E-21 | 6.14E-20 |
| SIK3 | -0.4015833 | 1.43E-21 | 6.68E-20 |
| DIO2 | -0.4015464 | 1.44E-21 | 6.73E-20 |
| COL24A1 | -0.401215 | 1.57E-21 | 7.29E-20 |
| C15orf40 | 0.4008123 | 1.73E-21 | 8.05E-20 |
| DBF4B | 0.4007528 | 1.76E-21 | 8.15E-20 |
| ZNF423 | -0.4004342 | 1.90E-21 | 8.80E-20 |
| MRPL39 | 0.400418 | 1.91E-21 | 8.82E-20 |
| PARP2 | 0.4002893 | 1.97E-21 | 9.09E-20 |
| ETFA | 0.3993552 | 2.49E-21 | 1.14E-19 |
| PSMG1 | 0.3981256 | 3.38E-21 | 1.55E-19 |
| COQ2 | 0.3980971 | 3.40E-21 | 1.56E-19 |
| CCDC99 | 0.398044 | 3.45E-21 | 1.57E-19 |
| SLC24A2 | -0.3979848 | 3.50E-21 | 1.59E-19 |
| COL12A1 | -0.3978739 | 3.60E-21 | 1.63E-19 |
| MACF1 | -0.3978711 | 3.60E-21 | 1.63E-19 |
| C19orf40 | 0.3976905 | 3.76E-21 | 1.70E-19 |
| BRI3BP | 0.3973097 | 4.13E-21 | 1.86E-19 |
| C17orf103 | -0.3971515 | 4.30E-21 | 1.93E-19 |
| PTTG2 | 0.3967282 | 4.77E-21 | 2.14E-19 |
| ZNF469 | -0.3966645 | 4.85E-21 | 2.17E-19 |
| DCTPP1 | 0.3962952 | 5.31E-21 | 2.37E-19 |
| NDUFA12 | 0.3962285 | 5.39E-21 | 2.41E-19 |
| FBLN2 | -0.3955333 | 6.40E-21 | 2.84E-19 |
| MITD1 | 0.3955331 | 6.40E-21 | 2.84E-19 |
| C4orf46 | 0.395474 | 6.49E-21 | 2.88E-19 |
| TCF4 | -0.3954579 | 6.52E-21 | 2.88E-19 |
| PLBD2 | -0.3954256 | 6.57E-21 | 2.90E-19 |
| CREB3L2 | -0.395256 | 6.85E-21 | 3.02E-19 |
| FIBIN | -0.3950168 | 7.26E-21 | 3.19E-19 |
| KIAA1524 | 0.3949131 | 7.45E-21 | 3.27E-19 |
| HMMR | 0.3948004 | 7.66E-21 | 3.35E-19 |
| LOC642846 | 0.3947852 | 7.69E-21 | 3.36E-19 |
| LAMB2 | -0.394719 | 7.81E-21 | 3.40E-19 |
| PTPLAD1 | 0.3944124 | 8.42E-21 | 3.66E-19 |
| COX6A1 | 0.3942372 | 8.79E-21 | 3.81E-19 |
| THOC1 | 0.394192 | 8.89E-21 | 3.85E-19 |
| CHEK2 | 0.394176 | 8.92E-21 | 3.85E-19 |
| COL1A1 | -0.3939372 | 9.46E-21 | 4.07E-19 |
| UQCRHL | 0.3936959 | 1.00E-20 | 4.31E-19 |
| GEN1 | 0.3935348 | 1.04E-20 | 4.48E-19 |
| GEMIN6 | 0.3933282 | 1.10E-20 | 4.70E-19 |
| PHF5A | 0.3932825 | 1.11E-20 | 4.74E-19 |
| ADAMTS12 | -0.3932682 | 1.11E-20 | 4.75E-19 |
| TBC1D16 | -0.3932542 | 1.12E-20 | 4.75E-19 |
| SESN3 | -0.393043 | 1.18E-20 | 4.99E-19 |
| TMEM127 | -0.3929677 | 1.20E-20 | 5.07E-19 |
| SRP9 | 0.3928546 | 1.23E-20 | 5.20E-19 |
| SPATA5L1 | 0.3928208 | 1.24E-20 | 5.24E-19 |
| PRKG1 | -0.3927938 | 1.25E-20 | 5.26E-19 |
| COL5A1 | -0.392688 | 1.28E-20 | 5.39E-19 |
| NUDCD2 | 0.392442 | 1.36E-20 | 5.70E-19 |
| WDR61 | 0.3924151 | 1.37E-20 | 5.73E-19 |
| YPEL2 | -0.3918887 | 1.56E-20 | 6.50E-19 |
| AURKAPS1 | 0.3913093 | 1.79E-20 | 7.46E-19 |
| TAF5 | 0.3910438 | 1.91E-20 | 7.94E-19 |
| C17orf42 | 0.3908704 | 1.99E-20 | 8.26E-19 |
| HNRNPF | 0.3907067 | 2.07E-20 | 8.58E-19 |
| PCDHGA3 | -0.3906833 | 2.08E-20 | 8.61E-19 |
| CPSF3 | 0.3906007 | 2.12E-20 | 8.76E-19 |
| PAM | -0.3899031 | 2.51E-20 | 1.03E-18 |
| CRISPLD2 | -0.3896954 | 2.64E-20 | 1.09E-18 |
| BICC1 | -0.3895291 | 2.75E-20 | 1.13E-18 |
| WRAP53 | 0.3890443 | 3.09E-20 | 1.26E-18 |
| TNS1 | -0.3889546 | 3.16E-20 | 1.29E-18 |
| SHANK1 | -0.3887962 | 3.28E-20 | 1.34E-18 |
| SAC3D1 | 0.3887726 | 3.30E-20 | 1.34E-18 |
| WIPI1 | -0.3886896 | 3.36E-20 | 1.37E-18 |
| SORCS2 | -0.3886402 | 3.40E-20 | 1.38E-18 |
| GGCT | 0.3886333 | 3.41E-20 | 1.38E-18 |
| SFRS1 | 0.3885728 | 3.46E-20 | 1.40E-18 |
| FAM114A1 | -0.3882886 | 3.70E-20 | 1.49E-18 |
| CHPF2 | -0.3882423 | 3.75E-20 | 1.50E-18 |
| TSHZ3 | -0.3881991 | 3.78E-20 | 1.52E-18 |
| DNAJC8 | 0.3879918 | 3.98E-20 | 1.59E-18 |
| COL1A2 | -0.3877702 | 4.19E-20 | 1.67E-18 |
| DPY30 | 0.387464 | 4.51E-20 | 1.80E-18 |
| MEF2D | -0.3873567 | 4.63E-20 | 1.84E-18 |
| PRICKLE1 | -0.3872653 | 4.73E-20 | 1.88E-18 |
| SGOL2 | 0.3871062 | 4.91E-20 | 1.95E-18 |
| HPRT1 | 0.3869686 | 5.08E-20 | 2.01E-18 |
| ZMYND19 | 0.3868425 | 5.23E-20 | 2.06E-18 |
| BCAS2 | 0.3868178 | 5.26E-20 | 2.07E-18 |
| CDC42BPB | -0.3866667 | 5.45E-20 | 2.14E-18 |
| CCHCR1 | 0.3865728 | 5.58E-20 | 2.19E-18 |
| ANGPTL2 | -0.3862147 | 6.07E-20 | 2.38E-18 |
| SS18L2 | 0.3857652 | 6.76E-20 | 2.64E-18 |
| MAML2 | -0.3857082 | 6.85E-20 | 2.67E-18 |
| GTF3C1 | -0.3855629 | 7.09E-20 | 2.76E-18 |
| MRPL37 | 0.3848846 | 8.33E-20 | 3.23E-18 |
| POLR2H | 0.3845299 | 9.05E-20 | 3.51E-18 |
| SGCD | -0.3838067 | 1.07E-19 | 4.16E-18 |
| HINT1 | 0.3837865 | 1.08E-19 | 4.17E-18 |
| NUDT5 | 0.3837768 | 1.08E-19 | 4.17E-18 |
| PKNOX2 | -0.3835647 | 1.14E-19 | 4.38E-18 |
| ASPM | 0.3835068 | 1.15E-19 | 4.43E-18 |
| PDGFRB | -0.383495 | 1.16E-19 | 4.43E-18 |
| GAB2 | -0.3833762 | 1.19E-19 | 4.55E-18 |
| GAS7 | -0.3830082 | 1.30E-19 | 4.95E-18 |
| MKI67 | 0.3829748 | 1.31E-19 | 4.98E-18 |
| LRP10 | -0.3828209 | 1.35E-19 | 5.15E-18 |
| MTHFD1 | 0.3827971 | 1.36E-19 | 5.17E-18 |
| ZFYVE19 | 0.3824905 | 1.46E-19 | 5.55E-18 |
| CBX3 | 0.3824383 | 1.48E-19 | 5.60E-18 |
| KIF20B | 0.3824267 | 1.49E-19 | 5.61E-18 |
| SH3PXD2A | -0.3816712 | 1.77E-19 | 6.68E-18 |
| IL1R1 | -0.3815161 | 1.84E-19 | 6.92E-18 |
| MRPL1 | 0.3812843 | 1.94E-19 | 7.29E-18 |
| CDKN2AIPNL | 0.3811201 | 2.02E-19 | 7.56E-18 |
| AEBP1 | -0.3809828 | 2.08E-19 | 7.79E-18 |
| ADPGK | 0.3809428 | 2.10E-19 | 7.85E-18 |
| GJA1 | -0.3809079 | 2.12E-19 | 7.90E-18 |
| SLC25A19 | 0.3808755 | 2.14E-19 | 7.95E-18 |
| FBXO43 | 0.3807735 | 2.19E-19 | 8.12E-18 |
| GALNTL2 | -0.3805793 | 2.29E-19 | 8.48E-18 |
| MTBP | 0.3805226 | 2.32E-19 | 8.58E-18 |
| KIAA0913 | -0.3804881 | 2.34E-19 | 8.63E-18 |
| COL5A2 | -0.380473 | 2.35E-19 | 8.65E-18 |
| PA2G4P4 | 0.380429 | 2.37E-19 | 8.72E-18 |
| COL3A1 | -0.3800953 | 2.56E-19 | 9.41E-18 |
| RPS17 | 0.3800695 | 2.58E-19 | 9.45E-18 |
| SSC5D | -0.3799689 | 2.64E-19 | 9.65E-18 |
| THOC4 | 0.3798734 | 2.70E-19 | 9.85E-18 |
| EPR1 | 0.3797473 | 2.78E-19 | 1.01E-17 |
| TBCA | 0.3795409 | 2.91E-19 | 1.06E-17 |
| HIST1H2AJ | 0.3790152 | 3.29E-19 | 1.19E-17 |
| NPM1 | 0.3790138 | 3.29E-19 | 1.19E-17 |
| TCOF1 | 0.3789849 | 3.31E-19 | 1.20E-17 |
| C11orf82 | 0.3786144 | 3.61E-19 | 1.31E-17 |
| MAGOHB | 0.3786065 | 3.62E-19 | 1.31E-17 |
| HMCN1 | -0.3785516 | 3.66E-19 | 1.32E-17 |
| GLIS3 | -0.3785222 | 3.69E-19 | 1.33E-17 |
| PAFAH1B3 | 0.3785034 | 3.71E-19 | 1.33E-17 |
| C4orf31 | -0.3784939 | 3.71E-19 | 1.33E-17 |
| HSPA14 | 0.3784767 | 3.73E-19 | 1.33E-17 |
| AHNAK | -0.3783097 | 3.88E-19 | 1.38E-17 |
| PCDHGA12 | -0.3781972 | 3.98E-19 | 1.42E-17 |
| TUBG1 | 0.3780334 | 4.13E-19 | 1.47E-17 |
| SNRPB | 0.3777796 | 4.38E-19 | 1.56E-17 |
| ATP5J2 | 0.3775832 | 4.58E-19 | 1.62E-17 |
| TMEM18 | 0.3773859 | 4.80E-19 | 1.70E-17 |
| TCP11L2 | -0.3773345 | 4.85E-19 | 1.71E-17 |
| SPON1 | -0.3772419 | 4.96E-19 | 1.75E-17 |
| MSH2 | 0.3772241 | 4.98E-19 | 1.75E-17 |
| POP7 | 0.3771364 | 5.08E-19 | 1.79E-17 |
| CLEC2D | 0.377082 | 5.15E-19 | 1.80E-17 |
| JAM3 | -0.3770515 | 5.18E-19 | 1.81E-17 |
| SF3B14 | 0.376823 | 5.46E-19 | 1.91E-17 |
| E2F2 | 0.3768128 | 5.47E-19 | 1.91E-17 |
| MRPS18C | 0.37676 | 5.54E-19 | 1.93E-17 |
| PPFIBP1 | -0.3767366 | 5.57E-19 | 1.94E-17 |
| WDR73 | 0.3764836 | 5.90E-19 | 2.05E-17 |
| BCL9L | -0.3759971 | 6.60E-19 | 2.29E-17 |
| MYLK | -0.3759461 | 6.68E-19 | 2.31E-17 |
| THOC7 | 0.3759062 | 6.74E-19 | 2.33E-17 |
| FBL | 0.3757617 | 6.97E-19 | 2.40E-17 |
| CISD2 | 0.3753855 | 7.60E-19 | 2.61E-17 |
| FANCC | 0.3753312 | 7.69E-19 | 2.64E-17 |
| BMPR2 | -0.37529 | 7.76E-19 | 2.66E-17 |
| KIAA0355 | -0.3750488 | 8.20E-19 | 2.81E-17 |
| NRM | 0.3749295 | 8.43E-19 | 2.88E-17 |
| CCDC34 | 0.3744202 | 9.47E-19 | 3.23E-17 |
| PCDHB18 | -0.3744078 | 9.50E-19 | 3.24E-17 |
| AAK1 | -0.3741226 | 1.01E-18 | 3.45E-17 |
| LRDD | 0.3740133 | 1.04E-18 | 3.53E-17 |
| RPS7 | 0.3739465 | 1.06E-18 | 3.58E-17 |
| DLGAP4 | -0.3739045 | 1.07E-18 | 3.60E-17 |
| LIMCH1 | -0.3738975 | 1.07E-18 | 3.60E-17 |
| CPSF4 | 0.3738945 | 1.07E-18 | 3.60E-17 |
| SPARC | -0.3738386 | 1.08E-18 | 3.64E-17 |
| SET | 0.3738271 | 1.08E-18 | 3.64E-17 |
| PAQR4 | 0.3737496 | 1.10E-18 | 3.70E-17 |
| HYLS1 | 0.3737004 | 1.12E-18 | 3.73E-17 |
| MRPS16 | 0.3736988 | 1.12E-18 | 3.73E-17 |
| TMEM183A | 0.3730612 | 1.29E-18 | 4.31E-17 |
| RBM17 | 0.3728229 | 1.36E-18 | 4.54E-17 |
| JAK1 | -0.3728006 | 1.37E-18 | 4.56E-17 |
| NKD1 | -0.3726685 | 1.41E-18 | 4.69E-17 |
| FOSL2 | -0.3726456 | 1.42E-18 | 4.70E-17 |
| KIF4B | 0.3724653 | 1.48E-18 | 4.89E-17 |
| ZNF521 | -0.3722471 | 1.55E-18 | 5.13E-17 |
| ARCN1 | -0.3722361 | 1.56E-18 | 5.14E-17 |
| ZBTB4 | -0.3721735 | 1.58E-18 | 5.20E-17 |
| ZHX3 | -0.3719738 | 1.65E-18 | 5.43E-17 |
| ZFYVE1 | -0.3718886 | 1.68E-18 | 5.53E-17 |
| MUTYH | 0.3717705 | 1.73E-18 | 5.67E-17 |
| ADD1 | -0.371589 | 1.80E-18 | 5.90E-17 |
| THBS2 | -0.3715247 | 1.83E-18 | 5.96E-17 |
| DHRS13 | 0.3715238 | 1.83E-18 | 5.96E-17 |
| GREM1 | -0.3713024 | 1.92E-18 | 6.26E-17 |
| SFXN2 | 0.371094 | 2.01E-18 | 6.55E-17 |
| CHCHD1 | 0.3710282 | 2.04E-18 | 6.64E-17 |
| SNRPD2 | 0.370784 | 2.16E-18 | 7.00E-17 |
| PALLD | -0.3706619 | 2.22E-18 | 7.19E-17 |
| ZNRD1 | 0.3705617 | 2.27E-18 | 7.34E-17 |
| CLSTN2 | -0.3704782 | 2.31E-18 | 7.47E-17 |
| DEPDC4 | 0.3703217 | 2.40E-18 | 7.71E-17 |
| PCDHGC3 | -0.3703201 | 2.40E-18 | 7.71E-17 |
| DNAJC3 | -0.3699137 | 2.63E-18 | 8.43E-17 |
| IGF2R | -0.3699094 | 2.63E-18 | 8.43E-17 |
| TOMM5 | 0.3698114 | 2.69E-18 | 8.60E-17 |
| CNOT10 | 0.3697136 | 2.75E-18 | 8.78E-17 |
| COL6A1 | -0.3696176 | 2.81E-18 | 8.96E-17 |
| TRIAP1 | 0.3695856 | 2.83E-18 | 9.01E-17 |
| MN1 | -0.3693853 | 2.96E-18 | 9.41E-17 |
| EEF1E1 | 0.3692818 | 3.03E-18 | 9.61E-17 |
| F13A1 | -0.3691201 | 3.14E-18 | 9.94E-17 |
| MTFMT | 0.3691178 | 3.14E-18 | 9.94E-17 |
| GPR124 | -0.3690722 | 3.17E-18 | 1.00E-16 |
| COPS3 | 0.3687355 | 3.42E-18 | 1.08E-16 |
| ANKRD32 | 0.3685638 | 3.56E-18 | 1.12E-16 |
| PAICS | 0.3685441 | 3.57E-18 | 1.12E-16 |
| ZEB1 | -0.3684839 | 3.62E-18 | 1.14E-16 |
| CETN3 | 0.3683468 | 3.73E-18 | 1.17E-16 |
| SCARF2 | -0.3681499 | 3.90E-18 | 1.22E-16 |
| RRM1 | 0.3679227 | 4.10E-18 | 1.28E-16 |
| FAF1 | 0.3677884 | 4.23E-18 | 1.32E-16 |
| COL8A1 | -0.3677265 | 4.29E-18 | 1.34E-16 |
| HEPH | -0.3676904 | 4.32E-18 | 1.34E-16 |
| CENPJ | 0.3673619 | 4.65E-18 | 1.44E-16 |
| SPON2 | -0.3673036 | 4.71E-18 | 1.46E-16 |
| C6orf182 | 0.3670988 | 4.93E-18 | 1.53E-16 |
| LAMA4 | -0.3668302 | 5.23E-18 | 1.62E-16 |
| PCDHGA5 | -0.366818 | 5.25E-18 | 1.62E-16 |
| LAS1L | 0.3666935 | 5.39E-18 | 1.66E-16 |
| FBN1 | -0.3666718 | 5.42E-18 | 1.67E-16 |
| CIT | 0.3666184 | 5.48E-18 | 1.69E-16 |
| FAM72A | 0.3660653 | 6.20E-18 | 1.90E-16 |
| C20orf194 | -0.365618 | 6.85E-18 | 2.10E-16 |
| CMC1 | 0.3655533 | 6.94E-18 | 2.12E-16 |
| MNS1 | 0.3655142 | 7.00E-18 | 2.14E-16 |
| IGF2 | -0.3654012 | 7.18E-18 | 2.19E-16 |
| CSTF3 | 0.3653347 | 7.29E-18 | 2.22E-16 |
| C14orf37 | -0.3652649 | 7.40E-18 | 2.25E-16 |
| DDX55 | 0.3652114 | 7.49E-18 | 2.27E-16 |
| ARHGEF12 | -0.36519 | 7.53E-18 | 2.28E-16 |
| RAN | 0.3651151 | 7.65E-18 | 2.32E-16 |
| KIAA0513 | -0.3650918 | 7.69E-18 | 2.32E-16 |
| FYCO1 | -0.36499 | 7.87E-18 | 2.37E-16 |
| EDIL3 | -0.364606 | 8.56E-18 | 2.58E-16 |
| LEAP2 | 0.3645713 | 8.63E-18 | 2.60E-16 |
| SFXN4 | 0.3645226 | 8.72E-18 | 2.62E-16 |
| HADH | 0.364027 | 9.73E-18 | 2.92E-16 |
| HNRNPA3P1 | 0.3639057 | 9.99E-18 | 2.99E-16 |
| HAUS7 | 0.363905 | 9.99E-18 | 2.99E-16 |
| QSOX1 | -0.3637486 | 1.03E-17 | 3.09E-16 |
| ZNHIT3 | 0.3637092 | 1.04E-17 | 3.11E-16 |
| FAM198B | -0.3636123 | 1.07E-17 | 3.17E-16 |
| MRPL42 | 0.3635097 | 1.09E-17 | 3.24E-16 |
| DYSF | -0.3634762 | 1.10E-17 | 3.26E-16 |
| UNG | 0.3633332 | 1.13E-17 | 3.36E-16 |
| ERN1 | -0.3632493 | 1.15E-17 | 3.42E-16 |
| NCAPH2 | 0.3626907 | 1.30E-17 | 3.86E-16 |
| ODZ4 | -0.3626454 | 1.32E-17 | 3.88E-16 |
| COL5A3 | -0.3626453 | 1.32E-17 | 3.88E-16 |
| ATP5G1 | 0.362476 | 1.37E-17 | 4.02E-16 |
| MXRA5 | -0.3624297 | 1.38E-17 | 4.06E-16 |
| MRPL50 | 0.3623887 | 1.39E-17 | 4.09E-16 |
| WDR67 | 0.3623478 | 1.41E-17 | 4.12E-16 |
| SEC11A | 0.3621536 | 1.47E-17 | 4.29E-16 |
| UBAC1 | 0.3619614 | 1.53E-17 | 4.47E-16 |
| IMMP1L | 0.3617576 | 1.60E-17 | 4.67E-16 |
| EVC | -0.3616791 | 1.63E-17 | 4.74E-16 |
| C17orf91 | -0.3616096 | 1.65E-17 | 4.81E-16 |
| MDH1 | 0.3614485 | 1.71E-17 | 4.97E-16 |
| DYNC1H1 | -0.3613083 | 1.76E-17 | 5.12E-16 |
| ALKBH2 | 0.3610656 | 1.86E-17 | 5.39E-16 |
| VDAC3 | 0.3609525 | 1.91E-17 | 5.51E-16 |
| VAT1 | -0.3608491 | 1.95E-17 | 5.63E-16 |
| CENPF | 0.3608195 | 1.96E-17 | 5.66E-16 |
| SHMT2 | 0.3606597 | 2.03E-17 | 5.85E-16 |
| DAAM2 | -0.3606254 | 2.05E-17 | 5.89E-16 |
| C16orf45 | -0.3603774 | 2.16E-17 | 6.20E-16 |
| BGN | -0.3601661 | 2.26E-17 | 6.49E-16 |
| SFRP2 | -0.3601394 | 2.27E-17 | 6.51E-16 |
| C13orf37 | 0.3599454 | 2.37E-17 | 6.79E-16 |
| HIRIP3 | 0.3598594 | 2.42E-17 | 6.90E-16 |
| SGIP1 | -0.359848 | 2.42E-17 | 6.91E-16 |
| C12orf32 | 0.3598119 | 2.44E-17 | 6.95E-16 |
| IQCC | 0.3597433 | 2.48E-17 | 7.05E-16 |
| NCAPG2 | 0.3594941 | 2.62E-17 | 7.43E-16 |
| TIMP3 | -0.3594162 | 2.66E-17 | 7.55E-16 |
| GLRX2 | 0.3593677 | 2.69E-17 | 7.61E-16 |
| RUNX1T1 | -0.3593488 | 2.70E-17 | 7.63E-16 |
| TRNAU1AP | 0.3593276 | 2.71E-17 | 7.66E-16 |
| LATS2 | -0.3590457 | 2.88E-17 | 8.13E-16 |
| KIAA1462 | -0.3590172 | 2.90E-17 | 8.17E-16 |
| NHP2 | 0.3589918 | 2.92E-17 | 8.20E-16 |
| DACT1 | -0.3588585 | 3.00E-17 | 8.43E-16 |
| NDUFA2 | 0.3588439 | 3.01E-17 | 8.44E-16 |
| ADAMTS7 | -0.3587982 | 3.04E-17 | 8.52E-16 |
| HAUS4 | 0.3586745 | 3.12E-17 | 8.73E-16 |
| OXNAD1 | 0.3583315 | 3.36E-17 | 9.39E-16 |
| TRIM62 | -0.3582325 | 3.44E-17 | 9.58E-16 |
| SLC25A5 | 0.3580458 | 3.58E-17 | 9.96E-16 |
| PPFIA2 | -0.3579882 | 3.62E-17 | 1.01E-15 |
| APOO | 0.3577584 | 3.81E-17 | 1.06E-15 |
| C9orf84 | 0.3574936 | 4.03E-17 | 1.12E-15 |
| MAPKAPK5 | 0.3573221 | 4.18E-17 | 1.16E-15 |
| ATP5C1 | 0.3570235 | 4.46E-17 | 1.23E-15 |
| BOC | -0.3568479 | 4.63E-17 | 1.28E-15 |
| MXRA8 | -0.3566852 | 4.79E-17 | 1.32E-15 |
| TECPR2 | -0.3566635 | 4.81E-17 | 1.33E-15 |
| CCDC138 | 0.356609 | 4.87E-17 | 1.34E-15 |
| LPPR2 | -0.3565343 | 4.95E-17 | 1.36E-15 |
| LTBP2 | -0.3565259 | 4.96E-17 | 1.36E-15 |
| KIF1B | -0.3564431 | 5.05E-17 | 1.38E-15 |
| RPL4 | 0.3563365 | 5.16E-17 | 1.41E-15 |
| CDCA4 | 0.3561243 | 5.40E-17 | 1.48E-15 |
| CLIP2 | -0.3561187 | 5.41E-17 | 1.48E-15 |
| AKAP12 | -0.3560641 | 5.47E-17 | 1.49E-15 |
| NAP1L1 | 0.3559406 | 5.62E-17 | 1.53E-15 |
| TDP1 | 0.3559011 | 5.67E-17 | 1.54E-15 |
| METTL10 | 0.3558606 | 5.72E-17 | 1.55E-15 |
| DNAJC17 | 0.355841 | 5.74E-17 | 1.56E-15 |
| SVIL | -0.355494 | 6.18E-17 | 1.67E-15 |
| COL6A2 | -0.3553222 | 6.41E-17 | 1.73E-15 |
| FLNC | -0.3551675 | 6.63E-17 | 1.79E-15 |
| FNIP2 | -0.355102 | 6.72E-17 | 1.81E-15 |
| NID2 | -0.3550312 | 6.82E-17 | 1.84E-15 |
| TOM1 | -0.3548552 | 7.09E-17 | 1.90E-15 |
| ZFAND3 | -0.3548422 | 7.11E-17 | 1.91E-15 |
| EML1 | -0.354783 | 7.20E-17 | 1.93E-15 |
| MRTO4 | 0.3547749 | 7.21E-17 | 1.93E-15 |
| FKBP9 | -0.354703 | 7.32E-17 | 1.96E-15 |
| TNN | -0.3546662 | 7.38E-17 | 1.97E-15 |
| C18orf55 | 0.3545334 | 7.59E-17 | 2.02E-15 |
| RIBC2 | 0.3545191 | 7.61E-17 | 2.03E-15 |
| COL18A1 | -0.3544514 | 7.72E-17 | 2.05E-15 |
| EMX2OS | -0.3544267 | 7.76E-17 | 2.06E-15 |
| IGDCC4 | -0.3543201 | 7.94E-17 | 2.11E-15 |
| SMC2 | 0.3542699 | 8.03E-17 | 2.13E-15 |
| HIVEP2 | -0.3542513 | 8.06E-17 | 2.13E-15 |
| CSMD2 | -0.3541052 | 8.31E-17 | 2.20E-15 |
| DCLRE1B | 0.3540903 | 8.34E-17 | 2.20E-15 |
| DCLK1 | -0.3537862 | 8.89E-17 | 2.34E-15 |
| EBF2 | -0.3536982 | 9.06E-17 | 2.39E-15 |
| CPEB4 | -0.3535126 | 9.43E-17 | 2.48E-15 |
| THUMPD2 | 0.3534984 | 9.45E-17 | 2.48E-15 |
| UPF3B | 0.3534975 | 9.46E-17 | 2.48E-15 |
| RUNDC2A | -0.3534854 | 9.48E-17 | 2.48E-15 |
| PSIP1 | 0.3534738 | 9.50E-17 | 2.49E-15 |
| HEG1 | -0.3531735 | 1.01E-16 | 2.65E-15 |
| LYSMD2 | 0.3531114 | 1.03E-16 | 2.68E-15 |
| WFS1 | -0.3530754 | 1.03E-16 | 2.69E-15 |
| PMF1 | 0.3529529 | 1.06E-16 | 2.76E-15 |
| C1orf35 | 0.3526906 | 1.12E-16 | 2.91E-15 |
| TULP4 | -0.3526811 | 1.12E-16 | 2.92E-15 |
| PDHA1 | 0.3524795 | 1.17E-16 | 3.04E-15 |
| APTX | 0.3524434 | 1.18E-16 | 3.06E-15 |
| MPZL3 | -0.3523676 | 1.20E-16 | 3.10E-15 |
| CDKL5 | -0.3523083 | 1.22E-16 | 3.14E-15 |
| SERINC1 | -0.3519361 | 1.32E-16 | 3.39E-15 |
| RPLP1 | 0.3518442 | 1.34E-16 | 3.45E-15 |
| HIST2H2AC | 0.351828 | 1.35E-16 | 3.46E-15 |
| IGLON5 | -0.3518211 | 1.35E-16 | 3.46E-15 |
| ZMIZ1 | -0.3517607 | 1.36E-16 | 3.50E-15 |
| KIRREL | -0.3515893 | 1.42E-16 | 3.63E-15 |
| TMEM180 | 0.3513724 | 1.48E-16 | 3.79E-15 |
| UBL7 | 0.3513445 | 1.49E-16 | 3.81E-15 |
| TSEN34 | 0.3511913 | 1.54E-16 | 3.93E-15 |
| DLC1 | -0.3511113 | 1.56E-16 | 3.99E-15 |
| PSPC1 | 0.351034 | 1.59E-16 | 4.05E-15 |
| ZNF684 | 0.3509743 | 1.61E-16 | 4.10E-15 |
| FAM190B | -0.3509365 | 1.62E-16 | 4.12E-15 |
| DCLRE1C | 0.3506886 | 1.71E-16 | 4.34E-15 |
| RPF1 | 0.3506409 | 1.73E-16 | 4.38E-15 |
| FAM103A1 | 0.350629 | 1.73E-16 | 4.38E-15 |
| UQCRC1 | 0.3506098 | 1.74E-16 | 4.39E-15 |
| SIP1 | 0.3506087 | 1.74E-16 | 4.39E-15 |
| LOC723972 | 0.3504546 | 1.80E-16 | 4.53E-15 |
| SERINC3 | -0.3503934 | 1.82E-16 | 4.58E-15 |
| VPS13D | -0.3503179 | 1.85E-16 | 4.65E-15 |
| ATRN | -0.3502208 | 1.89E-16 | 4.74E-15 |
| GPR56 | -0.3501613 | 1.91E-16 | 4.79E-15 |
| TARDBP | 0.3500709 | 1.95E-16 | 4.88E-15 |
| CCDC72 | 0.3499718 | 1.99E-16 | 4.97E-15 |
| ICT1 | 0.349864 | 2.03E-16 | 5.08E-15 |
| KIF24 | 0.3498529 | 2.04E-16 | 5.08E-15 |
| RNASEH2B | 0.3498218 | 2.05E-16 | 5.11E-15 |
| FOXN3 | -0.3497902 | 2.06E-16 | 5.14E-15 |
| ERAL1 | 0.3497844 | 2.07E-16 | 5.14E-15 |
| E2F7 | 0.3497508 | 2.08E-16 | 5.17E-15 |
| NDUFC1 | 0.3497262 | 2.09E-16 | 5.19E-15 |
| ODZ2 | -0.349656 | 2.12E-16 | 5.26E-15 |
| ACSS3 | -0.3496242 | 2.14E-16 | 5.29E-15 |
| PSMA3 | 0.349567 | 2.16E-16 | 5.34E-15 |
| RHOJ | -0.3494813 | 2.20E-16 | 5.43E-15 |
| F2R | -0.349455 | 2.21E-16 | 5.46E-15 |
| NDUFS3 | 0.3489177 | 2.48E-16 | 6.10E-15 |
| WDHD1 | 0.3487851 | 2.55E-16 | 6.26E-15 |
| CACNA1G | -0.3485867 | 2.65E-16 | 6.52E-15 |
| THOP1 | 0.3485618 | 2.67E-16 | 6.54E-15 |
| HAT1 | 0.3485545 | 2.67E-16 | 6.55E-15 |
| NID1 | -0.3485399 | 2.68E-16 | 6.56E-15 |
| BTBD9 | -0.3484283 | 2.74E-16 | 6.70E-15 |
| ARHGEF17 | -0.3484216 | 2.75E-16 | 6.71E-15 |
| MMAB | 0.3483725 | 2.78E-16 | 6.77E-15 |
| WDR62 | 0.3482989 | 2.82E-16 | 6.86E-15 |
| SATB2 | -0.3482564 | 2.84E-16 | 6.92E-15 |
| NOP56 | 0.34821 | 2.87E-16 | 6.97E-15 |
| AMFR | -0.3481923 | 2.88E-16 | 6.99E-15 |
| GLG1 | -0.3480782 | 2.95E-16 | 7.15E-15 |
| TRAF2 | 0.3480611 | 2.96E-16 | 7.17E-15 |
| MTP18 | 0.348033 | 2.98E-16 | 7.20E-15 |
| PKN3 | 0.3478091 | 3.12E-16 | 7.53E-15 |
| TECTA | -0.3477744 | 3.14E-16 | 7.58E-15 |
| ATRIP | 0.3475471 | 3.29E-16 | 7.94E-15 |
| MRPL18 | 0.347509 | 3.32E-16 | 7.99E-15 |
| TSFM | 0.3474335 | 3.37E-16 | 8.11E-15 |
| UBE2H | -0.3472492 | 3.50E-16 | 8.41E-15 |
| NAV1 | -0.3470946 | 3.62E-16 | 8.68E-15 |
| VGLL3 | -0.3469257 | 3.75E-16 | 8.97E-15 |
| RDM1 | 0.346905 | 3.76E-16 | 9.00E-15 |
| PODNL1 | -0.3468552 | 3.80E-16 | 9.07E-15 |
| EMILIN1 | -0.3468532 | 3.80E-16 | 9.07E-15 |
| C13orf34 | 0.3468531 | 3.80E-16 | 9.07E-15 |
| PDCD5 | 0.3465349 | 4.06E-16 | 9.67E-15 |
| EWSR1 | 0.3465061 | 4.09E-16 | 9.72E-15 |
| ZCCHC9 | 0.3463999 | 4.18E-16 | 9.92E-15 |
| C14orf156 | 0.3463978 | 4.18E-16 | 9.92E-15 |
| WNT11 | -0.3463165 | 4.25E-16 | 1.01E-14 |
| MMP16 | -0.3463056 | 4.26E-16 | 1.01E-14 |
| SLC36A1 | -0.3463003 | 4.27E-16 | 1.01E-14 |
| NSL1 | 0.3461746 | 4.38E-16 | 1.03E-14 |
| AXIN2 | -0.3460869 | 4.46E-16 | 1.05E-14 |
| MRPS27 | 0.346036 | 4.50E-16 | 1.06E-14 |
| SCN3B | -0.3459972 | 4.54E-16 | 1.07E-14 |
| SDHB | 0.3459657 | 4.57E-16 | 1.07E-14 |
| AHNAK2 | -0.3458723 | 4.66E-16 | 1.09E-14 |
| PPP2R3A | -0.3458394 | 4.69E-16 | 1.10E-14 |
| HIST1H2AL | 0.3457647 | 4.76E-16 | 1.12E-14 |
| STRA13 | 0.3456565 | 4.87E-16 | 1.14E-14 |
| TBCEL | -0.3456408 | 4.89E-16 | 1.14E-14 |
| SNHG6 | 0.3456375 | 4.89E-16 | 1.14E-14 |
| CCDC77 | 0.3455622 | 4.97E-16 | 1.16E-14 |
| MYO1D | -0.3455591 | 4.97E-16 | 1.16E-14 |
| KDM5B | -0.3455511 | 4.98E-16 | 1.16E-14 |
| ECT2 | 0.3454317 | 5.10E-16 | 1.19E-14 |
| PCDHB4 | -0.3453732 | 5.16E-16 | 1.20E-14 |
| PPP1CC | 0.3453298 | 5.21E-16 | 1.21E-14 |
| MFAP4 | -0.3452586 | 5.29E-16 | 1.22E-14 |
| LRRC15 | -0.3452099 | 5.34E-16 | 1.23E-14 |
| CSE1L | 0.3451995 | 5.35E-16 | 1.24E-14 |
| HNRPDL | 0.3450358 | 5.54E-16 | 1.28E-14 |
| ECM2 | -0.3450077 | 5.57E-16 | 1.28E-14 |
| MFAP1 | 0.345 | 5.58E-16 | 1.28E-14 |
| MYH9 | -0.3449901 | 5.59E-16 | 1.28E-14 |
| C12orf45 | 0.3449034 | 5.69E-16 | 1.31E-14 |
| ATP5F1 | 0.3448896 | 5.70E-16 | 1.31E-14 |
| SCN2B | -0.344886 | 5.71E-16 | 1.31E-14 |
| UTP6 | 0.3447519 | 5.87E-16 | 1.34E-14 |
| PSMA2 | 0.3447069 | 5.92E-16 | 1.35E-14 |
| KCTD10 | -0.3443918 | 6.32E-16 | 1.44E-14 |
| FAM26E | -0.3443201 | 6.41E-16 | 1.46E-14 |
| SEL1L | -0.3442579 | 6.49E-16 | 1.48E-14 |
| GTF3C5 | 0.3442562 | 6.50E-16 | 1.48E-14 |
| TRIO | -0.3441906 | 6.58E-16 | 1.50E-14 |
| PXDN | -0.3441138 | 6.69E-16 | 1.52E-14 |
| MSH6 | 0.3440291 | 6.81E-16 | 1.54E-14 |
| TMEM14B | 0.3439553 | 6.91E-16 | 1.57E-14 |
| RBMX2 | 0.3439205 | 6.96E-16 | 1.57E-14 |
| FAM104B | 0.3439076 | 6.98E-16 | 1.58E-14 |
| PCDHGB6 | -0.3438808 | 7.02E-16 | 1.58E-14 |
| PCDH18 | -0.3438205 | 7.10E-16 | 1.60E-14 |
| C9orf46 | 0.3437674 | 7.18E-16 | 1.62E-14 |
| MYCBP | 0.3437168 | 7.26E-16 | 1.63E-14 |
| PSMB4 | 0.3436926 | 7.29E-16 | 1.64E-14 |
| VCAN | -0.3435501 | 7.51E-16 | 1.69E-14 |
| SALL1 | -0.3435024 | 7.58E-16 | 1.70E-14 |
| SH3RF3 | -0.3434508 | 7.66E-16 | 1.72E-14 |
| CDH11 | -0.3432446 | 7.99E-16 | 1.79E-14 |
| BRIX1 | 0.343202 | 8.06E-16 | 1.80E-14 |
| FAM64A | 0.3431725 | 8.11E-16 | 1.81E-14 |
| EXOSC3 | 0.3430834 | 8.26E-16 | 1.84E-14 |
| SYCE2 | 0.3429839 | 8.43E-16 | 1.88E-14 |
| IDS | -0.3429322 | 8.52E-16 | 1.89E-14 |
| UTP18 | 0.3429299 | 8.52E-16 | 1.89E-14 |
| HTRA3 | -0.342899 | 8.58E-16 | 1.90E-14 |
| PINK1 | -0.3428734 | 8.62E-16 | 1.91E-14 |
| WDFY3 | -0.3427107 | 8.91E-16 | 1.97E-14 |
| TFAP4 | 0.3427055 | 8.92E-16 | 1.97E-14 |
| RPSAP9 | 0.3425547 | 9.20E-16 | 2.03E-14 |
| COQ5 | 0.3424573 | 9.38E-16 | 2.07E-14 |
| DAB2 | -0.3422469 | 9.80E-16 | 2.16E-14 |
| SSRP1 | 0.3421254 | 1.00E-15 | 2.21E-14 |
| MAGEL2 | -0.3421095 | 1.01E-15 | 2.22E-14 |
| DCI | 0.3420281 | 1.02E-15 | 2.25E-14 |
| KIAA0427 | -0.341908 | 1.05E-15 | 2.31E-14 |
| POLA1 | 0.3418568 | 1.06E-15 | 2.33E-14 |
| ARL6IP1 | 0.3418366 | 1.06E-15 | 2.33E-14 |
| BTBD19 | -0.3418105 | 1.07E-15 | 2.34E-14 |
| MRPL23 | 0.3417773 | 1.08E-15 | 2.36E-14 |
| EIF4G3 | -0.3416836 | 1.10E-15 | 2.40E-14 |
| DDX20 | 0.3415278 | 1.13E-15 | 2.47E-14 |
| ANKRD50 | -0.3415191 | 1.14E-15 | 2.48E-14 |
| MAP6 | -0.3414704 | 1.15E-15 | 2.50E-14 |
| C1orf198 | -0.341458 | 1.15E-15 | 2.50E-14 |
| NUDT6 | 0.3413746 | 1.17E-15 | 2.54E-14 |
| NUP107 | 0.3413403 | 1.18E-15 | 2.56E-14 |
| MRPS23 | 0.3413119 | 1.18E-15 | 2.57E-14 |
| DDB2 | 0.3412817 | 1.19E-15 | 2.58E-14 |
| RMI1 | 0.3412779 | 1.19E-15 | 2.58E-14 |
| MAN1A1 | -0.3411085 | 1.23E-15 | 2.67E-14 |
| CAPN5 | -0.3409913 | 1.26E-15 | 2.73E-14 |
| BCS1L | 0.3409591 | 1.27E-15 | 2.74E-14 |
| PPP1R8 | 0.3409331 | 1.28E-15 | 2.76E-14 |
| RRBP1 | -0.3407961 | 1.32E-15 | 2.83E-14 |
| EDNRA | -0.3404541 | 1.41E-15 | 3.03E-14 |
| RHOBTB1 | -0.3403189 | 1.45E-15 | 3.11E-14 |
| DSC1 | -0.3401959 | 1.49E-15 | 3.19E-14 |
| PAPPA | -0.3401698 | 1.49E-15 | 3.20E-14 |
| MRPL17 | 0.3399149 | 1.57E-15 | 3.36E-14 |
| PINX1 | 0.3396891 | 1.65E-15 | 3.52E-14 |
| STXBP5 | -0.3396534 | 1.66E-15 | 3.54E-14 |
| C10orf26 | -0.3395667 | 1.69E-15 | 3.60E-14 |
| GOLGA2 | -0.3393547 | 1.76E-15 | 3.75E-14 |
| PPAPDC3 | -0.3393043 | 1.78E-15 | 3.79E-14 |
| LOC729082 | 0.3391836 | 1.82E-15 | 3.87E-14 |
| C6orf130 | 0.3391434 | 1.84E-15 | 3.90E-14 |
| PSMA5 | 0.3391313 | 1.84E-15 | 3.91E-14 |
| DKC1 | 0.339125 | 1.84E-15 | 3.91E-14 |
| CHRD | -0.339036 | 1.88E-15 | 3.97E-14 |
| C16orf61 | 0.3388941 | 1.93E-15 | 4.08E-14 |
| AKAP13 | -0.3388925 | 1.93E-15 | 4.08E-14 |
| C22orf9 | -0.3388364 | 1.95E-15 | 4.12E-14 |
| METT11D1 | 0.33861 | 2.04E-15 | 4.31E-14 |
| SAAL1 | 0.3383865 | 2.14E-15 | 4.51E-14 |
| TSEN2 | 0.3383145 | 2.17E-15 | 4.57E-14 |
| ATP5O | 0.3383073 | 2.17E-15 | 4.57E-14 |
| NUP54 | 0.338219 | 2.21E-15 | 4.64E-14 |
| KIF13A | -0.338169 | 2.23E-15 | 4.69E-14 |
| PCSK5 | -0.3379563 | 2.33E-15 | 4.89E-14 |
| DUSP12 | 0.3378814 | 2.37E-15 | 4.95E-14 |
| FAM65A | -0.3377817 | 2.41E-15 | 5.05E-14 |
| RPP21 | 0.3376606 | 2.47E-15 | 5.17E-14 |
| DCAF5 | -0.3376377 | 2.48E-15 | 5.19E-14 |
| OLFML2B | -0.3374988 | 2.55E-15 | 5.33E-14 |
| MNT | -0.3373567 | 2.63E-15 | 5.48E-14 |
| SEC14L1 | -0.3370911 | 2.77E-15 | 5.77E-14 |
| TACO1 | 0.3369964 | 2.82E-15 | 5.87E-14 |
| PCDHGA6 | -0.3369149 | 2.87E-15 | 5.96E-14 |
| SEC31A | -0.3369136 | 2.87E-15 | 5.96E-14 |
| COX7A2 | 0.336755 | 2.96E-15 | 6.14E-14 |
| NRP1 | -0.3366055 | 3.05E-15 | 6.32E-14 |
| DDX11 | 0.3365922 | 3.06E-15 | 6.33E-14 |
| PLEKHM2 | -0.3364505 | 3.15E-15 | 6.51E-14 |
| DIAPH3 | 0.3363282 | 3.23E-15 | 6.66E-14 |
| PABPN1 | 0.3363246 | 3.23E-15 | 6.66E-14 |
| GLT8D2 | -0.3361502 | 3.34E-15 | 6.89E-14 |
| MRPS15 | 0.3360622 | 3.40E-15 | 7.00E-14 |
| SCLT1 | 0.3360224 | 3.43E-15 | 7.05E-14 |
| NAA38 | 0.3359132 | 3.51E-15 | 7.20E-14 |
| C5orf34 | 0.3359073 | 3.51E-15 | 7.20E-14 |
| RAB11FIP3 | -0.3358934 | 3.52E-15 | 7.21E-14 |
| HAX1 | 0.3357398 | 3.63E-15 | 7.43E-14 |
| C18orf54 | 0.3356591 | 3.69E-15 | 7.54E-14 |
| INHBB | -0.3354391 | 3.85E-15 | 7.87E-14 |
| MSH5 | 0.3354365 | 3.85E-15 | 7.87E-14 |
| FIGNL1 | 0.3353039 | 3.96E-15 | 8.07E-14 |
| YEATS4 | 0.335292 | 3.97E-15 | 8.08E-14 |
| C9orf142 | 0.3351623 | 4.07E-15 | 8.28E-14 |
| CENPI | 0.3351099 | 4.11E-15 | 8.36E-14 |
| KALRN | -0.3350862 | 4.13E-15 | 8.39E-14 |
| KIAA0232 | -0.3350788 | 4.14E-15 | 8.39E-14 |
| DCHS1 | -0.3349492 | 4.25E-15 | 8.60E-14 |
| TMCO6 | 0.3348537 | 4.33E-15 | 8.76E-14 |
| CDAN1 | 0.3347921 | 4.38E-15 | 8.86E-14 |
| H3F3A | 0.334702 | 4.46E-15 | 9.01E-14 |
| WNK1 | -0.3346562 | 4.50E-15 | 9.08E-14 |
| SETD7 | -0.3345798 | 4.57E-15 | 9.21E-14 |
| SLC18A2 | -0.3345619 | 4.58E-15 | 9.24E-14 |
| MLXIP | -0.3344687 | 4.67E-15 | 9.40E-14 |
| PIN4 | 0.3344578 | 4.68E-15 | 9.41E-14 |
| OLFM2 | -0.3343931 | 4.74E-15 | 9.52E-14 |
| ENPEP | -0.3342771 | 4.85E-15 | 9.73E-14 |
| CMIP | -0.3341424 | 4.98E-15 | 9.98E-14 |
| SRPX2 | -0.3340994 | 5.02E-15 | 1.01E-13 |
| NFYB | 0.3340371 | 5.09E-15 | 1.02E-13 |
| NMU | 0.3339384 | 5.19E-15 | 1.04E-13 |
| FRG1 | 0.3339311 | 5.19E-15 | 1.04E-13 |
| FZD1 | -0.3338256 | 5.30E-15 | 1.06E-13 |
| CHAC2 | 0.3338204 | 5.31E-15 | 1.06E-13 |
| HNRNPA3 | 0.3337961 | 5.33E-15 | 1.06E-13 |
| SNED1 | -0.3337518 | 5.38E-15 | 1.07E-13 |
| WDR26 | -0.3337253 | 5.41E-15 | 1.07E-13 |
| ATAD5 | 0.3336614 | 5.48E-15 | 1.09E-13 |
| BEAN | -0.3336235 | 5.52E-15 | 1.09E-13 |
| HMSD | 0.3336047 | 5.54E-15 | 1.10E-13 |
| UCK2 | 0.3335495 | 5.60E-15 | 1.11E-13 |
| ITGA11 | -0.333259 | 5.93E-15 | 1.17E-13 |
| LMOD1 | -0.3331728 | 6.03E-15 | 1.19E-13 |
| C11orf48 | 0.3330477 | 6.18E-15 | 1.22E-13 |
| HEYL | -0.3330047 | 6.24E-15 | 1.23E-13 |
| MRPL47 | 0.3328793 | 6.39E-15 | 1.26E-13 |
| BTF3 | 0.332695 | 6.63E-15 | 1.30E-13 |
| HNRNPU | 0.3326793 | 6.65E-15 | 1.31E-13 |
| TAF9 | 0.3326716 | 6.66E-15 | 1.31E-13 |
| GUCY1A2 | -0.3326187 | 6.73E-15 | 1.32E-13 |
| HTR1B | -0.3325812 | 6.78E-15 | 1.33E-13 |
| PPA2 | 0.3325773 | 6.78E-15 | 1.33E-13 |
| PPM1G | 0.3325284 | 6.85E-15 | 1.34E-13 |
| ZNF833 | 0.3325243 | 6.85E-15 | 1.34E-13 |
| SORBS1 | -0.332254 | 7.23E-15 | 1.41E-13 |
| HDLBP | -0.3321473 | 7.38E-15 | 1.44E-13 |
| RASGRF2 | -0.3319657 | 7.65E-15 | 1.49E-13 |
| SFRS9 | 0.3319431 | 7.68E-15 | 1.50E-13 |
| NVL | 0.3319145 | 7.73E-15 | 1.50E-13 |
| AGTR1 | -0.3318552 | 7.82E-15 | 1.52E-13 |
| CTGF | -0.3318058 | 7.89E-15 | 1.53E-13 |
| EPDR1 | -0.3317593 | 7.96E-15 | 1.54E-13 |
| CRYAB | -0.3317294 | 8.01E-15 | 1.55E-13 |
| HIST1H1B | 0.331726 | 8.02E-15 | 1.55E-13 |
| GALNT10 | -0.331695 | 8.06E-15 | 1.56E-13 |
| ZEB2 | -0.3315953 | 8.22E-15 | 1.59E-13 |
| MRPS25 | 0.3315815 | 8.25E-15 | 1.59E-13 |
| SEMA3C | -0.3315556 | 8.29E-15 | 1.60E-13 |
| XRCC2 | 0.331379 | 8.58E-15 | 1.65E-13 |
| C1orf156 | 0.331347 | 8.63E-15 | 1.66E-13 |
| PCDHB15 | -0.3313267 | 8.67E-15 | 1.67E-13 |
| DIABLO | 0.3313194 | 8.68E-15 | 1.67E-13 |
| CPEB2 | -0.331319 | 8.68E-15 | 1.67E-13 |
| FSTL1 | -0.3311268 | 9.01E-15 | 1.73E-13 |
| DIP2B | -0.330916 | 9.39E-15 | 1.80E-13 |
| DIXDC1 | -0.3308943 | 9.43E-15 | 1.80E-13 |
| FAM19A5 | -0.3307393 | 9.72E-15 | 1.86E-13 |
| COL14A1 | -0.3306082 | 9.97E-15 | 1.90E-13 |
| RAB11FIP5 | -0.3304645 | 1.03E-14 | 1.96E-13 |
| HSPB3 | -0.3304084 | 1.04E-14 | 1.98E-13 |
| COLEC12 | -0.3304054 | 1.04E-14 | 1.98E-13 |
| CYCS | 0.3303346 | 1.05E-14 | 2.00E-13 |
| RCAN2 | -0.3302194 | 1.08E-14 | 2.05E-13 |
| TFAM | 0.3302088 | 1.08E-14 | 2.05E-13 |
| CDC123 | 0.3301605 | 1.09E-14 | 2.06E-13 |
| TOMM40 | 0.330159 | 1.09E-14 | 2.06E-13 |
| RNF217 | -0.3301069 | 1.10E-14 | 2.08E-13 |
| PLEKHM1 | -0.3300681 | 1.11E-14 | 2.10E-13 |
| TMCC1 | -0.3300043 | 1.12E-14 | 2.12E-13 |
| NFAT5 | -0.3298899 | 1.15E-14 | 2.17E-13 |
| RPL35A | 0.3298854 | 1.15E-14 | 2.17E-13 |
| CTSK | -0.3297151 | 1.19E-14 | 2.24E-13 |
| HNRNPH3 | 0.3296803 | 1.20E-14 | 2.25E-13 |
| SNAPC5 | 0.3296169 | 1.21E-14 | 2.28E-13 |
| DIRAS1 | -0.3295785 | 1.22E-14 | 2.29E-13 |
| PDE7B | -0.3295324 | 1.23E-14 | 2.31E-13 |
| MRPL13 | 0.3294885 | 1.24E-14 | 2.33E-13 |
| SHROOM4 | -0.3293274 | 1.28E-14 | 2.40E-13 |
| NTM | -0.3292907 | 1.29E-14 | 2.41E-13 |
| FN3KRP | 0.3292206 | 1.31E-14 | 2.44E-13 |
| FNDC1 | -0.3290231 | 1.36E-14 | 2.54E-13 |
| SKIL | -0.3290114 | 1.36E-14 | 2.54E-13 |
| RASAL2 | -0.3289848 | 1.37E-14 | 2.55E-13 |
| HECW1 | -0.32886 | 1.40E-14 | 2.61E-13 |
| WDR5 | 0.3287721 | 1.43E-14 | 2.65E-13 |
| FAP | -0.3286587 | 1.46E-14 | 2.71E-13 |
| PEG3 | -0.3284399 | 1.52E-14 | 2.83E-13 |
| PLEKHM3 | -0.3283944 | 1.53E-14 | 2.85E-13 |
| SPOCK1 | -0.3283879 | 1.54E-14 | 2.85E-13 |
| KIAA1644 | -0.3283584 | 1.54E-14 | 2.86E-13 |
| RPL36A | 0.3283358 | 1.55E-14 | 2.87E-13 |
| EVI5 | -0.3281152 | 1.62E-14 | 3.00E-13 |
| USP2 | -0.3280008 | 1.66E-14 | 3.06E-13 |
| CACNA1H | -0.3279657 | 1.67E-14 | 3.08E-13 |
| NARG2 | 0.3279185 | 1.68E-14 | 3.10E-13 |
| C14orf142 | 0.3276783 | 1.76E-14 | 3.25E-13 |
| FTSJ2 | 0.3276743 | 1.76E-14 | 3.25E-13 |
| NDST1 | -0.3276436 | 1.77E-14 | 3.26E-13 |
| NCKAP5L | -0.32748 | 1.83E-14 | 3.36E-13 |
| SHMT1 | 0.3274736 | 1.83E-14 | 3.37E-13 |
| CD36 | -0.327424 | 1.85E-14 | 3.40E-13 |
| OXSM | 0.327402 | 1.86E-14 | 3.41E-13 |
| PFDN2 | 0.3273467 | 1.88E-14 | 3.44E-13 |
| SPSB1 | -0.3272636 | 1.91E-14 | 3.49E-13 |
| FAM128A | 0.3272504 | 1.91E-14 | 3.50E-13 |
| PHLDB1 | -0.3272016 | 1.93E-14 | 3.53E-13 |
| VPS13B | -0.3271766 | 1.94E-14 | 3.54E-13 |
| LIMS1 | -0.3271478 | 1.95E-14 | 3.56E-13 |
| ILKAP | 0.3270414 | 1.99E-14 | 3.63E-13 |
| COX7C | 0.327025 | 2.00E-14 | 3.64E-13 |
| DOK6 | -0.3270214 | 2.00E-14 | 3.64E-13 |
| SNX29 | -0.3269819 | 2.02E-14 | 3.66E-13 |
| MBNL2 | -0.3269599 | 2.02E-14 | 3.67E-13 |
| UBXN11 | 0.3268711 | 2.06E-14 | 3.73E-13 |
| KIF16B | -0.3268526 | 2.07E-14 | 3.74E-13 |
| PSTK | 0.3268352 | 2.07E-14 | 3.75E-13 |
| PMEPA1 | -0.326679 | 2.14E-14 | 3.86E-13 |
| SNRPD3 | 0.3265949 | 2.17E-14 | 3.92E-13 |
| FRMD6 | -0.3265461 | 2.19E-14 | 3.96E-13 |
| FUS | 0.3264346 | 2.24E-14 | 4.04E-13 |
| LOC341056 | 0.3264186 | 2.25E-14 | 4.05E-13 |
| KCTD21 | -0.3263725 | 2.27E-14 | 4.08E-13 |
| ASAP3 | -0.3263238 | 2.29E-14 | 4.11E-13 |
| NIF3L1 | 0.3262304 | 2.33E-14 | 4.18E-13 |
| RPSAP58 | 0.3260846 | 2.39E-14 | 4.30E-13 |
| SKI | -0.3260205 | 2.42E-14 | 4.35E-13 |
| ATP10A | -0.3260135 | 2.43E-14 | 4.35E-13 |
| ATPBD4 | 0.3259855 | 2.44E-14 | 4.37E-13 |
| HNRNPM | 0.3259795 | 2.44E-14 | 4.37E-13 |
| TATDN3 | 0.3259417 | 2.46E-14 | 4.40E-13 |
| DIP2C | -0.3258088 | 2.52E-14 | 4.51E-13 |
| PLXNC1 | -0.325747 | 2.55E-14 | 4.55E-13 |
| MORF4L1 | 0.3257469 | 2.55E-14 | 4.55E-13 |
| AK5 | -0.3256398 | 2.61E-14 | 4.65E-13 |
| TTYH3 | -0.3256298 | 2.61E-14 | 4.65E-13 |
| PDGFRA | -0.3256037 | 2.63E-14 | 4.67E-13 |
| SURF2 | 0.3255625 | 2.65E-14 | 4.70E-13 |
| PLEC | -0.3255602 | 2.65E-14 | 4.70E-13 |
| ADAM12 | -0.3255546 | 2.65E-14 | 4.70E-13 |
| CYBRD1 | -0.3255277 | 2.66E-14 | 4.72E-13 |
| PODN | -0.3255236 | 2.67E-14 | 4.72E-13 |
| PTPN21 | -0.3255133 | 2.67E-14 | 4.73E-13 |
| LOC728758 | 0.3254894 | 2.68E-14 | 4.74E-13 |
| ZNF385D | -0.3254504 | 2.70E-14 | 4.77E-13 |
| HSPA12A | -0.3253742 | 2.74E-14 | 4.84E-13 |
| ACOT4 | 0.3253155 | 2.77E-14 | 4.89E-13 |
| C1orf151 | 0.3252437 | 2.81E-14 | 4.95E-13 |
| GFPT1 | -0.3251772 | 2.85E-14 | 5.01E-13 |
| DSEL | -0.324824 | 3.05E-14 | 5.36E-13 |
| ANO5 | -0.324807 | 3.06E-14 | 5.37E-13 |
| ERI1 | 0.324773 | 3.08E-14 | 5.40E-13 |
| C7orf44 | 0.3247671 | 3.08E-14 | 5.40E-13 |
| ARHGAP32 | -0.3247619 | 3.08E-14 | 5.40E-13 |
| NOX4 | -0.3246246 | 3.17E-14 | 5.54E-13 |
| CDR1 | -0.3245727 | 3.20E-14 | 5.59E-13 |
| FAM180A | -0.3244581 | 3.27E-14 | 5.71E-13 |
| CLIP4 | -0.3242418 | 3.41E-14 | 5.95E-13 |
| ATG2B | -0.3241493 | 3.47E-14 | 6.05E-13 |
| C3orf26 | 0.3241389 | 3.47E-14 | 6.05E-13 |
| MTF2 | 0.3241124 | 3.49E-14 | 6.08E-13 |
| ZCCHC10 | 0.324014 | 3.56E-14 | 6.19E-13 |
| TSPYL4 | -0.3239761 | 3.58E-14 | 6.23E-13 |
| RPS24 | 0.3239257 | 3.62E-14 | 6.28E-13 |
| BNIP1 | 0.323911 | 3.63E-14 | 6.30E-13 |
| ZRANB2 | 0.3238027 | 3.70E-14 | 6.42E-13 |
| TRIP12 | -0.3237744 | 3.72E-14 | 6.45E-13 |
| NDUFA6 | 0.3235452 | 3.89E-14 | 6.73E-13 |
| PDE2A | -0.3233925 | 4.00E-14 | 6.93E-13 |
| HNRNPR | 0.3233754 | 4.02E-14 | 6.94E-13 |
| ZDHHC6 | 0.3232504 | 4.11E-14 | 7.10E-13 |
| C8orf59 | 0.323189 | 4.16E-14 | 7.18E-13 |
| DYRK2 | -0.3231736 | 4.17E-14 | 7.19E-13 |
| GREM2 | -0.3231636 | 4.18E-14 | 7.20E-13 |
| PDGFB | -0.3231054 | 4.23E-14 | 7.28E-13 |
| UBE2D2 | 0.3230305 | 4.29E-14 | 7.37E-13 |
| PHB | 0.3229802 | 4.33E-14 | 7.44E-13 |
| TMEM131 | -0.322968 | 4.34E-14 | 7.45E-13 |
| RAB30 | -0.322962 | 4.35E-14 | 7.45E-13 |
| HIPK2 | -0.3226512 | 4.61E-14 | 7.90E-13 |
| PLA2R1 | -0.3225839 | 4.67E-14 | 7.99E-13 |
| ABCA1 | -0.3225705 | 4.68E-14 | 8.01E-13 |
| ANAPC5 | 0.3223781 | 4.85E-14 | 8.30E-13 |
| SCN7A | -0.3222824 | 4.94E-14 | 8.44E-13 |
| NOTCH3 | -0.3222754 | 4.95E-14 | 8.44E-13 |
| EXOSC5 | 0.3221744 | 5.05E-14 | 8.60E-13 |
| SLC38A7 | -0.3221389 | 5.08E-14 | 8.65E-13 |
| SNHG12 | 0.3220564 | 5.16E-14 | 8.78E-13 |
| TSEN54 | 0.3219486 | 5.27E-14 | 8.95E-13 |
| MRPL51 | 0.3219141 | 5.30E-14 | 9.00E-13 |
| HECA | -0.3218267 | 5.39E-14 | 9.15E-13 |
| LIPK | -0.3218094 | 5.41E-14 | 9.17E-13 |
| C10orf78 | 0.3217938 | 5.42E-14 | 9.19E-13 |
| WHSC1 | 0.3217651 | 5.45E-14 | 9.23E-13 |
| PARK2 | -0.3216946 | 5.52E-14 | 9.34E-13 |
| SEC24D | -0.3216717 | 5.55E-14 | 9.38E-13 |
| TARBP2 | 0.3216206 | 5.60E-14 | 9.46E-13 |
| LYST | -0.321467 | 5.77E-14 | 9.73E-13 |
| RPS21 | 0.3214556 | 5.78E-14 | 9.74E-13 |
| C18orf1 | -0.3214342 | 5.80E-14 | 9.77E-13 |
| TMEM194A | 0.3214264 | 5.81E-14 | 9.78E-13 |
| RHOQ | -0.3213367 | 5.91E-14 | 9.94E-13 |
| CD248 | -0.3213161 | 5.93E-14 | 9.97E-13 |
| PROS1 | -0.3213068 | 5.94E-14 | 9.98E-13 |
| HMGB3 | 0.3212375 | 6.02E-14 | 1.01E-12 |
| SLC35E1 | -0.3210487 | 6.24E-14 | 1.05E-12 |
| STIM1 | -0.3210309 | 6.26E-14 | 1.05E-12 |
| ATP7A | -0.3208329 | 6.50E-14 | 1.09E-12 |
| FRY | -0.3208285 | 6.50E-14 | 1.09E-12 |
| MRPS18B | 0.3208007 | 6.54E-14 | 1.09E-12 |
| GABPB1 | 0.3207711 | 6.57E-14 | 1.10E-12 |
| XRCC1 | 0.3207098 | 6.65E-14 | 1.11E-12 |
| ABCA6 | -0.3206661 | 6.71E-14 | 1.12E-12 |
| MED30 | 0.3206481 | 6.73E-14 | 1.12E-12 |
| BANF1 | 0.3206416 | 6.74E-14 | 1.12E-12 |
| MRP63 | 0.3206227 | 6.76E-14 | 1.12E-12 |
| CALM2 | 0.3205996 | 6.79E-14 | 1.13E-12 |
| CDK5RAP1 | 0.3205776 | 6.82E-14 | 1.13E-12 |
| MRPS5 | 0.3205617 | 6.84E-14 | 1.13E-12 |
| SULF1 | -0.3204901 | 6.93E-14 | 1.15E-12 |
| CRY2 | -0.3204843 | 6.94E-14 | 1.15E-12 |
| WEE1 | 0.3204169 | 7.03E-14 | 1.16E-12 |
| UBR3 | -0.3204159 | 7.03E-14 | 1.16E-12 |
| CYTH3 | -0.320384 | 7.07E-14 | 1.17E-12 |
| NDUFA7 | 0.3202475 | 7.26E-14 | 1.20E-12 |
| SH3D19 | -0.3202369 | 7.27E-14 | 1.20E-12 |
| CILP | -0.3202288 | 7.28E-14 | 1.20E-12 |
| PPP2R2C | -0.3201284 | 7.42E-14 | 1.22E-12 |
| FGD5 | -0.3198653 | 7.79E-14 | 1.28E-12 |
| MCM4 | 0.3198004 | 7.89E-14 | 1.30E-12 |
| MGLL | -0.3197872 | 7.91E-14 | 1.30E-12 |
| MRPS9 | 0.3197516 | 7.96E-14 | 1.31E-12 |
| PMP22 | -0.3197143 | 8.02E-14 | 1.31E-12 |
| KIF14 | 0.3195346 | 8.29E-14 | 1.36E-12 |
| NEB | -0.3194087 | 8.49E-14 | 1.39E-12 |
| ADAMTS15 | -0.3192791 | 8.70E-14 | 1.42E-12 |
| ATP9A | -0.3191904 | 8.85E-14 | 1.45E-12 |
| ZBTB38 | -0.3191507 | 8.91E-14 | 1.45E-12 |
| OGG1 | 0.3191117 | 8.98E-14 | 1.46E-12 |
| ABCA9 | -0.3190108 | 9.15E-14 | 1.49E-12 |
| PTPRG | -0.3188928 | 9.35E-14 | 1.52E-12 |
| EFEMP2 | -0.3188742 | 9.38E-14 | 1.53E-12 |
| KANK4 | -0.318862 | 9.41E-14 | 1.53E-12 |
| LMNB2 | 0.3188092 | 9.50E-14 | 1.54E-12 |
| TBC1D20 | -0.3186926 | 9.71E-14 | 1.58E-12 |
| LOC643387 | 0.3186822 | 9.73E-14 | 1.58E-12 |
| C3orf75 | 0.3186389 | 9.81E-14 | 1.59E-12 |
| ASPN | -0.318534 | 1.00E-13 | 1.62E-12 |
| DCN | -0.3184247 | 1.02E-13 | 1.65E-12 |
| SUPV3L1 | 0.3184104 | 1.02E-13 | 1.65E-12 |
| TIMM8A | 0.3183232 | 1.04E-13 | 1.68E-12 |
| SLC4A5 | 0.3183193 | 1.04E-13 | 1.68E-12 |
| ADAM33 | -0.3182343 | 1.06E-13 | 1.71E-12 |
| NRK | -0.3182005 | 1.06E-13 | 1.72E-12 |
| TIMM17B | 0.3181867 | 1.07E-13 | 1.72E-12 |
| PCDHGA4 | -0.3181435 | 1.08E-13 | 1.73E-12 |
| TM9SF2 | -0.3179573 | 1.11E-13 | 1.79E-12 |
| CALD1 | -0.3179222 | 1.12E-13 | 1.80E-12 |
| RPL39 | 0.3178961 | 1.13E-13 | 1.81E-12 |
| ZNF727 | -0.3178129 | 1.14E-13 | 1.83E-12 |
| TUBB | 0.3177722 | 1.15E-13 | 1.85E-12 |
| ZNF608 | -0.317768 | 1.15E-13 | 1.85E-12 |
| C18orf21 | 0.3177636 | 1.15E-13 | 1.85E-12 |
| KLHL3 | -0.3177166 | 1.16E-13 | 1.86E-12 |
| RPL35 | 0.3177119 | 1.17E-13 | 1.86E-12 |
| C10orf119 | 0.3174992 | 1.21E-13 | 1.94E-12 |
| NOP10 | 0.3174642 | 1.22E-13 | 1.95E-12 |
| MAP1B | -0.3173865 | 1.24E-13 | 1.97E-12 |
| INTS7 | 0.3173376 | 1.25E-13 | 1.99E-12 |
| C19orf43 | 0.3172672 | 1.27E-13 | 2.01E-12 |
| STOML2 | 0.317114 | 1.30E-13 | 2.07E-12 |
| NAP1L4 | 0.317041 | 1.32E-13 | 2.10E-12 |
| F2RL2 | -0.3168614 | 1.37E-13 | 2.17E-12 |
| RPS3A | 0.3168162 | 1.38E-13 | 2.18E-12 |
| USP47 | -0.3166361 | 1.42E-13 | 2.25E-12 |
| C6orf125 | 0.3166352 | 1.42E-13 | 2.25E-12 |
| C13orf33 | -0.3164777 | 1.47E-13 | 2.32E-12 |
| PHB2 | 0.3162743 | 1.52E-13 | 2.41E-12 |
| PPIA | 0.3162676 | 1.52E-13 | 2.41E-12 |
| PSMG2 | 0.3162282 | 1.54E-13 | 2.42E-12 |
| RPS27L | 0.3160654 | 1.58E-13 | 2.50E-12 |
| PDZD11 | 0.3159946 | 1.60E-13 | 2.53E-12 |
| C16orf62 | -0.3159838 | 1.61E-13 | 2.53E-12 |
| GOLGA2B | 0.3159071 | 1.63E-13 | 2.56E-12 |
| DOCK9 | -0.3158115 | 1.66E-13 | 2.61E-12 |
| ZNF326 | 0.315797 | 1.66E-13 | 2.61E-12 |
| ATP5J | 0.3157438 | 1.68E-13 | 2.64E-12 |
| UQCRQ | 0.3156575 | 1.71E-13 | 2.68E-12 |
| RPS6 | 0.3155907 | 1.73E-13 | 2.71E-12 |
| UXT | 0.3154022 | 1.79E-13 | 2.80E-12 |
| AAAS | 0.3153958 | 1.79E-13 | 2.80E-12 |
| RPS14 | 0.3153253 | 1.81E-13 | 2.84E-12 |
| TBC1D14 | -0.3152915 | 1.83E-13 | 2.85E-12 |
| MYO18A | -0.315274 | 1.83E-13 | 2.86E-12 |
| MRPL27 | 0.3152326 | 1.84E-13 | 2.88E-12 |
| NEGR1 | -0.3151864 | 1.86E-13 | 2.90E-12 |
| RFESD | 0.3150591 | 1.90E-13 | 2.97E-12 |
| GPR157 | -0.3149884 | 1.93E-13 | 3.00E-12 |
| SLC25A3 | 0.3149344 | 1.95E-13 | 3.03E-12 |
| KIAA0556 | -0.314867 | 1.97E-13 | 3.07E-12 |
| TAOK1 | -0.3147867 | 2.00E-13 | 3.11E-12 |
| ACOX1 | -0.3147192 | 2.03E-13 | 3.15E-12 |
| ABCC9 | -0.3146861 | 2.04E-13 | 3.16E-12 |
| HDAC8 | 0.3146578 | 2.05E-13 | 3.18E-12 |
| PFDN6 | 0.3146534 | 2.05E-13 | 3.18E-12 |
| PAMR1 | -0.3144813 | 2.12E-13 | 3.28E-12 |
| RAE1 | 0.3144561 | 2.13E-13 | 3.29E-12 |
| DACT3 | -0.3143766 | 2.16E-13 | 3.34E-12 |
| FMOD | -0.314375 | 2.16E-13 | 3.34E-12 |
| ATP5G2 | 0.3143386 | 2.17E-13 | 3.36E-12 |
| SNX22 | 0.3142223 | 2.22E-13 | 3.43E-12 |
| SSNA1 | 0.3141552 | 2.25E-13 | 3.47E-12 |
| P4HA3 | -0.314106 | 2.27E-13 | 3.49E-12 |
| RFT1 | 0.3140469 | 2.29E-13 | 3.53E-12 |
| PCDHGA1 | -0.3140441 | 2.30E-13 | 3.53E-12 |
| MYOM3 | -0.3139131 | 2.35E-13 | 3.61E-12 |
| SCML1 | 0.3139038 | 2.36E-13 | 3.61E-12 |
| NDUFA1 | 0.3138174 | 2.39E-13 | 3.67E-12 |
| NXT1 | 0.313808 | 2.40E-13 | 3.67E-12 |
| NSA2 | 0.313787 | 2.41E-13 | 3.68E-12 |
| ULK1 | -0.3137627 | 2.42E-13 | 3.70E-12 |
| XPO1 | 0.3135552 | 2.51E-13 | 3.84E-12 |
| RPL32 | 0.3133861 | 2.59E-13 | 3.96E-12 |
| IQSEC1 | -0.3133388 | 2.61E-13 | 3.99E-12 |
| UNC5B | -0.3133327 | 2.62E-13 | 3.99E-12 |
| C17orf75 | 0.3132752 | 2.64E-13 | 4.03E-12 |
| RAPGEF2 | -0.3131848 | 2.69E-13 | 4.09E-12 |
| PSMB3 | 0.3131749 | 2.69E-13 | 4.10E-12 |
| SLC1A7 | -0.3131654 | 2.70E-13 | 4.10E-12 |
| YBX1 | 0.3131384 | 2.71E-13 | 4.12E-12 |
| TIMM9 | 0.3130814 | 2.74E-13 | 4.16E-12 |
| NDUFB1 | 0.3129241 | 2.82E-13 | 4.28E-12 |
| FAT4 | -0.3128727 | 2.85E-13 | 4.31E-12 |
| HTT | -0.3128635 | 2.85E-13 | 4.32E-12 |
| CDK15 | -0.3128227 | 2.87E-13 | 4.35E-12 |
| CSDC2 | -0.3127055 | 2.93E-13 | 4.44E-12 |
| MMP14 | -0.3126885 | 2.94E-13 | 4.45E-12 |
| RSPH3 | -0.312645 | 2.97E-13 | 4.48E-12 |
| PRPF3 | 0.3126394 | 2.97E-13 | 4.48E-12 |
| LSM7 | 0.3126291 | 2.97E-13 | 4.49E-12 |
| FBLN5 | -0.3124111 | 3.10E-13 | 4.66E-12 |
| ATP10D | -0.3122604 | 3.18E-13 | 4.79E-12 |
| NPM3 | 0.3121774 | 3.23E-13 | 4.86E-12 |
| DTX1 | -0.312093 | 3.28E-13 | 4.93E-12 |
| CEP72 | 0.3120319 | 3.32E-13 | 4.98E-12 |
| PPA1 | 0.3120235 | 3.32E-13 | 4.99E-12 |
| MMP1 | -0.3119959 | 3.34E-13 | 5.01E-12 |
| FAM168A | -0.3119648 | 3.36E-13 | 5.03E-12 |
| CHPF | -0.3119584 | 3.36E-13 | 5.04E-12 |
| DGKI | -0.3118909 | 3.40E-13 | 5.09E-12 |
| RPL23A | 0.3118654 | 3.42E-13 | 5.11E-12 |
| ANP32C | 0.3117084 | 3.52E-13 | 5.26E-12 |
| RPS27A | 0.3114781 | 3.67E-13 | 5.48E-12 |
| CCDC51 | 0.3114491 | 3.69E-13 | 5.50E-12 |
| ASAM | -0.3114307 | 3.70E-13 | 5.52E-12 |
| C10orf118 | -0.3114161 | 3.71E-13 | 5.53E-12 |
| LPPR4 | -0.3112442 | 3.83E-13 | 5.70E-12 |
| RPSA | 0.3111575 | 3.89E-13 | 5.79E-12 |
| RPS23 | 0.3109894 | 4.01E-13 | 5.96E-12 |
| THOC6 | 0.310977 | 4.02E-13 | 5.97E-12 |
| EXOSC1 | 0.3109256 | 4.06E-13 | 6.02E-12 |
| SPRY1 | -0.3108721 | 4.10E-13 | 6.08E-12 |
| LOC254559 | 0.3107937 | 4.15E-13 | 6.16E-12 |
| KCNA6 | -0.3105398 | 4.35E-13 | 6.44E-12 |
| USP32 | -0.3104741 | 4.40E-13 | 6.52E-12 |
| KIAA0247 | -0.3104646 | 4.41E-13 | 6.52E-12 |
| CCDC45 | 0.3103676 | 4.49E-13 | 6.63E-12 |
| MAP1LC3B2 | -0.3103255 | 4.52E-13 | 6.68E-12 |
| FTO | -0.3102633 | 4.57E-13 | 6.75E-12 |
| OLFML1 | -0.3102522 | 4.58E-13 | 6.76E-12 |
| ANPEP | -0.3102259 | 4.60E-13 | 6.79E-12 |
| GNG5 | 0.3101314 | 4.68E-13 | 6.90E-12 |
| C8orf38 | 0.3100869 | 4.72E-13 | 6.95E-12 |
| RTN4IP1 | 0.3100744 | 4.73E-13 | 6.96E-12 |
| STK32B | -0.3100296 | 4.77E-13 | 7.01E-12 |
| OR2B6 | 0.3099528 | 4.84E-13 | 7.10E-12 |
| CLTC | -0.3098908 | 4.89E-13 | 7.18E-12 |
| PREX2 | -0.3096756 | 5.09E-13 | 7.46E-12 |
| CD93 | -0.309659 | 5.10E-13 | 7.47E-12 |
| E2F8 | 0.3095088 | 5.24E-13 | 7.67E-12 |
| SLC37A2 | -0.3094373 | 5.31E-13 | 7.77E-12 |
| ELMOD1 | -0.3090535 | 5.69E-13 | 8.32E-12 |
| C15orf61 | 0.309016 | 5.73E-13 | 8.37E-12 |
| POC5 | 0.3089745 | 5.77E-13 | 8.43E-12 |
| GBF1 | -0.3089369 | 5.81E-13 | 8.48E-12 |
| CCDC80 | -0.3089104 | 5.84E-13 | 8.51E-12 |
| VWF | -0.3088855 | 5.86E-13 | 8.54E-12 |
| RPUSD3 | 0.3087259 | 6.03E-13 | 8.79E-12 |
| POLR2G | 0.3086727 | 6.09E-13 | 8.86E-12 |
| SERINC5 | -0.3085731 | 6.20E-13 | 9.01E-12 |
| MMP13 | -0.3085707 | 6.21E-13 | 9.01E-12 |
| RPP38 | 0.3083717 | 6.43E-13 | 9.34E-12 |
| LOC339674 | 0.3082702 | 6.55E-13 | 9.50E-12 |
| GOT1 | 0.3082457 | 6.58E-13 | 9.54E-12 |
| PLEKHJ1 | 0.3082082 | 6.62E-13 | 9.59E-12 |
| NHSL2 | -0.30811 | 6.74E-13 | 9.76E-12 |
| SLC41A2 | -0.3081001 | 6.75E-13 | 9.77E-12 |
| ARHGAP31 | -0.3080907 | 6.76E-13 | 9.78E-12 |
| MICAL2 | -0.3080702 | 6.79E-13 | 9.81E-12 |
| EHD3 | -0.3079506 | 6.94E-13 | 1.00E-11 |
| ENAH | -0.3077801 | 7.15E-13 | 1.03E-11 |
| FAM81A | 0.307758 | 7.18E-13 | 1.03E-11 |
| EXOSC7 | 0.3076938 | 7.26E-13 | 1.05E-11 |
| LAMC3 | -0.3076447 | 7.33E-13 | 1.05E-11 |
| CIB1 | 0.3076041 | 7.38E-13 | 1.06E-11 |
| USMG5 | 0.3075719 | 7.42E-13 | 1.07E-11 |
| SLC35F5 | -0.3075481 | 7.45E-13 | 1.07E-11 |
| INO80E | 0.307543 | 7.46E-13 | 1.07E-11 |
| DYNC1LI2 | -0.3074983 | 7.52E-13 | 1.08E-11 |
| ARMCX1 | -0.3074722 | 7.56E-13 | 1.08E-11 |
| LOC399815 | 0.3074284 | 7.62E-13 | 1.09E-11 |
| NUDT8 | 0.3073281 | 7.75E-13 | 1.11E-11 |
| CORIN | -0.3072289 | 7.89E-13 | 1.13E-11 |
| NAP1L3 | -0.3071558 | 8.00E-13 | 1.14E-11 |
| GLIS1 | -0.3069182 | 8.34E-13 | 1.19E-11 |
| SUCLG1 | 0.3069145 | 8.35E-13 | 1.19E-11 |
| OSGEP | 0.3068966 | 8.38E-13 | 1.19E-11 |
| PARP16 | 0.3068502 | 8.45E-13 | 1.20E-11 |
| TTC32 | 0.3068167 | 8.50E-13 | 1.21E-11 |
| RFTN2 | -0.306785 | 8.54E-13 | 1.22E-11 |
| RBM14 | 0.3067658 | 8.57E-13 | 1.22E-11 |
| HIST1H3B | 0.3065899 | 8.85E-13 | 1.26E-11 |
| C17orf79 | 0.3065741 | 8.87E-13 | 1.26E-11 |
| NBEAL1 | -0.3065322 | 8.94E-13 | 1.27E-11 |
| RBM34 | 0.3064846 | 9.02E-13 | 1.28E-11 |
| INS-IGF2 | -0.3063798 | 9.19E-13 | 1.30E-11 |
| CATSPER2 | 0.3061975 | 9.49E-13 | 1.34E-11 |
| ANAPC16 | 0.3061926 | 9.50E-13 | 1.34E-11 |
| FXN | 0.3061611 | 9.55E-13 | 1.35E-11 |
| PCDHGA9 | -0.3060466 | 9.75E-13 | 1.38E-11 |
| ORC5L | 0.3060246 | 9.79E-13 | 1.38E-11 |
| INSR | -0.3059999 | 9.83E-13 | 1.39E-11 |
| C17orf58 | 0.3059468 | 9.92E-13 | 1.40E-11 |
| MRPL40 | 0.3059382 | 9.94E-13 | 1.40E-11 |
| C16orf72 | -0.3057465 | 1.03E-12 | 1.45E-11 |
| PTCD3 | 0.3056945 | 1.04E-12 | 1.46E-11 |
| C1QTNF7 | -0.3056803 | 1.04E-12 | 1.46E-11 |
| C12orf73 | 0.3056141 | 1.05E-12 | 1.48E-11 |
| TIMM16 | 0.3055715 | 1.06E-12 | 1.49E-11 |
| DBC1 | -0.3055008 | 1.07E-12 | 1.51E-11 |
| EPHA4 | -0.3054783 | 1.08E-12 | 1.51E-11 |
| CUL7 | -0.3054331 | 1.09E-12 | 1.52E-11 |
| GAR1 | 0.3053902 | 1.10E-12 | 1.53E-11 |
| MRPS2 | 0.3053827 | 1.10E-12 | 1.53E-11 |
| RNF138 | 0.3052905 | 1.11E-12 | 1.56E-11 |
| MAP2 | -0.3052212 | 1.13E-12 | 1.58E-11 |
| RPP25 | 0.3052178 | 1.13E-12 | 1.58E-11 |
| SEMA6B | -0.3051767 | 1.14E-12 | 1.59E-11 |
| EIF2B1 | 0.3051564 | 1.14E-12 | 1.59E-11 |
| ALPK3 | -0.3050733 | 1.16E-12 | 1.61E-11 |
| VWA5A | -0.3049705 | 1.18E-12 | 1.64E-11 |
| ABR | -0.3048573 | 1.20E-12 | 1.68E-11 |
| AMOTL1 | -0.3048463 | 1.21E-12 | 1.68E-11 |
| H1FX | 0.3048434 | 1.21E-12 | 1.68E-11 |
| AAGAB | 0.3048257 | 1.21E-12 | 1.68E-11 |
| ADAMTS14 | -0.304736 | 1.23E-12 | 1.71E-11 |
| POU5F1 | 0.3046706 | 1.24E-12 | 1.73E-11 |
| NDUFS4 | 0.3045735 | 1.27E-12 | 1.75E-11 |
| RASA3 | -0.3045625 | 1.27E-12 | 1.76E-11 |
| SLIT3 | -0.3045469 | 1.27E-12 | 1.76E-11 |
| FN1 | -0.3044882 | 1.29E-12 | 1.78E-11 |
| SAMD4B | -0.304448 | 1.29E-12 | 1.79E-11 |
| ADAMTS16 | -0.3044389 | 1.30E-12 | 1.79E-11 |
| VPS41 | -0.3044369 | 1.30E-12 | 1.79E-11 |
| MAP3K2 | -0.3044303 | 1.30E-12 | 1.79E-11 |
| ILF2 | 0.3043519 | 1.32E-12 | 1.81E-11 |
| NUDT2 | 0.3043397 | 1.32E-12 | 1.82E-11 |
| C12orf24 | 0.3043201 | 1.32E-12 | 1.82E-11 |
| CDH15 | -0.3043166 | 1.33E-12 | 1.82E-11 |
| HIC1 | -0.3041808 | 1.36E-12 | 1.87E-11 |
| ABL2 | -0.3040995 | 1.38E-12 | 1.89E-11 |
| SPG21 | 0.3040713 | 1.38E-12 | 1.90E-11 |
| AIFM1 | 0.3040456 | 1.39E-12 | 1.91E-11 |
| CCT8 | 0.3040401 | 1.39E-12 | 1.91E-11 |
| FAM120B | -0.3040394 | 1.39E-12 | 1.91E-11 |
| JAGN1 | 0.3038908 | 1.43E-12 | 1.96E-11 |
| CYC1 | 0.3038403 | 1.44E-12 | 1.97E-11 |
| AFF4 | -0.3038394 | 1.44E-12 | 1.97E-11 |
| RNPS1 | 0.3037997 | 1.45E-12 | 1.98E-11 |
| CPZ | -0.303754 | 1.46E-12 | 2.00E-11 |
| FAM83D | 0.303702 | 1.48E-12 | 2.01E-11 |
| PALM | -0.3036981 | 1.48E-12 | 2.01E-11 |
| GPR1 | -0.3036976 | 1.48E-12 | 2.01E-11 |
| PTPRM | -0.3035098 | 1.53E-12 | 2.08E-11 |
| FDPS | 0.3035089 | 1.53E-12 | 2.08E-11 |
| TIMM10 | 0.303473 | 1.54E-12 | 2.09E-11 |
| KERA | -0.3034234 | 1.55E-12 | 2.11E-11 |
| RBMS3 | -0.3033914 | 1.56E-12 | 2.12E-11 |
| ARL6IP4 | 0.3033375 | 1.58E-12 | 2.14E-11 |
| INHBA | -0.3032408 | 1.60E-12 | 2.17E-11 |
| RAD9A | 0.3032399 | 1.60E-12 | 2.17E-11 |
| PSMG3 | 0.3031275 | 1.63E-12 | 2.21E-11 |
| PAFAH1B2 | -0.3030088 | 1.67E-12 | 2.26E-11 |
| CNN1 | -0.3029544 | 1.68E-12 | 2.28E-11 |
| EMD | 0.3029388 | 1.69E-12 | 2.28E-11 |
| SMUG1 | 0.3028855 | 1.71E-12 | 2.30E-11 |
| FAM136A | 0.3028838 | 1.71E-12 | 2.30E-11 |
| ZNF462 | -0.3028667 | 1.71E-12 | 2.31E-11 |
| PTPRK | -0.3028046 | 1.73E-12 | 2.33E-11 |
| MESDC2 | 0.3026394 | 1.78E-12 | 2.40E-11 |
| KIAA0754 | -0.3026313 | 1.78E-12 | 2.40E-11 |
| ASXL3 | -0.3025893 | 1.80E-12 | 2.42E-11 |
| AVPR1A | -0.3025452 | 1.81E-12 | 2.43E-11 |
| APBB2 | -0.3024769 | 1.83E-12 | 2.46E-11 |
| CALU | -0.3024597 | 1.84E-12 | 2.47E-11 |
| CSTF2 | 0.3022794 | 1.90E-12 | 2.55E-11 |
| RUSC2 | -0.302242 | 1.91E-12 | 2.56E-11 |
| POSTN | -0.302146 | 1.94E-12 | 2.60E-11 |
| SCAND2 | 0.3020791 | 1.97E-12 | 2.63E-11 |
| DOK5 | -0.3020194 | 1.99E-12 | 2.66E-11 |
| ZZEF1 | -0.3020096 | 1.99E-12 | 2.66E-11 |
| RPL5 | 0.3020017 | 1.99E-12 | 2.66E-11 |
| MMP3 | -0.3018192 | 2.06E-12 | 2.75E-11 |
| WDR85 | 0.3018049 | 2.06E-12 | 2.75E-11 |
| TIMM44 | 0.3016159 | 2.13E-12 | 2.84E-11 |
| NDUFB11 | 0.3016031 | 2.14E-12 | 2.85E-11 |
| NPR2 | -0.3015513 | 2.16E-12 | 2.87E-11 |
| PPP5C | 0.3015142 | 2.17E-12 | 2.89E-11 |
| BPIL2 | -0.3014851 | 2.18E-12 | 2.90E-11 |
| PCOLCE | -0.3014194 | 2.21E-12 | 2.93E-11 |
| STARD13 | -0.3013812 | 2.22E-12 | 2.95E-11 |
| VMA21 | 0.3012877 | 2.26E-12 | 3.00E-11 |
| MAP4 | -0.3012596 | 2.27E-12 | 3.01E-11 |
| MRPS24 | 0.3011899 | 2.30E-12 | 3.04E-11 |
| ANK3 | -0.3010911 | 2.34E-12 | 3.09E-11 |
| SMAD9 | -0.3010243 | 2.36E-12 | 3.13E-11 |
| WISP1 | -0.3009843 | 2.38E-12 | 3.15E-11 |
| POLR2D | 0.3009358 | 2.40E-12 | 3.17E-11 |
| PSMG4 | 0.3008359 | 2.44E-12 | 3.23E-11 |
| SVEP1 | -0.3007105 | 2.50E-12 | 3.30E-11 |
| HEATR5A | -0.3005855 | 2.55E-12 | 3.37E-11 |
| SCARA5 | -0.3004946 | 2.59E-12 | 3.42E-11 |
| ISM1 | -0.3004916 | 2.59E-12 | 3.42E-11 |
| SLITRK4 | -0.3004864 | 2.60E-12 | 3.42E-11 |
| RPL17 | 0.3004076 | 2.63E-12 | 3.46E-11 |
| A2M | -0.3003712 | 2.65E-12 | 3.48E-11 |
| NAA10 | 0.3003584 | 2.65E-12 | 3.49E-11 |
| NOP16 | 0.3003347 | 2.67E-12 | 3.50E-11 |
| AQP1 | -0.300278 | 2.69E-12 | 3.53E-11 |
| MME | -0.3002727 | 2.69E-12 | 3.53E-11 |
| RBM9 | -0.3000198 | 2.82E-12 | 3.69E-11 |
| RGS11 | -0.2999578 | 2.85E-12 | 3.73E-11 |
| TPP1 | -0.2999492 | 2.85E-12 | 3.73E-11 |
| CXCL14 | -0.2998707 | 2.89E-12 | 3.78E-11 |
| ZNF365 | -0.2997035 | 2.97E-12 | 3.89E-11 |
| C1orf52 | 0.299693 | 2.98E-12 | 3.89E-11 |
| NACA2 | 0.2996625 | 3.00E-12 | 3.91E-11 |
| VASN | -0.2996411 | 3.01E-12 | 3.93E-11 |
| GRP | -0.2994317 | 3.12E-12 | 4.07E-11 |
| NCDN | -0.2994227 | 3.12E-12 | 4.07E-11 |
| SIPA1L2 | -0.2994207 | 3.12E-12 | 4.07E-11 |
| CCDC25 | 0.2993544 | 3.16E-12 | 4.11E-11 |
| ADCY7 | -0.2992998 | 3.19E-12 | 4.15E-11 |
| TADA2B | -0.299295 | 3.19E-12 | 4.15E-11 |
| PICALM | -0.299179 | 3.26E-12 | 4.23E-11 |
| NUDT15 | 0.2991468 | 3.28E-12 | 4.25E-11 |
| COL8A2 | -0.299133 | 3.28E-12 | 4.26E-11 |
| METTL11B | -0.2991061 | 3.30E-12 | 4.28E-11 |
| NOTCH2 | -0.2990201 | 3.35E-12 | 4.34E-11 |
| LYAR | 0.2989381 | 3.40E-12 | 4.40E-11 |
| METTL2A | 0.2987255 | 3.52E-12 | 4.56E-11 |
| KIF1C | -0.2987028 | 3.54E-12 | 4.58E-11 |
| ISLR | -0.2986677 | 3.56E-12 | 4.60E-11 |
| FLJ10357 | -0.2986614 | 3.56E-12 | 4.60E-11 |
| KCNE4 | -0.2986418 | 3.58E-12 | 4.62E-11 |
| SRPR | -0.2986004 | 3.60E-12 | 4.65E-11 |
| TRIP11 | -0.2985787 | 3.62E-12 | 4.66E-11 |
| MYADM | -0.2985011 | 3.66E-12 | 4.72E-11 |
| ENY2 | 0.2984807 | 3.68E-12 | 4.74E-11 |
| C19orf36 | 0.2984329 | 3.71E-12 | 4.77E-11 |
| OMD | -0.2983429 | 3.77E-12 | 4.84E-11 |
| KCND2 | -0.2982946 | 3.80E-12 | 4.88E-11 |
| EFNB3 | -0.298243 | 3.83E-12 | 4.92E-11 |
| TGFBR2 | -0.2982235 | 3.84E-12 | 4.93E-11 |
| POLR2K | 0.2980897 | 3.93E-12 | 5.05E-11 |
| SGK269 | -0.2980134 | 3.99E-12 | 5.11E-11 |
| RECK | -0.2979988 | 4.00E-12 | 5.12E-11 |
| C1orf131 | 0.2979797 | 4.01E-12 | 5.13E-11 |
| STON1 | -0.2979416 | 4.04E-12 | 5.16E-11 |
| KCND3 | -0.2978665 | 4.09E-12 | 5.23E-11 |
| C3orf31 | 0.2978577 | 4.10E-12 | 5.23E-11 |
| TMEM69 | 0.297806 | 4.13E-12 | 5.28E-11 |
| SNX9 | -0.2977573 | 4.17E-12 | 5.32E-11 |
| ILF3 | 0.2977521 | 4.17E-12 | 5.32E-11 |
| PDRG1 | 0.2977325 | 4.18E-12 | 5.33E-11 |
| THY1 | -0.297689 | 4.22E-12 | 5.37E-11 |
| RBM22 | 0.2975893 | 4.29E-12 | 5.46E-11 |
| ABP1 | -0.2975327 | 4.33E-12 | 5.51E-11 |
| ITGA8 | -0.2974952 | 4.36E-12 | 5.54E-11 |
| SUV420H2 | 0.2974833 | 4.37E-12 | 5.55E-11 |
| COX8A | 0.2974621 | 4.38E-12 | 5.57E-11 |
| ELL3 | 0.2973456 | 4.47E-12 | 5.68E-11 |
| TIAL1 | 0.2973148 | 4.50E-12 | 5.70E-11 |
| RWDD3 | 0.2972478 | 4.55E-12 | 5.77E-11 |
| ST20 | 0.2972367 | 4.56E-12 | 5.77E-11 |
| BBOX1 | -0.2971972 | 4.59E-12 | 5.81E-11 |
| AKAP6 | -0.2969997 | 4.75E-12 | 6.01E-11 |
| CALCOCO1 | -0.2969579 | 4.78E-12 | 6.05E-11 |
| UBE2N | 0.2969148 | 4.82E-12 | 6.09E-11 |
| TMEM47 | -0.2968875 | 4.84E-12 | 6.11E-11 |
| FAM13C | -0.2968317 | 4.89E-12 | 6.17E-11 |
| SLC15A1 | -0.2967689 | 4.94E-12 | 6.23E-11 |
| CTDSP2 | -0.2967599 | 4.95E-12 | 6.23E-11 |
| FAM160B1 | -0.2967441 | 4.96E-12 | 6.25E-11 |
| ARHGAP17 | -0.2967158 | 4.98E-12 | 6.27E-11 |
| GORASP2 | -0.2966916 | 5.01E-12 | 6.30E-11 |
| HECTD1 | -0.2966303 | 5.06E-12 | 6.36E-11 |
| UBL4A | 0.2966213 | 5.07E-12 | 6.37E-11 |
| FOXO1 | -0.2965618 | 5.12E-12 | 6.43E-11 |
| GUCY1A3 | -0.2965331 | 5.14E-12 | 6.45E-11 |
| FREM1 | -0.2964925 | 5.18E-12 | 6.50E-11 |
| ITGA1 | -0.296431 | 5.23E-12 | 6.56E-11 |
| PLD1 | -0.2964205 | 5.24E-12 | 6.57E-11 |
| TAF15 | 0.2963545 | 5.30E-12 | 6.64E-11 |
| RNF24 | -0.2963226 | 5.33E-12 | 6.67E-11 |
| LSM1 | 0.2963188 | 5.34E-12 | 6.67E-11 |
| NME2P1 | 0.2963122 | 5.34E-12 | 6.67E-11 |
| FERMT2 | -0.2962684 | 5.38E-12 | 6.72E-11 |
| SFMBT2 | -0.296223 | 5.42E-12 | 6.77E-11 |
| C16orf53 | 0.2961236 | 5.52E-12 | 6.88E-11 |
| CKLF | 0.2961205 | 5.52E-12 | 6.88E-11 |
| THOC3 | 0.2961082 | 5.53E-12 | 6.89E-11 |
| MFRP | -0.2960709 | 5.57E-12 | 6.93E-11 |
| C1QBP | 0.2960191 | 5.62E-12 | 6.99E-11 |
| ANK2 | -0.2958734 | 5.76E-12 | 7.16E-11 |
| WDR90 | 0.2957947 | 5.84E-12 | 7.25E-11 |
| LEPROT | -0.2957677 | 5.86E-12 | 7.28E-11 |
| ADAMTS10 | -0.2957547 | 5.88E-12 | 7.29E-11 |
| TXLNB | -0.2956868 | 5.95E-12 | 7.37E-11 |
| MYO19 | 0.2956788 | 5.95E-12 | 7.38E-11 |
| KLK4 | -0.295604 | 6.03E-12 | 7.47E-11 |
| SLC25A14 | 0.295548 | 6.09E-12 | 7.54E-11 |
| TSPAN11 | -0.2955277 | 6.11E-12 | 7.56E-11 |
| VCL | -0.2954432 | 6.20E-12 | 7.66E-11 |
| LRRC17 | -0.2954416 | 6.20E-12 | 7.66E-11 |
| MON1B | -0.2953503 | 6.30E-12 | 7.78E-11 |
| HTRA1 | -0.2952513 | 6.41E-12 | 7.91E-11 |
| PAR5 | -0.2952358 | 6.42E-12 | 7.92E-11 |
| GPR111 | -0.2952336 | 6.43E-12 | 7.92E-11 |
| LUC7L3 | 0.2951952 | 6.47E-12 | 7.96E-11 |
| FBXL7 | -0.2951934 | 6.47E-12 | 7.96E-11 |
| PQBP1 | 0.2951849 | 6.48E-12 | 7.97E-11 |
| TRA2A | 0.2951227 | 6.55E-12 | 8.05E-11 |
| PLXDC1 | -0.2951147 | 6.56E-12 | 8.06E-11 |
| SSX2IP | 0.2950966 | 6.58E-12 | 8.08E-11 |
| MSRB3 | -0.2950898 | 6.59E-12 | 8.08E-11 |
| HSD17B10 | 0.2950014 | 6.69E-12 | 8.20E-11 |
| PBXIP1 | -0.2949963 | 6.69E-12 | 8.20E-11 |
| ATP8B2 | -0.2948168 | 6.90E-12 | 8.45E-11 |
| MRGPRF | -0.2948071 | 6.91E-12 | 8.46E-11 |
| C19orf26 | -0.2947877 | 6.93E-12 | 8.48E-11 |
| SLC22A23 | -0.2947763 | 6.95E-12 | 8.49E-11 |
| KIDINS220 | -0.2947715 | 6.95E-12 | 8.50E-11 |
| CUX1 | -0.2947476 | 6.98E-12 | 8.53E-11 |
| LUM | -0.294724 | 7.01E-12 | 8.56E-11 |
| ATP6V0A1 | -0.2946998 | 7.04E-12 | 8.59E-11 |
| JAM2 | -0.2945201 | 7.26E-12 | 8.85E-11 |
| CDCA7L | 0.2944308 | 7.37E-12 | 8.98E-11 |
| C6orf147 | 0.2944031 | 7.40E-12 | 9.01E-11 |
| MSX2 | -0.2943951 | 7.41E-12 | 9.02E-11 |
| ELFN1 | -0.294379 | 7.43E-12 | 9.04E-11 |
| JAG1 | -0.2943372 | 7.49E-12 | 9.10E-11 |
| WNT5A | -0.2942816 | 7.56E-12 | 9.18E-11 |
| FGF10 | -0.294194 | 7.67E-12 | 9.31E-11 |
| C18orf22 | 0.294194 | 7.67E-12 | 9.31E-11 |
| FLJ22536 | -0.2941636 | 7.71E-12 | 9.35E-11 |
| HSPB2 | -0.2941406 | 7.74E-12 | 9.38E-11 |
| HIST1H2BO | 0.2940628 | 7.84E-12 | 9.50E-11 |
| PCYOX1 | -0.2940396 | 7.88E-12 | 9.53E-11 |
| MUCL1 | -0.2939843 | 7.95E-12 | 9.62E-11 |
| DUSP18 | -0.2939814 | 7.95E-12 | 9.62E-11 |
| LHX2 | 0.2939521 | 7.99E-12 | 9.66E-11 |
| MYOF | -0.2938984 | 8.07E-12 | 9.74E-11 |
| MRPL12 | 0.2938796 | 8.09E-12 | 9.77E-11 |
| SLC8A3 | -0.2938657 | 8.11E-12 | 9.78E-11 |
| PPIAL4G | 0.293759 | 8.26E-12 | 9.96E-11 |
| PSME2 | 0.2935437 | 8.57E-12 | 1.03E-10 |
| RALGAPA2 | -0.2934664 | 8.68E-12 | 1.04E-10 |
| TOMM6 | 0.2934582 | 8.69E-12 | 1.05E-10 |
| C10orf4 | 0.2933377 | 8.87E-12 | 1.07E-10 |
| C6orf167 | 0.2932987 | 8.93E-12 | 1.07E-10 |
| BNC2 | -0.2932885 | 8.95E-12 | 1.07E-10 |
| TUBA1A | -0.2932425 | 9.02E-12 | 1.08E-10 |
| LOC150786 | -0.293224 | 9.04E-12 | 1.08E-10 |
| SNRNP70 | 0.293209 | 9.07E-12 | 1.09E-10 |
| TSPAN9 | -0.2931806 | 9.11E-12 | 1.09E-10 |
| SEPHS1 | 0.293113 | 9.22E-12 | 1.10E-10 |
| HECW2 | -0.2931063 | 9.23E-12 | 1.10E-10 |
| SCNM1 | 0.2930689 | 9.29E-12 | 1.11E-10 |
| ANAPC7 | 0.2930572 | 9.30E-12 | 1.11E-10 |
| LAMC1 | -0.2930055 | 9.39E-12 | 1.12E-10 |
| C9orf23 | 0.292996 | 9.40E-12 | 1.12E-10 |
| TMEM184B | -0.2928861 | 9.58E-12 | 1.14E-10 |
| C8orf41 | 0.2928661 | 9.61E-12 | 1.15E-10 |
| SNF8 | 0.2927727 | 9.76E-12 | 1.16E-10 |
| HIGD2A | 0.2926703 | 9.93E-12 | 1.18E-10 |
| LRRC41 | -0.2925911 | 1.01E-11 | 1.20E-10 |
| SPRED3 | -0.2925832 | 1.01E-11 | 1.20E-10 |
| HSPD1 | 0.2925543 | 1.01E-11 | 1.20E-10 |
| EIF3J | 0.2925501 | 1.01E-11 | 1.20E-10 |
| PPAPDC1A | -0.2924996 | 1.02E-11 | 1.21E-10 |
| RPS10 | 0.2924937 | 1.02E-11 | 1.21E-10 |
| NDUFAF2 | 0.2924211 | 1.04E-11 | 1.23E-10 |
| PDZRN3 | -0.2923838 | 1.04E-11 | 1.24E-10 |
| PDLIM5 | -0.2923013 | 1.06E-11 | 1.25E-10 |
| ANGPTL7 | -0.2922313 | 1.07E-11 | 1.27E-10 |
| PTK7 | -0.2921704 | 1.08E-11 | 1.28E-10 |
| GAB1 | -0.2921625 | 1.08E-11 | 1.28E-10 |
| SOS2 | -0.2920419 | 1.10E-11 | 1.31E-10 |
| PCDH17 | -0.2920116 | 1.11E-11 | 1.31E-10 |
| NPAS2 | -0.2919771 | 1.12E-11 | 1.32E-10 |
| VPS26B | -0.2919682 | 1.12E-11 | 1.32E-10 |
| PTPN14 | -0.2918996 | 1.13E-11 | 1.33E-10 |
| RBM24 | -0.2918896 | 1.13E-11 | 1.34E-10 |
| SYNE1 | -0.2918863 | 1.13E-11 | 1.34E-10 |
| ZNF135 | -0.2916824 | 1.17E-11 | 1.38E-10 |
| NDUFA9 | 0.291612 | 1.19E-11 | 1.40E-10 |
| PSMC3 | 0.2914514 | 1.22E-11 | 1.43E-10 |
| SEC23A | -0.2913894 | 1.23E-11 | 1.45E-10 |
| TGFB3 | -0.2913686 | 1.24E-11 | 1.45E-10 |
| POLR1E | 0.2913509 | 1.24E-11 | 1.46E-10 |
| HIPK3 | -0.2913135 | 1.25E-11 | 1.46E-10 |
| ELMO2 | -0.291135 | 1.29E-11 | 1.51E-10 |
| CCT4 | 0.2911164 | 1.29E-11 | 1.51E-10 |
| ZNF232 | 0.2910878 | 1.30E-11 | 1.52E-10 |
| BOD1L | -0.291031 | 1.31E-11 | 1.53E-10 |
| HSD17B7 | 0.2910168 | 1.31E-11 | 1.54E-10 |
| ARHGAP1 | -0.2909627 | 1.32E-11 | 1.55E-10 |
| SHFM1 | 0.2909433 | 1.33E-11 | 1.55E-10 |
| SFRS2B | -0.2908874 | 1.34E-11 | 1.57E-10 |
| MRPS7 | 0.2908738 | 1.34E-11 | 1.57E-10 |
| KLF10 | -0.2908581 | 1.35E-11 | 1.57E-10 |
| AGMAT | 0.2908469 | 1.35E-11 | 1.57E-10 |
| FKBP3 | 0.2907887 | 1.36E-11 | 1.59E-10 |
| PLXNA4 | -0.2907773 | 1.37E-11 | 1.59E-10 |
| LOC401052 | 0.2907646 | 1.37E-11 | 1.59E-10 |
| ISOC1 | 0.2906761 | 1.39E-11 | 1.62E-10 |
| CBLB | -0.2906569 | 1.39E-11 | 1.62E-10 |
| GATSL1 | -0.2906489 | 1.40E-11 | 1.62E-10 |
| PARK7 | 0.2906122 | 1.40E-11 | 1.63E-10 |
| FAM111A | 0.2905844 | 1.41E-11 | 1.64E-10 |
| PDCD2L | 0.290534 | 1.42E-11 | 1.65E-10 |
| ZNF660 | -0.2903641 | 1.46E-11 | 1.70E-10 |
| CASP6 | 0.2902085 | 1.50E-11 | 1.74E-10 |
| C11orf85 | 0.2901315 | 1.52E-11 | 1.76E-10 |
| ZBTB47 | -0.2900416 | 1.55E-11 | 1.79E-10 |
| DYNLT1 | 0.2900359 | 1.55E-11 | 1.79E-10 |
| EDC3 | 0.2899696 | 1.56E-11 | 1.81E-10 |
| C15orf24 | 0.2899173 | 1.58E-11 | 1.82E-10 |
| POLD3 | 0.2898744 | 1.59E-11 | 1.83E-10 |
| NCAM1 | -0.2898513 | 1.60E-11 | 1.84E-10 |
| ABCB4 | -0.289836 | 1.60E-11 | 1.84E-10 |
| DSG1 | -0.2897196 | 1.63E-11 | 1.88E-10 |
| CNPY2 | 0.2897011 | 1.64E-11 | 1.88E-10 |
| BAI3 | -0.289698 | 1.64E-11 | 1.88E-10 |
| MAD2L1BP | 0.2896017 | 1.66E-11 | 1.91E-10 |
| ACBD7 | 0.2895819 | 1.67E-11 | 1.92E-10 |
| UBTD1 | -0.2895601 | 1.68E-11 | 1.92E-10 |
| CDH6 | -0.2895269 | 1.68E-11 | 1.93E-10 |
| ALDH1L2 | -0.2894943 | 1.69E-11 | 1.94E-10 |
| FAM131B | -0.2894629 | 1.70E-11 | 1.95E-10 |
| GRIA3 | -0.2894312 | 1.71E-11 | 1.96E-10 |
| CCDC89 | -0.2894024 | 1.72E-11 | 1.97E-10 |
| F10 | -0.2893751 | 1.73E-11 | 1.98E-10 |
| TRIM28 | 0.2893629 | 1.73E-11 | 1.98E-10 |
| TWIST2 | -0.2893467 | 1.74E-11 | 1.99E-10 |
| ST8SIA2 | -0.2893228 | 1.74E-11 | 1.99E-10 |
| SMG6 | -0.2893078 | 1.75E-11 | 2.00E-10 |
| CCBL1 | 0.2892699 | 1.76E-11 | 2.01E-10 |
| RUVBL1 | 0.2890397 | 1.83E-11 | 2.08E-10 |
| BLOC1S1 | 0.2890378 | 1.83E-11 | 2.08E-10 |
| BACE1 | -0.2890275 | 1.83E-11 | 2.09E-10 |
| MRPL24 | 0.2890143 | 1.84E-11 | 2.09E-10 |
| NFE2L1 | -0.2889037 | 1.87E-11 | 2.13E-10 |
| SDR39U1 | 0.2888923 | 1.87E-11 | 2.13E-10 |
| C14orf2 | 0.2888812 | 1.88E-11 | 2.13E-10 |
| SFRS6 | 0.2888384 | 1.89E-11 | 2.15E-10 |
| GFRA1 | -0.2887706 | 1.91E-11 | 2.17E-10 |
| GPX1 | 0.2887109 | 1.93E-11 | 2.19E-10 |
| LIMK2 | -0.2886935 | 1.94E-11 | 2.20E-10 |
| ZNF519 | 0.2886762 | 1.94E-11 | 2.20E-10 |
| PCBD2 | 0.2886736 | 1.94E-11 | 2.20E-10 |
| PIGB | 0.288666 | 1.94E-11 | 2.20E-10 |
| NME2 | 0.2886307 | 1.96E-11 | 2.21E-10 |
| C9orf140 | 0.2886207 | 1.96E-11 | 2.22E-10 |
| NDUFAB1 | 0.2886163 | 1.96E-11 | 2.22E-10 |
| FAM158A | 0.2886028 | 1.97E-11 | 2.22E-10 |
| COL15A1 | -0.2884937 | 2.00E-11 | 2.26E-10 |
| UBE4A | -0.2884866 | 2.00E-11 | 2.26E-10 |
| ABT1 | 0.288434 | 2.02E-11 | 2.28E-10 |
| CSGALNACT1 | -0.2883928 | 2.04E-11 | 2.29E-10 |
| SHC2 | -0.288334 | 2.06E-11 | 2.32E-10 |
| PKD1 | -0.2882864 | 2.07E-11 | 2.33E-10 |
| PDZD8 | -0.2882597 | 2.08E-11 | 2.34E-10 |
| MYH10 | -0.2882098 | 2.10E-11 | 2.36E-10 |
| FMN1 | -0.2882054 | 2.10E-11 | 2.36E-10 |
| UTRN | -0.2881972 | 2.10E-11 | 2.36E-10 |
| SEC24A | -0.2881784 | 2.11E-11 | 2.37E-10 |
| CCNH | 0.2881565 | 2.12E-11 | 2.38E-10 |
| TNKS1BP1 | -0.2881321 | 2.13E-11 | 2.38E-10 |
| CREB3L1 | -0.2880987 | 2.14E-11 | 2.40E-10 |
| THSD7A | -0.288034 | 2.16E-11 | 2.42E-10 |
| ACD | 0.2880338 | 2.16E-11 | 2.42E-10 |
| C12orf10 | 0.2880243 | 2.16E-11 | 2.42E-10 |
| IL6R | -0.2879922 | 2.18E-11 | 2.43E-10 |
| ANAPC10 | 0.2879749 | 2.18E-11 | 2.44E-10 |
| RPL12 | 0.2879269 | 2.20E-11 | 2.46E-10 |
| UBE2I | 0.287886 | 2.21E-11 | 2.47E-10 |
| LUZP1 | -0.2878557 | 2.22E-11 | 2.48E-10 |
| A2BP1 | -0.2878105 | 2.24E-11 | 2.50E-10 |
| LOC149837 | -0.287718 | 2.28E-11 | 2.54E-10 |
| RPL27 | 0.2876936 | 2.29E-11 | 2.55E-10 |
| ZBTB20 | -0.2876602 | 2.30E-11 | 2.56E-10 |
| STARD8 | -0.2875219 | 2.35E-11 | 2.62E-10 |
| TTF1 | 0.2875166 | 2.35E-11 | 2.62E-10 |
| PFDN1 | 0.2874816 | 2.37E-11 | 2.63E-10 |
| ADAMTS8 | -0.2873681 | 2.41E-11 | 2.68E-10 |
| HNRNPH1 | 0.2873646 | 2.41E-11 | 2.68E-10 |
| NCOR2 | -0.2872633 | 2.45E-11 | 2.73E-10 |
| RPS8 | 0.2872598 | 2.46E-11 | 2.73E-10 |
| ADAMTSL1 | -0.2872278 | 2.47E-11 | 2.74E-10 |
| ERP29 | 0.2872232 | 2.47E-11 | 2.74E-10 |
| NDUFB3 | 0.287178 | 2.49E-11 | 2.76E-10 |
| TAZ | 0.2871447 | 2.50E-11 | 2.77E-10 |
| C12orf47 | 0.2870547 | 2.54E-11 | 2.81E-10 |
| ATPIF1 | 0.2870405 | 2.55E-11 | 2.82E-10 |
| CCL22 | -0.2870226 | 2.55E-11 | 2.82E-10 |
| GPR116 | -0.2869196 | 2.60E-11 | 2.87E-10 |
| GPR155 | -0.2868819 | 2.61E-11 | 2.89E-10 |
| RBX1 | 0.2866668 | 2.71E-11 | 2.99E-10 |
| PRUNE2 | -0.2866504 | 2.72E-11 | 3.00E-10 |
| MYO1C | -0.2865936 | 2.74E-11 | 3.02E-10 |
| AMPH | -0.2865896 | 2.74E-11 | 3.02E-10 |
| NRIP1 | -0.2865443 | 2.76E-11 | 3.04E-10 |
| PASK | 0.2864011 | 2.83E-11 | 3.12E-10 |
| TRPC4 | -0.2863085 | 2.87E-11 | 3.16E-10 |
| MRPS21 | 0.2861858 | 2.93E-11 | 3.22E-10 |
| KIAA0182 | -0.2861285 | 2.96E-11 | 3.25E-10 |
| RPLP0 | 0.2861256 | 2.96E-11 | 3.25E-10 |
| SOCS5 | -0.2861206 | 2.96E-11 | 3.25E-10 |
| TXNL4A | 0.2861203 | 2.96E-11 | 3.25E-10 |
| WDR74 | 0.2860748 | 2.99E-11 | 3.27E-10 |
| RDH12 | -0.2859993 | 3.02E-11 | 3.31E-10 |
| RPL24 | 0.2859772 | 3.03E-11 | 3.32E-10 |
| COL4A1 | -0.2859146 | 3.07E-11 | 3.35E-10 |
| HIST2H2BA | -0.2859139 | 3.07E-11 | 3.35E-10 |
| MFSD2B | 0.2859123 | 3.07E-11 | 3.35E-10 |
| ZNF789 | 0.2858873 | 3.08E-11 | 3.37E-10 |
| MRPL52 | 0.2858662 | 3.09E-11 | 3.38E-10 |
| TOR3A | 0.2857873 | 3.13E-11 | 3.42E-10 |
| SOX11 | -0.2857513 | 3.15E-11 | 3.44E-10 |
| NDRG4 | -0.2856811 | 3.19E-11 | 3.48E-10 |
| SIK2 | -0.28557 | 3.25E-11 | 3.54E-10 |
| ACN9 | 0.2854919 | 3.29E-11 | 3.58E-10 |
| MAP1LC3B | -0.2854535 | 3.31E-11 | 3.60E-10 |
| EIF2S2 | 0.2853492 | 3.37E-11 | 3.66E-10 |
| DARS2 | 0.2852871 | 3.40E-11 | 3.70E-10 |
| RBM10 | 0.2852171 | 3.44E-11 | 3.74E-10 |
| HIST1H2AH | 0.285189 | 3.45E-11 | 3.75E-10 |
| CHRNG | -0.2851476 | 3.48E-11 | 3.78E-10 |
| CPXM1 | -0.2850666 | 3.53E-11 | 3.83E-10 |
| C1orf174 | 0.2850407 | 3.54E-11 | 3.84E-10 |
| EEF1A2 | -0.2850312 | 3.55E-11 | 3.84E-10 |
| GLIS2 | -0.2849481 | 3.59E-11 | 3.89E-10 |
| NFATC2 | -0.2848623 | 3.65E-11 | 3.95E-10 |
| AOC3 | -0.2848549 | 3.65E-11 | 3.95E-10 |
| AHR | -0.2848189 | 3.67E-11 | 3.97E-10 |
| ADH5 | 0.2848 | 3.68E-11 | 3.98E-10 |
| TMEM223 | 0.2847983 | 3.68E-11 | 3.98E-10 |
| SNTB1 | -0.2847779 | 3.70E-11 | 3.99E-10 |
| SRP19 | 0.284757 | 3.71E-11 | 4.00E-10 |
| CAMTA2 | -0.2847267 | 3.73E-11 | 4.02E-10 |
| EFNB2 | -0.2846998 | 3.74E-11 | 4.04E-10 |
| LOXL2 | -0.2846939 | 3.75E-11 | 4.04E-10 |
| LDB2 | -0.2846259 | 3.79E-11 | 4.08E-10 |
| ENPP1 | -0.2845228 | 3.85E-11 | 4.15E-10 |
| C1orf14 | 0.2845107 | 3.86E-11 | 4.15E-10 |
| THG1L | 0.284498 | 3.87E-11 | 4.16E-10 |
| LCMT2 | 0.2844417 | 3.91E-11 | 4.20E-10 |
| SMOX | -0.2844368 | 3.91E-11 | 4.20E-10 |
| HAUS6 | 0.2843112 | 3.99E-11 | 4.28E-10 |
| GPR137B | -0.2842434 | 4.03E-11 | 4.33E-10 |
| TMEM45A | -0.2841437 | 4.10E-11 | 4.40E-10 |
| ADI1 | 0.2840767 | 4.15E-11 | 4.44E-10 |
| CLPP | 0.2840589 | 4.16E-11 | 4.45E-10 |
| CWC27 | 0.2840589 | 4.16E-11 | 4.45E-10 |
| SHISA4 | -0.2840221 | 4.18E-11 | 4.48E-10 |
| TAB2 | -0.2840142 | 4.19E-11 | 4.48E-10 |
| SSPN | -0.2839947 | 4.20E-11 | 4.49E-10 |
| LRRN4CL | -0.2839639 | 4.22E-11 | 4.51E-10 |
| DNAJC19 | 0.2839256 | 4.25E-11 | 4.54E-10 |
| PUS1 | 0.2838642 | 4.29E-11 | 4.58E-10 |
| MBTPS1 | -0.2838642 | 4.29E-11 | 4.58E-10 |
| GLE1 | 0.2838628 | 4.29E-11 | 4.58E-10 |
| NOL11 | 0.2838055 | 4.33E-11 | 4.62E-10 |
| ZNF646 | -0.2837911 | 4.34E-11 | 4.63E-10 |
| FRAT2 | 0.2837639 | 4.36E-11 | 4.64E-10 |
| LSMD1 | 0.2837006 | 4.41E-11 | 4.69E-10 |
| PPM1L | -0.2836924 | 4.41E-11 | 4.69E-10 |
| XPNPEP2 | -0.2836093 | 4.47E-11 | 4.75E-10 |
| DAND5 | 0.2836088 | 4.47E-11 | 4.75E-10 |
| SCN5A | -0.2835995 | 4.48E-11 | 4.76E-10 |
| INO80C | 0.2835796 | 4.50E-11 | 4.77E-10 |
| PTPRE | -0.2835598 | 4.51E-11 | 4.78E-10 |
| SOX5 | -0.2835402 | 4.52E-11 | 4.80E-10 |
| PRMT1 | 0.2835338 | 4.53E-11 | 4.80E-10 |
| ITGB3 | -0.2835168 | 4.54E-11 | 4.81E-10 |
| HDGF | 0.2834984 | 4.56E-11 | 4.82E-10 |
| NIPAL1 | -0.2834979 | 4.56E-11 | 4.82E-10 |
| CHD9 | -0.2834972 | 4.56E-11 | 4.82E-10 |
| POLG2 | 0.2834581 | 4.59E-11 | 4.85E-10 |
| SCN1B | -0.2833696 | 4.65E-11 | 4.91E-10 |
| KHK | 0.28336 | 4.66E-11 | 4.92E-10 |
| CD44 | -0.2833177 | 4.69E-11 | 4.95E-10 |
| TUBB2C | 0.2832895 | 4.71E-11 | 4.97E-10 |
| PCDHGB7 | -0.2832013 | 4.78E-11 | 5.04E-10 |
| ABCA12 | -0.2831844 | 4.80E-11 | 5.05E-10 |
| SOD1 | 0.2831208 | 4.84E-11 | 5.10E-10 |
| APP | -0.2830981 | 4.86E-11 | 5.12E-10 |
| SULT1C4 | -0.2830876 | 4.87E-11 | 5.12E-10 |
| NUMBL | -0.2830724 | 4.88E-11 | 5.13E-10 |
| NDUFAF1 | 0.2830131 | 4.93E-11 | 5.18E-10 |
| ZMAT3 | -0.2829461 | 4.98E-11 | 5.23E-10 |
| N4BP1 | -0.2828848 | 5.03E-11 | 5.28E-10 |
| DUS4L | 0.2828346 | 5.08E-11 | 5.33E-10 |
| ANLN | 0.2826632 | 5.22E-11 | 5.47E-10 |
| NDP | -0.2826214 | 5.25E-11 | 5.51E-10 |
| COL10A1 | -0.282595 | 5.28E-11 | 5.53E-10 |
| C14orf166 | 0.2825881 | 5.28E-11 | 5.53E-10 |
| ANO6 | -0.2825347 | 5.33E-11 | 5.58E-10 |
| UNC5C | -0.2825195 | 5.34E-11 | 5.59E-10 |
| CKAP2 | 0.2824203 | 5.43E-11 | 5.68E-10 |
| KIAA0430 | -0.2824156 | 5.43E-11 | 5.68E-10 |
| SLC2A10 | -0.2822356 | 5.59E-11 | 5.84E-10 |
| ENG | -0.2822285 | 5.60E-11 | 5.85E-10 |
| CDH5 | -0.2821342 | 5.69E-11 | 5.93E-10 |
| ATXN7L2 | 0.2820944 | 5.72E-11 | 5.97E-10 |
| C6orf136 | 0.2820782 | 5.74E-11 | 5.98E-10 |
| SPAG9 | -0.282046 | 5.77E-11 | 6.01E-10 |
| NDUFB5 | 0.28203 | 5.78E-11 | 6.02E-10 |
| RALY | 0.2819801 | 5.83E-11 | 6.07E-10 |
| TSHZ2 | -0.2819279 | 5.88E-11 | 6.12E-10 |
| 5-Sep | -0.2818836 | 5.92E-11 | 6.16E-10 |
| GFRA2 | -0.2818504 | 5.96E-11 | 6.19E-10 |
| GPC6 | -0.2817814 | 6.02E-11 | 6.25E-10 |
| KAL1 | -0.2817367 | 6.07E-11 | 6.29E-10 |
| SLC6A6 | -0.2817014 | 6.10E-11 | 6.33E-10 |
| CLEC16A | -0.2816625 | 6.14E-11 | 6.36E-10 |
| PIH1D1 | 0.2816196 | 6.18E-11 | 6.41E-10 |
| KRT1 | -0.2816152 | 6.19E-11 | 6.41E-10 |
| ZFR2 | 0.2815779 | 6.22E-11 | 6.44E-10 |
| REL | -0.28149 | 6.31E-11 | 6.53E-10 |
| ATP11A | -0.2814822 | 6.32E-11 | 6.54E-10 |
| COX16 | 0.2814377 | 6.37E-11 | 6.58E-10 |
| SSTR1 | -0.2813921 | 6.41E-11 | 6.63E-10 |
| RUNX1 | -0.2813195 | 6.49E-11 | 6.70E-10 |
| GRID1 | -0.2812911 | 6.52E-11 | 6.73E-10 |
| CAMSAP1L1 | -0.2812399 | 6.57E-11 | 6.78E-10 |
| RBM8A | 0.2811324 | 6.69E-11 | 6.90E-10 |
| TRAP1 | 0.281095 | 6.73E-11 | 6.93E-10 |
| SPARCL1 | -0.2810834 | 6.74E-11 | 6.94E-10 |
| CYB5R3 | -0.2810609 | 6.77E-11 | 6.96E-10 |
| NDUFA8 | 0.2810398 | 6.79E-11 | 6.98E-10 |
| PCDHB5 | -0.2809898 | 6.84E-11 | 7.04E-10 |
| PGP | 0.280923 | 6.92E-11 | 7.11E-10 |
| ERC1 | -0.2808273 | 7.03E-11 | 7.22E-10 |
| SNHG3 | 0.2808158 | 7.04E-11 | 7.23E-10 |
| KIFAP3 | -0.2808073 | 7.05E-11 | 7.23E-10 |
| GALNS | -0.2807458 | 7.12E-11 | 7.30E-10 |
| VPS53 | -0.2806498 | 7.23E-11 | 7.41E-10 |
| XYLT1 | -0.2806252 | 7.26E-11 | 7.44E-10 |
| APOM | 0.2805877 | 7.30E-11 | 7.48E-10 |
| TIE1 | -0.2805847 | 7.31E-11 | 7.48E-10 |
| PSMD9 | 0.2805804 | 7.31E-11 | 7.48E-10 |
| ACBD6 | 0.2805229 | 7.38E-11 | 7.55E-10 |
| EXOC6B | -0.2805075 | 7.40E-11 | 7.56E-10 |
| LHFPL2 | -0.2804828 | 7.43E-11 | 7.59E-10 |
| NACA | 0.2803858 | 7.54E-11 | 7.70E-10 |
| LONP2 | -0.2803264 | 7.62E-11 | 7.77E-10 |
| C6orf26 | 0.2801933 | 7.78E-11 | 7.93E-10 |
| CLTCL1 | -0.2801917 | 7.78E-11 | 7.93E-10 |
| GCNT4 | -0.2801632 | 7.82E-11 | 7.97E-10 |
| EHD2 | -0.280153 | 7.83E-11 | 7.98E-10 |
| CERCAM | -0.2801495 | 7.84E-11 | 7.98E-10 |
| PCDHGA11 | -0.2801449 | 7.84E-11 | 7.98E-10 |
| GTF2E2 | 0.280054 | 7.96E-11 | 8.09E-10 |
| SIRPB1 | -0.2800288 | 7.99E-11 | 8.12E-10 |
| LAGE3 | 0.2800132 | 8.01E-11 | 8.14E-10 |
| JPH3 | -0.2799727 | 8.06E-11 | 8.19E-10 |
| MLL3 | -0.2799488 | 8.09E-11 | 8.21E-10 |
| ARHGAP19 | 0.2797237 | 8.39E-11 | 8.51E-10 |
| APCDD1L | -0.2796423 | 8.50E-11 | 8.62E-10 |
| CAP2 | -0.2796331 | 8.52E-11 | 8.63E-10 |
| ZCCHC17 | 0.2796274 | 8.52E-11 | 8.63E-10 |
| LYNX1 | -0.2795836 | 8.58E-11 | 8.69E-10 |
| ATP8B5P | 0.2795739 | 8.60E-11 | 8.70E-10 |
| TXNL4B | 0.2795193 | 8.67E-11 | 8.77E-10 |
| GOLGA5 | -0.2795075 | 8.69E-11 | 8.78E-10 |
| GGT5 | -0.2795039 | 8.69E-11 | 8.78E-10 |
| RNF103 | -0.2793626 | 8.89E-11 | 8.98E-10 |
| ADAMTS4 | -0.2793515 | 8.91E-11 | 8.99E-10 |
| B4GALNT3 | -0.2793434 | 8.92E-11 | 9.00E-10 |
| NDEL1 | -0.2792663 | 9.03E-11 | 9.11E-10 |
| C20orf103 | -0.2792022 | 9.12E-11 | 9.19E-10 |
| COMP | -0.2791658 | 9.18E-11 | 9.24E-10 |
| NBR1 | -0.2791339 | 9.22E-11 | 9.29E-10 |
| KLHL30 | -0.2791003 | 9.27E-11 | 9.33E-10 |
| C9orf114 | 0.2790732 | 9.32E-11 | 9.37E-10 |
| POLR2F | 0.2790658 | 9.33E-11 | 9.37E-10 |
| POLD2 | 0.2790525 | 9.35E-11 | 9.39E-10 |
| DHTKD1 | 0.279049 | 9.35E-11 | 9.39E-10 |
| CNIH4 | 0.2788248 | 9.69E-11 | 9.73E-10 |
| IP6K3 | -0.2786848 | 9.91E-11 | 9.94E-10 |
| C1orf31 | 0.2786809 | 9.92E-11 | 9.95E-10 |
| MRPS34 | 0.2786677 | 9.94E-11 | 9.96E-10 |
| HSBP1L1 | 0.2786419 | 9.98E-11 | 1.00E-09 |
| PREPL | -0.2785911 | 1.01E-10 | 1.01E-09 |
| D4S234E | -0.2785711 | 1.01E-10 | 1.01E-09 |
| LPAR4 | -0.2785628 | 1.01E-10 | 1.01E-09 |
| PIP5K1C | -0.2785485 | 1.01E-10 | 1.01E-09 |
| ATP13A2 | -0.2785448 | 1.01E-10 | 1.01E-09 |
| UBL3 | -0.278535 | 1.02E-10 | 1.01E-09 |
| FAM83G | -0.2785312 | 1.02E-10 | 1.01E-09 |
| IRS1 | -0.2784637 | 1.03E-10 | 1.02E-09 |
| MYH11 | -0.2783591 | 1.04E-10 | 1.04E-09 |
| MAN2B2 | -0.2783189 | 1.05E-10 | 1.05E-09 |
| DTNA | -0.2783008 | 1.05E-10 | 1.05E-09 |
| RPL41 | 0.2782444 | 1.06E-10 | 1.06E-09 |
| MTG1 | 0.2781373 | 1.08E-10 | 1.08E-09 |
| ABCA8 | -0.278131 | 1.08E-10 | 1.08E-09 |
| EP300 | -0.2780105 | 1.10E-10 | 1.10E-09 |
| CNRIP1 | -0.2779611 | 1.11E-10 | 1.11E-09 |
| DDX6 | -0.2779576 | 1.11E-10 | 1.11E-09 |
| TCAM1P | 0.2779379 | 1.12E-10 | 1.11E-09 |
| GEMIN7 | 0.2779271 | 1.12E-10 | 1.11E-09 |
| ATP1B4 | -0.2778105 | 1.14E-10 | 1.13E-09 |
| TNC | -0.2778002 | 1.14E-10 | 1.13E-09 |
| CKAP2L | 0.2776459 | 1.17E-10 | 1.16E-09 |
| HHIP | -0.277598 | 1.18E-10 | 1.17E-09 |
| PTH1R | -0.277564 | 1.19E-10 | 1.17E-09 |
| FBXO10 | -0.2774629 | 1.20E-10 | 1.19E-09 |
| PDXDC1 | -0.2774124 | 1.21E-10 | 1.20E-09 |
| C11orf10 | 0.2773713 | 1.22E-10 | 1.21E-09 |
| CYS1 | -0.2773193 | 1.23E-10 | 1.22E-09 |
| LPL | -0.2772502 | 1.25E-10 | 1.23E-09 |
| MRPL38 | 0.2771843 | 1.26E-10 | 1.24E-09 |
| RPL36 | 0.2771089 | 1.27E-10 | 1.26E-09 |
| GART | 0.2770253 | 1.29E-10 | 1.27E-09 |
| USP39 | 0.2769917 | 1.30E-10 | 1.28E-09 |
| GNRH1 | 0.2769686 | 1.30E-10 | 1.28E-09 |
| ZPBP2 | 0.2769132 | 1.31E-10 | 1.29E-09 |
| GALNT13 | -0.2768976 | 1.32E-10 | 1.30E-09 |
| C9orf37 | 0.2768787 | 1.32E-10 | 1.30E-09 |
| RPS6KA2 | -0.2768425 | 1.33E-10 | 1.31E-09 |
| RPL11 | 0.2768372 | 1.33E-10 | 1.31E-09 |
| ZNF491 | -0.2767157 | 1.36E-10 | 1.33E-09 |
| PABPC5 | -0.2766969 | 1.36E-10 | 1.34E-09 |
| SMS | 0.2766826 | 1.36E-10 | 1.34E-09 |
| WDR86 | -0.2766621 | 1.37E-10 | 1.34E-09 |
| TPCN1 | -0.2766096 | 1.38E-10 | 1.35E-09 |
| TRUB2 | 0.2765805 | 1.39E-10 | 1.36E-09 |
| PCDHGA7 | -0.2765749 | 1.39E-10 | 1.36E-09 |
| DUS3L | 0.2764758 | 1.41E-10 | 1.38E-09 |
| KIAA1024 | -0.2764753 | 1.41E-10 | 1.38E-09 |
| GOLGB1 | -0.2764736 | 1.41E-10 | 1.38E-09 |
| MMP11 | -0.2764734 | 1.41E-10 | 1.38E-09 |
| NALCN | -0.2764603 | 1.41E-10 | 1.38E-09 |
| PLP2 | 0.2763969 | 1.43E-10 | 1.39E-09 |
| SIRPA | -0.2763843 | 1.43E-10 | 1.40E-09 |
| UBR4 | -0.2763723 | 1.43E-10 | 1.40E-09 |
| DCK | 0.276356 | 1.44E-10 | 1.40E-09 |
| MNAT1 | 0.2763405 | 1.44E-10 | 1.40E-09 |
| GTF2H4 | 0.2763335 | 1.44E-10 | 1.40E-09 |
| UBE4B | -0.2762123 | 1.47E-10 | 1.43E-09 |
| PTPMT1 | 0.2761894 | 1.47E-10 | 1.43E-09 |
| PANX1 | -0.2761408 | 1.49E-10 | 1.45E-09 |
| MEF2C | -0.276098 | 1.50E-10 | 1.45E-09 |
| DUSP13 | -0.2760965 | 1.50E-10 | 1.45E-09 |
| FOXO3B | -0.2760459 | 1.51E-10 | 1.47E-09 |
| TMEM59L | -0.2758705 | 1.55E-10 | 1.51E-09 |
| KLK7 | -0.2758628 | 1.55E-10 | 1.51E-09 |
| RPL14 | 0.2758299 | 1.56E-10 | 1.51E-09 |
| PXDNL | -0.2758159 | 1.56E-10 | 1.52E-09 |
| PTPRB | -0.275802 | 1.57E-10 | 1.52E-09 |
| ORAI1 | 0.2757921 | 1.57E-10 | 1.52E-09 |
| TLN2 | -0.2757913 | 1.57E-10 | 1.52E-09 |
| MEIS3 | -0.2757863 | 1.57E-10 | 1.52E-09 |
| MTX1 | 0.2757769 | 1.57E-10 | 1.52E-09 |
| CDH13 | -0.2756886 | 1.60E-10 | 1.54E-09 |
| NIPAL2 | -0.2756754 | 1.60E-10 | 1.55E-09 |
| RDBP | 0.2756405 | 1.61E-10 | 1.55E-09 |
| SAP18 | 0.2755315 | 1.64E-10 | 1.58E-09 |
| H6PD | -0.2754562 | 1.66E-10 | 1.60E-09 |
| N6AMT2 | 0.2754476 | 1.66E-10 | 1.60E-09 |
| F12 | 0.2754192 | 1.67E-10 | 1.61E-09 |
| PGCP | -0.2753811 | 1.68E-10 | 1.61E-09 |
| TMEM87B | -0.2753542 | 1.68E-10 | 1.62E-09 |
| RPL6 | 0.2752901 | 1.70E-10 | 1.64E-09 |
| RPS18 | 0.2752868 | 1.70E-10 | 1.64E-09 |
| GCFC1 | 0.2752835 | 1.70E-10 | 1.64E-09 |
| C5orf41 | -0.2752733 | 1.70E-10 | 1.64E-09 |
| THBS3 | -0.2751833 | 1.73E-10 | 1.66E-09 |
| EVI5L | -0.2751782 | 1.73E-10 | 1.66E-09 |
| C2orf79 | 0.2751679 | 1.73E-10 | 1.66E-09 |
| LIMK1 | -0.275076 | 1.76E-10 | 1.69E-09 |
| WDR83 | 0.2750706 | 1.76E-10 | 1.69E-09 |
| CLASP1 | -0.2750702 | 1.76E-10 | 1.69E-09 |
| NCOA1 | -0.2750652 | 1.76E-10 | 1.69E-09 |
| NGDN | 0.275009 | 1.78E-10 | 1.70E-09 |
| NSUN6 | 0.2749384 | 1.80E-10 | 1.72E-09 |
| CRIM1 | -0.2749272 | 1.80E-10 | 1.72E-09 |
| PCNX | -0.2749214 | 1.80E-10 | 1.72E-09 |
| CCDC56 | 0.2749119 | 1.80E-10 | 1.72E-09 |
| PCDHB19P | -0.2748009 | 1.84E-10 | 1.75E-09 |
| ZNF778 | -0.2747973 | 1.84E-10 | 1.75E-09 |
| APOD | -0.2747377 | 1.85E-10 | 1.77E-09 |
| PLCB1 | -0.2747317 | 1.86E-10 | 1.77E-09 |
| DKK3 | -0.2747222 | 1.86E-10 | 1.77E-09 |
| ETV3L | -0.2746611 | 1.88E-10 | 1.79E-09 |
| PI4KA | -0.2745149 | 1.92E-10 | 1.83E-09 |
| ATMIN | -0.2745059 | 1.92E-10 | 1.83E-09 |
| ZNF835 | -0.2744829 | 1.93E-10 | 1.84E-09 |
| RPL7A | 0.274367 | 1.97E-10 | 1.87E-09 |
| PUM1 | -0.2743468 | 1.97E-10 | 1.87E-09 |
| C3orf37 | 0.2742903 | 1.99E-10 | 1.89E-09 |
| UBR5 | -0.2742834 | 1.99E-10 | 1.89E-09 |
| MYO5A | -0.2742272 | 2.01E-10 | 1.91E-09 |
| DENR | 0.2742193 | 2.01E-10 | 1.91E-09 |
| KLF9 | -0.2741725 | 2.03E-10 | 1.92E-09 |
| MURC | -0.2741618 | 2.03E-10 | 1.92E-09 |
| TECPR1 | -0.2741606 | 2.03E-10 | 1.92E-09 |
| ENTPD7 | -0.2740634 | 2.06E-10 | 1.95E-09 |
| FAM124B | -0.274041 | 2.07E-10 | 1.96E-09 |
| ARHGEF10L | -0.2739753 | 2.09E-10 | 1.98E-09 |
| PPIL1 | 0.2739634 | 2.09E-10 | 1.98E-09 |
| C8orf40 | 0.2739446 | 2.10E-10 | 1.99E-09 |
| ASNA1 | 0.2738895 | 2.12E-10 | 2.00E-09 |
| C7orf40 | 0.2738573 | 2.13E-10 | 2.01E-09 |
| SPDYA | 0.2738301 | 2.14E-10 | 2.02E-09 |
| JMJD1C | -0.2738229 | 2.14E-10 | 2.02E-09 |
| GOLGA3 | -0.2738186 | 2.14E-10 | 2.02E-09 |
| GSN | -0.2738021 | 2.15E-10 | 2.02E-09 |
| NSMCE2 | 0.2737869 | 2.15E-10 | 2.03E-09 |
| COPZ1 | 0.2736736 | 2.19E-10 | 2.06E-09 |
| QTRT1 | 0.2736566 | 2.20E-10 | 2.07E-09 |
| DNMT1 | 0.2736377 | 2.20E-10 | 2.07E-09 |
| PMS1 | 0.2736208 | 2.21E-10 | 2.08E-09 |
| ABCG1 | -0.2735714 | 2.23E-10 | 2.09E-09 |
| GLA | 0.2735101 | 2.25E-10 | 2.11E-09 |
| RGPD3 | -0.2734805 | 2.26E-10 | 2.12E-09 |
| BUD31 | 0.2734589 | 2.27E-10 | 2.13E-09 |
| IGFL3 | -0.2734298 | 2.28E-10 | 2.14E-09 |
| PFDN5 | 0.2734132 | 2.28E-10 | 2.14E-09 |
| CACHD1 | -0.2734068 | 2.29E-10 | 2.14E-09 |
| SDC2 | -0.2733976 | 2.29E-10 | 2.14E-09 |
| SUMO1 | 0.2733766 | 2.30E-10 | 2.15E-09 |
| STK40 | -0.2733748 | 2.30E-10 | 2.15E-09 |
| IMMT | 0.2733258 | 2.31E-10 | 2.17E-09 |
| FREM2 | -0.273303 | 2.32E-10 | 2.17E-09 |
| RUVBL2 | 0.2732234 | 2.35E-10 | 2.20E-09 |
| C10orf72 | -0.273087 | 2.40E-10 | 2.24E-09 |
| OBSL1 | -0.2730343 | 2.42E-10 | 2.26E-09 |
| IGFBP5 | -0.2729271 | 2.46E-10 | 2.30E-09 |
| PCDHGB5 | -0.2728946 | 2.48E-10 | 2.31E-09 |
| ITPKA | 0.2728722 | 2.48E-10 | 2.32E-09 |
| NDUFB8 | 0.2728677 | 2.49E-10 | 2.32E-09 |
| WDYHV1 | 0.2727855 | 2.52E-10 | 2.35E-09 |
| TSPYL5 | -0.2727713 | 2.52E-10 | 2.35E-09 |
| BRD8 | 0.2727317 | 2.54E-10 | 2.36E-09 |
| BRP44 | 0.272726 | 2.54E-10 | 2.37E-09 |
| C7orf47 | 0.272689 | 2.56E-10 | 2.38E-09 |
| CORT | 0.2726785 | 2.56E-10 | 2.38E-09 |
| TRPV3 | -0.2726327 | 2.58E-10 | 2.40E-09 |
| LRP12 | -0.2725814 | 2.60E-10 | 2.41E-09 |
| SLC30A1 | -0.2725346 | 2.62E-10 | 2.43E-09 |
| SNAPIN | 0.2725152 | 2.63E-10 | 2.44E-09 |
| XRCC6BP1 | 0.2725108 | 2.63E-10 | 2.44E-09 |
| UNC93A | -0.2724688 | 2.65E-10 | 2.45E-09 |
| SYTL5 | -0.2724298 | 2.66E-10 | 2.47E-09 |
| GEFT | -0.2724225 | 2.67E-10 | 2.47E-09 |
| BMP8A | -0.2723604 | 2.69E-10 | 2.49E-09 |
| DOPEY2 | -0.2723194 | 2.71E-10 | 2.51E-09 |
| CLIP3 | -0.2722753 | 2.73E-10 | 2.52E-09 |
| STRN | -0.2721104 | 2.80E-10 | 2.59E-09 |
| ARHGAP20 | -0.2720786 | 2.81E-10 | 2.60E-09 |
| LRRC45 | 0.272037 | 2.83E-10 | 2.61E-09 |
| PPHLN1 | 0.2720317 | 2.83E-10 | 2.62E-09 |
| RIN2 | -0.2719735 | 2.86E-10 | 2.64E-09 |
| MOCS1 | -0.2719288 | 2.88E-10 | 2.65E-09 |
| RPS4X | 0.271906 | 2.89E-10 | 2.66E-09 |
| ACTR6 | 0.2716869 | 2.99E-10 | 2.75E-09 |
| SYNPO2L | -0.2715144 | 3.07E-10 | 2.83E-09 |
| GNL2 | 0.2714634 | 3.09E-10 | 2.85E-09 |
| DPP4 | -0.271412 | 3.12E-10 | 2.87E-09 |
| CUL5 | -0.2713829 | 3.13E-10 | 2.88E-09 |
| VPS72 | 0.2713712 | 3.14E-10 | 2.89E-09 |
| ELN | -0.2713195 | 3.16E-10 | 2.91E-09 |
| ZNF843 | -0.271313 | 3.17E-10 | 2.91E-09 |
| DOCK6 | -0.2712628 | 3.19E-10 | 2.93E-09 |
| RIMBP2 | -0.2712176 | 3.21E-10 | 2.95E-09 |
| NANS | 0.2711841 | 3.23E-10 | 2.96E-09 |
| IL17RA | -0.2711719 | 3.24E-10 | 2.97E-09 |
| PAK1IP1 | 0.2709948 | 3.33E-10 | 3.05E-09 |
| DGUOK | 0.2709224 | 3.36E-10 | 3.08E-09 |
| GTF3C6 | 0.270905 | 3.37E-10 | 3.09E-09 |
| ZCCHC14 | -0.2708874 | 3.38E-10 | 3.10E-09 |
| MAP4K4 | -0.2708624 | 3.40E-10 | 3.11E-09 |
| HDAC3 | 0.270811 | 3.42E-10 | 3.13E-09 |
| DHODH | 0.2707531 | 3.45E-10 | 3.16E-09 |
| RAET1L | -0.270752 | 3.46E-10 | 3.16E-09 |
| NFATC4 | -0.2707196 | 3.47E-10 | 3.17E-09 |
| FBLIM1 | -0.2706863 | 3.49E-10 | 3.19E-09 |
| GSTM5 | -0.270662 | 3.50E-10 | 3.20E-09 |
| GTF2A1 | -0.2706564 | 3.51E-10 | 3.20E-09 |
| ZDHHC9 | -0.270651 | 3.51E-10 | 3.20E-09 |
| CXorf36 | -0.2706402 | 3.52E-10 | 3.20E-09 |
| BRIP1 | 0.27064 | 3.52E-10 | 3.20E-09 |
| PROX1 | -0.2706084 | 3.53E-10 | 3.22E-09 |
| GLOD4 | 0.2705772 | 3.55E-10 | 3.23E-09 |
| MIR17HG | 0.2705527 | 3.56E-10 | 3.24E-09 |
| ATF7 | -0.2705449 | 3.57E-10 | 3.24E-09 |
| HNRNPK | 0.2705104 | 3.59E-10 | 3.26E-09 |
| ING2 | 0.270385 | 3.66E-10 | 3.32E-09 |
| RPS2 | 0.2703266 | 3.69E-10 | 3.35E-09 |
| AGT | -0.2703229 | 3.69E-10 | 3.35E-09 |
| DPM1 | 0.2703159 | 3.70E-10 | 3.35E-09 |
| GBX1 | 0.270294 | 3.71E-10 | 3.36E-09 |
| PCDHGC5 | -0.2702701 | 3.72E-10 | 3.37E-09 |
| PITPNA | -0.2702197 | 3.75E-10 | 3.40E-09 |
| GCDH | 0.2701065 | 3.82E-10 | 3.46E-09 |
| SSBP1 | 0.2701025 | 3.82E-10 | 3.46E-09 |
| HSPA12B | -0.2700664 | 3.84E-10 | 3.48E-09 |
| RUNX2 | -0.2700258 | 3.87E-10 | 3.50E-09 |
| APBA1 | -0.2700129 | 3.87E-10 | 3.50E-09 |
| SMURF1 | -0.2700048 | 3.88E-10 | 3.50E-09 |
| TGM5 | -0.2699012 | 3.94E-10 | 3.56E-09 |
| ARSE | -0.2697358 | 4.04E-10 | 3.65E-09 |
| PMAIP1 | 0.2696654 | 4.09E-10 | 3.69E-09 |
| PPARA | -0.2695976 | 4.13E-10 | 3.72E-09 |
| LIPN | -0.2695685 | 4.15E-10 | 3.74E-09 |
| TMEM201 | 0.2695587 | 4.15E-10 | 3.74E-09 |
| POLR2I | 0.2695137 | 4.18E-10 | 3.77E-09 |
| PRSS23 | -0.2694954 | 4.20E-10 | 3.78E-09 |
| OLFML3 | -0.2694927 | 4.20E-10 | 3.78E-09 |
| SLC38A4 | -0.2694851 | 4.20E-10 | 3.78E-09 |
| DSCR6 | 0.2694582 | 4.22E-10 | 3.79E-09 |
| GFOD1 | -0.2694139 | 4.25E-10 | 3.82E-09 |
| MTPAP | 0.2693591 | 4.28E-10 | 3.85E-09 |
| MTHFD2 | 0.2692795 | 4.34E-10 | 3.89E-09 |
| NOD2 | -0.2692655 | 4.35E-10 | 3.90E-09 |
| CXXC5 | -0.2692295 | 4.37E-10 | 3.92E-09 |
| CDC42BPA | -0.2692183 | 4.38E-10 | 3.93E-09 |
| FER1L6 | -0.2692114 | 4.38E-10 | 3.93E-09 |
| ATP5H | 0.2691742 | 4.41E-10 | 3.95E-09 |
| LTBP3 | -0.2691581 | 4.42E-10 | 3.96E-09 |
| SETBP1 | -0.2690384 | 4.50E-10 | 4.03E-09 |
| PPWD1 | 0.2690279 | 4.51E-10 | 4.03E-09 |
| EMX2 | -0.2689318 | 4.58E-10 | 4.09E-09 |
| FAM38A | -0.2689167 | 4.59E-10 | 4.10E-09 |
| LATS1 | -0.2689048 | 4.59E-10 | 4.10E-09 |
| MRPS14 | 0.2687617 | 4.70E-10 | 4.19E-09 |
| OPCML | -0.268761 | 4.70E-10 | 4.19E-09 |
| SYCP2 | 0.268748 | 4.71E-10 | 4.20E-09 |
| SPATA13 | -0.2686807 | 4.76E-10 | 4.24E-09 |
| MRC2 | -0.2686619 | 4.77E-10 | 4.25E-09 |
| COPG | -0.2685997 | 4.81E-10 | 4.29E-09 |
| NIT2 | 0.2685647 | 4.84E-10 | 4.31E-09 |
| PPAT | 0.2685462 | 4.85E-10 | 4.32E-09 |
| ZNF366 | -0.2684251 | 4.95E-10 | 4.40E-09 |
| GIYD2 | 0.26841 | 4.96E-10 | 4.41E-09 |
| SYTL2 | -0.2683409 | 5.01E-10 | 4.45E-09 |
| MRPL14 | 0.2683193 | 5.03E-10 | 4.47E-09 |
| ATP5G3 | 0.2682565 | 5.07E-10 | 4.51E-09 |
| FAM83B | -0.2682396 | 5.09E-10 | 4.52E-09 |
| PPID | 0.2682064 | 5.11E-10 | 4.54E-09 |
| EGF | -0.2682041 | 5.12E-10 | 4.54E-09 |
| EFR3B | -0.2681182 | 5.18E-10 | 4.60E-09 |
| C17orf89 | 0.2681086 | 5.19E-10 | 4.60E-09 |
| ZNF576 | 0.2681035 | 5.20E-10 | 4.60E-09 |
| KRT75 | -0.2680681 | 5.22E-10 | 4.63E-09 |
| ZC3HC1 | 0.2680374 | 5.25E-10 | 4.65E-09 |
| COL4A2 | -0.2680093 | 5.27E-10 | 4.66E-09 |
| C1S | -0.2679948 | 5.28E-10 | 4.67E-09 |
| TCHHL1 | -0.2679826 | 5.29E-10 | 4.68E-09 |
| TGFB1 | -0.2679798 | 5.29E-10 | 4.68E-09 |
| DCTN1 | -0.2679291 | 5.34E-10 | 4.71E-09 |
| SRPX | -0.2679278 | 5.34E-10 | 4.71E-09 |
| ATXN1L | -0.2679058 | 5.35E-10 | 4.72E-09 |
| RANBP2 | -0.2679022 | 5.36E-10 | 4.72E-09 |
| PSKH1 | -0.2679008 | 5.36E-10 | 4.72E-09 |
| DSTYK | -0.267845 | 5.40E-10 | 4.76E-09 |
| RGL1 | -0.2678189 | 5.43E-10 | 4.78E-09 |
| ACBD3 | -0.2677923 | 5.45E-10 | 4.80E-09 |
| PFDN4 | 0.2677722 | 5.47E-10 | 4.81E-09 |
| CLCN3 | -0.2676132 | 5.60E-10 | 4.93E-09 |
| DCLK2 | -0.2676079 | 5.60E-10 | 4.93E-09 |
| RWDD4A | 0.2675972 | 5.61E-10 | 4.93E-09 |
| RELN | -0.2675763 | 5.63E-10 | 4.95E-09 |
| FAM8A1 | -0.2675484 | 5.66E-10 | 4.97E-09 |
| COX11 | 0.267427 | 5.76E-10 | 5.06E-09 |
| SLC43A2 | -0.2673261 | 5.85E-10 | 5.13E-09 |
| HIST1H3I | 0.2673092 | 5.87E-10 | 5.15E-09 |
| MUSK | -0.2672048 | 5.96E-10 | 5.23E-09 |
| TAGLN | -0.2672018 | 5.96E-10 | 5.23E-09 |
| BCAT2 | 0.2671174 | 6.04E-10 | 5.29E-09 |
| GAS5 | 0.2670926 | 6.06E-10 | 5.31E-09 |
| MAZ | 0.2670903 | 6.07E-10 | 5.31E-09 |
| GIPC3 | -0.2670478 | 6.11E-10 | 5.34E-09 |
| GCH1 | 0.2668859 | 6.26E-10 | 5.47E-09 |
| RAB3IL1 | -0.2668575 | 6.28E-10 | 5.49E-09 |
| FAM128B | 0.2668323 | 6.31E-10 | 5.51E-09 |
| SLC6A2 | -0.2668067 | 6.33E-10 | 5.53E-09 |
| PCDHB7 | -0.2667924 | 6.35E-10 | 5.54E-09 |
| ARHGAP11B | 0.2667893 | 6.35E-10 | 5.54E-09 |
| TNS3 | -0.266745 | 6.39E-10 | 5.58E-09 |
| ZKSCAN1 | -0.2667203 | 6.42E-10 | 5.59E-09 |
| FXYD1 | -0.2667037 | 6.43E-10 | 5.61E-09 |
| NUBP1 | 0.2666604 | 6.48E-10 | 5.64E-09 |
| RBMS1 | -0.2666168 | 6.52E-10 | 5.68E-09 |
| DLEU2L | 0.266546 | 6.59E-10 | 5.74E-09 |
| IFI27L1 | 0.266482 | 6.65E-10 | 5.79E-09 |
| PNPLA1 | -0.2664803 | 6.66E-10 | 5.79E-09 |
| TOMM22 | 0.266463 | 6.67E-10 | 5.80E-09 |
| ST6GAL2 | -0.2664055 | 6.73E-10 | 5.85E-09 |
| TRMT112 | 0.2663569 | 6.78E-10 | 5.89E-09 |
| TRAF6 | -0.2663203 | 6.82E-10 | 5.92E-09 |
| C14orf145 | 0.2663091 | 6.83E-10 | 5.93E-09 |
| TEK | -0.2662488 | 6.89E-10 | 5.98E-09 |
| MMP10 | -0.2662398 | 6.90E-10 | 5.99E-09 |
| KTI12 | 0.2660314 | 7.13E-10 | 6.18E-09 |
| POLR2J | 0.2660209 | 7.14E-10 | 6.18E-09 |
| HPDL | 0.2659733 | 7.19E-10 | 6.22E-09 |
| WDR8 | 0.265955 | 7.21E-10 | 6.24E-09 |
| MED27 | 0.2659458 | 7.22E-10 | 6.25E-09 |
| ARHGAP28 | -0.2659415 | 7.22E-10 | 6.25E-09 |
| MRPS26 | 0.2659277 | 7.24E-10 | 6.26E-09 |
| ECHS1 | 0.2659127 | 7.26E-10 | 6.27E-09 |
| RTN4 | -0.2658753 | 7.30E-10 | 6.30E-09 |
| NLRX1 | -0.2658727 | 7.30E-10 | 6.30E-09 |
| CYR61 | -0.2658189 | 7.36E-10 | 6.35E-09 |
| ROCK1 | -0.2658113 | 7.37E-10 | 6.35E-09 |
| VASH1 | -0.2657924 | 7.39E-10 | 6.37E-09 |
| JARID2 | -0.265703 | 7.49E-10 | 6.45E-09 |
| PTPRD | -0.2656616 | 7.54E-10 | 6.49E-09 |
| SMC1B | 0.2656582 | 7.54E-10 | 6.49E-09 |
| FARP2 | -0.2656558 | 7.54E-10 | 6.49E-09 |
| OGN | -0.2655627 | 7.65E-10 | 6.58E-09 |
| PPIE | 0.2654474 | 7.79E-10 | 6.70E-09 |
| NBAS | -0.2654006 | 7.84E-10 | 6.74E-09 |
| PCDHB16 | -0.2653756 | 7.87E-10 | 6.76E-09 |
| PAFAH1B1 | -0.2653739 | 7.87E-10 | 6.76E-09 |
| RPL34 | 0.2653478 | 7.90E-10 | 6.78E-09 |
| MFSD9 | -0.2653473 | 7.91E-10 | 6.78E-09 |
| EMG1 | 0.2653375 | 7.92E-10 | 6.79E-09 |
| FNTA | 0.2652865 | 7.98E-10 | 6.84E-09 |
| COPS6 | 0.2652788 | 7.99E-10 | 6.85E-09 |
| ANO10 | -0.2652393 | 8.04E-10 | 6.88E-09 |
| NDUFS5 | 0.2651746 | 8.11E-10 | 6.95E-09 |
| AGER | 0.2651472 | 8.15E-10 | 6.97E-09 |
| TOR2A | 0.2650801 | 8.23E-10 | 7.04E-09 |
| WWTR1 | -0.2649941 | 8.34E-10 | 7.13E-09 |
| RRP1 | 0.2649681 | 8.37E-10 | 7.15E-09 |
| EBNA1BP2 | 0.2649679 | 8.37E-10 | 7.15E-09 |
| MRPL2 | 0.2649408 | 8.41E-10 | 7.18E-09 |
| ABCG4 | -0.2649152 | 8.44E-10 | 7.20E-09 |
| ADAMTS5 | -0.2649105 | 8.45E-10 | 7.21E-09 |
| ARX | -0.2648933 | 8.47E-10 | 7.22E-09 |
| PRPS1 | 0.2648699 | 8.50E-10 | 7.25E-09 |
| IFRD2 | 0.2648566 | 8.51E-10 | 7.26E-09 |
| MRAS | -0.2648333 | 8.54E-10 | 7.28E-09 |
| MAFB | -0.2647751 | 8.62E-10 | 7.34E-09 |
| PSMB2 | 0.2646547 | 8.78E-10 | 7.47E-09 |
| KLF7 | -0.2646087 | 8.84E-10 | 7.52E-09 |
| EGFR | -0.264462 | 9.04E-10 | 7.68E-09 |
| MRPL32 | 0.2644613 | 9.04E-10 | 7.68E-09 |
| EEF1B2 | 0.2644543 | 9.05E-10 | 7.69E-09 |
| HIST1H3F | 0.2644437 | 9.06E-10 | 7.70E-09 |
| CAMK2A | -0.2644401 | 9.07E-10 | 7.70E-09 |
| HIST1H4C | 0.2644263 | 9.09E-10 | 7.71E-09 |
| CCT7 | 0.264295 | 9.27E-10 | 7.86E-09 |
| HHIPL1 | -0.264268 | 9.31E-10 | 7.89E-09 |
| OSBPL5 | -0.2642317 | 9.36E-10 | 7.93E-09 |
| IDH3A | 0.2642119 | 9.38E-10 | 7.95E-09 |
| TOB2 | -0.2641198 | 9.52E-10 | 8.06E-09 |
| MLH1 | 0.2641146 | 9.52E-10 | 8.06E-09 |
| TIMM17A | 0.2640994 | 9.55E-10 | 8.08E-09 |
| FAM24B | 0.2639973 | 9.69E-10 | 8.20E-09 |
| PSMA6 | 0.2639889 | 9.71E-10 | 8.21E-09 |
| RPS9 | 0.2639703 | 9.73E-10 | 8.23E-09 |
| RPP40 | 0.2639498 | 9.76E-10 | 8.25E-09 |
| NUDC | 0.2638263 | 9.95E-10 | 8.40E-09 |
| CEP70 | 0.2638096 | 9.97E-10 | 8.42E-09 |
| RAD9B | 0.2637906 | 1.00E-09 | 8.44E-09 |
| PRKAB2 | -0.2637658 | 1.00E-09 | 8.46E-09 |
| ADRA2A | -0.2637422 | 1.01E-09 | 8.49E-09 |
| DSG4 | -0.2636373 | 1.02E-09 | 8.62E-09 |
| ZSWIM4 | -0.2636249 | 1.03E-09 | 8.63E-09 |
| NOTCH4 | -0.2635813 | 1.03E-09 | 8.69E-09 |
| C9orf156 | 0.2635355 | 1.04E-09 | 8.74E-09 |
| TM6SF2 | -0.2634428 | 1.05E-09 | 8.86E-09 |
| SGCA | -0.2634377 | 1.05E-09 | 8.87E-09 |
| SOBP | -0.2634049 | 1.06E-09 | 8.91E-09 |
| FRMD3 | -0.2633167 | 1.07E-09 | 9.02E-09 |
| FKBP9L | -0.2632642 | 1.08E-09 | 9.09E-09 |
| CSGALNACT2 | -0.263246 | 1.09E-09 | 9.11E-09 |
| NUP43 | 0.2632407 | 1.09E-09 | 9.11E-09 |
| RPS19 | 0.2631709 | 1.10E-09 | 9.21E-09 |
| HIST1H3C | 0.2631675 | 1.10E-09 | 9.21E-09 |
| C6orf89 | -0.2631314 | 1.10E-09 | 9.25E-09 |
| LIAS | 0.263061 | 1.12E-09 | 9.35E-09 |
| SLC30A4 | -0.2630214 | 1.12E-09 | 9.40E-09 |
| ENOPH1 | 0.2630172 | 1.12E-09 | 9.40E-09 |
| ZCCHC5 | -0.2630112 | 1.12E-09 | 9.41E-09 |
| C17orf39 | -0.2629731 | 1.13E-09 | 9.45E-09 |
| GNL3 | 0.2629722 | 1.13E-09 | 9.45E-09 |
| GPRC5B | -0.2628183 | 1.16E-09 | 9.67E-09 |
| PNLIPRP3 | -0.2627556 | 1.17E-09 | 9.76E-09 |
| MED13 | -0.2627453 | 1.17E-09 | 9.77E-09 |
| MYL9 | -0.2626935 | 1.18E-09 | 9.84E-09 |
| VIPR1 | -0.2626926 | 1.18E-09 | 9.84E-09 |
| UTP11L | 0.2626634 | 1.18E-09 | 9.88E-09 |
| PSMB7 | 0.2625792 | 1.20E-09 | 1.00E-08 |
| MED6 | 0.2625486 | 1.20E-09 | 1.00E-08 |
| PAX7 | -0.2625355 | 1.21E-09 | 1.01E-08 |
| FLJ16779 | -0.26253 | 1.21E-09 | 1.01E-08 |
| HBXIP | 0.2624991 | 1.21E-09 | 1.01E-08 |
| RASL12 | -0.2624973 | 1.21E-09 | 1.01E-08 |
| MID2 | -0.2624579 | 1.22E-09 | 1.02E-08 |
| ZNF516 | -0.2624538 | 1.22E-09 | 1.02E-08 |
| C17orf95 | 0.2623909 | 1.23E-09 | 1.02E-08 |
| ITGA9 | -0.2623826 | 1.24E-09 | 1.03E-08 |
| DAAM1 | -0.2623281 | 1.25E-09 | 1.03E-08 |
| GNS | -0.2623096 | 1.25E-09 | 1.04E-08 |
| CAMK2N2 | 0.2623064 | 1.25E-09 | 1.04E-08 |
| ROR1 | -0.2622664 | 1.26E-09 | 1.04E-08 |
| SF3A2 | 0.2622493 | 1.26E-09 | 1.04E-08 |
| HIST1H2BL | 0.2621544 | 1.28E-09 | 1.06E-08 |
| SPRR2G | -0.2621034 | 1.29E-09 | 1.07E-08 |
| NCKAP1 | -0.2620919 | 1.29E-09 | 1.07E-08 |
| MRC1 | -0.2620873 | 1.29E-09 | 1.07E-08 |
| SC5DL | -0.2620828 | 1.29E-09 | 1.07E-08 |
| PCDHGB4 | -0.2620687 | 1.29E-09 | 1.07E-08 |
| LAMP1 | -0.2619754 | 1.31E-09 | 1.08E-08 |
| MAN1A2 | -0.2619695 | 1.31E-09 | 1.09E-08 |
| IGF2AS | -0.2619568 | 1.32E-09 | 1.09E-08 |
| TP53INP2 | -0.2618916 | 1.33E-09 | 1.10E-08 |
| MOCS2 | 0.2618831 | 1.33E-09 | 1.10E-08 |
| MGAT5 | -0.2618714 | 1.33E-09 | 1.10E-08 |
| C21orf59 | 0.2618481 | 1.34E-09 | 1.10E-08 |
| DNAJC7 | 0.2618424 | 1.34E-09 | 1.10E-08 |
| CATSPER3 | 0.261777 | 1.35E-09 | 1.11E-08 |
| KIAA0319L | -0.261671 | 1.37E-09 | 1.13E-08 |
| SGCG | -0.2616667 | 1.37E-09 | 1.13E-08 |
| SDR9C7 | -0.2616343 | 1.38E-09 | 1.14E-08 |
| CREB3L4 | 0.2616116 | 1.39E-09 | 1.14E-08 |
| RPL15 | 0.2615676 | 1.40E-09 | 1.15E-08 |
| SOD3 | -0.2615468 | 1.40E-09 | 1.15E-08 |
| MRPL43 | 0.2615106 | 1.41E-09 | 1.16E-08 |
| SUSD2 | -0.261508 | 1.41E-09 | 1.16E-08 |
| LOC647946 | 0.2614137 | 1.43E-09 | 1.17E-08 |
| IER3IP1 | 0.2614106 | 1.43E-09 | 1.17E-08 |
| ITPR2 | -0.2613986 | 1.43E-09 | 1.17E-08 |
| COX6C | 0.2613094 | 1.45E-09 | 1.19E-08 |
| C3orf10 | 0.2613076 | 1.45E-09 | 1.19E-08 |
| ARFGEF1 | -0.2612641 | 1.46E-09 | 1.20E-08 |
| NCOA2 | -0.2612049 | 1.47E-09 | 1.21E-08 |
| LRP4 | -0.2611983 | 1.47E-09 | 1.21E-08 |
| C12orf41 | 0.2611936 | 1.47E-09 | 1.21E-08 |
| BHMT2 | -0.2611615 | 1.48E-09 | 1.21E-08 |
| ARPC3 | 0.2611073 | 1.49E-09 | 1.22E-08 |
| ELK3 | -0.2610653 | 1.50E-09 | 1.23E-08 |
| RPS29 | 0.2610582 | 1.50E-09 | 1.23E-08 |
| CPSF6 | 0.2609847 | 1.52E-09 | 1.24E-08 |
| MEGF8 | -0.2609569 | 1.53E-09 | 1.25E-08 |
| S100A7A | -0.2609441 | 1.53E-09 | 1.25E-08 |
| CPA3 | -0.260893 | 1.54E-09 | 1.26E-08 |
| HTR2A | -0.2608484 | 1.55E-09 | 1.27E-08 |
| AOX1 | -0.2608142 | 1.56E-09 | 1.27E-08 |
| ZNF486 | -0.2607888 | 1.57E-09 | 1.28E-08 |
| SEMA6D | -0.2607647 | 1.57E-09 | 1.28E-08 |
| LDOC1L | -0.2607531 | 1.57E-09 | 1.28E-08 |
| CYYR1 | -0.260703 | 1.59E-09 | 1.29E-08 |
| PPIAL4C | 0.2606786 | 1.59E-09 | 1.29E-08 |
| NOL7 | 0.2606092 | 1.61E-09 | 1.31E-08 |
| TMEM90B | -0.2605846 | 1.61E-09 | 1.31E-08 |
| MYBL1 | 0.2605684 | 1.62E-09 | 1.31E-08 |
| RPAIN | 0.260566 | 1.62E-09 | 1.31E-08 |
| HIST1H3J | 0.2605646 | 1.62E-09 | 1.31E-08 |
| MAGI1 | -0.2605016 | 1.63E-09 | 1.33E-08 |
| MRPL45 | 0.2604763 | 1.64E-09 | 1.33E-08 |
| CRK | -0.2603645 | 1.67E-09 | 1.35E-08 |
| PPP1R9B | -0.2603586 | 1.67E-09 | 1.35E-08 |
| FNDC3B | -0.2603502 | 1.67E-09 | 1.35E-08 |
| SLC7A8 | -0.2603266 | 1.68E-09 | 1.36E-08 |
| THPO | -0.2603109 | 1.68E-09 | 1.36E-08 |
| KIAA0284 | -0.2601815 | 1.71E-09 | 1.39E-08 |
| DOK4 | -0.2601665 | 1.72E-09 | 1.39E-08 |
| KLHL38 | -0.2601377 | 1.73E-09 | 1.39E-08 |
| ZCRB1 | 0.2601327 | 1.73E-09 | 1.39E-08 |
| SBF2 | -0.2601044 | 1.73E-09 | 1.40E-08 |
| SCARB2 | -0.2600944 | 1.74E-09 | 1.40E-08 |
| KLF1 | 0.2600786 | 1.74E-09 | 1.40E-08 |
| CLEC11A | -0.2599024 | 1.79E-09 | 1.44E-08 |
| CHSY3 | -0.2598489 | 1.80E-09 | 1.45E-08 |
| PLCL1 | -0.2598482 | 1.80E-09 | 1.45E-08 |
| MEA1 | 0.259827 | 1.81E-09 | 1.46E-08 |
| UCHL5 | 0.2598239 | 1.81E-09 | 1.46E-08 |
| MBOAT1 | 0.2597548 | 1.83E-09 | 1.47E-08 |
| EPB41L3 | -0.2596864 | 1.84E-09 | 1.48E-08 |
| PARVA | -0.2595829 | 1.87E-09 | 1.51E-08 |
| CDH8 | -0.2595295 | 1.89E-09 | 1.52E-08 |
| NKAPL | -0.2595117 | 1.89E-09 | 1.52E-08 |
| KPRP | -0.2594971 | 1.90E-09 | 1.52E-08 |
| USF1 | 0.2593729 | 1.93E-09 | 1.55E-08 |
| SLIT2 | -0.2593445 | 1.94E-09 | 1.56E-08 |
| MTCP1NB | 0.2593086 | 1.95E-09 | 1.57E-08 |
| KDM4B | -0.2592638 | 1.96E-09 | 1.57E-08 |
| SH3RF1 | -0.259233 | 1.97E-09 | 1.58E-08 |
| TAF11 | 0.2592011 | 1.98E-09 | 1.59E-08 |
| ARMCX2 | -0.2591927 | 1.98E-09 | 1.59E-08 |
| UMPS | 0.2591435 | 2.00E-09 | 1.60E-08 |
| RPL10A | 0.2591048 | 2.01E-09 | 1.61E-08 |
| IMPDH2 | 0.2590579 | 2.02E-09 | 1.62E-08 |
| LY6K | 0.2590501 | 2.03E-09 | 1.62E-08 |
| PMPCB | 0.2590495 | 2.03E-09 | 1.62E-08 |
| MED7 | 0.2590093 | 2.04E-09 | 1.63E-08 |
| RERE | -0.2590028 | 2.04E-09 | 1.63E-08 |
| CACNA1S | -0.2590002 | 2.04E-09 | 1.63E-08 |
| PCDHB11 | -0.2589961 | 2.04E-09 | 1.63E-08 |
| FAU | 0.2589148 | 2.07E-09 | 1.65E-08 |
| ENSA | 0.2589146 | 2.07E-09 | 1.65E-08 |
| NCOA3 | -0.2589004 | 2.07E-09 | 1.65E-08 |
| FAM43B | -0.2588497 | 2.09E-09 | 1.66E-08 |
| SYT3 | -0.2588325 | 2.09E-09 | 1.67E-08 |
| TMEM119 | -0.2588276 | 2.09E-09 | 1.67E-08 |
| SORT1 | -0.2588088 | 2.10E-09 | 1.67E-08 |
| MCTS1 | 0.2587779 | 2.11E-09 | 1.68E-08 |
| CASP3 | 0.2586921 | 2.14E-09 | 1.70E-08 |
| MYOCD | -0.2586768 | 2.14E-09 | 1.70E-08 |
| BAHCC1 | -0.2586633 | 2.15E-09 | 1.70E-08 |
| PRPF19 | 0.2586514 | 2.15E-09 | 1.71E-08 |
| SYNRG | -0.2586439 | 2.15E-09 | 1.71E-08 |
| TACC2 | -0.258569 | 2.18E-09 | 1.73E-08 |
| 8-Mar | -0.2585575 | 2.18E-09 | 1.73E-08 |
| ADAT2 | 0.2585512 | 2.18E-09 | 1.73E-08 |
| ATP6V0A4 | -0.2584297 | 2.22E-09 | 1.76E-08 |
| EPHA3 | -0.258393 | 2.23E-09 | 1.77E-08 |
| GLI3 | -0.2583639 | 2.24E-09 | 1.78E-08 |
| STAR | 0.2583387 | 2.25E-09 | 1.78E-08 |
| UBR2 | -0.2583373 | 2.25E-09 | 1.78E-08 |
| COIL | 0.258327 | 2.25E-09 | 1.78E-08 |
| MRPL54 | 0.258326 | 2.25E-09 | 1.78E-08 |
| KLHL31 | -0.2582352 | 2.28E-09 | 1.81E-08 |
| ZNF544 | 0.2582202 | 2.29E-09 | 1.81E-08 |
| HSCB | 0.2581975 | 2.30E-09 | 1.82E-08 |
| MXD4 | -0.2581904 | 2.30E-09 | 1.82E-08 |
| DMC1 | 0.2581764 | 2.30E-09 | 1.82E-08 |
| WASL | -0.2581418 | 2.32E-09 | 1.83E-08 |
| MYO18B | -0.2581108 | 2.33E-09 | 1.84E-08 |
| RPL22 | 0.2580959 | 2.33E-09 | 1.84E-08 |
| IFFO2 | -0.2580948 | 2.33E-09 | 1.84E-08 |
| C16orf79 | 0.2580852 | 2.34E-09 | 1.84E-08 |
| TYRP1 | -0.2579324 | 2.39E-09 | 1.88E-08 |
| ZNF676 | -0.2579192 | 2.39E-09 | 1.88E-08 |
| TIPRL | 0.2578649 | 2.41E-09 | 1.90E-08 |
| BAZ2B | -0.2578054 | 2.43E-09 | 1.91E-08 |
| CD320 | 0.2577744 | 2.44E-09 | 1.92E-08 |
| S1PR1 | -0.2577706 | 2.45E-09 | 1.92E-08 |
| SLITRK6 | -0.2577469 | 2.45E-09 | 1.93E-08 |
| ANAPC11 | 0.2576998 | 2.47E-09 | 1.94E-08 |
| MRPL30 | 0.2576684 | 2.48E-09 | 1.95E-08 |
| MITF | -0.2576337 | 2.50E-09 | 1.96E-08 |
| HDDC3 | 0.2575827 | 2.51E-09 | 1.97E-08 |
| GPR133 | -0.2575048 | 2.54E-09 | 1.99E-08 |
| GSDMA | -0.2574936 | 2.55E-09 | 2.00E-08 |
| RSPRY1 | -0.2574549 | 2.56E-09 | 2.01E-08 |
| THUMPD3 | 0.2574547 | 2.56E-09 | 2.01E-08 |
| UTP14A | 0.2574387 | 2.57E-09 | 2.01E-08 |
| POLE4 | 0.257408 | 2.58E-09 | 2.02E-08 |
| CHRND | -0.2573843 | 2.59E-09 | 2.03E-08 |
| C7orf58 | -0.2573593 | 2.60E-09 | 2.03E-08 |
| RPS15A | 0.2573555 | 2.60E-09 | 2.03E-08 |
| CCDC3 | -0.2573531 | 2.60E-09 | 2.03E-08 |
| PRTG | -0.2573035 | 2.62E-09 | 2.05E-08 |
| KCNB1 | -0.2572875 | 2.63E-09 | 2.05E-08 |
| MRPL55 | 0.2571553 | 2.68E-09 | 2.09E-08 |
| MARK4 | -0.2571532 | 2.68E-09 | 2.09E-08 |
| C6orf41 | 0.2570899 | 2.70E-09 | 2.11E-08 |
| LIPM | -0.257057 | 2.72E-09 | 2.12E-08 |
| GRIA1 | -0.2570526 | 2.72E-09 | 2.12E-08 |
| SPTAN1 | -0.2570408 | 2.72E-09 | 2.12E-08 |
| VPS11 | -0.2570124 | 2.73E-09 | 2.13E-08 |
| R3HCC1 | 0.256895 | 2.78E-09 | 2.16E-08 |
| MCRS1 | 0.2568812 | 2.79E-09 | 2.17E-08 |
| AHCY | 0.2568495 | 2.80E-09 | 2.18E-08 |
| CHST1 | -0.2568209 | 2.81E-09 | 2.19E-08 |
| NOC4L | 0.2568087 | 2.82E-09 | 2.19E-08 |
| FAM171B | -0.2567813 | 2.83E-09 | 2.20E-08 |
| PRPH2 | -0.2567494 | 2.84E-09 | 2.21E-08 |
| DCTN6 | 0.2567421 | 2.84E-09 | 2.21E-08 |
| MTHFD2L | 0.2567197 | 2.85E-09 | 2.21E-08 |
| TMEM204 | -0.2566368 | 2.89E-09 | 2.24E-08 |
| MCC | -0.2566163 | 2.90E-09 | 2.25E-08 |
| FICD | -0.2566109 | 2.90E-09 | 2.25E-08 |
| ARHGAP42 | -0.2566041 | 2.90E-09 | 2.25E-08 |
| SCN4A | -0.2565065 | 2.94E-09 | 2.28E-08 |
| C14orf179 | 0.2564883 | 2.95E-09 | 2.28E-08 |
| PTRH2 | 0.2564768 | 2.96E-09 | 2.29E-08 |
| GPR123 | -0.256408 | 2.99E-09 | 2.31E-08 |
| USP9X | -0.2563888 | 2.99E-09 | 2.32E-08 |
| MS4A2 | -0.2563067 | 3.03E-09 | 2.34E-08 |
| FKBP7 | -0.2562969 | 3.03E-09 | 2.35E-08 |
| C12orf43 | 0.25624 | 3.06E-09 | 2.36E-08 |
| REST | -0.2560297 | 3.15E-09 | 2.44E-08 |
| NKX3-2 | -0.2560227 | 3.16E-09 | 2.44E-08 |
| FAM165B | 0.2559869 | 3.17E-09 | 2.45E-08 |
| MRPL21 | 0.2559768 | 3.18E-09 | 2.45E-08 |
| TMEM2 | -0.2559747 | 3.18E-09 | 2.45E-08 |
| SMN2 | 0.25596 | 3.19E-09 | 2.46E-08 |
| NOLC1 | 0.2559518 | 3.19E-09 | 2.46E-08 |
| FAM98C | 0.2559338 | 3.20E-09 | 2.46E-08 |
| ACER1 | -0.2559328 | 3.20E-09 | 2.46E-08 |
| LPP | -0.2558692 | 3.23E-09 | 2.49E-08 |
| MGRN1 | -0.2558644 | 3.23E-09 | 2.49E-08 |
| ZFP92 | -0.2557443 | 3.29E-09 | 2.53E-08 |
| C10orf71 | -0.2557272 | 3.30E-09 | 2.53E-08 |
| DGCR2 | -0.2557195 | 3.30E-09 | 2.54E-08 |
| TREX1 | 0.2557162 | 3.30E-09 | 2.54E-08 |
| SUZ12P | 0.2557119 | 3.30E-09 | 2.54E-08 |
| PRKCA | -0.2556684 | 3.33E-09 | 2.55E-08 |
| TPST2 | -0.2556545 | 3.33E-09 | 2.56E-08 |
| ISCU | 0.2555907 | 3.36E-09 | 2.58E-08 |
| ZFAND6 | 0.2555765 | 3.37E-09 | 2.58E-08 |
| SUB1 | 0.2555114 | 3.40E-09 | 2.61E-08 |
| NDUFS6 | 0.2554997 | 3.41E-09 | 2.61E-08 |
| MYCBP2 | -0.2554819 | 3.42E-09 | 2.62E-08 |
| CCDC125 | 0.2554691 | 3.42E-09 | 2.62E-08 |
| IK | 0.2554393 | 3.44E-09 | 2.63E-08 |
| GLMN | 0.2554042 | 3.46E-09 | 2.64E-08 |
| RNF34 | 0.2553939 | 3.46E-09 | 2.65E-08 |
| ARAP2 | -0.255373 | 3.47E-09 | 2.65E-08 |
| HIST1H3E | -0.2553104 | 3.50E-09 | 2.68E-08 |
| INTS10 | 0.2552847 | 3.52E-09 | 2.69E-08 |
| OSBP | -0.2552547 | 3.53E-09 | 2.70E-08 |
| RG9MTD1 | 0.2552004 | 3.56E-09 | 2.72E-08 |
| SYPL2 | -0.2551677 | 3.58E-09 | 2.73E-08 |
| BRCC3 | 0.2551487 | 3.59E-09 | 2.73E-08 |
| ZNF319 | -0.2551471 | 3.59E-09 | 2.73E-08 |
| C1orf96 | 0.2550256 | 3.65E-09 | 2.78E-08 |
| METTL6 | 0.2550063 | 3.66E-09 | 2.79E-08 |
| RAI2 | -0.2550037 | 3.66E-09 | 2.79E-08 |
| RPS15 | 0.2549618 | 3.68E-09 | 2.80E-08 |
| NLRP9 | 0.2549271 | 3.70E-09 | 2.82E-08 |
| PRRG1 | -0.2549244 | 3.70E-09 | 2.82E-08 |
| RARA | -0.2548954 | 3.72E-09 | 2.83E-08 |
| DUSP27 | -0.2548387 | 3.75E-09 | 2.85E-08 |
| ACSL1 | -0.2548306 | 3.76E-09 | 2.85E-08 |
| NDUFS7 | 0.2548269 | 3.76E-09 | 2.85E-08 |
| RNF5 | 0.2548142 | 3.76E-09 | 2.86E-08 |
| PRDX2 | 0.2548052 | 3.77E-09 | 2.86E-08 |
| FAM108C1 | 0.2547988 | 3.77E-09 | 2.86E-08 |
| TTN | -0.2547897 | 3.78E-09 | 2.86E-08 |
| ARL15 | -0.2546883 | 3.83E-09 | 2.91E-08 |
| SAMD5 | -0.2546687 | 3.84E-09 | 2.91E-08 |
| ACTA2 | -0.2546121 | 3.88E-09 | 2.94E-08 |
| RHBDD1 | -0.2546033 | 3.88E-09 | 2.94E-08 |
| LPHN2 | -0.2545945 | 3.89E-09 | 2.94E-08 |
| PTPN2 | 0.2545768 | 3.90E-09 | 2.95E-08 |
| DMKN | -0.2545472 | 3.91E-09 | 2.96E-08 |
| ELAVL1 | 0.2544865 | 3.95E-09 | 2.98E-08 |
| PAPLN | -0.254476 | 3.95E-09 | 2.99E-08 |
| NPY6R | -0.2544755 | 3.95E-09 | 2.99E-08 |
| HSPB7 | -0.2544627 | 3.96E-09 | 2.99E-08 |
| KDR | -0.2544459 | 3.97E-09 | 3.00E-08 |
| PCDHGB1 | -0.2544443 | 3.97E-09 | 3.00E-08 |
| BAX | 0.2544381 | 3.97E-09 | 3.00E-08 |
| UNC45B | -0.2544312 | 3.98E-09 | 3.00E-08 |
| MRPS12 | 0.2544304 | 3.98E-09 | 3.00E-08 |
| LOC150776 | 0.2543965 | 4.00E-09 | 3.01E-08 |
| ZNF70 | -0.2543488 | 4.03E-09 | 3.03E-08 |
| WARS2 | 0.2542716 | 4.07E-09 | 3.07E-08 |
| GGN | -0.2541909 | 4.12E-09 | 3.10E-08 |
| RFXANK | 0.254128 | 4.16E-09 | 3.13E-08 |
| FAM69A | -0.2540965 | 4.18E-09 | 3.14E-08 |
| CYP4F22 | -0.2540939 | 4.18E-09 | 3.14E-08 |
| PCDHGB3 | -0.2540433 | 4.21E-09 | 3.16E-08 |
| FLII | -0.2539933 | 4.24E-09 | 3.18E-08 |
| RGP1 | -0.2539784 | 4.25E-09 | 3.19E-08 |
| ST3GAL1 | -0.253967 | 4.25E-09 | 3.19E-08 |
| DPH5 | 0.2538843 | 4.31E-09 | 3.23E-08 |
| LOC728554 | 0.2538685 | 4.32E-09 | 3.24E-08 |
| NUP93 | 0.2538592 | 4.32E-09 | 3.24E-08 |
| IQSEC3 | -0.2538174 | 4.35E-09 | 3.26E-08 |
| SNPH | -0.2538093 | 4.35E-09 | 3.26E-08 |
| MICALCL | -0.2537679 | 4.38E-09 | 3.28E-08 |
| ASH1L | -0.253765 | 4.38E-09 | 3.28E-08 |
| TIGD3 | 0.253741 | 4.40E-09 | 3.29E-08 |
| MKS1 | 0.2537331 | 4.40E-09 | 3.29E-08 |
| WRNIP1 | 0.2537262 | 4.40E-09 | 3.29E-08 |
| C2orf84 | -0.2536962 | 4.42E-09 | 3.31E-08 |
| CDSN | -0.2536542 | 4.45E-09 | 3.33E-08 |
| WDR81 | -0.2536371 | 4.46E-09 | 3.33E-08 |
| ERI2 | 0.2535894 | 4.49E-09 | 3.36E-08 |
| TNFRSF1A | -0.253569 | 4.51E-09 | 3.36E-08 |
| MYO9B | -0.2535566 | 4.51E-09 | 3.37E-08 |
| MRPS33 | 0.2535278 | 4.53E-09 | 3.38E-08 |
| PIGU | 0.2535211 | 4.54E-09 | 3.38E-08 |
| PEA15 | -0.2534312 | 4.60E-09 | 3.43E-08 |
| SBSN | -0.2534024 | 4.62E-09 | 3.44E-08 |
| PRRC1 | -0.2533042 | 4.68E-09 | 3.49E-08 |
| EIF4EBP1 | 0.2532951 | 4.69E-09 | 3.49E-08 |
| SDK2 | -0.2531814 | 4.76E-09 | 3.55E-08 |
| NCAPD2 | 0.2531758 | 4.77E-09 | 3.55E-08 |
| KRTDAP | -0.2531605 | 4.78E-09 | 3.55E-08 |
| ETV3 | -0.2531103 | 4.81E-09 | 3.58E-08 |
| ATP5B | 0.2530513 | 4.85E-09 | 3.61E-08 |
| PPM1J | 0.2529927 | 4.90E-09 | 3.64E-08 |
| PGM5 | -0.2529759 | 4.91E-09 | 3.64E-08 |
| FAM48B1 | -0.2529744 | 4.91E-09 | 3.64E-08 |
| DPT | -0.2529591 | 4.92E-09 | 3.65E-08 |
| ENOSF1 | 0.252957 | 4.92E-09 | 3.65E-08 |
| F2RL1 | -0.2529464 | 4.93E-09 | 3.65E-08 |
| C2CD2L | -0.2529298 | 4.94E-09 | 3.66E-08 |
| MYT1L | -0.2529092 | 4.95E-09 | 3.67E-08 |
| PSAP | -0.2529087 | 4.96E-09 | 3.67E-08 |
| GRK6 | 0.252907 | 4.96E-09 | 3.67E-08 |
| IGFL2 | -0.252884 | 4.97E-09 | 3.68E-08 |
| C2orf64 | 0.2528798 | 4.98E-09 | 3.68E-08 |
| MYH4 | -0.2528666 | 4.99E-09 | 3.69E-08 |
| NONO | 0.252819 | 5.02E-09 | 3.71E-08 |
| XCR1 | -0.252805 | 5.03E-09 | 3.72E-08 |
| PGM2L1 | -0.2527956 | 5.04E-09 | 3.72E-08 |
| KRT10 | -0.2527812 | 5.05E-09 | 3.73E-08 |
| NDUFA11 | 0.2527132 | 5.10E-09 | 3.76E-08 |
| CD163L1 | -0.2526823 | 5.12E-09 | 3.78E-08 |
| SCARNA12 | 0.2526146 | 5.17E-09 | 3.81E-08 |
| TMEM203 | 0.2525904 | 5.19E-09 | 3.83E-08 |
| AR | -0.2525298 | 5.23E-09 | 3.86E-08 |
| RCL1 | 0.2525056 | 5.25E-09 | 3.87E-08 |
| RASGRP4 | -0.2524988 | 5.26E-09 | 3.87E-08 |
| KRR1 | 0.2524785 | 5.27E-09 | 3.88E-08 |
| MCM8 | 0.2522952 | 5.41E-09 | 3.98E-08 |
| SELK | 0.2522901 | 5.42E-09 | 3.99E-08 |
| RAPGEF4 | -0.2522604 | 5.44E-09 | 4.00E-08 |
| SMOC2 | -0.2522543 | 5.44E-09 | 4.00E-08 |
| GIN1 | 0.2522022 | 5.48E-09 | 4.03E-08 |
| ATG3 | 0.2521834 | 5.50E-09 | 4.04E-08 |
| JHDM1D | -0.2521472 | 5.53E-09 | 4.06E-08 |
| MYF5 | -0.2520782 | 5.58E-09 | 4.10E-08 |
| WDR77 | 0.2520084 | 5.64E-09 | 4.14E-08 |
| CCDC111 | 0.2519575 | 5.68E-09 | 4.17E-08 |
| C5orf4 | -0.2519539 | 5.68E-09 | 4.17E-08 |
| NCK2 | -0.2519067 | 5.72E-09 | 4.19E-08 |
| C14orf138 | 0.2518941 | 5.73E-09 | 4.20E-08 |
| PLEKHG1 | -0.2518279 | 5.79E-09 | 4.24E-08 |
| TMEM150A | -0.2518005 | 5.81E-09 | 4.25E-08 |
| UBR7 | 0.2517875 | 5.82E-09 | 4.26E-08 |
| SYT11 | -0.2517514 | 5.85E-09 | 4.28E-08 |
| EDARADD | 0.2517442 | 5.86E-09 | 4.28E-08 |
| RXFP3 | -0.2517365 | 5.86E-09 | 4.29E-08 |
| EXOSC10 | 0.2517203 | 5.88E-09 | 4.30E-08 |
| CACNA2D1 | -0.2517144 | 5.88E-09 | 4.30E-08 |
| DENND1B | -0.2516871 | 5.90E-09 | 4.31E-08 |
| NT5C | 0.2516598 | 5.93E-09 | 4.33E-08 |
| C6orf162 | 0.2516413 | 5.94E-09 | 4.34E-08 |
| FGFR1 | -0.2516264 | 5.95E-09 | 4.35E-08 |
| SCAI | 0.2515898 | 5.99E-09 | 4.37E-08 |
| NDUFV2 | 0.251568 | 6.00E-09 | 4.38E-08 |
| WNT7B | -0.2515589 | 6.01E-09 | 4.38E-08 |
| THAP1 | 0.2515201 | 6.05E-09 | 4.41E-08 |
| FBXO30 | -0.2515089 | 6.06E-09 | 4.41E-08 |
| RSRC2 | 0.2514699 | 6.09E-09 | 4.43E-08 |
| TEX2 | -0.2514582 | 6.10E-09 | 4.44E-08 |
| METTL4 | 0.2514167 | 6.14E-09 | 4.46E-08 |
| NSUN5 | 0.2513862 | 6.16E-09 | 4.48E-08 |
| ADAMTSL2 | -0.2513834 | 6.17E-09 | 4.48E-08 |
| KAT2A | 0.2513759 | 6.17E-09 | 4.49E-08 |
| CMYA5 | -0.2513691 | 6.18E-09 | 4.49E-08 |
| ZNF474 | -0.2513658 | 6.18E-09 | 4.49E-08 |
| C11orf63 | -0.2513071 | 6.23E-09 | 4.53E-08 |
| EXTL1 | -0.2513027 | 6.24E-09 | 4.53E-08 |
| APCDD1 | -0.2511995 | 6.33E-09 | 4.59E-08 |
| MMACHC | 0.2511798 | 6.35E-09 | 4.60E-08 |
| ZNF541 | 0.2510773 | 6.44E-09 | 4.67E-08 |
| MEF2A | -0.2510293 | 6.48E-09 | 4.70E-08 |
| ROBO4 | -0.2510105 | 6.50E-09 | 4.71E-08 |
| CACNG6 | -0.2509576 | 6.55E-09 | 4.74E-08 |
| TRAF4 | 0.2509528 | 6.56E-09 | 4.75E-08 |
| MRPL4 | 0.2509029 | 6.60E-09 | 4.78E-08 |
| VGLL2 | -0.2508013 | 6.70E-09 | 4.85E-08 |
| LHFP | -0.2507975 | 6.70E-09 | 4.85E-08 |
| C1GALT1C1 | 0.250734 | 6.76E-09 | 4.89E-08 |
| GIGYF2 | -0.2507306 | 6.77E-09 | 4.89E-08 |
| NAV3 | -0.2507162 | 6.78E-09 | 4.90E-08 |
| ADCY2 | -0.2506588 | 6.84E-09 | 4.94E-08 |
| SLC25A38 | 0.2505797 | 6.91E-09 | 4.99E-08 |
| LMBRD2 | -0.2505764 | 6.92E-09 | 4.99E-08 |
| GPNMB | -0.2505715 | 6.92E-09 | 4.99E-08 |
| DCUN1D3 | -0.2504992 | 6.99E-09 | 5.04E-08 |
| LRP1B | -0.2504731 | 7.02E-09 | 5.06E-08 |
| MFAP3L | -0.2504679 | 7.02E-09 | 5.06E-08 |
| TMEM39B | 0.2504675 | 7.02E-09 | 5.06E-08 |
| MBD5 | -0.2504644 | 7.03E-09 | 5.06E-08 |
| MYH8 | -0.2504511 | 7.04E-09 | 5.07E-08 |
| PJA2 | -0.250414 | 7.08E-09 | 5.09E-08 |
| SIGMAR1 | 0.250397 | 7.10E-09 | 5.10E-08 |
| ISCA2 | 0.2503957 | 7.10E-09 | 5.10E-08 |
| MPDZ | -0.2503453 | 7.15E-09 | 5.14E-08 |
| PSMA7 | 0.250345 | 7.15E-09 | 5.14E-08 |
| CDHR1 | -0.2503183 | 7.17E-09 | 5.15E-08 |
| ADK | 0.2502092 | 7.29E-09 | 5.23E-08 |
| OSBPL1A | -0.2501995 | 7.30E-09 | 5.24E-08 |
| CAMK2D | -0.2501912 | 7.31E-09 | 5.24E-08 |
| COPS2 | 0.2501355 | 7.36E-09 | 5.28E-08 |
| NHP2L1 | 0.2501216 | 7.38E-09 | 5.29E-08 |
| UBE2CBP | 0.2501158 | 7.38E-09 | 5.29E-08 |
| RCN2 | 0.250098 | 7.40E-09 | 5.30E-08 |
| PSME1 | 0.250057 | 7.45E-09 | 5.33E-08 |
| LRRC14B | -0.2500386 | 7.47E-09 | 5.35E-08 |
| RAB43 | -0.2500035 | 7.50E-09 | 5.37E-08 |
| ZNF677 | -0.2500015 | 7.51E-09 | 5.37E-08 |
| LBR | 0.2499942 | 7.51E-09 | 5.37E-08 |
| C1orf95 | -0.2499922 | 7.52E-09 | 5.37E-08 |
| COMMD3 | 0.2499294 | 7.58E-09 | 5.42E-08 |
| COL25A1 | -0.2499206 | 7.59E-09 | 5.42E-08 |
| AMPD2 | -0.2499087 | 7.60E-09 | 5.43E-08 |
| PSMD4 | 0.2498892 | 7.63E-09 | 5.44E-08 |
| TIMM13 | 0.249846 | 7.67E-09 | 5.48E-08 |
| HMGA1 | 0.2498411 | 7.68E-09 | 5.48E-08 |
| EIF3K | 0.2498145 | 7.71E-09 | 5.50E-08 |
| ZC3H13 | -0.2497956 | 7.73E-09 | 5.51E-08 |
| NCRNA00188 | 0.2497866 | 7.74E-09 | 5.51E-08 |
| SLC25A17 | 0.2497776 | 7.75E-09 | 5.52E-08 |
| PARP1 | 0.2497549 | 7.77E-09 | 5.54E-08 |
| MFAP5 | -0.24973 | 7.80E-09 | 5.55E-08 |
| CCDC155 | 0.2496367 | 7.90E-09 | 5.63E-08 |
| GPR4 | -0.249594 | 7.95E-09 | 5.66E-08 |
| PI15 | -0.2495793 | 7.97E-09 | 5.67E-08 |
| UBE2M | 0.2495743 | 7.97E-09 | 5.67E-08 |
| LCE3E | -0.2495239 | 8.03E-09 | 5.71E-08 |
| TMEM214 | -0.2494939 | 8.07E-09 | 5.73E-08 |
| MGP | -0.2494211 | 8.15E-09 | 5.79E-08 |
| LARP7 | 0.2493989 | 8.17E-09 | 5.80E-08 |
| NUAK1 | -0.2493886 | 8.19E-09 | 5.81E-08 |
| TSPAN7 | -0.2493342 | 8.25E-09 | 5.85E-08 |
| CISD3 | 0.2493034 | 8.29E-09 | 5.88E-08 |
| SDCCAG3 | 0.2493019 | 8.29E-09 | 5.88E-08 |
| TMEM14C | 0.2492929 | 8.30E-09 | 5.88E-08 |
| TSPAN18 | -0.2492702 | 8.32E-09 | 5.90E-08 |
| EBF1 | -0.2492143 | 8.39E-09 | 5.94E-08 |
| EFHD2 | 0.2491752 | 8.44E-09 | 5.97E-08 |
| DDX50 | 0.2491628 | 8.45E-09 | 5.98E-08 |
| IL12A | 0.2491372 | 8.48E-09 | 6.00E-08 |
| BCL7C | 0.249135 | 8.49E-09 | 6.00E-08 |
| TMEM160 | 0.2491311 | 8.49E-09 | 6.00E-08 |
| SLC23A2 | -0.2491049 | 8.52E-09 | 6.02E-08 |
| TMEM8C | -0.2490564 | 8.58E-09 | 6.06E-08 |
| TMEM171 | 0.2490469 | 8.59E-09 | 6.07E-08 |
| LOC647979 | -0.2490132 | 8.63E-09 | 6.09E-08 |
| CDYL2 | -0.2489791 | 8.67E-09 | 6.12E-08 |
| RPIA | 0.2488772 | 8.80E-09 | 6.21E-08 |
| RNF11 | -0.2488616 | 8.82E-09 | 6.22E-08 |
| CSRNP3 | -0.2488336 | 8.85E-09 | 6.24E-08 |
| MMP27 | -0.2488064 | 8.89E-09 | 6.26E-08 |
| IQSEC2 | -0.2487468 | 8.96E-09 | 6.32E-08 |
| CTSG | -0.2487402 | 8.97E-09 | 6.32E-08 |
| NOL12 | 0.2487326 | 8.98E-09 | 6.32E-08 |
| ARHGEF7 | -0.2487252 | 8.99E-09 | 6.33E-08 |
| SAMD14 | -0.2486989 | 9.02E-09 | 6.35E-08 |
| RCVRN | -0.2486953 | 9.03E-09 | 6.35E-08 |
| HIST1H2AB | 0.2486944 | 9.03E-09 | 6.35E-08 |
| FGF18 | -0.2486808 | 9.05E-09 | 6.36E-08 |
| SLC5A1 | -0.2486306 | 9.11E-09 | 6.40E-08 |
| MRRF | 0.2486138 | 9.13E-09 | 6.41E-08 |
| FOXD4 | 0.2485429 | 9.23E-09 | 6.48E-08 |
| PHACTR1 | -0.248515 | 9.26E-09 | 6.50E-08 |
| SNORD116-4 | -0.248474 | 9.32E-09 | 6.54E-08 |
| IVL | -0.2484497 | 9.35E-09 | 6.56E-08 |
| KIAA1217 | -0.2484367 | 9.36E-09 | 6.57E-08 |
| RPTN | -0.2483632 | 9.46E-09 | 6.63E-08 |
| PLXND1 | -0.2482785 | 9.58E-09 | 6.71E-08 |
| PSMA1 | 0.2482282 | 9.64E-09 | 6.75E-08 |
| TSPAN5 | -0.2482086 | 9.67E-09 | 6.77E-08 |
| JPH2 | -0.2481818 | 9.71E-09 | 6.79E-08 |
| GXYLT2 | -0.2481816 | 9.71E-09 | 6.79E-08 |
| OLA1 | 0.2481495 | 9.75E-09 | 6.82E-08 |
| MTL5 | 0.2481216 | 9.79E-09 | 6.84E-08 |
| SLC29A4 | -0.2481108 | 9.80E-09 | 6.85E-08 |
| ITGA5 | -0.2481008 | 9.82E-09 | 6.86E-08 |
| MYH13 | -0.2480638 | 9.87E-09 | 6.89E-08 |
| ZYG11B | -0.2479303 | 1.01E-08 | 7.02E-08 |
| KRT18 | 0.2479001 | 1.01E-08 | 7.05E-08 |
| MTMR2 | -0.2478957 | 1.01E-08 | 7.05E-08 |
| POLR1C | 0.2478544 | 1.02E-08 | 7.09E-08 |
| TCEAL7 | -0.2478117 | 1.02E-08 | 7.13E-08 |
| CRYBG3 | -0.2477863 | 1.03E-08 | 7.15E-08 |
| B9D2 | 0.2477731 | 1.03E-08 | 7.16E-08 |
| THBS1 | -0.2477399 | 1.03E-08 | 7.19E-08 |
| OGDH | -0.2477145 | 1.04E-08 | 7.22E-08 |
| PHLDB2 | -0.247627 | 1.05E-08 | 7.30E-08 |
| HMGN3 | 0.2475965 | 1.05E-08 | 7.33E-08 |
| FGF5 | -0.24755 | 1.06E-08 | 7.38E-08 |
| COPA | -0.247539 | 1.06E-08 | 7.39E-08 |
| FLG | -0.2474585 | 1.07E-08 | 7.47E-08 |
| TEP1 | -0.2474312 | 1.08E-08 | 7.49E-08 |
| FKBP14 | -0.2474305 | 1.08E-08 | 7.49E-08 |
| CCDC23 | 0.2474049 | 1.08E-08 | 7.52E-08 |
| SNORD116-20 | -0.2473693 | 1.09E-08 | 7.55E-08 |
| PCDH1 | -0.2473536 | 1.09E-08 | 7.57E-08 |
| SAMD4A | -0.2473441 | 1.09E-08 | 7.57E-08 |
| SYDE1 | -0.2473271 | 1.09E-08 | 7.59E-08 |
| CLIP1 | -0.2473079 | 1.10E-08 | 7.61E-08 |
| HIST1H3G | 0.2473037 | 1.10E-08 | 7.61E-08 |
| KIAA1109 | -0.2472958 | 1.10E-08 | 7.62E-08 |
| FAM195A | 0.2472342 | 1.11E-08 | 7.68E-08 |
| ADPRH | -0.2472147 | 1.11E-08 | 7.70E-08 |
| MYO9A | -0.2471864 | 1.12E-08 | 7.73E-08 |
| CPXM2 | -0.2471232 | 1.13E-08 | 7.79E-08 |
| TAF12 | 0.2470883 | 1.13E-08 | 7.83E-08 |
| TAF7L | 0.2470742 | 1.13E-08 | 7.84E-08 |
| KDM5A | -0.2470508 | 1.14E-08 | 7.86E-08 |
| CALCR | -0.247028 | 1.14E-08 | 7.88E-08 |
| PCDHGB2 | -0.247025 | 1.14E-08 | 7.89E-08 |
| PRKAG3 | -0.2470006 | 1.15E-08 | 7.91E-08 |
| URM1 | 0.2469777 | 1.15E-08 | 7.93E-08 |
| TMEM14A | 0.2469736 | 1.15E-08 | 7.93E-08 |
| STAG3 | 0.2469694 | 1.15E-08 | 7.94E-08 |
| APEX2 | 0.2469242 | 1.16E-08 | 7.98E-08 |
| DYNC2H1 | -0.2468742 | 1.17E-08 | 8.04E-08 |
| LMTK2 | -0.2468408 | 1.17E-08 | 8.07E-08 |
| KIF3C | -0.2468117 | 1.18E-08 | 8.10E-08 |
| TIFA | 0.2468009 | 1.18E-08 | 8.11E-08 |
| RAPSN | -0.2467855 | 1.18E-08 | 8.13E-08 |
| ATPAF1 | 0.2467729 | 1.18E-08 | 8.14E-08 |
| CRYGN | -0.2467388 | 1.19E-08 | 8.17E-08 |
| LOC284441 | -0.2467241 | 1.19E-08 | 8.19E-08 |
| BAZ2A | -0.2466744 | 1.20E-08 | 8.24E-08 |
| PRDM2 | -0.2466678 | 1.20E-08 | 8.25E-08 |
| SRA1 | 0.2466463 | 1.20E-08 | 8.27E-08 |
| ACY3 | 0.2466184 | 1.21E-08 | 8.30E-08 |
| CNTN3 | -0.2465865 | 1.21E-08 | 8.33E-08 |
| CR1L | 0.2465416 | 1.22E-08 | 8.38E-08 |
| SYNPO | -0.2464852 | 1.23E-08 | 8.45E-08 |
| ESAM | -0.2464117 | 1.24E-08 | 8.53E-08 |
| ZNF296 | 0.2463924 | 1.25E-08 | 8.55E-08 |
| H2AFY | 0.2463899 | 1.25E-08 | 8.55E-08 |
| DCLRE1A | 0.2463639 | 1.25E-08 | 8.58E-08 |
| CHCHD3 | 0.2462934 | 1.26E-08 | 8.66E-08 |
| KIAA0494 | -0.2462655 | 1.27E-08 | 8.69E-08 |
| LPCAT4 | 0.246262 | 1.27E-08 | 8.69E-08 |
| SPEG | -0.2462159 | 1.28E-08 | 8.75E-08 |
| MRPS31 | 0.2461927 | 1.28E-08 | 8.77E-08 |
| CHRAC1 | 0.2461846 | 1.28E-08 | 8.78E-08 |
| ARNT | -0.2461796 | 1.29E-08 | 8.78E-08 |
| FOXO3 | -0.2461738 | 1.29E-08 | 8.79E-08 |
| CDK2AP2 | 0.2461208 | 1.30E-08 | 8.85E-08 |
| DTWD1 | 0.2460821 | 1.30E-08 | 8.89E-08 |
| AP1G1 | -0.2460361 | 1.31E-08 | 8.95E-08 |
| ST3GAL2 | -0.2460276 | 1.31E-08 | 8.95E-08 |
| ITFG1 | -0.2460048 | 1.32E-08 | 8.98E-08 |
| RPS20 | 0.245936 | 1.33E-08 | 9.06E-08 |
| TTC3 | -0.245904 | 1.34E-08 | 9.10E-08 |
| MED9 | 0.2458879 | 1.34E-08 | 9.12E-08 |
| AZI1 | 0.2458757 | 1.34E-08 | 9.13E-08 |
| ART1 | -0.2458672 | 1.34E-08 | 9.14E-08 |
| TMEM132C | -0.2458455 | 1.35E-08 | 9.16E-08 |
| NDUFA5 | 0.2458342 | 1.35E-08 | 9.17E-08 |
| PCDH12 | -0.2457731 | 1.36E-08 | 9.25E-08 |
| TAL1 | -0.2457657 | 1.36E-08 | 9.26E-08 |
| CCDC14 | 0.245733 | 1.37E-08 | 9.30E-08 |
| NOMO1 | -0.2457184 | 1.37E-08 | 9.31E-08 |
| LPPR3 | -0.2456941 | 1.38E-08 | 9.34E-08 |
| MORN2 | 0.2456881 | 1.38E-08 | 9.34E-08 |
| PDPK1 | -0.2456482 | 1.38E-08 | 9.39E-08 |
| FLT4 | -0.24561 | 1.39E-08 | 9.44E-08 |
| DDX51 | 0.245609 | 1.39E-08 | 9.44E-08 |
| PCDHB12 | -0.24559 | 1.40E-08 | 9.46E-08 |
| C11orf31 | 0.2455201 | 1.41E-08 | 9.55E-08 |
| C3orf36 | -0.245508 | 1.41E-08 | 9.56E-08 |
| LRRFIP1 | -0.2454997 | 1.41E-08 | 9.57E-08 |
| MRPL34 | 0.2454838 | 1.42E-08 | 9.59E-08 |
| VPS25 | 0.2454531 | 1.42E-08 | 9.63E-08 |
| LETMD1 | 0.2454358 | 1.43E-08 | 9.65E-08 |
| CHST15 | -0.2454007 | 1.43E-08 | 9.69E-08 |
| LOC388796 | 0.2453844 | 1.44E-08 | 9.71E-08 |
| MAMDC2 | -0.2453508 | 1.44E-08 | 9.75E-08 |
| KCNA1 | -0.2453417 | 1.44E-08 | 9.76E-08 |
| SHISA6 | -0.2453245 | 1.45E-08 | 9.78E-08 |
| HDX | -0.2452622 | 1.46E-08 | 9.86E-08 |
| C11orf83 | 0.2452197 | 1.47E-08 | 9.92E-08 |
| SFI1 | 0.2451252 | 1.49E-08 | 1.00E-07 |
| CCDC12 | 0.2451056 | 1.49E-08 | 1.01E-07 |
| TOM1L2 | -0.2450906 | 1.50E-08 | 1.01E-07 |
| TRIM55 | -0.2450679 | 1.50E-08 | 1.01E-07 |
| GPR162 | -0.2450613 | 1.50E-08 | 1.01E-07 |
| DIRC1 | -0.2450305 | 1.51E-08 | 1.02E-07 |
| KIAA1712 | 0.2450037 | 1.51E-08 | 1.02E-07 |
| DAP3 | 0.2448387 | 1.55E-08 | 1.04E-07 |
| MRPL44 | 0.2448338 | 1.55E-08 | 1.04E-07 |
| FMR1 | 0.2448232 | 1.55E-08 | 1.04E-07 |
| KIAA1919 | -0.2448015 | 1.56E-08 | 1.05E-07 |
| PTGFR | -0.2447693 | 1.56E-08 | 1.05E-07 |
| BAI1 | -0.2447499 | 1.57E-08 | 1.05E-07 |
| MRPS22 | 0.2447339 | 1.57E-08 | 1.06E-07 |
| PCDHB2 | -0.2446826 | 1.58E-08 | 1.06E-07 |
| WWP2 | -0.2446687 | 1.59E-08 | 1.06E-07 |
| SLC16A2 | -0.2446664 | 1.59E-08 | 1.06E-07 |
| CNTN4 | -0.2446276 | 1.59E-08 | 1.07E-07 |
| FNDC3A | -0.2445987 | 1.60E-08 | 1.07E-07 |
| PRDX3 | 0.2445916 | 1.60E-08 | 1.07E-07 |
| ZFHX3 | -0.2445827 | 1.60E-08 | 1.08E-07 |
| FZD4 | -0.2445761 | 1.61E-08 | 1.08E-07 |
| PRICKLE2 | -0.2445258 | 1.62E-08 | 1.08E-07 |
| FGFR1OP | 0.2445044 | 1.62E-08 | 1.09E-07 |
| FNDC4 | -0.2444782 | 1.63E-08 | 1.09E-07 |
| SDHC | 0.2444645 | 1.63E-08 | 1.09E-07 |
| RAI14 | -0.2444644 | 1.63E-08 | 1.09E-07 |
| CECR5 | 0.2444562 | 1.63E-08 | 1.09E-07 |
| CHL1 | -0.2444217 | 1.64E-08 | 1.10E-07 |
| TANC2 | -0.2443747 | 1.65E-08 | 1.10E-07 |
| C6orf15 | -0.2443621 | 1.65E-08 | 1.11E-07 |
| PURG | -0.2443431 | 1.66E-08 | 1.11E-07 |
| FGF16 | -0.2443358 | 1.66E-08 | 1.11E-07 |
| FAM133B | 0.2443259 | 1.66E-08 | 1.11E-07 |
| NDUFA4 | 0.2443113 | 1.67E-08 | 1.11E-07 |
| C1orf70 | -0.2442604 | 1.68E-08 | 1.12E-07 |
| PRDM1 | -0.2442416 | 1.68E-08 | 1.12E-07 |
| ITPRIPL2 | -0.2442287 | 1.69E-08 | 1.12E-07 |
| SOCS7 | -0.2442195 | 1.69E-08 | 1.12E-07 |
| MTERFD3 | 0.2441713 | 1.70E-08 | 1.13E-07 |
| MCAM | -0.2441672 | 1.70E-08 | 1.13E-07 |
| PSMD13 | 0.2441591 | 1.70E-08 | 1.13E-07 |
| CBL | -0.2440971 | 1.72E-08 | 1.14E-07 |
| ORMDL1 | 0.2440797 | 1.72E-08 | 1.14E-07 |
| GLO1 | 0.2440731 | 1.72E-08 | 1.15E-07 |
| C21orf71 | 0.2440669 | 1.72E-08 | 1.15E-07 |
| RPS13 | 0.2440543 | 1.73E-08 | 1.15E-07 |
| TMCC2 | -0.2440445 | 1.73E-08 | 1.15E-07 |
| POLR2A | -0.2439949 | 1.74E-08 | 1.16E-07 |
| UQCRB | 0.2439175 | 1.76E-08 | 1.17E-07 |
| RAD54L2 | -0.2438766 | 1.77E-08 | 1.17E-07 |
| DDX25 | 0.2438481 | 1.78E-08 | 1.18E-07 |
| DSG3 | -0.2438274 | 1.78E-08 | 1.18E-07 |
| LOC440944 | 0.2438102 | 1.79E-08 | 1.18E-07 |
| ARHGEF15 | -0.243799 | 1.79E-08 | 1.19E-07 |
| FOXJ2 | -0.2437932 | 1.79E-08 | 1.19E-07 |
| SULT1A3 | 0.2437887 | 1.79E-08 | 1.19E-07 |
| RPS27 | 0.2436605 | 1.82E-08 | 1.21E-07 |
| CCDC92 | -0.2436503 | 1.83E-08 | 1.21E-07 |
| KLK6 | -0.2435823 | 1.84E-08 | 1.22E-07 |
| DYNC1I2 | -0.2435814 | 1.84E-08 | 1.22E-07 |
| EIF2B3 | 0.2435357 | 1.85E-08 | 1.23E-07 |
| CHCHD6 | 0.2435254 | 1.86E-08 | 1.23E-07 |
| PLVAP | -0.2435023 | 1.86E-08 | 1.23E-07 |
| SDHAF1 | 0.2434961 | 1.87E-08 | 1.23E-07 |
| SERPINA9 | -0.2434936 | 1.87E-08 | 1.23E-07 |
| EPHB3 | -0.2434905 | 1.87E-08 | 1.23E-07 |
| DNALI1 | -0.2434708 | 1.87E-08 | 1.24E-07 |
| NNAT | -0.2433562 | 1.90E-08 | 1.25E-07 |
| C13orf29 | -0.2433178 | 1.91E-08 | 1.26E-07 |
| FILIP1 | -0.2433103 | 1.91E-08 | 1.26E-07 |
| RC3H2 | -0.2432961 | 1.92E-08 | 1.26E-07 |
| LOC541473 | 0.2432944 | 1.92E-08 | 1.26E-07 |
| TCTEX1D2 | 0.2432868 | 1.92E-08 | 1.26E-07 |
| ZDHHC12 | 0.2432301 | 1.93E-08 | 1.27E-07 |
| ADRA1D | -0.2431664 | 1.95E-08 | 1.28E-07 |
| C6orf174 | -0.2431624 | 1.95E-08 | 1.29E-07 |
| KCTD6 | 0.243145 | 1.96E-08 | 1.29E-07 |
| SMPD1 | -0.2431236 | 1.96E-08 | 1.29E-07 |
| POMP | 0.2431085 | 1.97E-08 | 1.29E-07 |
| SULF2 | -0.2430313 | 1.99E-08 | 1.31E-07 |
| SLURP1 | -0.2430239 | 1.99E-08 | 1.31E-07 |
| RGS4 | -0.2429987 | 2.00E-08 | 1.31E-07 |
| ZNF691 | 0.2429098 | 2.02E-08 | 1.33E-07 |
| C7orf23 | 0.2428943 | 2.03E-08 | 1.33E-07 |
| KLHDC10 | -0.2428856 | 2.03E-08 | 1.33E-07 |
| MYPN | -0.2428703 | 2.03E-08 | 1.33E-07 |
| TOP1P2 | 0.2428679 | 2.03E-08 | 1.33E-07 |
| CTSA | -0.2428398 | 2.04E-08 | 1.34E-07 |
| LIG4 | -0.2428212 | 2.05E-08 | 1.34E-07 |
| TGFB1I1 | -0.2428173 | 2.05E-08 | 1.34E-07 |
| NPRL2 | 0.2427935 | 2.05E-08 | 1.35E-07 |
| APLP2 | -0.2427119 | 2.08E-08 | 1.36E-07 |
| BMP6 | -0.2427006 | 2.08E-08 | 1.36E-07 |
| SYT13 | -0.2426881 | 2.08E-08 | 1.36E-07 |
| LGTN | 0.242668 | 2.09E-08 | 1.37E-07 |
| BMP4 | -0.2426472 | 2.10E-08 | 1.37E-07 |
| CTHRC1 | -0.2426468 | 2.10E-08 | 1.37E-07 |
| ACLY | -0.2426367 | 2.10E-08 | 1.37E-07 |
| SEC16A | -0.2426094 | 2.11E-08 | 1.38E-07 |
| ZNF134 | -0.2425636 | 2.12E-08 | 1.38E-07 |
| NBEA | -0.242548 | 2.13E-08 | 1.39E-07 |
| UTP15 | 0.2425213 | 2.13E-08 | 1.39E-07 |
| PABPC1L | 0.242511 | 2.14E-08 | 1.39E-07 |
| SLC12A4 | -0.2425075 | 2.14E-08 | 1.39E-07 |
| LRRCC1 | 0.2424888 | 2.14E-08 | 1.40E-07 |
| PRKAR2A | -0.2424278 | 2.16E-08 | 1.41E-07 |
| ATP5I | 0.2424171 | 2.16E-08 | 1.41E-07 |
| CNIH3 | -0.2424136 | 2.16E-08 | 1.41E-07 |
| C20orf72 | 0.2423961 | 2.17E-08 | 1.41E-07 |
| SEMA3D | -0.2423892 | 2.17E-08 | 1.41E-07 |
| C12orf62 | 0.2423814 | 2.17E-08 | 1.41E-07 |
| LOC144486 | 0.2423468 | 2.18E-08 | 1.42E-07 |
| MMRN2 | -0.2423461 | 2.18E-08 | 1.42E-07 |
| TADA1 | 0.2423264 | 2.19E-08 | 1.42E-07 |
| HSPB8 | -0.2423245 | 2.19E-08 | 1.42E-07 |
| GSDMB | 0.242289 | 2.20E-08 | 1.43E-07 |
| SPTLC3 | -0.2422288 | 2.22E-08 | 1.44E-07 |
| TSSC1 | 0.242221 | 2.22E-08 | 1.44E-07 |
| RNF128 | -0.2421205 | 2.25E-08 | 1.46E-07 |
| MAP3K3 | -0.2420994 | 2.26E-08 | 1.47E-07 |
| SPCS1 | 0.2420973 | 2.26E-08 | 1.47E-07 |
| UCK1 | 0.2420877 | 2.26E-08 | 1.47E-07 |
| MXRA7 | -0.2420643 | 2.27E-08 | 1.47E-07 |
| BTBD7 | -0.2420376 | 2.28E-08 | 1.48E-07 |
| FLRT3 | -0.2419867 | 2.30E-08 | 1.49E-07 |
| GPR89C | 0.2419123 | 2.32E-08 | 1.50E-07 |
| HSPBP1 | 0.2418761 | 2.33E-08 | 1.51E-07 |
| GPR173 | -0.2418713 | 2.33E-08 | 1.51E-07 |
| SNHG10 | 0.2418535 | 2.34E-08 | 1.51E-07 |
| RSL1D1 | 0.241843 | 2.34E-08 | 1.51E-07 |
| KIAA0240 | -0.2418327 | 2.34E-08 | 1.52E-07 |
| APOBEC3B | 0.2418167 | 2.35E-08 | 1.52E-07 |
| PIK3R1 | -0.241722 | 2.38E-08 | 1.54E-07 |
| FANCM | 0.2417106 | 2.38E-08 | 1.54E-07 |
| C16orf93 | 0.2417104 | 2.38E-08 | 1.54E-07 |
| BRD4 | -0.2416772 | 2.39E-08 | 1.55E-07 |
| FLJ23867 | -0.2416472 | 2.40E-08 | 1.55E-07 |
| GOLGA4 | -0.2416168 | 2.41E-08 | 1.56E-07 |
| PRR13 | 0.2416143 | 2.42E-08 | 1.56E-07 |
| HBP1 | -0.2416117 | 2.42E-08 | 1.56E-07 |
| NKAIN4 | -0.2415861 | 2.42E-08 | 1.56E-07 |
| FAM134B | -0.2414763 | 2.46E-08 | 1.59E-07 |
| TBC1D10A | -0.2414434 | 2.47E-08 | 1.59E-07 |
| FHL1 | -0.2414221 | 2.48E-08 | 1.60E-07 |
| ZDHHC5 | -0.241405 | 2.49E-08 | 1.60E-07 |
| GABARAPL1 | -0.2413879 | 2.49E-08 | 1.60E-07 |
| CARD18 | -0.2413207 | 2.51E-08 | 1.62E-07 |
| CCDC28A | 0.2412958 | 2.52E-08 | 1.62E-07 |
| MYL4 | -0.2412434 | 2.54E-08 | 1.63E-07 |
| KIAA1274 | -0.2412291 | 2.55E-08 | 1.64E-07 |
| CKMT2 | -0.2411613 | 2.57E-08 | 1.65E-07 |
| MYH1 | -0.2411526 | 2.57E-08 | 1.65E-07 |
| ATIC | 0.2411489 | 2.57E-08 | 1.65E-07 |
| FOXD2 | 0.2411314 | 2.58E-08 | 1.66E-07 |
| TLN1 | -0.2411225 | 2.58E-08 | 1.66E-07 |
| COPB2 | -0.2410811 | 2.60E-08 | 1.67E-07 |
| ADAMTS1 | -0.241051 | 2.61E-08 | 1.67E-07 |
| ISY1 | 0.241 | 2.63E-08 | 1.68E-07 |
| C17orf81 | 0.2408667 | 2.68E-08 | 1.71E-07 |
| NOMO2 | -0.2408612 | 2.68E-08 | 1.71E-07 |
| C14orf169 | 0.2408442 | 2.68E-08 | 1.72E-07 |
| IL6ST | -0.2408407 | 2.68E-08 | 1.72E-07 |
| ISPD | -0.240802 | 2.70E-08 | 1.73E-07 |
| DCLK3 | -0.2407895 | 2.70E-08 | 1.73E-07 |
| SEH1L | 0.240781 | 2.71E-08 | 1.73E-07 |
| C1orf150 | -0.2407466 | 2.72E-08 | 1.74E-07 |
| LAMB1 | -0.2407147 | 2.73E-08 | 1.75E-07 |
| HSPC159 | -0.2406589 | 2.75E-08 | 1.76E-07 |
| CSNK2A1P | -0.2406294 | 2.76E-08 | 1.76E-07 |
| AKAP2 | -0.240611 | 2.77E-08 | 1.77E-07 |
| RALGAPB | -0.2405818 | 2.78E-08 | 1.78E-07 |
| SIAE | -0.2405266 | 2.80E-08 | 1.79E-07 |
| SUMO1P3 | 0.2405154 | 2.81E-08 | 1.79E-07 |
| BTRC | -0.2404622 | 2.83E-08 | 1.80E-07 |
| C12orf60 | 0.2404598 | 2.83E-08 | 1.80E-07 |
| METTL11A | 0.240458 | 2.83E-08 | 1.80E-07 |
| ASXL2 | -0.2404413 | 2.83E-08 | 1.81E-07 |
| NMI | 0.2403948 | 2.85E-08 | 1.82E-07 |
| PPIB | 0.2403774 | 2.86E-08 | 1.82E-07 |
| DLL4 | -0.2403633 | 2.86E-08 | 1.82E-07 |
| SNX21 | -0.2402925 | 2.89E-08 | 1.84E-07 |
| ZNF90 | 0.2402642 | 2.90E-08 | 1.85E-07 |
| HEXDC | 0.2402244 | 2.92E-08 | 1.86E-07 |
| AIPL1 | 0.2401779 | 2.94E-08 | 1.87E-07 |
| ATP5A1 | 0.2401753 | 2.94E-08 | 1.87E-07 |
| PNN | 0.2401501 | 2.95E-08 | 1.87E-07 |
| NLGN4X | -0.2401222 | 2.96E-08 | 1.88E-07 |
| GLRX5 | 0.2401156 | 2.96E-08 | 1.88E-07 |
| PHKG2 | 0.2401052 | 2.97E-08 | 1.88E-07 |
| PLEKHG4B | -0.2401034 | 2.97E-08 | 1.88E-07 |
| C19orf62 | 0.2400566 | 2.99E-08 | 1.89E-07 |
| C10orf84 | 0.2400102 | 3.01E-08 | 1.91E-07 |
| RCN3 | -0.2400093 | 3.01E-08 | 1.91E-07 |
| AKAP11 | -0.2400072 | 3.01E-08 | 1.91E-07 |
| ADAMTS9 | -0.2399959 | 3.01E-08 | 1.91E-07 |
| RNF180 | -0.2399902 | 3.01E-08 | 1.91E-07 |
| C10orf35 | 0.2399202 | 3.04E-08 | 1.93E-07 |
| METTL5 | 0.2399152 | 3.04E-08 | 1.93E-07 |
| RAD1 | 0.2399045 | 3.05E-08 | 1.93E-07 |
| PGM3 | -0.2398723 | 3.06E-08 | 1.94E-07 |
| RSPH9 | -0.2398178 | 3.09E-08 | 1.95E-07 |
| COPS7B | 0.2397198 | 3.13E-08 | 1.98E-07 |
| MED13L | -0.239708 | 3.13E-08 | 1.98E-07 |
| ZNF167 | -0.2397053 | 3.13E-08 | 1.98E-07 |
| EIF3M | 0.2396812 | 3.14E-08 | 1.98E-07 |
| SSFA2 | -0.2396767 | 3.14E-08 | 1.99E-07 |
| CLIC4 | -0.2396385 | 3.16E-08 | 2.00E-07 |
| SURF4 | -0.2395826 | 3.19E-08 | 2.01E-07 |
| CDH2 | -0.2394887 | 3.23E-08 | 2.03E-07 |
| FLG2 | -0.2394667 | 3.24E-08 | 2.04E-07 |
| SERPINF1 | -0.2394421 | 3.25E-08 | 2.05E-07 |
| SCG2 | -0.2394261 | 3.25E-08 | 2.05E-07 |
| ARID5B | -0.23939 | 3.27E-08 | 2.06E-07 |
| SLC16A4 | -0.2393864 | 3.27E-08 | 2.06E-07 |
| ADAT1 | -0.2393622 | 3.28E-08 | 2.07E-07 |
| NRF1 | 0.2392607 | 3.33E-08 | 2.09E-07 |
| FAM38B | -0.2392413 | 3.34E-08 | 2.10E-07 |
| RMND1 | 0.2392328 | 3.34E-08 | 2.10E-07 |
| BMP2K | -0.2392277 | 3.34E-08 | 2.10E-07 |
| ZNF620 | 0.2392092 | 3.35E-08 | 2.11E-07 |
| MEGF10 | -0.239197 | 3.36E-08 | 2.11E-07 |
| AP4B1 | 0.2391498 | 3.38E-08 | 2.12E-07 |
| PCDHB10 | -0.2390506 | 3.42E-08 | 2.15E-07 |
| TOR1AIP2 | -0.2390445 | 3.43E-08 | 2.15E-07 |
| CISD1 | 0.2390383 | 3.43E-08 | 2.15E-07 |
| CTRL | 0.2390174 | 3.44E-08 | 2.16E-07 |
| RNF220 | 0.2390133 | 3.44E-08 | 2.16E-07 |
| MYO10 | -0.2389345 | 3.48E-08 | 2.18E-07 |
| PDE1A | -0.2389161 | 3.49E-08 | 2.19E-07 |
| BIRC6 | -0.238868 | 3.51E-08 | 2.20E-07 |
| CYP26B1 | -0.2388617 | 3.51E-08 | 2.20E-07 |
| FUNDC1 | 0.2388425 | 3.52E-08 | 2.20E-07 |
| TRPS1 | -0.2388281 | 3.53E-08 | 2.21E-07 |
| MIS12 | 0.2388012 | 3.54E-08 | 2.22E-07 |
| CRIPT | 0.238797 | 3.54E-08 | 2.22E-07 |
| TTC28 | -0.2387705 | 3.56E-08 | 2.22E-07 |
| C14orf106 | 0.2387701 | 3.56E-08 | 2.22E-07 |
| PSMB9 | 0.2387695 | 3.56E-08 | 2.22E-07 |
| HRH2 | -0.2387192 | 3.58E-08 | 2.24E-07 |
| RSC1A1 | -0.2386627 | 3.61E-08 | 2.25E-07 |
| FOXRED1 | 0.2386581 | 3.61E-08 | 2.25E-07 |
| SCN3A | -0.2386546 | 3.61E-08 | 2.25E-07 |
| KIAA2018 | -0.2386368 | 3.62E-08 | 2.26E-07 |
| SFRP4 | -0.2385691 | 3.65E-08 | 2.28E-07 |
| FAM58A | 0.238567 | 3.65E-08 | 2.28E-07 |
| MYOM1 | -0.2385669 | 3.65E-08 | 2.28E-07 |
| CCDC41 | 0.2385487 | 3.66E-08 | 2.28E-07 |
| PRPF4 | 0.2385379 | 3.67E-08 | 2.29E-07 |
| ZFYVE26 | -0.2385363 | 3.67E-08 | 2.29E-07 |
| FMO5 | -0.2385337 | 3.67E-08 | 2.29E-07 |
| PCDHB6 | -0.2385204 | 3.68E-08 | 2.29E-07 |
| HK1 | -0.2384852 | 3.70E-08 | 2.30E-07 |
| RSF1 | -0.238451 | 3.71E-08 | 2.31E-07 |
| AP3M2 | 0.238437 | 3.72E-08 | 2.31E-07 |
| HCN2 | -0.2383996 | 3.74E-08 | 2.32E-07 |
| TSC22D2 | -0.2383956 | 3.74E-08 | 2.32E-07 |
| ELP3 | 0.2383662 | 3.75E-08 | 2.33E-07 |
| AKR7A3 | 0.2383593 | 3.76E-08 | 2.33E-07 |
| CDO1 | -0.238357 | 3.76E-08 | 2.33E-07 |
| KLHL36 | -0.2383454 | 3.77E-08 | 2.34E-07 |
| ZFYVE28 | -0.2383118 | 3.78E-08 | 2.35E-07 |
| MTERFD1 | 0.2383017 | 3.79E-08 | 2.35E-07 |
| TCTEX1D1 | -0.2382974 | 3.79E-08 | 2.35E-07 |
| RPL26 | 0.2382424 | 3.82E-08 | 2.37E-07 |
| EIF2S3 | 0.2382417 | 3.82E-08 | 2.37E-07 |
| ZFPM2 | -0.238073 | 3.91E-08 | 2.42E-07 |
| MYCT1 | -0.2380576 | 3.91E-08 | 2.42E-07 |
| CREBBP | -0.2380522 | 3.92E-08 | 2.43E-07 |
| PPP1R3A | -0.2380217 | 3.93E-08 | 2.44E-07 |
| RPL31 | 0.2380137 | 3.94E-08 | 2.44E-07 |
| SECISBP2L | -0.2380115 | 3.94E-08 | 2.44E-07 |
| GDF6 | -0.2379795 | 3.96E-08 | 2.45E-07 |
| HPGDS | -0.2379758 | 3.96E-08 | 2.45E-07 |
| PNRC2 | 0.2379221 | 3.99E-08 | 2.46E-07 |
| ATP1A2 | -0.237869 | 4.02E-08 | 2.48E-07 |
| C7orf36 | 0.2378584 | 4.02E-08 | 2.48E-07 |
| RPS11 | 0.2378186 | 4.04E-08 | 2.50E-07 |
| RPL38 | 0.2377594 | 4.07E-08 | 2.52E-07 |
| RORA | -0.2377178 | 4.10E-08 | 2.53E-07 |
| ARHGAP6 | -0.2375299 | 4.20E-08 | 2.59E-07 |
| C20orf112 | -0.2375037 | 4.22E-08 | 2.60E-07 |
| SNAI1 | -0.2374878 | 4.23E-08 | 2.61E-07 |
| LOC146880 | 0.2374716 | 4.24E-08 | 2.61E-07 |
| DLX3 | -0.2374716 | 4.24E-08 | 2.61E-07 |
| MYL3 | -0.2374506 | 4.25E-08 | 2.62E-07 |
| MAN2A1 | -0.2374072 | 4.27E-08 | 2.63E-07 |
| FAM63B | -0.2373803 | 4.29E-08 | 2.64E-07 |
| PRDM16 | -0.237315 | 4.33E-08 | 2.66E-07 |
| ATF6 | -0.2373002 | 4.33E-08 | 2.67E-07 |
| SASH1 | -0.2372699 | 4.35E-08 | 2.68E-07 |
| GLI2 | -0.2372381 | 4.37E-08 | 2.69E-07 |
| GCC2 | -0.2372236 | 4.38E-08 | 2.69E-07 |
| AP1S1 | 0.2372045 | 4.39E-08 | 2.70E-07 |
| STRN3 | -0.2371858 | 4.40E-08 | 2.70E-07 |
| CD1B | -0.237174 | 4.41E-08 | 2.71E-07 |
| ATF7IP | -0.2371452 | 4.43E-08 | 2.72E-07 |
| ZNF720 | -0.2371183 | 4.44E-08 | 2.73E-07 |
| TMEM233 | -0.2370888 | 4.46E-08 | 2.74E-07 |
| CMTM3 | -0.2370091 | 4.51E-08 | 2.76E-07 |
| CCDC48 | -0.2369966 | 4.51E-08 | 2.77E-07 |
| TRIM63 | -0.2369843 | 4.52E-08 | 2.77E-07 |
| EDEM3 | -0.2369308 | 4.55E-08 | 2.79E-07 |
| DYNLT3 | -0.2368736 | 4.59E-08 | 2.81E-07 |
| TIAM2 | -0.2368075 | 4.63E-08 | 2.84E-07 |
| WNT2 | -0.2367745 | 4.65E-08 | 2.85E-07 |
| MATN2 | -0.2367578 | 4.66E-08 | 2.85E-07 |
| TENC1 | -0.2367524 | 4.66E-08 | 2.85E-07 |
| ZNF704 | -0.236744 | 4.67E-08 | 2.86E-07 |
| ING5 | 0.2367303 | 4.68E-08 | 2.86E-07 |
| IDE | -0.236685 | 4.71E-08 | 2.88E-07 |
| SPIN2A | -0.2366849 | 4.71E-08 | 2.88E-07 |
| GADD45GIP1 | 0.2365684 | 4.78E-08 | 2.92E-07 |
| ADCY9 | -0.2365289 | 4.81E-08 | 2.94E-07 |
| HIST1H2BH | 0.236508 | 4.82E-08 | 2.94E-07 |
| RPS10P7 | 0.2364931 | 4.83E-08 | 2.95E-07 |
| FURIN | -0.2364916 | 4.83E-08 | 2.95E-07 |
| NSUN5P1 | 0.2364828 | 4.84E-08 | 2.95E-07 |
| C20orf123 | -0.2364492 | 4.86E-08 | 2.96E-07 |
| BMX | -0.236405 | 4.89E-08 | 2.98E-07 |
| CWH43 | -0.2363435 | 4.93E-08 | 3.00E-07 |
| TLX3 | 0.2363107 | 4.95E-08 | 3.02E-07 |
| NSUN5P2 | 0.2362987 | 4.96E-08 | 3.02E-07 |
| RPS16 | 0.236198 | 5.02E-08 | 3.06E-07 |
| KARS | 0.2361547 | 5.05E-08 | 3.08E-07 |
| B3GNT9 | -0.2360601 | 5.12E-08 | 3.11E-07 |
| CCDC149 | -0.2360497 | 5.12E-08 | 3.12E-07 |
| CDC23 | 0.2360047 | 5.15E-08 | 3.14E-07 |
| C20orf200 | -0.2359737 | 5.18E-08 | 3.15E-07 |
| MVP | -0.2359303 | 5.21E-08 | 3.16E-07 |
| GPR176 | -0.2358861 | 5.24E-08 | 3.18E-07 |
| FASTKD3 | 0.2358631 | 5.25E-08 | 3.19E-07 |
| USO1 | -0.2358417 | 5.27E-08 | 3.20E-07 |
| C6orf186 | -0.2358122 | 5.29E-08 | 3.21E-07 |
| LOC100129550 | -0.2358088 | 5.29E-08 | 3.21E-07 |
| RPL27A | 0.2358047 | 5.29E-08 | 3.21E-07 |
| SATB1 | -0.2357986 | 5.30E-08 | 3.21E-07 |
| MLL | -0.2357901 | 5.30E-08 | 3.22E-07 |
| ZNF264 | -0.2357865 | 5.31E-08 | 3.22E-07 |
| LTBP1 | -0.2357848 | 5.31E-08 | 3.22E-07 |
| NCRNA00201 | 0.2357695 | 5.32E-08 | 3.22E-07 |
| DKK2 | -0.2357516 | 5.33E-08 | 3.23E-07 |
| ACVRL1 | -0.235747 | 5.33E-08 | 3.23E-07 |
| PLCE1 | -0.2357345 | 5.34E-08 | 3.23E-07 |
| RAB5B | -0.235719 | 5.35E-08 | 3.24E-07 |
| EIF2C4 | -0.2357168 | 5.36E-08 | 3.24E-07 |
| TMEM200A | -0.2356734 | 5.39E-08 | 3.26E-07 |
| C6orf203 | 0.2356031 | 5.44E-08 | 3.29E-07 |
| CYP2D6 | 0.2355805 | 5.45E-08 | 3.30E-07 |
| TMEM43 | -0.2355573 | 5.47E-08 | 3.31E-07 |
| CES7 | -0.2354851 | 5.52E-08 | 3.34E-07 |
| TNRC6B | -0.2354774 | 5.53E-08 | 3.34E-07 |
| DLG4 | -0.2354458 | 5.55E-08 | 3.35E-07 |
| ZNF473 | 0.2354331 | 5.56E-08 | 3.36E-07 |
| SEC61B | 0.2353588 | 5.62E-08 | 3.39E-07 |
| LOC256880 | 0.2353318 | 5.64E-08 | 3.40E-07 |
| HESX1 | 0.235308 | 5.66E-08 | 3.41E-07 |
| BAT4 | 0.2352787 | 5.68E-08 | 3.42E-07 |
| SORCS1 | -0.2352679 | 5.69E-08 | 3.43E-07 |
| SMPD4 | 0.2352585 | 5.69E-08 | 3.43E-07 |
| STAB2 | -0.2352024 | 5.74E-08 | 3.45E-07 |
| CCNO | 0.2351625 | 5.77E-08 | 3.47E-07 |
| CCDC94 | 0.235153 | 5.77E-08 | 3.48E-07 |
| KLK5 | -0.2351126 | 5.80E-08 | 3.49E-07 |
| PCDHB3 | -0.2350811 | 5.83E-08 | 3.51E-07 |
| KIAA1210 | -0.2349554 | 5.93E-08 | 3.56E-07 |
| COX6B1 | 0.2349397 | 5.94E-08 | 3.57E-07 |
| LTA4H | 0.234932 | 5.95E-08 | 3.57E-07 |
| SNX5 | 0.2349235 | 5.95E-08 | 3.58E-07 |
| PLP1 | -0.2349184 | 5.96E-08 | 3.58E-07 |
| OSGEPL1 | 0.2348997 | 5.97E-08 | 3.59E-07 |
| XRCC6 | 0.234889 | 5.98E-08 | 3.59E-07 |
| STON1-GTF2A1L | -0.2348841 | 5.98E-08 | 3.59E-07 |
| FAM189A1 | -0.2348629 | 6.00E-08 | 3.60E-07 |
| RGPD4 | -0.234855 | 6.01E-08 | 3.60E-07 |
| ZNF415 | -0.234821 | 6.03E-08 | 3.62E-07 |
| TGOLN2 | -0.2348178 | 6.04E-08 | 3.62E-07 |
| SMARCE1 | 0.2347641 | 6.08E-08 | 3.64E-07 |
| DHRS7B | 0.2347628 | 6.08E-08 | 3.64E-07 |
| C12orf51 | -0.2347237 | 6.11E-08 | 3.66E-07 |
| ZC3H7B | -0.2347196 | 6.12E-08 | 3.66E-07 |
| CUBN | -0.2346488 | 6.17E-08 | 3.69E-07 |
| YBX2 | 0.234643 | 6.18E-08 | 3.70E-07 |
| PPARGC1A | -0.234634 | 6.19E-08 | 3.70E-07 |
| DOPEY1 | -0.2345624 | 6.24E-08 | 3.73E-07 |
| GNB2L1 | 0.2345623 | 6.24E-08 | 3.73E-07 |
| ELP4 | 0.2345337 | 6.27E-08 | 3.75E-07 |
| IPO13 | -0.2345149 | 6.28E-08 | 3.75E-07 |
| CES1 | -0.2344995 | 6.30E-08 | 3.76E-07 |
| ASH2L | 0.2344968 | 6.30E-08 | 3.76E-07 |
| FBXW9 | 0.2344878 | 6.31E-08 | 3.76E-07 |
| FLT1 | -0.2344829 | 6.31E-08 | 3.77E-07 |
| SLC1A4 | -0.2344007 | 6.38E-08 | 3.81E-07 |
| SLFN13 | 0.2343736 | 6.40E-08 | 3.82E-07 |
| TMEM60 | 0.2343571 | 6.42E-08 | 3.83E-07 |
| NBPF10 | -0.2343318 | 6.44E-08 | 3.84E-07 |
| CHI3L1 | -0.2343148 | 6.45E-08 | 3.84E-07 |
| HRC | -0.2342357 | 6.52E-08 | 3.88E-07 |
| DPYSL4 | -0.2342012 | 6.55E-08 | 3.90E-07 |
| KRT16 | -0.2341909 | 6.56E-08 | 3.91E-07 |
| SEC24B | -0.2341779 | 6.57E-08 | 3.91E-07 |
| PDGFRL | -0.2341351 | 6.61E-08 | 3.93E-07 |
| ARPP21 | -0.2341076 | 6.63E-08 | 3.94E-07 |
| NOTUM | -0.2340918 | 6.65E-08 | 3.95E-07 |
| SMYD2 | 0.2340192 | 6.71E-08 | 3.99E-07 |
| PIN1 | 0.2339216 | 6.80E-08 | 4.04E-07 |
| C7orf41 | -0.23392 | 6.80E-08 | 4.04E-07 |
| HCG27 | 0.2338247 | 6.89E-08 | 4.09E-07 |
| KIAA1539 | -0.2337896 | 6.92E-08 | 4.11E-07 |
| SUFU | -0.2337828 | 6.92E-08 | 4.11E-07 |
| SEMA6C | -0.2337777 | 6.93E-08 | 4.11E-07 |
| RPL30 | 0.2337272 | 6.97E-08 | 4.14E-07 |
| ZDHHC7 | -0.2336911 | 7.01E-08 | 4.16E-07 |
| KLK9 | -0.2336378 | 7.06E-08 | 4.18E-07 |
| TBCD | 0.2335774 | 7.11E-08 | 4.22E-07 |
| MGC12982 | 0.2335597 | 7.13E-08 | 4.22E-07 |
| DSP | -0.2335302 | 7.16E-08 | 4.24E-07 |
| PRPF31 | 0.2335156 | 7.17E-08 | 4.25E-07 |
| ARID1B | -0.2334936 | 7.19E-08 | 4.26E-07 |
| AP1B1 | -0.233472 | 7.21E-08 | 4.27E-07 |
| ADCYAP1R1 | -0.2334119 | 7.27E-08 | 4.30E-07 |
| SF4 | 0.2333726 | 7.31E-08 | 4.32E-07 |
| IPMK | -0.2333577 | 7.32E-08 | 4.33E-07 |
| CSPG4 | -0.2333441 | 7.34E-08 | 4.34E-07 |
| SPRYD3 | -0.233329 | 7.35E-08 | 4.34E-07 |
| PCDHB8 | -0.2332905 | 7.39E-08 | 4.36E-07 |
| CD34 | -0.2332751 | 7.40E-08 | 4.37E-07 |
| DNTTIP1 | 0.2332706 | 7.41E-08 | 4.37E-07 |
| PIK3C2B | -0.2332657 | 7.41E-08 | 4.38E-07 |
| PPIAL4D | 0.2332421 | 7.44E-08 | 4.39E-07 |
| NELL1 | -0.2331414 | 7.53E-08 | 4.44E-07 |
| TMEM181 | -0.2330951 | 7.58E-08 | 4.47E-07 |
| FGFRL1 | -0.2330451 | 7.63E-08 | 4.50E-07 |
| RRP9 | 0.2330363 | 7.64E-08 | 4.50E-07 |
| ATP2B4 | -0.2330319 | 7.64E-08 | 4.50E-07 |
| RPS5 | 0.2330125 | 7.66E-08 | 4.51E-07 |
| GRIN2C | 0.233008 | 7.67E-08 | 4.51E-07 |
| ATP5E | 0.2330072 | 7.67E-08 | 4.51E-07 |
| CCDC53 | 0.2329785 | 7.70E-08 | 4.53E-07 |
| BOD1 | 0.2329283 | 7.75E-08 | 4.56E-07 |
| INTS9 | 0.2329258 | 7.75E-08 | 4.56E-07 |
| KIAA1755 | -0.2329154 | 7.76E-08 | 4.56E-07 |
| TSKU | -0.2329135 | 7.76E-08 | 4.56E-07 |
| ABHD11 | 0.2328564 | 7.82E-08 | 4.60E-07 |
| NCRNA00219 | 0.2328472 | 7.83E-08 | 4.60E-07 |
| MUM1L1 | -0.2328431 | 7.84E-08 | 4.60E-07 |
| CMA1 | -0.2327991 | 7.88E-08 | 4.63E-07 |
| RRAGC | -0.2327793 | 7.90E-08 | 4.64E-07 |
| CCT3 | 0.2326959 | 7.99E-08 | 4.69E-07 |
| EYA4 | -0.2326915 | 7.99E-08 | 4.69E-07 |
| NR4A3 | -0.2326906 | 8.00E-08 | 4.69E-07 |
| MAD2L2 | 0.2326654 | 8.02E-08 | 4.70E-07 |
| SRL | -0.2326568 | 8.03E-08 | 4.71E-07 |
| C11orf95 | -0.2325814 | 8.11E-08 | 4.75E-07 |
| GUCA1A | -0.2325216 | 8.17E-08 | 4.79E-07 |
| SFRS16 | 0.2324829 | 8.22E-08 | 4.81E-07 |
| COL11A1 | -0.2324817 | 8.22E-08 | 4.81E-07 |
| UPB1 | 0.2324571 | 8.24E-08 | 4.83E-07 |
| TLL1 | -0.2324223 | 8.28E-08 | 4.85E-07 |
| LEPR | -0.232413 | 8.29E-08 | 4.85E-07 |
| NDUFB7 | 0.2323901 | 8.32E-08 | 4.86E-07 |
| TOPBP1 | 0.2323834 | 8.32E-08 | 4.87E-07 |
| SNX1 | 0.2323662 | 8.34E-08 | 4.88E-07 |
| LIN54 | 0.2323617 | 8.35E-08 | 4.88E-07 |
| PRRX1 | -0.232331 | 8.38E-08 | 4.90E-07 |
| DLX2 | -0.2322874 | 8.43E-08 | 4.92E-07 |
| USP46 | -0.2321808 | 8.55E-08 | 4.99E-07 |
| TM2D3 | 0.2321757 | 8.55E-08 | 4.99E-07 |
| C9orf123 | 0.2321495 | 8.58E-08 | 5.01E-07 |
| CNTD1 | 0.2321451 | 8.59E-08 | 5.01E-07 |
| ARG1 | -0.2321332 | 8.60E-08 | 5.02E-07 |
| MAP4K5 | -0.2320574 | 8.69E-08 | 5.06E-07 |
| TRDN | -0.2320323 | 8.72E-08 | 5.08E-07 |
| HIST1H2BJ | 0.2320143 | 8.74E-08 | 5.09E-07 |
| CBWD6 | 0.2320095 | 8.74E-08 | 5.09E-07 |
| MLX | 0.2319979 | 8.76E-08 | 5.10E-07 |
| AHCYL2 | -0.2319275 | 8.84E-08 | 5.14E-07 |
| AADACL2 | -0.2319185 | 8.85E-08 | 5.15E-07 |
| C1QTNF6 | -0.2319038 | 8.86E-08 | 5.16E-07 |
| AGAP4 | 0.2318901 | 8.88E-08 | 5.16E-07 |
| ZNF266 | 0.231851 | 8.93E-08 | 5.19E-07 |
| MAPRE2 | -0.231848 | 8.93E-08 | 5.19E-07 |
| UTP14C | -0.2318045 | 8.98E-08 | 5.22E-07 |
| ACTN1 | -0.2317953 | 8.99E-08 | 5.22E-07 |
| GNPDA1 | 0.2317809 | 9.01E-08 | 5.23E-07 |
| TBC1D8B | -0.2317765 | 9.01E-08 | 5.23E-07 |
| CTDSPL2 | 0.2317559 | 9.04E-08 | 5.25E-07 |
| MRPL48 | 0.2316244 | 9.20E-08 | 5.34E-07 |
| MAPK1 | -0.2316007 | 9.22E-08 | 5.35E-07 |
| TFB1M | 0.2315543 | 9.28E-08 | 5.38E-07 |
| SHC1 | -0.2315265 | 9.31E-08 | 5.40E-07 |
| RASA4 | -0.2314887 | 9.36E-08 | 5.42E-07 |
| SYVN1 | -0.2314748 | 9.38E-08 | 5.43E-07 |
| SLC25A10 | 0.2314693 | 9.38E-08 | 5.44E-07 |
| DPY19L3 | -0.231465 | 9.39E-08 | 5.44E-07 |
| WBSCR22 | 0.2314121 | 9.45E-08 | 5.47E-07 |
| NFATC2IP | 0.231379 | 9.49E-08 | 5.50E-07 |
| JTB | 0.2313428 | 9.54E-08 | 5.52E-07 |
| RGS22 | -0.2313275 | 9.56E-08 | 5.53E-07 |
| RPL19 | 0.231322 | 9.57E-08 | 5.53E-07 |
| MRPL20 | 0.2313207 | 9.57E-08 | 5.53E-07 |
| BRAF | -0.2313142 | 9.58E-08 | 5.53E-07 |
| PDAP1 | 0.2313108 | 9.58E-08 | 5.53E-07 |
| PARL | 0.2313003 | 9.59E-08 | 5.54E-07 |
| CROCCL1 | 0.2312668 | 9.64E-08 | 5.56E-07 |
| ITPRIP | -0.2312375 | 9.67E-08 | 5.58E-07 |
| ZNF610 | -0.2311962 | 9.72E-08 | 5.61E-07 |
| MRPL28 | 0.2311124 | 9.83E-08 | 5.67E-07 |
| CDC25B | 0.2311064 | 9.84E-08 | 5.67E-07 |
| PRELID2 | 0.2310891 | 9.86E-08 | 5.69E-07 |
| FCER1A | -0.2310835 | 9.87E-08 | 5.69E-07 |
| CA6 | -0.2310269 | 9.94E-08 | 5.73E-07 |
| ADARB1 | -0.2310203 | 9.95E-08 | 5.73E-07 |
| KDM1B | -0.23102 | 9.95E-08 | 5.73E-07 |
| ZNF699 | -0.2310091 | 9.96E-08 | 5.74E-07 |
| CLSPN | 0.230988 | 9.99E-08 | 5.75E-07 |
| CD300LB | -0.2309694 | 1.00E-07 | 5.76E-07 |
| PRSS35 | -0.2308747 | 1.01E-07 | 5.83E-07 |
| SMNDC1 | 0.2308683 | 1.01E-07 | 5.84E-07 |
| ROS1 | -0.2308438 | 1.02E-07 | 5.85E-07 |
| NOL10 | 0.230795 | 1.02E-07 | 5.89E-07 |
| C1orf59 | 0.2307779 | 1.03E-07 | 5.90E-07 |
| C15orf29 | 0.2306652 | 1.04E-07 | 5.99E-07 |
| RHBDF1 | -0.2305812 | 1.05E-07 | 6.05E-07 |
| GEM | -0.2305811 | 1.05E-07 | 6.05E-07 |
| LY6G6C | -0.2305608 | 1.06E-07 | 6.06E-07 |
| LIMS2 | -0.2305524 | 1.06E-07 | 6.07E-07 |
| NUBP2 | 0.230511 | 1.06E-07 | 6.10E-07 |
| TNNI1 | -0.2305066 | 1.06E-07 | 6.10E-07 |
| SEC24C | -0.2304761 | 1.07E-07 | 6.12E-07 |
| BPTF | -0.2304683 | 1.07E-07 | 6.13E-07 |
| PSMB8 | 0.2304484 | 1.07E-07 | 6.14E-07 |
| TP53INP1 | -0.230403 | 1.08E-07 | 6.18E-07 |
| PRKAR1A | -0.2302703 | 1.10E-07 | 6.28E-07 |
| HDAC5 | -0.2302547 | 1.10E-07 | 6.29E-07 |
| SLC38A10 | -0.2302526 | 1.10E-07 | 6.29E-07 |
| RTEL1 | 0.2302437 | 1.10E-07 | 6.30E-07 |
| CDK6 | -0.23022 | 1.10E-07 | 6.32E-07 |
| ZRANB1 | -0.2301984 | 1.11E-07 | 6.33E-07 |
| RPS26 | 0.2301439 | 1.12E-07 | 6.38E-07 |
| HS3ST3B1 | -0.2300937 | 1.12E-07 | 6.42E-07 |
| NTHL1 | 0.2300177 | 1.13E-07 | 6.48E-07 |
| TMEM107 | 0.2299928 | 1.14E-07 | 6.50E-07 |
| VIT | -0.2299852 | 1.14E-07 | 6.50E-07 |
| ITGAV | -0.2299487 | 1.14E-07 | 6.53E-07 |
| C19orf53 | 0.2299478 | 1.14E-07 | 6.53E-07 |
| TMEM62 | 0.2299469 | 1.14E-07 | 6.53E-07 |
| USP34 | -0.2299271 | 1.15E-07 | 6.54E-07 |
| PRELP | -0.2299214 | 1.15E-07 | 6.55E-07 |
| RNF152 | -0.2299003 | 1.15E-07 | 6.56E-07 |
| TRMT1 | 0.2298396 | 1.16E-07 | 6.61E-07 |
| C2orf3 | 0.2298043 | 1.17E-07 | 6.64E-07 |
| SSH1 | -0.2297931 | 1.17E-07 | 6.65E-07 |
| LINS1 | 0.2297897 | 1.17E-07 | 6.65E-07 |
| ESYT2 | -0.2297879 | 1.17E-07 | 6.65E-07 |
| ARFGEF2 | -0.2297639 | 1.17E-07 | 6.67E-07 |
| CCDC73 | 0.2297494 | 1.17E-07 | 6.68E-07 |
| TMPPE | -0.2296938 | 1.18E-07 | 6.73E-07 |
| RPL23 | 0.2296524 | 1.19E-07 | 6.76E-07 |
| MTOR | -0.2296232 | 1.19E-07 | 6.78E-07 |
| TBX2 | -0.2295887 | 1.20E-07 | 6.81E-07 |
| TMEM93 | 0.2295856 | 1.20E-07 | 6.81E-07 |
| SAMD1 | 0.229582 | 1.20E-07 | 6.81E-07 |
| SMYD1 | -0.2295818 | 1.20E-07 | 6.81E-07 |
| EFR3A | -0.229513 | 1.21E-07 | 6.87E-07 |
| MOSC1 | 0.2294781 | 1.22E-07 | 6.90E-07 |
| C6orf108 | 0.2294385 | 1.22E-07 | 6.93E-07 |
| PSMC5 | 0.2294257 | 1.22E-07 | 6.94E-07 |
| C17orf63 | -0.2294006 | 1.23E-07 | 6.96E-07 |
| SHANK2 | -0.2292937 | 1.25E-07 | 7.06E-07 |
| AHDC1 | -0.2292832 | 1.25E-07 | 7.07E-07 |
| MYH6 | -0.2292521 | 1.25E-07 | 7.09E-07 |
| ZNF91 | -0.2292347 | 1.25E-07 | 7.11E-07 |
| NUP35 | 0.2292308 | 1.26E-07 | 7.11E-07 |
| ATP7B | -0.229212 | 1.26E-07 | 7.12E-07 |
| RPL36AL | 0.2291812 | 1.26E-07 | 7.15E-07 |
| TTC13 | 0.229181 | 1.26E-07 | 7.15E-07 |
| SAMD11 | -0.2291679 | 1.27E-07 | 7.16E-07 |
| CCDC85A | -0.2291664 | 1.27E-07 | 7.16E-07 |
| EDA2R | -0.2291617 | 1.27E-07 | 7.16E-07 |
| MRPL41 | 0.2291531 | 1.27E-07 | 7.17E-07 |
| C6orf25 | -0.2291285 | 1.27E-07 | 7.19E-07 |
| GPBP1 | 0.2290788 | 1.28E-07 | 7.23E-07 |
| PLN | -0.2290358 | 1.29E-07 | 7.27E-07 |
| MRPL53 | 0.2290332 | 1.29E-07 | 7.27E-07 |
| MFGE8 | -0.2290329 | 1.29E-07 | 7.27E-07 |
| SAFB | 0.2289808 | 1.30E-07 | 7.32E-07 |
| MORC4 | 0.2288067 | 1.33E-07 | 7.48E-07 |
| FRMPD1 | -0.228806 | 1.33E-07 | 7.48E-07 |
| OSMR | -0.2287915 | 1.33E-07 | 7.49E-07 |
| LYVE1 | -0.2287882 | 1.33E-07 | 7.49E-07 |
| MEOX2 | -0.2287748 | 1.33E-07 | 7.50E-07 |
| ZBTB46 | -0.2287138 | 1.34E-07 | 7.56E-07 |
| GCHFR | 0.2286816 | 1.35E-07 | 7.59E-07 |
| CCRN4L | -0.2286762 | 1.35E-07 | 7.59E-07 |
| PGLYRP4 | -0.228622 | 1.36E-07 | 7.64E-07 |
| CTTNBP2NL | -0.2285917 | 1.36E-07 | 7.67E-07 |
| FETUB | -0.228573 | 1.37E-07 | 7.69E-07 |
| NPTX2 | -0.2285482 | 1.37E-07 | 7.71E-07 |
| CSK | 0.228533 | 1.37E-07 | 7.72E-07 |
| CASKIN2 | -0.2285319 | 1.37E-07 | 7.72E-07 |
| BRP44L | 0.2285299 | 1.37E-07 | 7.72E-07 |
| SUPT4H1 | 0.2285279 | 1.37E-07 | 7.72E-07 |
| HIST1H4I | 0.2285158 | 1.38E-07 | 7.73E-07 |
| CCDC101 | 0.2284952 | 1.38E-07 | 7.75E-07 |
| CAV3 | -0.2284863 | 1.38E-07 | 7.76E-07 |
| BIN3 | 0.22847 | 1.39E-07 | 7.77E-07 |
| SCT | -0.2284689 | 1.39E-07 | 7.77E-07 |
| KIAA0020 | 0.2284257 | 1.39E-07 | 7.81E-07 |
| ACTL6A | 0.2284251 | 1.39E-07 | 7.81E-07 |
| ALPK2 | -0.228378 | 1.40E-07 | 7.86E-07 |
| FNIP1 | -0.2283587 | 1.41E-07 | 7.87E-07 |
| AMACR | -0.2283531 | 1.41E-07 | 7.88E-07 |
| AG2 | -0.228352 | 1.41E-07 | 7.88E-07 |
| COX4I1 | 0.2283377 | 1.41E-07 | 7.89E-07 |
| UTS2R | -0.2282815 | 1.42E-07 | 7.94E-07 |
| ZNF117 | -0.2282787 | 1.42E-07 | 7.94E-07 |
| ZNF737 | -0.2281942 | 1.44E-07 | 8.03E-07 |
| ANKRD35 | -0.2281899 | 1.44E-07 | 8.03E-07 |
| ITGBL1 | -0.2281633 | 1.44E-07 | 8.05E-07 |
| ZNF626 | -0.228162 | 1.44E-07 | 8.05E-07 |
| HIST1H4B | 0.2281262 | 1.45E-07 | 8.09E-07 |
| PAPL | -0.228093 | 1.45E-07 | 8.12E-07 |
| CLDN11 | -0.2280581 | 1.46E-07 | 8.16E-07 |
| MYO3B | -0.2280571 | 1.46E-07 | 8.16E-07 |
| SLAIN2 | -0.228028 | 1.47E-07 | 8.18E-07 |
| UQCR10 | 0.2280031 | 1.47E-07 | 8.21E-07 |
| ITGA7 | -0.2279101 | 1.49E-07 | 8.30E-07 |
| PALM2-AKAP2 | -0.2278916 | 1.49E-07 | 8.32E-07 |
| HSPB6 | -0.227857 | 1.50E-07 | 8.36E-07 |
| TMEM104 | -0.2278433 | 1.50E-07 | 8.37E-07 |
| KANK1 | -0.2278095 | 1.51E-07 | 8.40E-07 |
| FARSB | 0.2277986 | 1.51E-07 | 8.41E-07 |
| EGR3 | -0.2277982 | 1.51E-07 | 8.41E-07 |
| APOA1BP | 0.2277899 | 1.51E-07 | 8.42E-07 |
| FBXO32 | -0.2277802 | 1.51E-07 | 8.43E-07 |
| GATSL2 | -0.2277587 | 1.52E-07 | 8.45E-07 |
| DLEU1 | 0.2277394 | 1.52E-07 | 8.46E-07 |
| C5orf30 | 0.2277349 | 1.52E-07 | 8.47E-07 |
| WDR4 | 0.2277247 | 1.52E-07 | 8.48E-07 |
| PALM2 | -0.2276797 | 1.53E-07 | 8.52E-07 |
| RPL18 | 0.2276579 | 1.54E-07 | 8.54E-07 |
| LRRC3 | -0.2276211 | 1.55E-07 | 8.58E-07 |
| PCSK1 | -0.2276203 | 1.55E-07 | 8.58E-07 |
| MYOZ1 | -0.227614 | 1.55E-07 | 8.59E-07 |
| DUSP16 | -0.2275471 | 1.56E-07 | 8.66E-07 |
| C9orf6 | 0.2275047 | 1.57E-07 | 8.70E-07 |
| POLH | 0.2273709 | 1.60E-07 | 8.85E-07 |
| SCN4B | -0.2273499 | 1.60E-07 | 8.87E-07 |
| RNF146 | -0.2273488 | 1.60E-07 | 8.87E-07 |
| HACL1 | 0.2273169 | 1.61E-07 | 8.90E-07 |
| RNMTL1 | 0.2273056 | 1.61E-07 | 8.92E-07 |
| KLHL18 | -0.2273025 | 1.61E-07 | 8.92E-07 |
| SON | -0.2272471 | 1.62E-07 | 8.98E-07 |
| DOCK1 | -0.2272464 | 1.62E-07 | 8.98E-07 |
| COX5B | 0.2271962 | 1.63E-07 | 9.03E-07 |
| CALB2 | -0.227163 | 1.64E-07 | 9.07E-07 |
| SYNGR3 | 0.2271247 | 1.65E-07 | 9.11E-07 |
| FAM110C | -0.2271178 | 1.65E-07 | 9.12E-07 |
| C11orf87 | -0.2271003 | 1.65E-07 | 9.13E-07 |
| CHRDL2 | -0.2269879 | 1.68E-07 | 9.26E-07 |
| NCRNA00081 | 0.2269232 | 1.69E-07 | 9.34E-07 |
| SGK494 | 0.2269211 | 1.69E-07 | 9.34E-07 |
| CSRNP2 | -0.2269146 | 1.69E-07 | 9.34E-07 |
| SLC35C1 | -0.2269072 | 1.69E-07 | 9.35E-07 |
| FAM98B | 0.2268981 | 1.70E-07 | 9.36E-07 |
| PCDHAC2 | -0.226812 | 1.71E-07 | 9.46E-07 |
| ARRDC4 | -0.2267936 | 1.72E-07 | 9.48E-07 |
| MRPS6 | 0.2267582 | 1.73E-07 | 9.52E-07 |
| NCRNA00183 | 0.2267571 | 1.73E-07 | 9.52E-07 |
| TGFB2 | -0.2267355 | 1.73E-07 | 9.54E-07 |
| NENF | 0.2267245 | 1.73E-07 | 9.55E-07 |
| GSS | 0.2267073 | 1.74E-07 | 9.57E-07 |
| NOS3 | -0.2266996 | 1.74E-07 | 9.58E-07 |
| MAST4 | -0.2266963 | 1.74E-07 | 9.58E-07 |
| SERPINE1 | -0.2266854 | 1.74E-07 | 9.59E-07 |
| GOPC | -0.226658 | 1.75E-07 | 9.62E-07 |
| CCDC90A | 0.2266568 | 1.75E-07 | 9.62E-07 |
| DDI2 | -0.226652 | 1.75E-07 | 9.62E-07 |
| MYOZ3 | -0.2266238 | 1.76E-07 | 9.65E-07 |
| MFAP2 | -0.2266119 | 1.76E-07 | 9.67E-07 |
| SNAPC4 | 0.2265916 | 1.76E-07 | 9.69E-07 |
| C7orf59 | 0.2265583 | 1.77E-07 | 9.73E-07 |
| C2orf44 | 0.226461 | 1.79E-07 | 9.85E-07 |
| RERG | -0.2264286 | 1.80E-07 | 9.89E-07 |
| STX11 | -0.2263666 | 1.81E-07 | 9.96E-07 |
| DCAF12L2 | -0.2263093 | 1.83E-07 | 1.00E-06 |
| GPN1 | 0.2262947 | 1.83E-07 | 1.00E-06 |
| BEND6 | -0.2262803 | 1.83E-07 | 1.01E-06 |
| SUPT6H | -0.2262664 | 1.84E-07 | 1.01E-06 |
| OTP | 0.2262188 | 1.85E-07 | 1.01E-06 |
| C6orf204 | -0.2261849 | 1.86E-07 | 1.02E-06 |
| ETAA1 | 0.2261398 | 1.87E-07 | 1.02E-06 |
| FAM82A2 | 0.2261193 | 1.87E-07 | 1.03E-06 |
| CPA4 | -0.2260994 | 1.88E-07 | 1.03E-06 |
| ZBTB43 | -0.2260723 | 1.88E-07 | 1.03E-06 |
| SERPINH1 | -0.2260702 | 1.88E-07 | 1.03E-06 |
| ATP12A | -0.2260294 | 1.89E-07 | 1.04E-06 |
| GREB1 | -0.226024 | 1.90E-07 | 1.04E-06 |
| AGPAT5 | 0.2259072 | 1.92E-07 | 1.05E-06 |
| SLCO2A1 | -0.2258987 | 1.93E-07 | 1.05E-06 |
| S100A13 | 0.2258937 | 1.93E-07 | 1.05E-06 |
| PGLYRP3 | -0.2258796 | 1.93E-07 | 1.05E-06 |
| GPC1 | -0.225879 | 1.93E-07 | 1.05E-06 |
| MUS81 | 0.2258767 | 1.93E-07 | 1.05E-06 |
| LRFN5 | -0.2258348 | 1.94E-07 | 1.06E-06 |
| FAM59A | -0.2258306 | 1.94E-07 | 1.06E-06 |
| C11orf51 | 0.2258158 | 1.95E-07 | 1.06E-06 |
| KLHL21 | -0.2257967 | 1.95E-07 | 1.06E-06 |
| CKMT1A | 0.2257699 | 1.96E-07 | 1.07E-06 |
| MAPRE3 | -0.2257519 | 1.96E-07 | 1.07E-06 |
| MT1H | 0.2257411 | 1.97E-07 | 1.07E-06 |
| DRD5 | -0.2257362 | 1.97E-07 | 1.07E-06 |
| GAP43 | -0.225733 | 1.97E-07 | 1.07E-06 |
| STAU1 | -0.2256849 | 1.98E-07 | 1.08E-06 |
| UBE2MP1 | 0.2256802 | 1.98E-07 | 1.08E-06 |
| CBWD1 | 0.22566 | 1.99E-07 | 1.08E-06 |
| BEST3 | -0.2256432 | 1.99E-07 | 1.08E-06 |
| AKAP9 | -0.2256348 | 1.99E-07 | 1.08E-06 |
| NRP2 | -0.2255962 | 2.00E-07 | 1.09E-06 |
| CCBP2 | -0.2255864 | 2.00E-07 | 1.09E-06 |
| PMPCA | 0.2255619 | 2.01E-07 | 1.09E-06 |
| SMAD7 | -0.2254629 | 2.04E-07 | 1.11E-06 |
| STAP2 | 0.2254543 | 2.04E-07 | 1.11E-06 |
| LDB3 | -0.2254459 | 2.04E-07 | 1.11E-06 |
| EED | 0.2254196 | 2.05E-07 | 1.11E-06 |
| CTPS | 0.2253653 | 2.06E-07 | 1.12E-06 |
| DDIT4L | -0.2253642 | 2.06E-07 | 1.12E-06 |
| RANGRF | 0.2253018 | 2.08E-07 | 1.13E-06 |
| SURF6 | 0.2252998 | 2.08E-07 | 1.13E-06 |
| PWP1 | 0.2252951 | 2.08E-07 | 1.13E-06 |
| FAM110B | -0.2252706 | 2.09E-07 | 1.13E-06 |
| KPNA6 | -0.2252689 | 2.09E-07 | 1.13E-06 |
| DOLPP1 | 0.225259 | 2.09E-07 | 1.13E-06 |
| BCHE | -0.225249 | 2.09E-07 | 1.13E-06 |
| ZNF77 | 0.2252147 | 2.10E-07 | 1.14E-06 |
| PSMD6 | 0.2251853 | 2.11E-07 | 1.14E-06 |
| ARV1 | 0.2251447 | 2.12E-07 | 1.15E-06 |
| USP35 | -0.2251446 | 2.12E-07 | 1.15E-06 |
| MTA2 | 0.2251291 | 2.12E-07 | 1.15E-06 |
| FARP1 | -0.2250784 | 2.14E-07 | 1.16E-06 |
| UBA52 | 0.2250633 | 2.14E-07 | 1.16E-06 |
| UST | -0.2250556 | 2.14E-07 | 1.16E-06 |
| C19orf25 | 0.2250377 | 2.15E-07 | 1.16E-06 |
| PTPRA | -0.225034 | 2.15E-07 | 1.16E-06 |
| GNA12 | -0.2250216 | 2.15E-07 | 1.16E-06 |
| NAP1L2 | -0.2249552 | 2.17E-07 | 1.17E-06 |
| LOC285419 | -0.2249342 | 2.18E-07 | 1.18E-06 |
| DNAJC6 | -0.2249304 | 2.18E-07 | 1.18E-06 |
| ANKH | -0.2248581 | 2.20E-07 | 1.19E-06 |
| CLCA2 | -0.2248472 | 2.20E-07 | 1.19E-06 |
| CCR8 | -0.2248363 | 2.20E-07 | 1.19E-06 |
| FBXL6 | 0.2248273 | 2.21E-07 | 1.19E-06 |
| MLL2 | -0.2248106 | 2.21E-07 | 1.19E-06 |
| SPNS2 | -0.2247614 | 2.23E-07 | 1.20E-06 |
| CD207 | -0.2247061 | 2.24E-07 | 1.21E-06 |
| AP2A1 | -0.2247054 | 2.24E-07 | 1.21E-06 |
| MYOG | -0.2246773 | 2.25E-07 | 1.21E-06 |
| RPL29 | 0.2246667 | 2.25E-07 | 1.21E-06 |
| MAF | -0.2246471 | 2.26E-07 | 1.22E-06 |
| FLRT2 | -0.2246402 | 2.26E-07 | 1.22E-06 |
| BCAS3 | -0.2246166 | 2.27E-07 | 1.22E-06 |
| SPTBN1 | -0.2245739 | 2.28E-07 | 1.23E-06 |
| ETV7 | 0.2245732 | 2.28E-07 | 1.23E-06 |
| SMU1 | 0.2245481 | 2.29E-07 | 1.23E-06 |
| ACE | -0.2244937 | 2.30E-07 | 1.24E-06 |
| UIMC1 | 0.2243488 | 2.35E-07 | 1.26E-06 |
| DCTN3 | 0.2243384 | 2.35E-07 | 1.26E-06 |
| NKAIN2 | -0.2243377 | 2.35E-07 | 1.26E-06 |
| ODZ3 | -0.2242901 | 2.36E-07 | 1.27E-06 |
| GOLGA6L9 | 0.2242476 | 2.38E-07 | 1.27E-06 |
| TGFBRAP1 | -0.224232 | 2.38E-07 | 1.28E-06 |
| PEAR1 | -0.2242044 | 2.39E-07 | 1.28E-06 |
| PCMTD1 | -0.224188 | 2.39E-07 | 1.28E-06 |
| ABHD4 | -0.2241747 | 2.40E-07 | 1.28E-06 |
| GTF2H2C | 0.2241462 | 2.41E-07 | 1.29E-06 |
| PDIA3 | 0.2241431 | 2.41E-07 | 1.29E-06 |
| TRMU | 0.2241298 | 2.41E-07 | 1.29E-06 |
| PRELID1 | 0.2241238 | 2.41E-07 | 1.29E-06 |
| ZFYVE9 | -0.224113 | 2.42E-07 | 1.29E-06 |
| ASB15 | -0.2241007 | 2.42E-07 | 1.29E-06 |
| BTG3 | 0.2240844 | 2.42E-07 | 1.30E-06 |
| LZTS1 | -0.2240541 | 2.43E-07 | 1.30E-06 |
| PLA2G4F | -0.2240469 | 2.44E-07 | 1.30E-06 |
| AGPAT9 | -0.2240018 | 2.45E-07 | 1.31E-06 |
| RRP8 | 0.2239256 | 2.47E-07 | 1.32E-06 |
| OAZ1 | 0.2239073 | 2.48E-07 | 1.32E-06 |
| NME6 | 0.2239058 | 2.48E-07 | 1.32E-06 |
| LEPREL2 | -0.2238842 | 2.49E-07 | 1.33E-06 |
| ABCC1 | -0.2238532 | 2.50E-07 | 1.33E-06 |
| LOC283867 | -0.2237744 | 2.52E-07 | 1.35E-06 |
| EIF4G1 | -0.223723 | 2.54E-07 | 1.35E-06 |
| MSMP | -0.2236959 | 2.55E-07 | 1.36E-06 |
| TUBGCP5 | 0.2236877 | 2.55E-07 | 1.36E-06 |
| EGR2 | -0.2236718 | 2.55E-07 | 1.36E-06 |
| RPL3L | -0.2236672 | 2.56E-07 | 1.36E-06 |
| GPR68 | -0.2236516 | 2.56E-07 | 1.36E-06 |
| KRTCAP2 | 0.2236501 | 2.56E-07 | 1.36E-06 |
| TMEM194B | 0.2236451 | 2.56E-07 | 1.37E-06 |
| FAM73A | -0.2236386 | 2.57E-07 | 1.37E-06 |
| FZD7 | -0.223595 | 2.58E-07 | 1.37E-06 |
| LRRN1 | -0.2235872 | 2.58E-07 | 1.37E-06 |
| PDCL3 | 0.2235625 | 2.59E-07 | 1.38E-06 |
| NUCKS1 | 0.2235497 | 2.59E-07 | 1.38E-06 |
| POLB | 0.2235341 | 2.60E-07 | 1.38E-06 |
| CDK5RAP3 | 0.2235256 | 2.60E-07 | 1.38E-06 |
| ABLIM1 | -0.2234424 | 2.63E-07 | 1.40E-06 |
| TMEM130 | -0.2234289 | 2.63E-07 | 1.40E-06 |
| C1orf212 | 0.223416 | 2.64E-07 | 1.40E-06 |
| KY | -0.2233962 | 2.64E-07 | 1.40E-06 |
| CCL24 | -0.2233775 | 2.65E-07 | 1.41E-06 |
| MICALL1 | -0.2233623 | 2.66E-07 | 1.41E-06 |
| TSPAN31 | -0.2233432 | 2.66E-07 | 1.41E-06 |
| MAMDC4 | 0.2232806 | 2.68E-07 | 1.42E-06 |
| COX7A2L | 0.2232522 | 2.69E-07 | 1.43E-06 |
| TCEB1 | 0.2232143 | 2.71E-07 | 1.43E-06 |
| RASSF8 | -0.2231981 | 2.71E-07 | 1.44E-06 |
| LMO7 | -0.2231928 | 2.71E-07 | 1.44E-06 |
| ENAM | -0.2231378 | 2.73E-07 | 1.45E-06 |
| ZNF853 | -0.2231019 | 2.74E-07 | 1.45E-06 |
| LY6G5B | 0.2230583 | 2.76E-07 | 1.46E-06 |
| C1orf97 | 0.2230229 | 2.77E-07 | 1.47E-06 |
| GABRA3 | -0.2230217 | 2.77E-07 | 1.47E-06 |
| ZNF530 | 0.223017 | 2.77E-07 | 1.47E-06 |
| BSDC1 | -0.2230008 | 2.78E-07 | 1.47E-06 |
| TBX3 | -0.222976 | 2.79E-07 | 1.47E-06 |
| C5orf43 | 0.2229755 | 2.79E-07 | 1.47E-06 |
| KCNA7 | -0.2229632 | 2.79E-07 | 1.48E-06 |
| NEDD8 | 0.2229309 | 2.80E-07 | 1.48E-06 |
| OSBPL2 | -0.2229212 | 2.81E-07 | 1.48E-06 |
| SIGIRR | 0.2229131 | 2.81E-07 | 1.48E-06 |
| C5orf42 | -0.2228981 | 2.82E-07 | 1.49E-06 |
| DRG1 | 0.2228935 | 2.82E-07 | 1.49E-06 |
| PRPF39 | 0.222825 | 2.84E-07 | 1.50E-06 |
| TAGLN2 | 0.2228164 | 2.84E-07 | 1.50E-06 |
| DNAJB4 | -0.2228026 | 2.85E-07 | 1.50E-06 |
| AKR7L | 0.2227644 | 2.86E-07 | 1.51E-06 |
| EHBP1 | -0.2227601 | 2.87E-07 | 1.51E-06 |
| WWC2 | -0.2227427 | 2.87E-07 | 1.51E-06 |
| HRNR | -0.2226745 | 2.90E-07 | 1.53E-06 |
| GRN | -0.2226716 | 2.90E-07 | 1.53E-06 |
| EIF1AX | 0.2226483 | 2.91E-07 | 1.53E-06 |
| CADM2 | -0.2226219 | 2.92E-07 | 1.53E-06 |
| MYBPH | -0.2226181 | 2.92E-07 | 1.54E-06 |
| RPLP2 | 0.2226122 | 2.92E-07 | 1.54E-06 |
| NACAP1 | 0.2226093 | 2.92E-07 | 1.54E-06 |
| BAG1 | 0.2226038 | 2.92E-07 | 1.54E-06 |
| DHDH | 0.2225829 | 2.93E-07 | 1.54E-06 |
| CRIP3 | 0.2225823 | 2.93E-07 | 1.54E-06 |
| PKD2 | -0.2225348 | 2.95E-07 | 1.55E-06 |
| REEP4 | 0.2225332 | 2.95E-07 | 1.55E-06 |
| FAM49A | -0.2225112 | 2.96E-07 | 1.55E-06 |
| RPL22L1 | 0.2225039 | 2.96E-07 | 1.55E-06 |
| PHPT1 | 0.2223964 | 3.00E-07 | 1.57E-06 |
| PTPN23 | -0.2223875 | 3.00E-07 | 1.58E-06 |
| ZNF692 | 0.2223507 | 3.02E-07 | 1.58E-06 |
| ZC3H8 | 0.2223259 | 3.03E-07 | 1.59E-06 |
| TIMM50 | 0.2223103 | 3.03E-07 | 1.59E-06 |
| TMEM169 | -0.2222696 | 3.05E-07 | 1.60E-06 |
| OMA1 | 0.2222492 | 3.05E-07 | 1.60E-06 |
| CALM3 | 0.2222248 | 3.06E-07 | 1.61E-06 |
| TUSC2 | 0.2221996 | 3.07E-07 | 1.61E-06 |
| SERPINB12 | -0.2221639 | 3.09E-07 | 1.62E-06 |
| ARHGAP33 | 0.222154 | 3.09E-07 | 1.62E-06 |
| PRR19 | 0.222114 | 3.11E-07 | 1.63E-06 |
| ABCA17P | 0.2220935 | 3.11E-07 | 1.63E-06 |
| XIRP1 | -0.2220878 | 3.12E-07 | 1.63E-06 |
| CCR4 | -0.2220618 | 3.13E-07 | 1.64E-06 |
| JAKMIP2 | -0.2219821 | 3.16E-07 | 1.65E-06 |
| CYTSA | -0.221967 | 3.16E-07 | 1.65E-06 |
| IGFL1 | -0.2219613 | 3.17E-07 | 1.66E-06 |
| KCNJ8 | -0.2219332 | 3.18E-07 | 1.66E-06 |
| ODF2 | 0.2218652 | 3.21E-07 | 1.67E-06 |
| GPSM1 | -0.2218488 | 3.21E-07 | 1.68E-06 |
| KIAA1737 | -0.221836 | 3.22E-07 | 1.68E-06 |
| PPOX | 0.2217843 | 3.24E-07 | 1.69E-06 |
| DUSP10 | -0.2217593 | 3.25E-07 | 1.69E-06 |
| VSIG10L | -0.2217435 | 3.25E-07 | 1.70E-06 |
| CCIN | -0.2217374 | 3.26E-07 | 1.70E-06 |
| AFAP1 | -0.2217176 | 3.26E-07 | 1.70E-06 |
| SCN9A | -0.2217148 | 3.27E-07 | 1.70E-06 |
| UBL5 | 0.2217118 | 3.27E-07 | 1.70E-06 |
| CRHR2 | -0.2217092 | 3.27E-07 | 1.70E-06 |
| CDKN2BAS | 0.221704 | 3.27E-07 | 1.70E-06 |
| 4-Sep | -0.2217026 | 3.27E-07 | 1.70E-06 |
| PRR22 | 0.2216735 | 3.28E-07 | 1.71E-06 |
| ABTB2 | -0.2216337 | 3.30E-07 | 1.72E-06 |
| PLCD1 | -0.2216326 | 3.30E-07 | 1.72E-06 |
| BCORL1 | -0.2216281 | 3.30E-07 | 1.72E-06 |
| C17orf61 | 0.2215835 | 3.32E-07 | 1.73E-06 |
| SNHG7 | 0.2215756 | 3.32E-07 | 1.73E-06 |
| ZNF195 | 0.2215511 | 3.33E-07 | 1.73E-06 |
| DDT | 0.2215372 | 3.34E-07 | 1.74E-06 |
| PCDH7 | -0.2215192 | 3.35E-07 | 1.74E-06 |
| CBWD2 | 0.2215155 | 3.35E-07 | 1.74E-06 |
| HIST1H2BM | 0.2215007 | 3.35E-07 | 1.74E-06 |
| ZNF208 | -0.2214955 | 3.36E-07 | 1.74E-06 |
| TMBIM1 | -0.2214934 | 3.36E-07 | 1.74E-06 |
| BICD2 | -0.2214849 | 3.36E-07 | 1.74E-06 |
| KLC1 | -0.2214802 | 3.36E-07 | 1.74E-06 |
| CASS4 | -0.2214489 | 3.38E-07 | 1.75E-06 |
| RNASEH2C | 0.2214129 | 3.39E-07 | 1.76E-06 |
| GFOD2 | -0.2213979 | 3.40E-07 | 1.76E-06 |
| TUG1 | -0.2213752 | 3.41E-07 | 1.77E-06 |
| GDF7 | -0.2213584 | 3.41E-07 | 1.77E-06 |
| MCFD2 | -0.2213411 | 3.42E-07 | 1.77E-06 |
| ABCC10 | -0.2213161 | 3.43E-07 | 1.78E-06 |
| ROCK2 | -0.221293 | 3.44E-07 | 1.78E-06 |
| SLC2A11 | -0.2212693 | 3.45E-07 | 1.79E-06 |
| ZNF426 | -0.2212679 | 3.45E-07 | 1.79E-06 |
| ADSSL1 | -0.2212032 | 3.48E-07 | 1.80E-06 |
| WIBG | 0.2211991 | 3.48E-07 | 1.80E-06 |
| NRXN2 | -0.2211519 | 3.50E-07 | 1.81E-06 |
| ATAD3A | 0.2211035 | 3.52E-07 | 1.82E-06 |
| COQ6 | 0.2210953 | 3.53E-07 | 1.82E-06 |
| MBD4 | 0.2210895 | 3.53E-07 | 1.82E-06 |
| TRIM61 | -0.2210637 | 3.54E-07 | 1.83E-06 |
| WNT5B | -0.221045 | 3.55E-07 | 1.83E-06 |
| NFIX | -0.2210321 | 3.56E-07 | 1.84E-06 |
| MYLK4 | -0.2209847 | 3.58E-07 | 1.85E-06 |
| MYH7 | -0.2209605 | 3.59E-07 | 1.85E-06 |
| SYN3 | -0.2208954 | 3.62E-07 | 1.87E-06 |
| SMO | -0.2208796 | 3.62E-07 | 1.87E-06 |
| SIGLEC6 | -0.2207427 | 3.69E-07 | 1.90E-06 |
| PGAM2 | -0.2207202 | 3.70E-07 | 1.91E-06 |
| GPR137C | 0.2206274 | 3.74E-07 | 1.93E-06 |
| DOM3Z | 0.2206178 | 3.74E-07 | 1.93E-06 |
| LUZP6 | -0.2205783 | 3.76E-07 | 1.94E-06 |
| CASZ1 | -0.2205569 | 3.77E-07 | 1.94E-06 |
| ZCCHC6 | -0.2205489 | 3.78E-07 | 1.94E-06 |
| SLC6A20 | -0.2205412 | 3.78E-07 | 1.95E-06 |
| TPRG1L | -0.220541 | 3.78E-07 | 1.95E-06 |
| EDF1 | 0.2205254 | 3.79E-07 | 1.95E-06 |
| CXorf42 | 0.2205127 | 3.79E-07 | 1.95E-06 |
| TCERG1 | 0.220498 | 3.80E-07 | 1.95E-06 |
| LDLRAD3 | -0.2204659 | 3.82E-07 | 1.96E-06 |
| MYOT | -0.2204379 | 3.83E-07 | 1.97E-06 |
| RAB31 | -0.2204374 | 3.83E-07 | 1.97E-06 |
| PACSIN2 | -0.2204255 | 3.84E-07 | 1.97E-06 |
| SIGLECP3 | -0.2204051 | 3.85E-07 | 1.97E-06 |
| RASSF1 | 0.2204042 | 3.85E-07 | 1.97E-06 |
| RASSF7 | 0.2203646 | 3.86E-07 | 1.98E-06 |
| KLHL28 | -0.2203587 | 3.87E-07 | 1.98E-06 |
| KLKP1 | -0.22032 | 3.89E-07 | 1.99E-06 |
| MANBA | -0.2202891 | 3.90E-07 | 2.00E-06 |
| PTPRN | -0.2202316 | 3.93E-07 | 2.01E-06 |
| THYN1 | 0.2202178 | 3.94E-07 | 2.02E-06 |
| ZYX | -0.2202127 | 3.94E-07 | 2.02E-06 |
| GRINL1A | 0.2201462 | 3.97E-07 | 2.03E-06 |
| SHANK3 | -0.2201151 | 3.99E-07 | 2.04E-06 |
| BICD1 | -0.2200984 | 3.99E-07 | 2.05E-06 |
| APBA2 | -0.2200964 | 4.00E-07 | 2.05E-06 |
| SFXN3 | -0.2200446 | 4.02E-07 | 2.06E-06 |
| TLCD1 | 0.2200344 | 4.03E-07 | 2.06E-06 |
| SORD | 0.220029 | 4.03E-07 | 2.06E-06 |
| FAM70B | -0.2199544 | 4.07E-07 | 2.08E-06 |
| TRO | -0.2199256 | 4.08E-07 | 2.09E-06 |
| ADAL | 0.2199078 | 4.09E-07 | 2.09E-06 |
| RBL2 | -0.2198914 | 4.10E-07 | 2.09E-06 |
| CASP14 | -0.2198576 | 4.12E-07 | 2.10E-06 |
| DCAF15 | 0.2198537 | 4.12E-07 | 2.10E-06 |
| ANKRD42 | -0.219838 | 4.13E-07 | 2.11E-06 |
| TFE3 | -0.2197583 | 4.17E-07 | 2.13E-06 |
| C14orf21 | -0.2197444 | 4.17E-07 | 2.13E-06 |
| HFE2 | -0.2196983 | 4.20E-07 | 2.14E-06 |
| DECR1 | 0.2196982 | 4.20E-07 | 2.14E-06 |
| CLEC5A | -0.2196918 | 4.20E-07 | 2.14E-06 |
| KIAA0196 | -0.219672 | 4.21E-07 | 2.15E-06 |
| LIMS3-LOC440895 | -0.2195379 | 4.28E-07 | 2.18E-06 |
| SERF2 | 0.2195199 | 4.29E-07 | 2.19E-06 |
| PALMD | -0.2195134 | 4.29E-07 | 2.19E-06 |
| TERT | 0.2195072 | 4.30E-07 | 2.19E-06 |
| TM4SF19 | -0.219466 | 4.32E-07 | 2.20E-06 |
| TRIP4 | 0.219431 | 4.34E-07 | 2.21E-06 |
| HMGN4 | 0.2194215 | 4.34E-07 | 2.21E-06 |
| MCHR1 | -0.2194036 | 4.35E-07 | 2.21E-06 |
| SLC16A7 | -0.2194022 | 4.35E-07 | 2.21E-06 |
| SMARCA1 | -0.2193822 | 4.36E-07 | 2.22E-06 |
| ASB10 | -0.2193777 | 4.37E-07 | 2.22E-06 |
| C14orf73 | 0.219353 | 4.38E-07 | 2.23E-06 |
| CAMTA1 | 0.2193433 | 4.39E-07 | 2.23E-06 |
| LMOD2 | -0.2192777 | 4.42E-07 | 2.25E-06 |
| EFNA3 | -0.2192766 | 4.42E-07 | 2.25E-06 |
| C17orf65 | 0.2192514 | 4.44E-07 | 2.25E-06 |
| NCKAP5 | -0.2192344 | 4.45E-07 | 2.26E-06 |
| TMED7-TICAM2 | -0.2191668 | 4.48E-07 | 2.27E-06 |
| NPLOC4 | -0.2191667 | 4.48E-07 | 2.27E-06 |
| TNFRSF19 | -0.2191324 | 4.50E-07 | 2.28E-06 |
| MIB1 | -0.2191197 | 4.51E-07 | 2.29E-06 |
| ACTN2 | -0.2191069 | 4.52E-07 | 2.29E-06 |
| CCDC144B | -0.2190781 | 4.53E-07 | 2.30E-06 |
| RPS19BP1 | 0.2190571 | 4.54E-07 | 2.30E-06 |
| KCTD15 | -0.2190357 | 4.56E-07 | 2.31E-06 |
| UBA3 | 0.219022 | 4.56E-07 | 2.31E-06 |
| STOML1 | 0.2189945 | 4.58E-07 | 2.32E-06 |
| GAN | -0.2189922 | 4.58E-07 | 2.32E-06 |
| CST6 | -0.2189503 | 4.60E-07 | 2.33E-06 |
| GFER | 0.2189429 | 4.61E-07 | 2.33E-06 |
| NUP62 | 0.2189289 | 4.62E-07 | 2.34E-06 |
| LAMA1 | -0.2189255 | 4.62E-07 | 2.34E-06 |
| HIST2H2BE | -0.2189205 | 4.62E-07 | 2.34E-06 |
| ALKBH4 | 0.2189194 | 4.62E-07 | 2.34E-06 |
| SYF2 | 0.2189088 | 4.63E-07 | 2.34E-06 |
| CCDC76 | 0.218908 | 4.63E-07 | 2.34E-06 |
| C1QTNF3 | -0.2189062 | 4.63E-07 | 2.34E-06 |
| PDK4 | -0.2189042 | 4.63E-07 | 2.34E-06 |
| NRBF2 | -0.2188967 | 4.63E-07 | 2.34E-06 |
| UBN2 | -0.2188762 | 4.65E-07 | 2.35E-06 |
| DTNBP1 | 0.2188738 | 4.65E-07 | 2.35E-06 |
| C14orf118 | -0.2188722 | 4.65E-07 | 2.35E-06 |
| ECE2 | 0.2188682 | 4.65E-07 | 2.35E-06 |
| GCC1 | -0.2188247 | 4.68E-07 | 2.36E-06 |
| ANKRD1 | -0.2188096 | 4.68E-07 | 2.36E-06 |
| TNKS2 | -0.2187838 | 4.70E-07 | 2.37E-06 |
| CUZD1 | 0.218776 | 4.70E-07 | 2.37E-06 |
| VDAC2 | 0.218774 | 4.71E-07 | 2.37E-06 |
| FAM196B | -0.2185304 | 4.85E-07 | 2.44E-06 |
| MYB | 0.2185196 | 4.85E-07 | 2.44E-06 |
| ZNF700 | 0.2184606 | 4.89E-07 | 2.46E-06 |
| LPPR5 | -0.2184424 | 4.90E-07 | 2.47E-06 |
| MYST4 | -0.2184084 | 4.92E-07 | 2.48E-06 |
| AMH | 0.2184039 | 4.92E-07 | 2.48E-06 |
| HHATL | -0.2183957 | 4.93E-07 | 2.48E-06 |
| FUT11 | -0.2183898 | 4.93E-07 | 2.48E-06 |
| VGF | 0.2183521 | 4.96E-07 | 2.49E-06 |
| PDZRN4 | -0.2183342 | 4.97E-07 | 2.50E-06 |
| CASQ2 | -0.218274 | 5.00E-07 | 2.51E-06 |
| PIM1 | -0.2182612 | 5.01E-07 | 2.52E-06 |
| GPRIN3 | -0.218261 | 5.01E-07 | 2.52E-06 |
| TYSND1 | 0.2182524 | 5.02E-07 | 2.52E-06 |
| PLB1 | -0.218203 | 5.05E-07 | 2.53E-06 |
| MMP8 | -0.2181612 | 5.07E-07 | 2.55E-06 |
| NES | -0.2181495 | 5.08E-07 | 2.55E-06 |
| SHD | -0.2181459 | 5.08E-07 | 2.55E-06 |
| ABCB9 | 0.2181053 | 5.11E-07 | 2.56E-06 |
| APC | -0.2180792 | 5.13E-07 | 2.57E-06 |
| RAB2A | -0.2180735 | 5.13E-07 | 2.57E-06 |
| RRP15 | 0.2180375 | 5.15E-07 | 2.58E-06 |
| YIPF7 | -0.2180329 | 5.15E-07 | 2.58E-06 |
| COBL | -0.2180255 | 5.16E-07 | 2.58E-06 |
| AKT3 | -0.2180216 | 5.16E-07 | 2.58E-06 |
| ANTXR2 | -0.2179663 | 5.20E-07 | 2.60E-06 |
| ARPC5 | 0.21795 | 5.21E-07 | 2.61E-06 |
| C19orf70 | 0.2179312 | 5.22E-07 | 2.61E-06 |
| RNF40 | -0.2178958 | 5.24E-07 | 2.62E-06 |
| NFIC | -0.2178956 | 5.24E-07 | 2.62E-06 |
| XRCC4 | 0.2178739 | 5.26E-07 | 2.63E-06 |
| C3orf23 | 0.2178713 | 5.26E-07 | 2.63E-06 |
| CELSR1 | -0.2178642 | 5.26E-07 | 2.63E-06 |
| GMPS | 0.2178436 | 5.28E-07 | 2.64E-06 |
| ARHGEF2 | -0.2178222 | 5.29E-07 | 2.64E-06 |
| DGKH | -0.2177948 | 5.31E-07 | 2.65E-06 |
| FAM160A1 | -0.2177893 | 5.31E-07 | 2.65E-06 |
| CACNG1 | -0.2177019 | 5.37E-07 | 2.68E-06 |
| COX17 | 0.2176648 | 5.39E-07 | 2.69E-06 |
| REC8 | 0.2176564 | 5.40E-07 | 2.69E-06 |
| TFDP1 | 0.2176333 | 5.41E-07 | 2.70E-06 |
| RBM15 | 0.2176032 | 5.43E-07 | 2.71E-06 |
| PRKRIP1 | 0.2175855 | 5.45E-07 | 2.71E-06 |
| TPSAB1 | -0.2175835 | 5.45E-07 | 2.71E-06 |
| SURF1 | 0.2175445 | 5.47E-07 | 2.73E-06 |
| RP9 | 0.2175197 | 5.49E-07 | 2.73E-06 |
| GGH | 0.2174811 | 5.52E-07 | 2.75E-06 |
| EHBP1L1 | -0.2173924 | 5.58E-07 | 2.78E-06 |
| SPRY4 | -0.217385 | 5.58E-07 | 2.78E-06 |
| RNF32 | 0.217364 | 5.60E-07 | 2.78E-06 |
| DCXR | 0.2173574 | 5.60E-07 | 2.79E-06 |
| TPI1 | 0.2173361 | 5.61E-07 | 2.79E-06 |
| JMY | -0.217333 | 5.62E-07 | 2.79E-06 |
| EXOG | 0.2173108 | 5.63E-07 | 2.80E-06 |
| MAK16 | 0.2173064 | 5.63E-07 | 2.80E-06 |
| CYLD | -0.2172961 | 5.64E-07 | 2.80E-06 |
| KRT23 | -0.2172702 | 5.66E-07 | 2.81E-06 |
| GPKOW | 0.2172642 | 5.66E-07 | 2.81E-06 |
| FAM101B | -0.217261 | 5.67E-07 | 2.81E-06 |
| SLC25A35 | 0.2172456 | 5.68E-07 | 2.82E-06 |
| ABCC3 | -0.2172418 | 5.68E-07 | 2.82E-06 |
| APEX1 | 0.2172159 | 5.70E-07 | 2.83E-06 |
| NUP88 | 0.2171874 | 5.72E-07 | 2.84E-06 |
| PDZD4 | -0.2171779 | 5.72E-07 | 2.84E-06 |
| NDST2 | -0.2171665 | 5.73E-07 | 2.84E-06 |
| LEO1 | 0.2171334 | 5.76E-07 | 2.85E-06 |
| MKL2 | -0.217086 | 5.79E-07 | 2.87E-06 |
| TIRAP | -0.2170858 | 5.79E-07 | 2.87E-06 |
| CNOT7 | 0.2170716 | 5.80E-07 | 2.87E-06 |
| ITIH5 | -0.2169391 | 5.89E-07 | 2.92E-06 |
| SLC25A41 | 0.2169359 | 5.90E-07 | 2.92E-06 |
| KIAA0100 | -0.2169356 | 5.90E-07 | 2.92E-06 |
| HILS1 | -0.216921 | 5.91E-07 | 2.92E-06 |
| AIMP2 | 0.2168897 | 5.93E-07 | 2.93E-06 |
| BNIPL | -0.2168849 | 5.93E-07 | 2.93E-06 |
| RB1CC1 | -0.2168819 | 5.93E-07 | 2.93E-06 |
| CLIC1 | 0.2168737 | 5.94E-07 | 2.94E-06 |
| SPTY2D1 | -0.2168626 | 5.95E-07 | 2.94E-06 |
| HERC5 | 0.2168316 | 5.97E-07 | 2.95E-06 |
| MEGF9 | -0.2168007 | 5.99E-07 | 2.96E-06 |
| ST8SIA5 | -0.2167906 | 6.00E-07 | 2.96E-06 |
| TRAK2 | -0.2167709 | 6.02E-07 | 2.97E-06 |
| ANKS1A | -0.2167491 | 6.03E-07 | 2.98E-06 |
| GPRASP1 | -0.2167306 | 6.05E-07 | 2.98E-06 |
| SENP8 | 0.216721 | 6.05E-07 | 2.99E-06 |
| ARHGEF18 | -0.2167015 | 6.07E-07 | 2.99E-06 |
| ZDHHC20 | -0.2166813 | 6.08E-07 | 3.00E-06 |
| RNF216 | -0.2165421 | 6.19E-07 | 3.05E-06 |
| TMEM42 | 0.2165241 | 6.20E-07 | 3.06E-06 |
| TRAM1L1 | -0.2163915 | 6.30E-07 | 3.10E-06 |
| CXorf64 | -0.2163911 | 6.30E-07 | 3.10E-06 |
| SMCR8 | -0.2163785 | 6.31E-07 | 3.11E-06 |
| NR1I3 | 0.216365 | 6.32E-07 | 3.11E-06 |
| RAB3GAP1 | -0.2163563 | 6.33E-07 | 3.11E-06 |
| SORL1 | -0.2163279 | 6.35E-07 | 3.12E-06 |
| MAFG | -0.2163249 | 6.35E-07 | 3.12E-06 |
| STK32A | -0.2162931 | 6.38E-07 | 3.14E-06 |
| LOC441208 | -0.2162898 | 6.38E-07 | 3.14E-06 |
| DUSP14 | -0.2162408 | 6.42E-07 | 3.15E-06 |
| CPN2 | -0.216221 | 6.43E-07 | 3.16E-06 |
| ZNF333 | -0.2162193 | 6.43E-07 | 3.16E-06 |
| PPIL3 | 0.2162151 | 6.44E-07 | 3.16E-06 |
| ASB5 | -0.2162031 | 6.45E-07 | 3.17E-06 |
| CLEC14A | -0.2161406 | 6.50E-07 | 3.19E-06 |
| HIF1A | -0.2160888 | 6.54E-07 | 3.21E-06 |
| FBRS | -0.2160214 | 6.59E-07 | 3.23E-06 |
| GPSM2 | 0.2159971 | 6.61E-07 | 3.24E-06 |
| DEM1 | 0.2159622 | 6.64E-07 | 3.26E-06 |
| HOXC13 | -0.2159442 | 6.65E-07 | 3.26E-06 |
| ZC4H2 | -0.2159439 | 6.65E-07 | 3.26E-06 |
| ZNF827 | -0.2159329 | 6.66E-07 | 3.27E-06 |
| ZSCAN23 | -0.215921 | 6.67E-07 | 3.27E-06 |
| CSNK2B | 0.2158936 | 6.69E-07 | 3.28E-06 |
| KHDC1 | 0.215893 | 6.70E-07 | 3.28E-06 |
| ACTC1 | -0.2158733 | 6.71E-07 | 3.29E-06 |
| PRDM11 | -0.2158676 | 6.72E-07 | 3.29E-06 |
| SGTA | 0.215859 | 6.72E-07 | 3.29E-06 |
| FGF14 | -0.2158516 | 6.73E-07 | 3.29E-06 |
| RNF113A | 0.2158335 | 6.74E-07 | 3.30E-06 |
| FHL5 | -0.2157953 | 6.78E-07 | 3.31E-06 |
| UBA2 | 0.2157425 | 6.82E-07 | 3.33E-06 |
| THRB | -0.2156603 | 6.89E-07 | 3.37E-06 |
| ROMO1 | 0.2156332 | 6.91E-07 | 3.38E-06 |
| ALG14 | 0.2155848 | 6.95E-07 | 3.40E-06 |
| CABLES2 | 0.215576 | 6.96E-07 | 3.40E-06 |
| CLK3 | 0.215564 | 6.97E-07 | 3.40E-06 |
| RPS12 | 0.2155601 | 6.97E-07 | 3.40E-06 |
| COX19 | 0.2155517 | 6.98E-07 | 3.41E-06 |
| SFT2D1 | 0.2155048 | 7.02E-07 | 3.43E-06 |
| NFKB2 | 0.2154635 | 7.05E-07 | 3.44E-06 |
| HYOU1 | -0.2154611 | 7.06E-07 | 3.44E-06 |
| FASTKD1 | 0.2154312 | 7.08E-07 | 3.45E-06 |
| MT1G | 0.2153685 | 7.14E-07 | 3.48E-06 |
| MTSS1 | -0.2153087 | 7.19E-07 | 3.50E-06 |
| FOXE3 | 0.2152762 | 7.22E-07 | 3.52E-06 |
| TMEM11 | 0.2152683 | 7.22E-07 | 3.52E-06 |
| PCBP2 | 0.2152618 | 7.23E-07 | 3.52E-06 |
| MYLPF | -0.2152434 | 7.24E-07 | 3.53E-06 |
| MAFK | -0.2152407 | 7.25E-07 | 3.53E-06 |
| WNT9A | -0.215237 | 7.25E-07 | 3.53E-06 |
| ZNF323 | -0.2152045 | 7.28E-07 | 3.54E-06 |
| LOC339524 | -0.2151878 | 7.29E-07 | 3.55E-06 |
| ZFHX4 | -0.215163 | 7.32E-07 | 3.56E-06 |
| ZAR1 | 0.215158 | 7.32E-07 | 3.56E-06 |
| HSN2 | -0.2151558 | 7.32E-07 | 3.56E-06 |
| MSC | -0.2151523 | 7.33E-07 | 3.56E-06 |
| FBXL20 | -0.2151287 | 7.35E-07 | 3.57E-06 |
| RCAN1 | -0.2151183 | 7.36E-07 | 3.57E-06 |
| GTPBP3 | 0.2151172 | 7.36E-07 | 3.57E-06 |
| WASF3 | -0.2151171 | 7.36E-07 | 3.57E-06 |
| PDE1C | -0.215116 | 7.36E-07 | 3.57E-06 |
| MAFA | -0.2150899 | 7.38E-07 | 3.58E-06 |
| RNF10 | -0.2150686 | 7.40E-07 | 3.59E-06 |
| PAIP2 | 0.2150045 | 7.46E-07 | 3.62E-06 |
| TSPYL1 | -0.2149827 | 7.48E-07 | 3.63E-06 |
| CYP39A1 | -0.2149773 | 7.48E-07 | 3.63E-06 |
| TBC1D7 | 0.2149702 | 7.49E-07 | 3.63E-06 |
| UBE2QL1 | -0.2149637 | 7.49E-07 | 3.63E-06 |
| SLC45A3 | -0.214953 | 7.50E-07 | 3.64E-06 |
| TRPC6 | -0.214951 | 7.51E-07 | 3.64E-06 |
| NDUFB9 | 0.2149466 | 7.51E-07 | 3.64E-06 |
| RAD23A | 0.2149062 | 7.55E-07 | 3.65E-06 |
| FBLN7 | -0.214863 | 7.59E-07 | 3.67E-06 |
| KBTBD10 | -0.2148449 | 7.60E-07 | 3.68E-06 |
| VGLL4 | -0.2148378 | 7.61E-07 | 3.68E-06 |
| NOXO1 | 0.2148131 | 7.63E-07 | 3.69E-06 |
| ATP6V0D2 | -0.214791 | 7.65E-07 | 3.70E-06 |
| GALNT6 | -0.214771 | 7.67E-07 | 3.71E-06 |
| TNFAIP6 | -0.2147121 | 7.73E-07 | 3.73E-06 |
| ZNF582 | -0.2147104 | 7.73E-07 | 3.73E-06 |
| TRIM29 | -0.2146634 | 7.77E-07 | 3.75E-06 |
| C5orf13 | -0.2146125 | 7.82E-07 | 3.78E-06 |
| KCTD11 | -0.2145435 | 7.89E-07 | 3.81E-06 |
| LOC100128788 | 0.2145147 | 7.91E-07 | 3.82E-06 |
| ANKRD34B | 0.2144447 | 7.98E-07 | 3.85E-06 |
| C9orf7 | -0.2144436 | 7.98E-07 | 3.85E-06 |
| C6orf145 | -0.2143461 | 8.08E-07 | 3.90E-06 |
| RAD51L1 | 0.2143376 | 8.08E-07 | 3.90E-06 |
| C7orf30 | 0.2143324 | 8.09E-07 | 3.90E-06 |
| CREB5 | -0.2142951 | 8.13E-07 | 3.92E-06 |
| TSR2 | 0.2142843 | 8.14E-07 | 3.92E-06 |
| NDUFA3 | 0.2142806 | 8.14E-07 | 3.92E-06 |
| ZNF880 | -0.21421 | 8.21E-07 | 3.95E-06 |
| SEPP1 | -0.2141845 | 8.23E-07 | 3.97E-06 |
| FAM82B | 0.2141642 | 8.25E-07 | 3.97E-06 |
| HSPA9 | 0.2141481 | 8.27E-07 | 3.98E-06 |
| AVL9 | -0.2139844 | 8.44E-07 | 4.06E-06 |
| STX10 | 0.2139482 | 8.47E-07 | 4.08E-06 |
| TUBD1 | 0.2139477 | 8.47E-07 | 4.08E-06 |
| KCNA10 | -0.2139381 | 8.48E-07 | 4.08E-06 |
| BACH1 | -0.2139161 | 8.51E-07 | 4.09E-06 |
| PLEKHN1 | -0.2139083 | 8.51E-07 | 4.09E-06 |
| C6orf142 | -0.2138991 | 8.52E-07 | 4.10E-06 |
| ZIC2 | 0.2138082 | 8.62E-07 | 4.14E-06 |
| NUDT3 | 0.2138044 | 8.62E-07 | 4.14E-06 |
| GSTCD | 0.2137437 | 8.68E-07 | 4.17E-06 |
| LMOD3 | -0.2137354 | 8.69E-07 | 4.17E-06 |
| GPR153 | -0.2137143 | 8.71E-07 | 4.18E-06 |
| CORO2B | -0.2135879 | 8.85E-07 | 4.25E-06 |
| ACTA1 | -0.2135718 | 8.87E-07 | 4.25E-06 |
| SMARCD3 | -0.2135535 | 8.88E-07 | 4.26E-06 |
| PROSC | 0.213553 | 8.89E-07 | 4.26E-06 |
| GAD2 | 0.2134879 | 8.96E-07 | 4.29E-06 |
| COL16A1 | -0.2134825 | 8.96E-07 | 4.29E-06 |
| LIMS3 | -0.2134745 | 8.97E-07 | 4.30E-06 |
| SNRPB2 | 0.2134553 | 8.99E-07 | 4.31E-06 |
| PARD3B | -0.2134279 | 9.02E-07 | 4.32E-06 |
| RASA2 | -0.2134202 | 9.03E-07 | 4.32E-06 |
| TBC1D9 | -0.2133979 | 9.05E-07 | 4.33E-06 |
| LOC649330 | 0.2133604 | 9.09E-07 | 4.35E-06 |
| PDE1B | -0.2132672 | 9.20E-07 | 4.40E-06 |
| REXO4 | 0.2132254 | 9.24E-07 | 4.42E-06 |
| RGS6 | -0.2132124 | 9.26E-07 | 4.43E-06 |
| ANKRD19 | 0.2131188 | 9.36E-07 | 4.48E-06 |
| C20orf199 | 0.2131136 | 9.37E-07 | 4.48E-06 |
| FAM173A | 0.2131015 | 9.38E-07 | 4.48E-06 |
| CMTM4 | -0.2130406 | 9.45E-07 | 4.52E-06 |
| TMEM177 | 0.2130264 | 9.47E-07 | 4.52E-06 |
| MMP17 | -0.2130022 | 9.49E-07 | 4.53E-06 |
| CLTA | 0.2129818 | 9.52E-07 | 4.54E-06 |
| ABLIM2 | -0.2129802 | 9.52E-07 | 4.54E-06 |
| NDUFB4 | 0.2129399 | 9.56E-07 | 4.56E-06 |
| ENDOG | 0.2129333 | 9.57E-07 | 4.57E-06 |
| AFF1 | -0.2129144 | 9.59E-07 | 4.58E-06 |
| DPCD | 0.2128784 | 9.63E-07 | 4.59E-06 |
| PTGFRN | -0.2128313 | 9.69E-07 | 4.62E-06 |
| COQ7 | 0.212802 | 9.72E-07 | 4.64E-06 |
| MAGEE2 | -0.212797 | 9.73E-07 | 4.64E-06 |
| CDC42BPG | -0.2127312 | 9.81E-07 | 4.67E-06 |
| SLC25A37 | 0.2127021 | 9.84E-07 | 4.69E-06 |
| TSN | 0.2126832 | 9.86E-07 | 4.70E-06 |
| PSMB1 | 0.2126719 | 9.88E-07 | 4.70E-06 |
| ROBLD3 | 0.2126709 | 9.88E-07 | 4.70E-06 |
| C2CD4D | 0.2126682 | 9.88E-07 | 4.70E-06 |
| TMEM86A | -0.212653 | 9.90E-07 | 4.71E-06 |
| CCL20 | 0.2126397 | 9.91E-07 | 4.72E-06 |
| VIM | -0.2125757 | 9.99E-07 | 4.75E-06 |
| PVRL1 | -0.2125661 | 1.00E-06 | 4.76E-06 |
| WDR18 | 0.2125585 | 1.00E-06 | 4.76E-06 |
| KIAA1875 | 0.2125581 | 1.00E-06 | 4.76E-06 |
| MTIF2 | 0.2125557 | 1.00E-06 | 4.76E-06 |
| BTF3L4 | 0.2124765 | 1.01E-06 | 4.80E-06 |
| PBX4 | 0.2124556 | 1.01E-06 | 4.81E-06 |
| IDH3B | 0.2123933 | 1.02E-06 | 4.85E-06 |
| FAM198A | -0.2123667 | 1.02E-06 | 4.86E-06 |
| SNORA76 | 0.2123666 | 1.02E-06 | 4.86E-06 |
| SAMD9 | -0.2123226 | 1.03E-06 | 4.89E-06 |
| ILDR2 | -0.2122988 | 1.03E-06 | 4.90E-06 |
| TRIM72 | -0.2122743 | 1.04E-06 | 4.91E-06 |
| PILRB | 0.2122593 | 1.04E-06 | 4.92E-06 |
| ZNF467 | -0.2122384 | 1.04E-06 | 4.93E-06 |
| MTMR15 | 0.2121814 | 1.05E-06 | 4.96E-06 |
| EN1 | -0.2121559 | 1.05E-06 | 4.98E-06 |
| DMAP1 | 0.2121541 | 1.05E-06 | 4.98E-06 |
| LOC400940 | -0.2121521 | 1.05E-06 | 4.98E-06 |
| RPL10 | 0.2120247 | 1.07E-06 | 5.05E-06 |
| DNAH3 | -0.2119924 | 1.07E-06 | 5.07E-06 |
| INPP5A | -0.2119879 | 1.07E-06 | 5.07E-06 |
| CTNNA3 | -0.211921 | 1.08E-06 | 5.11E-06 |
| DSC2 | -0.2119061 | 1.08E-06 | 5.12E-06 |
| MTUS2 | -0.2118893 | 1.08E-06 | 5.13E-06 |
| COQ10B | -0.2118328 | 1.09E-06 | 5.16E-06 |
| MLLT4 | -0.2118092 | 1.09E-06 | 5.18E-06 |
| PMP2 | -0.2117994 | 1.10E-06 | 5.18E-06 |
| TMEM141 | 0.2117632 | 1.10E-06 | 5.20E-06 |
| CSF1R | -0.2117631 | 1.10E-06 | 5.20E-06 |
| HRSP12 | 0.2117604 | 1.10E-06 | 5.20E-06 |
| LCE1A | -0.2116383 | 1.12E-06 | 5.28E-06 |
| RGS10 | 0.2116238 | 1.12E-06 | 5.28E-06 |
| ZNF304 | -0.2115874 | 1.12E-06 | 5.30E-06 |
| MED18 | 0.2115863 | 1.12E-06 | 5.30E-06 |
| MAP2K5 | 0.2115832 | 1.12E-06 | 5.31E-06 |
| HES2 | -0.2115164 | 1.13E-06 | 5.35E-06 |
| SPO11 | 0.2114779 | 1.14E-06 | 5.37E-06 |
| NDUFV1 | 0.2114489 | 1.14E-06 | 5.39E-06 |
| TBRG1 | -0.2114238 | 1.15E-06 | 5.40E-06 |
| IGSF1 | -0.2114216 | 1.15E-06 | 5.40E-06 |
| KIAA1530 | -0.2114115 | 1.15E-06 | 5.41E-06 |
| RILPL1 | -0.2113648 | 1.15E-06 | 5.44E-06 |
| HERPUD1 | -0.2113236 | 1.16E-06 | 5.46E-06 |
| FLJ42289 | 0.2113178 | 1.16E-06 | 5.46E-06 |
| C12orf76 | 0.2112976 | 1.16E-06 | 5.48E-06 |
| STX3 | -0.2112948 | 1.16E-06 | 5.48E-06 |
| FBXO36 | -0.2112762 | 1.17E-06 | 5.49E-06 |
| U2AF2 | 0.2112497 | 1.17E-06 | 5.50E-06 |
| NHSL1 | -0.2112412 | 1.17E-06 | 5.51E-06 |
| SNTB2 | -0.2112128 | 1.18E-06 | 5.52E-06 |
| ACTG2 | -0.2111696 | 1.18E-06 | 5.55E-06 |
| C16orf55 | 0.2111338 | 1.19E-06 | 5.57E-06 |
| NCKIPSD | 0.2110683 | 1.20E-06 | 5.62E-06 |
| C11orf17 | 0.2109883 | 1.21E-06 | 5.67E-06 |
| TBRG4 | 0.2109805 | 1.21E-06 | 5.67E-06 |
| TMEM48 | 0.2109631 | 1.21E-06 | 5.68E-06 |
| CHGB | -0.2109533 | 1.21E-06 | 5.69E-06 |
| NDUFA13 | 0.210906 | 1.22E-06 | 5.72E-06 |
| MIIP | 0.2108956 | 1.22E-06 | 5.73E-06 |
| TMCO3 | -0.2108534 | 1.23E-06 | 5.75E-06 |
| TSEN15 | 0.2108434 | 1.23E-06 | 5.76E-06 |
| SLC27A5 | 0.2108367 | 1.23E-06 | 5.76E-06 |
| PPP1CA | 0.210836 | 1.23E-06 | 5.76E-06 |
| UBE2V2 | 0.2108174 | 1.23E-06 | 5.77E-06 |
| LOC442454 | 0.2108067 | 1.23E-06 | 5.78E-06 |
| P2RX7 | -0.2107916 | 1.24E-06 | 5.79E-06 |
| SBK2 | -0.210743 | 1.24E-06 | 5.82E-06 |
| RPAP3 | 0.2106758 | 1.25E-06 | 5.86E-06 |
| EPAS1 | -0.2105896 | 1.27E-06 | 5.92E-06 |
| GEMIN4 | 0.2105729 | 1.27E-06 | 5.93E-06 |
| IGF1 | -0.2105582 | 1.27E-06 | 5.94E-06 |
| SNX18 | -0.21054 | 1.27E-06 | 5.95E-06 |
| SPRR2B | -0.2105258 | 1.28E-06 | 5.96E-06 |
| LCE2C | -0.2105031 | 1.28E-06 | 5.98E-06 |
| FUNDC2 | 0.210498 | 1.28E-06 | 5.98E-06 |
| ETS1 | -0.2104674 | 1.28E-06 | 6.00E-06 |
| OTUB2 | -0.2104144 | 1.29E-06 | 6.04E-06 |
| DMWD | -0.2103971 | 1.29E-06 | 6.05E-06 |
| SPRR4 | -0.2103649 | 1.30E-06 | 6.07E-06 |
| LMBRD1 | -0.2103 | 1.31E-06 | 6.11E-06 |
| 6-Mar | -0.2102931 | 1.31E-06 | 6.12E-06 |
| NKAP | 0.2102922 | 1.31E-06 | 6.12E-06 |
| MLLT11 | -0.2102676 | 1.31E-06 | 6.13E-06 |
| DRD2 | -0.2102322 | 1.32E-06 | 6.16E-06 |
| RHOXF1 | 0.2102259 | 1.32E-06 | 6.16E-06 |
| PIK3CA | -0.2102128 | 1.32E-06 | 6.17E-06 |
| ADAM17 | -0.2101991 | 1.33E-06 | 6.18E-06 |
| BCL6 | -0.2101785 | 1.33E-06 | 6.19E-06 |
| CAMK1D | -0.2101605 | 1.33E-06 | 6.20E-06 |
| NCRNA00158 | 0.2101177 | 1.34E-06 | 6.23E-06 |
| CCDC15 | 0.2100796 | 1.34E-06 | 6.26E-06 |
| AFMID | 0.210054 | 1.35E-06 | 6.28E-06 |
| MRPL3 | 0.2100113 | 1.36E-06 | 6.31E-06 |
| GLS2 | 0.2099357 | 1.37E-06 | 6.36E-06 |
| MED17 | -0.209902 | 1.37E-06 | 6.39E-06 |
| MEGF6 | -0.2098636 | 1.38E-06 | 6.41E-06 |
| ACTR5 | 0.2098462 | 1.38E-06 | 6.43E-06 |
| TMPRSS13 | -0.2098082 | 1.39E-06 | 6.45E-06 |
| TUFM | 0.2097319 | 1.40E-06 | 6.51E-06 |
| PIGX | 0.2097296 | 1.40E-06 | 6.51E-06 |
| RAG1 | -0.2097207 | 1.40E-06 | 6.52E-06 |
| ACTN4 | -0.2097195 | 1.40E-06 | 6.52E-06 |
| VDR | -0.2097164 | 1.40E-06 | 6.52E-06 |
| UBN1 | -0.2096802 | 1.41E-06 | 6.54E-06 |
| KCNK2 | -0.209635 | 1.42E-06 | 6.58E-06 |
| LEF1 | -0.209611 | 1.42E-06 | 6.59E-06 |
| FOXD4L1 | 0.2096019 | 1.42E-06 | 6.60E-06 |
| GOSR2 | -0.2095987 | 1.42E-06 | 6.60E-06 |
| CIDEA | -0.2095417 | 1.43E-06 | 6.64E-06 |
| RTN2 | -0.2095317 | 1.43E-06 | 6.65E-06 |
| C13orf31 | -0.2094755 | 1.44E-06 | 6.69E-06 |
| LOC150381 | 0.2094621 | 1.45E-06 | 6.70E-06 |
| LRRC55 | -0.2094345 | 1.45E-06 | 6.72E-06 |
| TTC9B | 0.2094095 | 1.46E-06 | 6.74E-06 |
| NOC3L | 0.2093803 | 1.46E-06 | 6.76E-06 |
| TMEM26 | -0.2093217 | 1.47E-06 | 6.80E-06 |
| L2HGDH | 0.2093214 | 1.47E-06 | 6.80E-06 |
| RHEB | 0.2093106 | 1.47E-06 | 6.81E-06 |
| SLC25A33 | 0.2092962 | 1.47E-06 | 6.82E-06 |
| PTBP2 | 0.2092574 | 1.48E-06 | 6.85E-06 |
| DLX1 | -0.2092326 | 1.49E-06 | 6.87E-06 |
| ITGAE | 0.2092185 | 1.49E-06 | 6.88E-06 |
| RG9MTD3 | 0.2092108 | 1.49E-06 | 6.88E-06 |
| CKM | -0.2092051 | 1.49E-06 | 6.89E-06 |
| AATK | -0.2091915 | 1.49E-06 | 6.90E-06 |
| FANCE | 0.2091713 | 1.50E-06 | 6.91E-06 |
| SEC14L2 | -0.2091553 | 1.50E-06 | 6.92E-06 |
| FAM119A | 0.2091071 | 1.51E-06 | 6.96E-06 |
| CD209 | -0.2091012 | 1.51E-06 | 6.96E-06 |
| CLOCK | -0.2090782 | 1.51E-06 | 6.98E-06 |
| BBS2 | -0.2090682 | 1.51E-06 | 6.99E-06 |
| MYL2 | -0.2090548 | 1.52E-06 | 7.00E-06 |
| QKI | -0.2090304 | 1.52E-06 | 7.01E-06 |
| CHD8 | -0.2090301 | 1.52E-06 | 7.01E-06 |
| SPATA5 | 0.2089997 | 1.53E-06 | 7.04E-06 |
| NNMT | -0.2089129 | 1.54E-06 | 7.11E-06 |
| C9orf43 | 0.2089037 | 1.54E-06 | 7.12E-06 |
| PHKG1 | -0.2088863 | 1.55E-06 | 7.13E-06 |
| KLHL24 | -0.2088288 | 1.56E-06 | 7.17E-06 |
| ZBTB7A | -0.2087815 | 1.57E-06 | 7.21E-06 |
| CD302 | -0.2087373 | 1.57E-06 | 7.25E-06 |
| YAP1 | -0.2087239 | 1.58E-06 | 7.26E-06 |
| KBTBD11 | -0.2086838 | 1.58E-06 | 7.29E-06 |
| ANKRD23 | -0.2086794 | 1.59E-06 | 7.29E-06 |
| SPEN | -0.2086413 | 1.59E-06 | 7.32E-06 |
| C12orf53 | -0.2085979 | 1.60E-06 | 7.36E-06 |
| EEA1 | -0.2085295 | 1.61E-06 | 7.42E-06 |
| PTRF | -0.2085221 | 1.62E-06 | 7.42E-06 |
| ZYG11A | 0.2085109 | 1.62E-06 | 7.43E-06 |
| IMPG2 | -0.2084968 | 1.62E-06 | 7.44E-06 |
| CADPS | -0.208487 | 1.62E-06 | 7.45E-06 |
| SLC39A13 | -0.2084738 | 1.62E-06 | 7.46E-06 |
| XIRP2 | -0.2084398 | 1.63E-06 | 7.49E-06 |
| B3GALT1 | -0.2083475 | 1.65E-06 | 7.57E-06 |
| MED4 | 0.2083116 | 1.66E-06 | 7.60E-06 |
| ABL1 | -0.2082868 | 1.66E-06 | 7.62E-06 |
| LMCD1 | -0.2082797 | 1.66E-06 | 7.62E-06 |
| PTPLB | -0.2082781 | 1.66E-06 | 7.62E-06 |
| LOC100130331 | -0.2082752 | 1.66E-06 | 7.62E-06 |
| NDUFAF4 | 0.2082582 | 1.67E-06 | 7.64E-06 |
| HERPUD2 | -0.2082554 | 1.67E-06 | 7.64E-06 |
| NDN | -0.208224 | 1.67E-06 | 7.66E-06 |
| CARNS1 | -0.2082064 | 1.68E-06 | 7.68E-06 |
| LPCAT2 | -0.2081401 | 1.69E-06 | 7.73E-06 |
| RBM39 | 0.2080636 | 1.70E-06 | 7.80E-06 |
| SLC22A17 | -0.2080607 | 1.71E-06 | 7.80E-06 |
| TTC22 | -0.208047 | 1.71E-06 | 7.81E-06 |
| TTC27 | 0.208037 | 1.71E-06 | 7.82E-06 |
| GHR | -0.2080198 | 1.71E-06 | 7.84E-06 |
| ARPC5L | 0.2079831 | 1.72E-06 | 7.87E-06 |
| TMEM100 | -0.2079725 | 1.72E-06 | 7.88E-06 |
| FLJ90757 | -0.2079622 | 1.72E-06 | 7.88E-06 |
| LOC728264 | -0.2078674 | 1.74E-06 | 7.97E-06 |
| SYT7 | -0.2078474 | 1.75E-06 | 7.99E-06 |
| NRAP | -0.2078122 | 1.76E-06 | 8.02E-06 |
| GTF2I | -0.2077913 | 1.76E-06 | 8.03E-06 |
| CDK14 | -0.2077879 | 1.76E-06 | 8.03E-06 |
| SCARF1 | -0.2077876 | 1.76E-06 | 8.03E-06 |
| KRT6C | -0.2077417 | 1.77E-06 | 8.08E-06 |
| PPAN | 0.2077132 | 1.78E-06 | 8.10E-06 |
| MEIG1 | 0.2076883 | 1.78E-06 | 8.12E-06 |
| KIAA0562 | -0.2076742 | 1.78E-06 | 8.13E-06 |
| EEFSEC | 0.2076739 | 1.78E-06 | 8.13E-06 |
| DPM2 | 0.2076613 | 1.79E-06 | 8.14E-06 |
| LCE2B | -0.2076553 | 1.79E-06 | 8.15E-06 |
| KRT6B | -0.2075972 | 1.80E-06 | 8.20E-06 |
| MYH2 | -0.2075741 | 1.80E-06 | 8.22E-06 |
| LLPH | 0.2075667 | 1.81E-06 | 8.23E-06 |
| TTC7B | -0.20752 | 1.82E-06 | 8.27E-06 |
| ORC3L | 0.2074545 | 1.83E-06 | 8.33E-06 |
| LMAN2 | 0.2074516 | 1.83E-06 | 8.33E-06 |
| AEN | 0.2074306 | 1.84E-06 | 8.35E-06 |
| BAALC | -0.207415 | 1.84E-06 | 8.36E-06 |
| MESP1 | 0.2074031 | 1.84E-06 | 8.37E-06 |
| DHPS | 0.2073512 | 1.85E-06 | 8.42E-06 |
| ERG | -0.2073475 | 1.85E-06 | 8.42E-06 |
| RANBP10 | -0.2072558 | 1.87E-06 | 8.51E-06 |
| DIS3L | 0.2072253 | 1.88E-06 | 8.54E-06 |
| DYSFIP1 | -0.2072182 | 1.88E-06 | 8.55E-06 |
| TATDN1 | 0.2072119 | 1.88E-06 | 8.55E-06 |
| LEPRE1 | -0.2072037 | 1.88E-06 | 8.56E-06 |
| CCDC24 | 0.2071971 | 1.89E-06 | 8.56E-06 |
| MYH3 | -0.2071661 | 1.89E-06 | 8.59E-06 |
| MYF6 | -0.2071231 | 1.90E-06 | 8.63E-06 |
| RRP7B | 0.2070737 | 1.91E-06 | 8.68E-06 |
| KLK11 | -0.2070688 | 1.91E-06 | 8.68E-06 |
| OTUD7B | -0.207065 | 1.92E-06 | 8.68E-06 |
| KANK2 | -0.2070646 | 1.92E-06 | 8.68E-06 |
| ELOF1 | 0.2070451 | 1.92E-06 | 8.70E-06 |
| MAVS | -0.2070249 | 1.92E-06 | 8.72E-06 |
| CSPG5 | 0.207002 | 1.93E-06 | 8.74E-06 |
| PFKFB2 | -0.2069912 | 1.93E-06 | 8.75E-06 |
| AARSD1 | 0.2069821 | 1.93E-06 | 8.76E-06 |
| C18orf10 | 0.2069477 | 1.94E-06 | 8.79E-06 |
| LACTB2 | 0.2069476 | 1.94E-06 | 8.79E-06 |
| TCP1 | 0.2069368 | 1.94E-06 | 8.80E-06 |
| IGFBP7 | -0.2068986 | 1.95E-06 | 8.84E-06 |
| ZNF844 | -0.2068926 | 1.95E-06 | 8.84E-06 |
| TNNT2 | -0.2068808 | 1.96E-06 | 8.85E-06 |
| RLN1 | 0.2068754 | 1.96E-06 | 8.85E-06 |
| FAM96B | 0.2068024 | 1.97E-06 | 8.93E-06 |
| ZNF585B | -0.2067911 | 1.98E-06 | 8.94E-06 |
| TMSB10 | 0.2067866 | 1.98E-06 | 8.94E-06 |
| WDR89 | 0.2067849 | 1.98E-06 | 8.94E-06 |
| PTBP1 | 0.2067734 | 1.98E-06 | 8.95E-06 |
| LPAR5 | -0.2067316 | 1.99E-06 | 8.99E-06 |
| MDM1 | 0.2067291 | 1.99E-06 | 8.99E-06 |
| ALOX5 | -0.2067142 | 2.00E-06 | 9.00E-06 |
| TMEM79 | -0.206706 | 2.00E-06 | 9.01E-06 |
| ZMYND12 | -0.206702 | 2.00E-06 | 9.01E-06 |
| USP12 | -0.2066718 | 2.01E-06 | 9.04E-06 |
| TTC1 | 0.2066637 | 2.01E-06 | 9.05E-06 |
| MTCH2 | 0.2066622 | 2.01E-06 | 9.05E-06 |
| PCDHB17 | -0.2065998 | 2.02E-06 | 9.11E-06 |
| APLNR | -0.2065853 | 2.03E-06 | 9.13E-06 |
| SLTM | 0.2064484 | 2.06E-06 | 9.27E-06 |
| KIF5A | -0.2064376 | 2.06E-06 | 9.28E-06 |
| SLC46A3 | -0.2064023 | 2.07E-06 | 9.32E-06 |
| RTTN | 0.2063824 | 2.07E-06 | 9.33E-06 |
| IFT20 | 0.2063814 | 2.07E-06 | 9.33E-06 |
| FOXRED2 | 0.2063696 | 2.08E-06 | 9.35E-06 |
| HIST1H3A | 0.2063657 | 2.08E-06 | 9.35E-06 |
| GMDS | 0.2063571 | 2.08E-06 | 9.35E-06 |
| EMP1 | -0.2063522 | 2.08E-06 | 9.36E-06 |
| KIAA1841 | 0.2063287 | 2.09E-06 | 9.38E-06 |
| PDHB | 0.2062834 | 2.10E-06 | 9.43E-06 |
| GPIHBP1 | -0.2062811 | 2.10E-06 | 9.43E-06 |
| ATP2A2 | -0.2062616 | 2.10E-06 | 9.45E-06 |
| ME1 | -0.2062212 | 2.11E-06 | 9.49E-06 |
| GTF2B | 0.2061401 | 2.13E-06 | 9.58E-06 |
| PTGIS | -0.2061367 | 2.13E-06 | 9.58E-06 |
| TPM1 | -0.2061093 | 2.14E-06 | 9.61E-06 |
| RAI1 | -0.2060905 | 2.15E-06 | 9.63E-06 |
| APOBEC2 | -0.2060843 | 2.15E-06 | 9.63E-06 |
| LOC100126784 | -0.2060439 | 2.16E-06 | 9.68E-06 |
| FAM102A | -0.2059889 | 2.17E-06 | 9.73E-06 |
| FARSA | 0.2059686 | 2.18E-06 | 9.76E-06 |
| SLC14A2 | -0.2059333 | 2.18E-06 | 9.79E-06 |
| DUPD1 | -0.205917 | 2.19E-06 | 9.81E-06 |
| SEMA5A | -0.2059033 | 2.19E-06 | 9.82E-06 |
| NACAD | -0.2058546 | 2.20E-06 | 9.88E-06 |
| RIT1 | -0.2058364 | 2.21E-06 | 9.90E-06 |
| KCNQ4 | -0.2057682 | 2.23E-06 | 9.97E-06 |
| IFNG | 0.2057467 | 2.23E-06 | 9.99E-06 |
| NLE1 | 0.2056935 | 2.25E-06 | 1.01E-05 |
| NPBWR1 | -0.2056816 | 2.25E-06 | 1.01E-05 |
| SLC37A3 | -0.2056207 | 2.27E-06 | 1.01E-05 |
| MRPS18A | 0.2055998 | 2.27E-06 | 1.02E-05 |
| FKBP10 | -0.2055937 | 2.27E-06 | 1.02E-05 |
| TRAPPC2 | 0.2055934 | 2.27E-06 | 1.02E-05 |
| HGF | -0.205589 | 2.27E-06 | 1.02E-05 |
| BMP1 | -0.2055624 | 2.28E-06 | 1.02E-05 |
| ELTD1 | -0.2055501 | 2.28E-06 | 1.02E-05 |
| HIST1H4A | 0.2055466 | 2.28E-06 | 1.02E-05 |
| RPUSD1 | 0.2055391 | 2.29E-06 | 1.02E-05 |
| CHD6 | -0.205506 | 2.30E-06 | 1.02E-05 |
| CNOT4 | -0.2054345 | 2.31E-06 | 1.03E-05 |
| FAM73B | 0.2054114 | 2.32E-06 | 1.04E-05 |
| RABGGTB | 0.205403 | 2.32E-06 | 1.04E-05 |
| IPO9 | 0.2054008 | 2.32E-06 | 1.04E-05 |
| FRYL | -0.205349 | 2.34E-06 | 1.04E-05 |
| CDA | -0.2053428 | 2.34E-06 | 1.04E-05 |
| RNF214 | -0.2053418 | 2.34E-06 | 1.04E-05 |
| NSF | -0.2053326 | 2.34E-06 | 1.04E-05 |
| PPP2R5B | -0.2053286 | 2.34E-06 | 1.04E-05 |
| NKRF | 0.2052937 | 2.35E-06 | 1.05E-05 |
| PCDHA3 | -0.2052224 | 2.37E-06 | 1.06E-05 |
| BEND7 | -0.2052188 | 2.37E-06 | 1.06E-05 |
| TOX2 | -0.2052167 | 2.37E-06 | 1.06E-05 |
| C7orf11 | 0.2051984 | 2.38E-06 | 1.06E-05 |
| ACAN | -0.2051664 | 2.39E-06 | 1.06E-05 |
| ANXA13 | -0.2051374 | 2.40E-06 | 1.07E-05 |
| CPD | -0.205107 | 2.40E-06 | 1.07E-05 |
| CYP7B1 | -0.2051065 | 2.40E-06 | 1.07E-05 |
| NIPBL | -0.2049757 | 2.44E-06 | 1.09E-05 |
| C19orf57 | 0.2049712 | 2.44E-06 | 1.09E-05 |
| TMEM132B | -0.2049638 | 2.44E-06 | 1.09E-05 |
| TMEM63B | -0.2049226 | 2.46E-06 | 1.09E-05 |
| ADAM23 | -0.2049085 | 2.46E-06 | 1.09E-05 |
| GATA1 | -0.2048194 | 2.48E-06 | 1.10E-05 |
| C1orf159 | 0.2047866 | 2.49E-06 | 1.11E-05 |
| RTN4RL2 | -0.204781 | 2.50E-06 | 1.11E-05 |
| ZBTB8OS | 0.2047796 | 2.50E-06 | 1.11E-05 |
| ERLIN1 | 0.2047545 | 2.50E-06 | 1.11E-05 |
| AXL | -0.2047527 | 2.50E-06 | 1.11E-05 |
| RNF169 | -0.2046918 | 2.52E-06 | 1.12E-05 |
| NUPL2 | 0.2046872 | 2.52E-06 | 1.12E-05 |
| CHST3 | -0.2046269 | 2.54E-06 | 1.13E-05 |
| MAPK7 | -0.2046161 | 2.54E-06 | 1.13E-05 |
| C22orf40 | 0.2045829 | 2.55E-06 | 1.13E-05 |
| EDNRB | -0.2045665 | 2.56E-06 | 1.13E-05 |
| SEZ6L | -0.2045663 | 2.56E-06 | 1.13E-05 |
| COG6 | -0.2045601 | 2.56E-06 | 1.13E-05 |
| ABHD10 | 0.2045374 | 2.57E-06 | 1.14E-05 |
| SLMO2 | 0.2045291 | 2.57E-06 | 1.14E-05 |
| STX12 | -0.2045227 | 2.57E-06 | 1.14E-05 |
| AGPAT4 | -0.2044524 | 2.59E-06 | 1.15E-05 |
| SERPINB7 | -0.2044469 | 2.59E-06 | 1.15E-05 |
| SRGAP1 | -0.2043975 | 2.61E-06 | 1.15E-05 |
| BOLA1 | 0.2043025 | 2.64E-06 | 1.17E-05 |
| HS3ST2 | -0.2043024 | 2.64E-06 | 1.17E-05 |
| FOXF1 | -0.2042834 | 2.64E-06 | 1.17E-05 |
| ARL10 | -0.2042302 | 2.66E-06 | 1.18E-05 |
| DNAJC16 | -0.204221 | 2.66E-06 | 1.18E-05 |
| ZNF439 | -0.2042172 | 2.66E-06 | 1.18E-05 |
| TOLLIP | -0.2041934 | 2.67E-06 | 1.18E-05 |
| LOC440957 | 0.2041856 | 2.67E-06 | 1.18E-05 |
| PDXP | 0.2041544 | 2.68E-06 | 1.18E-05 |
| TMTC1 | -0.2041388 | 2.69E-06 | 1.19E-05 |
| SLC4A3 | -0.204112 | 2.70E-06 | 1.19E-05 |
| C6orf97 | -0.2040995 | 2.70E-06 | 1.19E-05 |
| BRF2 | 0.2039927 | 2.73E-06 | 1.21E-05 |
| COBRA1 | 0.2039774 | 2.74E-06 | 1.21E-05 |
| RGS14 | 0.2039681 | 2.74E-06 | 1.21E-05 |
| HIST2H3C | 0.2039672 | 2.74E-06 | 1.21E-05 |
| NOL8 | 0.2039566 | 2.74E-06 | 1.21E-05 |
| WDR53 | 0.2039175 | 2.76E-06 | 1.21E-05 |
| SLN | -0.203897 | 2.76E-06 | 1.22E-05 |
| CUEDC2 | 0.2038174 | 2.79E-06 | 1.23E-05 |
| HECTD3 | -0.2037892 | 2.80E-06 | 1.23E-05 |
| HMX2 | 0.2037733 | 2.80E-06 | 1.23E-05 |
| AMMECR1L | -0.203747 | 2.81E-06 | 1.24E-05 |
| FABP3 | -0.2037303 | 2.82E-06 | 1.24E-05 |
| ZNF192 | -0.2036744 | 2.83E-06 | 1.25E-05 |
| TAX1BP1 | -0.2035635 | 2.87E-06 | 1.26E-05 |
| C1orf58 | -0.2035627 | 2.87E-06 | 1.26E-05 |
| KLK8 | -0.2035522 | 2.87E-06 | 1.26E-05 |
| DES | -0.2035416 | 2.88E-06 | 1.26E-05 |
| RFWD3 | 0.2035414 | 2.88E-06 | 1.26E-05 |
| RNF222 | -0.2035268 | 2.88E-06 | 1.27E-05 |
| RNF123 | -0.2035246 | 2.88E-06 | 1.27E-05 |
| STAC | -0.2035041 | 2.89E-06 | 1.27E-05 |
| PITPNM2 | -0.2034818 | 2.90E-06 | 1.27E-05 |
| ZNF556 | -0.2034716 | 2.90E-06 | 1.27E-05 |
| C15orf57 | 0.2034225 | 2.92E-06 | 1.28E-05 |
| RPF2 | 0.2034219 | 2.92E-06 | 1.28E-05 |
| CSH2 | 0.2034129 | 2.92E-06 | 1.28E-05 |
| SYCP3 | 0.2033553 | 2.94E-06 | 1.29E-05 |
| FNDC5 | -0.2033221 | 2.95E-06 | 1.29E-05 |
| TCP11L1 | -0.2032644 | 2.97E-06 | 1.30E-05 |
| RPL37A | 0.2032135 | 2.99E-06 | 1.31E-05 |
| MYOD1 | -0.2031894 | 3.00E-06 | 1.31E-05 |
| C12orf29 | 0.2031842 | 3.00E-06 | 1.31E-05 |
| HAPLN4 | -0.2031743 | 3.00E-06 | 1.31E-05 |
| MCAT | 0.2031683 | 3.00E-06 | 1.32E-05 |
| MARVELD1 | -0.2031642 | 3.00E-06 | 1.32E-05 |
| SCRN1 | -0.2031259 | 3.02E-06 | 1.32E-05 |
| CSTF1 | 0.2031132 | 3.02E-06 | 1.32E-05 |
| C15orf52 | -0.2031111 | 3.02E-06 | 1.32E-05 |
| KCNF1 | -0.2030798 | 3.03E-06 | 1.33E-05 |
| ALG5 | 0.2030527 | 3.04E-06 | 1.33E-05 |
| FBXO40 | -0.2030102 | 3.06E-06 | 1.34E-05 |
| TBC1D9B | -0.202991 | 3.06E-06 | 1.34E-05 |
| NEU3 | -0.2029581 | 3.08E-06 | 1.34E-05 |
| C5orf44 | 0.202947 | 3.08E-06 | 1.35E-05 |
| HCN3 | 0.2029211 | 3.09E-06 | 1.35E-05 |
| RARRES2 | -0.2029198 | 3.09E-06 | 1.35E-05 |
| GEMIN8P4 | 0.2029106 | 3.09E-06 | 1.35E-05 |
| C20orf166 | -0.2028487 | 3.11E-06 | 1.36E-05 |
| ZMYND17 | 0.202826 | 3.12E-06 | 1.36E-05 |
| C16orf58 | -0.2027983 | 3.13E-06 | 1.37E-05 |
| PAPPA2 | -0.2027599 | 3.15E-06 | 1.37E-05 |
| MAP2K1 | 0.2027385 | 3.15E-06 | 1.38E-05 |
| IGSF3 | -0.2027314 | 3.16E-06 | 1.38E-05 |
| HN1L | 0.2027144 | 3.16E-06 | 1.38E-05 |
| DYX1C1 | 0.202708 | 3.17E-06 | 1.38E-05 |
| FBXW11 | -0.2026902 | 3.17E-06 | 1.38E-05 |
| SCLY | 0.2026833 | 3.17E-06 | 1.38E-05 |
| CCDC66 | 0.2026549 | 3.18E-06 | 1.39E-05 |
| ZIK1 | -0.2026352 | 3.19E-06 | 1.39E-05 |
| ZNF578 | -0.2026211 | 3.20E-06 | 1.39E-05 |
| LPAR1 | -0.2026093 | 3.20E-06 | 1.39E-05 |
| TNNT3 | -0.2026073 | 3.20E-06 | 1.39E-05 |
| COG3 | -0.2025853 | 3.21E-06 | 1.40E-05 |
| HCFC2 | -0.2025754 | 3.21E-06 | 1.40E-05 |
| DDX56 | 0.2025238 | 3.23E-06 | 1.41E-05 |
| CCDC113 | -0.2025158 | 3.24E-06 | 1.41E-05 |
| TAF6L | 0.2024678 | 3.25E-06 | 1.41E-05 |
| BEST1 | -0.2024666 | 3.25E-06 | 1.41E-05 |
| LOC283731 | -0.2024558 | 3.26E-06 | 1.42E-05 |
| KRT6A | -0.2024553 | 3.26E-06 | 1.42E-05 |
| TXN2 | 0.2024047 | 3.28E-06 | 1.42E-05 |
| ERCC4 | -0.2023733 | 3.29E-06 | 1.43E-05 |
| CCT6P1 | 0.2023579 | 3.29E-06 | 1.43E-05 |
| MRPL15 | 0.2023159 | 3.31E-06 | 1.44E-05 |
| MRPS35 | 0.2022981 | 3.32E-06 | 1.44E-05 |
| TMEM182 | -0.2022637 | 3.33E-06 | 1.44E-05 |
| NEU2 | -0.2022511 | 3.33E-06 | 1.45E-05 |
| EPS15L1 | -0.2022322 | 3.34E-06 | 1.45E-05 |
| SMPX | -0.2022248 | 3.34E-06 | 1.45E-05 |
| IQCG | 0.2022227 | 3.35E-06 | 1.45E-05 |
| CTSO | -0.2022094 | 3.35E-06 | 1.45E-05 |
| GATSL3 | -0.2022024 | 3.35E-06 | 1.45E-05 |
| PSMC6 | 0.2022014 | 3.35E-06 | 1.45E-05 |
| DHRS11 | 0.2021099 | 3.39E-06 | 1.47E-05 |
| FLVCR2 | -0.2020775 | 3.40E-06 | 1.47E-05 |
| OAF | -0.2020561 | 3.41E-06 | 1.48E-05 |
| PAX6 | 0.2020325 | 3.42E-06 | 1.48E-05 |
| GRIP2 | -0.2020211 | 3.42E-06 | 1.48E-05 |
| ATP11B | -0.2020157 | 3.42E-06 | 1.48E-05 |
| RTF1 | 0.2020008 | 3.43E-06 | 1.48E-05 |
| MAGEH1 | -0.2019632 | 3.45E-06 | 1.49E-05 |
| PIKFYVE | -0.2019562 | 3.45E-06 | 1.49E-05 |
| ID4 | -0.20184 | 3.49E-06 | 1.51E-05 |
| GNG10 | 0.2018118 | 3.51E-06 | 1.52E-05 |
| SERBP1 | 0.2018068 | 3.51E-06 | 1.52E-05 |
| RNF185 | -0.2017674 | 3.52E-06 | 1.52E-05 |
| OLFML2A | -0.2017371 | 3.53E-06 | 1.53E-05 |
| VPS33B | 0.2016252 | 3.58E-06 | 1.55E-05 |
| ENC1 | -0.201576 | 3.60E-06 | 1.55E-05 |
| FLNA | -0.2015526 | 3.61E-06 | 1.56E-05 |
| CIDEB | 0.2015436 | 3.61E-06 | 1.56E-05 |
| PLA2G15 | -0.2015393 | 3.62E-06 | 1.56E-05 |
| SLCO2B1 | -0.2015376 | 3.62E-06 | 1.56E-05 |
| DEF6 | 0.2015262 | 3.62E-06 | 1.56E-05 |
| SSB | 0.2014873 | 3.64E-06 | 1.57E-05 |
| ZNF710 | -0.2014465 | 3.65E-06 | 1.58E-05 |
| RG9MTD2 | 0.2014271 | 3.66E-06 | 1.58E-05 |
| ESRRG | -0.2014121 | 3.67E-06 | 1.58E-05 |
| ATP5EP2 | 0.2013849 | 3.68E-06 | 1.59E-05 |
| HRH1 | -0.2013106 | 3.71E-06 | 1.60E-05 |
| GPHA2 | 0.2012695 | 3.73E-06 | 1.61E-05 |
| SPOCD1 | -0.2012394 | 3.74E-06 | 1.61E-05 |
| CASC5 | 0.2011296 | 3.79E-06 | 1.63E-05 |
| ABI3BP | -0.2011245 | 3.79E-06 | 1.63E-05 |
| WASH7P | 0.2010936 | 3.80E-06 | 1.64E-05 |
| COL4A6 | -0.2010646 | 3.81E-06 | 1.64E-05 |
| C6orf70 | 0.2010452 | 3.82E-06 | 1.64E-05 |
| SLC44A5 | -0.2010411 | 3.83E-06 | 1.64E-05 |
| PERP | -0.201032 | 3.83E-06 | 1.65E-05 |
| PCDHB13 | -0.201015 | 3.84E-06 | 1.65E-05 |
| ZNF532 | -0.200965 | 3.86E-06 | 1.66E-05 |
| APIP | 0.2009572 | 3.86E-06 | 1.66E-05 |
| SERINC4 | 0.2009549 | 3.86E-06 | 1.66E-05 |
| PSAT1 | 0.2008877 | 3.89E-06 | 1.67E-05 |
| PGR | -0.2008527 | 3.91E-06 | 1.68E-05 |
| YDJC | 0.2008398 | 3.91E-06 | 1.68E-05 |
| HSP90AB4P | 0.2008089 | 3.93E-06 | 1.69E-05 |
| ZNF493 | -0.2008067 | 3.93E-06 | 1.69E-05 |
| ATP5L | 0.2008003 | 3.93E-06 | 1.69E-05 |
| PTHLH | -0.2007837 | 3.94E-06 | 1.69E-05 |
| RASD2 | -0.2007818 | 3.94E-06 | 1.69E-05 |
| THRA | -0.2007072 | 3.97E-06 | 1.70E-05 |
| NGF | -0.2006919 | 3.98E-06 | 1.71E-05 |
| DNM1P35 | 0.2006892 | 3.98E-06 | 1.71E-05 |
| TMEM63C | -0.2006676 | 3.99E-06 | 1.71E-05 |
| GTDC1 | -0.2006229 | 4.01E-06 | 1.72E-05 |
| KIFC3 | -0.2006028 | 4.02E-06 | 1.72E-05 |
| PPP3CA | -0.2005936 | 4.02E-06 | 1.72E-05 |
| CHSY1 | -0.2005749 | 4.03E-06 | 1.73E-05 |
| CTNS | -0.2005639 | 4.04E-06 | 1.73E-05 |
| BNIP2 | 0.2005397 | 4.05E-06 | 1.73E-05 |
| CCDC109B | 0.2004711 | 4.08E-06 | 1.75E-05 |
| C9orf80 | 0.2004655 | 4.08E-06 | 1.75E-05 |
| BCAP29 | 0.2004612 | 4.08E-06 | 1.75E-05 |
| ASB11 | -0.2004446 | 4.09E-06 | 1.75E-05 |
| HMOX1 | -0.2003863 | 4.12E-06 | 1.76E-05 |
| LDHB | 0.2003637 | 4.13E-06 | 1.76E-05 |
| GLRX3 | 0.2003622 | 4.13E-06 | 1.76E-05 |
| PLXNA3 | -0.2003408 | 4.14E-06 | 1.77E-05 |
| PTPN5 | -0.2003354 | 4.14E-06 | 1.77E-05 |
| MRPS28 | 0.2003003 | 4.16E-06 | 1.78E-05 |
| PPM1N | 0.2002589 | 4.18E-06 | 1.78E-05 |
| TRAK1 | -0.2002548 | 4.18E-06 | 1.78E-05 |
| SR140 | 0.2002517 | 4.18E-06 | 1.78E-05 |
| METAP2 | 0.2002063 | 4.20E-06 | 1.79E-05 |
| AMOTL2 | -0.2001964 | 4.21E-06 | 1.79E-05 |
| DIRC2 | -0.2001558 | 4.23E-06 | 1.80E-05 |
| YWHAG | -0.2001469 | 4.23E-06 | 1.80E-05 |
| STAB1 | -0.2001428 | 4.23E-06 | 1.80E-05 |
| PRMT7 | 0.2001427 | 4.23E-06 | 1.80E-05 |
| LOC152217 | 0.2001057 | 4.25E-06 | 1.81E-05 |
| DICER1 | -0.2000946 | 4.26E-06 | 1.81E-05 |
| KDELR3 | -0.2000909 | 4.26E-06 | 1.81E-05 |
| ARHGEF11 | -0.200065 | 4.27E-06 | 1.82E-05 |
| TLX2 | 0.2000596 | 4.27E-06 | 1.82E-05 |
| TET3 | -0.2000473 | 4.28E-06 | 1.82E-05 |
| RTN1 | -0.2000472 | 4.28E-06 | 1.82E-05 |
| LSAMP | -0.2000321 | 4.29E-06 | 1.82E-05 |
| KLF12 | -0.2000036 | 4.30E-06 | 1.83E-05 |
| CEP170 | -0.1999452 | 4.33E-06 | 1.84E-05 |
| CARD6 | -0.1999355 | 4.33E-06 | 1.84E-05 |
| MGST2 | 0.1998488 | 4.38E-06 | 1.86E-05 |
| ELAC1 | 0.1997953 | 4.40E-06 | 1.87E-05 |
| CALCRL | -0.1997769 | 4.41E-06 | 1.87E-05 |
| KRT80 | -0.1997276 | 4.44E-06 | 1.88E-05 |
| DPM3 | 0.1997064 | 4.45E-06 | 1.89E-05 |
| LRCH2 | -0.1996818 | 4.46E-06 | 1.89E-05 |
| ITGA10 | -0.1996426 | 4.48E-06 | 1.90E-05 |
| ANKRD17 | -0.199635 | 4.48E-06 | 1.90E-05 |
| YLPM1 | -0.1995735 | 4.51E-06 | 1.92E-05 |
| AGAP6 | 0.1995672 | 4.52E-06 | 1.92E-05 |
| LCE3D | -0.1995647 | 4.52E-06 | 1.92E-05 |
| SH3GLB1 | -0.199535 | 4.53E-06 | 1.92E-05 |
| SPTB | -0.1995055 | 4.55E-06 | 1.93E-05 |
| ITPR1 | -0.1994839 | 4.56E-06 | 1.93E-05 |
| HIATL2 | 0.1994498 | 4.58E-06 | 1.94E-05 |
| PDE4B | -0.199449 | 4.58E-06 | 1.94E-05 |
| SOHLH2 | -0.1994215 | 4.59E-06 | 1.95E-05 |
| ATAD3B | 0.1993922 | 4.61E-06 | 1.95E-05 |
| LOC729991 | 0.1993802 | 4.61E-06 | 1.95E-05 |
| ARHGEF4 | -0.1993051 | 4.65E-06 | 1.97E-05 |
| NOSIP | 0.1993027 | 4.65E-06 | 1.97E-05 |
| ZP3 | 0.1993013 | 4.65E-06 | 1.97E-05 |
| FKBP6 | 0.1992968 | 4.66E-06 | 1.97E-05 |
| LOC100271836 | -0.199279 | 4.66E-06 | 1.97E-05 |
| MINK1 | -0.1992741 | 4.67E-06 | 1.97E-05 |
| DAPK1 | -0.199274 | 4.67E-06 | 1.97E-05 |
| THBS4 | -0.1992369 | 4.69E-06 | 1.98E-05 |
| GPD1 | -0.1992287 | 4.69E-06 | 1.98E-05 |
| NT5C1A | -0.1992012 | 4.71E-06 | 1.99E-05 |
| FAM20A | -0.1991652 | 4.72E-06 | 2.00E-05 |
| CCNT1 | -0.1991608 | 4.73E-06 | 2.00E-05 |
| EXOC7 | -0.1991584 | 4.73E-06 | 2.00E-05 |
| NHEJ1 | 0.1991502 | 4.73E-06 | 2.00E-05 |
| CAT | -0.1991399 | 4.74E-06 | 2.00E-05 |
| RBM4B | 0.199114 | 4.75E-06 | 2.01E-05 |
| POP4 | 0.1990708 | 4.77E-06 | 2.02E-05 |
| SPATA6 | -0.1990016 | 4.81E-06 | 2.03E-05 |
| KLHL6 | -0.1989972 | 4.81E-06 | 2.03E-05 |
| HDAC4 | -0.1989939 | 4.82E-06 | 2.03E-05 |
| DACH1 | -0.1989517 | 4.84E-06 | 2.04E-05 |
| CSRP3 | -0.1989366 | 4.85E-06 | 2.04E-05 |
| SLC39A6 | -0.1989361 | 4.85E-06 | 2.04E-05 |
| CDK12 | -0.1989085 | 4.86E-06 | 2.05E-05 |
| MTF1 | -0.1988989 | 4.87E-06 | 2.05E-05 |
| MGC16142 | 0.1988766 | 4.88E-06 | 2.06E-05 |
| LRRC57 | 0.1988326 | 4.90E-06 | 2.07E-05 |
| RAD51L3 | 0.1988167 | 4.91E-06 | 2.07E-05 |
| 3-Mar | -0.1988003 | 4.92E-06 | 2.07E-05 |
| CNST | -0.1987969 | 4.92E-06 | 2.07E-05 |
| MRM1 | 0.1987687 | 4.94E-06 | 2.08E-05 |
| NAT1 | 0.1987184 | 4.97E-06 | 2.09E-05 |
| C2orf34 | 0.1987137 | 4.97E-06 | 2.09E-05 |
| POLR3C | 0.1986994 | 4.98E-06 | 2.09E-05 |
| CNOT8 | 0.198699 | 4.98E-06 | 2.09E-05 |
| COMTD1 | 0.198662 | 5.00E-06 | 2.10E-05 |
| ZNF583 | -0.1986453 | 5.01E-06 | 2.10E-05 |
| TNNC1 | -0.1986407 | 5.01E-06 | 2.11E-05 |
| C12orf49 | -0.1986033 | 5.03E-06 | 2.11E-05 |
| C9orf68 | 0.1985747 | 5.05E-06 | 2.12E-05 |
| CLSTN1 | -0.1985702 | 5.05E-06 | 2.12E-05 |
| TBCK | -0.1985317 | 5.07E-06 | 2.13E-05 |
| KHNYN | -0.1985031 | 5.09E-06 | 2.14E-05 |
| EIF5A | 0.1984808 | 5.10E-06 | 2.14E-05 |
| MUC15 | -0.19845 | 5.12E-06 | 2.15E-05 |
| SUSD5 | -0.1983686 | 5.16E-06 | 2.17E-05 |
| GADD45B | -0.1983489 | 5.18E-06 | 2.17E-05 |
| C19orf12 | -0.1983305 | 5.19E-06 | 2.17E-05 |
| PTCD2 | 0.1982731 | 5.22E-06 | 2.19E-05 |
| DMRTA2 | 0.1982664 | 5.22E-06 | 2.19E-05 |
| HBEGF | -0.198265 | 5.22E-06 | 2.19E-05 |
| GSC | -0.1982638 | 5.23E-06 | 2.19E-05 |
| C10orf140 | 0.1982483 | 5.23E-06 | 2.19E-05 |
| SCYL2 | -0.1982132 | 5.25E-06 | 2.20E-05 |
| TBX18 | -0.1981829 | 5.27E-06 | 2.21E-05 |
| KBTBD5 | -0.1981653 | 5.28E-06 | 2.21E-05 |
| MEIS3P1 | -0.1981259 | 5.31E-06 | 2.22E-05 |
| C7orf60 | -0.1981061 | 5.32E-06 | 2.23E-05 |
| ADSL | 0.1980974 | 5.32E-06 | 2.23E-05 |
| ARMCX3 | -0.1980586 | 5.35E-06 | 2.24E-05 |
| ZRSR2 | 0.1980421 | 5.36E-06 | 2.24E-05 |
| SLFN5 | -0.1980356 | 5.36E-06 | 2.24E-05 |
| LAMP2 | -0.1980064 | 5.38E-06 | 2.25E-05 |
| LARGE | -0.1980005 | 5.38E-06 | 2.25E-05 |
| PDGFD | -0.1979897 | 5.39E-06 | 2.25E-05 |
| MRPL10 | 0.1979878 | 5.39E-06 | 2.25E-05 |
| C14orf181 | 0.1979855 | 5.39E-06 | 2.25E-05 |
| TRIM59 | 0.1979642 | 5.40E-06 | 2.26E-05 |
| GLCE | 0.1979011 | 5.44E-06 | 2.27E-05 |
| SLC2A8 | 0.1978995 | 5.44E-06 | 2.27E-05 |
| MYADML2 | -0.1978893 | 5.45E-06 | 2.27E-05 |
| CDK13 | -0.1978525 | 5.47E-06 | 2.28E-05 |
| TPRN | 0.1978445 | 5.48E-06 | 2.28E-05 |
| PRND | -0.1978441 | 5.48E-06 | 2.28E-05 |
| DNAJB9 | -0.1978336 | 5.48E-06 | 2.29E-05 |
| SOS1 | -0.1978168 | 5.49E-06 | 2.29E-05 |
| BAG5 | -0.1978014 | 5.50E-06 | 2.29E-05 |
| RPS3 | 0.1977963 | 5.50E-06 | 2.29E-05 |
| NPC1 | -0.1977839 | 5.51E-06 | 2.30E-05 |
| GPC4 | -0.1977728 | 5.52E-06 | 2.30E-05 |
| ATG10 | 0.1977685 | 5.52E-06 | 2.30E-05 |
| MB | -0.1977667 | 5.52E-06 | 2.30E-05 |
| TUBB2A | -0.1977624 | 5.53E-06 | 2.30E-05 |
| LRRC4B | -0.1977567 | 5.53E-06 | 2.30E-05 |
| SGMS2 | -0.1977474 | 5.53E-06 | 2.30E-05 |
| C1D | 0.1976532 | 5.59E-06 | 2.33E-05 |
| KLHL33 | -0.1976173 | 5.62E-06 | 2.34E-05 |
| IGSF9B | -0.1976049 | 5.62E-06 | 2.34E-05 |
| SMPD3 | -0.1975752 | 5.64E-06 | 2.35E-05 |
| ZNF542 | -0.1975567 | 5.65E-06 | 2.35E-05 |
| LRRC8E | -0.1975361 | 5.67E-06 | 2.35E-05 |
| LNPEP | -0.1975254 | 5.67E-06 | 2.36E-05 |
| TACR1 | -0.1975181 | 5.68E-06 | 2.36E-05 |
| TAF9B | 0.1974865 | 5.70E-06 | 2.37E-05 |
| NPEPPS | -0.1974828 | 5.70E-06 | 2.37E-05 |
| RAET1K | 0.1974792 | 5.70E-06 | 2.37E-05 |
| NR2C1 | 0.1974446 | 5.72E-06 | 2.38E-05 |
| MTERF | 0.1973969 | 5.75E-06 | 2.39E-05 |
| SDK1 | -0.1973618 | 5.78E-06 | 2.40E-05 |
| SERPING1 | -0.1972996 | 5.82E-06 | 2.41E-05 |
| EIF3F | 0.1972882 | 5.82E-06 | 2.42E-05 |
| NLRP12 | -0.1972806 | 5.83E-06 | 2.42E-05 |
| BPI | -0.1972685 | 5.84E-06 | 2.42E-05 |
| CRYZL1 | 0.1972547 | 5.85E-06 | 2.42E-05 |
| FOXO4 | -0.1972532 | 5.85E-06 | 2.42E-05 |
| ARMC6 | 0.197241 | 5.86E-06 | 2.43E-05 |
| DDX49 | 0.1972332 | 5.86E-06 | 2.43E-05 |
| ASAP1 | -0.1972052 | 5.88E-06 | 2.43E-05 |
| ADAMTS3 | -0.1971845 | 5.89E-06 | 2.44E-05 |
| MFSD6 | -0.1970681 | 5.97E-06 | 2.47E-05 |
| TNXB | -0.1970351 | 5.99E-06 | 2.48E-05 |
| POLR2L | 0.1970221 | 6.00E-06 | 2.48E-05 |
| ASNS | 0.1970119 | 6.01E-06 | 2.48E-05 |
| C5AR1 | -0.1970113 | 6.01E-06 | 2.48E-05 |
| FBXO4 | 0.1969621 | 6.04E-06 | 2.50E-05 |
| ZGLP1 | 0.196956 | 6.04E-06 | 2.50E-05 |
| SLC40A1 | -0.1968285 | 6.13E-06 | 2.53E-05 |
| TOMM40L | 0.1968236 | 6.13E-06 | 2.53E-05 |
| ZFP36 | -0.1968178 | 6.14E-06 | 2.54E-05 |
| PIPSL | 0.1967903 | 6.16E-06 | 2.54E-05 |
| ATF2 | -0.1967673 | 6.17E-06 | 2.55E-05 |
| SHPRH | -0.1967651 | 6.17E-06 | 2.55E-05 |
| EEPD1 | -0.1967603 | 6.18E-06 | 2.55E-05 |
| DUSP26 | -0.1967587 | 6.18E-06 | 2.55E-05 |
| SBF1 | -0.1967244 | 6.20E-06 | 2.56E-05 |
| LRCH1 | -0.1967121 | 6.21E-06 | 2.56E-05 |
| METTL12 | 0.1967019 | 6.22E-06 | 2.56E-05 |
| FAM124A | -0.1966757 | 6.23E-06 | 2.57E-05 |
| GALNT2 | -0.1966522 | 6.25E-06 | 2.58E-05 |
| MED1 | -0.1966108 | 6.28E-06 | 2.59E-05 |
| DKFZp434J0226 | -0.1965896 | 6.29E-06 | 2.59E-05 |
| YIPF3 | -0.196583 | 6.30E-06 | 2.60E-05 |
| DIMT1L | 0.1965718 | 6.31E-06 | 2.60E-05 |
| HARS2 | 0.1965706 | 6.31E-06 | 2.60E-05 |
| SSH2 | -0.1965271 | 6.34E-06 | 2.61E-05 |
| ZNF772 | -0.1965103 | 6.35E-06 | 2.61E-05 |
| GPR107 | -0.1965025 | 6.35E-06 | 2.62E-05 |
| FYN | -0.196482 | 6.37E-06 | 2.62E-05 |
| PADI2 | -0.196473 | 6.37E-06 | 2.62E-05 |
| VDAC1 | 0.1964673 | 6.38E-06 | 2.62E-05 |
| CDR2L | -0.1964618 | 6.38E-06 | 2.63E-05 |
| WDTC1 | -0.196461 | 6.38E-06 | 2.63E-05 |
| AKR1B10 | -0.1964336 | 6.40E-06 | 2.63E-05 |
| C9orf129 | -0.1964072 | 6.42E-06 | 2.64E-05 |
| ADAMTS18 | -0.1963973 | 6.43E-06 | 2.64E-05 |
| PSMC1 | 0.1963722 | 6.45E-06 | 2.65E-05 |
| EIF4A3 | 0.1963409 | 6.47E-06 | 2.66E-05 |
| SFRS4 | 0.1963289 | 6.48E-06 | 2.66E-05 |
| SF3B5 | 0.196324 | 6.48E-06 | 2.66E-05 |
| SEC23IP | -0.1962633 | 6.52E-06 | 2.68E-05 |
| HARS | 0.1962407 | 6.54E-06 | 2.68E-05 |
| CDK16 | 0.1962347 | 6.54E-06 | 2.69E-05 |
| SH3BP4 | -0.1962314 | 6.55E-06 | 2.69E-05 |
| KLK10 | -0.1962276 | 6.55E-06 | 2.69E-05 |
| LOC388789 | 0.1962253 | 6.55E-06 | 2.69E-05 |
| CSRNP1 | -0.1962106 | 6.56E-06 | 2.69E-05 |
| GKAP1 | 0.1961818 | 6.58E-06 | 2.70E-05 |
| BHLHE22 | -0.1961678 | 6.59E-06 | 2.70E-05 |
| RPTOR | -0.1961164 | 6.63E-06 | 2.72E-05 |
| SLC7A1 | -0.1960963 | 6.65E-06 | 2.72E-05 |
| SNW1 | 0.1960904 | 6.65E-06 | 2.72E-05 |
| GTPBP10 | 0.1960659 | 6.67E-06 | 2.73E-05 |
| HYI | 0.1960655 | 6.67E-06 | 2.73E-05 |
| ZNFX1 | -0.1960581 | 6.67E-06 | 2.73E-05 |
| GBA | -0.1960469 | 6.68E-06 | 2.74E-05 |
| PIK3C2A | -0.1960439 | 6.68E-06 | 2.74E-05 |
| ARHGEF33 | 0.1960246 | 6.70E-06 | 2.74E-05 |
| GNAO1 | -0.1960204 | 6.70E-06 | 2.74E-05 |
| AGPHD1 | 0.1960062 | 6.71E-06 | 2.75E-05 |
| TMEM68 | 0.1959818 | 6.73E-06 | 2.75E-05 |
| FIP1L1 | 0.1959509 | 6.75E-06 | 2.76E-05 |
| DOCK7 | -0.1959382 | 6.76E-06 | 2.76E-05 |
| VWA1 | -0.1959196 | 6.78E-06 | 2.77E-05 |
| C8orf76 | 0.1958914 | 6.80E-06 | 2.78E-05 |
| MMP7 | -0.1958671 | 6.82E-06 | 2.78E-05 |
| SRPK2 | -0.1957873 | 6.88E-06 | 2.81E-05 |
| UQCR11 | 0.1957835 | 6.88E-06 | 2.81E-05 |
| CCDC141 | -0.1957826 | 6.88E-06 | 2.81E-05 |
| MCCC2 | 0.19578 | 6.88E-06 | 2.81E-05 |
| C14orf93 | 0.1957203 | 6.93E-06 | 2.83E-05 |
| PDIK1L | 0.1957088 | 6.94E-06 | 2.83E-05 |
| KRT79 | -0.1957029 | 6.94E-06 | 2.83E-05 |
| MAP3K4 | -0.1956977 | 6.94E-06 | 2.83E-05 |
| SNORD116-28 | -0.1956933 | 6.95E-06 | 2.83E-05 |
| LOC152024 | 0.1956106 | 7.01E-06 | 2.86E-05 |
| SGK223 | -0.1955869 | 7.03E-06 | 2.87E-05 |
| NLGN2 | -0.195572 | 7.04E-06 | 2.87E-05 |
| CDH20 | -0.1955239 | 7.08E-06 | 2.88E-05 |
| NYNRIN | -0.195516 | 7.08E-06 | 2.89E-05 |
| CDKN2B | -0.195491 | 7.10E-06 | 2.89E-05 |
| MECR | 0.1954538 | 7.13E-06 | 2.90E-05 |
| AGAP7 | 0.1954365 | 7.15E-06 | 2.91E-05 |
| WIPF1 | -0.1954348 | 7.15E-06 | 2.91E-05 |
| LIF | -0.1954013 | 7.17E-06 | 2.92E-05 |
| ALKBH8 | -0.1953795 | 7.19E-06 | 2.93E-05 |
| TTYH2 | -0.1953756 | 7.19E-06 | 2.93E-05 |
| ZNF649 | -0.1953676 | 7.20E-06 | 2.93E-05 |
| ATP6V1A | -0.1953304 | 7.23E-06 | 2.94E-05 |
| GABARAPL3 | -0.1953229 | 7.24E-06 | 2.94E-05 |
| BRD1 | -0.1953224 | 7.24E-06 | 2.94E-05 |
| C10orf75 | 0.1953187 | 7.24E-06 | 2.94E-05 |
| CYSLTR1 | -0.1953016 | 7.25E-06 | 2.95E-05 |
| ALG13 | 0.1952958 | 7.26E-06 | 2.95E-05 |
| CHRNA1 | -0.1952936 | 7.26E-06 | 2.95E-05 |
| PLCB4 | -0.1952795 | 7.27E-06 | 2.95E-05 |
| IL1RL1 | -0.1952498 | 7.29E-06 | 2.96E-05 |
| NCRNA00105 | 0.1952349 | 7.31E-06 | 2.97E-05 |
| FAM63A | -0.1952292 | 7.31E-06 | 2.97E-05 |
| GRLF1 | -0.1951848 | 7.35E-06 | 2.98E-05 |
| SPOCK3 | -0.1951805 | 7.35E-06 | 2.98E-05 |
| GABRB3 | -0.1951612 | 7.37E-06 | 2.99E-05 |
| HYDIN | -0.1950955 | 7.42E-06 | 3.01E-05 |
| PNMA1 | -0.1950842 | 7.43E-06 | 3.01E-05 |
| SUMO3 | 0.195077 | 7.43E-06 | 3.01E-05 |
| SLC39A2 | -0.1950532 | 7.45E-06 | 3.02E-05 |
| VPS37C | -0.195043 | 7.46E-06 | 3.02E-05 |
| NOB1 | 0.1950352 | 7.47E-06 | 3.03E-05 |
| DCST2 | 0.1949814 | 7.51E-06 | 3.04E-05 |
| ANKHD1-EIF4EBP3 | -0.1949683 | 7.52E-06 | 3.05E-05 |
| LOC388152 | 0.1949616 | 7.53E-06 | 3.05E-05 |
| MAT2A | 0.1949549 | 7.53E-06 | 3.05E-05 |
| RAB6C | -0.1949477 | 7.54E-06 | 3.05E-05 |
| ZNF157 | 0.1949273 | 7.56E-06 | 3.06E-05 |
| N4BP2 | -0.1949116 | 7.57E-06 | 3.06E-05 |
| GAPDH | 0.1949065 | 7.57E-06 | 3.06E-05 |
| C9orf86 | 0.1948414 | 7.63E-06 | 3.08E-05 |
| SERTAD2 | -0.1948338 | 7.64E-06 | 3.09E-05 |
| MST1P2 | 0.1947898 | 7.67E-06 | 3.10E-05 |
| C22orf41 | 0.1947582 | 7.70E-06 | 3.11E-05 |
| FDFT1 | 0.1947109 | 7.74E-06 | 3.13E-05 |
| SLITRK3 | -0.1947087 | 7.74E-06 | 3.13E-05 |
| HLX | -0.1947049 | 7.74E-06 | 3.13E-05 |
| RGS5 | -0.1946884 | 7.76E-06 | 3.13E-05 |
| SMTNL2 | -0.19463 | 7.81E-06 | 3.15E-05 |
| TEAD3 | -0.1946222 | 7.81E-06 | 3.15E-05 |
| LRRC2 | -0.1946015 | 7.83E-06 | 3.16E-05 |
| UGGT1 | -0.1945859 | 7.85E-06 | 3.17E-05 |
| LOC650623 | -0.1945616 | 7.87E-06 | 3.17E-05 |
| FAM106C | -0.1945601 | 7.87E-06 | 3.17E-05 |
| TBCE | 0.1945445 | 7.88E-06 | 3.18E-05 |
| LOC653653 | -0.1945111 | 7.91E-06 | 3.19E-05 |
| ARRDC1 | 0.1944927 | 7.93E-06 | 3.19E-05 |
| SAMD8 | -0.1944927 | 7.93E-06 | 3.19E-05 |
| ATE1 | -0.194491 | 7.93E-06 | 3.19E-05 |
| FRAS1 | -0.1944838 | 7.93E-06 | 3.20E-05 |
| WWC3 | -0.1944704 | 7.95E-06 | 3.20E-05 |
| GPR77 | -0.1944637 | 7.95E-06 | 3.20E-05 |
| CDH19 | -0.1944533 | 7.96E-06 | 3.20E-05 |
| C8G | 0.194451 | 7.96E-06 | 3.20E-05 |
| PLEKHB2 | -0.1944493 | 7.96E-06 | 3.20E-05 |
| ANKRD11 | -0.1944 | 8.01E-06 | 3.22E-05 |
| AIP | 0.1943897 | 8.02E-06 | 3.22E-05 |
| PAG1 | -0.1943406 | 8.06E-06 | 3.24E-05 |
| DLG3 | 0.1943266 | 8.07E-06 | 3.25E-05 |
| C19orf56 | 0.1943122 | 8.08E-06 | 3.25E-05 |
| THAP3 | 0.1942277 | 8.16E-06 | 3.28E-05 |
| GNA14 | -0.194227 | 8.16E-06 | 3.28E-05 |
| SLC25A28 | 0.1942211 | 8.17E-06 | 3.28E-05 |
| GPR3 | 0.1942169 | 8.17E-06 | 3.28E-05 |
| UGDH | -0.1941555 | 8.22E-06 | 3.30E-05 |
| CA8 | -0.1940978 | 8.28E-06 | 3.32E-05 |
| ZNF81 | -0.1940802 | 8.29E-06 | 3.33E-05 |
| HRAS | 0.1940739 | 8.30E-06 | 3.33E-05 |
| FBXO31 | -0.1940521 | 8.32E-06 | 3.34E-05 |
| EXT2 | -0.1940039 | 8.36E-06 | 3.35E-05 |
| ANKRD36BP1 | -0.1940035 | 8.36E-06 | 3.35E-05 |
| SF3B4 | 0.1939843 | 8.38E-06 | 3.36E-05 |
| SEPN1 | -0.1939764 | 8.39E-06 | 3.36E-05 |
| CCNE1 | 0.193966 | 8.40E-06 | 3.37E-05 |
| KRT77 | -0.1939583 | 8.40E-06 | 3.37E-05 |
| CDK5R1 | -0.1939581 | 8.40E-06 | 3.37E-05 |
| MAPT | -0.1939564 | 8.40E-06 | 3.37E-05 |
| GJB6 | -0.1939293 | 8.43E-06 | 3.38E-05 |
| LETM2 | 0.1939242 | 8.43E-06 | 3.38E-05 |
| HIP1 | -0.1939192 | 8.44E-06 | 3.38E-05 |
| FBXL4 | -0.1939175 | 8.44E-06 | 3.38E-05 |
| MKL1 | -0.1939072 | 8.45E-06 | 3.38E-05 |
| WDR7 | -0.1938356 | 8.52E-06 | 3.41E-05 |
| WDR1 | -0.1937715 | 8.58E-06 | 3.43E-05 |
| ZNF33B | 0.1937655 | 8.58E-06 | 3.43E-05 |
| METTL13 | 0.1937585 | 8.59E-06 | 3.43E-05 |
| IL28RA | -0.1937437 | 8.60E-06 | 3.44E-05 |
| FMN2 | -0.1937317 | 8.61E-06 | 3.44E-05 |
| ITGB1BP1 | 0.1937168 | 8.63E-06 | 3.45E-05 |
| PRODH2 | 0.1937094 | 8.63E-06 | 3.45E-05 |
| LCE6A | -0.193702 | 8.64E-06 | 3.45E-05 |
| C20orf27 | 0.193691 | 8.65E-06 | 3.46E-05 |
| WISP2 | -0.1936835 | 8.66E-06 | 3.46E-05 |
| SUCNR1 | -0.1936761 | 8.67E-06 | 3.46E-05 |
| ZC3H12C | -0.1936729 | 8.67E-06 | 3.46E-05 |
| GPR125 | -0.1936721 | 8.67E-06 | 3.46E-05 |
| C14orf45 | -0.1936718 | 8.67E-06 | 3.46E-05 |
| C10orf137 | 0.1936151 | 8.72E-06 | 3.48E-05 |
| DDX31 | 0.193611 | 8.73E-06 | 3.48E-05 |
| PANK3 | -0.1935738 | 8.76E-06 | 3.49E-05 |
| HEATR5B | -0.1935545 | 8.78E-06 | 3.50E-05 |
| FAM156A | 0.1935462 | 8.79E-06 | 3.50E-05 |
| CASP12 | -0.1935166 | 8.82E-06 | 3.51E-05 |
| MEX3B | -0.1935102 | 8.82E-06 | 3.52E-05 |
| NXT2 | 0.1934919 | 8.84E-06 | 3.52E-05 |
| KIAA1609 | -0.1934687 | 8.86E-06 | 3.53E-05 |
| FAM109A | -0.1934579 | 8.87E-06 | 3.53E-05 |
| C1orf89 | -0.1934429 | 8.89E-06 | 3.54E-05 |
| C22orf30 | -0.1934091 | 8.92E-06 | 3.55E-05 |
| IL7R | -0.1933757 | 8.95E-06 | 3.56E-05 |
| TPM4 | -0.1933412 | 8.99E-06 | 3.58E-05 |
| IL17RB | 0.1932782 | 9.05E-06 | 3.60E-05 |
| PIK3CG | -0.1932757 | 9.05E-06 | 3.60E-05 |
| BTBD8 | -0.1932091 | 9.12E-06 | 3.63E-05 |
| ZNF593 | 0.1931839 | 9.14E-06 | 3.64E-05 |
| C8orf22 | -0.1931797 | 9.15E-06 | 3.64E-05 |
| P4HA2 | -0.1931768 | 9.15E-06 | 3.64E-05 |
| YPEL4 | -0.1931759 | 9.15E-06 | 3.64E-05 |
| KISS1 | -0.1931696 | 9.16E-06 | 3.64E-05 |
| C2orf7 | 0.1931342 | 9.19E-06 | 3.65E-05 |
| C3orf57 | -0.193123 | 9.20E-06 | 3.66E-05 |
| VEPH1 | -0.1931093 | 9.22E-06 | 3.66E-05 |
| SARS2 | 0.1931089 | 9.22E-06 | 3.66E-05 |
| BCAS1 | -0.193099 | 9.23E-06 | 3.66E-05 |
| RPL37 | 0.1930831 | 9.24E-06 | 3.67E-05 |
| UQCRFS1 | 0.193068 | 9.26E-06 | 3.67E-05 |
| PCSK7 | -0.1930335 | 9.29E-06 | 3.69E-05 |
| C17orf88 | 0.1930114 | 9.32E-06 | 3.69E-05 |
| FDX1L | 0.192964 | 9.36E-06 | 3.71E-05 |
| PCDHB14 | -0.1929606 | 9.37E-06 | 3.71E-05 |
| PSAPL1 | -0.1929526 | 9.37E-06 | 3.72E-05 |
| CCDC84 | 0.1929373 | 9.39E-06 | 3.72E-05 |
| WNT7A | -0.192918 | 9.41E-06 | 3.73E-05 |
| FLOT2 | 0.192862 | 9.47E-06 | 3.75E-05 |
| SKP2 | 0.1928304 | 9.50E-06 | 3.76E-05 |
| FLJ33630 | 0.1928289 | 9.50E-06 | 3.76E-05 |
| HS6ST1 | -0.192803 | 9.53E-06 | 3.77E-05 |
| EXOC8 | -0.192796 | 9.54E-06 | 3.77E-05 |
| ZNF454 | -0.1927401 | 9.59E-06 | 3.80E-05 |
| CADM1 | -0.1927283 | 9.61E-06 | 3.80E-05 |
| SLC4A8 | -0.192712 | 9.62E-06 | 3.81E-05 |
| GSK3B | -0.1927106 | 9.62E-06 | 3.81E-05 |
| C4orf34 | -0.1926614 | 9.68E-06 | 3.83E-05 |
| JMJD4 | 0.1926543 | 9.68E-06 | 3.83E-05 |
| C10orf2 | 0.1926468 | 9.69E-06 | 3.83E-05 |
| MLST8 | 0.1926424 | 9.70E-06 | 3.83E-05 |
| VPS13C | -0.1925725 | 9.77E-06 | 3.86E-05 |
| C15orf59 | -0.1925599 | 9.78E-06 | 3.86E-05 |
| UROS | 0.1925459 | 9.80E-06 | 3.87E-05 |
| SMYD5 | 0.1925134 | 9.83E-06 | 3.88E-05 |
| KLHL11 | -0.1925096 | 9.84E-06 | 3.88E-05 |
| HOXB2 | -0.1924987 | 9.85E-06 | 3.89E-05 |
| FAM122B | 0.1924952 | 9.85E-06 | 3.89E-05 |
| PLCG2 | -0.1924874 | 9.86E-06 | 3.89E-05 |
| PPL | -0.1924788 | 9.87E-06 | 3.89E-05 |
| MMP9 | -0.1924431 | 9.91E-06 | 3.91E-05 |
| WFDC12 | -0.1924295 | 9.92E-06 | 3.91E-05 |
| GHRLOS | 0.1924209 | 9.93E-06 | 3.92E-05 |
| MYO1B | -0.1923807 | 9.97E-06 | 3.93E-05 |
| FAM49B | 0.1923659 | 9.99E-06 | 3.94E-05 |
| TMEM101 | 0.1923522 | 1.00E-05 | 3.94E-05 |
| COPS5 | 0.1923399 | 1.00E-05 | 3.95E-05 |
| SNHG8 | 0.1923049 | 1.01E-05 | 3.96E-05 |
| CYP46A1 | -0.1922839 | 1.01E-05 | 3.97E-05 |
| SNX12 | -0.1922527 | 1.01E-05 | 3.98E-05 |
| PRX | -0.1922476 | 1.01E-05 | 3.98E-05 |
| SCAMP1 | -0.192245 | 1.01E-05 | 3.98E-05 |
| KLK13 | -0.1922298 | 1.01E-05 | 3.99E-05 |
| CCDC137 | 0.1921988 | 1.02E-05 | 4.00E-05 |
| COQ4 | 0.1921855 | 1.02E-05 | 4.01E-05 |
| FOXD4L6 | 0.192185 | 1.02E-05 | 4.01E-05 |
| MKI67IP | 0.1921842 | 1.02E-05 | 4.01E-05 |
| LGMN | -0.1921447 | 1.02E-05 | 4.02E-05 |
| CEP110 | 0.1921314 | 1.02E-05 | 4.03E-05 |
| ZCCHC8 | 0.1920281 | 1.04E-05 | 4.07E-05 |
| RAPGEF1 | -0.1920248 | 1.04E-05 | 4.07E-05 |
| PEMT | 0.1919842 | 1.04E-05 | 4.09E-05 |
| MAGED2 | -0.1919818 | 1.04E-05 | 4.09E-05 |
| SLC7A6 | -0.1919525 | 1.04E-05 | 4.10E-05 |
| LYRM4 | 0.1919505 | 1.04E-05 | 4.10E-05 |
| ITSN1 | -0.1919481 | 1.05E-05 | 4.10E-05 |
| MAP7D1 | -0.1918972 | 1.05E-05 | 4.12E-05 |
| CCNB1IP1 | 0.1918783 | 1.05E-05 | 4.13E-05 |
| ARHGEF6 | -0.1918038 | 1.06E-05 | 4.16E-05 |
| UEVLD | -0.1918037 | 1.06E-05 | 4.16E-05 |
| C6orf129 | 0.1917155 | 1.07E-05 | 4.20E-05 |
| ABRA | -0.1916878 | 1.07E-05 | 4.21E-05 |
| C20orf111 | 0.1916741 | 1.08E-05 | 4.22E-05 |
| ISLR2 | -0.1916571 | 1.08E-05 | 4.23E-05 |
| C11orf45 | -0.1916533 | 1.08E-05 | 4.23E-05 |
| IGSF10 | -0.191642 | 1.08E-05 | 4.23E-05 |
| PKD1L1 | -0.1916125 | 1.08E-05 | 4.24E-05 |
| GFPT2 | -0.1916104 | 1.08E-05 | 4.24E-05 |
| PLAC8L1 | 0.1916037 | 1.08E-05 | 4.25E-05 |
| WDR12 | 0.1916032 | 1.08E-05 | 4.25E-05 |
| ZNF229 | -0.1916005 | 1.09E-05 | 4.25E-05 |
| USP54 | -0.1916002 | 1.09E-05 | 4.25E-05 |
| RELA | -0.1915644 | 1.09E-05 | 4.26E-05 |
| MLYCD | -0.1915548 | 1.09E-05 | 4.27E-05 |
| FLJ45079 | 0.1915205 | 1.09E-05 | 4.28E-05 |
| SYNPO2 | -0.1915025 | 1.10E-05 | 4.29E-05 |
| APBB1 | -0.1914663 | 1.10E-05 | 4.30E-05 |
| TNFAIP8L3 | -0.1914369 | 1.10E-05 | 4.32E-05 |
| PNMA2 | -0.1914364 | 1.10E-05 | 4.32E-05 |
| NLRP10 | -0.1914179 | 1.11E-05 | 4.32E-05 |
| CCT5 | 0.1914157 | 1.11E-05 | 4.32E-05 |
| IL13RA1 | -0.1914154 | 1.11E-05 | 4.32E-05 |
| FAM179B | -0.1913748 | 1.11E-05 | 4.34E-05 |
| ZFYVE27 | 0.1913619 | 1.11E-05 | 4.35E-05 |
| KCNE2 | 0.1913617 | 1.11E-05 | 4.35E-05 |
| ARMC9 | -0.1913416 | 1.12E-05 | 4.35E-05 |
| C16orf80 | 0.1913414 | 1.12E-05 | 4.35E-05 |
| CNGA3 | -0.1913413 | 1.12E-05 | 4.35E-05 |
| PTGES2 | 0.1913325 | 1.12E-05 | 4.36E-05 |
| FDXR | 0.1913262 | 1.12E-05 | 4.36E-05 |
| EEF1DP3 | -0.1912711 | 1.12E-05 | 4.38E-05 |
| TRIT1 | 0.191254 | 1.13E-05 | 4.39E-05 |
| GTPBP1 | -0.1912346 | 1.13E-05 | 4.40E-05 |
| ZNF37B | 0.1912216 | 1.13E-05 | 4.40E-05 |
| RC3H1 | -0.1912175 | 1.13E-05 | 4.41E-05 |
| RPL39L | 0.1911937 | 1.13E-05 | 4.42E-05 |
| ALPL | -0.1911635 | 1.14E-05 | 4.43E-05 |
| ATP5D | 0.1911594 | 1.14E-05 | 4.43E-05 |
| C1R | -0.1911146 | 1.14E-05 | 4.45E-05 |
| RGPD5 | -0.1911063 | 1.14E-05 | 4.45E-05 |
| RABGEF1 | -0.191092 | 1.15E-05 | 4.46E-05 |
| C2orf56 | 0.1910881 | 1.15E-05 | 4.46E-05 |
| APOL5 | -0.1910753 | 1.15E-05 | 4.47E-05 |
| NPM2 | 0.1910565 | 1.15E-05 | 4.47E-05 |
| PDE5A | -0.1910295 | 1.15E-05 | 4.49E-05 |
| INPP5E | 0.1910146 | 1.16E-05 | 4.49E-05 |
| THAP7 | 0.1910073 | 1.16E-05 | 4.50E-05 |
| PNMAL1 | -0.191002 | 1.16E-05 | 4.50E-05 |
| C20orf117 | -0.1909826 | 1.16E-05 | 4.51E-05 |
| FAM23A | -0.1909574 | 1.16E-05 | 4.52E-05 |
| C9orf78 | 0.190957 | 1.16E-05 | 4.52E-05 |
| LCE2A | -0.1909493 | 1.16E-05 | 4.52E-05 |
| NCRNA00182 | -0.1908936 | 1.17E-05 | 4.55E-05 |
| CCNA1 | -0.190814 | 1.18E-05 | 4.58E-05 |
| CDYL | -0.1907971 | 1.18E-05 | 4.59E-05 |
| ZNF430 | -0.1907966 | 1.18E-05 | 4.59E-05 |
| PDGFC | -0.1907462 | 1.19E-05 | 4.61E-05 |
| IL31RA | -0.1907422 | 1.19E-05 | 4.62E-05 |
| C6orf52 | 0.190731 | 1.19E-05 | 4.62E-05 |
| RAP2A | -0.1907073 | 1.19E-05 | 4.63E-05 |
| ZNF281 | -0.1906872 | 1.20E-05 | 4.64E-05 |
| TRIP6 | 0.1906742 | 1.20E-05 | 4.65E-05 |
| C2orf40 | -0.1906495 | 1.20E-05 | 4.66E-05 |
| PCYT1B | -0.1906127 | 1.21E-05 | 4.67E-05 |
| PTK2 | -0.1905965 | 1.21E-05 | 4.68E-05 |
| IBTK | -0.1905933 | 1.21E-05 | 4.68E-05 |
| STXBP1 | -0.1905824 | 1.21E-05 | 4.69E-05 |
| CCKAR | -0.190525 | 1.22E-05 | 4.72E-05 |
| LRBA | -0.1904982 | 1.22E-05 | 4.73E-05 |
| GRTP1 | 0.1904654 | 1.23E-05 | 4.74E-05 |
| MAGED1 | -0.1904429 | 1.23E-05 | 4.75E-05 |
| TMEM133 | -0.1904412 | 1.23E-05 | 4.75E-05 |
| TRPC1 | -0.1904399 | 1.23E-05 | 4.75E-05 |
| KCNK6 | -0.1904329 | 1.23E-05 | 4.76E-05 |
| LHX8 | -0.1903946 | 1.24E-05 | 4.78E-05 |
| C6orf27 | -0.1903733 | 1.24E-05 | 4.79E-05 |
| RHBDF2 | -0.1903555 | 1.24E-05 | 4.79E-05 |
| SRRM3 | -0.1903403 | 1.24E-05 | 4.80E-05 |
| ZDHHC8 | -0.1903391 | 1.24E-05 | 4.80E-05 |
| CA14 | 0.1903222 | 1.24E-05 | 4.81E-05 |
| SDC1 | -0.1903083 | 1.25E-05 | 4.81E-05 |
| YARS2 | 0.1903041 | 1.25E-05 | 4.82E-05 |
| PYGO1 | -0.190296 | 1.25E-05 | 4.82E-05 |
| KCNJ12 | -0.1902783 | 1.25E-05 | 4.83E-05 |
| VCPIP1 | -0.1902434 | 1.26E-05 | 4.84E-05 |
| CHCHD5 | 0.1902315 | 1.26E-05 | 4.85E-05 |
| RET | -0.1902209 | 1.26E-05 | 4.85E-05 |
| P2RY2 | -0.1902107 | 1.26E-05 | 4.86E-05 |
| MRPL33 | 0.1902091 | 1.26E-05 | 4.86E-05 |
| CASK | -0.1901876 | 1.26E-05 | 4.87E-05 |
| TPSB2 | -0.1901751 | 1.26E-05 | 4.87E-05 |
| RRP7A | 0.1901448 | 1.27E-05 | 4.89E-05 |
| FOLR1 | -0.1901297 | 1.27E-05 | 4.90E-05 |
| ZNF776 | -0.1900935 | 1.28E-05 | 4.91E-05 |
| TNRC6A | -0.1900485 | 1.28E-05 | 4.94E-05 |
| RNF39 | -0.1900265 | 1.28E-05 | 4.95E-05 |
| GPR88 | -0.1900017 | 1.29E-05 | 4.96E-05 |
| DNAJC2 | 0.189995 | 1.29E-05 | 4.96E-05 |
| SLCO5A1 | -0.1899506 | 1.30E-05 | 4.98E-05 |
| MLL5 | -0.1899471 | 1.30E-05 | 4.99E-05 |
| COX4NB | 0.1898888 | 1.30E-05 | 5.02E-05 |
| WRB | 0.189868 | 1.31E-05 | 5.03E-05 |
| CCDC43 | 0.1898572 | 1.31E-05 | 5.03E-05 |
| OR4D9 | 0.1898544 | 1.31E-05 | 5.03E-05 |
| BPGM | -0.1898119 | 1.31E-05 | 5.05E-05 |
| PSMD10 | 0.1898114 | 1.31E-05 | 5.05E-05 |
| SDHAP1 | 0.1898109 | 1.31E-05 | 5.05E-05 |
| C10orf128 | -0.1897491 | 1.32E-05 | 5.08E-05 |
| MYBPC2 | -0.1897156 | 1.33E-05 | 5.10E-05 |
| UBXN7 | -0.1897128 | 1.33E-05 | 5.10E-05 |
| RLN2 | 0.1897061 | 1.33E-05 | 5.11E-05 |
| TNFAIP1 | -0.189701 | 1.33E-05 | 5.11E-05 |
| L3MBTL3 | -0.1896207 | 1.34E-05 | 5.15E-05 |
| TUBB8 | 0.1895996 | 1.34E-05 | 5.16E-05 |
| ITGB1BP3 | -0.1895936 | 1.35E-05 | 5.16E-05 |
| SHF | -0.1895828 | 1.35E-05 | 5.17E-05 |
| SLC2A5 | -0.1895686 | 1.35E-05 | 5.17E-05 |
| GPR115 | -0.1895645 | 1.35E-05 | 5.18E-05 |
| ZBTB16 | -0.1895452 | 1.35E-05 | 5.19E-05 |
| ARL11 | -0.1895323 | 1.35E-05 | 5.19E-05 |
| TMEM186 | 0.1895281 | 1.36E-05 | 5.19E-05 |
| PCDHGA8 | -0.1895216 | 1.36E-05 | 5.20E-05 |
| CNTN1 | -0.1895151 | 1.36E-05 | 5.20E-05 |
| EPN2 | -0.1894652 | 1.36E-05 | 5.22E-05 |
| HAUS3 | 0.1894435 | 1.37E-05 | 5.24E-05 |
| KRT8 | 0.1894247 | 1.37E-05 | 5.25E-05 |
| LGI1 | -0.1894137 | 1.37E-05 | 5.25E-05 |
| TNRC18 | -0.1893821 | 1.38E-05 | 5.27E-05 |
| AGBL1 | -0.1893798 | 1.38E-05 | 5.27E-05 |
| C15orf56 | 0.1893766 | 1.38E-05 | 5.27E-05 |
| PIK3R2 | -0.1893648 | 1.38E-05 | 5.27E-05 |
| HEPHL1 | -0.1893504 | 1.38E-05 | 5.28E-05 |
| CCDC21 | 0.189311 | 1.39E-05 | 5.30E-05 |
| C12orf57 | 0.1892388 | 1.40E-05 | 5.34E-05 |
| RNASE7 | -0.1892347 | 1.40E-05 | 5.34E-05 |
| BUD13 | 0.1892008 | 1.40E-05 | 5.36E-05 |
| TAF10 | 0.1891843 | 1.41E-05 | 5.37E-05 |
| PMCH | 0.1891021 | 1.42E-05 | 5.42E-05 |
| CCDC7 | 0.1890946 | 1.42E-05 | 5.42E-05 |
| SETX | -0.1890936 | 1.42E-05 | 5.42E-05 |
| EHMT2 | 0.1890777 | 1.42E-05 | 5.43E-05 |
| C7orf55 | 0.1890451 | 1.43E-05 | 5.45E-05 |
| FADS6 | -0.1890369 | 1.43E-05 | 5.45E-05 |
| FAIM | 0.1890242 | 1.43E-05 | 5.46E-05 |
| ULBP3 | -0.188994 | 1.43E-05 | 5.47E-05 |
| CATSPERB | -0.1889835 | 1.44E-05 | 5.48E-05 |
| ZNF788 | -0.1889516 | 1.44E-05 | 5.49E-05 |
| MFSD11 | -0.1889378 | 1.44E-05 | 5.50E-05 |
| SNRNP35 | 0.1889378 | 1.44E-05 | 5.50E-05 |
| MTMR7 | 0.1889205 | 1.45E-05 | 5.51E-05 |
| C5orf45 | 0.1889198 | 1.45E-05 | 5.51E-05 |
| TGFBI | -0.188865 | 1.45E-05 | 5.54E-05 |
| CH25H | -0.1888646 | 1.45E-05 | 5.54E-05 |
| BASP1 | -0.1888557 | 1.46E-05 | 5.54E-05 |
| PPARD | -0.1888165 | 1.46E-05 | 5.57E-05 |
| JUP | -0.1888102 | 1.46E-05 | 5.57E-05 |
| RIBC1 | 0.1887556 | 1.47E-05 | 5.60E-05 |
| LOC653113 | 0.1887478 | 1.47E-05 | 5.60E-05 |
| ATP10B | -0.1887446 | 1.47E-05 | 5.60E-05 |
| DAB1 | -0.1887438 | 1.47E-05 | 5.60E-05 |
| PTENP1 | -0.1887198 | 1.48E-05 | 5.62E-05 |
| KIAA1826 | -0.1887007 | 1.48E-05 | 5.63E-05 |
| POU2F3 | -0.1886813 | 1.48E-05 | 5.64E-05 |
| STK10 | -0.1886591 | 1.49E-05 | 5.65E-05 |
| ARL6IP6 | 0.1886368 | 1.49E-05 | 5.66E-05 |
| CXXC1 | 0.188636 | 1.49E-05 | 5.66E-05 |
| SLC35D1 | -0.1886171 | 1.49E-05 | 5.67E-05 |
| FBXO24 | 0.1885737 | 1.50E-05 | 5.70E-05 |
| TPST1 | -0.1885665 | 1.50E-05 | 5.70E-05 |
| MFSD1 | -0.1885635 | 1.50E-05 | 5.70E-05 |
| CITED2 | -0.1885533 | 1.50E-05 | 5.71E-05 |
| CA3 | -0.1885284 | 1.51E-05 | 5.72E-05 |
| TLR4 | -0.1885243 | 1.51E-05 | 5.72E-05 |
| AURKAIP1 | 0.1885186 | 1.51E-05 | 5.72E-05 |
| BCAS4 | 0.1885059 | 1.51E-05 | 5.73E-05 |
| ZNF25 | -0.1884947 | 1.51E-05 | 5.74E-05 |
| GTPBP8 | 0.1884582 | 1.52E-05 | 5.76E-05 |
| NT5M | 0.1884467 | 1.52E-05 | 5.76E-05 |
| MCPH1 | 0.1884378 | 1.52E-05 | 5.77E-05 |
| ELMO1 | -0.1884279 | 1.52E-05 | 5.77E-05 |
| C7orf42 | -0.1883992 | 1.53E-05 | 5.79E-05 |
| CNTLN | 0.1883687 | 1.53E-05 | 5.81E-05 |
| SLC39A14 | -0.1883686 | 1.53E-05 | 5.81E-05 |
| C2orf28 | 0.1883659 | 1.53E-05 | 5.81E-05 |
| FOLR2 | -0.1883649 | 1.53E-05 | 5.81E-05 |
| 2-Mar | -0.1883476 | 1.54E-05 | 5.82E-05 |
| LOXL4 | -0.188346 | 1.54E-05 | 5.82E-05 |
| ZMYM2 | -0.1883118 | 1.54E-05 | 5.84E-05 |
| KIN | 0.1882918 | 1.55E-05 | 5.85E-05 |
| BCL9 | -0.1882546 | 1.55E-05 | 5.87E-05 |
| AMPD1 | -0.1882519 | 1.55E-05 | 5.87E-05 |
| C3orf74 | -0.1881976 | 1.56E-05 | 5.90E-05 |
| WHAMM | 0.1881349 | 1.57E-05 | 5.94E-05 |
| OSTM1 | -0.1880987 | 1.58E-05 | 5.96E-05 |
| ZNF330 | 0.188098 | 1.58E-05 | 5.96E-05 |
| LOC400657 | 0.1880184 | 1.59E-05 | 6.01E-05 |
| SPRR1B | -0.1879842 | 1.60E-05 | 6.03E-05 |
| CD1E | -0.1879816 | 1.60E-05 | 6.03E-05 |
| SMC6 | 0.1879698 | 1.60E-05 | 6.04E-05 |
| LOR | -0.1879663 | 1.60E-05 | 6.04E-05 |
| PFKFB3 | -0.1879396 | 1.60E-05 | 6.06E-05 |
| GJB2 | -0.1879022 | 1.61E-05 | 6.08E-05 |
| TH1L | 0.1879004 | 1.61E-05 | 6.08E-05 |
| NOVA2 | -0.187897 | 1.61E-05 | 6.08E-05 |
| TWIST1 | -0.1878922 | 1.61E-05 | 6.08E-05 |
| HPSE2 | -0.1878912 | 1.61E-05 | 6.08E-05 |
| BBS9 | -0.1878567 | 1.62E-05 | 6.10E-05 |
| C21orf7 | -0.1878552 | 1.62E-05 | 6.10E-05 |
| RNF181 | 0.1878534 | 1.62E-05 | 6.10E-05 |
| YWHAE | 0.1878329 | 1.62E-05 | 6.11E-05 |
| CYP8B1 | -0.1877882 | 1.63E-05 | 6.14E-05 |
| S100A1 | -0.1877783 | 1.63E-05 | 6.15E-05 |
| CACNA1E | -0.1877599 | 1.64E-05 | 6.16E-05 |
| DENND2A | -0.1877563 | 1.64E-05 | 6.16E-05 |
| S100A7 | -0.1877422 | 1.64E-05 | 6.17E-05 |
| C1orf203 | 0.1877263 | 1.64E-05 | 6.18E-05 |
| PGAM5 | 0.1877196 | 1.64E-05 | 6.18E-05 |
| TTBK2 | -0.187704 | 1.64E-05 | 6.19E-05 |
| SLC25A15 | 0.1877007 | 1.65E-05 | 6.19E-05 |
| LASS6 | -0.1876992 | 1.65E-05 | 6.19E-05 |
| HEMK1 | 0.1876792 | 1.65E-05 | 6.20E-05 |
| IPP | -0.1876676 | 1.65E-05 | 6.21E-05 |
| HOMER1 | -0.1876542 | 1.65E-05 | 6.22E-05 |
| ALKBH6 | 0.1875982 | 1.66E-05 | 6.25E-05 |
| TTLL7 | -0.1875937 | 1.66E-05 | 6.25E-05 |
| QPCTL | 0.1875762 | 1.67E-05 | 6.26E-05 |
| NHEDC2 | -0.1875664 | 1.67E-05 | 6.27E-05 |
| PAFAH2 | -0.1875249 | 1.68E-05 | 6.29E-05 |
| C9orf119 | 0.1875234 | 1.68E-05 | 6.29E-05 |
| ATF4 | 0.1874982 | 1.68E-05 | 6.31E-05 |
| KIAA1671 | -0.1874973 | 1.68E-05 | 6.31E-05 |
| COBLL1 | -0.1873432 | 1.71E-05 | 6.41E-05 |
| KIAA0114 | 0.1873369 | 1.71E-05 | 6.41E-05 |
| SPIRE2 | 0.1872982 | 1.72E-05 | 6.44E-05 |
| KRBA2 | -0.1872971 | 1.72E-05 | 6.44E-05 |
| PCDHGC4 | -0.1872879 | 1.72E-05 | 6.44E-05 |
| PLXNB2 | -0.1872608 | 1.72E-05 | 6.46E-05 |
| CXCL12 | -0.187251 | 1.73E-05 | 6.47E-05 |
| CDK3 | 0.1872013 | 1.73E-05 | 6.50E-05 |
| MAP3K12 | -0.1871932 | 1.74E-05 | 6.50E-05 |
| SOSTDC1 | -0.1871448 | 1.74E-05 | 6.54E-05 |
| CD1C | -0.187131 | 1.75E-05 | 6.54E-05 |
| ADPRHL2 | 0.1871191 | 1.75E-05 | 6.55E-05 |
| ALS2 | -0.1871007 | 1.75E-05 | 6.56E-05 |
| CRKL | -0.1870668 | 1.76E-05 | 6.59E-05 |
| FCGR2C | -0.187065 | 1.76E-05 | 6.59E-05 |
| ZBTB8B | 0.1870547 | 1.76E-05 | 6.59E-05 |
| ADRM1 | 0.1870412 | 1.76E-05 | 6.60E-05 |
| RBBP8 | 0.1869771 | 1.78E-05 | 6.64E-05 |
| TIMP4 | -0.1869535 | 1.78E-05 | 6.66E-05 |
| ADPRHL1 | -0.186945 | 1.78E-05 | 6.66E-05 |
| ERBB4 | -0.1869295 | 1.78E-05 | 6.67E-05 |
| OPN4 | -0.1869257 | 1.79E-05 | 6.67E-05 |
| TCEB2 | 0.1869178 | 1.79E-05 | 6.68E-05 |
| TAS2R20 | 0.1869136 | 1.79E-05 | 6.68E-05 |
| GALE | 0.1869048 | 1.79E-05 | 6.68E-05 |
| NDUFB10 | 0.1869041 | 1.79E-05 | 6.68E-05 |
| TRNT1 | 0.1868897 | 1.79E-05 | 6.69E-05 |
| RAPH1 | -0.1868831 | 1.79E-05 | 6.70E-05 |
| RUSC1 | 0.1868814 | 1.79E-05 | 6.70E-05 |
| GPATCH8 | -0.1868582 | 1.80E-05 | 6.71E-05 |
| B3GNTL1 | 0.1868311 | 1.80E-05 | 6.73E-05 |
| GOLGA7B | -0.1867973 | 1.81E-05 | 6.75E-05 |
| ZNF616 | -0.1867717 | 1.81E-05 | 6.77E-05 |
| ANGPT2 | -0.1867586 | 1.82E-05 | 6.78E-05 |
| UBQLN2 | -0.1867457 | 1.82E-05 | 6.79E-05 |
| ARHGEF37 | -0.1866675 | 1.83E-05 | 6.84E-05 |
| PPEF1 | -0.1866388 | 1.84E-05 | 6.86E-05 |
| FKBPL | 0.1866384 | 1.84E-05 | 6.86E-05 |
| C14orf49 | -0.1865803 | 1.85E-05 | 6.90E-05 |
| MPRIP | -0.186571 | 1.85E-05 | 6.90E-05 |
| CTNNB1 | -0.186571 | 1.85E-05 | 6.90E-05 |
| TK2 | -0.1865636 | 1.85E-05 | 6.91E-05 |
| LOC134466 | -0.1865631 | 1.85E-05 | 6.91E-05 |
| C20orf20 | 0.1865503 | 1.86E-05 | 6.91E-05 |
| UBXN8 | 0.1865435 | 1.86E-05 | 6.92E-05 |
| LOH3CR2A | -0.1863587 | 1.89E-05 | 7.05E-05 |
| KPNA3 | -0.1863558 | 1.90E-05 | 7.05E-05 |
| HTR7 | -0.1863483 | 1.90E-05 | 7.06E-05 |
| MIR155HG | 0.1863443 | 1.90E-05 | 7.06E-05 |
| SHE | -0.186329 | 1.90E-05 | 7.07E-05 |
| PAQR7 | -0.1863176 | 1.90E-05 | 7.08E-05 |
| CYP2D7P1 | 0.1863122 | 1.90E-05 | 7.08E-05 |
| PLA2G4E | -0.1863059 | 1.91E-05 | 7.08E-05 |
| NPAS3 | -0.1862151 | 1.92E-05 | 7.15E-05 |
| ABCB7 | 0.1861888 | 1.93E-05 | 7.17E-05 |
| PLD3 | -0.1861757 | 1.93E-05 | 7.18E-05 |
| CUTC | 0.1861622 | 1.93E-05 | 7.18E-05 |
| UBE2A | 0.1861419 | 1.94E-05 | 7.20E-05 |
| ARHGEF16 | 0.1860902 | 1.95E-05 | 7.24E-05 |
| RGS3 | -0.1860735 | 1.95E-05 | 7.25E-05 |
| FAR1 | 0.1860264 | 1.96E-05 | 7.28E-05 |
| AKR1B15 | -0.1860115 | 1.97E-05 | 7.29E-05 |
| KIAA0467 | -0.1859907 | 1.97E-05 | 7.31E-05 |
| C5orf54 | 0.1859518 | 1.98E-05 | 7.33E-05 |
| CASQ1 | -0.1859144 | 1.99E-05 | 7.36E-05 |
| COX7A1 | -0.1859062 | 1.99E-05 | 7.37E-05 |
| TNFRSF11B | -0.1858766 | 1.99E-05 | 7.39E-05 |
| FAM101A | -0.1858745 | 1.99E-05 | 7.39E-05 |
| FAM35B | 0.1858735 | 1.99E-05 | 7.39E-05 |
| NCOR1 | -0.1858289 | 2.00E-05 | 7.42E-05 |
| ALG10 | 0.1858162 | 2.01E-05 | 7.43E-05 |
| C20orf24 | 0.1857989 | 2.01E-05 | 7.44E-05 |
| FAM100B | 0.1857686 | 2.02E-05 | 7.46E-05 |
| CEP350 | -0.1857634 | 2.02E-05 | 7.47E-05 |
| SHOC2 | -0.1857614 | 2.02E-05 | 7.47E-05 |
| PREP | -0.1857562 | 2.02E-05 | 7.47E-05 |
| CLCN5 | -0.1857554 | 2.02E-05 | 7.47E-05 |
| PDE4DIP | -0.1857427 | 2.02E-05 | 7.48E-05 |
| NFATC1 | -0.1857375 | 2.02E-05 | 7.48E-05 |
| VPS45 | 0.1857224 | 2.03E-05 | 7.49E-05 |
| TRIM13 | -0.1857167 | 2.03E-05 | 7.49E-05 |
| PSG4 | -0.1856768 | 2.04E-05 | 7.52E-05 |
| ZBTB22 | -0.1856717 | 2.04E-05 | 7.53E-05 |
| STAT5B | -0.1856712 | 2.04E-05 | 7.53E-05 |
| TXN | 0.1855965 | 2.05E-05 | 7.58E-05 |
| DYNC1I1 | -0.1855945 | 2.05E-05 | 7.58E-05 |
| SRD5A2 | -0.1855934 | 2.05E-05 | 7.58E-05 |
| TMEM205 | 0.1855392 | 2.06E-05 | 7.62E-05 |
| DCP2 | 0.185531 | 2.07E-05 | 7.63E-05 |
| TM9SF3 | -0.1855256 | 2.07E-05 | 7.63E-05 |
| TMEM70 | 0.1855233 | 2.07E-05 | 7.63E-05 |
| TBC1D23 | -0.1854768 | 2.08E-05 | 7.67E-05 |
| TMEM184C | -0.1854574 | 2.08E-05 | 7.68E-05 |
| BZW2 | 0.1854495 | 2.08E-05 | 7.69E-05 |
| WNT9B | -0.1854316 | 2.09E-05 | 7.70E-05 |
| CDH24 | 0.1854248 | 2.09E-05 | 7.70E-05 |
| RCOR1 | -0.1853744 | 2.10E-05 | 7.74E-05 |
| TMEM132D | -0.1853428 | 2.11E-05 | 7.77E-05 |
| ABCA4 | -0.1853422 | 2.11E-05 | 7.77E-05 |
| LCE2D | -0.185335 | 2.11E-05 | 7.77E-05 |
| SMAP1 | -0.1853347 | 2.11E-05 | 7.77E-05 |
| C19orf24 | 0.1853296 | 2.11E-05 | 7.77E-05 |
| FRMD4B | -0.185294 | 2.12E-05 | 7.80E-05 |
| PDLIM3 | -0.1852592 | 2.13E-05 | 7.83E-05 |
| SCO2 | 0.185226 | 2.13E-05 | 7.85E-05 |
| PHACTR2 | -0.185207 | 2.14E-05 | 7.87E-05 |
| TMCO4 | 0.1851871 | 2.14E-05 | 7.88E-05 |
| ZDHHC16 | 0.1851862 | 2.14E-05 | 7.88E-05 |
| CEACAM19 | -0.1851705 | 2.15E-05 | 7.89E-05 |
| PCDHA7 | -0.1851186 | 2.16E-05 | 7.93E-05 |
| SPAG17 | -0.1850872 | 2.16E-05 | 7.96E-05 |
| LASS1 | -0.1850395 | 2.18E-05 | 8.00E-05 |
| TEX19 | 0.1850191 | 2.18E-05 | 8.01E-05 |
| OSBPL8 | -0.1850156 | 2.18E-05 | 8.01E-05 |
| RPS6KA1 | 0.1850086 | 2.18E-05 | 8.02E-05 |
| PHF20L1 | -0.1849743 | 2.19E-05 | 8.05E-05 |
| NPHS1 | 0.1849679 | 2.19E-05 | 8.05E-05 |
| ELF1 | -0.1849077 | 2.21E-05 | 8.10E-05 |
| POLR1D | 0.1848894 | 2.21E-05 | 8.11E-05 |
| ACAT2 | 0.1848788 | 2.21E-05 | 8.12E-05 |
| YIPF6 | -0.1848774 | 2.21E-05 | 8.12E-05 |
| GUSB | 0.1847363 | 2.24E-05 | 8.24E-05 |
| TMEM199 | 0.1847346 | 2.25E-05 | 8.24E-05 |
| C9orf102 | -0.1847286 | 2.25E-05 | 8.24E-05 |
| LOC388955 | 0.1847065 | 2.25E-05 | 8.26E-05 |
| OR8S1 | 0.1846936 | 2.25E-05 | 8.27E-05 |
| PSG5 | -0.1846745 | 2.26E-05 | 8.28E-05 |
| PSD | -0.1846598 | 2.26E-05 | 8.30E-05 |
| IGSF11 | -0.1846064 | 2.28E-05 | 8.34E-05 |
| MYL1 | -0.1845926 | 2.28E-05 | 8.35E-05 |
| RASSF3 | -0.1845717 | 2.28E-05 | 8.37E-05 |
| SCUBE2 | -0.1845613 | 2.29E-05 | 8.37E-05 |
| PRB2 | -0.1845448 | 2.29E-05 | 8.39E-05 |
| NDUFB2 | 0.1844655 | 2.31E-05 | 8.46E-05 |
| SUV420H1 | -0.1844628 | 2.31E-05 | 8.46E-05 |
| BRCA2 | 0.184446 | 2.31E-05 | 8.47E-05 |
| C1QTNF1 | -0.1844437 | 2.31E-05 | 8.47E-05 |
| ISOC2 | 0.1844342 | 2.32E-05 | 8.48E-05 |
| REEP1 | -0.1844151 | 2.32E-05 | 8.49E-05 |
| STAT3 | -0.18439 | 2.33E-05 | 8.51E-05 |
| EXOC3L2 | -0.1843414 | 2.34E-05 | 8.55E-05 |
| PDCD2 | 0.1843302 | 2.34E-05 | 8.56E-05 |
| ADRBK2 | -0.1843288 | 2.34E-05 | 8.56E-05 |
| SNX13 | -0.1843242 | 2.34E-05 | 8.56E-05 |
| EXD1 | 0.1842891 | 2.35E-05 | 8.59E-05 |
| KLHL29 | -0.1842812 | 2.35E-05 | 8.60E-05 |
| IL28B | 0.1842302 | 2.37E-05 | 8.64E-05 |
| GNB1 | -0.184223 | 2.37E-05 | 8.65E-05 |
| IPW | -0.1842195 | 2.37E-05 | 8.65E-05 |
| HIST1H4D | 0.1842011 | 2.37E-05 | 8.67E-05 |
| DERL2 | 0.1841915 | 2.38E-05 | 8.67E-05 |
| SIN3B | -0.1841903 | 2.38E-05 | 8.67E-05 |
| C14orf43 | -0.184163 | 2.38E-05 | 8.69E-05 |
| SUPT5H | -0.1841623 | 2.38E-05 | 8.69E-05 |
| NTF3 | -0.1841217 | 2.39E-05 | 8.73E-05 |
| VRK2 | 0.184105 | 2.40E-05 | 8.74E-05 |
| SLC5A6 | 0.1841014 | 2.40E-05 | 8.74E-05 |
| AQP10 | -0.1840781 | 2.40E-05 | 8.76E-05 |
| PORCN | -0.184078 | 2.40E-05 | 8.76E-05 |
| ARHGAP24 | -0.1839852 | 2.43E-05 | 8.85E-05 |
| C19orf54 | 0.1839822 | 2.43E-05 | 8.85E-05 |
| MAP2K4 | -0.1839738 | 2.43E-05 | 8.85E-05 |
| TELO2 | 0.1839687 | 2.43E-05 | 8.86E-05 |
| SLC8A1 | -0.1839635 | 2.43E-05 | 8.86E-05 |
| SETD1B | -0.1839224 | 2.44E-05 | 8.90E-05 |
| C17orf64 | 0.1839086 | 2.45E-05 | 8.91E-05 |
| GOLIM4 | -0.1838877 | 2.45E-05 | 8.92E-05 |
| TBC1D19 | -0.1838844 | 2.45E-05 | 8.93E-05 |
| ZNF101 | 0.1838706 | 2.46E-05 | 8.94E-05 |
| SERAC1 | -0.1838642 | 2.46E-05 | 8.94E-05 |
| SPRED2 | -0.1838079 | 2.47E-05 | 8.99E-05 |
| BVES | -0.1837964 | 2.47E-05 | 9.00E-05 |
| PPIC | -0.1837742 | 2.48E-05 | 9.02E-05 |
| HK2 | -0.1837264 | 2.49E-05 | 9.06E-05 |
| KRT9 | -0.1836606 | 2.51E-05 | 9.12E-05 |
| SBNO1 | -0.1836379 | 2.52E-05 | 9.14E-05 |
| LRRIQ3 | 0.1835634 | 2.54E-05 | 9.21E-05 |
| TSPAN2 | -0.1835604 | 2.54E-05 | 9.21E-05 |
| KIAA0652 | -0.1835514 | 2.54E-05 | 9.22E-05 |
| WIPF2 | -0.1835168 | 2.55E-05 | 9.25E-05 |
| CD84 | -0.1835035 | 2.55E-05 | 9.26E-05 |
| ELL | -0.183502 | 2.55E-05 | 9.26E-05 |
| RGPD1 | -0.1834901 | 2.55E-05 | 9.27E-05 |
| MTSS1L | -0.1834713 | 2.56E-05 | 9.29E-05 |
| PACS2 | -0.1834662 | 2.56E-05 | 9.29E-05 |
| PIP4K2C | -0.1834305 | 2.57E-05 | 9.32E-05 |
| RAP1GAP | -0.1834262 | 2.57E-05 | 9.33E-05 |
| INPP5B | 0.1834173 | 2.57E-05 | 9.33E-05 |
| CHD4 | -0.1833703 | 2.59E-05 | 9.38E-05 |
| LOX | -0.1833611 | 2.59E-05 | 9.38E-05 |
| PDPR | -0.183359 | 2.59E-05 | 9.38E-05 |
| BFSP1 | -0.1833415 | 2.59E-05 | 9.40E-05 |
| ANGPTL1 | -0.1833354 | 2.60E-05 | 9.40E-05 |
| DNAH10 | -0.1833278 | 2.60E-05 | 9.41E-05 |
| C12orf70 | 0.1832424 | 2.62E-05 | 9.49E-05 |
| SLC28A3 | -0.1831955 | 2.63E-05 | 9.54E-05 |
| SLC35F1 | -0.1831791 | 2.64E-05 | 9.55E-05 |
| BHLHE41 | -0.1831653 | 2.64E-05 | 9.56E-05 |
| POU3F1 | -0.1831651 | 2.64E-05 | 9.56E-05 |
| NDUFS8 | 0.1831647 | 2.64E-05 | 9.56E-05 |
| KIAA0892 | -0.1831372 | 2.65E-05 | 9.58E-05 |
| LUC7L | 0.1830841 | 2.66E-05 | 9.64E-05 |
| TNFSF11 | -0.1830812 | 2.66E-05 | 9.64E-05 |
| STEAP4 | -0.183081 | 2.66E-05 | 9.64E-05 |
| TDRG1 | 0.1830021 | 2.69E-05 | 9.71E-05 |
| ZNF528 | -0.1829929 | 2.69E-05 | 9.72E-05 |
| TGIF2 | 0.1829761 | 2.69E-05 | 9.73E-05 |
| ZNF254 | -0.1828859 | 2.72E-05 | 9.82E-05 |
| 10-Sep | -0.1828731 | 2.72E-05 | 9.84E-05 |
| PTPN1 | -0.1828606 | 2.73E-05 | 9.85E-05 |
| DBR1 | 0.1828425 | 2.73E-05 | 9.86E-05 |
| MAP9 | -0.1828267 | 2.74E-05 | 9.88E-05 |
| LOXHD1 | -0.1828187 | 2.74E-05 | 9.88E-05 |
| GDF1 | -0.1827765 | 2.75E-05 | 9.92E-05 |
| C7orf50 | 0.1827552 | 2.76E-05 | 9.94E-05 |
| CLCN7 | -0.1827498 | 2.76E-05 | 9.95E-05 |
| SLC25A39 | 0.1827293 | 2.76E-05 | 9.97E-05 |
| ATP1B2 | -0.1826873 | 2.77E-05 | 0.00010009 |
| STOX2 | -0.1826826 | 2.78E-05 | 0.00010012 |
| CCBL2 | 0.1826782 | 2.78E-05 | 0.00010015 |
| FAM186A | 0.1826745 | 2.78E-05 | 0.00010017 |
| LOC80154 | 0.18267 | 2.78E-05 | 0.0001002 |
| C16orf91 | 0.1826155 | 2.80E-05 | 0.00010074 |
| CYTL1 | -0.1825948 | 2.80E-05 | 0.00010094 |
| BRWD3 | -0.1825688 | 2.81E-05 | 0.00010119 |
| MRGPRE | -0.1825451 | 2.82E-05 | 0.00010142 |
| DEGS1 | -0.1825289 | 2.82E-05 | 0.00010157 |
| SRRM2 | -0.1824959 | 2.83E-05 | 0.0001019 |
| WDR44 | -0.182478 | 2.84E-05 | 0.00010206 |
| ARPC4 | 0.1823906 | 2.86E-05 | 0.00010297 |
| PNPT1 | 0.182379 | 2.86E-05 | 0.00010307 |
| MEI1 | 0.1823569 | 2.87E-05 | 0.00010329 |
| SNX27 | -0.1823376 | 2.88E-05 | 0.00010347 |
| ALDH16A1 | 0.1822939 | 2.89E-05 | 0.00010391 |
| C1orf105 | -0.182293 | 2.89E-05 | 0.00010391 |
| LHX5 | -0.1822868 | 2.89E-05 | 0.00010396 |
| C2orf47 | 0.1822782 | 2.89E-05 | 0.00010403 |
| KCNJ5 | -0.1822399 | 2.91E-05 | 0.00010442 |
| PIGW | 0.1822339 | 2.91E-05 | 0.00010447 |
| C17orf90 | 0.1821636 | 2.93E-05 | 0.00010521 |
| GNA11 | -0.1821456 | 2.93E-05 | 0.00010536 |
| HIST1H1D | 0.1821453 | 2.93E-05 | 0.00010536 |
| KIAA1549 | -0.1821445 | 2.93E-05 | 0.00010536 |
| LOC440356 | 0.1821125 | 2.94E-05 | 0.00010568 |
| FAM55C | -0.1821109 | 2.94E-05 | 0.00010568 |
| KCNN3 | -0.1821007 | 2.95E-05 | 0.00010577 |
| TMEM184A | -0.1820892 | 2.95E-05 | 0.00010587 |
| MMRN1 | -0.1820886 | 2.95E-05 | 0.00010587 |
| PMS2L1 | 0.1820636 | 2.96E-05 | 0.00010612 |
| DDTL | 0.1820038 | 2.98E-05 | 0.00010675 |
| LTBP4 | -0.1819885 | 2.98E-05 | 0.0001069 |
| GOLGA8A | 0.1819441 | 2.99E-05 | 0.00010737 |
| SRR | 0.181854 | 3.02E-05 | 0.00010835 |
| EFHD1 | -0.1818475 | 3.02E-05 | 0.0001084 |
| TOMM20 | 0.1818421 | 3.03E-05 | 0.00010844 |
| DGKZ | 0.1818355 | 3.03E-05 | 0.00010849 |
| CREG2 | -0.1818333 | 3.03E-05 | 0.0001085 |
| SPATA24 | 0.1818084 | 3.04E-05 | 0.00010876 |
| FAM155A | -0.181793 | 3.04E-05 | 0.00010891 |
| SIRT6 | 0.1817861 | 3.04E-05 | 0.00010897 |
| CELF2 | -0.1817769 | 3.05E-05 | 0.00010905 |
| MRPS17 | 0.1817217 | 3.06E-05 | 0.00010965 |
| NTNG2 | -0.1817134 | 3.07E-05 | 0.00010972 |
| NFE2 | -0.1817081 | 3.07E-05 | 0.00010976 |
| HUWE1 | -0.1816965 | 3.07E-05 | 0.00010987 |
| DOCK4 | -0.1816655 | 3.08E-05 | 0.0001102 |
| TNNC2 | -0.1816544 | 3.08E-05 | 0.00011031 |
| LGR5 | -0.1816464 | 3.09E-05 | 0.00011038 |
| RPL23P8 | 0.1816443 | 3.09E-05 | 0.00011038 |
| C5orf46 | -0.1816296 | 3.09E-05 | 0.00011051 |
| UBXN10 | -0.1816294 | 3.09E-05 | 0.00011051 |
| CXCR2 | -0.1816251 | 3.09E-05 | 0.00011054 |
| GLT1D1 | -0.181615 | 3.10E-05 | 0.00011064 |
| TMEM170A | 0.1815882 | 3.11E-05 | 0.00011092 |
| DNTTIP2 | 0.181537 | 3.12E-05 | 0.00011148 |
| FAM43A | -0.1815321 | 3.12E-05 | 0.00011152 |
| ATRNL1 | -0.1815194 | 3.13E-05 | 0.00011164 |
| TMX2 | 0.1815179 | 3.13E-05 | 0.00011164 |
| THSD1 | -0.1815114 | 3.13E-05 | 0.0001117 |
| TRAM2 | -0.1814755 | 3.14E-05 | 0.00011209 |
| RPL19P12 | 0.1814686 | 3.14E-05 | 0.00011215 |
| YPEL5 | -0.1814621 | 3.15E-05 | 0.0001122 |
| TFCP2L1 | -0.181437 | 3.15E-05 | 0.00011247 |
| TEX10 | 0.1814048 | 3.16E-05 | 0.00011282 |
| CDC42EP3 | -0.1813748 | 3.17E-05 | 0.00011315 |
| DSC3 | -0.1813724 | 3.18E-05 | 0.00011315 |
| LOC143666 | 0.1813612 | 3.18E-05 | 0.00011326 |
| EML6 | -0.1813493 | 3.18E-05 | 0.00011338 |
| C12orf54 | 0.181346 | 3.18E-05 | 0.0001134 |
| HIST1H4J | 0.1813401 | 3.19E-05 | 0.00011345 |
| SFRS8 | 0.1813384 | 3.19E-05 | 0.00011345 |
| CHRNA9 | -0.1812968 | 3.20E-05 | 0.00011391 |
| C16orf46 | -0.1812945 | 3.20E-05 | 0.00011392 |
| IQCH | 0.1812904 | 3.20E-05 | 0.00011395 |
| EMCN | -0.1812787 | 3.21E-05 | 0.00011406 |
| C21orf70 | 0.1812473 | 3.22E-05 | 0.00011441 |
| EIF3IP1 | 0.1812366 | 3.22E-05 | 0.00011451 |
| MICB | 0.181217 | 3.23E-05 | 0.00011472 |
| ADCY4 | -0.1812032 | 3.23E-05 | 0.00011486 |
| IL17D | -0.1811915 | 3.23E-05 | 0.00011498 |
| NDUFS2 | 0.1811799 | 3.24E-05 | 0.0001151 |
| TSTD1 | 0.1811741 | 3.24E-05 | 0.00011514 |
| LOC100216545 | 0.1811701 | 3.24E-05 | 0.00011517 |
| ZMAT2 | 0.1811668 | 3.24E-05 | 0.00011519 |
| ANKFY1 | -0.1811542 | 3.25E-05 | 0.00011532 |
| C3orf62 | 0.1811478 | 3.25E-05 | 0.00011537 |
| LIN28A | 0.1811137 | 3.26E-05 | 0.00011575 |
| SOX10 | -0.1810962 | 3.27E-05 | 0.00011594 |
| LOC93622 | 0.181093 | 3.27E-05 | 0.00011596 |
| RAB22A | -0.1810811 | 3.27E-05 | 0.00011608 |
| PRSS3 | -0.1810691 | 3.27E-05 | 0.0001162 |
| PLAC9 | -0.1810483 | 3.28E-05 | 0.00011642 |
| DNAJA1 | 0.1809962 | 3.30E-05 | 0.00011702 |
| USP38 | -0.1809584 | 3.31E-05 | 0.00011746 |
| ZNF275 | -0.1809076 | 3.33E-05 | 0.00011804 |
| ACTR2 | -0.1808624 | 3.34E-05 | 0.00011857 |
| LOC606724 | 0.1808113 | 3.36E-05 | 0.00011917 |
| OR2AT4 | 0.1807923 | 3.37E-05 | 0.00011938 |
| GPX8 | -0.1807897 | 3.37E-05 | 0.00011939 |
| SUZ12 | 0.1807672 | 3.38E-05 | 0.00011964 |
| PPAP2B | -0.1807654 | 3.38E-05 | 0.00011964 |
| TNFRSF25 | 0.1807529 | 3.38E-05 | 0.00011977 |
| ANKRD31 | -0.1807094 | 3.40E-05 | 0.00012028 |
| EIF5AL1 | 0.1807067 | 3.40E-05 | 0.00012029 |
| CD1A | -0.1806938 | 3.40E-05 | 0.00012042 |
| ZNF443 | 0.1806928 | 3.40E-05 | 0.00012042 |
| KIAA1199 | -0.1806666 | 3.41E-05 | 0.00012072 |
| C2orf68 | 0.1806434 | 3.42E-05 | 0.00012099 |
| TBC1D5 | -0.1805986 | 3.44E-05 | 0.00012152 |
| CRAMP1L | -0.1805781 | 3.44E-05 | 0.00012175 |
| GNMT | 0.1805756 | 3.44E-05 | 0.00012176 |
| RAB11FIP2 | -0.1805706 | 3.45E-05 | 0.0001218 |
| CLCN4 | -0.1805663 | 3.45E-05 | 0.00012183 |
| BMPER | -0.1805583 | 3.45E-05 | 0.00012191 |
| RFTN1 | -0.180555 | 3.45E-05 | 0.00012193 |
| C17orf72 | -0.1805187 | 3.46E-05 | 0.00012236 |
| CRCT1 | -0.1805134 | 3.47E-05 | 0.0001224 |
| ZNF703 | -0.1805031 | 3.47E-05 | 0.00012251 |
| NCAPD3 | 0.1804799 | 3.48E-05 | 0.00012278 |
| GYS1 | -0.1804734 | 3.48E-05 | 0.00012284 |
| MTHFR | -0.1804694 | 3.48E-05 | 0.00012287 |
| ZNF488 | 0.1804513 | 3.49E-05 | 0.00012307 |
| ARSI | -0.1804187 | 3.50E-05 | 0.00012346 |
| ARHGAP23 | -0.1804002 | 3.51E-05 | 0.00012366 |
| SH2D4B | 0.1803988 | 3.51E-05 | 0.00012366 |
| SEPT7P2 | 0.180391 | 3.51E-05 | 0.00012374 |
| MBD2 | 0.1803727 | 3.52E-05 | 0.00012395 |
| PLEKHH1 | 0.1803183 | 3.53E-05 | 0.00012461 |
| CDH22 | -0.1803102 | 3.54E-05 | 0.0001247 |
| NR2F6 | 0.180259 | 3.56E-05 | 0.00012532 |
| TARS2 | 0.1801929 | 3.58E-05 | 0.00012614 |
| ZNF154 | -0.1801631 | 3.59E-05 | 0.0001265 |
| C1orf57 | 0.1801621 | 3.59E-05 | 0.0001265 |
| HTR7P1 | -0.1801552 | 3.59E-05 | 0.00012656 |
| HINT2 | 0.1801301 | 3.60E-05 | 0.00012686 |
| SART3 | 0.1800864 | 3.62E-05 | 0.0001274 |
| RPS6KA6 | -0.1800685 | 3.63E-05 | 0.0001276 |
| LOC100129387 | 0.1800681 | 3.63E-05 | 0.0001276 |
| SLC43A1 | -0.1800635 | 3.63E-05 | 0.00012763 |
| ASPA | -0.180044 | 3.63E-05 | 0.00012786 |
| KIAA0776 | -0.1800184 | 3.64E-05 | 0.00012817 |
| MED31 | 0.1800062 | 3.65E-05 | 0.00012831 |
| RWDD2B | 0.1799403 | 3.67E-05 | 0.00012915 |
| STK16 | 0.1799272 | 3.68E-05 | 0.00012928 |
| C11orf54 | -0.1799271 | 3.68E-05 | 0.00012928 |
| MYL6B | 0.1799119 | 3.68E-05 | 0.00012945 |
| CBR4 | 0.1798988 | 3.69E-05 | 0.0001296 |
| WIZ | -0.179877 | 3.70E-05 | 0.00012987 |
| CD109 | -0.1798649 | 3.70E-05 | 0.00013 |
| TRAPPC10 | -0.1797957 | 3.73E-05 | 0.00013089 |
| SLC27A1 | -0.1797507 | 3.74E-05 | 0.00013147 |
| HAND2 | -0.179739 | 3.75E-05 | 0.00013159 |
| TM9SF4 | -0.1797385 | 3.75E-05 | 0.00013159 |
| FAM89B | -0.1797269 | 3.75E-05 | 0.00013172 |
| DNAJC25 | 0.1796748 | 3.77E-05 | 0.00013237 |
| MNX1 | 0.1796743 | 3.77E-05 | 0.00013237 |
| PCGF3 | -0.1796411 | 3.79E-05 | 0.0001328 |
| TBX1 | -0.1796317 | 3.79E-05 | 0.0001329 |
| FLJ41941 | -0.1796289 | 3.79E-05 | 0.00013292 |
| NOS1 | -0.179618 | 3.80E-05 | 0.00013304 |
| SOCS1 | 0.1796001 | 3.80E-05 | 0.00013326 |
| FZD6 | -0.1795471 | 3.82E-05 | 0.00013395 |
| HTRA2 | 0.1795036 | 3.84E-05 | 0.00013452 |
| ARL3 | 0.1794986 | 3.84E-05 | 0.00013456 |
| PIK3IP1 | -0.1794943 | 3.84E-05 | 0.0001346 |
| PANK2 | 0.1794809 | 3.85E-05 | 0.00013476 |
| RNF144A | -0.179478 | 3.85E-05 | 0.00013477 |
| LYPD3 | -0.1794535 | 3.86E-05 | 0.00013508 |
| NARFL | 0.1794415 | 3.86E-05 | 0.00013522 |
| TCTE3 | 0.1794189 | 3.87E-05 | 0.00013551 |
| LOC145474 | 0.1794093 | 3.88E-05 | 0.00013562 |
| PSEN1 | -0.1794031 | 3.88E-05 | 0.00013568 |
| CDON | -0.1793906 | 3.88E-05 | 0.00013583 |
| THAP9 | 0.1793848 | 3.89E-05 | 0.00013588 |
| PGM5P2 | -0.1793426 | 3.90E-05 | 0.00013644 |
| ERLEC1 | -0.1793412 | 3.90E-05 | 0.00013644 |
| TMEM145 | 0.1793387 | 3.90E-05 | 0.00013645 |
| MAML3 | -0.1793192 | 3.91E-05 | 0.00013669 |
| ZNF623 | -0.1793155 | 3.91E-05 | 0.00013672 |
| LPA | -0.179301 | 3.92E-05 | 0.0001369 |
| PCYT1A | -0.1792914 | 3.92E-05 | 0.000137 |
| CRABP2 | -0.1792872 | 3.92E-05 | 0.00013703 |
| DYNLRB1 | 0.1792863 | 3.92E-05 | 0.00013703 |
| CENPV | 0.1792807 | 3.93E-05 | 0.00013708 |
| VPS8 | -0.1792252 | 3.95E-05 | 0.00013783 |
| IRS2 | -0.1792192 | 3.95E-05 | 0.00013789 |
| ANAPC4 | 0.1792116 | 3.95E-05 | 0.00013797 |
| MED23 | -0.1792101 | 3.95E-05 | 0.00013797 |
| AATF | 0.1792065 | 3.96E-05 | 0.00013799 |
| GLIPR1 | -0.179186 | 3.96E-05 | 0.00013825 |
| GALNT5 | -0.1791842 | 3.97E-05 | 0.00013826 |
| SGSH | -0.1791737 | 3.97E-05 | 0.00013838 |
| MET | -0.1791545 | 3.98E-05 | 0.00013862 |
| HERC3 | -0.1791079 | 4.00E-05 | 0.00013925 |
| GIT2 | -0.1791057 | 4.00E-05 | 0.00013926 |
| PHGDH | 0.1790944 | 4.00E-05 | 0.00013939 |
| ZNF642 | 0.1790916 | 4.00E-05 | 0.00013941 |
| TAC1 | -0.179066 | 4.01E-05 | 0.00013975 |
| ARL2BP | -0.1790357 | 4.03E-05 | 0.00014015 |
| LOC151009 | 0.1790266 | 4.03E-05 | 0.00014025 |
| BAGE2 | -0.1790088 | 4.04E-05 | 0.00014048 |
| ZNF43 | -0.1789926 | 4.04E-05 | 0.00014069 |
| METTL3 | 0.1789039 | 4.08E-05 | 0.00014193 |
| C22orf23 | -0.1788982 | 4.08E-05 | 0.00014198 |
| CDH23 | -0.1788967 | 4.08E-05 | 0.00014198 |
| OPRL1 | -0.1788888 | 4.09E-05 | 0.00014207 |
| IAH1 | 0.1788613 | 4.10E-05 | 0.00014244 |
| GRSF1 | 0.1788597 | 4.10E-05 | 0.00014244 |
| CNTNAP1 | -0.1788323 | 4.11E-05 | 0.00014281 |
| GPATCH1 | 0.1788301 | 4.11E-05 | 0.00014281 |
| CETN2 | 0.1788145 | 4.12E-05 | 0.00014301 |
| SFRS11 | 0.1787907 | 4.13E-05 | 0.00014333 |
| LIPC | -0.1787576 | 4.14E-05 | 0.00014379 |
| MTMR14 | 0.1787518 | 4.14E-05 | 0.00014384 |
| TMEM108 | -0.1787194 | 4.16E-05 | 0.00014428 |
| ENDOU | -0.1787182 | 4.16E-05 | 0.00014428 |
| C1orf104 | 0.178715 | 4.16E-05 | 0.0001443 |
| IDI1 | 0.178627 | 4.19E-05 | 0.00014556 |
| MAP1LC3C | -0.1785977 | 4.21E-05 | 0.00014597 |
| PYGB | -0.17855 | 4.23E-05 | 0.00014664 |
| ATRX | -0.1785345 | 4.23E-05 | 0.00014685 |
| LAYN | -0.1785229 | 4.24E-05 | 0.00014699 |
| NFASC | -0.1785147 | 4.24E-05 | 0.00014709 |
| ZNF429 | -0.1785107 | 4.24E-05 | 0.00014712 |
| LOC642826 | 0.1784869 | 4.25E-05 | 0.00014745 |
| TMF1 | -0.178468 | 4.26E-05 | 0.00014771 |
| RNF5P1 | 0.1784509 | 4.27E-05 | 0.00014793 |
| KIAA0895L | 0.1784498 | 4.27E-05 | 0.00014793 |
| PDE4A | -0.1784303 | 4.28E-05 | 0.00014819 |
| DISC1 | -0.1784289 | 4.28E-05 | 0.00014819 |
| DAXX | 0.178419 | 4.28E-05 | 0.00014831 |
| APPL2 | -0.1784122 | 4.29E-05 | 0.00014838 |
| NIPA2 | 0.1783746 | 4.30E-05 | 0.00014892 |
| ZNHIT2 | 0.1783537 | 4.31E-05 | 0.00014921 |
| HEY2 | -0.1783476 | 4.31E-05 | 0.00014927 |
| ZNF385B | -0.1783224 | 4.32E-05 | 0.00014963 |
| PITPNM3 | -0.17831 | 4.33E-05 | 0.00014979 |
| TANC1 | -0.1783024 | 4.33E-05 | 0.00014988 |
| ACY1 | 0.1782914 | 4.34E-05 | 0.00015002 |
| DDX47 | 0.1782891 | 4.34E-05 | 0.00015003 |
| ENO1 | 0.1782675 | 4.35E-05 | 0.00015033 |
| PCDHGA10 | -0.1782339 | 4.36E-05 | 0.00015081 |
| PLSCR3 | -0.1782123 | 4.37E-05 | 0.00015111 |
| PPP1R2P3 | -0.1781656 | 4.39E-05 | 0.00015179 |
| FAM192A | 0.1781599 | 4.40E-05 | 0.00015186 |
| GDF10 | -0.1781522 | 4.40E-05 | 0.00015195 |
| C17orf104 | 0.1781254 | 4.41E-05 | 0.00015233 |
| TXLNA | 0.1780928 | 4.43E-05 | 0.0001528 |
| SNHG11 | 0.1780896 | 4.43E-05 | 0.00015282 |
| PLTP | -0.1780495 | 4.44E-05 | 0.00015342 |
| ATL1 | -0.1780426 | 4.45E-05 | 0.0001535 |
| LCA5 | -0.1780106 | 4.46E-05 | 0.00015396 |
| ZNF830 | 0.1780074 | 4.46E-05 | 0.00015399 |
| C20orf7 | 0.1779994 | 4.47E-05 | 0.00015408 |
| GFM2 | 0.1779977 | 4.47E-05 | 0.00015408 |
| FAM27C | 0.177971 | 4.48E-05 | 0.00015447 |
| SLC35B1 | 0.1779482 | 4.49E-05 | 0.0001548 |
| MASP1 | -0.1779197 | 4.50E-05 | 0.00015521 |
| GPR17 | -0.1779175 | 4.50E-05 | 0.00015522 |
| EIF3I | 0.1779019 | 4.51E-05 | 0.00015544 |
| PCDH19 | -0.1778996 | 4.51E-05 | 0.00015545 |
| MEPCE | 0.1778828 | 4.52E-05 | 0.00015568 |
| IGF1R | -0.1778585 | 4.53E-05 | 0.00015603 |
| HAS2 | -0.1778431 | 4.54E-05 | 0.00015625 |
| C16orf13 | 0.1778308 | 4.54E-05 | 0.00015642 |
| ELP2P | 0.1778214 | 4.55E-05 | 0.00015654 |
| HDGFRP2 | 0.1777966 | 4.56E-05 | 0.0001569 |
| GPS2 | 0.1777816 | 4.57E-05 | 0.00015711 |
| WBP2 | -0.177725 | 4.59E-05 | 0.00015797 |
| ABHD13 | -0.1777172 | 4.60E-05 | 0.00015807 |
| FCGBP | -0.17771 | 4.60E-05 | 0.00015816 |
| EBF3 | -0.1776758 | 4.61E-05 | 0.00015867 |
| PSMB6 | 0.1776695 | 4.62E-05 | 0.00015875 |
| XPR1 | -0.1776486 | 4.63E-05 | 0.00015905 |
| P2RY1 | -0.1776261 | 4.64E-05 | 0.00015938 |
| ANO8 | -0.1776235 | 4.64E-05 | 0.00015938 |
| MKLN1 | -0.1776231 | 4.64E-05 | 0.00015938 |
| TNPO3 | -0.1776013 | 4.65E-05 | 0.0001597 |
| PDE6D | 0.1775404 | 4.68E-05 | 0.00016065 |
| C10orf95 | 0.1775179 | 4.69E-05 | 0.00016098 |
| DGKQ | -0.1775137 | 4.69E-05 | 0.00016102 |
| WWP1 | -0.1774991 | 4.70E-05 | 0.00016123 |
| ATP6V0D1 | -0.1774273 | 4.73E-05 | 0.00016235 |
| GLYCTK | 0.1774262 | 4.73E-05 | 0.00016235 |
| ECSIT | 0.1774031 | 4.74E-05 | 0.0001627 |
| CSNK2A2 | -0.1773977 | 4.74E-05 | 0.00016276 |
| PISD | 0.1773563 | 4.76E-05 | 0.00016341 |
| LNX2 | -0.1773205 | 4.78E-05 | 0.00016397 |
| CACNB1 | -0.1772998 | 4.79E-05 | 0.00016428 |
| DUS1L | 0.1772647 | 4.81E-05 | 0.00016483 |
| KIF26A | -0.1772543 | 4.81E-05 | 0.00016497 |
| LFNG | -0.1772519 | 4.81E-05 | 0.00016497 |
| NDRG2 | -0.1772495 | 4.82E-05 | 0.00016497 |
| NAALAD2 | -0.1772493 | 4.82E-05 | 0.00016497 |
| UGT2B11 | 0.1772326 | 4.82E-05 | 0.00016522 |
| SDHAP2 | 0.1772109 | 4.83E-05 | 0.00016555 |
| C2orf18 | -0.1771759 | 4.85E-05 | 0.0001661 |
| LOC100125556 | 0.1771677 | 4.85E-05 | 0.00016621 |
| MOV10 | 0.1771415 | 4.87E-05 | 0.00016661 |
| USP18 | 0.177131 | 4.87E-05 | 0.00016676 |
| LOC374443 | 0.1770768 | 4.90E-05 | 0.00016763 |
| HOMER2 | -0.1770563 | 4.91E-05 | 0.00016795 |
| IDH3G | 0.1770062 | 4.93E-05 | 0.00016876 |
| ATP5SL | 0.1769991 | 4.94E-05 | 0.00016885 |
| HMG20B | 0.1769962 | 4.94E-05 | 0.00016887 |
| DLX5 | -0.1769764 | 4.95E-05 | 0.00016918 |
| CCDC46 | -0.1769548 | 4.96E-05 | 0.00016951 |
| MAP3K7 | -0.1769489 | 4.96E-05 | 0.00016959 |
| PCBD1 | 0.1769298 | 4.97E-05 | 0.00016988 |
| C6orf150 | 0.1769213 | 4.98E-05 | 0.00017 |
| MFF | 0.1769192 | 4.98E-05 | 0.00017 |
| PSORS1C3 | 0.1769151 | 4.98E-05 | 0.00017004 |
| ST6GALNAC3 | -0.1769067 | 4.98E-05 | 0.00017014 |
| KIT | -0.176906 | 4.98E-05 | 0.00017014 |
| ZNF570 | -0.1768614 | 5.01E-05 | 0.00017087 |
| GZMB | 0.1768242 | 5.02E-05 | 0.00017147 |
| ZNF213 | -0.176815 | 5.03E-05 | 0.0001716 |
| C6orf106 | -0.1768088 | 5.03E-05 | 0.00017168 |
| TCAP | -0.1767919 | 5.04E-05 | 0.00017194 |
| IFFO1 | -0.1767892 | 5.04E-05 | 0.00017196 |
| LBP | -0.1767554 | 5.06E-05 | 0.00017251 |
| STX7 | -0.1767384 | 5.07E-05 | 0.00017277 |
| MPI | 0.1767225 | 5.08E-05 | 0.00017302 |
| PDF | 0.1766896 | 5.09E-05 | 0.00017355 |
| TMEM57 | -0.1766808 | 5.10E-05 | 0.00017368 |
| TNFRSF10B | 0.176675 | 5.10E-05 | 0.00017375 |
| PEBP1 | 0.1766678 | 5.10E-05 | 0.00017384 |
| HN1 | 0.176665 | 5.10E-05 | 0.00017386 |
| SF3A1 | -0.1766593 | 5.11E-05 | 0.00017393 |
| FLAD1 | 0.1766425 | 5.12E-05 | 0.00017419 |
| NR2F1 | -0.1766383 | 5.12E-05 | 0.00017424 |
| YIPF5 | -0.1766216 | 5.13E-05 | 0.0001745 |
| TRIM3 | -0.1765928 | 5.14E-05 | 0.00017495 |
| SMARCD2 | 0.1765924 | 5.14E-05 | 0.00017495 |
| SLC4A4 | -0.1765772 | 5.15E-05 | 0.00017518 |
| DOCK11 | -0.1765721 | 5.15E-05 | 0.00017524 |
| ANKLE1 | 0.176559 | 5.16E-05 | 0.00017544 |
| NPIPL3 | 0.1765511 | 5.16E-05 | 0.00017555 |
| SEC61A1 | -0.1765443 | 5.17E-05 | 0.00017564 |
| MTIF3 | 0.176537 | 5.17E-05 | 0.00017574 |
| RSPO1 | -0.176517 | 5.18E-05 | 0.00017606 |
| EMR2 | -0.1765101 | 5.18E-05 | 0.00017615 |
| HDDC2 | 0.1764968 | 5.19E-05 | 0.00017635 |
| STOX1 | 0.1764766 | 5.20E-05 | 0.00017666 |
| PLEKHG5 | -0.1764759 | 5.20E-05 | 0.00017666 |
| FBXO3 | -0.1764733 | 5.20E-05 | 0.00017667 |
| IDI2 | 0.1764618 | 5.21E-05 | 0.00017681 |
| SST | -0.1764603 | 5.21E-05 | 0.00017681 |
| FPGS | 0.1764602 | 5.21E-05 | 0.00017681 |
| USP7 | -0.1764536 | 5.21E-05 | 0.00017689 |
| SMTN | -0.1764527 | 5.21E-05 | 0.00017689 |
| ANO2 | -0.1764452 | 5.22E-05 | 0.00017699 |
| SYK | 0.1764359 | 5.22E-05 | 0.00017712 |
| TMEM86B | 0.1764323 | 5.22E-05 | 0.00017716 |
| ANKRD57 | -0.1764045 | 5.24E-05 | 0.00017762 |
| REM2 | 0.1763852 | 5.25E-05 | 0.00017793 |
| ARF4 | -0.1763649 | 5.26E-05 | 0.00017826 |
| F5 | -0.1763403 | 5.27E-05 | 0.00017866 |
| GNAI1 | -0.1763238 | 5.28E-05 | 0.00017893 |
| C1orf128 | 0.176315 | 5.29E-05 | 0.00017905 |
| PCNXL2 | -0.1763067 | 5.29E-05 | 0.00017917 |
| TXNDC16 | 0.1763029 | 5.29E-05 | 0.00017921 |
| SNHG5 | 0.1762887 | 5.30E-05 | 0.00017943 |
| TRIM7 | 0.1762829 | 5.30E-05 | 0.0001795 |
| ITGB1 | -0.1762807 | 5.30E-05 | 0.00017951 |
| EDN1 | -0.1762726 | 5.31E-05 | 0.00017963 |
| FBXL5 | -0.1762692 | 5.31E-05 | 0.00017966 |
| FDPSL2A | 0.1762573 | 5.32E-05 | 0.00017984 |
| NETO1 | -0.1761809 | 5.36E-05 | 0.00018118 |
| WLS | -0.1761733 | 5.36E-05 | 0.00018129 |
| COX6A2 | -0.1760999 | 5.40E-05 | 0.00018258 |
| C1orf116 | -0.1760773 | 5.41E-05 | 0.00018294 |
| BREA2 | 0.1760768 | 5.41E-05 | 0.00018294 |
| SUPT16H | 0.1760747 | 5.41E-05 | 0.00018295 |
| RPL13AP3 | 0.1760545 | 5.42E-05 | 0.00018328 |
| HDAC9 | -0.1760301 | 5.44E-05 | 0.0001837 |
| MYOZ2 | -0.1760285 | 5.44E-05 | 0.0001837 |
| NUMB | -0.176024 | 5.44E-05 | 0.00018375 |
| PDCD10 | 0.1759984 | 5.45E-05 | 0.00018419 |
| GBGT1 | -0.1759865 | 5.46E-05 | 0.00018437 |
| DIRC3 | -0.1759741 | 5.47E-05 | 0.00018457 |
| ZNF460 | -0.1759722 | 5.47E-05 | 0.00018457 |
| F2RL3 | -0.1759452 | 5.48E-05 | 0.00018504 |
| CFLAR | -0.1759363 | 5.49E-05 | 0.00018517 |
| NSDHL | 0.1759305 | 5.49E-05 | 0.00018524 |
| FOLR3 | -0.1759271 | 5.49E-05 | 0.00018527 |
| TTC31 | 0.1759102 | 5.50E-05 | 0.00018555 |
| ARGLU1 | 0.1758881 | 5.51E-05 | 0.00018593 |
| LMBR1 | -0.1758768 | 5.52E-05 | 0.00018611 |
| CCDC104 | 0.1758743 | 5.52E-05 | 0.00018611 |
| COPE | 0.1758735 | 5.52E-05 | 0.00018611 |
| SLC19A1 | 0.1758539 | 5.53E-05 | 0.00018644 |
| AQP9 | -0.1758226 | 5.55E-05 | 0.00018699 |
| CDC26 | 0.1758172 | 5.55E-05 | 0.00018705 |
| CBX4 | -0.1758072 | 5.56E-05 | 0.00018721 |
| NRADDP | 0.1757913 | 5.57E-05 | 0.00018747 |
| PCDHA6 | -0.1757767 | 5.58E-05 | 0.00018771 |
| HNMT | -0.1757703 | 5.58E-05 | 0.0001878 |
| TRIML1 | -0.1757295 | 5.60E-05 | 0.00018853 |
| SNRNP48 | 0.1757224 | 5.61E-05 | 0.0001886 |
| APOBEC3H | 0.1757223 | 5.61E-05 | 0.0001886 |
| TRAPPC2L | 0.175683 | 5.63E-05 | 0.0001893 |
| ANKRD54 | 0.1756697 | 5.63E-05 | 0.00018952 |
| ASCC2 | -0.1756498 | 5.65E-05 | 0.00018986 |
| ARHGAP36 | -0.1756483 | 5.65E-05 | 0.00018986 |
| KLF13 | -0.1756227 | 5.66E-05 | 0.00019031 |
| DNAJB6 | -0.1755583 | 5.70E-05 | 0.0001915 |
| LOC100130557 | 0.1755521 | 5.70E-05 | 0.00019157 |
| KLF8 | -0.1755508 | 5.70E-05 | 0.00019157 |
| GATA2 | -0.175527 | 5.71E-05 | 0.000192 |
| IGSF21 | -0.1755202 | 5.72E-05 | 0.00019209 |
| HDAC2 | 0.1755157 | 5.72E-05 | 0.00019214 |
| PKIG | -0.1755137 | 5.72E-05 | 0.00019214 |
| RAD17 | 0.175512 | 5.72E-05 | 0.00019214 |
| CTSE | 0.1755109 | 5.72E-05 | 0.00019214 |
| DHX38 | -0.175461 | 5.75E-05 | 0.00019306 |
| PRPSAP2 | 0.1754579 | 5.75E-05 | 0.00019309 |
| ZNF800 | -0.1754424 | 5.76E-05 | 0.00019335 |
| KIAA1147 | -0.1754314 | 5.77E-05 | 0.00019353 |
| ATP5L2 | 0.1754261 | 5.77E-05 | 0.0001936 |
| SENP1 | 0.1753912 | 5.79E-05 | 0.00019424 |
| HNF1A | 0.1753682 | 5.81E-05 | 0.00019465 |
| GOLGA7 | 0.1753268 | 5.83E-05 | 0.00019541 |
| CAMK2N1 | -0.1753081 | 5.84E-05 | 0.00019574 |
| SNX30 | -0.1752634 | 5.87E-05 | 0.00019657 |
| ACSBG2 | 0.1752599 | 5.87E-05 | 0.00019661 |
| ZCCHC3 | 0.1752518 | 5.87E-05 | 0.00019673 |
| C9orf5 | -0.1752473 | 5.88E-05 | 0.00019679 |
| ADAM19 | -0.1752135 | 5.89E-05 | 0.00019741 |
| ALDH3B2 | -0.1751988 | 5.90E-05 | 0.00019767 |
| AFAP1L2 | -0.1751817 | 5.91E-05 | 0.00019797 |
| CES4 | -0.1751411 | 5.94E-05 | 0.00019873 |
| PLAGL1 | -0.1751138 | 5.95E-05 | 0.00019923 |
| DST | -0.1751074 | 5.96E-05 | 0.00019933 |
| ITGA2 | -0.1750898 | 5.97E-05 | 0.00019963 |
| RAB6A | -0.1750887 | 5.97E-05 | 0.00019963 |
| ENPP2 | -0.1750579 | 5.99E-05 | 0.0002002 |
| CRYBB3 | 0.1750338 | 6.00E-05 | 0.00020065 |
| GPX3 | -0.1750149 | 6.01E-05 | 0.00020099 |
| COX15 | 0.1749935 | 6.02E-05 | 0.00020138 |
| PYGM | -0.1749767 | 6.03E-05 | 0.00020168 |
| DNASE1L1 | -0.1749583 | 6.05E-05 | 0.00020201 |
| RNF8 | 0.1749447 | 6.05E-05 | 0.00020225 |
| MATR3 | 0.1749299 | 6.06E-05 | 0.00020251 |
| ITGAM | -0.174915 | 6.07E-05 | 0.00020278 |
| IL29 | 0.1748988 | 6.08E-05 | 0.00020307 |
| ZNF57 | 0.174897 | 6.08E-05 | 0.00020307 |
| TMEM159 | -0.1748756 | 6.09E-05 | 0.00020346 |
| AZGP1 | -0.1748741 | 6.10E-05 | 0.00020346 |
| SFXN1 | 0.1748548 | 6.11E-05 | 0.00020382 |
| SPRY3 | -0.1748086 | 6.14E-05 | 0.00020471 |
| ZNF346 | 0.1747871 | 6.15E-05 | 0.00020511 |
| DDX28 | 0.1747628 | 6.16E-05 | 0.00020557 |
| NSUN4 | 0.1747205 | 6.19E-05 | 0.00020639 |
| INTS12 | 0.1747132 | 6.19E-05 | 0.00020651 |
| ZER1 | -0.1747029 | 6.20E-05 | 0.00020668 |
| FAM115A | -0.1746366 | 6.24E-05 | 0.000208 |
| RILPL2 | 0.1746236 | 6.25E-05 | 0.00020824 |
| PIAS3 | -0.1746109 | 6.26E-05 | 0.00020846 |
| FERMT1 | -0.1745775 | 6.28E-05 | 0.00020911 |
| CCL14 | -0.174537 | 6.30E-05 | 0.00020991 |
| RASD1 | -0.1744935 | 6.33E-05 | 0.00021078 |
| CLEC18B | -0.1744449 | 6.36E-05 | 0.00021176 |
| C1orf85 | -0.1744421 | 6.36E-05 | 0.00021176 |
| KIAA0907 | 0.1744413 | 6.36E-05 | 0.00021176 |
| SPCS2 | 0.1744371 | 6.36E-05 | 0.00021181 |
| OGFOD2 | 0.1744171 | 6.38E-05 | 0.00021219 |
| ZNF773 | -0.1744118 | 6.38E-05 | 0.00021224 |
| DCBLD1 | -0.1744118 | 6.38E-05 | 0.00021224 |
| CHCHD7 | 0.1743444 | 6.42E-05 | 0.00021361 |
| LCN12 | 0.1743171 | 6.44E-05 | 0.00021415 |
| ROBO2 | -0.1743119 | 6.44E-05 | 0.00021423 |
| CLK2 | 0.1742793 | 6.46E-05 | 0.00021488 |
| POC1B | 0.1742518 | 6.48E-05 | 0.00021542 |
| KIAA0564 | -0.1742335 | 6.49E-05 | 0.00021577 |
| HMBS | 0.1742173 | 6.50E-05 | 0.00021608 |
| MARK2 | -0.1741946 | 6.52E-05 | 0.00021653 |
| C20orf26 | -0.1741797 | 6.53E-05 | 0.00021681 |
| NAA30 | -0.1741703 | 6.53E-05 | 0.00021698 |
| TM7SF3 | 0.1741538 | 6.54E-05 | 0.00021729 |
| FGF7 | -0.1741205 | 6.56E-05 | 0.00021797 |
| BCL2L2 | -0.1741187 | 6.57E-05 | 0.00021797 |
| ITGA4 | -0.1741075 | 6.57E-05 | 0.00021817 |
| TAOK3 | -0.1740988 | 6.58E-05 | 0.00021832 |
| LCE1F | -0.1740768 | 6.59E-05 | 0.00021876 |
| NUMA1 | -0.1740514 | 6.61E-05 | 0.00021925 |
| AP3D1 | -0.1740508 | 6.61E-05 | 0.00021925 |
| RGS16 | -0.1740345 | 6.62E-05 | 0.00021956 |
| FAM26F | 0.1740197 | 6.63E-05 | 0.00021984 |
| NAE1 | 0.1739962 | 6.65E-05 | 0.00022032 |
| DNAJC1 | 0.173973 | 6.66E-05 | 0.00022076 |
| CBS | 0.1739725 | 6.66E-05 | 0.00022076 |
| PROP1 | 0.1739248 | 6.69E-05 | 0.00022175 |
| ZCCHC7 | 0.1739197 | 6.70E-05 | 0.00022183 |
| RNF160 | -0.1739157 | 6.70E-05 | 0.00022188 |
| ZBTB41 | -0.1739036 | 6.71E-05 | 0.00022211 |
| CEPT1 | 0.173889 | 6.72E-05 | 0.00022239 |
| DENND2C | -0.1738742 | 6.73E-05 | 0.00022268 |
| TTC4 | 0.1738682 | 6.73E-05 | 0.00022275 |
| SLC5A10 | -0.1738675 | 6.73E-05 | 0.00022275 |
| RHOT2 | 0.1738572 | 6.74E-05 | 0.00022294 |
| PLD6 | 0.1738525 | 6.74E-05 | 0.000223 |
| RCC2 | 0.1738462 | 6.74E-05 | 0.00022311 |
| ZFP62 | 0.1738232 | 6.76E-05 | 0.00022357 |
| PEX26 | -0.173816 | 6.76E-05 | 0.00022369 |
| TXNDC12 | 0.1737633 | 6.80E-05 | 0.00022481 |
| CCDC144C | -0.17373 | 6.82E-05 | 0.00022551 |
| WDR92 | 0.1737072 | 6.84E-05 | 0.00022598 |
| CCND3 | 0.1737051 | 6.84E-05 | 0.00022599 |
| TMEM80 | 0.1736948 | 6.84E-05 | 0.00022618 |
| VAMP2 | -0.1736869 | 6.85E-05 | 0.00022632 |
| RNF114 | 0.1736838 | 6.85E-05 | 0.00022635 |
| TTC19 | 0.1736813 | 6.85E-05 | 0.00022635 |
| KDM5C | -0.1736803 | 6.85E-05 | 0.00022635 |
| SPRY2 | -0.1736744 | 6.86E-05 | 0.00022645 |
| EAF1 | -0.1736638 | 6.87E-05 | 0.00022664 |
| CDKN1A | -0.1736443 | 6.88E-05 | 0.00022704 |
| HERC2P2 | 0.1736401 | 6.88E-05 | 0.0002271 |
| GLP1R | -0.1736334 | 6.89E-05 | 0.00022721 |
| FAHD2A | 0.1736281 | 6.89E-05 | 0.00022729 |
| NCRNA00120 | -0.1736118 | 6.90E-05 | 0.00022761 |
| C1QL1 | 0.1736056 | 6.90E-05 | 0.00022772 |
| DKFZp779M0652 | -0.1735774 | 6.92E-05 | 0.00022831 |
| YTHDF3 | -0.173533 | 6.95E-05 | 0.00022927 |
| CNGB1 | -0.1735173 | 6.96E-05 | 0.00022958 |
| ASB9 | 0.1735025 | 6.97E-05 | 0.00022988 |
| CEBPZ | 0.1734957 | 6.98E-05 | 0.00022999 |
| ERI3 | 0.1734848 | 6.99E-05 | 0.0002302 |
| USP3 | 0.1734582 | 7.00E-05 | 0.00023076 |
| AMOT | -0.1734467 | 7.01E-05 | 0.00023098 |
| NARF | 0.173422 | 7.03E-05 | 0.0002315 |
| BLOC1S2 | 0.173403 | 7.04E-05 | 0.0002319 |
| KCTD12 | -0.173343 | 7.08E-05 | 0.00023322 |
| HIPK1 | -0.1733411 | 7.09E-05 | 0.00023323 |
| PRIMA1 | 0.1733051 | 7.11E-05 | 0.00023401 |
| ATM | -0.1732917 | 7.12E-05 | 0.00023428 |
| GPR15 | -0.1732773 | 7.13E-05 | 0.00023457 |
| EGFLAM | -0.1732755 | 7.13E-05 | 0.00023457 |
| C20orf94 | -0.173249 | 7.15E-05 | 0.00023515 |
| EPB41L5 | -0.1732382 | 7.16E-05 | 0.00023536 |
| NEK3 | 0.1732095 | 7.18E-05 | 0.00023598 |
| SELE | -0.1731994 | 7.18E-05 | 0.00023617 |
| TOR1AIP1 | -0.1731937 | 7.19E-05 | 0.00023627 |
| BRPF3 | -0.1731714 | 7.20E-05 | 0.00023674 |
| RFX8 | -0.173119 | 7.24E-05 | 0.00023792 |
| TMEM161B | 0.1730893 | 7.26E-05 | 0.00023857 |
| C3orf42 | 0.1730765 | 7.27E-05 | 0.00023883 |
| PRPF8 | -0.173067 | 7.28E-05 | 0.00023901 |
| CYB5B | 0.1730609 | 7.28E-05 | 0.00023911 |
| CFHR3 | -0.1730346 | 7.30E-05 | 0.00023969 |
| ZNF662 | -0.17303 | 7.30E-05 | 0.00023976 |
| AGRN | -0.1729621 | 7.35E-05 | 0.00024131 |
| LIPT1 | 0.1729194 | 7.38E-05 | 0.00024225 |
| DRAM2 | 0.172919 | 7.38E-05 | 0.00024225 |
| TBX15 | -0.1729131 | 7.39E-05 | 0.00024232 |
| DDN | -0.1729128 | 7.39E-05 | 0.00024232 |
| LRTM1 | -0.1728892 | 7.41E-05 | 0.0002428 |
| LCOR | -0.1728892 | 7.41E-05 | 0.0002428 |
| CIDECP | 0.1728294 | 7.45E-05 | 0.00024418 |
| SEC63 | -0.1728124 | 7.46E-05 | 0.00024454 |
| RWDD1 | 0.1727937 | 7.47E-05 | 0.00024495 |
| BAD | 0.172775 | 7.49E-05 | 0.00024535 |
| DIO3 | -0.1727736 | 7.49E-05 | 0.00024535 |
| APPBP2 | -0.1727589 | 7.50E-05 | 0.00024566 |
| TRIM44 | -0.1727507 | 7.51E-05 | 0.00024582 |
| LOC644538 | -0.172737 | 7.52E-05 | 0.00024611 |
| DPYSL3 | -0.1727216 | 7.53E-05 | 0.00024644 |
| IRF3 | 0.1726821 | 7.56E-05 | 0.00024735 |
| ZSCAN18 | -0.172634 | 7.59E-05 | 0.00024841 |
| PNPLA3 | -0.172633 | 7.59E-05 | 0.00024841 |
| MFAP3 | -0.172633 | 7.59E-05 | 0.00024841 |
| CXCR7 | -0.1726102 | 7.61E-05 | 0.00024892 |
| C9orf53 | 0.1725972 | 7.62E-05 | 0.0002492 |
| CHRNA10 | 0.1725623 | 7.64E-05 | 0.00025 |
| CTAGE1 | -0.1725493 | 7.65E-05 | 0.00025028 |
| RP9P | 0.1725394 | 7.66E-05 | 0.00025048 |
| DDX23 | 0.1725252 | 7.67E-05 | 0.00025079 |
| TMSB15A | 0.1725235 | 7.67E-05 | 0.00025079 |
| PRR3 | 0.1724768 | 7.71E-05 | 0.00025189 |
| PDPN | -0.1724176 | 7.75E-05 | 0.0002533 |
| FAM13AOS | 0.1724131 | 7.76E-05 | 0.00025337 |
| XG | -0.1723993 | 7.77E-05 | 0.00025367 |
| SLC38A11 | -0.1723939 | 7.77E-05 | 0.00025376 |
| TPM2 | -0.1723836 | 7.78E-05 | 0.00025398 |
| GLRB | -0.1723658 | 7.79E-05 | 0.00025438 |
| LOC146336 | -0.1723526 | 7.80E-05 | 0.00025466 |
| NEXN | -0.1723487 | 7.81E-05 | 0.0002547 |
| BACH2 | -0.1723477 | 7.81E-05 | 0.0002547 |
| KCNJ15 | -0.1723319 | 7.82E-05 | 0.00025504 |
| LILRP2 | 0.1723306 | 7.82E-05 | 0.00025504 |
| CCNI2 | 0.1723281 | 7.82E-05 | 0.00025506 |
| SOX30 | 0.1723101 | 7.83E-05 | 0.00025547 |
| PPCS | 0.1722922 | 7.85E-05 | 0.00025587 |
| OR7C2 | 0.1722658 | 7.87E-05 | 0.00025649 |
| MYLK3 | -0.1722558 | 7.88E-05 | 0.0002567 |
| C12orf52 | 0.1722295 | 7.90E-05 | 0.00025731 |
| SPRR2E | -0.1722217 | 7.90E-05 | 0.00025746 |
| RPL8 | 0.1722076 | 7.91E-05 | 0.00025775 |
| UQCC | 0.1722069 | 7.91E-05 | 0.00025775 |
| CCDC36 | -0.1721915 | 7.93E-05 | 0.0002581 |
| NEUROG2 | 0.1721626 | 7.95E-05 | 0.00025878 |
| ZMYND11 | -0.172143 | 7.96E-05 | 0.00025923 |
| EPS15 | -0.1721379 | 7.97E-05 | 0.00025932 |
| CELSR2 | -0.1721027 | 7.99E-05 | 0.00026016 |
| C2orf39 | -0.1720837 | 8.01E-05 | 0.0002606 |
| PGRMC1 | 0.1720621 | 8.03E-05 | 0.00026111 |
| CTU1 | 0.172044 | 8.04E-05 | 0.00026152 |
| PSD3 | -0.1720319 | 8.05E-05 | 0.00026179 |
| KCTD20 | -0.172021 | 8.06E-05 | 0.00026202 |
| DNAJA3 | 0.1719988 | 8.08E-05 | 0.00026255 |
| PPP1R1A | -0.1719655 | 8.10E-05 | 0.00026333 |
| ASPRV1 | -0.1719641 | 8.10E-05 | 0.00026333 |
| AQP7 | -0.1719632 | 8.10E-05 | 0.00026333 |
| GPR158 | -0.1719478 | 8.12E-05 | 0.00026368 |
| RHBDL1 | 0.1719442 | 8.12E-05 | 0.00026373 |
| CCT2 | 0.1718968 | 8.16E-05 | 0.0002649 |
| STON2 | -0.1718637 | 8.18E-05 | 0.00026571 |
| ITPKB | -0.1718577 | 8.19E-05 | 0.00026582 |
| PNKP | 0.1718463 | 8.20E-05 | 0.00026607 |
| FAM126A | -0.1718203 | 8.22E-05 | 0.0002667 |
| POU6F1 | -0.1718144 | 8.22E-05 | 0.00026681 |
| RPL13A | 0.1717608 | 8.26E-05 | 0.00026816 |
| FAM167A | -0.1717536 | 8.27E-05 | 0.0002683 |
| C19orf30 | 0.1717488 | 8.27E-05 | 0.00026838 |
| S1PR2 | -0.1717296 | 8.29E-05 | 0.00026884 |
| CX3CR1 | -0.1717148 | 8.30E-05 | 0.00026918 |
| ARHGAP5 | -0.171706 | 8.31E-05 | 0.00026937 |
| SEC11C | 0.1716936 | 8.32E-05 | 0.00026965 |
| LONRF2 | -0.1716681 | 8.34E-05 | 0.00027027 |
| EIF1 | 0.1716631 | 8.34E-05 | 0.00027036 |
| RANBP3 | 0.1716177 | 8.38E-05 | 0.00027151 |
| NCF2 | -0.1715914 | 8.40E-05 | 0.00027215 |
| C6orf57 | 0.1715653 | 8.42E-05 | 0.0002728 |
| HMGB3L1 | 0.1715434 | 8.44E-05 | 0.00027333 |
| PELP1 | 0.171535 | 8.45E-05 | 0.00027351 |
| ZFP36L2 | -0.1714889 | 8.48E-05 | 0.00027469 |
| CPT1B | 0.1714825 | 8.49E-05 | 0.00027482 |
| SLC26A6 | 0.1714589 | 8.51E-05 | 0.0002754 |
| ATP6V0B | 0.1714456 | 8.52E-05 | 0.00027571 |
| OSGIN1 | -0.1714434 | 8.52E-05 | 0.00027573 |
| TM7SF4 | -0.1714279 | 8.53E-05 | 0.0002761 |
| PNPLA8 | -0.1713995 | 8.56E-05 | 0.00027681 |
| TRMT11 | 0.171373 | 8.58E-05 | 0.00027748 |
| STRA6 | -0.1713638 | 8.59E-05 | 0.00027768 |
| WNT8A | 0.1712808 | 8.66E-05 | 0.00027987 |
| ZC3H12B | -0.1712628 | 8.67E-05 | 0.00028031 |
| FAIM2 | -0.1712603 | 8.67E-05 | 0.00028034 |
| MIF | 0.1712482 | 8.68E-05 | 0.00028062 |
| LOC100129034 | -0.1712285 | 8.70E-05 | 0.00028111 |
| ZNF841 | -0.1712108 | 8.72E-05 | 0.00028155 |
| ATOH8 | -0.1712053 | 8.72E-05 | 0.00028165 |
| HEATR7A | -0.1711688 | 8.75E-05 | 0.0002826 |
| TBPL1 | 0.1711664 | 8.75E-05 | 0.00028262 |
| RBM7 | -0.171089 | 8.82E-05 | 0.00028469 |
| ARID1A | -0.1710639 | 8.84E-05 | 0.00028534 |
| PPAP2A | -0.1710563 | 8.85E-05 | 0.00028545 |
| RPA1 | 0.1710545 | 8.85E-05 | 0.00028545 |
| CCDC28B | 0.1710529 | 8.85E-05 | 0.00028545 |
| CADM3 | -0.1710525 | 8.85E-05 | 0.00028545 |
| WTAP | 0.1710514 | 8.85E-05 | 0.00028545 |
| SPINK5 | -0.1710383 | 8.86E-05 | 0.00028577 |
| CTSD | -0.1710252 | 8.87E-05 | 0.00028609 |
| GTF3A | 0.1710067 | 8.89E-05 | 0.00028655 |
| UBTD2 | -0.170999 | 8.90E-05 | 0.0002867 |
| PIGG | -0.1709981 | 8.90E-05 | 0.0002867 |
| UHRF1BP1L | -0.1709824 | 8.91E-05 | 0.00028709 |
| PI4K2A | -0.1709519 | 8.94E-05 | 0.00028789 |
| SLC29A3 | -0.1709209 | 8.96E-05 | 0.0002887 |
| ITIH3 | -0.1709148 | 8.97E-05 | 0.00028883 |
| PLA2G2A | -0.1709056 | 8.98E-05 | 0.00028904 |
| MEX3C | -0.1708576 | 9.02E-05 | 0.00029033 |
| AIDA | 0.1708342 | 9.04E-05 | 0.00029094 |
| SNORD97 | 0.1708146 | 9.06E-05 | 0.00029142 |
| NCL | 0.1708137 | 9.06E-05 | 0.00029142 |
| SNRK | -0.170802 | 9.07E-05 | 0.00029171 |
| APOOL | -0.1707953 | 9.07E-05 | 0.00029185 |
| CHIT1 | -0.1707797 | 9.09E-05 | 0.00029224 |
| ANGPT1 | -0.1707626 | 9.10E-05 | 0.00029268 |
| CCDC42B | 0.1707505 | 9.11E-05 | 0.00029297 |
| FPR3 | -0.1707478 | 9.11E-05 | 0.000293 |
| LRRC42 | 0.1707371 | 9.12E-05 | 0.00029325 |
| HEXIM2 | 0.1706849 | 9.17E-05 | 0.00029466 |
| SLC2A14 | -0.1706824 | 9.17E-05 | 0.00029466 |
| C11orf84 | 0.1706808 | 9.17E-05 | 0.00029466 |
| CREBL2 | -0.1706807 | 9.17E-05 | 0.00029466 |
| TXNDC17 | 0.1706791 | 9.17E-05 | 0.00029466 |
| AFF3 | -0.1706724 | 9.18E-05 | 0.00029481 |
| BET1 | 0.1706179 | 9.23E-05 | 0.00029631 |
| NETO2 | 0.1706125 | 9.23E-05 | 0.00029642 |
| FLJ45244 | 0.1706068 | 9.24E-05 | 0.00029653 |
| SGMS1 | -0.1705946 | 9.25E-05 | 0.00029684 |
| BCO2 | -0.1705849 | 9.26E-05 | 0.00029707 |
| RGPD6 | -0.1705483 | 9.29E-05 | 0.00029804 |
| ZBTB8A | 0.1705476 | 9.29E-05 | 0.00029804 |
| C17orf67 | 0.1705383 | 9.30E-05 | 0.00029826 |
| ADORA2B | 0.1704868 | 9.35E-05 | 0.00029969 |
| KLHL34 | -0.1704724 | 9.36E-05 | 0.00030006 |
| LPHN3 | -0.1704581 | 9.37E-05 | 0.00030039 |
| NKX2-3 | 0.1704575 | 9.37E-05 | 0.00030039 |
| TERF2IP | -0.1704531 | 9.38E-05 | 0.00030046 |
| HOXB13 | 0.1704521 | 9.38E-05 | 0.00030046 |
| ANXA6 | -0.1704448 | 9.38E-05 | 0.00030062 |
| SHC3 | -0.1704219 | 9.40E-05 | 0.00030123 |
| HIVEP1 | -0.17042 | 9.41E-05 | 0.00030124 |
| UBFD1 | -0.1704099 | 9.42E-05 | 0.00030149 |
| BAT2L1 | -0.1703194 | 9.50E-05 | 0.00030407 |
| SPG20 | -0.1703018 | 9.51E-05 | 0.00030454 |
| RPL28 | 0.1702511 | 9.56E-05 | 0.00030598 |
| COL27A1 | -0.1702228 | 9.59E-05 | 0.00030676 |
| LOC399959 | -0.1702097 | 9.60E-05 | 0.0003071 |
| IL1F8 | -0.1702055 | 9.60E-05 | 0.00030717 |
| RAPGEF6 | -0.1701862 | 9.62E-05 | 0.0003077 |
| AP2S1 | 0.1701727 | 9.63E-05 | 0.00030805 |
| GATAD2A | -0.1701629 | 9.64E-05 | 0.00030829 |
| ODZ1 | -0.1701135 | 9.69E-05 | 0.00030969 |
| C6orf120 | -0.1701125 | 9.69E-05 | 0.00030969 |
| GULP1 | -0.1700717 | 9.73E-05 | 0.00031085 |
| NEDD9 | -0.1700359 | 9.76E-05 | 0.00031187 |
| SLC25A22 | 0.1699988 | 9.79E-05 | 0.00031293 |
| KCNAB1 | -0.1699821 | 9.81E-05 | 0.00031339 |
| PKP1 | -0.1699796 | 9.81E-05 | 0.00031341 |
| KIAA0408 | -0.1699378 | 9.85E-05 | 0.00031462 |
| CYTSB | -0.169934 | 9.86E-05 | 0.00031465 |
| TRPV2 | -0.1699336 | 9.86E-05 | 0.00031465 |
| CEP120 | 0.1699311 | 9.86E-05 | 0.00031467 |
| CPE | -0.1699173 | 9.87E-05 | 0.000315 |
| RBM20 | -0.1699169 | 9.87E-05 | 0.000315 |
| PSMB10 | 0.1698745 | 9.91E-05 | 0.00031624 |
| NAT8L | -0.1698206 | 9.96E-05 | 0.00031773 |
| RGN | -0.1698205 | 9.96E-05 | 0.00031773 |
| MYEOV2 | 0.1698205 | 9.96E-05 | 0.00031773 |
| ZNF695 | 0.1698125 | 9.97E-05 | 0.00031792 |
| TTC9C | 0.1698105 | 9.97E-05 | 0.00031793 |
| DLEU7 | -0.1697751 | 0.00010007 | 0.00031896 |
| RBM6 | 0.1697699 | 0.00010012 | 0.00031907 |
| POPDC3 | -0.169763 | 0.00010018 | 0.00031923 |
| RTBDN | 0.1697381 | 0.00010042 | 0.00031994 |
| IGFN1 | -0.169734 | 0.00010046 | 0.00032002 |
| INPP4B | -0.1697291 | 0.00010051 | 0.00032012 |
| P2RX6 | -0.1696868 | 0.00010092 | 0.00032137 |
| HNRNPA0 | 0.1696817 | 0.00010097 | 0.00032147 |
| SFRS5 | 0.169663 | 0.00010115 | 0.000322 |
| SELS | 0.1696311 | 0.00010146 | 0.00032293 |
| STK35 | -0.1695957 | 0.0001018 | 0.00032395 |
| COPS4 | 0.169595 | 0.00010181 | 0.00032395 |
| FCGR2B | -0.1695867 | 0.00010189 | 0.00032415 |
| FAM13A | -0.1695424 | 0.00010232 | 0.00032548 |
| ITGA2B | 0.1695265 | 0.00010248 | 0.00032593 |
| APOLD1 | -0.1694264 | 0.00010346 | 0.00032901 |
| TCTEX1D4 | -0.1693987 | 0.00010374 | 0.00032983 |
| TCHH | -0.1693891 | 0.00010383 | 0.00033008 |
| RREB1 | -0.16937 | 0.00010402 | 0.00033063 |
| RBM43 | -0.1693416 | 0.00010431 | 0.00033148 |
| INPP4A | -0.1693318 | 0.0001044 | 0.00033174 |
| GLI1 | -0.1693204 | 0.00010452 | 0.00033205 |
| ANGEL2 | 0.1693187 | 0.00010454 | 0.00033205 |
| REXO2 | -0.1692822 | 0.0001049 | 0.00033316 |
| NEK10 | -0.1692658 | 0.00010506 | 0.00033363 |
| IQUB | -0.1692582 | 0.00010514 | 0.00033382 |
| PRDX1 | 0.1691999 | 0.00010573 | 0.00033563 |
| GJA10 | 0.1691959 | 0.00010577 | 0.0003357 |
| LOC652276 | -0.1691793 | 0.00010594 | 0.00033619 |
| TBC1D2B | -0.1691644 | 0.00010609 | 0.00033661 |
| ACTR3 | -0.169064 | 0.00010711 | 0.0003398 |
| EPHB1 | -0.1690607 | 0.00010714 | 0.00033985 |
| NACC2 | -0.1690384 | 0.00010737 | 0.00034052 |
| SLC13A3 | -0.1690312 | 0.00010745 | 0.0003407 |
| GPATCH4 | 0.1690026 | 0.00010774 | 0.00034158 |
| IL10 | -0.1690007 | 0.00010776 | 0.00034159 |
| NFATC3 | -0.1689878 | 0.00010789 | 0.00034196 |
| SLC35E2 | -0.1689853 | 0.00010792 | 0.00034198 |
| FMNL3 | -0.1689765 | 0.00010801 | 0.00034222 |
| C3orf54 | 0.1689496 | 0.00010829 | 0.00034304 |
| UBE2J1 | -0.1688996 | 0.0001088 | 0.00034463 |
| PTPRU | -0.1688258 | 0.00010957 | 0.00034701 |
| PTPN12 | -0.16879 | 0.00010995 | 0.00034814 |
| POLR1A | -0.1687812 | 0.00011004 | 0.00034838 |
| LOC100272146 | 0.1687695 | 0.00011016 | 0.00034871 |
| TES | -0.1687265 | 0.00011061 | 0.00035009 |
| HTATSF1 | 0.1687131 | 0.00011075 | 0.00035048 |
| TET2 | -0.1686851 | 0.00011105 | 0.00035133 |
| SGPL1 | -0.1686844 | 0.00011106 | 0.00035133 |
| ZNF673 | 0.1686685 | 0.00011123 | 0.00035181 |
| PHF20 | -0.1686552 | 0.00011137 | 0.0003522 |
| FBN2 | -0.1686363 | 0.00011157 | 0.00035278 |
| TAAR8 | 0.1686261 | 0.00011168 | 0.00035306 |
| HHAT | -0.1686238 | 0.0001117 | 0.00035308 |
| C9orf82 | 0.1686218 | 0.00011172 | 0.0003531 |
| NTRK3 | -0.1686174 | 0.00011177 | 0.00035319 |
| ARL17A | 0.1686139 | 0.00011181 | 0.00035325 |
| PTTG1IP | -0.1686041 | 0.00011191 | 0.00035353 |
| ST6GALNAC5 | -0.1685885 | 0.00011208 | 0.00035399 |
| CHCHD2 | 0.168587 | 0.00011209 | 0.00035399 |
| PARP6 | 0.1685806 | 0.00011216 | 0.00035415 |
| MST1P9 | 0.1685787 | 0.00011218 | 0.00035416 |
| BCAM | -0.1685588 | 0.00011239 | 0.00035477 |
| EHD1 | -0.1685456 | 0.00011253 | 0.00035516 |
| WNT3 | -0.168544 | 0.00011255 | 0.00035516 |
| ZNF624 | -0.168538 | 0.00011262 | 0.00035531 |
| ATP8A1 | -0.1685222 | 0.00011279 | 0.00035576 |
| ING3 | 0.1685215 | 0.00011279 | 0.00035576 |
| GUSBP3 | 0.1685194 | 0.00011282 | 0.00035577 |
| TUT1 | 0.1685004 | 0.00011302 | 0.00035636 |
| MED19 | 0.1684891 | 0.00011314 | 0.00035669 |
| ARIH2 | 0.1684795 | 0.00011324 | 0.00035696 |
| NIN | -0.1684617 | 0.00011344 | 0.00035751 |
| LILRA1 | -0.1684301 | 0.00011378 | 0.00035853 |
| CABP5 | 0.1684058 | 0.00011404 | 0.0003593 |
| MSN | -0.168385 | 0.00011427 | 0.00035996 |
| SLC16A8 | 0.1683715 | 0.00011441 | 0.00036036 |
| ITIH4 | -0.1683521 | 0.00011462 | 0.00036097 |
| IL23A | 0.1683427 | 0.00011473 | 0.00036122 |
| EPHB2 | -0.1683417 | 0.00011474 | 0.00036122 |
| GOLGA8B | 0.1682063 | 0.00011622 | 0.00036584 |
| UHMK1 | -0.1681849 | 0.00011646 | 0.00036652 |
| GRAMD1B | -0.1681763 | 0.00011656 | 0.00036676 |
| ZC3HAV1L | -0.168162 | 0.00011671 | 0.00036721 |
| ANKRD12 | -0.1680986 | 0.00011742 | 0.00036937 |
| POLR2E | 0.1680875 | 0.00011754 | 0.0003697 |
| SGCE | -0.168071 | 0.00011773 | 0.00037022 |
| TCF21 | -0.1680485 | 0.00011798 | 0.00037095 |
| TAF6 | 0.1680305 | 0.00011818 | 0.00037153 |
| COG1 | -0.1680058 | 0.00011846 | 0.00037234 |
| MATN3 | -0.1679998 | 0.00011852 | 0.0003725 |
| SLITRK2 | -0.1679874 | 0.00011867 | 0.00037288 |
| ATG12 | 0.1679802 | 0.00011875 | 0.00037307 |
| LZTR1 | -0.1679367 | 0.00011924 | 0.00037456 |
| C14orf162 | 0.1679211 | 0.00011941 | 0.00037505 |
| KIAA1632 | -0.1679167 | 0.00011946 | 0.00037515 |
| ACSM5 | -0.1678843 | 0.00011983 | 0.00037625 |
| MSR1 | -0.1678727 | 0.00011996 | 0.00037661 |
| CTTNBP2 | -0.1678436 | 0.00012029 | 0.00037758 |
| HSBP1 | 0.1678071 | 0.00012071 | 0.00037883 |
| PALB2 | 0.1678004 | 0.00012079 | 0.00037901 |
| KLC3 | -0.1677232 | 0.00012167 | 0.00038174 |
| SLC38A3 | -0.1677201 | 0.00012171 | 0.00038179 |
| KIAA1161 | -0.1677064 | 0.00012187 | 0.00038223 |
| CEACAM6 | -0.1676907 | 0.00012205 | 0.0003827 |
| GNG7 | -0.16769 | 0.00012206 | 0.0003827 |
| MFSD8 | 0.1676841 | 0.00012213 | 0.00038286 |
| RABL5 | 0.1676686 | 0.0001223 | 0.00038336 |
| SLC45A2 | -0.1676525 | 0.00012249 | 0.00038389 |
| NCCRP1 | -0.167647 | 0.00012256 | 0.00038403 |
| ZIC5 | 0.1676012 | 0.00012309 | 0.00038563 |
| INPPL1 | -0.1675638 | 0.00012353 | 0.00038694 |
| NKX3-1 | 0.1675411 | 0.00012379 | 0.00038772 |
| NPHP4 | -0.1675267 | 0.00012396 | 0.00038818 |
| MAB21L1 | -0.1675147 | 0.0001241 | 0.00038856 |
| VENTXP7 | 0.1675087 | 0.00012417 | 0.00038867 |
| CRLF1 | -0.1675085 | 0.00012417 | 0.00038867 |
| RNU11 | 0.1674967 | 0.00012431 | 0.00038903 |
| EDAR | -0.1674956 | 0.00012432 | 0.00038903 |
| KRT14 | -0.1674928 | 0.00012436 | 0.00038907 |
| CGNL1 | -0.1674856 | 0.00012444 | 0.00038927 |
| FAM162A | 0.1674805 | 0.0001245 | 0.0003894 |
| TIGD2 | 0.167471 | 0.00012461 | 0.00038969 |
| GREB1L | -0.1674675 | 0.00012466 | 0.00038976 |
| TMED1 | 0.1674317 | 0.00012508 | 0.00039098 |
| BCAT1 | -0.167431 | 0.00012509 | 0.00039098 |
| DBI | 0.1674236 | 0.00012517 | 0.0003912 |
| SIAH1 | -0.167397 | 0.00012549 | 0.00039212 |
| C8orf48 | -0.1673904 | 0.00012557 | 0.00039231 |
| PM20D2 | 0.1673288 | 0.0001263 | 0.00039454 |
| AFG3L1 | 0.1673235 | 0.00012636 | 0.00039467 |
| NKX6-1 | -0.1672761 | 0.00012693 | 0.00039638 |
| TEX264 | 0.1672602 | 0.00012712 | 0.00039692 |
| CBR3 | 0.1672452 | 0.0001273 | 0.00039742 |
| TRPC3 | -0.1672319 | 0.00012746 | 0.00039786 |
| TMED6 | 0.1672299 | 0.00012749 | 0.00039787 |
| ORC2L | 0.1672279 | 0.00012751 | 0.00039788 |
| ENOX1 | -0.1672242 | 0.00012755 | 0.00039796 |
| FLNB | -0.1672149 | 0.00012767 | 0.00039825 |
| WASH3P | 0.1671868 | 0.00012801 | 0.00039925 |
| PMFBP1 | -0.1671314 | 0.00012868 | 0.00040128 |
| SPHAR | 0.1671181 | 0.00012884 | 0.00040172 |
| LIN7A | -0.1671071 | 0.00012897 | 0.00040207 |
| CHKA | 0.1671056 | 0.00012899 | 0.00040207 |
| ZBTB10 | -0.1670871 | 0.00012922 | 0.00040271 |
| KIAA1033 | -0.1670733 | 0.00012939 | 0.00040317 |
| TCEA1 | 0.1670627 | 0.00012951 | 0.00040351 |
| TMEM106B | -0.1670521 | 0.00012965 | 0.00040381 |
| SLC9A9 | -0.1670516 | 0.00012965 | 0.00040381 |
| EIF3A | -0.1670287 | 0.00012993 | 0.00040462 |
| B3GNT2 | -0.1670268 | 0.00012995 | 0.00040463 |
| ZMYM4 | -0.1670151 | 0.0001301 | 0.00040502 |
| HMGXB3 | -0.1669803 | 0.00013053 | 0.00040629 |
| CKMT1B | 0.1669652 | 0.00013071 | 0.0004068 |
| PELI2 | -0.1669602 | 0.00013077 | 0.00040693 |
| ACOX3 | -0.1668863 | 0.00013169 | 0.00040968 |
| LOC100286793 | 0.1668856 | 0.0001317 | 0.00040968 |
| SNORD115-26 | -0.1668634 | 0.00013197 | 0.00041047 |
| THAP4 | 0.1668403 | 0.00013226 | 0.00041131 |
| STAC2 | -0.1668317 | 0.00013237 | 0.00041158 |
| MAP7 | -0.1668253 | 0.00013245 | 0.00041176 |
| UCN | 0.1668204 | 0.00013251 | 0.00041189 |
| HSH2D | 0.1668009 | 0.00013275 | 0.00041258 |
| VSIG8 | -0.1667829 | 0.00013298 | 0.00041322 |
| NUDT21 | 0.166778 | 0.00013304 | 0.00041335 |
| CSAD | 0.1667698 | 0.00013314 | 0.0004136 |
| SNORA63 | 0.166751 | 0.00013338 | 0.00041427 |
| CHN1 | -0.1667469 | 0.00013343 | 0.00041437 |
| MOBKL1A | -0.1667019 | 0.000134 | 0.00041606 |
| ZFAND1 | 0.1666766 | 0.00013432 | 0.00041699 |
| KREMEN2 | 0.1666118 | 0.00013514 | 0.00041948 |
| PROM2 | -0.1666076 | 0.00013519 | 0.00041958 |
| C14orf174 | -0.1666026 | 0.00013525 | 0.00041967 |
| RABEPK | 0.1666019 | 0.00013526 | 0.00041967 |
| LDLRAD2 | -0.1665832 | 0.0001355 | 0.00042035 |
| ANKIB1 | -0.166517 | 0.00013635 | 0.00042287 |
| MRS2P2 | 0.1665165 | 0.00013636 | 0.00042287 |
| HPCA | 0.1664769 | 0.00013686 | 0.00042438 |
| DTNB | 0.166456 | 0.00013713 | 0.00042515 |
| FSD2 | -0.1664492 | 0.00013722 | 0.00042535 |
| AKR1A1 | 0.1664253 | 0.00013753 | 0.00042625 |
| RBP4 | -0.1664141 | 0.00013768 | 0.00042663 |
| PTGDR | -0.1663595 | 0.00013838 | 0.00042876 |
| CHST2 | -0.1663368 | 0.00013868 | 0.00042961 |
| SIGLEC15 | -0.1663251 | 0.00013883 | 0.00043002 |
| NBPF16 | -0.1663214 | 0.00013888 | 0.0004301 |
| UCKL1 | 0.1662946 | 0.00013923 | 0.00043112 |
| IDO1 | 0.1662879 | 0.00013932 | 0.00043132 |
| SEMA5B | -0.1662293 | 0.00014009 | 0.00043359 |
| OR4D5 | 0.1662287 | 0.00014009 | 0.00043359 |
| GTF2H5 | 0.1661914 | 0.00014059 | 0.00043505 |
| CDK7 | 0.1661571 | 0.00014104 | 0.00043639 |
| 15-Sep | 0.1661447 | 0.0001412 | 0.00043683 |
| KIAA1715 | -0.1661426 | 0.00014123 | 0.00043685 |
| ARHGAP4 | 0.1661357 | 0.00014132 | 0.00043706 |
| ENHO | 0.1661254 | 0.00014146 | 0.00043742 |
| ABCB10 | 0.1661164 | 0.00014158 | 0.00043772 |
| XPA | 0.1661035 | 0.00014175 | 0.00043819 |
| ZBTB7B | -0.1660902 | 0.00014193 | 0.00043866 |
| ACP6 | 0.1660622 | 0.0001423 | 0.00043975 |
| OR56B4 | -0.1660536 | 0.00014242 | 0.00044004 |
| ATP2C2 | -0.1660263 | 0.00014278 | 0.0004411 |
| CCDC61 | 0.1660109 | 0.00014299 | 0.00044167 |
| LOC729603 | -0.165994 | 0.00014322 | 0.0004423 |
| GRK4 | -0.165989 | 0.00014328 | 0.00044244 |
| PCDHGB8P | -0.1659786 | 0.00014342 | 0.00044281 |
| TUBA4A | -0.165905 | 0.00014442 | 0.00044581 |
| EPOR | 0.1658882 | 0.00014464 | 0.00044644 |
| KLK14 | -0.1658645 | 0.00014497 | 0.00044727 |
| LOC401463 | -0.165864 | 0.00014497 | 0.00044727 |
| ITM2B | -0.1658635 | 0.00014498 | 0.00044727 |
| UBE2Q2P1 | -0.1658543 | 0.0001451 | 0.00044758 |
| UBE3C | -0.1658321 | 0.00014541 | 0.00044845 |
| IGFL4 | -0.1657669 | 0.0001463 | 0.00045113 |
| PTH2 | 0.1657179 | 0.00014697 | 0.00045313 |
| S1PR3 | -0.1657108 | 0.00014707 | 0.00045337 |
| MMP23B | -0.1656846 | 0.00014743 | 0.00045436 |
| LGI4 | -0.1656842 | 0.00014743 | 0.00045436 |
| TMSL3 | 0.1656725 | 0.0001476 | 0.00045479 |
| MRPS10 | 0.1656622 | 0.00014774 | 0.00045515 |
| C6orf115 | 0.1656299 | 0.00014819 | 0.00045646 |
| TGM6 | -0.1655993 | 0.00014861 | 0.00045771 |
| STAG3L3 | 0.1655914 | 0.00014872 | 0.00045797 |
| ASCL2 | 0.1655531 | 0.00014925 | 0.00045955 |
| LOXL1 | -0.165541 | 0.00014942 | 0.00046 |
| CTCFL | 0.1655352 | 0.0001495 | 0.00046018 |
| GLT25D2 | -0.165531 | 0.00014956 | 0.00046029 |
| AMBRA1 | -0.1654754 | 0.00015034 | 0.00046262 |
| KIAA1614 | -0.1654323 | 0.00015095 | 0.00046441 |
| MGAM | -0.1654303 | 0.00015098 | 0.00046443 |
| RNF148 | 0.1654247 | 0.00015106 | 0.0004646 |
| AUTS2 | -0.1654074 | 0.0001513 | 0.00046528 |
| YIPF2 | -0.1653695 | 0.00015184 | 0.00046686 |
| CEMP1 | 0.1653678 | 0.00015186 | 0.00046687 |
| STRADB | 0.1653609 | 0.00015196 | 0.0004671 |
| EAF2 | 0.165348 | 0.00015214 | 0.00046759 |
| ALOXE3 | -0.165332 | 0.00015237 | 0.00046822 |
| ZNF672 | 0.1653143 | 0.00015262 | 0.00046892 |
| ACHE | -0.1652998 | 0.00015283 | 0.00046948 |
| PLCG1 | -0.1652936 | 0.00015292 | 0.00046964 |
| ZNF670 | 0.1652929 | 0.00015293 | 0.00046964 |
| SETD8 | 0.1652832 | 0.00015307 | 0.00047 |
| SNORA10 | 0.1652792 | 0.00015313 | 0.0004701 |
| PTX3 | -0.1651977 | 0.0001543 | 0.00047362 |
| CBWD3 | 0.1651896 | 0.00015441 | 0.00047391 |
| EID2B | 0.1651808 | 0.00015454 | 0.00047423 |
| KCNG4 | 0.1651577 | 0.00015487 | 0.00047518 |
| KLK12 | -0.1651542 | 0.00015492 | 0.00047526 |
| KHSRP | 0.1650678 | 0.00015618 | 0.00047904 |
| TRMT2A | 0.1650656 | 0.00015621 | 0.00047906 |
| LUZP2 | -0.1650069 | 0.00015707 | 0.00048162 |
| STAC3 | -0.1649993 | 0.00015718 | 0.00048188 |
| CTSB | -0.1649498 | 0.00015791 | 0.00048404 |
| C20orf107 | 0.1649303 | 0.00015819 | 0.00048485 |
| SOST | -0.1649286 | 0.00015822 | 0.00048485 |
| STK38L | -0.1649025 | 0.0001586 | 0.00048596 |
| CBFA2T3 | -0.1648873 | 0.00015883 | 0.00048657 |
| C8orf34 | -0.1648657 | 0.00015915 | 0.00048748 |
| POLL | 0.1648626 | 0.0001592 | 0.00048755 |
| IL28A | 0.1648463 | 0.00015944 | 0.00048821 |
| CLGN | 0.1648293 | 0.00015969 | 0.00048891 |
| C3orf19 | 0.1648083 | 0.00016 | 0.0004898 |
| EPPK1 | -0.1647605 | 0.00016072 | 0.00049191 |
| JAKMIP3 | -0.164722 | 0.00016129 | 0.0004936 |
| BCL11B | -0.1646855 | 0.00016184 | 0.00049521 |
| GON4L | -0.1646683 | 0.0001621 | 0.00049592 |
| FAM45B | -0.1646648 | 0.00016216 | 0.00049601 |
| C19orf60 | 0.1646505 | 0.00016237 | 0.0004966 |
| ZBTB37 | -0.164636 | 0.00016259 | 0.00049719 |
| SETMAR | 0.1646166 | 0.00016288 | 0.00049802 |
| GRK5 | -0.1646059 | 0.00016305 | 0.00049843 |
| TBP | 0.1645951 | 0.00016321 | 0.00049886 |
| ISG20 | 0.1645864 | 0.00016334 | 0.00049916 |
| CYSLTR2 | -0.1645853 | 0.00016336 | 0.00049916 |
| SALL4 | -0.1645828 | 0.0001634 | 0.0004992 |
| GRHPR | 0.1645756 | 0.00016351 | 0.0004994 |
| ERAS | 0.1645754 | 0.00016351 | 0.0004994 |
| ZNF792 | -0.1644879 | 0.00016485 | 0.0005034 |
| NKPD1 | -0.1644771 | 0.00016501 | 0.00050383 |
| WBSCR17 | -0.1644522 | 0.00016539 | 0.00050492 |
| ZNF587 | -0.164431 | 0.00016572 | 0.00050584 |
| TRAPPC3 | 0.1644247 | 0.00016582 | 0.00050606 |
| MTX2 | 0.1644171 | 0.00016594 | 0.00050634 |
| GPR89A | 0.1644143 | 0.00016598 | 0.0005064 |
| ZNF625 | -0.1643855 | 0.00016642 | 0.00050768 |
| PRCP | -0.1643733 | 0.00016661 | 0.00050818 |
| NKD2 | -0.1643264 | 0.00016734 | 0.00051026 |
| PKM2 | 0.1643261 | 0.00016734 | 0.00051026 |
| SLCO1C1 | -0.1643035 | 0.0001677 | 0.00051125 |
| TOMM20L | 0.1642416 | 0.00016866 | 0.00051412 |
| ZBTB48 | 0.1642025 | 0.00016928 | 0.00051591 |
| GAK | -0.1641739 | 0.00016973 | 0.00051721 |
| COL4A5 | -0.1641537 | 0.00017005 | 0.0005181 |
| CEP164 | -0.1641376 | 0.0001703 | 0.0005188 |
| HIST2H4A | 0.1641353 | 0.00017034 | 0.00051883 |
| CTBP2 | 0.1641306 | 0.00017041 | 0.00051898 |
| SYNJ2 | -0.1640826 | 0.00017117 | 0.00052121 |
| BRSK1 | -0.1640154 | 0.00017224 | 0.00052439 |
| REV3L | -0.1639956 | 0.00017256 | 0.00052528 |
| C4orf26 | -0.1639577 | 0.00017316 | 0.00052705 |
| KDM4A | -0.1639311 | 0.00017359 | 0.00052827 |
| ZNF503 | -0.1638751 | 0.0001745 | 0.00053094 |
| DND1 | 0.1638078 | 0.00017559 | 0.00053418 |
| CXCL10 | 0.1637919 | 0.00017585 | 0.00053488 |
| SNORA7B | 0.1637807 | 0.00017603 | 0.00053536 |
| CLDN10 | 0.1637726 | 0.00017616 | 0.00053568 |
| RMND5B | 0.1637379 | 0.00017673 | 0.00053733 |
| SLC38A2 | -0.163708 | 0.00017722 | 0.00053873 |
| NF1 | -0.1636956 | 0.00017742 | 0.00053927 |
| GLOD5 | 0.163691 | 0.0001775 | 0.00053942 |
| MDK | 0.163683 | 0.00017763 | 0.0005397 |
| LOC92249 | -0.163682 | 0.00017764 | 0.0005397 |
| ANKRD52 | -0.16368 | 0.00017768 | 0.00053972 |
| FZD10 | -0.1636348 | 0.00017842 | 0.0005419 |
| DENND2D | 0.1636036 | 0.00017894 | 0.00054339 |
| TLE4 | -0.1635799 | 0.00017933 | 0.0005445 |
| CNGA1 | -0.1635772 | 0.00017937 | 0.00054455 |
| FGF13 | -0.1635704 | 0.00017949 | 0.00054481 |
| SEC22A | 0.163558 | 0.00017969 | 0.00054536 |
| C12orf27 | 0.1635379 | 0.00018003 | 0.00054629 |
| C21orf67 | 0.1635334 | 0.0001801 | 0.00054644 |
| TBR1 | 0.1635042 | 0.00018059 | 0.00054783 |
| EDN3 | -0.1634956 | 0.00018073 | 0.00054818 |
| TMED4 | -0.1634777 | 0.00018103 | 0.00054901 |
| TNNI2 | -0.1634698 | 0.00018117 | 0.00054933 |
| FAM40A | -0.1634641 | 0.00018126 | 0.00054953 |
| DDX3X | -0.1634531 | 0.00018145 | 0.00055001 |
| SMG1 | -0.1634188 | 0.00018202 | 0.00055168 |
| PCDP1 | -0.1633889 | 0.00018253 | 0.00055312 |
| RASGRP3 | -0.163381 | 0.00018266 | 0.00055344 |
| TXNL1 | 0.1633764 | 0.00018274 | 0.00055359 |
| NAIF1 | 0.1633741 | 0.00018278 | 0.00055363 |
| ZADH2 | 0.1633431 | 0.0001833 | 0.00055513 |
| FOXF2 | -0.1633279 | 0.00018356 | 0.00055583 |
| SLC22A3 | -0.1632926 | 0.00018416 | 0.00055756 |
| UBR1 | -0.1632868 | 0.00018425 | 0.00055777 |
| PRNP | -0.1632733 | 0.00018449 | 0.00055839 |
| LOC440563 | 0.1632716 | 0.00018451 | 0.00055839 |
| TAF1L | -0.1632044 | 0.00018566 | 0.00056178 |
| CEP68 | -0.1631943 | 0.00018584 | 0.00056222 |
| FSTL3 | -0.1631403 | 0.00018676 | 0.00056494 |
| LENG9 | 0.1631301 | 0.00018694 | 0.00056539 |
| PCMT1 | 0.1631138 | 0.00018722 | 0.00056616 |
| QRSL1 | 0.1630783 | 0.00018784 | 0.00056793 |
| SCEL | -0.1630409 | 0.00018848 | 0.00056981 |
| C1RL | -0.1630201 | 0.00018885 | 0.00057081 |
| ZNF165 | 0.1630067 | 0.00018908 | 0.00057143 |
| ATG2A | -0.1629964 | 0.00018926 | 0.00057189 |
| MAPK3 | -0.1629726 | 0.00018967 | 0.00057306 |
| PARS2 | 0.1629587 | 0.00018992 | 0.00057371 |
| MVK | 0.1629185 | 0.00019062 | 0.00057575 |
| PDCD4 | -0.1629169 | 0.00019065 | 0.00057575 |
| SPTBN2 | -0.162912 | 0.00019074 | 0.00057579 |
| SEMA3G | -0.1629114 | 0.00019075 | 0.00057579 |
| SLC31A2 | -0.1629112 | 0.00019075 | 0.00057579 |
| KRTCAP3 | 0.162909 | 0.00019079 | 0.00057583 |
| CSF2RB | -0.1629045 | 0.00019087 | 0.00057598 |
| ANKRD44 | -0.1628854 | 0.0001912 | 0.0005769 |
| KLHL4 | -0.1628223 | 0.00019232 | 0.00058019 |
| ZNF76 | 0.1628165 | 0.00019242 | 0.00058041 |
| RFX5 | 0.162805 | 0.00019263 | 0.00058094 |
| MICAL3 | -0.1627818 | 0.00019304 | 0.00058209 |
| PSMD14 | 0.1627782 | 0.0001931 | 0.0005822 |
| POLR3F | 0.1627677 | 0.00019329 | 0.00058267 |
| MTMR9 | -0.1627284 | 0.00019399 | 0.0005847 |
| C17orf49 | 0.1627154 | 0.00019422 | 0.00058531 |
| ZSCAN4 | -0.1627105 | 0.00019431 | 0.00058549 |
| WFDC1 | -0.1626899 | 0.00019468 | 0.00058651 |
| ZNHIT1 | 0.1626811 | 0.00019484 | 0.00058689 |
| MEPE | -0.1626764 | 0.00019492 | 0.00058706 |
| PDE11A | -0.1626219 | 0.0001959 | 0.00058993 |
| PPP4C | 0.1625978 | 0.00019634 | 0.00059115 |
| RAG1AP1 | 0.1625955 | 0.00019638 | 0.00059118 |
| FAM48B2 | -0.1625728 | 0.00019679 | 0.00059228 |
| GABRR2 | -0.1625721 | 0.0001968 | 0.00059228 |
| RIOK1 | 0.1625624 | 0.00019698 | 0.00059272 |
| ANKRD53 | -0.162559 | 0.00019704 | 0.00059282 |
| PDE8B | -0.1625538 | 0.00019713 | 0.00059301 |
| NBL1 | -0.1625448 | 0.00019729 | 0.00059341 |
| C1orf172 | 0.1625333 | 0.0001975 | 0.00059389 |
| OR5C1 | 0.1625328 | 0.00019751 | 0.00059389 |
| GDE1 | -0.1624991 | 0.00019813 | 0.00059565 |
| PPP2R3B | 0.1624842 | 0.0001984 | 0.00059637 |
| PWRN2 | 0.162475 | 0.00019856 | 0.00059679 |
| ZFPM1 | -0.1624653 | 0.00019874 | 0.00059723 |
| PNPLA7 | -0.1624611 | 0.00019882 | 0.00059737 |
| TPPP | -0.162423 | 0.00019952 | 0.00059938 |
| C5orf56 | 0.1624094 | 0.00019977 | 0.00060004 |
| WDR75 | 0.1624052 | 0.00019984 | 0.00060014 |
| ARHGAP18 | -0.1624043 | 0.00019986 | 0.00060014 |
| MTMR6 | -0.1623748 | 0.0002004 | 0.00060168 |
| CCDC40 | -0.1623709 | 0.00020047 | 0.00060181 |
| C7orf61 | 0.1623245 | 0.00020133 | 0.00060429 |
| CAGE1 | 0.1623212 | 0.00020139 | 0.00060438 |
| RAVER1 | 0.1622844 | 0.00020207 | 0.00060634 |
| NPR1 | -0.1622336 | 0.00020302 | 0.00060908 |
| MED15 | -0.1622072 | 0.00020351 | 0.00061047 |
| KIAA0802 | -0.1621565 | 0.00020446 | 0.00061322 |
| OTUD6B | 0.1621382 | 0.0002048 | 0.00061416 |
| SLC7A5 | -0.1620225 | 0.00020699 | 0.00062061 |
| RBMXL1 | 0.1620211 | 0.00020701 | 0.00062061 |
| C14orf104 | 0.1620172 | 0.00020709 | 0.00062074 |
| ARSA | -0.1620077 | 0.00020727 | 0.00062117 |
| CALHM2 | -0.1620064 | 0.00020729 | 0.00062117 |
| PVT1 | 0.1619829 | 0.00020774 | 0.00062242 |
| INO80D | -0.1619807 | 0.00020778 | 0.00062245 |
| FCN1 | -0.1619656 | 0.00020807 | 0.00062322 |
| PPP2R2A | 0.1619467 | 0.00020843 | 0.00062421 |
| TMEFF1 | -0.1618971 | 0.00020938 | 0.00062696 |
| TOMM7 | 0.1618927 | 0.00020946 | 0.00062712 |
| TMEM8B | -0.1618886 | 0.00020954 | 0.00062726 |
| ZNF823 | 0.161841 | 0.00021046 | 0.0006299 |
| ABHD14B | 0.1618263 | 0.00021074 | 0.00063066 |
| ZG16 | 0.1618128 | 0.000211 | 0.00063134 |
| GDPD2 | -0.1617887 | 0.00021147 | 0.00063265 |
| TRRAP | -0.1617732 | 0.00021177 | 0.00063345 |
| CGB8 | -0.1617453 | 0.00021231 | 0.00063497 |
| FGF9 | -0.1617036 | 0.00021312 | 0.00063731 |
| COQ10A | 0.1616972 | 0.00021325 | 0.00063759 |
| C12orf68 | -0.1616487 | 0.00021419 | 0.00064033 |
| LAG3 | 0.1616424 | 0.00021432 | 0.0006406 |
| INTS4L1 | -0.1616181 | 0.0002148 | 0.00064193 |
| KDM2A | -0.1615819 | 0.00021551 | 0.00064397 |
| C1orf43 | 0.1615784 | 0.00021558 | 0.00064408 |
| CHRM4 | -0.16155 | 0.00021614 | 0.00064566 |
| RBPMS2 | -0.1615124 | 0.00021688 | 0.00064778 |
| BTBD11 | -0.1614851 | 0.00021742 | 0.00064931 |
| ZNRF3 | -0.161454 | 0.00021804 | 0.00065106 |
| CTNND1 | -0.1614156 | 0.00021881 | 0.00065325 |
| CNIH2 | 0.1614008 | 0.0002191 | 0.00065403 |
| NCRNA00204B | 0.1613846 | 0.00021943 | 0.00065491 |
| ZIM2 | -0.1613648 | 0.00021982 | 0.00065599 |
| ARFGAP3 | -0.1613552 | 0.00022002 | 0.00065647 |
| PRMT5 | 0.161325 | 0.00022063 | 0.00065812 |
| SPDYE8P | 0.1613246 | 0.00022063 | 0.00065812 |
| TM4SF18 | -0.1613142 | 0.00022084 | 0.00065864 |
| LOC100134259 | -0.1613114 | 0.0002209 | 0.00065871 |
| RALGPS2 | -0.1612927 | 0.00022128 | 0.00065974 |
| ABLIM3 | -0.1612599 | 0.00022194 | 0.00066162 |
| AQPEP | -0.1612438 | 0.00022227 | 0.0006625 |
| PITX3 | -0.1612372 | 0.0002224 | 0.0006628 |
| TCP11 | 0.1612215 | 0.00022272 | 0.00066365 |
| C1orf123 | 0.1612118 | 0.00022292 | 0.00066414 |
| LOC645332 | 0.1611974 | 0.00022321 | 0.00066492 |
| GAS1 | -0.1611848 | 0.00022347 | 0.00066558 |
| CXorf26 | 0.1611764 | 0.00022364 | 0.00066599 |
| COL6A6 | -0.1611691 | 0.00022379 | 0.00066634 |
| TUBB3 | -0.1611479 | 0.00022422 | 0.00066746 |
| SYNGAP1 | -0.1611474 | 0.00022423 | 0.00066746 |
| PHOSPHO2 | 0.1611406 | 0.00022437 | 0.00066778 |
| RAD21 | 0.1611185 | 0.00022482 | 0.00066899 |
| TNFAIP8 | 0.1611174 | 0.00022484 | 0.00066899 |
| SNAP47 | 0.1611062 | 0.00022507 | 0.00066958 |
| NCRNA00203 | 0.1610933 | 0.00022534 | 0.00067027 |
| SCARNA10 | 0.1610886 | 0.00022544 | 0.00067046 |
| IGFBP4 | -0.1610723 | 0.00022577 | 0.00067136 |
| KRBA1 | -0.1610535 | 0.00022616 | 0.00067241 |
| C22orf32 | 0.161037 | 0.0002265 | 0.00067332 |
| OR8D2 | 0.161027 | 0.00022671 | 0.00067377 |
| AARS2 | 0.1610264 | 0.00022672 | 0.00067377 |
| PHF6 | 0.1610167 | 0.00022692 | 0.00067427 |
| GALNT1 | -0.1610139 | 0.00022698 | 0.00067434 |
| ANKRD36 | 0.1609635 | 0.00022802 | 0.00067734 |
| ASB6 | 0.1609438 | 0.00022843 | 0.00067842 |
| AHSA1 | 0.1609428 | 0.00022845 | 0.00067842 |
| EPCAM | 0.1609132 | 0.00022907 | 0.00068007 |
| LOC441089 | 0.160913 | 0.00022907 | 0.00068007 |
| PRDX4 | 0.1609051 | 0.00022924 | 0.00068046 |
| C6orf81 | 0.160899 | 0.00022936 | 0.0006807 |
| SMURF2 | -0.160898 | 0.00022939 | 0.0006807 |
| ZNF746 | -0.1608577 | 0.00023023 | 0.0006831 |
| EIF4E | 0.1608371 | 0.00023066 | 0.00068428 |
| PBLD | -0.1607952 | 0.00023154 | 0.00068679 |
| KIF1A | -0.1607833 | 0.00023179 | 0.00068728 |
| POU5F2 | 0.1607826 | 0.00023181 | 0.00068728 |
| IL1F5 | -0.1607825 | 0.00023181 | 0.00068728 |
| TUFT1 | -0.1607795 | 0.00023187 | 0.00068738 |
| TMEM140 | -0.1607675 | 0.00023213 | 0.00068802 |
| RAVER2 | 0.1607639 | 0.0002322 | 0.00068814 |
| RASSF2 | -0.1607619 | 0.00023225 | 0.00068817 |
| DENND5A | -0.1607482 | 0.00023253 | 0.00068893 |
| KPTN | 0.1607431 | 0.00023264 | 0.00068914 |
| FEM1C | -0.1607183 | 0.00023317 | 0.0006906 |
| C4orf39 | -0.1606885 | 0.0002338 | 0.00069237 |
| FAM36A | 0.1606855 | 0.00023387 | 0.00069243 |
| LOC441204 | -0.1606844 | 0.00023389 | 0.00069243 |
| KLF6 | -0.1606773 | 0.00023404 | 0.00069277 |
| CHST11 | -0.1606752 | 0.00023408 | 0.0006928 |
| CEP135 | 0.1606613 | 0.00023438 | 0.00069358 |
| HDC | -0.1606309 | 0.00023503 | 0.0006954 |
| TTC7A | -0.1606184 | 0.0002353 | 0.00069609 |
| POP1 | 0.1605694 | 0.00023635 | 0.0006991 |
| PLIN3 | -0.1605409 | 0.00023696 | 0.0007008 |
| PROCA1 | 0.1605391 | 0.000237 | 0.00070081 |
| LANCL1 | -0.1605266 | 0.00023727 | 0.00070151 |
| ZNF799 | 0.1605201 | 0.00023741 | 0.00070182 |
| RXRA | -0.1605161 | 0.0002375 | 0.00070198 |
| RPS6KC1 | -0.1604994 | 0.00023786 | 0.00070294 |
| SLC6A4 | -0.1604904 | 0.00023805 | 0.0007034 |
| NEDD1 | 0.1604639 | 0.00023863 | 0.000705 |
| NFKBIL1 | 0.1604227 | 0.00023952 | 0.00070754 |
| UBXN1 | 0.1604182 | 0.00023962 | 0.00070766 |
| WDFY2 | -0.1604176 | 0.00023963 | 0.00070766 |
| RNF150 | -0.16038 | 0.00024045 | 0.00070998 |
| RPL18A | 0.160365 | 0.00024078 | 0.00071084 |
| ATG4C | 0.1603337 | 0.00024146 | 0.00071276 |
| EXOC4 | -0.1603151 | 0.00024187 | 0.00071386 |
| HOXA10 | 0.1602672 | 0.00024292 | 0.00071686 |
| PRKAG1 | 0.1602628 | 0.00024302 | 0.00071704 |
| LOC348840 | 0.1602573 | 0.00024314 | 0.0007173 |
| RAB27A | 0.1602541 | 0.00024321 | 0.0007174 |
| FAM116A | 0.1602496 | 0.00024331 | 0.00071759 |
| DENND5B | -0.1602443 | 0.00024343 | 0.00071783 |
| TSC2 | -0.1602154 | 0.00024407 | 0.00071961 |
| E2F6 | 0.1602117 | 0.00024415 | 0.00071974 |
| RAB21 | -0.1602001 | 0.00024441 | 0.0007204 |
| SPTLC2 | -0.1601814 | 0.00024482 | 0.00072151 |
| TMEM158 | -0.1601707 | 0.00024506 | 0.00072211 |
| BOLA2 | 0.1601599 | 0.0002453 | 0.00072271 |
| DIRAS2 | -0.160153 | 0.00024545 | 0.00072306 |
| ONECUT2 | 0.1600881 | 0.0002469 | 0.00072722 |
| KRTAP5-9 | 0.1600564 | 0.00024761 | 0.0007292 |
| KIF6 | -0.1600204 | 0.00024842 | 0.00073148 |
| SLC30A2 | -0.1599859 | 0.0002492 | 0.00073366 |
| GAPVD1 | -0.1599637 | 0.0002497 | 0.00073503 |
| CHI3L2 | -0.1599614 | 0.00024975 | 0.00073508 |
| RHOU | -0.1599582 | 0.00024983 | 0.00073519 |
| ACTN3 | -0.1599536 | 0.00024993 | 0.00073539 |
| G6PC3 | 0.1599473 | 0.00025007 | 0.00073568 |
| TRPM4 | -0.159946 | 0.0002501 | 0.00073568 |
| TMIGD2 | 0.1599412 | 0.00025021 | 0.00073589 |
| NUDCD1 | 0.1599346 | 0.00025036 | 0.00073613 |
| IFITM4P | 0.1599344 | 0.00025036 | 0.00073613 |
| DHX29 | -0.1599226 | 0.00025063 | 0.00073681 |
| DPF3 | -0.1598875 | 0.00025143 | 0.00073904 |
| PLSCR1 | 0.1598775 | 0.00025166 | 0.00073961 |
| TADA2A | 0.1598745 | 0.00025173 | 0.0007397 |
| CTNND2 | -0.1598686 | 0.00025186 | 0.00073999 |
| INTS4L2 | -0.1598639 | 0.00025197 | 0.00074019 |
| LRRC39 | -0.1598395 | 0.00025253 | 0.00074172 |
| TNFSF14 | -0.159836 | 0.00025261 | 0.00074185 |
| PPM1A | -0.1597896 | 0.00025367 | 0.00074486 |
| KLHDC3 | 0.1597859 | 0.00025375 | 0.000745 |
| CDK19 | -0.1597772 | 0.00025395 | 0.00074548 |
| CD59 | -0.1597188 | 0.0002553 | 0.00074932 |
| ASRGL1 | 0.1596964 | 0.00025582 | 0.00075073 |
| APEH | 0.1596847 | 0.00025609 | 0.00075142 |
| PRR23C | 0.1596716 | 0.00025639 | 0.0007522 |
| TMPRSS11F | -0.1596603 | 0.00025665 | 0.00075285 |
| ZPLD1 | -0.1595897 | 0.0002583 | 0.00075757 |
| DDHD1 | -0.1595463 | 0.00025931 | 0.00076043 |
| GSTP1 | 0.1595445 | 0.00025935 | 0.00076044 |
| LOC253724 | 0.1595342 | 0.00025959 | 0.00076104 |
| MDN1 | -0.1595072 | 0.00026023 | 0.00076279 |
| DNAJB14 | -0.1594818 | 0.00026083 | 0.00076444 |
| PDCD6IP | -0.1594538 | 0.00026149 | 0.00076626 |
| SLC10A6 | -0.159446 | 0.00026167 | 0.00076669 |
| GRASP | -0.1593982 | 0.0002628 | 0.00076989 |
| CCDC83 | 0.1593784 | 0.00026327 | 0.00077115 |
| FAM55B | -0.1593564 | 0.0002638 | 0.00077258 |
| SIRT2 | -0.1593398 | 0.00026419 | 0.00077362 |
| DCAF6 | -0.1593369 | 0.00026426 | 0.00077367 |
| RDX | -0.1593359 | 0.00026428 | 0.00077367 |
| C6orf192 | 0.1592752 | 0.00026574 | 0.00077781 |
| GJA4 | -0.1592675 | 0.00026592 | 0.00077823 |
| ANKRD40 | -0.1592418 | 0.00026654 | 0.00077992 |
| PTPN11 | -0.1591665 | 0.00026835 | 0.00078504 |
| COL29A1 | -0.159166 | 0.00026836 | 0.00078504 |
| ZNF180 | 0.1591422 | 0.00026894 | 0.00078662 |
| SDHAF2 | 0.1591335 | 0.00026915 | 0.00078712 |
| SPIC | 0.1591216 | 0.00026944 | 0.00078785 |
| RAB34 | 0.159099 | 0.00026999 | 0.00078934 |
| ATN1 | -0.1590583 | 0.00027098 | 0.0007921 |
| STRC | 0.1590571 | 0.00027101 | 0.0007921 |
| LOC100190986 | -0.1590457 | 0.00027129 | 0.0007928 |
| PRPF38B | 0.1590375 | 0.00027149 | 0.00079327 |
| KLHL20 | -0.1590293 | 0.00027169 | 0.00079374 |
| GTPBP4 | 0.1590196 | 0.00027193 | 0.00079425 |
| ATXN2 | -0.159019 | 0.00027194 | 0.00079425 |
| EPYC | -0.1590072 | 0.00027223 | 0.00079494 |
| TMEM185A | -0.1590061 | 0.00027226 | 0.00079494 |
| CCDC78 | 0.1589891 | 0.00027268 | 0.00079604 |
| TRIM56 | -0.1589699 | 0.00027315 | 0.0007973 |
| ABHD2 | -0.1588999 | 0.00027488 | 0.00080223 |
| CEBPD | 0.1588425 | 0.0002763 | 0.00080627 |
| 7-Mar | -0.1588259 | 0.00027671 | 0.00080736 |
| PLRG1 | 0.158798 | 0.00027741 | 0.00080927 |
| SYT15 | -0.1587947 | 0.00027749 | 0.00080939 |
| ZNF471 | -0.158779 | 0.00027788 | 0.00081042 |
| MST1R | 0.1587499 | 0.00027861 | 0.00081242 |
| GALNTL6 | -0.1587464 | 0.0002787 | 0.00081257 |
| ARSJ | -0.158729 | 0.00027914 | 0.00081372 |
| NECAB2 | -0.1587115 | 0.00027957 | 0.00081488 |
| STAM2 | -0.158704 | 0.00027976 | 0.00081532 |
| MAT2B | 0.1586671 | 0.00028069 | 0.00081791 |
| INTS6 | -0.1586417 | 0.00028134 | 0.00081966 |
| FCAR | -0.1586226 | 0.00028182 | 0.00082095 |
| STT3B | -0.1586154 | 0.000282 | 0.00082136 |
| C8orf31 | -0.1586053 | 0.00028226 | 0.00082198 |
| TFRC | 0.1585502 | 0.00028366 | 0.00082595 |
| RRS1 | 0.1585231 | 0.00028435 | 0.00082784 |
| NKAIN1 | -0.1584921 | 0.00028514 | 0.00082998 |
| MRS2 | 0.1584911 | 0.00028517 | 0.00082998 |
| GALNTL4 | -0.1584407 | 0.00028646 | 0.00083363 |
| DFNB59 | 0.1584366 | 0.00028657 | 0.00083381 |
| SOCS3 | -0.1584335 | 0.00028664 | 0.00083392 |
| SPIRE1 | -0.1584204 | 0.00028698 | 0.00083478 |
| GP5 | -0.1584045 | 0.00028739 | 0.00083586 |
| C6 | -0.1583643 | 0.00028843 | 0.00083876 |
| MANBAL | 0.1583462 | 0.0002889 | 0.00084 |
| SLC4A7 | -0.1583343 | 0.00028921 | 0.00084078 |
| METTL1 | 0.1583147 | 0.00028972 | 0.00084213 |
| ST6GALNAC6 | -0.1582928 | 0.00029029 | 0.00084367 |
| CARD14 | -0.1582843 | 0.00029051 | 0.00084419 |
| ZNF675 | -0.1582564 | 0.00029124 | 0.00084618 |
| THSD7B | -0.1582211 | 0.00029216 | 0.00084874 |
| GPR21 | -0.1581927 | 0.00029291 | 0.00085078 |
| POLDIP2 | 0.1581752 | 0.00029336 | 0.000852 |
| PLEKHA5 | -0.1581634 | 0.00029368 | 0.00085274 |
| FEZ2 | -0.1581623 | 0.0002937 | 0.00085274 |
| SOCS6 | -0.1581252 | 0.00029468 | 0.00085545 |
| ASCC3 | -0.1580891 | 0.00029564 | 0.00085803 |
| XKRX | -0.1580883 | 0.00029566 | 0.00085803 |
| PCP2 | 0.1580655 | 0.00029626 | 0.00085965 |
| SUN2 | -0.1580641 | 0.0002963 | 0.00085965 |
| ZNF98 | -0.1580535 | 0.00029658 | 0.00086034 |
| NOVA1 | -0.1580328 | 0.00029713 | 0.00086182 |
| C19orf61 | 0.1580045 | 0.00029789 | 0.00086388 |
| FBXO25 | 0.1579761 | 0.00029864 | 0.00086595 |
| MPHOSPH9 | 0.1579549 | 0.00029921 | 0.00086747 |
| DNAJB11 | 0.1579309 | 0.00029985 | 0.00086921 |
| GUCY1B3 | -0.1579157 | 0.00030026 | 0.00087027 |
| CGB5 | -0.157897 | 0.00030077 | 0.00087161 |
| PREX1 | -0.1578896 | 0.00030096 | 0.00087206 |
| OR1E2 | 0.1578829 | 0.00030115 | 0.00087246 |
| FAM166A | 0.1578764 | 0.00030132 | 0.00087284 |
| GADL1 | -0.1578576 | 0.00030183 | 0.00087418 |
| STT3A | -0.157851 | 0.00030201 | 0.00087457 |
| OR52A4 | 0.1578348 | 0.00030244 | 0.00087571 |
| C1orf63 | 0.1577695 | 0.00030422 | 0.00088072 |
| FAM164C | 0.1577555 | 0.0003046 | 0.0008817 |
| C1orf213 | 0.1577389 | 0.00030505 | 0.00088287 |
| CD68 | -0.1577281 | 0.00030534 | 0.0008836 |
| SLC2A3 | -0.1577042 | 0.000306 | 0.00088537 |
| MBD3 | 0.1576525 | 0.00030741 | 0.00088934 |
| KIAA1239 | -0.1576322 | 0.00030797 | 0.00089082 |
| TMCO1 | 0.1576121 | 0.00030852 | 0.0008923 |
| ELMOD3 | 0.1575966 | 0.00030895 | 0.0008934 |
| HPS4 | -0.1575561 | 0.00031007 | 0.00089651 |
| PDLIM4 | -0.1575423 | 0.00031045 | 0.00089749 |
| C10orf82 | -0.1575325 | 0.00031073 | 0.00089815 |
| DIDO1 | -0.1575122 | 0.00031129 | 0.00089965 |
| CRX | 0.1575027 | 0.00031155 | 0.00090028 |
| RBP3 | 0.1574853 | 0.00031204 | 0.00090155 |
| HDAC1 | 0.1574652 | 0.0003126 | 0.00090304 |
| BTNL9 | 0.1574132 | 0.00031405 | 0.00090711 |
| SPA17 | 0.1573955 | 0.00031455 | 0.00090819 |
| GRAMD1A | -0.1573953 | 0.00031456 | 0.00090819 |
| C17orf66 | 0.157395 | 0.00031456 | 0.00090819 |
| PROC | -0.157377 | 0.00031507 | 0.00090952 |
| NME5 | -0.1573753 | 0.00031512 | 0.00090953 |
| YJEFN3 | 0.1573737 | 0.00031516 | 0.00090954 |
| HIST2H2BF | -0.1573349 | 0.00031625 | 0.00091256 |
| PFN1 | 0.1572997 | 0.00031725 | 0.00091529 |
| UFD1L | 0.1572851 | 0.00031766 | 0.00091636 |
| TKT | 0.1572824 | 0.00031774 | 0.00091636 |
| HIGD2B | 0.1572818 | 0.00031775 | 0.00091636 |
| G0S2 | -0.1572778 | 0.00031787 | 0.00091655 |
| FER1L4 | 0.1572652 | 0.00031823 | 0.00091735 |
| ZFP36L1 | -0.1572649 | 0.00031823 | 0.00091735 |
| DHX9 | 0.1572614 | 0.00031833 | 0.00091751 |
| BCL11A | 0.1572419 | 0.00031889 | 0.00091897 |
| KCNT1 | -0.1571768 | 0.00032074 | 0.00092419 |
| KCNS2 | -0.1571606 | 0.0003212 | 0.00092539 |
| LGR4 | -0.1571516 | 0.00032146 | 0.000926 |
| PRINS | 0.1571157 | 0.00032249 | 0.00092883 |
| PCDHA4 | -0.1571118 | 0.00032261 | 0.00092893 |
| GCET2 | 0.1571112 | 0.00032262 | 0.00092893 |
| IKBKE | 0.1570886 | 0.00032327 | 0.00093067 |
| TEAD1 | -0.1570626 | 0.00032402 | 0.0009327 |
| PTPRT | -0.157029 | 0.00032499 | 0.00093536 |
| SLC39A10 | -0.1569922 | 0.00032606 | 0.00093829 |
| ADAMTS6 | -0.1569873 | 0.0003262 | 0.00093857 |
| FGF17 | 0.1569771 | 0.0003265 | 0.00093929 |
| KIF7 | -0.1569597 | 0.000327 | 0.00094061 |
| ITFG3 | -0.1569198 | 0.00032817 | 0.00094382 |
| SERPINB5 | -0.1569162 | 0.00032827 | 0.00094399 |
| GPM6B | -0.1569094 | 0.00032847 | 0.00094443 |
| PREB | 0.1569008 | 0.00032872 | 0.00094501 |
| BST1 | -0.1568728 | 0.00032954 | 0.00094724 |
| FAM190A | -0.156832 | 0.00033074 | 0.00095055 |
| ANKK1 | -0.1567967 | 0.00033178 | 0.00095339 |
| PZP | -0.1567919 | 0.00033192 | 0.00095367 |
| ARID4B | -0.1567826 | 0.00033219 | 0.00095432 |
| NHS | -0.1567789 | 0.0003323 | 0.00095449 |
| RIC8B | 0.156741 | 0.00033342 | 0.00095758 |
| CDKAL1 | 0.1567374 | 0.00033353 | 0.00095775 |
| GRIK5 | -0.1567218 | 0.000334 | 0.00095895 |
| PFKFB1 | 0.1567161 | 0.00033416 | 0.0009593 |
| EIF4G2 | -0.1566852 | 0.00033508 | 0.00096179 |
| SMC3 | 0.1566793 | 0.00033526 | 0.00096216 |
| ACIN1 | 0.1566571 | 0.00033592 | 0.00096381 |
| IGFBP3 | -0.1566568 | 0.00033593 | 0.00096381 |
| NGEF | -0.1566469 | 0.00033622 | 0.00096452 |
| PHOSPHO1 | -0.1566337 | 0.00033662 | 0.00096551 |
| ADAM30 | 0.1565788 | 0.00033826 | 0.0009701 |
| TCF25 | -0.1565219 | 0.00033998 | 0.00097487 |
| COMMD6 | 0.156517 | 0.00034012 | 0.00097515 |
| PDP2 | -0.1565006 | 0.00034062 | 0.00097643 |
| KIAA1586 | 0.1564636 | 0.00034174 | 0.00097951 |
| AGAP8 | 0.1564206 | 0.00034304 | 0.00098311 |
| PATL2 | 0.1564098 | 0.00034338 | 0.00098392 |
| FAM115C | -0.1563869 | 0.00034407 | 0.00098577 |
| FAM86A | 0.1562279 | 0.00034896 | 0.00099963 |
| SH3BGR | -0.1562249 | 0.00034905 | 0.00099975 |
| PDDC1 | 0.1562184 | 0.00034925 | 0.00100019 |
| CORO6 | -0.1562146 | 0.00034937 | 0.00100038 |
| MS4A10 | 0.1562046 | 0.00034968 | 0.001001 |
| RAB11A | 0.1562044 | 0.00034968 | 0.001001 |
| ProSAPiP1 | -0.156198 | 0.00034988 | 0.00100143 |
| FAM18B2 | 0.1561879 | 0.0003502 | 0.00100218 |
| CHRNB1 | -0.1561639 | 0.00035094 | 0.00100416 |
| RPL13AP20 | 0.1561505 | 0.00035136 | 0.00100521 |
| MUSTN1 | -0.1561407 | 0.00035166 | 0.00100595 |
| EEF1G | 0.1561278 | 0.00035206 | 0.00100695 |
| DNAH9 | -0.1561113 | 0.00035258 | 0.00100828 |
| CCDC102B | -0.1560987 | 0.00035297 | 0.00100927 |
| MFHAS1 | -0.1560875 | 0.00035332 | 0.00101012 |
| TSPYL3 | -0.15608 | 0.00035356 | 0.0010106 |
| FAM98A | -0.1560789 | 0.00035359 | 0.0010106 |
| SRPK3 | -0.1560702 | 0.00035387 | 0.00101124 |
| IRF1 | 0.1560672 | 0.00035396 | 0.00101137 |
| LOC285359 | 0.1560552 | 0.00035433 | 0.00101229 |
| WDR78 | -0.15604 | 0.00035481 | 0.00101351 |
| DNAJC10 | -0.1560216 | 0.00035539 | 0.00101502 |
| CCDC144A | -0.1559871 | 0.00035648 | 0.00101798 |
| KIF5B | -0.1559809 | 0.00035667 | 0.0010184 |
| CDC14C | -0.1559403 | 0.00035795 | 0.00102191 |
| RESP18 | 0.1559379 | 0.00035803 | 0.00102198 |
| CNOT1 | -0.1559027 | 0.00035915 | 0.00102503 |
| GRIN3B | -0.155901 | 0.0003592 | 0.00102504 |
| TFR2 | 0.155842 | 0.00036108 | 0.00103025 |
| TRMT5 | 0.1558129 | 0.00036201 | 0.00103276 |
| LOXL3 | -0.1557989 | 0.00036246 | 0.0010339 |
| TRAM1 | -0.1557912 | 0.00036271 | 0.00103445 |
| DCAF8 | -0.1557731 | 0.00036328 | 0.00103596 |
| FKSG29 | -0.1557636 | 0.00036359 | 0.00103668 |
| GPR97 | -0.1557377 | 0.00036442 | 0.00103891 |
| C1orf9 | -0.1557061 | 0.00036544 | 0.00104166 |
| SCARNA16 | 0.1556783 | 0.00036634 | 0.00104408 |
| PRR21 | 0.1556634 | 0.00036682 | 0.0010453 |
| PIGL | 0.1556403 | 0.00036757 | 0.00104729 |
| EFCAB7 | 0.1556256 | 0.00036805 | 0.0010485 |
| GPLD1 | -0.1556057 | 0.0003687 | 0.0010502 |
| DOCK3 | -0.1556025 | 0.0003688 | 0.00105035 |
| DNM1 | -0.1555999 | 0.00036888 | 0.00105044 |
| ZBED4 | -0.1555861 | 0.00036933 | 0.00105152 |
| KBTBD7 | -0.1555838 | 0.00036941 | 0.00105152 |
| PRDM4 | -0.1555835 | 0.00036942 | 0.00105152 |
| LOC285593 | -0.1555762 | 0.00036966 | 0.00105205 |
| BPESC1 | 0.1555541 | 0.00037038 | 0.00105395 |
| GRB10 | -0.1555143 | 0.00037168 | 0.00105751 |
| C10orf99 | -0.1555014 | 0.0003721 | 0.00105856 |
| ZNF436 | -0.1554988 | 0.00037219 | 0.00105866 |
| MAN2B1 | -0.1554809 | 0.00037278 | 0.00106018 |
| COPB1 | -0.1554767 | 0.00037291 | 0.00106038 |
| RABEP1 | -0.1554756 | 0.00037295 | 0.00106038 |
| ARPP19 | 0.1554451 | 0.00037396 | 0.00106308 |
| CCDC54 | -0.1554326 | 0.00037437 | 0.0010641 |
| RHOB | -0.1553951 | 0.00037561 | 0.00106748 |
| SNRNP27 | 0.1553912 | 0.00037574 | 0.0010677 |
| OR1D2 | 0.1553674 | 0.00037653 | 0.00106978 |
| NMNAT2 | -0.1553359 | 0.00037757 | 0.00107252 |
| SCYL1 | -0.1553352 | 0.0003776 | 0.00107252 |
| ZNF138 | 0.1553196 | 0.00037811 | 0.00107385 |
| SPRR2D | -0.1553144 | 0.00037829 | 0.0010742 |
| TRIM54 | -0.1553106 | 0.00037842 | 0.0010744 |
| ZNF26 | 0.1553083 | 0.00037849 | 0.00107446 |
| SFRS2IP | -0.1552782 | 0.0003795 | 0.00107717 |
| ZNF75A | 0.155274 | 0.00037964 | 0.00107741 |
| DHDDS | -0.1552715 | 0.00037972 | 0.00107746 |
| TREM1 | -0.1552703 | 0.00037976 | 0.00107746 |
| PNCK | 0.1552638 | 0.00037998 | 0.00107793 |
| C10orf47 | 0.1552522 | 0.00038037 | 0.00107888 |
| LOC116437 | -0.1552418 | 0.00038072 | 0.0010797 |
| FHL3 | -0.1552403 | 0.00038077 | 0.0010797 |
| ELOVL4 | -0.1552111 | 0.00038175 | 0.00108233 |
| SP2 | -0.1551702 | 0.00038312 | 0.00108606 |
| DRG2 | 0.1551688 | 0.00038317 | 0.00108606 |
| SLC22A6 | 0.1551633 | 0.00038336 | 0.00108644 |
| PI3 | -0.1551588 | 0.00038351 | 0.00108672 |
| TBC1D25 | -0.1551551 | 0.00038364 | 0.00108692 |
| LOC285830 | 0.1551515 | 0.00038376 | 0.00108711 |
| COQ9 | 0.1551497 | 0.00038382 | 0.00108713 |
| ATG9A | -0.1551469 | 0.00038391 | 0.00108725 |
| ZNF205 | 0.1551256 | 0.00038463 | 0.00108913 |
| DPP6 | -0.1551231 | 0.00038472 | 0.00108922 |
| EPC1 | -0.1551104 | 0.00038515 | 0.00109028 |
| MED22 | 0.1551075 | 0.00038525 | 0.00109041 |
| LOC641298 | -0.1551015 | 0.00038545 | 0.00109084 |
| G3BP1 | 0.1550931 | 0.00038573 | 0.00109148 |
| ADAMTSL4 | -0.1550642 | 0.00038672 | 0.00109411 |
| ZNF561 | -0.1550181 | 0.00038829 | 0.0010984 |
| ITIH5L | -0.1550055 | 0.00038872 | 0.00109947 |
| SIRT3 | 0.1549661 | 0.00039007 | 0.00110313 |
| PRDX6 | 0.1549419 | 0.0003909 | 0.00110532 |
| DLG2 | -0.1549316 | 0.00039125 | 0.00110617 |
| CFI | -0.154908 | 0.00039206 | 0.00110831 |
| C14orf166B | 0.1548992 | 0.00039237 | 0.00110901 |
| RASIP1 | 0.1548851 | 0.00039285 | 0.00111023 |
| PRKAA2 | -0.1548824 | 0.00039295 | 0.00111034 |
| THEM5 | -0.1548757 | 0.00039318 | 0.00111084 |
| BIK | 0.1548669 | 0.00039348 | 0.00111154 |
| FAM133A | -0.1548531 | 0.00039396 | 0.00111273 |
| 1-Mar | -0.1548351 | 0.00039459 | 0.00111435 |
| UVRAG | -0.154818 | 0.00039518 | 0.00111587 |
| SLC39A9 | -0.1548054 | 0.00039562 | 0.00111694 |
| DHX36 | -0.1547923 | 0.00039607 | 0.00111807 |
| HIST1H2BI | 0.1547875 | 0.00039624 | 0.00111839 |
| CILP2 | -0.1547822 | 0.00039642 | 0.00111875 |
| PRSS16 | 0.1547763 | 0.00039663 | 0.00111917 |
| WTIP | -0.1547705 | 0.00039683 | 0.00111958 |
| LSM10 | 0.1547654 | 0.00039701 | 0.00111993 |
| SLC36A2 | -0.1547521 | 0.00039747 | 0.00112108 |
| AMHR2 | -0.1547147 | 0.00039878 | 0.00112461 |
| RBM16 | -0.154712 | 0.00039887 | 0.00112472 |
| FAM176A | -0.1546821 | 0.00039992 | 0.00112752 |
| SQRDL | 0.1546763 | 0.00040012 | 0.00112793 |
| GNRHR2 | 0.1546586 | 0.00040075 | 0.00112953 |
| COL28A1 | -0.1546535 | 0.00040093 | 0.00112988 |
| LIPA | -0.1546047 | 0.00040265 | 0.00113457 |
| CLEC2B | 0.1545957 | 0.00040297 | 0.00113531 |
| MYL6 | 0.1545801 | 0.00040352 | 0.00113671 |
| ERCC6 | -0.1545041 | 0.00040622 | 0.00114415 |
| WSB2 | -0.1544972 | 0.00040646 | 0.00114468 |
| ZSCAN20 | -0.1544744 | 0.00040728 | 0.00114682 |
| ARAP1 | -0.1544621 | 0.00040772 | 0.00114789 |
| SLC15A4 | -0.1544571 | 0.00040789 | 0.00114823 |
| INSC | -0.1544436 | 0.00040838 | 0.00114943 |
| LTV1 | 0.154382 | 0.00041059 | 0.00115549 |
| GPR78 | -0.1543684 | 0.00041108 | 0.00115671 |
| OR6S1 | 0.1543634 | 0.00041126 | 0.00115705 |
| RGS12 | -0.1543538 | 0.0004116 | 0.00115786 |
| CLCN6 | -0.1543503 | 0.00041173 | 0.00115806 |
| AES | 0.1542997 | 0.00041356 | 0.00116304 |
| MAST1 | 0.1542927 | 0.00041381 | 0.00116359 |
| ZNF787 | 0.1542741 | 0.00041449 | 0.00116532 |
| C13orf15 | -0.1542402 | 0.00041572 | 0.00116862 |
| FOLR4 | 0.1542175 | 0.00041655 | 0.00117079 |
| TPP2 | -0.1542062 | 0.00041696 | 0.00117178 |
| KDM1A | 0.1541048 | 0.00042067 | 0.00118206 |
| TMEM8A | -0.1540895 | 0.00042124 | 0.00118348 |
| SLC12A8 | -0.1540576 | 0.00042241 | 0.00118662 |
| NAT2 | 0.1540064 | 0.00042431 | 0.00119178 |
| PRDM15 | 0.1539927 | 0.00042482 | 0.00119305 |
| C9orf3 | 0.1539878 | 0.000425 | 0.0011933 |
| TAOK2 | -0.153987 | 0.00042503 | 0.0011933 |
| KIAA1967 | 0.1539706 | 0.00042564 | 0.00119485 |
| RABL2A | 0.1539648 | 0.00042586 | 0.00119529 |
| JRKL | -0.1539369 | 0.00042689 | 0.00119804 |
| DOHH | 0.1538989 | 0.00042832 | 0.00120186 |
| RDH14 | 0.1538972 | 0.00042838 | 0.00120187 |
| C3 | -0.1538953 | 0.00042845 | 0.0012019 |
| CTRC | 0.1538449 | 0.00043034 | 0.00120703 |
| GSTA3 | -0.153841 | 0.00043049 | 0.00120728 |
| WBSCR28 | 0.1538302 | 0.00043089 | 0.00120816 |
| ACOT11 | -0.1538295 | 0.00043092 | 0.00120816 |
| MDC1 | 0.1538213 | 0.00043123 | 0.00120885 |
| MLXIPL | -0.1538053 | 0.00043183 | 0.00121038 |
| FMO1 | -0.1537969 | 0.00043215 | 0.00121109 |
| TTC17 | -0.1537714 | 0.00043311 | 0.00121359 |
| MYRIP | -0.1537701 | 0.00043316 | 0.00121359 |
| LSM11 | 0.1537673 | 0.00043326 | 0.00121372 |
| HCCS | 0.1537568 | 0.00043366 | 0.00121466 |
| TUBB1 | 0.1537437 | 0.00043416 | 0.00121589 |
| LCE1D | -0.1537407 | 0.00043427 | 0.001216 |
| C16orf57 | -0.1537394 | 0.00043432 | 0.001216 |
| SOX17 | -0.1537283 | 0.00043474 | 0.00121702 |
| DNAJC18 | -0.1537238 | 0.00043491 | 0.00121732 |
| C11orf90 | -0.1537013 | 0.00043577 | 0.00121955 |
| LOC100128675 | 0.153685 | 0.00043639 | 0.00122112 |
| CEP57 | 0.1536745 | 0.00043679 | 0.00122201 |
| SNHG9 | 0.1536734 | 0.00043683 | 0.00122201 |
| TTF2 | 0.1536681 | 0.00043703 | 0.00122241 |
| ADRA2C | -0.1536649 | 0.00043716 | 0.00122258 |
| OS9 | -0.1536564 | 0.00043748 | 0.00122332 |
| LASP1 | -0.1536291 | 0.00043852 | 0.00122607 |
| LGR6 | -0.1535686 | 0.00044084 | 0.00123238 |
| KPNA1 | -0.1535623 | 0.00044109 | 0.00123289 |
| ME3 | -0.1535431 | 0.00044182 | 0.00123478 |
| PNKD | 0.1535306 | 0.0004423 | 0.00123595 |
| PIGY | 0.1535044 | 0.00044332 | 0.00123861 |
| TM2D2 | 0.1534678 | 0.00044473 | 0.00124239 |
| NIPAL4 | -0.1534658 | 0.00044481 | 0.00124244 |
| RAC1 | 0.1534525 | 0.00044533 | 0.00124371 |
| CIRH1A | 0.153432 | 0.00044612 | 0.00124576 |
| CTNNBL1 | 0.1534026 | 0.00044727 | 0.00124878 |
| ARL16 | 0.1533977 | 0.00044746 | 0.00124914 |
| DHRS9 | -0.1533896 | 0.00044777 | 0.00124985 |
| C20orf134 | 0.1533703 | 0.00044853 | 0.00125178 |
| ZNF697 | -0.1533683 | 0.00044861 | 0.00125183 |
| GALR3 | 0.153366 | 0.0004487 | 0.0012519 |
| KRTAP10-11 | 0.1532926 | 0.00045157 | 0.00125975 |
| ZDBF2 | -0.1532852 | 0.00045186 | 0.00126039 |
| ANGPTL3 | 0.1532611 | 0.00045281 | 0.00126286 |
| PIGC | 0.1532527 | 0.00045314 | 0.00126361 |
| C3orf55 | -0.153219 | 0.00045447 | 0.00126715 |
| HDHD2 | 0.153214 | 0.00045467 | 0.00126752 |
| RXFP1 | -0.1531974 | 0.00045533 | 0.00126917 |
| SLC9A7 | -0.1531762 | 0.00045617 | 0.00127134 |
| CRIP1 | 0.1531704 | 0.0004564 | 0.00127181 |
| MIOX | 0.1531604 | 0.00045679 | 0.00127274 |
| NFU1 | 0.1531169 | 0.00045853 | 0.00127739 |
| CNIH | 0.1531035 | 0.00045906 | 0.0012787 |
| LRRC29 | -0.1530997 | 0.00045921 | 0.00127895 |
| C20orf54 | -0.1530872 | 0.00045971 | 0.00128016 |
| WDR70 | 0.1530724 | 0.0004603 | 0.00128163 |
| KRAS | -0.1530622 | 0.00046071 | 0.0012826 |
| FHL2 | -0.1530291 | 0.00046204 | 0.00128611 |
| GSDMD | 0.1530233 | 0.00046227 | 0.00128655 |
| C4A | -0.153022 | 0.00046233 | 0.00128655 |
| GJA5 | -0.153007 | 0.00046293 | 0.00128802 |
| LCE3A | -0.1530046 | 0.00046303 | 0.00128802 |
| CCNG1 | 0.1530041 | 0.00046305 | 0.00128802 |
| KCNIP1 | -0.1529964 | 0.00046335 | 0.0012887 |
| KCNA4 | -0.1529827 | 0.00046391 | 0.00129006 |
| QPCT | -0.1529779 | 0.0004641 | 0.00129042 |
| CSDE1 | -0.1529594 | 0.00046485 | 0.00129232 |
| HSD17B8 | 0.1529524 | 0.00046513 | 0.00129293 |
| LRRK2 | -0.1528688 | 0.00046852 | 0.00130217 |
| RAB13 | 0.1528417 | 0.00046963 | 0.00130492 |
| EPM2A | -0.1528413 | 0.00046964 | 0.00130492 |
| KIRREL2 | 0.1528305 | 0.00047008 | 0.00130597 |
| LOC283856 | -0.1528087 | 0.00047097 | 0.00130826 |
| FAAH2 | 0.1528031 | 0.0004712 | 0.00130872 |
| ZNF568 | -0.152799 | 0.00047137 | 0.001309 |
| GPR183 | -0.1527541 | 0.00047321 | 0.00131393 |
| FKBP4 | 0.1527402 | 0.00047378 | 0.00131534 |
| CRTAC1 | -0.152725 | 0.0004744 | 0.00131688 |
| BAG3 | -0.1526995 | 0.00047546 | 0.00131963 |
| GDF9 | 0.1526852 | 0.00047605 | 0.001321 |
| ZNF85 | -0.1526843 | 0.00047608 | 0.001321 |
| NLGN1 | -0.1526287 | 0.00047838 | 0.0013272 |
| SDF4 | -0.152623 | 0.00047862 | 0.00132768 |
| PDE9A | -0.1526133 | 0.00047902 | 0.00132849 |
| ZNF549 | -0.1526128 | 0.00047904 | 0.00132849 |
| NUP188 | 0.1526096 | 0.00047918 | 0.00132867 |
| SERINC2 | -0.1526054 | 0.00047935 | 0.00132897 |
| MRPL36 | 0.1525903 | 0.00047998 | 0.00133053 |
| ACADL | -0.1525818 | 0.00048033 | 0.00133133 |
| XRCC5 | 0.1525629 | 0.00048112 | 0.00133333 |
| FOXC2 | -0.1525331 | 0.00048236 | 0.00133659 |
| SH3GL2 | 0.1525186 | 0.00048297 | 0.00133808 |
| ECHDC2 | 0.1525165 | 0.00048306 | 0.00133815 |
| KCTD4 | -0.1524674 | 0.00048512 | 0.00134367 |
| FAS | 0.1524181 | 0.00048719 | 0.00134923 |
| C1orf83 | 0.1523801 | 0.0004888 | 0.00135349 |
| GRPR | -0.1523486 | 0.00049013 | 0.00135699 |
| SFRP5 | -0.1523415 | 0.00049043 | 0.00135765 |
| ARID4A | -0.1523385 | 0.00049056 | 0.00135782 |
| LOC100129637 | 0.1523084 | 0.00049184 | 0.00136117 |
| CDKL4 | 0.1522328 | 0.00049507 | 0.00136991 |
| THAP11 | 0.1521615 | 0.00049813 | 0.00137819 |
| NPY1R | -0.1521473 | 0.00049874 | 0.0013796 |
| B4GALNT1 | -0.1521465 | 0.00049877 | 0.0013796 |
| NDUFA10 | 0.1521198 | 0.00049993 | 0.00138261 |
| REEP3 | -0.1520945 | 0.00050102 | 0.00138544 |
| NOG | -0.1520613 | 0.00050246 | 0.00138923 |
| NCRNA00160 | -0.1520529 | 0.00050283 | 0.00139005 |
| FAM46B | -0.1520415 | 0.00050332 | 0.00139122 |
| AP3S2 | 0.1520347 | 0.00050362 | 0.00139185 |
| DHX33 | -0.1520219 | 0.00050417 | 0.0013932 |
| PPP1R9A | -0.151989 | 0.00050561 | 0.00139697 |
| RIOK2 | 0.1519868 | 0.0005057 | 0.00139705 |
| BOK | -0.1519702 | 0.00050643 | 0.00139878 |
| TMEM74 | 0.1519693 | 0.00050647 | 0.00139878 |
| KRI1 | 0.1519528 | 0.00050719 | 0.00140058 |
| SLK | -0.1519418 | 0.00050767 | 0.00140172 |
| TMED10 | -0.1519215 | 0.00050856 | 0.00140398 |
| PSG7 | -0.1519003 | 0.0005095 | 0.00140637 |
| PPM1F | -0.151896 | 0.00050969 | 0.0014067 |
| GNG8 | 0.151889 | 0.00051 | 0.00140736 |
| SCRN2 | 0.1518647 | 0.00051106 | 0.00141011 |
| LPAR2 | 0.1518237 | 0.00051288 | 0.00141474 |
| KIF13B | -0.1518235 | 0.00051288 | 0.00141474 |
| FBXL15 | 0.151822 | 0.00051295 | 0.00141474 |
| C11orf41 | -0.1517907 | 0.00051434 | 0.00141837 |
| GZMH | 0.1517614 | 0.00051564 | 0.00142177 |
| CCL5 | 0.1517575 | 0.00051581 | 0.00142205 |
| CSDAP1 | -0.1517208 | 0.00051745 | 0.00142636 |
| C5orf36 | -0.1516947 | 0.00051862 | 0.00142938 |
| MPV17L2 | 0.1516839 | 0.0005191 | 0.00143052 |
| FOXD3 | 0.1516795 | 0.00051929 | 0.00143086 |
| DNAJB5 | -0.1516712 | 0.00051967 | 0.00143169 |
| CDKN2D | 0.1516586 | 0.00052023 | 0.00143305 |
| ZNRF4 | 0.1516548 | 0.0005204 | 0.00143333 |
| EPGN | -0.151602 | 0.00052277 | 0.00143966 |
| ZNF614 | -0.1515822 | 0.00052367 | 0.00144192 |
| RAB38 | -0.1515756 | 0.00052397 | 0.0014424 |
| ZDHHC1 | -0.1515752 | 0.00052398 | 0.0014424 |
| CCL25 | 0.1515427 | 0.00052545 | 0.00144625 |
| DHX34 | 0.1514998 | 0.0005274 | 0.0014514 |
| NXPH3 | -0.1514656 | 0.00052895 | 0.00145548 |
| GSPT2 | -0.1514391 | 0.00053016 | 0.0014586 |
| RNPC3 | 0.1514095 | 0.00053151 | 0.00146206 |
| C6orf132 | -0.1514084 | 0.00053156 | 0.00146206 |
| KCTD2 | -0.1513932 | 0.00053226 | 0.00146378 |
| THAP5 | 0.1513916 | 0.00053233 | 0.00146379 |
| ELF5 | -0.1513881 | 0.00053249 | 0.00146403 |
| C6orf170 | -0.1513584 | 0.00053386 | 0.00146758 |
| C6orf72 | -0.1513273 | 0.00053529 | 0.00147131 |
| C17orf86 | 0.1512806 | 0.00053744 | 0.00147702 |
| DLK1 | -0.151251 | 0.00053881 | 0.00148059 |
| ZNF652 | -0.1512213 | 0.00054019 | 0.00148417 |
| ATXN7L3 | -0.151188 | 0.00054173 | 0.00148821 |
| KIAA0831 | -0.1511668 | 0.00054272 | 0.0014906 |
| CLDN17 | -0.1511662 | 0.00054275 | 0.0014906 |
| MYBPC1 | -0.1511574 | 0.00054316 | 0.00149152 |
| CLDN7 | 0.151145 | 0.00054374 | 0.00149291 |
| FLJ36031 | -0.1511336 | 0.00054427 | 0.00149417 |
| APOBEC3G | 0.1511303 | 0.00054443 | 0.00149436 |
| ALDH1A3 | -0.1511275 | 0.00054456 | 0.00149436 |
| AGAP1 | -0.1511274 | 0.00054456 | 0.00149436 |
| STAM | -0.1511238 | 0.00054473 | 0.00149461 |
| GLRA4 | -0.1510876 | 0.00054643 | 0.00149907 |
| MYOM2 | -0.1510794 | 0.00054681 | 0.00149992 |
| CUTA | 0.1510586 | 0.00054779 | 0.00150239 |
| ADORA1 | -0.151057 | 0.00054787 | 0.0015024 |
| DMGDH | -0.1510315 | 0.00054906 | 0.00150548 |
| TMEM117 | -0.151018 | 0.0005497 | 0.00150702 |
| PRR23A | 0.1510136 | 0.00054991 | 0.00150739 |
| ZBED1 | -0.15101 | 0.00055008 | 0.00150766 |
| SREBF1 | 0.1509962 | 0.00055073 | 0.00150924 |
| TAS2R39 | 0.1509584 | 0.00055252 | 0.00151394 |
| SLC27A6 | -0.1509412 | 0.00055334 | 0.00151592 |
| HOPX | -0.1509399 | 0.0005534 | 0.00151592 |
| GPR85 | -0.1508857 | 0.00055598 | 0.00152279 |
| ST8SIA1 | -0.1508767 | 0.00055641 | 0.00152375 |
| TRIM9 | -0.1508654 | 0.00055695 | 0.00152502 |
| C19orf76 | 0.1508432 | 0.00055801 | 0.00152773 |
| ENTPD5 | -0.1508254 | 0.00055886 | 0.00152986 |
| ITSN2 | -0.1508155 | 0.00055933 | 0.00153094 |
| NMRAL1 | 0.1508076 | 0.00055972 | 0.00153178 |
| CSF1 | -0.1508044 | 0.00055987 | 0.00153198 |
| BAT2L2 | -0.1507838 | 0.00056086 | 0.00153432 |
| CCT8L2 | 0.1507835 | 0.00056087 | 0.00153432 |
| AGBL5 | 0.1507742 | 0.00056132 | 0.00153533 |
| FAM193A | -0.1507577 | 0.00056211 | 0.00153729 |
| SERPINA3 | -0.1507507 | 0.00056245 | 0.00153801 |
| CHRM5 | -0.1507053 | 0.00056464 | 0.00154379 |
| RAB3GAP2 | -0.150687 | 0.00056553 | 0.00154601 |
| TMEM98 | -0.1506838 | 0.00056569 | 0.00154623 |
| SLC25A16 | 0.1506813 | 0.00056581 | 0.00154635 |
| DMXL1 | -0.150676 | 0.00056607 | 0.00154684 |
| DHX8 | -0.1506227 | 0.00056865 | 0.00155371 |
| SLC47A2 | -0.1505887 | 0.00057031 | 0.00155802 |
| FAM92A1 | 0.150573 | 0.00057108 | 0.00155991 |
| SERTAD4 | -0.1505654 | 0.00057145 | 0.00156071 |
| MYSM1 | -0.1505634 | 0.00057155 | 0.00156077 |
| C11orf58 | 0.1505566 | 0.00057188 | 0.00156147 |
| LRRC30 | -0.1505347 | 0.00057296 | 0.00156419 |
| LOC121952 | -0.1505059 | 0.00057437 | 0.00156784 |
| OR2Z1 | 0.1504739 | 0.00057595 | 0.00157192 |
| CASC2 | 0.1504389 | 0.00057767 | 0.00157642 |
| DEFB131 | 0.1504344 | 0.00057789 | 0.00157681 |
| ZNF292 | -0.1504323 | 0.000578 | 0.00157688 |
| CCPG1 | -0.1503927 | 0.00057996 | 0.00158202 |
| MON2 | -0.1503879 | 0.0005802 | 0.00158246 |
| AIM2 | 0.1503781 | 0.00058069 | 0.00158344 |
| SLC30A6 | -0.1503775 | 0.00058072 | 0.00158344 |
| TMEM39A | -0.1503711 | 0.00058103 | 0.00158409 |
| ZNF385A | -0.1503534 | 0.00058191 | 0.00158622 |
| FSCN2 | -0.1503522 | 0.00058197 | 0.00158622 |
| DAPK3 | -0.1503356 | 0.0005828 | 0.00158826 |
| CCDC64B | -0.150317 | 0.00058373 | 0.00159058 |
| KCNK15 | -0.150305 | 0.00058433 | 0.001592 |
| KLHL35 | 0.1502934 | 0.0005849 | 0.00159335 |
| QSOX2 | 0.1502878 | 0.00058519 | 0.00159371 |
| ARHGEF10 | -0.1502876 | 0.00058519 | 0.00159371 |
| DHRS7C | -0.1502509 | 0.00058703 | 0.0015985 |
| EFHA2 | -0.1502414 | 0.00058751 | 0.00159959 |
| OBSCN | -0.150228 | 0.00058818 | 0.0016012 |
| MRPL49 | 0.1502141 | 0.00058888 | 0.00160289 |
| WASH5P | 0.1501804 | 0.00059058 | 0.0016073 |
| ALOX12B | -0.1501759 | 0.0005908 | 0.00160769 |
| DDB1 | -0.150162 | 0.00059151 | 0.00160939 |
| SLC35F3 | -0.1501173 | 0.00059377 | 0.00161532 |
| PIK3CD | -0.1500678 | 0.00059628 | 0.00162195 |
| NOX5 | -0.1500237 | 0.00059853 | 0.00162784 |
| BCL7B | 0.1500208 | 0.00059868 | 0.00162802 |
| SLC25A21 | 0.1499977 | 0.00059986 | 0.00163101 |
| MTCP1 | 0.1499803 | 0.00060075 | 0.00163322 |
| ORAI2 | -0.1499762 | 0.00060097 | 0.00163358 |
| FGF8 | 0.1499627 | 0.00060166 | 0.00163514 |
| C1orf192 | -0.1499618 | 0.0006017 | 0.00163514 |
| ZBTB11 | -0.1499579 | 0.0006019 | 0.00163546 |
| ANO9 | 0.1499435 | 0.00060264 | 0.00163709 |
| C1orf27 | 0.1499431 | 0.00060266 | 0.00163709 |
| NEUROG1 | 0.1498507 | 0.00060743 | 0.00164981 |
| RNASEL | -0.1498397 | 0.000608 | 0.00165114 |
| C4orf10 | -0.1498371 | 0.00060814 | 0.00165129 |
| EGR1 | -0.149797 | 0.00061022 | 0.00165672 |
| AKR1C2 | -0.1497806 | 0.00061107 | 0.00165881 |
| ENTPD3 | -0.1497669 | 0.00061179 | 0.00166052 |
| CDH1 | -0.149751 | 0.00061261 | 0.00166254 |
| MAN1C1 | -0.1497151 | 0.00061449 | 0.00166728 |
| TGIF2LY | -0.1497145 | 0.00061452 | 0.00166728 |
| METRNL | -0.1497075 | 0.00061489 | 0.00166805 |
| C9orf103 | 0.1496834 | 0.00061615 | 0.00167125 |
| C5orf51 | -0.1496254 | 0.0006192 | 0.00167929 |
| SCN2A | -0.149576 | 0.00062181 | 0.00168615 |
| ANKRD43 | 0.1495622 | 0.00062254 | 0.00168789 |
| ITGA6 | -0.1495338 | 0.00062405 | 0.00169175 |
| TOX4 | -0.1495237 | 0.00062458 | 0.00169298 |
| C9orf110 | -0.1495209 | 0.00062473 | 0.00169316 |
| EMR3 | -0.1494729 | 0.00062729 | 0.00169985 |
| ZNF92 | 0.1494637 | 0.00062778 | 0.00170096 |
| EPHA6 | 0.14945 | 0.00062851 | 0.00170272 |
| GAA | -0.1494465 | 0.0006287 | 0.001703 |
| PPP1R12B | -0.1494215 | 0.00063004 | 0.00170638 |
| TFIP11 | -0.1493688 | 0.00063286 | 0.00171381 |
| RFX1 | -0.1493647 | 0.00063309 | 0.00171418 |
| MAPKAPK3 | -0.1493502 | 0.00063386 | 0.00171606 |
| C15orf41 | 0.1493434 | 0.00063423 | 0.00171683 |
| DRP2 | -0.1493402 | 0.00063441 | 0.00171707 |
| BCL6B | -0.1493296 | 0.00063498 | 0.00171838 |
| PRDM10 | -0.1493114 | 0.00063596 | 0.00172081 |
| CD163 | -0.149306 | 0.00063625 | 0.00172124 |
| SDHAP3 | 0.1493053 | 0.00063629 | 0.00172124 |
| FLJ37201 | 0.1492967 | 0.00063675 | 0.00172226 |
| ZNF681 | -0.149286 | 0.00063733 | 0.0017236 |
| CALM1 | -0.1492779 | 0.00063777 | 0.00172455 |
| XAB2 | 0.1492741 | 0.00063798 | 0.00172488 |
| PLAU | -0.1492598 | 0.00063875 | 0.00172674 |
| C1orf50 | 0.1492542 | 0.00063905 | 0.00172733 |
| PCGF1 | 0.1492315 | 0.00064029 | 0.00173044 |
| PLXNA1 | -0.14921 | 0.00064146 | 0.00173336 |
| C22orf45 | 0.1490857 | 0.00064826 | 0.00175152 |
| APOF | 0.1490679 | 0.00064924 | 0.00175392 |
| FAM84B | -0.1490166 | 0.00065207 | 0.00176134 |
| TMEM45B | -0.1490022 | 0.00065287 | 0.00176313 |
| FAM171A2 | -0.1490005 | 0.00065296 | 0.00176313 |
| PAX3 | -0.1489999 | 0.000653 | 0.00176313 |
| FIBP | 0.1489825 | 0.00065396 | 0.00176549 |
| SSBP4 | 0.1489751 | 0.00065437 | 0.00176616 |
| GALK2 | 0.1489749 | 0.00065438 | 0.00176616 |
| RBM4 | 0.1489628 | 0.00065505 | 0.00176773 |
| CXCL11 | 0.1489593 | 0.00065525 | 0.00176802 |
| NFE2L3 | 0.1489558 | 0.00065544 | 0.00176831 |
| F8A1 | 0.1489458 | 0.000656 | 0.00176957 |
| YARS | 0.1489153 | 0.0006577 | 0.00177392 |
| RIMS4 | -0.1489096 | 0.00065801 | 0.00177453 |
| ETV1 | -0.1488964 | 0.00065875 | 0.00177628 |
| OR7G3 | 0.1488852 | 0.00065938 | 0.00177774 |
| CA12 | -0.1488196 | 0.00066305 | 0.00178736 |
| RPL31P11 | 0.1488183 | 0.00066312 | 0.00178736 |
| SIGLEC9 | -0.1487737 | 0.00066563 | 0.00179388 |
| LOC729020 | -0.1487691 | 0.0006659 | 0.00179435 |
| EBPL | 0.1487389 | 0.0006676 | 0.0017987 |
| C1orf66 | 0.1487265 | 0.0006683 | 0.00180035 |
| R3HDM2 | -0.1487016 | 0.00066971 | 0.00180368 |
| CRMP1 | -0.1487015 | 0.00066971 | 0.00180368 |
| MAP3K9 | -0.1486957 | 0.00067004 | 0.00180409 |
| TMEM132E | -0.1486957 | 0.00067005 | 0.00180409 |
| FANK1 | -0.1486919 | 0.00067026 | 0.00180442 |
| PTPN4 | -0.1486614 | 0.00067199 | 0.00180885 |
| TNFRSF12A | -0.1486311 | 0.00067372 | 0.00181324 |
| ZNF512B | -0.1486137 | 0.00067471 | 0.00181567 |
| SH2D4A | 0.1485917 | 0.00067596 | 0.00181881 |
| GSTO1 | 0.1485883 | 0.00067616 | 0.00181909 |
| PTPRZ1 | -0.1485684 | 0.0006773 | 0.00182192 |
| MED12 | -0.1485648 | 0.00067751 | 0.00182204 |
| HMG20A | 0.1485645 | 0.00067753 | 0.00182204 |
| SOX14 | 0.1485442 | 0.00067869 | 0.00182492 |
| ELL2 | -0.1485305 | 0.00067947 | 0.00182679 |
| YAF2 | 0.1485184 | 0.00068017 | 0.00182824 |
| PID1 | -0.1485166 | 0.00068027 | 0.00182824 |
| TMEM81 | 0.1485163 | 0.00068029 | 0.00182824 |
| MAP3K14 | 0.1484802 | 0.00068237 | 0.00183359 |
| MESDC1 | 0.148447 | 0.00068429 | 0.00183851 |
| C8orf45 | 0.1484138 | 0.00068621 | 0.00184335 |
| MGC14436 | 0.1484127 | 0.00068627 | 0.00184335 |
| G6PC | 0.1484007 | 0.00068697 | 0.00184498 |
| NRBP1 | -0.1483701 | 0.00068875 | 0.00184928 |
| LY6H | -0.14837 | 0.00068876 | 0.00184928 |
| SLC25A26 | 0.1483581 | 0.00068944 | 0.00185072 |
| MEIS1 | 0.1483568 | 0.00068952 | 0.00185072 |
| RICH2 | -0.148356 | 0.00068957 | 0.00185072 |
| LYRM2 | 0.1483288 | 0.00069115 | 0.00185473 |
| QARS | 0.1483251 | 0.00069137 | 0.00185506 |
| ZNF826 | -0.1482812 | 0.00069394 | 0.00186171 |
| NUDT18 | 0.1482667 | 0.00069479 | 0.00186373 |
| SLC22A15 | -0.1482561 | 0.00069541 | 0.00186516 |
| ABCE1 | 0.1482413 | 0.00069628 | 0.00186724 |
| TMEM189-UBE2V1 | -0.1482342 | 0.0006967 | 0.00186811 |
| CST2 | -0.1482229 | 0.00069736 | 0.00186965 |
| PRSS22 | -0.1482084 | 0.00069822 | 0.00187168 |
| CNFN | -0.1482035 | 0.0006985 | 0.0018722 |
| RPL29P2 | 0.1481869 | 0.00069948 | 0.00187459 |
| IQCB1 | 0.1481745 | 0.00070021 | 0.00187629 |
| PHLDB3 | -0.1481646 | 0.0007008 | 0.00187762 |
| DNAJC5 | -0.1481574 | 0.00070122 | 0.0018785 |
| SERF1A | 0.1481449 | 0.00070197 | 0.00188024 |
| TTC37 | -0.1481365 | 0.00070246 | 0.00188111 |
| UBD | 0.1481362 | 0.00070248 | 0.00188111 |
| DTD1 | 0.1481318 | 0.00070274 | 0.00188132 |
| CD3EAP | 0.1481316 | 0.00070275 | 0.00188132 |
| GDI2 | 0.1481302 | 0.00070283 | 0.00188132 |
| LOC283332 | 0.1481114 | 0.00070395 | 0.00188405 |
| GDPD5 | -0.1481002 | 0.00070462 | 0.00188559 |
| NUP210 | 0.1480942 | 0.00070497 | 0.00188629 |
| GNAT1 | 0.1480584 | 0.00070711 | 0.00189175 |
| TEX15 | 0.1480459 | 0.00070785 | 0.00189349 |
| PEPD | -0.1480422 | 0.00070807 | 0.00189382 |
| SELP | -0.1480141 | 0.00070975 | 0.00189807 |
| PACS1 | -0.1480065 | 0.0007102 | 0.00189903 |
| HSPC157 | 0.1479857 | 0.00071145 | 0.00190206 |
| ARHGEF35 | -0.1479844 | 0.00071153 | 0.00190206 |
| PPP2CA | 0.147976 | 0.00071203 | 0.00190316 |
| LONRF3 | -0.1479631 | 0.0007128 | 0.00190497 |
| GPR101 | 0.1478459 | 0.00071988 | 0.00192362 |
| RGS7BP | -0.147798 | 0.00072279 | 0.00193113 |
| FBXW5 | 0.1477893 | 0.00072332 | 0.00193229 |
| C10orf18 | -0.1477736 | 0.00072427 | 0.0019346 |
| TRAF7 | -0.1477168 | 0.00072774 | 0.00194359 |
| FAM200B | 0.1477113 | 0.00072808 | 0.00194414 |
| ART5 | -0.1477103 | 0.00072814 | 0.00194414 |
| CA9 | 0.1476908 | 0.00072933 | 0.00194708 |
| PLA2G5 | -0.1476756 | 0.00073027 | 0.00194932 |
| CLEC18A | -0.1476497 | 0.00073186 | 0.00195331 |
| LOC151162 | -0.1476253 | 0.00073336 | 0.00195705 |
| MKKS | 0.1476127 | 0.00073414 | 0.00195887 |
| CHD3 | -0.147609 | 0.00073437 | 0.00195922 |
| C1orf87 | -0.1475953 | 0.00073522 | 0.00196123 |
| RAB6B | -0.1475922 | 0.00073541 | 0.00196147 |
| ZNF562 | -0.1475844 | 0.00073589 | 0.0019625 |
| SEL1L2 | -0.14756 | 0.0007374 | 0.00196626 |
| FBXW8 | -0.1475377 | 0.00073878 | 0.00196969 |
| LRRN3 | -0.1475354 | 0.00073892 | 0.00196981 |
| RARB | -0.14753 | 0.00073926 | 0.00197046 |
| HLA-DQB2 | -0.1475141 | 0.00074025 | 0.00197282 |
| BRMS1L | 0.1475012 | 0.00074105 | 0.0019747 |
| FSIP1 | 0.1474455 | 0.00074453 | 0.0019837 |
| FAM161A | 0.1474417 | 0.00074477 | 0.00198408 |
| RAB3IP | 0.1474133 | 0.00074654 | 0.00198854 |
| ITGB2 | -0.1473785 | 0.00074873 | 0.00199401 |
| INMT | -0.1473775 | 0.00074879 | 0.00199401 |
| RAB1A | -0.1473664 | 0.00074949 | 0.0019956 |
| ZNF211 | -0.1473533 | 0.00075031 | 0.00199753 |
| TSNAX | 0.1473513 | 0.00075044 | 0.0019976 |
| ELOVL2 | -0.1473047 | 0.00075338 | 0.00200516 |
| HAS1 | -0.1472839 | 0.00075469 | 0.0020084 |
| KIAA1211 | -0.1472544 | 0.00075657 | 0.00201311 |
| FAM106A | -0.1472391 | 0.00075754 | 0.00201544 |
| TLE3 | -0.1472345 | 0.00075783 | 0.00201595 |
| CIAO1 | 0.1472192 | 0.0007588 | 0.00201827 |
| MAGED4B | -0.1472109 | 0.00075933 | 0.00201941 |
| NCSTN | -0.1471494 | 0.00076326 | 0.00202958 |
| SDCBP2 | -0.1471317 | 0.00076439 | 0.00203232 |
| C3orf47 | 0.1471267 | 0.00076471 | 0.00203292 |
| C15orf34 | -0.1471243 | 0.00076487 | 0.00203306 |
| NINJ2 | -0.1471001 | 0.00076642 | 0.00203691 |
| NCRNA00173 | 0.1470521 | 0.0007695 | 0.00204485 |
| CAMLG | 0.1470292 | 0.00077099 | 0.00204852 |
| TAS1R1 | -0.146964 | 0.00077521 | 0.00205946 |
| LOC100288778 | 0.1469382 | 0.00077688 | 0.00206363 |
| BIRC8 | 0.1469037 | 0.00077913 | 0.00206934 |
| MDP1 | 0.1468953 | 0.00077968 | 0.00207052 |
| IL17RD | -0.1468613 | 0.0007819 | 0.00207614 |
| C14orf135 | -0.1468502 | 0.00078263 | 0.0020778 |
| ABCB1 | -0.1468094 | 0.0007853 | 0.00208462 |
| ACAD9 | 0.1467949 | 0.00078626 | 0.00208689 |
| PML | 0.1467617 | 0.00078844 | 0.0020924 |
| C19orf50 | 0.1467539 | 0.00078896 | 0.0020935 |
| SLC39A7 | -0.1467397 | 0.00078989 | 0.0020957 |
| TMOD4 | -0.1467244 | 0.0007909 | 0.00209811 |
| TLK2 | 0.1467209 | 0.00079113 | 0.00209845 |
| RHOBTB3 | -0.1466873 | 0.00079336 | 0.00210408 |
| SND1 | -0.1466793 | 0.00079389 | 0.00210521 |
| RBBP4 | 0.1466343 | 0.00079688 | 0.00211287 |
| HDAC7 | -0.1466281 | 0.0007973 | 0.00211368 |
| SFRS18 | 0.1466212 | 0.00079775 | 0.00211461 |
| C6orf48 | 0.1466045 | 0.00079887 | 0.0021173 |
| NPTN | 0.1466029 | 0.00079897 | 0.0021173 |
| NCALD | -0.1465847 | 0.00080019 | 0.00212024 |
| LARS | 0.1465613 | 0.00080176 | 0.00212411 |
| PRSS1 | 0.1465586 | 0.00080194 | 0.00212431 |
| TNFAIP8L1 | 0.1465458 | 0.00080279 | 0.0021263 |
| MGMT | 0.1465223 | 0.00080437 | 0.0021302 |
| GRM5 | -0.1465086 | 0.00080529 | 0.00213236 |
| RASA1 | -0.1464864 | 0.00080679 | 0.00213604 |
| HIST1H1C | 0.1464836 | 0.00080698 | 0.00213625 |
| NPFF | 0.1464729 | 0.0008077 | 0.00213788 |
| ARSK | 0.1464566 | 0.0008088 | 0.00214051 |
| FTSJD1 | -0.1464429 | 0.00080972 | 0.00214269 |
| ARL5A | -0.1464323 | 0.00081044 | 0.00214416 |
| IRGQ | -0.1464315 | 0.00081049 | 0.00214416 |
| PLK3 | -0.1464089 | 0.00081202 | 0.00214792 |
| SNRPN | -0.146407 | 0.00081215 | 0.00214798 |
| TCEAL2 | -0.1463964 | 0.00081287 | 0.00214961 |
| ACAA2 | -0.1463881 | 0.00081343 | 0.00215081 |
| TPD52L3 | 0.1463767 | 0.0008142 | 0.00215257 |
| LOC338799 | 0.1463429 | 0.0008165 | 0.00215836 |
| ANXA2 | 0.1463398 | 0.00081672 | 0.00215865 |
| NRG2 | -0.1463308 | 0.00081733 | 0.00215999 |
| ZNF805 | -0.1463282 | 0.00081751 | 0.00216017 |
| ARSB | -0.1463021 | 0.00081929 | 0.0021646 |
| PPM1H | -0.1462947 | 0.00081979 | 0.00216564 |
| CINP | 0.1462748 | 0.00082116 | 0.00216895 |
| PPP1R14B | 0.1462665 | 0.00082172 | 0.00217016 |
| MSLNL | 0.1462196 | 0.00082494 | 0.00217838 |
| WDR60 | -0.1461933 | 0.00082675 | 0.00218269 |
| AJAP1 | -0.1461928 | 0.00082679 | 0.00218269 |
| ZNF791 | -0.1460612 | 0.0008359 | 0.00220624 |
| SLC39A11 | -0.1460608 | 0.00083593 | 0.00220624 |
| MC5R | -0.1460381 | 0.00083751 | 0.00221012 |
| RFK | 0.1459449 | 0.00084403 | 0.00222704 |
| TIAM1 | -0.1459389 | 0.00084445 | 0.00222786 |
| MED11 | 0.1459158 | 0.00084608 | 0.00223186 |
| MTDH | -0.1458928 | 0.0008477 | 0.0022357 |
| VANGL2 | -0.145892 | 0.00084776 | 0.0022357 |
| SH3BGRL | -0.1458875 | 0.00084807 | 0.00223623 |
| CTNNA2 | -0.1458511 | 0.00085065 | 0.00224273 |
| AMIGO3 | 0.1458374 | 0.00085161 | 0.00224499 |
| KIAA0415 | -0.1458328 | 0.00085194 | 0.00224555 |
| OR4D11 | 0.1458173 | 0.00085304 | 0.00224816 |
| SRM | 0.1458132 | 0.00085333 | 0.00224864 |
| VTCN1 | -0.1458111 | 0.00085348 | 0.00224872 |
| CLIC3 | -0.1457638 | 0.00085684 | 0.00225729 |
| SNURF | -0.1457591 | 0.00085717 | 0.00225788 |
| ZNF665 | -0.1457568 | 0.00085734 | 0.00225802 |
| FLJ12825 | 0.1457523 | 0.00085766 | 0.00225857 |
| SLC6A1 | -0.1457403 | 0.00085851 | 0.00226049 |
| psiTPTE22 | -0.1457389 | 0.00085861 | 0.00226049 |
| LRRC8A | -0.1457346 | 0.00085892 | 0.002261 |
| NEUROD1 | 0.145733 | 0.00085903 | 0.002261 |
| AKR7A2 | 0.145719 | 0.00086004 | 0.00226335 |
| RTKN2 | 0.1457157 | 0.00086027 | 0.00226368 |
| FAM27B | 0.1457113 | 0.00086058 | 0.0022642 |
| RNMT | -0.1457066 | 0.00086092 | 0.0022648 |
| B3GALT2 | -0.1456795 | 0.00086286 | 0.0022696 |
| KCTD9 | 0.1456473 | 0.00086517 | 0.00227538 |
| LOC284900 | 0.1456202 | 0.00086712 | 0.00228021 |
| NEBL | -0.1456137 | 0.00086759 | 0.00228115 |
| KIAA0317 | -0.1455935 | 0.00086905 | 0.00228468 |
| SNORD17 | 0.1455887 | 0.00086939 | 0.00228529 |
| PANK4 | -0.1455691 | 0.00087081 | 0.00228872 |
| IFNB1 | 0.1455648 | 0.00087112 | 0.00228923 |
| HIST1H4L | 0.1455581 | 0.0008716 | 0.0022902 |
| STMN2 | -0.1455416 | 0.0008728 | 0.00229304 |
| ASB4 | -0.1455232 | 0.00087413 | 0.00229625 |
| OR2G3 | 0.1455188 | 0.00087445 | 0.00229658 |
| CDK10 | 0.1455183 | 0.00087449 | 0.00229658 |
| SOX4 | -0.1454809 | 0.00087721 | 0.00230343 |
| LOC144438 | 0.1454508 | 0.0008794 | 0.00230887 |
| FAT1 | -0.1454399 | 0.00088019 | 0.0023104 |
| KCNH1 | -0.1454397 | 0.00088021 | 0.0023104 |
| CBX5 | 0.1453614 | 0.00088594 | 0.00232515 |
| SP7 | -0.1453557 | 0.00088636 | 0.00232595 |
| ZNF641 | -0.1453339 | 0.00088796 | 0.00232984 |
| PHKB | -0.1453036 | 0.0008902 | 0.0023354 |
| GNAL | -0.1452917 | 0.00089107 | 0.0023374 |
| COASY | 0.1452845 | 0.00089161 | 0.0023385 |
| CHKB-CPT1B | 0.1452794 | 0.00089198 | 0.00233918 |
| ADIPOQ | -0.1452663 | 0.00089296 | 0.00234142 |
| EEF1D | 0.1452336 | 0.00089538 | 0.00234747 |
| IQGAP1 | -0.1451796 | 0.00089938 | 0.00235767 |
| VPS36 | -0.1451407 | 0.00090229 | 0.00236497 |
| VENTX | -0.1451359 | 0.00090265 | 0.0023656 |
| ANAPC2 | 0.1451287 | 0.00090318 | 0.0023667 |
| GTF2F2 | 0.1451238 | 0.00090355 | 0.00236736 |
| FYTTD1 | -0.1451092 | 0.00090465 | 0.00236992 |
| GPR148 | 0.145103 | 0.00090511 | 0.00237083 |
| CCND1 | -0.1450865 | 0.00090635 | 0.00237377 |
| H3F3B | 0.1450663 | 0.00090786 | 0.00237742 |
| FAM92A3 | 0.1450629 | 0.00090812 | 0.00237778 |
| LOC284688 | -0.1450487 | 0.00090918 | 0.00238026 |
| RNF6 | -0.1450338 | 0.00091031 | 0.0023829 |
| EIF2B5 | 0.1450073 | 0.0009123 | 0.00238781 |
| ZNF320 | -0.1449836 | 0.00091409 | 0.00239218 |
| CAPZA1 | 0.1449693 | 0.00091517 | 0.00239448 |
| KRT34 | -0.1449689 | 0.00091521 | 0.00239448 |
| RBMXL2 | 0.1449072 | 0.00091989 | 0.00240641 |
| C2orf53 | 0.1449033 | 0.00092018 | 0.00240686 |
| POR | -0.1448915 | 0.00092109 | 0.00240892 |
| HS6ST3 | -0.1448329 | 0.00092556 | 0.0024203 |
| STS | -0.1448043 | 0.00092774 | 0.0024257 |
| PLK2 | -0.1447927 | 0.00092863 | 0.00242771 |
| PCCB | 0.1447804 | 0.00092957 | 0.00242968 |
| FBXO42 | -0.1447798 | 0.00092962 | 0.00242968 |
| TMEM126B | 0.1447625 | 0.00093095 | 0.00243283 |
| SLC1A5 | 0.1447525 | 0.00093172 | 0.00243453 |
| MXD1 | -0.1447238 | 0.00093393 | 0.00243999 |
| SRCAP | -0.1446894 | 0.00093659 | 0.0024466 |
| UBXN2A | 0.144671 | 0.00093801 | 0.00245002 |
| C1orf187 | -0.1446679 | 0.00093825 | 0.00245031 |
| WNT6 | -0.1446533 | 0.00093938 | 0.00245295 |
| EME2 | -0.1445859 | 0.00094462 | 0.00246631 |
| KCNK7 | -0.1445804 | 0.00094505 | 0.00246712 |
| ASS1 | 0.1445685 | 0.00094598 | 0.00246922 |
| DYDC2 | 0.1445643 | 0.0009463 | 0.00246975 |
| MAGI2 | -0.1445477 | 0.00094761 | 0.00247283 |
| FBXO28 | -0.1445166 | 0.00095004 | 0.00247885 |
| LRP5L | 0.1444748 | 0.00095331 | 0.00248708 |
| IFT140 | -0.1444573 | 0.00095469 | 0.00249036 |
| CSNK2A1 | -0.1444506 | 0.00095522 | 0.0024914 |
| WDR20 | -0.1444474 | 0.00095547 | 0.00249174 |
| COPZ2 | -0.1444299 | 0.00095685 | 0.00249502 |
| PSMB11 | 0.1444125 | 0.00095822 | 0.00249827 |
| GNPNAT1 | 0.1443765 | 0.00096107 | 0.00250536 |
| DSCR9 | 0.1443331 | 0.00096452 | 0.00251402 |
| ASMT | 0.1443236 | 0.00096526 | 0.00251565 |
| KRT2 | -0.1443116 | 0.00096622 | 0.00251781 |
| TRIM2 | -0.1443063 | 0.00096664 | 0.00251859 |
| KISS1R | 0.144286 | 0.00096826 | 0.00252247 |
| RBMXL3 | 0.1442767 | 0.00096901 | 0.0025241 |
| KIAA0649 | 0.1442455 | 0.00097149 | 0.00253025 |
| PRB1 | -0.1442376 | 0.00097213 | 0.00253157 |
| OR51E2 | -0.1442031 | 0.00097489 | 0.00253836 |
| VHLL | 0.1442019 | 0.00097499 | 0.00253836 |
| RASL10B | -0.1441968 | 0.00097539 | 0.00253909 |
| FARS2 | 0.1441806 | 0.0009767 | 0.00254217 |
| NDUFV3 | 0.1441644 | 0.000978 | 0.00254523 |
| ARL9 | 0.1441469 | 0.00097941 | 0.00254856 |
| PRR16 | -0.1441426 | 0.00097975 | 0.00254913 |
| RPS6KA3 | -0.144098 | 0.00098336 | 0.00255817 |
| FAM41C | 0.1440744 | 0.00098526 | 0.00256271 |
| CTPS2 | 0.1440733 | 0.00098535 | 0.00256271 |
| GPCPD1 | -0.1440404 | 0.00098803 | 0.00256933 |
| FCAMR | 0.1440258 | 0.00098921 | 0.00257207 |
| PCDHA10 | -0.1440188 | 0.00098978 | 0.00257322 |
| OR2S2 | 0.1439939 | 0.00099181 | 0.00257817 |
| TMEM219 | 0.1439906 | 0.00099208 | 0.00257854 |
| ZNF398 | -0.1439717 | 0.00099362 | 0.00258222 |
| CBX6 | -0.1439654 | 0.00099414 | 0.00258322 |
| KTN1 | -0.1439537 | 0.00099509 | 0.00258537 |
| CASD1 | -0.1438954 | 0.00099987 | 0.00259744 |
| MAPK8IP3 | -0.1438831 | 0.00100088 | 0.00259974 |
| CRYGA | 0.1438783 | 0.00100128 | 0.00260043 |
| STEAP3 | -0.1438527 | 0.00100338 | 0.00260556 |
| OR2V2 | 0.1438298 | 0.00100527 | 0.00261013 |
| HOXC9 | 0.1438138 | 0.00100659 | 0.00261305 |
| CHADL | 0.143813 | 0.00100665 | 0.00261305 |
| DDX1 | 0.143756 | 0.00101138 | 0.00262497 |
| VWC2 | -0.1437372 | 0.00101294 | 0.00262869 |
| ERCC5 | -0.143724 | 0.00101403 | 0.00263119 |
| IPO8 | -0.1437061 | 0.00101552 | 0.00263472 |
| PKHD1 | -0.1436975 | 0.00101625 | 0.00263625 |
| CLN8 | -0.1436431 | 0.00102079 | 0.0026477 |
| RPP14 | 0.1436299 | 0.0010219 | 0.00264995 |
| TBC1D17 | -0.1436296 | 0.00102192 | 0.00264995 |
| TANK | -0.1436245 | 0.00102235 | 0.00265071 |
| FGD4 | -0.1435785 | 0.00102621 | 0.00266038 |
| PFN2 | -0.1435725 | 0.00102671 | 0.00266135 |
| WFIKKN2 | -0.1435579 | 0.00102795 | 0.00266421 |
| C10orf116 | 0.1435509 | 0.00102853 | 0.0026651 |
| UGCG | -0.1435506 | 0.00102856 | 0.0026651 |
| NUTF2 | 0.1435445 | 0.00102907 | 0.00266609 |
| NKX2-4 | 0.143526 | 0.00103063 | 0.0026698 |
| PIR | 0.1435107 | 0.00103192 | 0.0026728 |
| ELOVL1 | -0.1435001 | 0.00103283 | 0.00267479 |
| C17orf37 | 0.1434618 | 0.00103607 | 0.00268284 |
| EPS8 | -0.1434538 | 0.00103675 | 0.00268426 |
| HOXA5 | 0.143393 | 0.00104192 | 0.00269731 |
| AMIGO2 | -0.143327 | 0.00104757 | 0.00271106 |
| FAM135B | -0.1433267 | 0.0010476 | 0.00271106 |
| C13orf26 | -0.1433262 | 0.00104764 | 0.00271106 |
| OR7E91P | -0.1433088 | 0.00104913 | 0.00271457 |
| DEPDC6 | -0.1433055 | 0.00104942 | 0.00271496 |
| EPC2 | -0.1432842 | 0.00105124 | 0.00271905 |
| GALC | -0.143284 | 0.00105126 | 0.00271905 |
| TEF | -0.1432804 | 0.00105157 | 0.00271934 |
| PRKD1 | -0.1432795 | 0.00105165 | 0.00271934 |
| LYPD4 | 0.1432749 | 0.00105204 | 0.00272002 |
| NT5DC1 | 0.1432694 | 0.00105252 | 0.0027209 |
| IFI35 | 0.1432436 | 0.00105474 | 0.0027263 |
| ARHGDIG | -0.1432386 | 0.00105517 | 0.00272706 |
| C19orf42 | 0.1432183 | 0.00105693 | 0.00273124 |
| TMEM176A | -0.1432097 | 0.00105767 | 0.00273281 |
| GLDN | -0.1431906 | 0.00105932 | 0.00273673 |
| C1orf163 | 0.1431778 | 0.00106044 | 0.00273926 |
| PEG3AS | -0.1431681 | 0.00106127 | 0.00274106 |
| TMEM164 | 0.1431613 | 0.00106187 | 0.00274225 |
| SAP30BP | 0.1431588 | 0.00106208 | 0.00274245 |
| PLEKHG2 | -0.1431279 | 0.00106477 | 0.00274904 |
| DOC2A | 0.1431219 | 0.00106529 | 0.00275004 |
| S100A3 | 0.1430691 | 0.00106989 | 0.00276156 |
| FMO2 | -0.1430513 | 0.00107145 | 0.00276524 |
| KL | -0.1430357 | 0.00107282 | 0.00276841 |
| UBE2B | 0.1430341 | 0.00107296 | 0.00276841 |
| PRG4 | -0.1429581 | 0.00107964 | 0.00278508 |
| NHLRC4 | -0.1429575 | 0.00107969 | 0.00278508 |
| HSD11B1 | -0.1429371 | 0.00108149 | 0.00278936 |
| CRTC1 | -0.1429062 | 0.00108422 | 0.00279604 |
| ADAP2 | -0.1429036 | 0.00108446 | 0.00279629 |
| FAM35B2 | 0.1428871 | 0.00108592 | 0.0027997 |
| LRRN2 | -0.1428817 | 0.00108639 | 0.00280057 |
| AK3 | 0.1428742 | 0.00108706 | 0.00280194 |
| SRF | -0.1428505 | 0.00108917 | 0.002807 |
| ATG5 | 0.1428307 | 0.00109093 | 0.00281117 |
| C10orf10 | -0.1428012 | 0.00109356 | 0.0028176 |
| CCDC57 | 0.1427908 | 0.00109448 | 0.00281962 |
| XRN1 | -0.1427629 | 0.00109698 | 0.00282569 |
| OR2B2 | 0.1427571 | 0.00109749 | 0.00282665 |
| TEX13A | 0.1427471 | 0.00109839 | 0.0028286 |
| LOC284749 | 0.1427185 | 0.00110095 | 0.00283484 |
| PEX11A | 0.1427121 | 0.00110153 | 0.00283596 |
| LYSMD3 | -0.1426708 | 0.00110524 | 0.00284515 |
| ACOT7 | 0.1426427 | 0.00110778 | 0.00285132 |
| DBN1 | -0.1426217 | 0.00110968 | 0.00285585 |
| DTX2 | 0.1426198 | 0.00110984 | 0.00285591 |
| TTLL6 | 0.1425854 | 0.00111296 | 0.00286356 |
| LASS3 | -0.1425731 | 0.00111408 | 0.00286607 |
| ING1 | 0.1425567 | 0.00111556 | 0.00286953 |
| KAT2B | -0.142555 | 0.00111572 | 0.00286956 |
| LOC729375 | 0.1425207 | 0.00111884 | 0.00287722 |
| CACNG3 | 0.1425171 | 0.00111917 | 0.0028777 |
| WFDC5 | -0.1425015 | 0.00112059 | 0.002881 |
| PRR12 | -0.1424253 | 0.00112756 | 0.00289854 |
| AIFM2 | 0.1424041 | 0.00112951 | 0.00290318 |
| LAMB3 | -0.1423721 | 0.00113245 | 0.00291037 |
| KIAA1486 | -0.1423671 | 0.00113291 | 0.00291118 |
| SNX20 | -0.142346 | 0.00113486 | 0.00291581 |
| PCDH9 | -0.1423225 | 0.00113704 | 0.00292098 |
| MAPK9 | 0.1423205 | 0.00113722 | 0.00292098 |
| UBASH3B | -0.1423196 | 0.0011373 | 0.00292098 |
| LOC154761 | -0.14231 | 0.00113819 | 0.00292288 |
| SLFN12 | -0.1423084 | 0.00113834 | 0.00292288 |
| CIC | -0.142272 | 0.00114171 | 0.00293118 |
| EGFL6 | -0.1422506 | 0.0011437 | 0.00293584 |
| LCAT | 0.1422493 | 0.00114382 | 0.00293584 |
| HDGFRP3 | 0.1422391 | 0.00114477 | 0.0029379 |
| COMMD8 | 0.1421681 | 0.0011514 | 0.00295454 |
| PCDH20 | -0.1421498 | 0.00115311 | 0.00295855 |
| ADCYAP1 | -0.1421359 | 0.00115441 | 0.00296151 |
| LOC728643 | 0.1421218 | 0.00115573 | 0.00296453 |
| PXK | -0.1421029 | 0.00115751 | 0.00296871 |
| ZNF708 | -0.1420384 | 0.00116359 | 0.00298392 |
| C11orf71 | -0.1420302 | 0.00116436 | 0.00298551 |
| TRMT61B | 0.1420228 | 0.00116506 | 0.00298693 |
| KRT24 | 0.142021 | 0.00116523 | 0.002987 |
| PTAFR | -0.1419902 | 0.00116814 | 0.00299379 |
| MSL3 | 0.1419899 | 0.00116818 | 0.00299379 |
| PTGER2 | -0.1419746 | 0.00116962 | 0.0029971 |
| ZNF611 | -0.1419614 | 0.00117088 | 0.00299994 |
| ANGEL1 | -0.1419584 | 0.00117116 | 0.00300028 |
| SPATS2 | 0.1419088 | 0.00117589 | 0.00301201 |
| PCSK6 | -0.1418829 | 0.00117836 | 0.00301797 |
| PEX5 | -0.1418642 | 0.00118015 | 0.00302216 |
| ZNF615 | -0.1418562 | 0.00118091 | 0.00302374 |
| HFE | -0.1418482 | 0.00118168 | 0.00302532 |
| LAMC2 | -0.1418382 | 0.00118264 | 0.00302739 |
| UGT1A1 | -0.1417534 | 0.00119079 | 0.00304782 |
| RASAL1 | -0.141752 | 0.00119092 | 0.00304782 |
| RAC3 | 0.1417392 | 0.00119216 | 0.00305061 |
| SULT1A1 | 0.1417294 | 0.00119311 | 0.00305264 |
| KIAA1045 | -0.1416986 | 0.00119609 | 0.00305989 |
| ZNF589 | 0.141669 | 0.00119896 | 0.00306684 |
| DCPS | 0.1416241 | 0.00120333 | 0.00307762 |
| P704P | -0.1416106 | 0.00120464 | 0.00308058 |
| RTP3 | 0.1415757 | 0.00120805 | 0.00308891 |
| LOC90110 | -0.1415469 | 0.00121087 | 0.0030956 |
| GRWD1 | 0.1415458 | 0.00121098 | 0.0030956 |
| FAM84A | -0.1415431 | 0.00121124 | 0.0030959 |
| UFC1 | 0.1415385 | 0.00121169 | 0.00309665 |
| CPSF3L | 0.1414791 | 0.00121754 | 0.00311119 |
| MRPS30 | 0.1414746 | 0.00121797 | 0.00311191 |
| PNPO | 0.1414604 | 0.00121938 | 0.00311511 |
| UTP3 | 0.1414502 | 0.00122038 | 0.00311727 |
| PEX3 | 0.1414461 | 0.00122079 | 0.00311792 |
| BBS7 | 0.1414176 | 0.0012236 | 0.00312472 |
| RSPO3 | -0.1414053 | 0.00122482 | 0.00312742 |
| CHRM3 | -0.1413907 | 0.00122627 | 0.00313072 |
| PLDN | 0.1413721 | 0.00122811 | 0.00313503 |
| RNF168 | -0.1413595 | 0.00122936 | 0.00313782 |
| C4orf33 | 0.1413576 | 0.00122955 | 0.00313792 |
| HAGHL | 0.1413336 | 0.00123194 | 0.00314362 |
| C5orf39 | 0.1413289 | 0.00123241 | 0.00314441 |
| PDZK1P1 | 0.1413193 | 0.00123336 | 0.00314645 |
| CLIC5 | -0.1412691 | 0.00123838 | 0.00315884 |
| B2M | 0.1412259 | 0.0012427 | 0.00316948 |
| TMEM41A | 0.1411957 | 0.00124573 | 0.0031768 |
| CXorf65 | 0.141194 | 0.00124591 | 0.00317684 |
| TGM2 | -0.141147 | 0.00125065 | 0.00318853 |
| LOC100128292 | 0.1411093 | 0.00125445 | 0.00319781 |
| PPP2R5E | -0.1411078 | 0.00125461 | 0.00319781 |
| TXNDC9 | 0.1410992 | 0.00125547 | 0.00319962 |
| CASKIN1 | -0.1410716 | 0.00125827 | 0.00320635 |
| KCNC4 | -0.1410349 | 0.00126201 | 0.00321545 |
| P2RY14 | -0.1410285 | 0.00126266 | 0.00321656 |
| CHML | 0.1410275 | 0.00126276 | 0.00321656 |
| PDCD6 | 0.1410097 | 0.00126458 | 0.00322071 |
| ZHX1 | -0.1410084 | 0.00126471 | 0.00322071 |
| PSTPIP2 | 0.1409929 | 0.00126629 | 0.00322411 |
| RALB | -0.1409922 | 0.00126636 | 0.00322411 |
| GLP2R | -0.1409863 | 0.00126696 | 0.00322523 |
| SH3BP2 | -0.1409551 | 0.00127015 | 0.00323295 |
| BET1L | -0.140925 | 0.00127324 | 0.0032404 |
| FLT3LG | 0.1409148 | 0.00127429 | 0.00324244 |
| NKIRAS2 | -0.140913 | 0.00127447 | 0.00324244 |
| ZBTB44 | -0.1409125 | 0.00127453 | 0.00324244 |
| TTLL4 | 0.1409074 | 0.00127505 | 0.00324297 |
| ADCY5 | -0.1409073 | 0.00127506 | 0.00324297 |
| CERK | -0.1408453 | 0.00128144 | 0.0032588 |
| RNF4 | -0.1408285 | 0.00128318 | 0.00326281 |
| TMEM88B | -0.1407943 | 0.00128672 | 0.00327139 |
| ZNF425 | -0.1407882 | 0.00128735 | 0.00327259 |
| NCRNA00189 | -0.1407783 | 0.00128838 | 0.00327478 |
| ARID3C | 0.1407709 | 0.00128914 | 0.00327601 |
| RBP7 | 0.1407705 | 0.00128918 | 0.00327601 |
| MPPED2 | -0.1407485 | 0.00129147 | 0.0032814 |
| UHRF2 | 0.1407429 | 0.00129206 | 0.00328248 |
| RIMS3 | -0.1407396 | 0.0012924 | 0.00328293 |
| GPR135 | -0.1407365 | 0.00129272 | 0.00328333 |
| C8ORFK29 | 0.1407327 | 0.00129311 | 0.00328392 |
| CCNI | -0.1407308 | 0.00129331 | 0.00328401 |
| PDGFA | -0.1407158 | 0.00129487 | 0.00328756 |
| DVL1 | -0.1407135 | 0.00129511 | 0.00328776 |
| TCF7L2 | -0.1407018 | 0.00129633 | 0.00329044 |
| INF2 | -0.1406889 | 0.00129769 | 0.00329346 |
| INPP5F | 0.1406834 | 0.00129826 | 0.00329451 |
| PLEKHA1 | -0.1406695 | 0.00129971 | 0.00329777 |
| NCOA5 | 0.1406544 | 0.00130129 | 0.00330136 |
| RNF207 | 0.1406513 | 0.00130161 | 0.00330177 |
| STK31 | -0.1406096 | 0.00130599 | 0.00331246 |
| CLCF1 | -0.1405945 | 0.00130758 | 0.00331606 |
| C9orf79 | 0.1405886 | 0.0013082 | 0.00331721 |
| SLC22A7 | 0.1405847 | 0.00130861 | 0.00331783 |
| ZNF207 | 0.1405777 | 0.00130935 | 0.00331929 |
| RUNDC3B | -0.1405603 | 0.00131118 | 0.00332328 |
| EVC2 | -0.1405588 | 0.00131133 | 0.00332328 |
| POMT2 | -0.1405581 | 0.00131141 | 0.00332328 |
| FAM32A | 0.1405461 | 0.00131268 | 0.00332607 |
| IKBKG | 0.1405432 | 0.00131299 | 0.00332625 |
| PRKACB | -0.1405423 | 0.00131308 | 0.00332625 |
| CDC42EP4 | 0.1405069 | 0.00131682 | 0.00333513 |
| SLC24A6 | -0.140506 | 0.00131692 | 0.00333513 |
| A2ML1 | -0.1404851 | 0.00131912 | 0.0033403 |
| CHMP1B | -0.140478 | 0.00131988 | 0.0033418 |
| ACACA | -0.140462 | 0.00132158 | 0.00334567 |
| LOC643677 | 0.1404283 | 0.00132516 | 0.00335432 |
| SLC37A4 | 0.140419 | 0.00132615 | 0.00335641 |
| LRRFIP2 | 0.1404129 | 0.00132681 | 0.00335765 |
| C6orf153 | 0.1404052 | 0.00132762 | 0.0033593 |
| PLAC1 | 0.1403997 | 0.00132821 | 0.00336036 |
| ARRDC3 | -0.1403389 | 0.00133472 | 0.00337602 |
| KRTAP5-8 | 0.1403387 | 0.00133474 | 0.00337602 |
| TUB | -0.1403001 | 0.00133888 | 0.00338606 |
| RASA4P | -0.140266 | 0.00134255 | 0.00339456 |
| CXorf40A | 0.1402658 | 0.00134257 | 0.00339456 |
| ULK3 | 0.1402615 | 0.00134303 | 0.0033953 |
| PHF21A | -0.1402517 | 0.00134409 | 0.00339754 |
| C10orf107 | -0.1402398 | 0.00134537 | 0.00340036 |
| NLRP3 | -0.1402153 | 0.00134802 | 0.00340631 |
| ZC3H6 | -0.1402149 | 0.00134806 | 0.00340631 |
| MGC72080 | 0.1401839 | 0.00135142 | 0.00341436 |
| EGFL8 | 0.1401775 | 0.00135212 | 0.0034157 |
| SLC1A2 | -0.1401723 | 0.00135268 | 0.00341669 |
| EBAG9 | 0.1401611 | 0.0013539 | 0.00341934 |
| SYN2 | -0.1401423 | 0.00135593 | 0.00342405 |
| BTBD6 | 0.1401345 | 0.00135678 | 0.00342577 |
| CYB561D1 | -0.1400987 | 0.00136069 | 0.00343519 |
| PEF1 | 0.1400861 | 0.00136206 | 0.00343822 |
| ZNF584 | 0.1400611 | 0.00136479 | 0.00344469 |
| TNIK | -0.1400305 | 0.00136815 | 0.00345273 |
| MARCKS | -0.1400194 | 0.00136937 | 0.00345537 |
| AASDHPPT | -0.1400095 | 0.00137045 | 0.00345767 |
| GPD2 | 0.1399672 | 0.0013751 | 0.00346879 |
| PRPF18 | 0.1399663 | 0.0013752 | 0.00346879 |
| LCN1 | -0.1399566 | 0.00137627 | 0.00347106 |
| POFUT2 | -0.1399424 | 0.00137784 | 0.00347457 |
| FGL1 | 0.139923 | 0.00137998 | 0.00347954 |
| TUBB2B | -0.1399201 | 0.0013803 | 0.00347991 |
| ZNF496 | -0.1399145 | 0.00138092 | 0.00348105 |
| SYPL1 | 0.1399089 | 0.00138153 | 0.00348216 |
| GJA9 | 0.139877 | 0.00138507 | 0.00349064 |
| AUP1 | 0.1398741 | 0.00138539 | 0.00349081 |
| C8orf55 | 0.1398732 | 0.00138549 | 0.00349081 |
| MCTP1 | -0.1398416 | 0.001389 | 0.00349923 |
| C10orf12 | -0.1398248 | 0.00139087 | 0.0035035 |
| INPP5K | -0.1398079 | 0.00139275 | 0.00350779 |
| NIPSNAP3A | 0.1398033 | 0.00139327 | 0.00350865 |
| CLP1 | 0.1397919 | 0.00139454 | 0.00351143 |
| PENK | -0.13977 | 0.00139698 | 0.00351713 |
| SH3BP5 | -0.1397524 | 0.00139895 | 0.00352166 |
| TLR7 | -0.1397432 | 0.00139998 | 0.00352373 |
| DUSP8 | -0.1397419 | 0.00140012 | 0.00352373 |
| RALBP1 | -0.1397188 | 0.00140272 | 0.00352981 |
| CD4 | -0.1397067 | 0.00140407 | 0.00353278 |
| LYRM7 | 0.139675 | 0.00140764 | 0.00354131 |
| PFN3 | 0.1396583 | 0.00140952 | 0.0035456 |
| WDR54 | 0.1396328 | 0.00141239 | 0.00355237 |
| ORC4L | 0.1396195 | 0.00141389 | 0.00355572 |
| GALR1 | -0.1396066 | 0.00141536 | 0.00355895 |
| EAPP | 0.1395978 | 0.00141635 | 0.00356101 |
| PIK3R5 | -0.1395621 | 0.0014204 | 0.00357075 |
| PYCRL | 0.1395513 | 0.00142162 | 0.00357337 |
| DNAJC13 | -0.1395348 | 0.0014235 | 0.00357765 |
| BPHL | 0.1394924 | 0.00142833 | 0.00358934 |
| GALT | 0.1394843 | 0.00142925 | 0.0035912 |
| ACPP | -0.1394739 | 0.00143043 | 0.00359343 |
| ZNF543 | -0.1394734 | 0.00143049 | 0.00359343 |
| PRKCG | 0.1394279 | 0.0014357 | 0.00360573 |
| FOXK1 | -0.1394275 | 0.00143575 | 0.00360573 |
| TTLL1 | -0.139384 | 0.00144074 | 0.00361781 |
| IFITM1 | 0.1393268 | 0.00144733 | 0.00363348 |
| OXCT2 | 0.1393268 | 0.00144734 | 0.00363348 |
| CNTNAP5 | -0.139299 | 0.00145055 | 0.00364108 |
| CHMP5 | 0.1392936 | 0.00145116 | 0.00364218 |
| C17orf96 | 0.1392892 | 0.00145168 | 0.00364302 |
| SPDYE7P | 0.1392857 | 0.00145208 | 0.00364358 |
| SERPINA12 | -0.1392797 | 0.00145278 | 0.00364487 |
| SCOC | 0.1392729 | 0.00145357 | 0.00364641 |
| ATP13A4 | -0.1392578 | 0.00145531 | 0.00365032 |
| DMRTB1 | 0.1392418 | 0.00145717 | 0.00365453 |
| UGT2B28 | 0.1392181 | 0.00145993 | 0.00366099 |
| SCUBE3 | -0.1392145 | 0.00146035 | 0.00366158 |
| AKAP3 | -0.1392118 | 0.00146066 | 0.00366191 |
| OXA1L | 0.1392007 | 0.00146195 | 0.0036647 |
| STAU2 | -0.1391971 | 0.00146237 | 0.00366529 |
| NTN4 | -0.1391788 | 0.00146451 | 0.0036702 |
| HCN1 | -0.139173 | 0.00146519 | 0.00367143 |
| FAM171A1 | -0.1391065 | 0.00147297 | 0.00369048 |
| TIA1 | 0.1390849 | 0.00147551 | 0.00369638 |
| KCNJ10 | 0.1390776 | 0.00147637 | 0.00369808 |
| OSM | -0.1390755 | 0.00147661 | 0.00369822 |
| PRRX2 | -0.1390453 | 0.00148017 | 0.00370668 |
| KRT17 | -0.1390267 | 0.00148237 | 0.00371172 |
| LRIG1 | -0.1390155 | 0.00148368 | 0.00371454 |
| EMID2 | -0.1389928 | 0.00148637 | 0.00372083 |
| INSIG2 | -0.1389743 | 0.00148856 | 0.00372585 |
| RYR1 | -0.1389457 | 0.00149195 | 0.00373346 |
| PHEX | -0.1389455 | 0.00149198 | 0.00373346 |
| FLI1 | -0.1389347 | 0.00149325 | 0.00373619 |
| DEFB103B | -0.1389272 | 0.00149415 | 0.00373798 |
| CRISPLD1 | -0.1389163 | 0.00149545 | 0.00374075 |
| CLEC18C | -0.1389045 | 0.00149685 | 0.00374379 |
| ZFP41 | -0.138895 | 0.00149798 | 0.00374617 |
| TPPP3 | -0.1388925 | 0.00149828 | 0.00374645 |
| PECI | 0.1388861 | 0.00149905 | 0.0037479 |
| RNASE4 | -0.1388832 | 0.00149939 | 0.00374829 |
| CD28 | -0.1388724 | 0.00150068 | 0.00375106 |
| ZNF767 | 0.1388682 | 0.00150117 | 0.00375182 |
| TAF1A | 0.138866 | 0.00150145 | 0.00375204 |
| DMD | -0.13885 | 0.00150336 | 0.00375634 |
| CHPT1 | 0.1388092 | 0.00150824 | 0.00376769 |
| PBRM1 | -0.1388089 | 0.00150827 | 0.00376769 |
| CAB39 | -0.1387958 | 0.00150985 | 0.00377116 |
| ILVBL | 0.1387481 | 0.00151559 | 0.00378503 |
| GAS6 | -0.1387224 | 0.00151869 | 0.00379229 |
| COL17A1 | -0.1386577 | 0.00152651 | 0.00381135 |
| CECR1 | -0.1385674 | 0.00153749 | 0.00383831 |
| BYSL | 0.1385625 | 0.00153809 | 0.0038391 |
| AMZ2 | 0.1385617 | 0.00153819 | 0.0038391 |
| BBS4 | 0.1385568 | 0.00153879 | 0.00384011 |
| RPGRIP1L | -0.1385523 | 0.00153934 | 0.00384101 |
| SYNGR4 | 0.1385289 | 0.00154219 | 0.00384766 |
| USHBP1 | -0.1385043 | 0.00154521 | 0.00385453 |
| EXPH5 | -0.1385034 | 0.00154533 | 0.00385453 |
| SLC45A1 | -0.1384947 | 0.00154639 | 0.00385649 |
| C19orf18 | 0.1384938 | 0.0015465 | 0.00385649 |
| SOX6 | -0.1384818 | 0.00154797 | 0.00385969 |
| ERBB2 | -0.1384798 | 0.00154822 | 0.00385983 |
| BBS1 | -0.138475 | 0.00154881 | 0.00386083 |
| WASH2P | 0.1384168 | 0.00155597 | 0.00387819 |
| SYT14 | -0.1384026 | 0.00155773 | 0.00388211 |
| HPCAL4 | -0.1383893 | 0.00155937 | 0.00388571 |
| OR1I1 | 0.1383727 | 0.00156143 | 0.00389036 |
| TRAPPC5 | 0.1383472 | 0.00156459 | 0.00389774 |
| TNR | -0.1383419 | 0.00156524 | 0.00389889 |
| FRS2 | -0.1383088 | 0.00156936 | 0.00390866 |
| SMAGP | 0.1382573 | 0.00157577 | 0.00392416 |
| FBXO11 | -0.1382508 | 0.00157658 | 0.00392569 |
| IGLL3 | 0.1382466 | 0.00157711 | 0.00392651 |
| FLJ46111 | 0.1382357 | 0.00157847 | 0.00392943 |
| FOXP3 | -0.1382255 | 0.00157975 | 0.00393184 |
| DHRS2 | 0.1382243 | 0.0015799 | 0.00393184 |
| CLCN1 | -0.138223 | 0.00158006 | 0.00393184 |
| GOLT1A | 0.1382217 | 0.00158022 | 0.00393184 |
| TMEM55A | -0.1381975 | 0.00158325 | 0.00393889 |
| C19orf23 | 0.1381412 | 0.00159032 | 0.00395599 |
| TMEM111 | 0.1381034 | 0.00159509 | 0.00396737 |
| FLCN | -0.1380989 | 0.00159567 | 0.00396831 |
| ALG10B | 0.1380741 | 0.0015988 | 0.0039756 |
| TBC1D4 | -0.1380725 | 0.001599 | 0.00397562 |
| SGSM1 | -0.1380406 | 0.00160304 | 0.00398517 |
| TATDN2 | 0.1380222 | 0.00160538 | 0.00399049 |
| BEX4 | -0.1379989 | 0.00160834 | 0.00399736 |
| IFLTD1 | -0.1379717 | 0.0016118 | 0.00400546 |
| GCKR | -0.1379478 | 0.00161485 | 0.00401255 |
| KCNK3 | -0.1379425 | 0.00161552 | 0.00401372 |
| POTEC | 0.1379217 | 0.00161819 | 0.00401985 |
| SSR2 | 0.1379135 | 0.00161923 | 0.00402195 |
| RIN3 | -0.1378937 | 0.00162177 | 0.0040276 |
| MBOAT4 | -0.1378926 | 0.00162191 | 0.0040276 |
| C21orf57 | 0.1378769 | 0.00162393 | 0.00403212 |
| DGKD | -0.1378196 | 0.00163129 | 0.0040499 |
| SPRYD4 | 0.1378075 | 0.00163285 | 0.00405329 |
| HAL | -0.1377976 | 0.00163413 | 0.00405595 |
| TFB2M | 0.1377916 | 0.0016349 | 0.00405738 |
| MAST2 | -0.1377786 | 0.00163659 | 0.00406107 |
| ZNF285 | -0.1377747 | 0.00163709 | 0.00406181 |
| HES7 | 0.1377679 | 0.00163797 | 0.00406348 |
| OSCAR | -0.1377651 | 0.00163833 | 0.00406387 |
| C4orf52 | 0.1377371 | 0.00164195 | 0.00407202 |
| OR4X1 | 0.1377357 | 0.00164214 | 0.00407202 |
| SBDS | -0.1377351 | 0.00164222 | 0.00407202 |
| CGB | -0.1377222 | 0.00164389 | 0.00407566 |
| PRR7 | 0.1377154 | 0.00164477 | 0.00407734 |
| FHOD3 | -0.1376851 | 0.00164871 | 0.00408661 |
| NR2E3 | 0.1376734 | 0.00165024 | 0.0040899 |
| USP53 | -0.137671 | 0.00165054 | 0.00409015 |
| COX18 | 0.1376244 | 0.00165663 | 0.00410473 |
| PHF3 | -0.1376208 | 0.0016571 | 0.00410539 |
| ATP6AP1 | -0.1376076 | 0.00165882 | 0.00410915 |
| SNN | -0.1376024 | 0.00165951 | 0.00411034 |
| SYN1 | -0.1375777 | 0.00166274 | 0.00411783 |
| FAM65B | -0.1375741 | 0.00166321 | 0.00411851 |
| CLK2P | 0.1375421 | 0.00166741 | 0.0041284 |
| LOC100128164 | -0.1375329 | 0.00166863 | 0.0041309 |
| SPRR1A | -0.1374931 | 0.00167387 | 0.00414337 |
| NRXN3 | -0.1374899 | 0.0016743 | 0.00414392 |
| ZDHHC21 | -0.1374407 | 0.0016808 | 0.00415951 |
| IP6K1 | -0.1374129 | 0.00168448 | 0.00416811 |
| CABIN1 | -0.1374106 | 0.00168479 | 0.00416836 |
| LOC390595 | 0.1374027 | 0.00168583 | 0.00417043 |
| DPP8 | -0.1374007 | 0.0016861 | 0.00417058 |
| CD99L2 | -0.1373968 | 0.00168662 | 0.00417136 |
| C11orf35 | 0.1373888 | 0.00168768 | 0.00417346 |
| CNPY3 | 0.1373801 | 0.00168884 | 0.00417583 |
| ZCWPW1 | 0.1373712 | 0.00169003 | 0.00417825 |
| PDLIM7 | -0.1373693 | 0.00169028 | 0.00417836 |
| SLC19A2 | -0.137349 | 0.00169299 | 0.00418434 |
| FZD9 | 0.137348 | 0.00169312 | 0.00418434 |
| JUN | -0.1373429 | 0.0016938 | 0.00418552 |
| PEBP4 | -0.1373028 | 0.00169915 | 0.00419822 |
| CASC4 | -0.1372172 | 0.00171064 | 0.00422609 |
| ARHGAP29 | -0.1372102 | 0.00171158 | 0.0042279 |
| RANGAP1 | 0.1371902 | 0.00171429 | 0.00423406 |
| XYLB | 0.1371775 | 0.00171599 | 0.00423775 |
| PPIP5K1 | 0.1371704 | 0.00171695 | 0.00423923 |
| FNBP4 | 0.13717 | 0.00171701 | 0.00423923 |
| DHRS7 | -0.1371661 | 0.00171753 | 0.00424 |
| MGC87042 | 0.1371245 | 0.00172316 | 0.00425337 |
| AFTPH | -0.1370884 | 0.00172807 | 0.00426496 |
| NCBP2 | 0.1369549 | 0.00174628 | 0.00430939 |
| AP3S1 | 0.1369401 | 0.00174832 | 0.00431389 |
| TMEM135 | -0.1368848 | 0.00175592 | 0.00433212 |
| LDB1 | -0.1368733 | 0.00175752 | 0.00433553 |
| KLRC3 | 0.136848 | 0.00176101 | 0.00434312 |
| WNT4 | -0.1368479 | 0.00176103 | 0.00434312 |
| ELFN2 | -0.1368147 | 0.00176562 | 0.00435391 |
| C10orf28 | 0.1367874 | 0.00176941 | 0.00436272 |
| MPZL1 | -0.1367807 | 0.00177033 | 0.00436446 |
| PLXNB3 | -0.1367666 | 0.00177229 | 0.00436876 |
| INO80 | 0.1367528 | 0.00177421 | 0.00437296 |
| FLVCR1 | 0.1367364 | 0.0017765 | 0.00437806 |
| TP53BP2 | -0.1367268 | 0.00177783 | 0.00438081 |
| HAPLN3 | 0.136677 | 0.00178479 | 0.00439742 |
| RTN4R | 0.1366447 | 0.00178931 | 0.00440803 |
| LOC338758 | -0.136643 | 0.00178956 | 0.0044081 |
| TAC4 | -0.136634 | 0.00179082 | 0.00441067 |
| PHLPP2 | -0.1366306 | 0.0017913 | 0.00441131 |
| AMZ1 | -0.136627 | 0.0017918 | 0.004412 |
| PSPN | 0.1366196 | 0.00179284 | 0.00441402 |
| DFFA | 0.1366 | 0.0017956 | 0.00442028 |
| OR8K1 | 0.1365491 | 0.00180278 | 0.00443741 |
| PSMC2 | 0.1365175 | 0.00180724 | 0.00444785 |
| C1orf53 | 0.136488 | 0.00181143 | 0.00445761 |
| TNNI3K | -0.1364562 | 0.00181595 | 0.00446818 |
| TNFSF12 | -0.1364414 | 0.00181805 | 0.00447281 |
| SLC16A6 | -0.136436 | 0.00181882 | 0.00447416 |
| NBPF15 | -0.1364227 | 0.00182072 | 0.00447828 |
| ZNF829 | -0.1364178 | 0.0018214 | 0.00447942 |
| ZAK | -0.1363818 | 0.00182654 | 0.00449151 |
| LOC100287718 | -0.1363763 | 0.00182733 | 0.00449291 |
| GPS1 | 0.1363705 | 0.00182817 | 0.00449441 |
| FAM25A | -0.1363545 | 0.00183045 | 0.00449927 |
| ROR2 | -0.1363536 | 0.00183059 | 0.00449927 |
| SETD4 | 0.1363329 | 0.00183355 | 0.00450601 |
| WISP3 | -0.1363286 | 0.00183417 | 0.00450698 |
| P2RY6 | -0.13631 | 0.00183683 | 0.00451297 |
| VAMP8 | 0.1363083 | 0.00183708 | 0.00451302 |
| ANKRD37 | 0.1363023 | 0.00183794 | 0.00451458 |
| USP51 | -0.1363007 | 0.00183818 | 0.00451462 |
| CARM1 | -0.1362964 | 0.00183879 | 0.00451556 |
| CCDC152 | -0.1362873 | 0.0018401 | 0.00451823 |
| WDFY4 | -0.1362397 | 0.00184696 | 0.00453454 |
| GPER | -0.1362304 | 0.0018483 | 0.00453728 |
| FGFBP2 | -0.1362203 | 0.00184977 | 0.00454032 |
| TMCC3 | -0.1361756 | 0.00185624 | 0.00455566 |
| SLC39A1 | 0.1361671 | 0.00185747 | 0.00455811 |
| ALDH3B1 | -0.1361212 | 0.00186415 | 0.00457394 |
| SFRS12 | 0.1361021 | 0.00186693 | 0.00458022 |
| ETV2 | 0.1360829 | 0.00186973 | 0.00458653 |
| TMPRSS3 | 0.1360813 | 0.00186997 | 0.00458657 |
| AVIL | -0.1360645 | 0.00187242 | 0.00459201 |
| GEMIN8 | 0.1360418 | 0.00187574 | 0.00459959 |
| HOXD10 | -0.1360251 | 0.00187819 | 0.00460504 |
| LBH | -0.1360225 | 0.00187858 | 0.00460544 |
| NTN1 | -0.1360082 | 0.00188068 | 0.00461002 |
| KATNA1 | 0.1359836 | 0.00188429 | 0.0046183 |
| SCG5 | -0.1359807 | 0.00188472 | 0.00461879 |
| KCTD8 | -0.1359792 | 0.00188494 | 0.00461879 |
| NFRKB | -0.1359644 | 0.00188711 | 0.00462353 |
| KCNJ14 | 0.1359611 | 0.0018876 | 0.00462416 |
| SLC9A1 | -0.1359586 | 0.00188797 | 0.00462453 |
| HDGFL1 | -0.1359369 | 0.00189117 | 0.00463179 |
| RPL3 | 0.135885 | 0.00189884 | 0.00465001 |
| USP24 | -0.1358814 | 0.00189938 | 0.00465078 |
| ZNF536 | -0.1358726 | 0.00190068 | 0.00465338 |
| BMP2 | -0.1358593 | 0.00190266 | 0.00465766 |
| VAMP1 | 0.135825 | 0.00190775 | 0.00466956 |
| GCN1L1 | -0.135812 | 0.00190969 | 0.00467363 |
| SNAI2 | -0.1358107 | 0.00190988 | 0.00467363 |
| RNASE10 | -0.1358075 | 0.00191035 | 0.00467422 |
| SLC2A1 | -0.1358016 | 0.00191124 | 0.00467583 |
| MGC16275 | 0.1357904 | 0.0019129 | 0.00467933 |
| C9orf163 | 0.1357286 | 0.00192214 | 0.00470137 |
| RBL1 | 0.1357163 | 0.00192398 | 0.00470517 |
| DYNC2LI1 | 0.1357151 | 0.00192416 | 0.00470517 |
| OR1K1 | 0.1356738 | 0.00193036 | 0.00471974 |
| PAWR | -0.1356701 | 0.00193092 | 0.00472056 |
| RPPH1 | 0.1356573 | 0.00193285 | 0.00472468 |
| ZNF771 | 0.1356368 | 0.00193594 | 0.00473166 |
| IFI16 | 0.1356319 | 0.00193668 | 0.0047329 |
| SRRM1 | 0.1356063 | 0.00194054 | 0.00474177 |
| ABHD15 | -0.1355911 | 0.00194284 | 0.00474649 |
| RAB5C | -0.1355904 | 0.00194294 | 0.00474649 |
| APBB3 | 0.1355603 | 0.00194751 | 0.00475706 |
| 8-Sep | -0.1355459 | 0.00194968 | 0.00476179 |
| GNAI2 | -0.1355359 | 0.00195119 | 0.00476491 |
| KAZ | -0.1355337 | 0.00195154 | 0.00476518 |
| KCTD7 | -0.135527 | 0.00195255 | 0.00476708 |
| NCAM2 | -0.1355231 | 0.00195315 | 0.00476795 |
| ANKZF1 | 0.1355181 | 0.00195391 | 0.00476923 |
| DCHS2 | -0.1355028 | 0.00195624 | 0.00477433 |
| LRRC40 | 0.1354963 | 0.00195722 | 0.00477615 |
| RAB3D | -0.1354921 | 0.00195786 | 0.00477715 |
| TXNDC15 | -0.1354796 | 0.00195977 | 0.00478123 |
| ACTL9 | 0.1354699 | 0.00196125 | 0.00478426 |
| ECD | 0.1354424 | 0.00196544 | 0.0047939 |
| TNFRSF6B | 0.1354406 | 0.00196573 | 0.00479402 |
| RNASEH1 | 0.1354369 | 0.00196629 | 0.00479481 |
| FAM162B | -0.1354325 | 0.00196697 | 0.00479588 |
| MYO3A | 0.1354045 | 0.00197125 | 0.00480575 |
| ACPT | -0.1353805 | 0.00197494 | 0.00481415 |
| CNGA4 | -0.1353748 | 0.0019758 | 0.00481568 |
| HMBOX1 | -0.1353591 | 0.00197822 | 0.00482099 |
| S100PBP | 0.135348 | 0.00197992 | 0.00482455 |
| PELI1 | -0.1353463 | 0.00198019 | 0.00482463 |
| C1orf114 | -0.1353396 | 0.00198122 | 0.00482655 |
| LMLN | -0.1353203 | 0.00198419 | 0.0048332 |
| GHRHR | 0.1353091 | 0.00198592 | 0.00483682 |
| SERPIND1 | -0.135248 | 0.00199537 | 0.00485925 |
| ARRB1 | -0.135238 | 0.00199692 | 0.00486244 |
| PKNOX1 | 0.1352244 | 0.00199904 | 0.00486701 |
| C16orf70 | -0.135216 | 0.00200033 | 0.00486958 |
| TCF12 | -0.1352117 | 0.002001 | 0.00487062 |
| PCNP | 0.1351949 | 0.00200363 | 0.0048762 |
| SYBU | -0.1351939 | 0.00200378 | 0.0048762 |
| S100A11 | 0.135165 | 0.00200828 | 0.00488657 |
| C2orf83 | 0.1351525 | 0.00201022 | 0.0048907 |
| OR6Y1 | 0.13514 | 0.00201218 | 0.00489486 |
| FAM35A | 0.1350895 | 0.00202007 | 0.00491348 |
| OR5J2 | 0.135083 | 0.00202109 | 0.00491537 |
| OR3A4 | 0.1350696 | 0.0020232 | 0.00491989 |
| FRMD7 | -0.1350655 | 0.00202384 | 0.00492034 |
| C21orf99 | 0.1350654 | 0.00202387 | 0.00492034 |
| ZNF431 | -0.1350431 | 0.00202737 | 0.00492826 |
| DLL1 | -0.1350404 | 0.00202779 | 0.00492869 |
| GTPBP5 | 0.1350267 | 0.00202994 | 0.00493331 |
| LOC646999 | -0.1349604 | 0.00204041 | 0.00495816 |
| CD300E | -0.1349286 | 0.00204544 | 0.00496979 |
| FAM113A | 0.1348851 | 0.00205236 | 0.0049854 |
| ARHGEF9 | -0.1348844 | 0.00205246 | 0.0049854 |
| TUBGCP2 | 0.1348835 | 0.00205261 | 0.0049854 |
| KRTAP12-2 | 0.1348722 | 0.00205441 | 0.00498917 |
| GRIK4 | -0.1348679 | 0.00205509 | 0.00499024 |
| NRTN | 0.1348644 | 0.00205564 | 0.00499045 |
| NR1D1 | -0.1348642 | 0.00205568 | 0.00499045 |
| CNTROB | 0.1348583 | 0.00205661 | 0.00499212 |
| UBE2D4 | -0.1348443 | 0.00205885 | 0.00499694 |
| NUP133 | 0.134837 | 0.00206002 | 0.00499918 |
| LOC220429 | -0.1348244 | 0.00206203 | 0.00500346 |
| PYGO2 | 0.1347811 | 0.00206895 | 0.00501965 |
| VSIG4 | -0.1347611 | 0.00207216 | 0.00502683 |
| SPP1 | -0.1347584 | 0.00207259 | 0.00502727 |
| SLC35E4 | -0.1347564 | 0.00207292 | 0.00502748 |
| PLS1 | 0.1347324 | 0.00207677 | 0.0050362 |
| STK11IP | -0.1347286 | 0.00207738 | 0.00503707 |
| GSTTP2 | 0.1346845 | 0.00208449 | 0.00505371 |
| C9orf85 | 0.1346812 | 0.00208502 | 0.00505437 |
| TFPI | -0.1346716 | 0.00208657 | 0.00505753 |
| SARM1 | -0.1346606 | 0.00208834 | 0.00506121 |
| POLR3G | 0.1346301 | 0.00209327 | 0.00507207 |
| BCL2L14 | 0.1346298 | 0.00209332 | 0.00507207 |
| SWAP70 | -0.1346208 | 0.00209479 | 0.00507502 |
| TMC7 | -0.1346045 | 0.00209742 | 0.00508079 |
| DNASE2B | -0.1345935 | 0.00209921 | 0.0050845 |
| ZNF417 | -0.1345875 | 0.00210018 | 0.00508625 |
| PRKAB1 | 0.1345836 | 0.00210082 | 0.00508719 |
| PMCHL2 | 0.1345758 | 0.00210208 | 0.00508963 |
| TRPV1 | 0.1345608 | 0.00210452 | 0.00509492 |
| KCTD17 | -0.1345406 | 0.00210782 | 0.00510228 |
| PCNXL3 | -0.1344688 | 0.00211954 | 0.00513005 |
| KIF2B | 0.1344385 | 0.00212451 | 0.00514146 |
| PLD2 | -0.1344272 | 0.00212637 | 0.00514534 |
| TRIM42 | 0.1344124 | 0.0021288 | 0.0051506 |
| LETM1 | -0.1343965 | 0.00213143 | 0.00515609 |
| RUNDC3A | -0.1343956 | 0.00213158 | 0.00515609 |
| ZNF524 | 0.134368 | 0.00213612 | 0.00516646 |
| P4HB | -0.134356 | 0.00213809 | 0.00517061 |
| ZNF22 | 0.1343435 | 0.00214016 | 0.00517499 |
| NPR3 | -0.1343383 | 0.00214103 | 0.00517646 |
| UBXN4 | -0.1343247 | 0.00214327 | 0.00518125 |
| GNG3 | 0.1342924 | 0.00214863 | 0.00519359 |
| OR4C3 | 0.1342801 | 0.00215066 | 0.00519788 |
| IYD | -0.1342673 | 0.00215279 | 0.0052024 |
| MPP7 | -0.1342296 | 0.00215907 | 0.00521696 |
| FPR1 | -0.1342137 | 0.00216172 | 0.00522272 |
| CACNB2 | -0.134175 | 0.00216818 | 0.00523772 |
| SLC35F2 | -0.134129 | 0.00217588 | 0.00525568 |
| MYD88 | -0.1340325 | 0.00219213 | 0.0052943 |
| SLC5A4 | -0.134015 | 0.00219509 | 0.00530082 |
| RMRP | 0.1340042 | 0.00219691 | 0.00530457 |
| BMPR1B | -0.1339994 | 0.00219772 | 0.00530591 |
| GABRR1 | -0.1339946 | 0.00219853 | 0.00530722 |
| TMEM84 | 0.1339779 | 0.00220136 | 0.00531342 |
| MORN3 | 0.1339697 | 0.00220277 | 0.00531617 |
| FAM27A | 0.1339616 | 0.00220414 | 0.00531885 |
| LYPLAL1 | 0.1339187 | 0.00221143 | 0.0053358 |
| ZNF407 | -0.1339147 | 0.0022121 | 0.00533679 |
| SLC5A9 | -0.1338886 | 0.00221656 | 0.00534691 |
| EFTUD2 | 0.1338788 | 0.00221823 | 0.00534969 |
| KCNQ3 | -0.1338787 | 0.00221825 | 0.00534969 |
| PLAC8 | 0.1337854 | 0.00223424 | 0.00538762 |
| HIST2H2AB | 0.1337684 | 0.00223716 | 0.005394 |
| C4orf12 | -0.1337574 | 0.00223904 | 0.0053979 |
| CPS1 | -0.1337516 | 0.00224004 | 0.00539967 |
| EPHA5 | -0.1337453 | 0.00224114 | 0.00540167 |
| SLC39A4 | 0.1337417 | 0.00224176 | 0.00540252 |
| KLB | 0.133739 | 0.00224222 | 0.00540297 |
| CD58 | 0.1337201 | 0.00224548 | 0.00541018 |
| FRZB | -0.1337135 | 0.00224662 | 0.0054123 |
| ZNF334 | -0.1337064 | 0.00224785 | 0.00541461 |
| LAMB4 | -0.1336893 | 0.0022508 | 0.00542107 |
| PM20D1 | -0.133646 | 0.00225831 | 0.0054385 |
| CLRN2 | 0.1335866 | 0.00226864 | 0.00546273 |
| UPF3A | 0.1335821 | 0.00226942 | 0.00546396 |
| FCGR2A | -0.1335695 | 0.00227162 | 0.00546858 |
| SYP | 0.133568 | 0.00227188 | 0.00546858 |
| C17orf107 | -0.133565 | 0.00227241 | 0.00546919 |
| FNBP1L | -0.1335551 | 0.00227414 | 0.0054727 |
| NFKBIB | 0.1335428 | 0.00227628 | 0.0054772 |
| GGCX | -0.1334933 | 0.00228496 | 0.00549742 |
| PACSIN1 | -0.1334833 | 0.00228672 | 0.00550099 |
| HTR2B | -0.1334809 | 0.00228714 | 0.00550135 |
| TUBE1 | 0.1334769 | 0.00228784 | 0.00550238 |
| ABHD12 | 0.1334693 | 0.00228917 | 0.00550472 |
| RRM2B | -0.1334683 | 0.00228936 | 0.00550472 |
| ALKBH1 | 0.133419 | 0.00229803 | 0.00552411 |
| C20orf108 | -0.1334182 | 0.00229817 | 0.00552411 |
| ACOT8 | 0.1334178 | 0.00229824 | 0.00552411 |
| RARRES3 | 0.1334026 | 0.00230092 | 0.00552988 |
| CPO | -0.13339 | 0.00230315 | 0.00553458 |
| RPN1 | 0.1333563 | 0.00230911 | 0.00554826 |
| ZBTB9 | 0.1333341 | 0.00231305 | 0.00555705 |
| WNT10B | 0.1333314 | 0.00231352 | 0.00555752 |
| LOC100192426 | 0.1333216 | 0.00231527 | 0.00556106 |
| RSAD1 | 0.1333153 | 0.00231638 | 0.00556281 |
| CHCHD10 | 0.1333144 | 0.00231655 | 0.00556281 |
| TPBG | -0.133271 | 0.00232426 | 0.00558066 |
| ABHD6 | -0.1332615 | 0.00232595 | 0.00558406 |
| RPL13AP6 | 0.1332451 | 0.00232889 | 0.00559045 |
| HHIPL2 | -0.1332364 | 0.00233043 | 0.00559348 |
| MIA3 | -0.1332262 | 0.00233227 | 0.00559722 |
| APOB | -0.1332203 | 0.00233333 | 0.00559909 |
| ZMYM6 | -0.1331505 | 0.00234582 | 0.00562842 |
| CDADC1 | 0.1331245 | 0.00235051 | 0.00563876 |
| CACNA2D3 | -0.1331235 | 0.00235069 | 0.00563876 |
| CFDP1 | 0.1331124 | 0.00235269 | 0.00564289 |
| C6orf217 | -0.1330613 | 0.00236191 | 0.0056643 |
| ZNF668 | -0.1330457 | 0.00236474 | 0.00567042 |
| MOSPD2 | -0.1330434 | 0.00236515 | 0.00567074 |
| EIF4ENIF1 | -0.1330308 | 0.00236743 | 0.00567554 |
| ERAP2 | 0.1330254 | 0.00236842 | 0.00567723 |
| DDO | -0.1329801 | 0.00237663 | 0.00569602 |
| RFC1 | 0.1329791 | 0.00237683 | 0.00569602 |
| TRIM65 | 0.1329734 | 0.00237785 | 0.0056978 |
| TMEM38A | -0.1329646 | 0.00237946 | 0.00570099 |
| SLC23A3 | 0.1329214 | 0.00238734 | 0.00571918 |
| TMEM206 | 0.1329182 | 0.00238792 | 0.0057199 |
| FIBCD1 | -0.1329149 | 0.00238852 | 0.00572065 |
| ZNF702P | -0.1329076 | 0.00238985 | 0.00572316 |
| COMMD1 | 0.1329 | 0.00239124 | 0.00572581 |
| NCRNA00176 | 0.1327867 | 0.00241204 | 0.00577492 |
| PDIA3P | 0.1327846 | 0.00241244 | 0.0057752 |
| TGFBR1 | -0.1327733 | 0.00241453 | 0.0057795 |
| COMMD10 | 0.1327627 | 0.00241647 | 0.00578296 |
| NPPC | 0.1327623 | 0.00241654 | 0.00578296 |
| GSTA4 | -0.1327545 | 0.00241799 | 0.00578573 |
| RAB11FIP4 | -0.1327524 | 0.00241838 | 0.00578597 |
| BPNT1 | 0.1327216 | 0.00242407 | 0.00579889 |
| LGI2 | -0.13272 | 0.00242436 | 0.00579891 |
| KLHL7 | -0.1327002 | 0.00242805 | 0.00580704 |
| DDX54 | 0.1326658 | 0.00243443 | 0.00582161 |
| DTHD1 | -0.1326516 | 0.00243706 | 0.00582722 |
| CYGB | -0.1326361 | 0.00243996 | 0.00583346 |
| C14orf1 | 0.1326161 | 0.00244368 | 0.00584165 |
| ANKDD1A | 0.1326039 | 0.00244595 | 0.00584639 |
| BHLHE40 | -0.1325993 | 0.00244682 | 0.00584778 |
| AFM | 0.1325527 | 0.00245553 | 0.0058679 |
| ICAM4 | 0.1325466 | 0.00245668 | 0.00586994 |
| NANOS2 | 0.1325257 | 0.0024606 | 0.00587861 |
| CHN2 | -0.13251 | 0.00246355 | 0.00588496 |
| RBPMS | -0.1324951 | 0.00246635 | 0.00589095 |
| LILRA2 | -0.1324867 | 0.00246793 | 0.00589404 |
| POU6F2 | -0.1324718 | 0.00247074 | 0.00589991 |
| KIF3B | -0.1324705 | 0.00247098 | 0.00589991 |
| TERC | 0.1324356 | 0.00247757 | 0.00591496 |
| LRRK1 | -0.1324327 | 0.00247811 | 0.00591555 |
| MYNN | 0.1324178 | 0.00248093 | 0.00592158 |
| CRISP3 | -0.1324073 | 0.00248291 | 0.0059256 |
| USP43 | -0.1323694 | 0.00249009 | 0.00594204 |
| VLDLR | -0.1323656 | 0.00249083 | 0.00594308 |
| RNASE1 | -0.132361 | 0.0024917 | 0.00594447 |
| ACP5 | -0.1323566 | 0.00249253 | 0.00594574 |
| CLEC4G | -0.1323242 | 0.00249869 | 0.0059591 |
| SCARNA22 | 0.132324 | 0.00249872 | 0.0059591 |
| IGSF9 | 0.1323191 | 0.00249966 | 0.00596063 |
| RAD50 | -0.1322972 | 0.00250383 | 0.00596987 |
| ITGB8 | -0.132288 | 0.00250558 | 0.00597335 |
| TRIM58 | -0.1322622 | 0.00251051 | 0.00598439 |
| RBMS2 | -0.1322485 | 0.00251314 | 0.00598995 |
| LOC150197 | 0.1322396 | 0.00251484 | 0.00599295 |
| PTGES | 0.1322388 | 0.00251499 | 0.00599295 |
| ARAP3 | -0.1322342 | 0.00251587 | 0.00599433 |
| OR2F2 | 0.1322214 | 0.00251832 | 0.00599883 |
| ORAOV1 | 0.1322213 | 0.00251835 | 0.00599883 |
| LRTOMT | 0.1321605 | 0.00253003 | 0.00602594 |
| ADD3 | -0.1321466 | 0.00253272 | 0.00603161 |
| COG5 | -0.1321276 | 0.00253636 | 0.00603959 |
| C17orf56 | 0.1320842 | 0.00254475 | 0.00605885 |
| PDE10A | -0.1320761 | 0.00254632 | 0.00606187 |
| DPY19L2P4 | 0.132074 | 0.00254674 | 0.00606214 |
| YKT6 | -0.1320609 | 0.00254927 | 0.00606745 |
| ZNF596 | 0.132058 | 0.00254985 | 0.00606811 |
| SCARNA9 | 0.1320155 | 0.00255808 | 0.006087 |
| PRKG2 | -0.1319974 | 0.00256161 | 0.00609466 |
| CHM | -0.1319891 | 0.00256322 | 0.00609778 |
| PCBP3 | -0.1319652 | 0.00256789 | 0.00610816 |
| KLF2 | -0.1319593 | 0.00256904 | 0.00611018 |
| NLRP13 | 0.1319261 | 0.00257552 | 0.00612488 |
| TUBA1C | 0.1319076 | 0.00257916 | 0.0061328 |
| KIAA1107 | -0.1318928 | 0.00258205 | 0.00613895 |
| MOGAT2 | -0.1318874 | 0.00258311 | 0.00614074 |
| SDF2 | 0.1318712 | 0.0025863 | 0.00614761 |
| TBXAS1 | -0.1318546 | 0.00258957 | 0.00615465 |
| LIMD2 | 0.1318509 | 0.00259029 | 0.00615563 |
| LOC158696 | -0.1318456 | 0.00259134 | 0.00615741 |
| PLD4 | -0.1318402 | 0.00259239 | 0.00615901 |
| HIST1H1A | 0.131839 | 0.00259262 | 0.00615901 |
| RCBTB2 | -0.1318368 | 0.00259306 | 0.00615931 |
| NXF2 | -0.1318248 | 0.00259542 | 0.0061642 |
| KEL | 0.1318222 | 0.00259593 | 0.00616469 |
| DCC | -0.1318141 | 0.00259753 | 0.00616776 |
| SIPA1L3 | -0.1318072 | 0.0025989 | 0.00617029 |
| TBCB | 0.1317454 | 0.00261111 | 0.00619855 |
| NME1-NME2 | 0.1317166 | 0.00261683 | 0.00621139 |
| FOXG1 | 0.1317144 | 0.00261726 | 0.00621169 |
| CSNK1A1P | -0.1317057 | 0.002619 | 0.00621507 |
| IL15 | 0.1316988 | 0.00262037 | 0.00621761 |
| AFF2 | -0.1316727 | 0.00262556 | 0.00622918 |
| EMB | -0.1316657 | 0.00262696 | 0.00623176 |
| WBSCR16 | 0.1316364 | 0.00263279 | 0.00624486 |
| SORBS2 | -0.1316327 | 0.00263354 | 0.00624591 |
| LAMA3 | -0.1316219 | 0.00263569 | 0.00625027 |
| TMEM35 | -0.1315989 | 0.0026403 | 0.00626046 |
| C1orf69 | 0.1315637 | 0.00264735 | 0.00627644 |
| GPR149 | -0.1315153 | 0.00265707 | 0.00629875 |
| CAPN6 | -0.1315028 | 0.0026596 | 0.00630401 |
| THAP8 | -0.1314836 | 0.00266347 | 0.00631243 |
| CORO7 | -0.1314819 | 0.00266381 | 0.00631251 |
| BBX | -0.1314489 | 0.00267047 | 0.00632754 |
| ZSCAN12P1 | -0.131447 | 0.00267085 | 0.0063277 |
| ELOVL3 | 0.1314436 | 0.00267155 | 0.0063286 |
| FOXA1 | 0.131436 | 0.00267309 | 0.00633151 |
| TAS1R3 | -0.1314277 | 0.00267477 | 0.00633409 |
| IL11 | -0.1314275 | 0.0026748 | 0.00633409 |
| FGR | -0.1313287 | 0.00269488 | 0.00638088 |
| GZMA | 0.1313139 | 0.00269791 | 0.00638673 |
| SEMA3A | -0.1313135 | 0.00269798 | 0.00638673 |
| TMEM27 | 0.1313076 | 0.00269919 | 0.00638884 |
| LOC401093 | -0.131305 | 0.00269973 | 0.00638936 |
| GNA13 | -0.1312843 | 0.00270395 | 0.00639749 |
| UBE2V1 | -0.1312835 | 0.00270412 | 0.00639749 |
| SEMA4C | -0.1312835 | 0.00270412 | 0.00639749 |
| DALRD3 | 0.1312701 | 0.00270688 | 0.00640327 |
| AHRR | -0.1312673 | 0.00270744 | 0.00640384 |
| LRRC49 | 0.131257 | 0.00270956 | 0.00640753 |
| CWC15 | 0.1312566 | 0.00270963 | 0.00640753 |
| AUH | 0.1312433 | 0.00271236 | 0.00641323 |
| B4GALT5 | -0.1312242 | 0.00271628 | 0.00642174 |
| C1orf170 | -0.1312038 | 0.00272048 | 0.00643092 |
| PRR23B | 0.1311994 | 0.00272138 | 0.00643229 |
| IVNS1ABP | -0.1311702 | 0.0027274 | 0.00644577 |
| USH1G | -0.1311626 | 0.00272896 | 0.00644869 |
| LYG1 | 0.1311571 | 0.0027301 | 0.00645063 |
| C1orf26 | -0.1311538 | 0.00273078 | 0.00645149 |
| CRB1 | -0.1311421 | 0.00273319 | 0.00645642 |
| PIGA | 0.1311328 | 0.00273511 | 0.0064602 |
| ZNF69 | -0.1311111 | 0.00273961 | 0.00647008 |
| MAEA | -0.131104 | 0.00274108 | 0.00647279 |
| MR1 | -0.1310874 | 0.00274452 | 0.0064801 |
| LIN7C | 0.1310859 | 0.00274482 | 0.0064801 |
| FAM161B | -0.1310569 | 0.00275084 | 0.00649306 |
| ZNF418 | -0.1310564 | 0.00275095 | 0.00649306 |
| TRAPPC9 | -0.1310354 | 0.00275531 | 0.00650259 |
| DGKE | 0.1310222 | 0.00275807 | 0.00650768 |
| PMS2L2 | 0.131022 | 0.00275812 | 0.00650768 |
| OR4D10 | 0.1309798 | 0.00276691 | 0.00652766 |
| KCNA3 | -0.1309728 | 0.00276838 | 0.00653037 |
| GYLTL1B | -0.13097 | 0.00276895 | 0.00653096 |
| C14orf109 | 0.1309683 | 0.00276931 | 0.00653104 |
| PRSS36 | 0.1309436 | 0.00277448 | 0.00654247 |
| ALG1L | 0.1309218 | 0.00277905 | 0.00655193 |
| LCE3C | -0.1309214 | 0.00277915 | 0.00655193 |
| TYK2 | 0.1308821 | 0.00278738 | 0.00657059 |
| ANKRD16 | 0.1308587 | 0.00279231 | 0.00658142 |
| DSTN | -0.1308428 | 0.00279567 | 0.00658859 |
| GAD1 | 0.1308258 | 0.00279925 | 0.00659493 |
| CYLC2 | 0.1308258 | 0.00279926 | 0.00659493 |
| ARL5B | -0.1308254 | 0.00279935 | 0.00659493 |
| CYP4Z2P | -0.1308231 | 0.00279983 | 0.00659529 |
| FAM22G | 0.1308056 | 0.00280352 | 0.00660322 |
| IGF2BP2 | -0.1308021 | 0.00280425 | 0.00660418 |
| ZNF548 | -0.1307879 | 0.00280727 | 0.00661051 |
| TMEM209 | 0.1307651 | 0.0028121 | 0.00662108 |
| LYPD1 | -0.1307636 | 0.00281242 | 0.00662108 |
| FEZF2 | 0.1307254 | 0.00282053 | 0.00663941 |
| OLR1 | -0.1307214 | 0.00282136 | 0.0066406 |
| ADAM8 | 0.1306501 | 0.00283657 | 0.00667561 |
| NOP2 | 0.1306412 | 0.00283849 | 0.00667934 |
| DGCR8 | 0.1306052 | 0.00284619 | 0.00669668 |
| PAR-SN | -0.1305953 | 0.00284831 | 0.00670089 |
| CST4 | -0.13058 | 0.0028516 | 0.00670784 |
| DMRTC2 | 0.1305681 | 0.00285414 | 0.00671304 |
| SLC11A1 | -0.1305273 | 0.00286293 | 0.00673294 |
| KLHDC4 | 0.1305088 | 0.0028669 | 0.00674149 |
| SACS | -0.1305039 | 0.00286796 | 0.0067432 |
| CD70 | 0.1304966 | 0.00286955 | 0.00674614 |
| NEURL | 0.1304892 | 0.00287115 | 0.00674912 |
| NR2C2 | -0.1304512 | 0.00287938 | 0.00676766 |
| PHF17 | 0.1304431 | 0.00288113 | 0.00677098 |
| GPRASP2 | -0.1304237 | 0.00288532 | 0.00678006 |
| USP21 | 0.1304197 | 0.0028862 | 0.00678134 |
| PSPH | 0.1303532 | 0.00290066 | 0.00681441 |
| BLMH | 0.1303519 | 0.00290096 | 0.00681441 |
| FABP5 | -0.1303126 | 0.00290954 | 0.00683376 |
| GTF2IRD2P1 | -0.1303105 | 0.00291 | 0.00683406 |
| PTEN | -0.1302914 | 0.00291417 | 0.00684307 |
| NR3C1 | -0.1302784 | 0.00291703 | 0.00684897 |
| CXCR1 | -0.1302737 | 0.00291806 | 0.00685059 |
| ZNF10 | 0.1302422 | 0.00292496 | 0.006866 |
| SHISA9 | -0.1301534 | 0.00294454 | 0.00691043 |
| OR5L1 | 0.1301532 | 0.00294458 | 0.00691043 |
| TGM3 | -0.1301513 | 0.00294501 | 0.00691064 |
| HS3ST6 | -0.130149 | 0.00294551 | 0.00691102 |
| ZSWIM6 | -0.130123 | 0.00295127 | 0.00692373 |
| KRTAP10-12 | 0.1301123 | 0.00295364 | 0.00692849 |
| GCOM1 | -0.1300954 | 0.00295739 | 0.00693646 |
| C7orf46 | 0.1300766 | 0.00296157 | 0.00694546 |
| HIST1H2BB | 0.1300087 | 0.00297669 | 0.0069801 |
| C5orf15 | 0.1300061 | 0.00297726 | 0.00698064 |
| ST3GAL6 | -0.1299761 | 0.00298398 | 0.00699557 |
| JSRP1 | -0.129943 | 0.00299138 | 0.00701212 |
| ATG7 | 0.1299184 | 0.00299691 | 0.00702425 |
| GNG11 | -0.129902 | 0.0030006 | 0.0070321 |
| SMC5 | 0.129891 | 0.00300308 | 0.00703709 |
| SLC6A8 | 0.1298822 | 0.00300506 | 0.0070409 |
| MADD | -0.1298655 | 0.00300881 | 0.00704838 |
| F13B | 0.1298649 | 0.00300895 | 0.00704838 |
| C2orf52 | 0.1298611 | 0.0030098 | 0.00704956 |
| YTHDF2 | 0.1298496 | 0.0030124 | 0.00705483 |
| OR5A1 | 0.1298368 | 0.00301529 | 0.00706078 |
| NAT9 | 0.1298288 | 0.00301711 | 0.00706421 |
| SCNN1B | -0.1298049 | 0.00302251 | 0.00707603 |
| C1orf157 | 0.1298022 | 0.00302312 | 0.00707605 |
| MMP19 | -0.1298003 | 0.00302355 | 0.00707605 |
| MOV10L1 | -0.1298002 | 0.00302357 | 0.00707605 |
| KIAA0947 | -0.1297885 | 0.00302622 | 0.00708144 |
| TOX | -0.1297793 | 0.00302832 | 0.00708552 |
| OGDHL | 0.1297626 | 0.00303211 | 0.00709357 |
| NBLA00301 | -0.1297401 | 0.00303722 | 0.0071047 |
| SNX14 | -0.1297106 | 0.00304393 | 0.00711957 |
| PIGH | 0.129698 | 0.00304679 | 0.00712544 |
| SRCRB4D | 0.1296924 | 0.00304809 | 0.00712764 |
| MAGIX | 0.1296599 | 0.0030555 | 0.00714415 |
| TBXA2R | -0.1296474 | 0.00305837 | 0.00715002 |
| OR10J3 | 0.1296262 | 0.00306321 | 0.00716052 |
| LBX1 | -0.1296145 | 0.0030659 | 0.00716598 |
| FBLN1 | -0.129599 | 0.00306946 | 0.00717346 |
| C1orf161 | -0.1295758 | 0.00307479 | 0.00718507 |
| NCBP1 | 0.1295314 | 0.00308502 | 0.00720814 |
| C9orf130 | 0.1295294 | 0.00308548 | 0.00720838 |
| PLS3 | -0.1295264 | 0.00308617 | 0.00720917 |
| TXNDC5 | -0.1295222 | 0.00308713 | 0.00721027 |
| NPW | 0.129521 | 0.00308741 | 0.00721027 |
| HP1BP3 | 0.1295197 | 0.00308771 | 0.00721027 |
| SEC23B | -0.1295174 | 0.00308825 | 0.00721068 |
| NFS1 | 0.1295076 | 0.00309051 | 0.00721512 |
| AMTN | -0.129422 | 0.00311035 | 0.0072606 |
| MAP3K13 | -0.1294126 | 0.00311253 | 0.00726486 |
| CNKSR1 | 0.12939 | 0.00311778 | 0.00727627 |
| FAM135A | -0.1293852 | 0.00311891 | 0.00727805 |
| SLC10A5 | 0.1293639 | 0.00312387 | 0.0072888 |
| ZNF234 | -0.1293402 | 0.00312941 | 0.00730087 |
| ZBTB32 | 0.1293374 | 0.00313005 | 0.00730152 |
| AQP11 | -0.1293218 | 0.00313369 | 0.00730917 |
| TAP1 | 0.1293131 | 0.00313573 | 0.00731308 |
| FNDC8 | 0.1293047 | 0.00313771 | 0.00731684 |
| EIF2S1 | 0.1292887 | 0.00314146 | 0.00732474 |
| TSSC4 | 0.1292659 | 0.00314681 | 0.00733638 |
| SLC39A5 | 0.1292595 | 0.00314832 | 0.00733904 |
| OR10AD1 | 0.129256 | 0.00314914 | 0.0073401 |
| TRIL | -0.1292511 | 0.00315028 | 0.00734191 |
| C1orf127 | -0.1292441 | 0.00315192 | 0.00734488 |
| LOC572558 | -0.1292407 | 0.00315273 | 0.00734594 |
| HSPBAP1 | 0.1292296 | 0.00315534 | 0.00735117 |
| C11orf67 | 0.1292259 | 0.00315621 | 0.00735234 |
| PRDM13 | 0.1291735 | 0.00316858 | 0.00738031 |
| CENPP | 0.12915 | 0.00317412 | 0.00739235 |
| ABAT | -0.1291447 | 0.00317538 | 0.00739442 |
[truncated: 563,529 more chars]
